# Remote homology clustering identifies lowly conserved families of effector proteins in plant-pathogenic fungi

**DOI:** 10.1099/mgen.0.000637

**Published:** 2021-09-01

**Authors:** Darcy A. B. Jones, Paula M. Moolhuijzen, James K. Hane

**Affiliations:** ^1^​ Centre for Crop & Disease Management, School of Molecular & Life Sciences, Curtin University, Perth, Australia; ^2^​ Curtin Institute for Computation, Curtin University, Perth, Australia

**Keywords:** effector, fungi, pathogen, protein family, protein function

## Abstract

Plant diseases caused by fungal pathogens are typically initiated by molecular interactions between ‘effector’ molecules released by a pathogen and receptor molecules on or within the plant host cell. In many cases these effector-receptor interactions directly determine host resistance or susceptibility. The search for fungal effector proteins is a developing area in fungal-plant pathology, with more than 165 distinct confirmed fungal effector proteins in the public domain. For a small number of these, novel effectors can be rapidly discovered across multiple fungal species through the identification of known effector homologues. However, many have no detectable homology by standard sequence-based search methods. This study employs a novel comparison method (RemEff) that is capable of identifying protein families with greater sensitivity than traditional homology-inference methods, leveraging a growing pool of confirmed fungal effector data to enable the prediction of novel fungal effector candidates by protein family association. Resources relating to the RemEff method and data used in this study are available from https://figshare.com/projects/Effector_protein_remote_homology/87965.

## Data Summary

Supplementary Material: https://doi.org/10.6084/m9.figshare.13285733.v1. Additional data: https://figshare.com/projects/Effector_protein_remote_homology/87965

**Impact Statement**

Effector proteins of fungal-plant pathogens may be the key to understanding and developing new methods of controlling important agricultural crop diseases. Progress towards identification of new effector proteins has been slowed by challenges related to mutations in fungal genomes. It is typically ineffective to search for new effectors by looking for similar sequences to those of known effectors. This study describes a new, more sensitive method of searching for remote effector homologs (RemEff), broadly applying pattern-based searches and hierarchical networks of protein similarity relationships across multiple fungal species. This allowed prediction of many new effector protein candidates, which are relevant across multiple pathosystems. This study also highlights previously observed and newly predicted effector family groups among known and predicted effectors, which are predominantly unrelated by sequence. The RemEff dataset is publicly available and can benefit ongoing research across the molecular plant pathology community, through fast-tracking effector discovery efforts.

## Introduction

Fungal-plant pathogens expose or secrete molecules called ‘effectors’ into the extracellular environment, which may interact with or be internalized by their host, to promote infection. Hosts in turn may recognize pathogen-associated molecular patterns (PAMPs) and initiate defence responses, which confers innate immunity against the majority of pathogens, termed pattern triggered immunity (PTI) [1]. However, some pathogens employ a specialized infection strategy on a narrow range of hosts by secreting proteinaceous or secondary metabolite effectors, which either bypass host defences or cause host-cell death. Necrotic effector activity has been observed to rely on the presence of a cognate sensitivity (S) gene in the host genome, whereupon recognition of the NE by the S-protein will initiate host-cell death and promote necrotrophic infection [2]. A second layer of defence, termed effector-triggered immunity (ETI) may also be employed by the host, where the presence of a cognate resistance (R) gene will confer the ability to activate host defences in the presence of a recognized effector. Effectors are sometimes divided into subclasses based on their known interactions with host S and R genes, with necrotrophic effectors (NEs) interacting with S genes but having no known R genes, and avirulence effectors (AVRs) interacting with known R genes [2, 3]. Crop disease resistance breeding is usually conducted on the basis of introducing beneficial R genes and removing deleterious S genes. The study and discovery of fungal effectors among the growing pool of fungal genome data is vital for ongoing resistance breeding efforts [4], however there are a number of challenges that need to be overcome.

Proteinaceous fungal effectors have long been considered to lack sequence conservation, and in many cases have been presumed to have arisen independently. The collective term ‘effector’ is most frequently used to describe a highly diverse group of proteins with a common but broadly defined role in virulence on a narrow range of hosts, but is sometimes also used to describe highly conserved families of pathogenicity proteins with broad host specificity, such as the NEPs [5], cerato-platanins [6], and ribotoxins [7]. Little sequence similarity has been observed between known host-specific fungal effectors, potentially due to relatively high levels of genome plasticity in some fungi [8–13]. This is in direct contrast to effectors of a separate microbial lineage – the oomycetes – which are comparatively less plastic due to a lack of repeat-induced point mutation (RIP), fewer rearrangements to chromosome structure between genera [14, 15]. Unlike fungal effectors, oomycete effectors frequently have conserved motifs including RxLR-dEER [16] and LXLFLAK (Crinklers/CRN) [17]. Traditional biochemical and structural analyses are the gold standard for the functional characterization of effector candidates [18] but are unsuitable for high-throughput analyses. Moreover, existing high-throughput experimental methods, such as proteomics and genome-wide association studies, routinely return numerous genes or proteins that may be associated with the phenotype of interest, necessitating some additional information to prioritize future experimental validation.

High-throughput bioinformatic identification of fungal effector candidates remains a significant challenge due to the apparent lack of homology among most fungal effectors [19]. The vast majority of fungal proteins have no experimentally determined function and the accurate annotation of fungal genes is impeded by the narrow taxonomic range of fungal species with high-quality gene annotation and by the relatively high gene density observed in fungal genomes [20]. Nevertheless, a small but growing number of fungal effector families have been described with members in taxonomically distinct pathogens including: ToxA-like [21, 22] MAX [23], RALPH [24] and RXLR-like [25]. In line with elevated fungal genome plasticity, these effector ‘families’ share conserved structures but lack significant primary sequence similarity. This raises the possibility that at least some effectors – rather than arising independently or via lateral transfer – may have been vertically inherited from ancestor effector genes that were subsequently heavily mutated by fungal-specific genome mutagenesis mechanisms such as RIP [26]. Among the currently identified effector families, conserved structural folds with similar functions can be observed, which are typically missed by simple sequence alignments. Effector family relationships with high sequence divergence are difficult to predict with traditional methods (e.g. blastp), but more sophisticated structural prediction and comparison methods (e.g. protein threading and structural alignment) are not yet computationally feasible to include in a high-throughput analysis of a whole fungal proteome. Suitable alternatives come in the form of search methods that incorporate protein redundancy, such as profile-hidden Markov models (HMMs) or position site-specific models (PSSMs), which offer viable methods for finding remote homologues of confirmed effector proteins. Also of note are the cysteine-spacing classification systems that have been successfully applied to non-fungal cytotoxic venoms, which appear to have similar basic protein properties to fungal effectors [27, 28]. As our understanding of fungal effector biology improves, it may also become possible to apply similarly simple pattern-based heuristics for fungal effector classification.

### Fungal effector protein families

Fungal pathogenicity effector proteins can be divided into those which (1) form family groupings using simple bioinformatics methods, i.e. conserved motifs/patterns identified via sequence-based alignment, and (2) those which cannot be grouped by the above methods. In the case of the latter, there have been several studies to date piecing together a growing set of small cysteine-rich, secreted, low molecular weight, protein families with at least some members having effector-like phenotypes. There is remarkable diversity across these families, both between families and within them, yet common themes are emerging. Structural homology and in some cases similar modes of action [21] are observed between proteins with very low sequence identity, and some conserved or functional motifs appear to comprise surface-exposed, positively charged residues. A selected set of these emerging protein families are introduced below.

### ToxA

The ToxA-like family is named after the ToxA effector originally characterized in the wheat pathogen *Pyrenophora tritici-repentis* [29, 30], and for which putatively horizontally transferred loci were later identified by varying degrees of sequence similarity of the locus and a~14 Kbp flanking region [31–34], to genomes of other cereal-pathogenic fungi *Parastagonospora nodorum* [31], *Bipolaris maydis* [21] and *B. sorokiniana* [32, 35]. The full PtrToxA pre-pro-protein is 178 aa in length, with a signal peptide (SP) domain at position 22–23, and an N-terminal pro-peptide with a conserved ‘LXXR’ motif [21], which is cleaved during secretion at positions 60–61, producing the mature ToxA protein that corresponds to position 61 to 178 [29, 36, 37]. PtrToxA (and the identical PnToxA) interact with a NBS-LRR domain membrane protein Tsn1, which confers host sensitivity to ToxA [38]. Within wheat host cells, mature PtrToxA is reported to bind two chloroplast-localized proteins: ToxA binding protein 1 (ToxABP1, syn. *Triticum aestivum* thylakoid formation protein TaThf1), plastocyanin protein TaPCN [39] and TaPR-1–5 PR-1–5 [40]. ToxA-mediated disruption of chloroplast function leads to host cell necrosis, which requires light [41] and conservation of a structural loop possessing an ‘RGD’ motif [42].

The ToxA homologue of *B. maydis* (syn. *Cochliobolus heterostrophus*), ChToxA, has poor sequence similarity (64%) with Ptr/PnToxA, but has highly conserved structural similarity [21] and a similar light-dependent necrosis phenotype on maize. Despite the similar structure, the ‘RGD’ motif required by Ptr/PnToxA for necrosis of wheat is substituted with a ‘SGN’ motif [21]. Broadened similarity searches using hidden-Markov model (HMM)-based methods have predicted many other ToxA-like proteins across the classes Dothideomycetes and Sordariomycetes [21], including Avr2 of *Fusarium oxysporum*. Like ChToxA, Avr2 has a virulence-promoting phenotype, poor sequence identity with Ptr/PnToxA (~5 %), and high structural similarity [43]. There are however, a few motifs that are conserved across the currently predicted members of the ToxA-like effector family, including the ‘LXXR’ motif within the pro-domain, three motifs located in beta sheets 4, 5 and 8 (LXVXIXN, LILTXY, WXXQ respectively), and an asparagine-rich WXXN(S)NXIXVXI motif [21].

### MAX

The Magnaporthe Avrs and ToxB-like (MAX) effector family comprises another set of fungal proteins that are structurally conserved but divergent at the sequence level. The MAX family was originally derived from effectors of *Magnaporthe oryzae* [23]. Similarity of NMR structures containing two anti-parallel three-stranded beta sheets with a single disulfide bond has been demonstrated between *M. oryzae* AVR-Pia, AVR1-CO39, AvrPiz-t and *Pyrenophora tritici-repentis* ToxB [23, 44]. Sequence alignment, position-specific score matrix (PSSM) and profile-HMM searches against these structural homologues had subsequently revealed numerous homologues in other species, including *P. bromi* [45], *Bipolaris oryzae*, *Colletotrichum* spp., *Zymoseptoria tritici*, *Leptosphaeria maculans* and even low but significant similarity a protein in plant-associated bacteria *Pseudomonas* sp. *StFLB209* [23]. Multiple paralogues of members of this family have also been reported for some species, including *Pyrenophora* spp. [34], *C. fioriniae*, *C. orbiculare* and *C. gloeosporioides* [23], suggesting the potential for duplication and diversification of the relatively broadly-conserved MAX effector family.

### AvrLm6

AvrLm6 is a well characterized AVR effector of the brassica pathogen *Leptosphaeria maculans*, which causes necrosis but has an avirulent phenotype in *Brassica napus* and *B. juncea* hosts [46] possessing the resistance (R) locus *Rlm6* [47]. Several AvrLm6-like proteins have been reported in other fungal pathogen species, including: *Colletotrichum* spp., *Fusarium oxysporum*, *L. biglobosa* and *Venturia* spp. [48, 49]. Notably in *V. inaequalis* and *V. pirina*, this family has undergone extensive clonal expansion [49].

### Ribotoxins and RALPHs

Fungi secrete a broad variety of toxic and non-toxic RNases into the extracellular space and host [50]. One set of cytotoxic RNAses, the ribotoxins, are a group of fungal proteins that target the sarcin-ricin loop (SRL) of the host ribosome. This cleaves a single phosphodiester bond of the ribosomal RNA, rendering it catalytically inactive and ultimately causing cell death [7, 51]. Fungal secreted RNases tend to share a common *ɑ*-helix *β*-sheet fold topology, but differ in their terminal and loop domains [7, 50]. Ribotoxins possess an extended positively charged loop that is absent in non-cytotoxic secreted RNases, which is presumed to be important for interacting with the host-SRL [50, 51]. Ribotoxins are well documented in entomopathogens of the Ascomycetes (e.g. *Aspergillus giganteus* ɑ-sarcin and *Aspergillus restrictus* restrictocin [52] and are also found in Basidiomycetes (e.g. white-rot *Agrocybe aegerita* [53, 54]). Aspergillus ribotoxins are relatively well conserved [52], however members from other genera (e.g. *Hirsutella thompsonii* - Hirsutellin and *Agrocybe aegerita* - Ageritin) share low sequence identity with the Aspergillus varieties but retain similar structures and activities [54, 55]. Recently a cytotoxic secreted RNase protein, Zt6, was reported in the wheat pathogen *Zymoseptoria tritici* [56]. Although SRL binding has not yet been demonstrated for Zt6, it structurally resembles canonical ribotoxins and has RNase catalytic activity and exhibits toxicity to plants, some fungi and bacteria, but not to *Z. tritici* [56].

Another group of non-toxic RNases have been reported in the *Blumeria* genus of biotrophic plant pathogens. *Blumeria* possesses several large families of effector candidates, with one of the largest groups containing RNase-associated domains with predicted structural similarity to RNase proteins – the RNase‐Like Proteins associated with Haustoria (RALPHs) [57]. RALPH effectors include *AvrPm2* (*BgtE-5845*) in *B. graminis* f. sp. *tritici* [24, 58] and AVRa13 (aka CSEP0372), BEC1011 (aka CSEP0264) and BEC1054 (aka CSEP0064). Like many other mildew effectors the RALPHs possess a conserved Y(x)xC motif after the signal peptide [57] as well as a RALPH-specific RxFP motif, which may suggest roles in protein localization or virulence [58]. Like ribotoxins, some RALPHs appear to bind the ribosomal SRL but lack a catalytic site for mRNA cleavage. They have been proposed to have a protective function against host Ribosome Inactivating Ribonucleases (RIPs), which may be induced as part of a resistance response [59].

### Prior efforts in remote homology

Fungal effectors do not frequently exhibit detectable sequence similarity with other known sequences, thus finding novel effector candidates in the form of distant homologues is challenging, and may involve relaxing significance thresholds of blast *e*-values beyond recommended limits [23, 60]. However, a range of more sensitive sequence-based search techniques are available, which can exploit sequence features that may indicate conserved tertiary structures. Classification systems using cysteine spacing are well established for antimicrobial peptides and some venoms/toxins, where the number of and distance between cysteine residues indicates a possible shared topology of disulphide bonds [61–63]. However, conserved cysteine patterns are not guaranteed to indicate common structure or function [28], and known functional domains or discriminative motif analysis may also be necessary to separate active from non-functional forms [64, 65]. Although they have been a useful heuristic in other applications, cysteine spacing classification generally requires prior knowledge of a well-defined family, which would limit their application to effector family discovery.

Generally, *de novo* remote homology detection falls into two camps: iterative searches and alignments utilizing sequence information from similar proteins (profile search methods) [66], and machine-learning methods, which map the sequence into a multidimensional space (called an embedding, sequence space or feature space) and perform a classification or ranking task. The latter form may use relatively simple sequence features such as k-mers and sequence auto-correlation/covariance features [67], or may themselves use profile search results to construct a redundant representation of the sequence [68, 69]. Although these methods can achieve excellent results, they lack some of the interpretability of classical sequence search methods and are still not in general use. Sequence-based searches are much more commonly used, and the profile sequence-based methods like position-specific scoring matrices (PSSMs; e.g. PSI-blast [70]) or profile hidden Markov models (profile-HMMs; e.g. HMMER [71]) can find protein homologues with less than 30 % sequence identity. Even more divergent homologues can be found using profile HMM-HMM comparisons [72] or Markov random fields (MRF) [73]. These more sensitive methods can be relatively computationally intensive and some pipelines for remote homology detection will first run PSSM-based methods to reduce run-time [74, 75].

Many of these remote homology detection methods are designed to find homologues of a single protein and are not always applicable to the task of protein family identification. Although iterative PSSM or profile-HMM methods are capable of detecting similarity between very distantly related proteins, extensive post-processing of search results is necessary to remove spurious matches, identify families within search results, and recover potential matches missed by search heuristics or filtering thresholds. Identification of protein families typically involves an all-vs-all comparison between proteins and the construction of a graph (aka network) from significant alignments, from which families can be identified as subgraphs [76]. The best known and still most commonly used algorithm for finding subgraphs corresponding to protein families is by Markov clustering (MCL) originally used in TRIBE-MCL [77]. More recent heuristic algorithms that do not require all-vs-all comparisons have been investigated [78], but are yet to gain widespread use or a stable toolset.

In this study we apply a combination of sensitive sequence comparison methods and protein clustering methods to investigate the possibility of extending fungal effector protein families from the currently known set of fungal effectors. We use an agglomerative clustering approach with iteratively increasing sensitivity to find clusters of protein groups that show differing levels of similarity, which we have termed RemEff. These groups highlight previously unreported relationships between several known effectors, the presence of large effector families, and will support future studies of fungal effector function and evolution. RemEff and the resulting data from this study will also serve as an important resource in the field of molecular plant pathology for effector candidate prediction and study, with relevance to multiple fungal-plant pathogen species.

## METHODS

### Data sets

Non-redundant fungal protein datasets (Table S1) were downloaded from the UniParc database (https://www.uniprot.org/uniparc/, filter: ‘taxonomy:‘Fungi (9FUNG) [4751]’, downloaded 24 January 2020) and the NCBI Identical Protein Groups database (https://www.ncbi.nlm.nih.gov/ipg/, filter: ‘Fungi’[Organism] OR fungi[All Fields]’, downloaded 28 January 2020) totalling 10 946 400 and 11 351 342 proteins, respectively. Data was supplemented using published genomes from JGI MycoCosm (https://genome.jgi.doe.gov/mycocosm/home), an Endophyte genome database (http://csbio-l.csr.uky.edu/endophyte/) [79–83], the *Alternaria* genome database (https://mycocosm.jgi.doe.gov/Alternaria/Alternaria.info.html) [84], and the ‘Gemo’ database (http://genome.jouy.inra.fr/gemo/) [85].

Additional genomes, proteomes and effector sequences collected from selected papers were included if they were not represented in the databases (Tables S2 and S3).

Datasets were combined to give a single non-redundant dataset using ‘seguid’ checksums [86] implemented in BioPython [87]. Proteins were filtered by length, including only proteins longer than 30AA and shorter than 6000AA. Unique sequences corresponding to published effectors and PHI-base entries were identified by searching the initial dataset using MMSeqs2 (version 10-6d92c) [88], requiring a minimum sequence identity of 90 % and at least 90 % reciprocal coverage, selecting the match with the highest bit-score.

### Clustering

The non-redundant fungal protein set was clustered in multiple stages using ‘MMSeqs2’ (version 10-6d92c) [88]. Protein sequences were initially clustered using the ‘cascade’ clustering pipeline in three steps to a minimum of 30 % sequence identity and 80 % coverage of all members. To group more distant sequences, a second stage of clustering was performed using sequence profiles. Clusters were converted to sequence profiles and the profiles were enriched using the original input dataset of fungal proteins (including those sequences ≤ 30 aa or ≥ 6000 aa) to include information from sequences that did not pass the coverage threshold. The enriched profiles were searched against consensus sequences from the cluster profiles and were clustered to have a minimum of 10 % identical AAs and 70 % reciprocal coverage. In further analyses in this study, these resulting clusters are referred to as ‘cluster level 1’.

MSAs for each cluster’s sequences were constructed using decipher version 2.10 [89] using the pfasum15 substitution matrix [90], two iterations, two refinement iterations and alignment adjustments with staggering. A consensus sequence was added to the MSAs using decipher, where columns with more than 50 % gaps were considered gaps in the consensus. Code used for clustering sequences and constructing MSAs is available at https://githubcom/darcyabjones/pclust.

### Remote homology comparison

To find ‘low-level’ sequence similarities between level 1 clusters, profile HMM-HMM searches were performed (Supplementary Data Sheet 1). MSAs with consensus sequences were first converted to MMSeqs2 profiles (--match-mode 1 --match-ratio 1) and enriched by searching against a database consisting of all fungal sequences of UniRef-90 (downloaded 2020-01-30. Query: ‘taxonomy:‘Fungi [4751]’ AND identity:0.9’) and the entire UniRef-50 database (downloaded 30 January 2020), selecting matches with a maximum *e*-value of 10^−5^. Cluster MSAs and MSAs constructed from the profile matches were combined and converted into an HH-Suite database (version 3.2.0) [72]. ‘Match’ states in the HMMs and A3M alignments were determined by the consensus sequences of the cluster MSAs prior to enrichment (--match-ratio=first), where gaps in the consensus represent an insertion in the model.

To reduce computational requirements and to focus on fungal effectors, a subset of clusters were found by searching selected sequences of known effectors and virulence factors from numerous pathogens included in PHI-base version 4.8 [91], and a custom database of known effector sequences and homologues (Table S3). PHI-base entries to use for subsetting were selected based on annotated phenotypes, functional descriptions and secretion prediction by SignalP versions 3, 4.1 g, and 5.0b [92–94], DeepSig version 1 [95], Phobius version 1.01 [96], and tmhmm version 2.0 c [97]. The sequences were first enriched into MSAs using the cluster HMMs, using two HHBlits search iterations. The enriched sequence MSAs were then searched against the cluster HMMs allowing a maximum *e*-value of 0.01, minimum probability of 0.20 and realigning up to 20 000 matches (-n 1 -e 0.01 p 20 -Z 20000 -z 0 -B 20000 -b 0). Code used for constructing HMMs, subsetting the database, and performing all-vs-all comparison is available at https://github.com/darcyabjones/pclust.

To identify remotely homologous clusters (referred to here as level 2 clusters or superclusters), the subset of HMMs matching selected PHI-base or effector sequences were searched against themselves (all-vs-all) using HHblits (-n 1 -e 0.01 -E 0.01 -z 0 -Z 20000 -b 0 -B 20000 -pre_evalue_thresh 10 min_prefilter_hits 10 -realign_max 20000). Pairwise matches were considered significant if they had an *e*-value ≤10^−5^, probability ≥0.9, alignment length ≥>=30 AAs, and where the alignment covered at least 70 % of at least one HMM in the pair. Where there were multiple alignments between the same pair of proteins, the alignment with the highest score was selected. Alignments were then filtered so that only reciprocally significant matches were retained. To reduce any score bias in alignments caused by HMM lengths, we adopt the normalization approach used by OrthoFinder [98] with modifications. Briefly, HMM search self matches were selected from the alignments, the HMM length was squared, the selected alignments were sorted by the squared HMM length, and the top 5 % of alignments (by score) were selected from non-overlapping 1000 element sized bins in the sorted list. The log_2_ transformed alignment scores were regressed on the log_2_ transformed squared HMM lengths, and the slope and intercept were taken to transform scores using the same formula described in Emms and Kelly [98]. Conceptually, this transformation normalizes the scores by the average maximum possible score for an alignment of two proteins with those lengths. Alignments were then further filtered to require alignments between both HMMs to be covered at least 70 % of their respective lengths, and reciprocal matches were selected again. Each pair of alignments were grouped and the arithmetic mean of the two normalized scores was used as a single score for each pair, and the scores were converted to a value between 0 and 1 by dividing by the highest score in the full set of pairwise matches.

The filtered, score-normalized alignments were used to construct a weighted, undirected graph (AKA network) using the Python libraries, networkx [99], Pandas [100] and SciPy [101]. Clusters (superclusters) in the graph were found using a reimplementation of the greedy set cover algorithm [102] and with Markov clustering [77] (https://github.com/GuyAllard/markov_clustering), which due to their relatively similar stringencies were designated ‘cluster level 2A’ and ‘cluster level 2B’, respectively. Connected components were also found to summarize higher-order relationships, which were also designated as ‘cluster level 3’. The MCL inflation parameter was selected by running MCL on ten randomly selected connected components containing 300–600 nodes, for a range of inflation parameters between 1.1 and 2.0. The inflation parameter that gave consistently higher modularity scores [103] was selected for overall clustering. Graphs and subgraphs were visualized using the Graph Tool Python library [104].

### Supercluster comparison

To interrogate sequence conservation within and between cluster levels 2 and 3, composite multiple sequence alignments were constructed and visualised as sequence logos [105].

Enriched multiple sequence alignments from level 1 clusters (used to form HMMs) were combined using a progressive algorithm, guided by the maximum spanning tree of the subgraph containing the clusters of interest, where MSAs were pairwise aligned using hhalign [72]. For each pairwise alignment, two alignments were computed using each MSA as the template. A pairwise alignment was considered to have succeeded if the resulting MSA contained sequences from both input MSAs, and if both pairwise MSAs succeeded the result containing more sequences was selected for further iterations. If an alignment failed to merge two MSAs, the alignment was scheduled to be retried after all other alignments had been completed, stopping if no more MSAs could be merged. Un-connected components from this progressive method were then pairwise aligned in random order, shuffling the list if no MSAs had been merged in a full pass through the list, and stopping the process if no MSAs had been merged in ten passes through the list, resulting in one or more MSAs. Each combined MSA was converted into an HMMER profile HMM [71], and used as a template profile to align all sequences from the level 1 clusters (not including the enrichment sequences) using Clustal omega [106].

Sequence logos were computed by filtering out sequences from the final MSA with more than 90 % pairwise identity using hhfiltr; [72 ], and the resulting MSAs were plotted using Logomaker [107] and matplotlib [108] using the information content as the logo heights.

### Comparison to iterative search results JackHMMER

We compared the output clusters of the RemEff pipeline to an output from a standard progressive HMM search using JackHMMER [71] for benchmarking purposes. The same sequences used to initial clusters of interest before remote homology inference (PHI-base version 4.8, and effector sequences and homologues in Table S3) were searched against the entire non-redundant fungal protein dataset using JackHMMER with a maximum of five iterations and a maximum *e*-value of 1 (-N 5 -E 1 –domE 1). The resulting matches were then filtered to have a full sequence *e*-value of ≤ 0.01, and summarized to show the number of significant matches, and matches to known effectors or published effector homologues.

## Results

### Protein dataset and initial sequence clustering

**Fig. 1.**
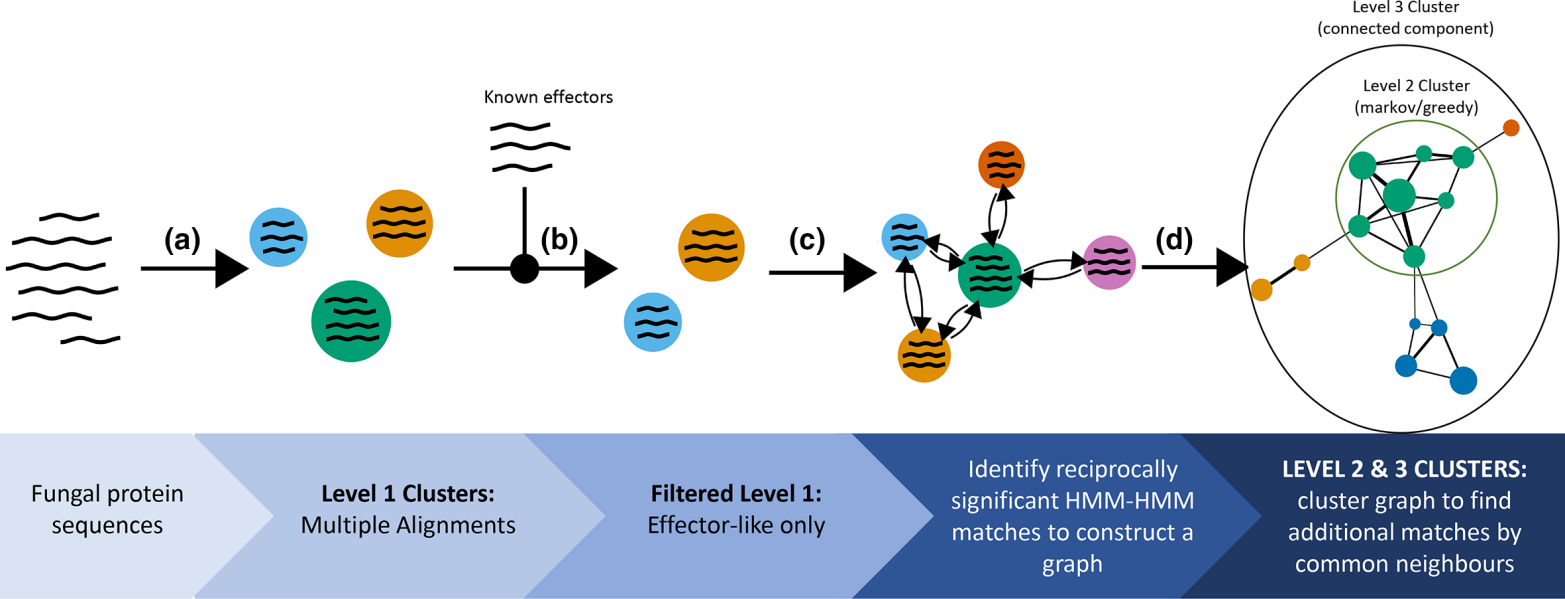
The clustering workflow employed in this study. (a) Sequences are initially clustered using MMSeqs2 resulting in 3 111 468 level 1 clusters. (b) A subset of 286 512 of these clusters with any similarity to known effectors are found using HHBlits. (c) All clusters from this subset are searched against each other and reciprocally significant alignments are selected to form a graph. (d) Clusters of the initial clusters are found within the resulting graph to form more distant sequence families. In the final graph, each point represents a level 1 cluster resulting from step A, the colours indicate level 2 clusters (Markov or greedy clustering), and the whole graph forms a single connected component (level 3 cluster).

**Fig. 2.**
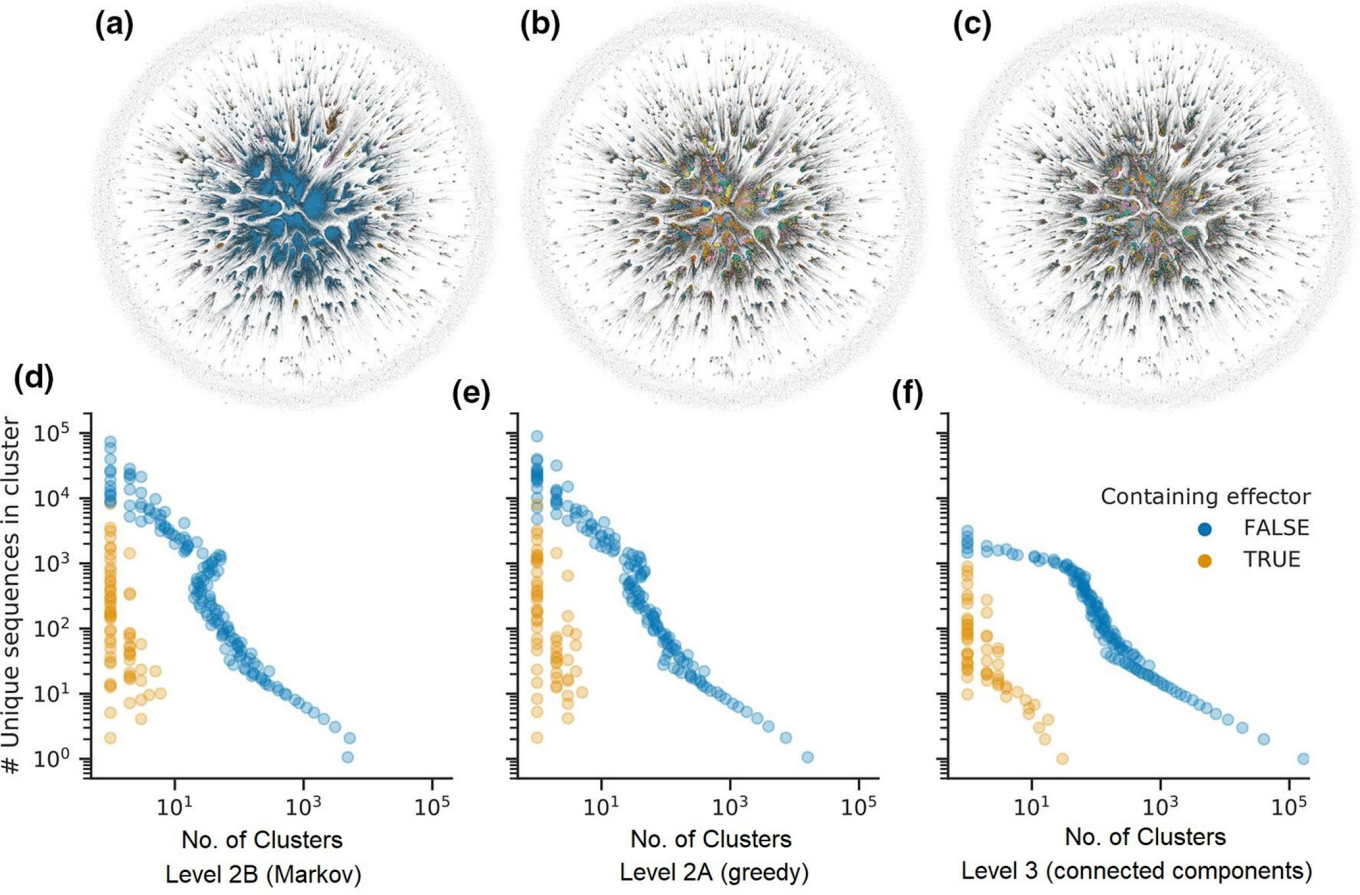
Top row: plot of graph coloured by connected components (a), and Markov (b) and greedy (c) clusters. Bottom row: the number of unique sequences compared with the number of clusters with that size, within Markov (d), greedy (e) and profile clusters (f). For the bottom row, Y-axis values are in binned into 100 evenly sized ranges taken from a 10-based exponential space (10^0^¨^max(#seqs)^).

Nearly fourteen and a half million unique sequences spanning 69 724 distinct NCBI taxonomic ids were collected from public databases for clustering and remote homology comparison (Table 1). A first pass clustering of these proteins with MMSeqs2 [88] yielded 3 111 468 clusters, which were designated as ‘level 1’ clusters (Fig. 1). Within this first pass, position-specific scoring matrix (PSSM) profile clustering did not merge any clusters from the standard MMSeqs2 ‘cascaded clustering’ pipeline, but was observed to merge clusters in datasets with fewer sequences and when the coverage criteria were relaxed from 80 % reciprocal as required here. The majority (1 918 741) of level 1 clusters consisted of one or two (the median) unique sequences (Fig. 2f). A smaller number of large clusters were observed, with the largest 10 % of clusters containing eight or more unique sequences and a maximum cluster size of 10 244. From these level 1 clusters, enriched profile-hidden markov models (HMMs) were constructed for use with HH-Suite version 3.2.0 [72]. To focus on finding potentially grouped families of effectors, HMMs for remote homology comparison were selected based on HHBlits matches to 6598 selected PHI-base [91] and effector sequences (Table S3). Of these sequences, 1078 sequences matched 286 512 level 1 cluster HMMs with a maximum *e*-value of 0.01 and minimum probability of 20%, which were selected for HMM-HMM comparisons. Of the subset of clusters selected for remote homology comparison, 2856 clusters contained unique sequences corresponding to 310 sequences from the effector and effector homologue dataset, and 3571 PHI-base entries.

**Table 1.** Summary of the number of unique sequences in the input dataset (A) and the number of clusters obtained using various methods for remote homology clustering (B)

(A) Initial clustering of input data for removal of sequence redundancies				
	Total	Uniparc	NCBI IPG	Custom
Unique Sequences	14 425 844	11 987 341	12 293 758	3 130 080
Unique sequences per taxid	23 351 787	19 081 482	14 297 803	3 302 707
**(B) Remote homology clustering**				
**Level**	**Total dataset**		**Containing known effectors**	
1 (profile)	286 512		200	
2A (greedy)	45 363		103	
2B (Markov)	27 851		104	
3 (connected components)	6538		80	

### Clustering of profile HMM-HMM matches to identify remote homology relationships between effector-like sequence clusters

To identify more distant relationships, all-vs-all profile HMM-HMM comparisons were performed on the 286 512 selected (effector-like) level 1 clusters (Fig. 1). A total of 224 230 level 1 clusters were connected by 30 472 762 reciprocally significant alignments (*e*-value <=10^−5^, probability >90 %, reciprocal coverage >70 %). The remaining clusters had no matches at this significance threshold, and so could not be grouped into more remote clusters. Of these clusters without significant matches, 71 clusters contained unique sequences corresponding to 104 effectors and effector homologues. A strong correlation between alignment scores and sequence lengths was observed, which was effectively removed by normalization (Fig. S1). A graph was constructed of the level 1 effector-like clusters and their connecting alignments, using the mean of the pair of normalized scores as edge weights. The graph consisted of 6538 connected components (sub-graphs of directly or indirectly connected level 1 clusters), which were designated ‘level 3’ clusters. A single large component containing 171 346 nodes/level 1 clusters (representing 1.9 million unique proteins) was observed, with numerous small components typically with fewer than 1000 nodes also present (Table 1b, Fig. 2a). Despite the presence of one large connected component, most level 1 clusters, which corresponded to known effectors were found in smaller components, with only 19 out of 310 known effectors and effector homologues found in the largest component. These were typically highly conserved protein families including LysM-domain containing proteins, CRN, Tom1, Ave1 (expansins/PNPs), MoMSP (a cerato-platanin), though the NEPs and ribotoxins each formed a separate component. In order to sub-divide larger connected components into more stringent remote-homology groupings, ‘communities’ or ‘superclusters’ of level 1 clusters were found within connected components using the greedy set cover [102] and Markov clustering [77] algorithms. A Markov clustering inflation value of 1.35 was selected for clustering, which gave the highest average modularity scores [103] for a random selection of smaller connected components. Greedy and Markov clustering (referred to as ‘level 2A’ and ‘level 2B’ clusters, respectively) generally yielded comparable groupings, but greedy clustering tended to produce more clusters with only one member (Fig. 2d, e, Tables S4 and S5). Although each clustering method is different, conceptually the cluster levels from 1 to 3 represent progressively more distant homology relationships.

### Level 2 and 3 clusters grouped multiple known effectors and predicted an expanded set of effector candidates across multiple pathogen species

**Fig. 3.**
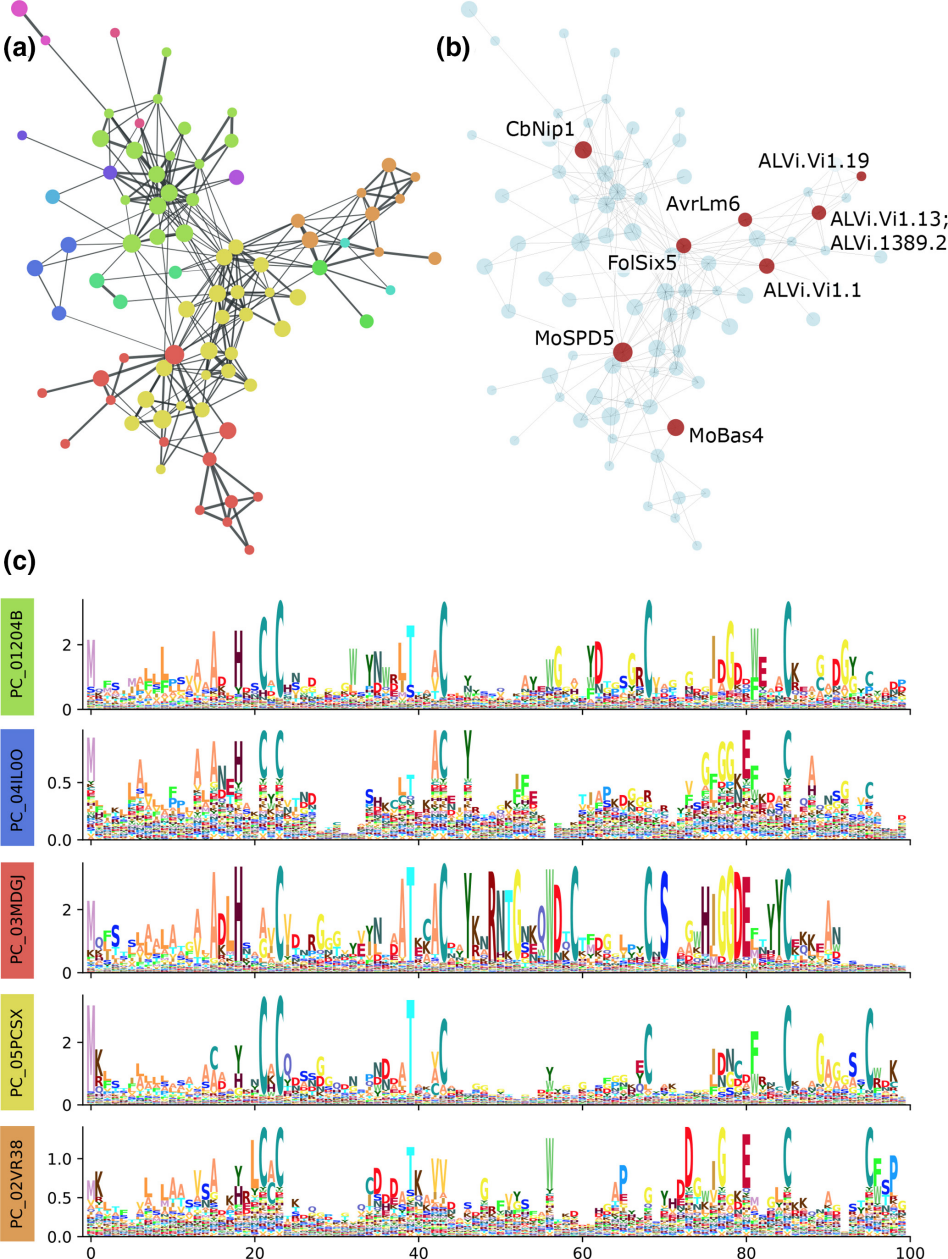
A family of SIX5-like effector sequences. (a) The connected component containing the effectors AvrLm6, Bas4, SPD5, CbNIP1 and SIX5, coloured by Markov cluster membership (level 2B). Nodes in the graph each represent a single HMM (level 1 cluster) with node size indicating the relative number of unique sequences contained in the HMM, and edges represent where a significant sequence alignment was found between the two HMMs. (b) The same graph, but highlighting the level 1 clusters containing effector sequences and published effector homologues (ALVI*). (c) Sequence logos resulting from multiple sequence alignment of all sequences in the connected component (level 3 clusters). Logos for Markov clusters with more than 10 members are shown separately. Columns in the multiple sequence alignment with more than 50 % gaps are excluded.

We found 80 clusters at level 3, 103 and 104 clusters at level 2A and 2B, respectively, and 200 clusters at level 1, which contained known effectors and published effector homologues (Table 1). Of these, 20, 24 and 26, and 10, respectively, contained two or more known effectors (Table S4, Fig. 2d, e). To demonstrate how known effectors have been grouped into novel ‘families’ in this study, we present three examples in detail. The first examples consist of a level 3 cluster (connected component) that contains *Leptosphaeria maculans* AvrLm6 [47], *Magnaporthe oryzae* BAS4 and SPD5 [109, 110], *Fusarium oxysporum* f. sp. *lycopersici* SIX5 [111], and *Cercospora beticola* NIP1 [112] (Fig. 3a, b). At cluster level 2, this group is further divided into sub-groups, with AvrLm6 and some published *Venturia inaequalis* AvrLm6 homologues [49] forming a distinct sub-group, *M. oryzae* SPD5 and BAS4 forming another sub-group, and *C. beticola* NIP1 and *F. oxysporum* SIX5 both forming their own distinct sub-groups, with additional sub-groups that did not match a known effector. Sequence logos generated from a multiple sequence alignment of all level 1 clusters contained in these subgroups (Figs 3 and S2, Supplementary Data Sheets 2–5) indicated conservation of specific cysteine, threonine, and glycine residues, as well as distinct motifs that were specific to each sub-group. Level 2A clusters PC_02VR38 (containing AvrLm6), PC_01204B (containing CbNip1), and PC_03MDGJ (containing SPD5 and BAS4) are found in numerous species from the Leotiomyceta clade including *Bipolaris* spp., *Colletotrichum* spp., *Leptosphaeria* spp., *Venturia* spp., and *Fusarium* spp. (Tables S4 and S5). The cluster containing *Fol* SIX5 (PC_05PCSX) was found in a broader range of taxa including the basidiomycetes *Jaapia argillacea* and *Plicaturopsis crispa*, but most observed sequences were from species in the Pezizomycotina, including other significant plant pathogens such as *Zymoseptoria tritici* and *Pyrenophora teres* f. sp. *teres*.

**Fig. 4.**
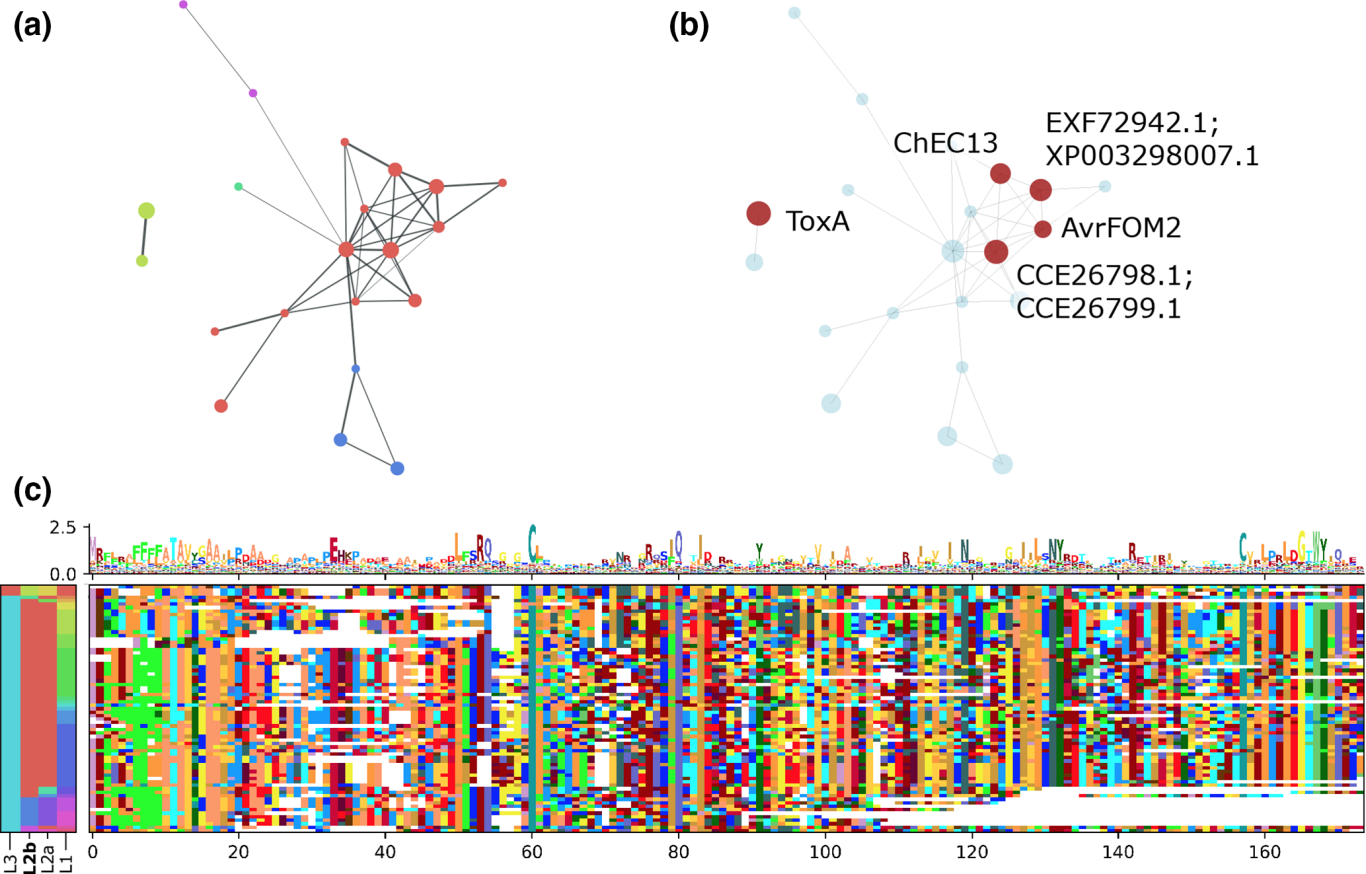
ToxA-like fungal effector groups. (a) The connected components (level 3 clusters) containing ToxA-like and AvrFOM2-like sequences, coloured by Markov cluster membership (level 2B). Nodes in the graph each represent a single HMM (level 1 cluster) with node size indicating the relative number of unique sequences contained in the HMM, and edges represent where a significant sequence alignment was found between the two HMMs. (b) The same graph shown in (a), but highlighting level 1 clusters containing known effectors and published effector homologues. (c) A multiple sequence alignment constructed from all sequences in the ToxA-like and AvrFOM2-like connected components. Columns in the multiple sequence alignment with more than 50 % gaps are excluded. Colours on the y-axis indicate the level 1, 2 and 3 clusters that members belong to, with level 2B (Markov) cluster colours matching those in (a).

The second example consists of two separate connected components (level 3) clusters that correspond to the conserved ToxA effectors of *Pyrenophora tritici-repentis*, *Parastagonospora nodorum* and *Bipolaris* spp. [21, 35], and a set of loosely conserved ‘ToxA-like’ proteins, which had been previously identified in other studies using PSI-blast searches, including ChEC13 [21] and AvrFOM2 [22] (Fig. 4a, b). Our method did not link these two reportedly related groups within a single level 3 cluster. Alignments between the two connected components were observed, but failed the *e*-value significance threshold (data not shown). Multiple sequence alignment combining all sequences from both level 3 clusters showed only low-level similarity between sequences of these two level 3 clusters (Figs 4 and S3, Supplementary Data Sheet 6). Between the ToxA and ToxA-like/AvrFOM2 clusters, there are several broadly conserved residues, most notably two cysteines, two aromatic [W|Y] residues, several aliphatic [L,I] residues, and an LxxRQ…C motif. The AvrFOM2 cluster(s) are more diverse than ToxA, with conserved residues separated by hypervariable regions, and also possess a phenylalanine rich region in the signal peptide. The AvrFOM2 cluster also lacks a recognizable ‘RGD’ [42] or ‘SGN’ [21] motif (positions 138–140 in Fig. 4c), which is absent in the level 2B (Markov) cluster PC_08EP4N and replaced in this alignment by a poorly conserved ‘TTP’ consensus in level 2B cluster PC_07OLPP. The component containing ToxA sequences (PC_03B2DN) was observed to have members in several species that have been previously described: *Pyrenophora teres formae speciales*, *Pyrenophora tritici-repentis*, *Parastagonospora nodorum*, *Parastagonospora avenae*, *Bipolaris maydis* and *Bipolaris sorokiniana* (Tables S4 and S5). The level 2B cluster containing AvrFOM2 and ChEC13 was observed in numerous species within the leotiomyceta clade, including *Epichloe* spp., *Fusarium* spp., *Pyrenophora* spp., *Colletotrichum* spp. and *Bipolaris* spp. Other level 2 clusters within the component containing AvrFOM2 and ChEC13 also contain members from the leotiomyceta, but are specific to genus (*Epichloe*), or a strain (*Zymoseptoria ardabiliae* STIR04_1.1.1, *Balansia obtecta* B249). In a similar manner to the ToxA and ToxA-like clusters, members of the MAX effector family and the homologues published by de Guillen *et al*. [23] were found in 12 separate connected components in this study. None of these components contained more than one of the experimentally validated MAX members (ToxB, Avr1_CO39, AVR_Pik, AvrPiz_t and AvrPib). (Fig. S4, Supplementary Data Sheet 7) (4).

**Fig. 5.**
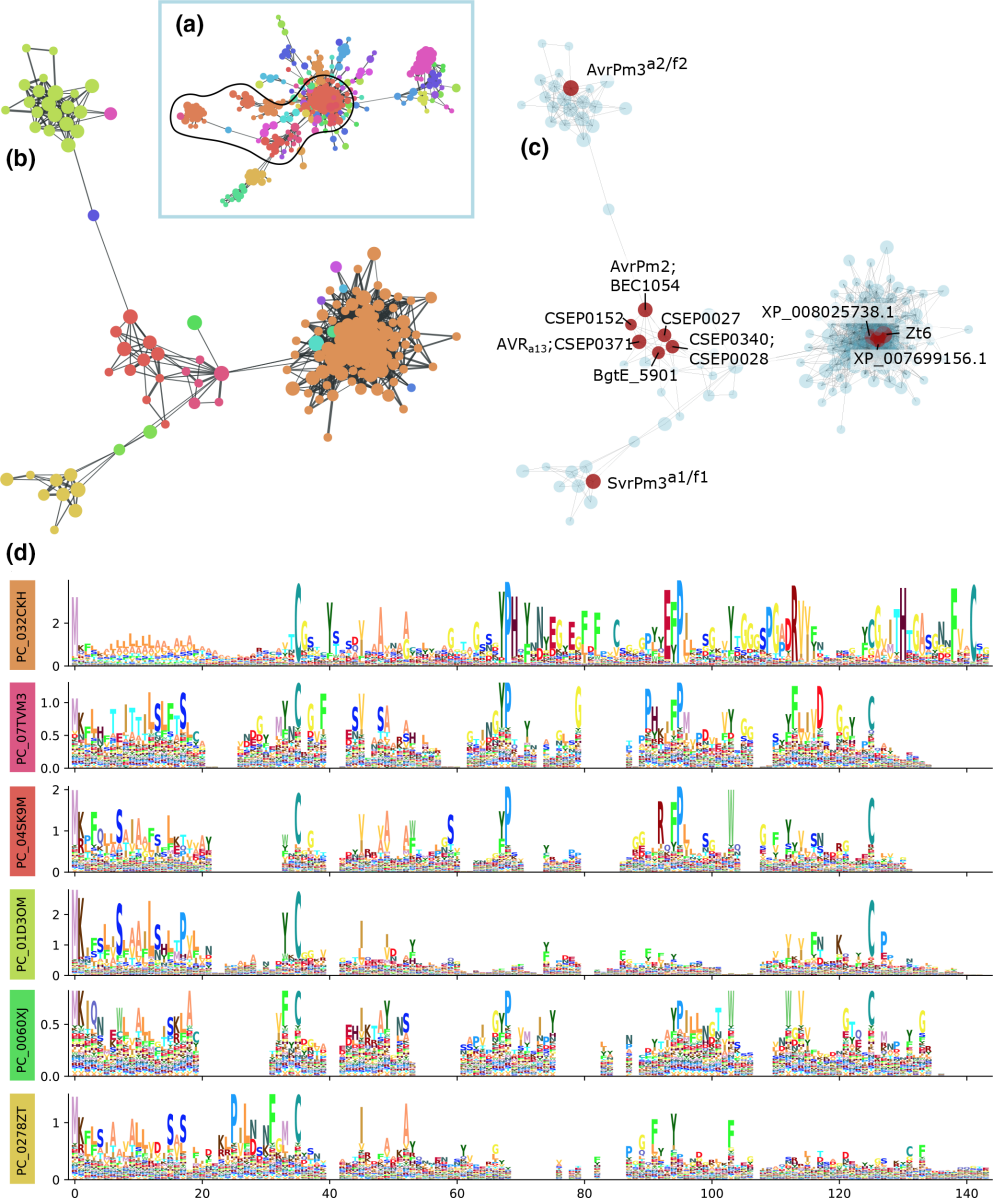
A connected component containing RNase-like effectors. A single connected component containing the ribotoxins and RALPH effectors was observed (a). Nodes in the graph each represent a single HMM (level 1 cluster) with node size indicating the relative number of unique sequences contained in the HMM, and edges represent where a significant sequence alignment was found between the two HMMs. (b) Shows a subset of the connected component containing all level 2 clusters containing effector sequences (c). (d) Sequence logos for selected level 2B (Markov) cluster from a multiple sequence alignment of all sequences in (b). Colours in the left boxes corresponding to colours in (b). Logos with fewer than 10 members are not shown. Columns in the MSA with greater than 50 % gaps are excluded from the visualisation.

The third example is a level 3 cluster of RNase-like effectors that grouped level 1 clusters, which were sufficiently divergent that profile alignments between all sequences in the MSA was not possible. Consequently, further presentation of this example focuses on a sub-graph, which includes level 2A and 2B clusters containing known RALPH [24, 58, 59] and ribotoxin [56] effectors (Fig. 5a). The ribotoxins (including Zt6) formed a large and densely connected cluster, which was distinct from all RALPH effectors (Fig. 5b, c). The RALPH effectors consist of three main groups: AvrPm3^a2/f2^, AvrPm2/BEC1054/AVR_a13_ and SvrPm3^a1/f1^, and are sparsely connected. Multiple sequence alignment of all sequences in the selected clusters indicate two or four conserved cysteine positions in the RALPH and ribotoxin logos, respectively (Figs 5 and S5, Supplementary Data Sheet 8). Additional conserved proline, aromatic [Y|F], and aliphatic [V|I] residues were observed. The clusters containing AvrPm2-like RALPH proteins (level 2B cluster PC_04SK9M) were more similar to the ribotoxin/Zt6-like cluster (level 2B cluster PC_032CKH), than the clusters containing AvrPm3^a2/f2^/SvrPm3^a1/f1^ sequences (level 2B clusters PC_01D3OM and PC_0278ZT, respectively). The Y(x)xC motif commonly found in after the signal peptide in *Blumeria* effectors [57, 58] appeared to be enriched in AvrPm2-like and AvrPm3^a2/f2^-like RALPH sequences, but may be replaced by an F(x)xC motif in SvrPm3^a1/f1^-like sequences. The level 2B cluster containing the known ribotoxin effector Zt6 (PC_032CKH) was broadly conserved in the Fungal kingdom (Tables S4 and S5). Sequences belonging to level 2B clusters corresponding to RALPH effectors (AvrPm2/PC_04SK9M, SvrPm3^a1/f1^/PC_0278ZT, and AvrPm3^a2/f2^/PC_01D3OM) were only found in *Blumeria graminis formae speciales*. However, several other lineage specific level 2 clusters were observed within the same connected component, which were most often associated with the Pezizomycotina.

Ten other level 2 clusters that grouped two or more known effectors were identified (Table S4), grouping: *Leptosphaeria maculans* AvrLm2 and *Fusarium oxysporum f.* sp*. lycopersici* SIX1 [113, 114]; *Zymoseptoria tritici* NIP1 and *Passalora fulva* Ecp2 [115, 116]; *Blumeria graminis f.* sp*. hordei* BEC2 and *Golovinomyces orontii* GoEC2 [117]; *Passalora fulva* Ecp6 and *Zymoseptoria tritici* Mg3LysM [118, 119]; NIS1 effectors [120, 121]; *Zymoseptoria tritici* MgxLysM and Mg1LysM [119]; *Magnaporthe oryzae* AVR-Pita and AVR-Pita2 [122, 123]; *Puccinia striiformis* Shr4 and Shr6 [124]; Pit2 effectors [125, 126]; and the NEP virulence factors [127–129, 130].

In other cases, clusters containing a single known effector were assigned functional annotations of high relevance to potential effector functions. For example, a large cluster (level 3: PC_07OBLJ, level 2: PC_058FSP, Table S4) corresponding to known effector BAS3 (biotrophy-associated secreted protein 3) of *Magnaporthe oryzae* [109], was functionally annotated as similar to scorpion knottin toxins [InterPro: IPR036574]. This group contained unconfirmed candidates from other *Pyricularia* spp., as well as *Colletotrichum* spp., *Macrophomina phaseolina*, *Neofusicoccum parvum* and *Monosporascus* spp.

Some known effectors were not able to be grouped beyond cluster level 1 (Table S4). Of these, 21 were within clusters that contained a single unique sequence, including AvrLm11 and AvrLmJ1 of *Leptosphaeria maculans* [131, 132], Avr5 of *Passalora fulva* [133], AVR_a10_ of *Blumeria graminis f.* sp*. tritici* [134], PIIN_08944 and FGB1 of *Piriformospora indica* [135, 136], CDIP3 and Slp1 of *Magnaporthe oryzae* [137, 138], lsc1 of *Verticillium dahliae* [139], Zt80707 of *Zymoseptoria tritici* [140], and the putative effector CSEP-07 of *Phakopsora pachyrhizi* [141]. Another 50 level 1 clusters containing a single effector possessed two or more unique sequences, for which most were restricted to isolates of the same species or genus. These included Tox1 of *Parastagonospora* [142]; SIX2, SIX4, and SIX8 of *Fusarium oxysporum* [111, 143]; DN3 and EP1 of *Colletotrichum* [144, 145]; AvrP4 and AvrL567 of *Melampsora* [146, 147]; SSVP1 of Sclerotiniaceae [148]; Shr5 and Shr7 of *Puccinia* [124]; NIP3 of *Rhynchosporium* [149]; and SCP7 of *Verticillium* [150]. Notable exceptions of level 1 clusters which spanned genera were Ecp1 of *Passalora fulva* [116] which had a homologue in *Pseudocercospora eumusae*, AVR4 of *P. fulva* [151] which had a homologue in *Dothistroma septosporum*, AvrLm3 of *L. maculans* [152] which had homologues in *P. fulva* and *Fusarium oxysporum f.* sp*. narcissi*, and SSVP1 of *Sclerotinia sclerotiorum* [148] which had homologs in *Botrytis* spp. and *Monilinia laxa*.

### Benchmarking RemEff clusters against progressive profile-HMMs (JackHMMER) cluster

To determine whether the observed relationships reported by RemEff are comparable to outputs from progressive profile-HMM methods that have previously been used to identify several effector families [21–23, 48, 49], the 591 effector and effector-like sequences (Table S3) were searched against the complete non-redundant protein dataset using JackHMMER [71] (five search iterations, full length *e*-value ≤ 0.01). Of the 591 query sequences, 433 had significant matches to 942 978 distinct proteins in this dataset. Of those, 640 727 were present in the filtered clustering dataset used for HMM-HMM comparison in the RemEff pipeline, corresponding to 97 887 level 1, 15 986 level 2A (greedy), 10 935 level 2B (Markov), and 2566 level 3 (connected component) clusters.

JackHMMER recovered many of the putative relationships between effectors identified by RemEff as well as some additional matches, but also missed relationships inferred by graph clustering (Table S6). In the SIX5-like group presented in Fig. 3, each of SIX5, CbNIP1, BAS4 and SPD5 were reciprocally significant. However, using AvrLm6 as the query only identified SIX5 and SPD5 as candidate homologues, and only the match with SIX5 was reciprocally significant. As previously described by [22] (with corresponding RemEff clusters presented in Fig. 4), Pn/Ptr-ToxA, ChToxA, ChEC13 and AvrFOM2 were all identified as being reciprocally significant matches using JackHMMER. The RALPH effectors AvrPm2, AvrA13 and BEC1054, were all reciprocally significant, and all matched the ribotoxin Zt6, but only the match to BEC1054 was reciprocally significant. Neither AvrPm3a2/f2 nor SvrPm3a2/f2 were identified as potential homologues of other RALPH members using JackHMMER. SvrPm3a2/f2 matched Zt6 but was not reciprocally significant. Similar to RemEff, none of the experimentally validated MAX effectors (ToxB, Avr1_CO39, AVR_Pik, AvrPiz_t, and AvrPib) were identified as matches to each other using JackHMMER.

Some additional potential effector similarities not reported by RemEff were observed in the JackHMMER results. Searching using AvrLm3 as the query identified AvrLmJ1 as a potential match, but this was not reciprocally significant. Local alignment of the AvrLm3 and AvrLmJ1 amino acid sequences using the EMBOSS water aligner with a BLOSUM45 substitution matrix [153] did show some similarity between the two sequences and potentially a shared cysteine spacing pattern. SIX6, SIX1 and SIX13 were identified as possible matches to SIX4 using JackHMMER, and SIX13 also matched SIX6. However, none of these alignments were reciprocally significant. Multiple sequence alignment of SIX1, SIX6, SIX13, SIX4 and AvrLm2 (which is a reciprocally significant match to SIX1, also identified using the clustering method) amino acid sequences using mafft [154] showed only a small central region of similarity containing four conserved cysteine positions. Using CfECP2 as a query JackHMMER identified PgShr8 as a potential candidate match, but this match was not reciprocally significant. Multiple sequence alignment of CfECP2, ZtNIP1 (which was a reciprocally significant match to CfECP2), and PgShr8 showed some conserved glycine, threonine, cysteine and valine residues between the three sequences in the central and C-terminal regions. However, the alignment indicated the presence of an additional N-terminal region in Shr8, and Shr8 was clearly distinct from ZtNIP1 and CfEcp2 (Supplementary Data Sheet 9), Finally, BEC2, GoEC2, BEC4 and MoCDIP2 were all identified as reciprocally significant matches using JackHMMER, though some alignments did have a high ‘bias’ score indicating that they may be spurious matches. Multiple sequence alignment of these four sequences identified a conserved N-terminal region after the signal peptide with several conserved cysteine, proline, aspartic acid and glycine residues. BEC2 and GoEC2 were more similar than BEC4 and MoCDIP2.

## Discussion

With a growing number of experimentally confirmed fungal ‘effector’ proteins in the public domain (Table S3) [91], there are emerging opportunities to mine this data and develop improved methods for effector and virulence factor discovery. However, basic homology-inference methods cannot necessarily be applied, as many known effector proteins are either sufficiently divergent or of independent origin to prevent their grouping into larger ‘effector families’. Comparisons between effector proteins and candidates at the structural level have indicated recognizable structural similarity between many emerging groupings, including the ToxA-like [21, 22], MAX [23], RALPH [24, 58, 59] and Hce2 [155] families. Tertiary structural homology may become the basis for reliable effector prediction in future studies; however, the application of protein structure prediction to large sets of effector candidates is not currently computationally feasible. This study applied a highly sensitive sequence clustering approach - termed ‘RemEff’ - to a large protein dataset to form novel protein clusters, leveraging known effectors to identify effector ‘family’ clusters and predict homologous effector candidates within them by association.

While the RemEff method has taken a ‘top-down’ approach that has identified a large number of ‘effector families’, we focus here on selected examples. In our first detailed example, we presented a previously undescribed expanded family of effectors containing the effectors *Leptosphaeria maculans* AvrLm6 [47], *Magnaporthe oryzae* BAS4 and SPD5 [109, 110], *Fusarium oxysporum* f. sp. *lycopersici* SIX5 [111] and *Cercospora beticola* NIP1 [112]. Each study describing the effectors has noted the presence of homologues of these effectors in multiple species. Numerous homologues of AvrLm6 have been previously observed in *Venturia*, *Colletotrichum* and *Fusarium* species [48, 49]. However, not all *Venturia* AvrLm6 homologues published by Shiller *et al*. [49] were identified as members of this superfamily. In that study the only restriction on matches was a maximum PSI-blast *e*-value of 10^−2^, so it is likely that the focus here on finding full-length homologues might have excluded these potential matches. Each of the five level 2 clusters within the level 3 cluster had a different cysteine spacing pattern, with four or six conserved cysteine positions each. Some cysteine residues were conserved across multiple groups, and two positions were conserved in all subgroups suggesting their functional relevance.

None of these SIX5-like effectors have yet been structurally determined. SIX5 appears to interact with plasmodesmata and mediates the intercellular translocation of another effector Avr2 (where it can then exert virulence promoting and avirulence function) [156]. Intriguingly, another pair of *Leptosphaeria maculans* effectors not present in this study, one of which is a SIX5 homologue, appear to show a similar interaction in that pathosystem [157]. *Magnaporthe* biotrophy-associated secreted protein 4 (BAS4) elicits a host defence response late in the biotrophic phase, which promotes cell death during the necrotrophic phase [158, 109]. Cytoplasmic effectors PWL2 and BAS1, but not BAS4, move from cell to cell preceding the invasive hyphae (IH), possibly through plasmodesmata [159]. Suppressor of cell death 5 (SPD5) is a known homologue of BAS4, which suppresses BAX- and NEP1-induced cell death [110]. CbNIP1 induces light-independent necrosis [112], but its specific activity and cell localization is unknown. In contrast to SIX5, which internalizes into the host cytoplasm [156], BAS4 is accumulated in the apoplast [158]. The host internalization mechanisms of most effector proteins are not well understood, but some may require short, conserved, amino acid motifs [25, 160]. If this is also the case for SIX5, these internalization motifs are not likely to be the conserved pattern or structure being detected by RemEff across the SIX5-like cluster. In a similar vein, previous tertiary structure comparisons (although not RemEff) had defined the MAX-effector group [23] which also grouped several cytoplasmic *Magnaporthe* effectors with the apoplastic *Pyrenophora* ToxB [161]. *Magnaporthe oryzae* appears to have a distinct mechanism for effector host-internalization involving a specialized infection structure – the biotrophic interfacial complex (BIC) – and via extracellular vesicles [162]. The necrotrophic *Pyrenophora* spp. lack such structures. We surmise that for both the SIX5-like cluster and the MAX family, any common functions related to detectable conserved structures are unrelated to cell internalization.

In our second detailed example, we compared two other clusters containing the effectors ToxA and AvrFOM2, which were previously reported as similar [22]. The cluster containing AvrFOM2 is much larger and more sequence diverse compared to the one containing ToxA. Within the ToxA level 3 cluster are only the canonical ToxA-like effectors of *Parastagonospora* spp., *Pyrenophora* spp., *Bipolaris sorokiniana* and *B. maydis*, many of which are identical and are thought to have arisen by a complex horizontal transfer event [32]. The level 3 cluster containing AvrFOM2 and ChEC13 overlaps considerably with the candidate homologues identified in Lu *et al*. [21]. Although a *Fusarium oxysporum f.* sp*. melonis* homologue was described in that paper, it does not appear to have been AvrFOM2. The multiple sequence alignment does show the conservation of some of the motifs previously described [21], including the LXXR pro-peptide cleavage site, and the three motifs found in beta sheets 4 (LXVXIXN, here replaced by IXVXIXN in PC_07OLPP containing AvrFOM2), 5 (LILTXY, replaced by I[VI]LSNY in PC_07OLPP) and 8 (WXXQ). However, neither the asparagine rich motif (WXXN(S)NXIXVXI) nor the RGD/SGN motif were observed. The level 2 cluster containing AvrFOM2 and ChEC13 (PC_07OLPP) exhibits a number of phenylalanine residues in the signal peptide (SP) at the junction of the N-region and the hydrophobic core. Hydrophobic amino acids near the N-region tend to decrease secretion efficiency [163] and although phenylalanine residues are found in the hydrophobic core regions of human signal peptides, it is not generally known in yeasts [164]. However, the amino acid composition of efficient signal peptides can vary between species, and the hydrophobic and N-terminal regions of the SP may be involved in directing proteins through different secretion pathways [163].

In our last detailed example including ribotoxins and RALPH effectors, the clusters containing ribotoxins (Zt6) and RNase-like proteins associated with haustoria (RALPH) effectors formed distinct clusters, but a clear similarity existed at specific regions between clusters PC_032CHK containing Zt6 and PC_04SK9M containing AvrPm2 and BEC1054. In the ribotoxin sequence ɑ-sarcin the active sites are Histidine in the YPH motif, Glutamine in EFP motif, and a Histidine between the last two cysteine residues, all of which are missing in RALPHs though they possess the conserved surrounding sequence of the former two [165–167]. Additionally, all RALPH sequences lacked the extended N-terminal loop that has previously been thought to be necessary for ribotoxin activity, though it was also poorly conserved in the cluster containing Zt6 [7]. Overall, the profiles of RALPH effectors, with only two conserved cysteine positions, is more like RNase T1 than the ribotoxins, are missing many of the previously described active sites, and have shorter loop sequences than the canonical ribotoxins. This is consistent with previous structural prediction analysis [58], and makes sense given that *Blumeria graminis*, to which this group appears to be restricted, are obligate biotrophs which would not benefit from effectors with cytotoxic activity. This also supports speculation that BEC1014 acts as a pseudoenzyme, binding host ribosomes but not cleaving the SRL [59]. Both AvrPm3^a2/f2^ and the suppressor SvrPm3^a1/f1^ form distinct level 2 clusters branching from the main group of RALPH effectors (PC_01D3OM and PC_0278ZT, respectively). SvrPm3^a1/f1^ was originally described as being a member of the RALPH group, but AvrPm3^a2/f2^ was not [24]. It has previously been demonstrated that high expression of SvrPm3^a1/f1^ suppresses the recognition of AvrPm3^a2/f2^ by Pm3 receptors [168], and that positive selection in the *avrpm3^a2/f2^* gene does not appear to be related to evasion of recognition by Pm3 [169]. Although the clusters are quite distinct, their association may suggest a possible mode of SvrPm3^a1/f1^ suppression, where it may act as a ‘bodyguard decoy’ to AvrPm3^a2/f2^ [170]. However, we note that the level 2 clusters containing AvrPm3^a2/f2^ and SvrPm3^a1/f1^ may be poorly aligned here, and AvrPm3^a2/f2^ shares little conserved sequence similarity with the other RALPH effectors beyond the signal peptide and the cysteine positions.

Several other effectors formed groups of more than one effector, including two that have previously been unreported: AvrLm2 and SIX1 [113, 114], and *Puccinia striiformis* Shr4 and Shr6 [124]. However, in addition to the groupings that the RemEff method has formed between known and candidate effector proteins, the absence of predicted groupings may also offer biological insights. The presence of effectors in ‘orphan’ clusters might be an indicator of their evolutionary histories involving either high sequence divergence or independent origin.

There were some notable cases where RemEff reported clusters that conflicted with past reports of fungal effector families. The AvrFOM2 level 3 cluster described above, which contained sequences that were previously reported to be ToxA-like [21, 22], failed to group with the ToxA (level 3) cluster despite weak overall sequence similarity (Fig. 4). Similarly, RemEff also failed to group the previously described MAX family proteins [23] into a single component. Our analysis of JackHMMER results did identify a potential relationship between ToxA-like proteins and AvrFOM2 and also recovered many of the relationships identified by our clustering method, including some members of the SIX5-like family but failed to identify similarity between any canonical MAX family members. An important distinction is that progressive search methods (e.g. JackHMMER or HHBlits) search for sequence homologues from a single query sequence. Whereas our clustering method extends the results of these search methods to identify families of reciprocally matching, full length homologues at a higher level of stringency, with the network of clusters representing the structure within and between these families. Additionally, detecting low sequence identity matches to a single query sequence may require manually evaluating matches (as was done with the JackHMMER results in this study), whereas clustering and HMM-HMM comparisons used by RemEff are an automated and unbiased procedure. Where past studies have used progressive searches to focus on a single or reduced set of effectors (e.g. AvrLm6, Zt6, MAX), RemEff mines a larger dataset spanning multiple fungal species which greatly increases its predictive potential.

Some RemEff clusters also contained proteins from non-pathogen species – including saprotrophs – which may initially appear to be in error. However, these results may be valid and worthy of deeper investigation on a case-by-case basis. The presence of false positive predictions of effector-like small secreted proteins (SSPs) in saprotrophs poses a challenge to several effector prediction methods, and it has been reported that all fungal species may have ~40–60 % effector-like SSP predictions within their secretomes: [171]. Nevertheless, some species that have been conventionally regarded as unequivocally saptrotrophic have recently had new evidence supporting rarely observed pathogenic modes [172]. For example, *Neurospora crassa* had long been regarded as the model saprotroph, yet we observed its proteins in ten level 3 clusters that contained known effectors (Table S4). *N. crassa* has been recently isolated as an pathogen of Scots pine [173], and its CAZyme content indicates this may resemble a biotrophic interaction [172]. Additionally, a proportion of fungal effectors are assumed to have broadly cytotoxic functions (albeit with a diverse range of mechanisms). While this study focusses on effectors that target host plant cells, there may be some functional overlap with cytotoxic peptides targeting other types of cells. Saprotrophic yeast species (also present in several RemEff clusters, Table S4) may still require mechanisms to compete with foreign microbes in a resource-scarce environment, e.g. via secretion of yeast killer toxins. It is not unreasonable to allow for the possibility that some plant pathogen effectors may share distant, underlying structural homology to certain saprotroph proteins and cytotoxic peptides from other organisms with vastly different lifestyles, including but not limited to: antimicrobial peptides (AMPs), defensins, conotoxins and venoms. A RemEff cluster matching any of these classes of non-plant pathogen proteins would be considered worthy of experimental follow-up.

## Conclusion

The RemEff method predicts remote homology relationships between known effectors and candidate effector proteins, allowing for the prediction of distantly related effector ‘families’ in plant-pathogenic fungi. HMM-HMM clustering based on pattern and/or structural similarities can be useful in organizing known effectors and predicting novel ones, however much more work will be needed to draw links between any structural and functional similarities that have been highlighted – which is far beyond the scope of this current study. We cannot demonstrate that pattern-based/structural homology detected by RemEff is consistently grouping clusters based on common internalisation, pathogenicity or other functions. We assume that there is a reasonable possibility of a common function within each cluster based on their similar sequence or secondary structure patterns, and that by using known effectors as initial ‘seeds’, most of the resulting clusters will be relevant to pathogenicity and useful in an effector discovery context. We have made RemEff’s underlying profile data available for further analysis (https://figshare.com/projects/Effector_protein_remote_homology/87965), which can serve as a useful resource for future plant pathology studies.

We have presented case studies of novel effector family groupings that both demonstrate the utility of this method to enhance effector discovery research, and highlight important similarities and differences between effector family sub-groups. This is illustrated well by sequence logos generated from the AvrLm6-like (Fig. 3) and ribonuclease (Fig. 5) level 2 clusters, which show a combination of overall conservation and motif diversity. We observe cysteine spacing to be a major conserved feature, sometimes in the absence of other defining sequence features. Given the potential overlap in modes of action of some fungal effectors and other non-fungal cytotoxic peptides, we speculate that it may be useful to further explore conservation of cysteine-spacing as a heuristic classification system for some groups of fungal effectors, similar to those that have been established for arthropod venoms [28, 174] and for snail conotoxins [63]. Alternatively, as protein structure prediction methods become more feasible to apply at large scale, it may become possible to predict effector candidates on the basis of structure modelling and molecular docking simulation. However, given the number and diversity of ‘effector’ proteins and their functions, we anticipate that neither method would be broadly applicable, and maintaining an ensemble of profile HMMs will be preferable for the foreseeable future.

## Supplementary Data

Supplementary material 1

Supplementary material 2

Supplementary material 3

Supplementary material 4

Supplementary material 5

Supplementary material 6

Supplementary material 7

Supplementary material 8

Supplementary material 9

Supplementary material 10

Supplementary material 11

Supplementary material 12

Supplementary material 13

Supplementary material 14

Supplementary material 15

Supplementary material 16

Supplementary material 17

Supplementary material 18

Supplementary material 19

Supplementary material 20
