# Supplementary material for: Remote homology clustering identifies lowly conserved families of effector proteins in plant-pathogenic fungi
Source: Microb Genom. 2021 Sep 1;7(9):000637. doi: 10.1099/mgen.0.000637 (PMC8715435; doi:10.1099/mgen.0.000637)
Supplement: Supplementary material 1 [file mgen-7-0637-s001.pdf]

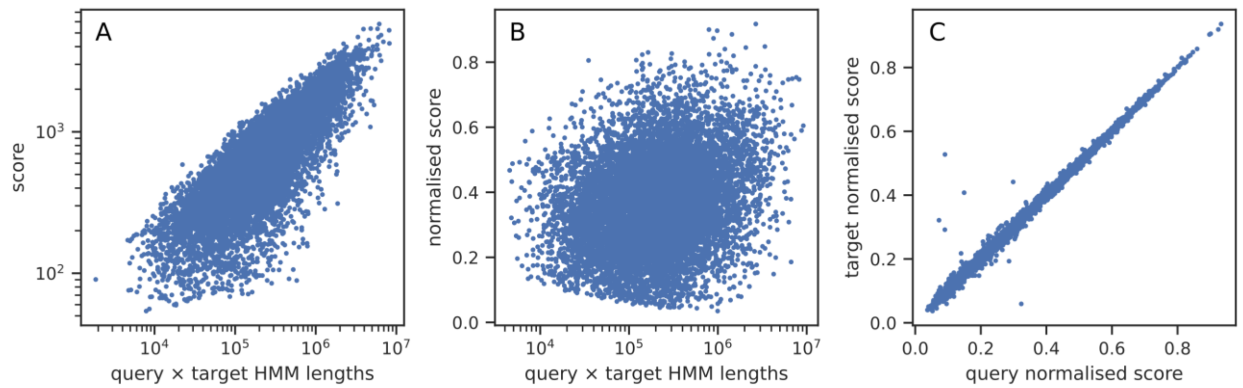

Fig. S1. HHBlits alignment score length normalisation. A) Alignment scores show a strong correlation with the product of HMM lengths in a log-log space for the top 10 matches of each query. B) The scores after normalisation show little dependence on the product of HMM lengths. C) The two normalised scores for each pair of a significant match (i.e. A vs B and B vs A) are highly correlated, indicating that the arithmetic mean is a reasonable combination of the scores.
