# Supplementary figures and images for: Remote homology clustering identifies lowly conserved families of effector proteins in plant-pathogenic fungi
Source: Microb Genom. 2021 Sep 1;7(9):000637. doi: 10.1099/mgen.0.000637 (PMC8715435; doi:10.1099/mgen.0.000637)

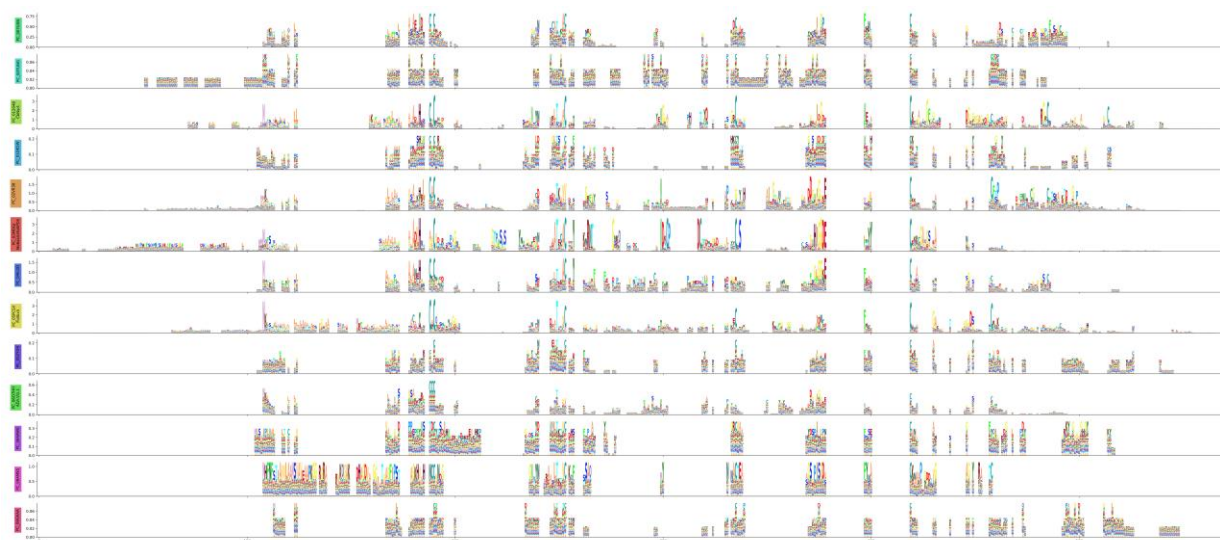

**Fig. S2.** Unfiltered logos for Six5-like group.

Supplement: Supplementary material 3 [file mgen-7-0637-s003.pdf]

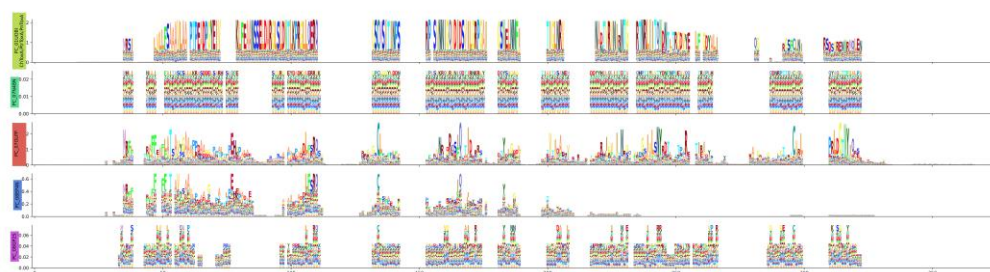

**Fig. S3.** Unfiltered logos for ToxA-like group.

Supplement: Supplementary material 5 [file mgen-7-0637-s005.pdf]

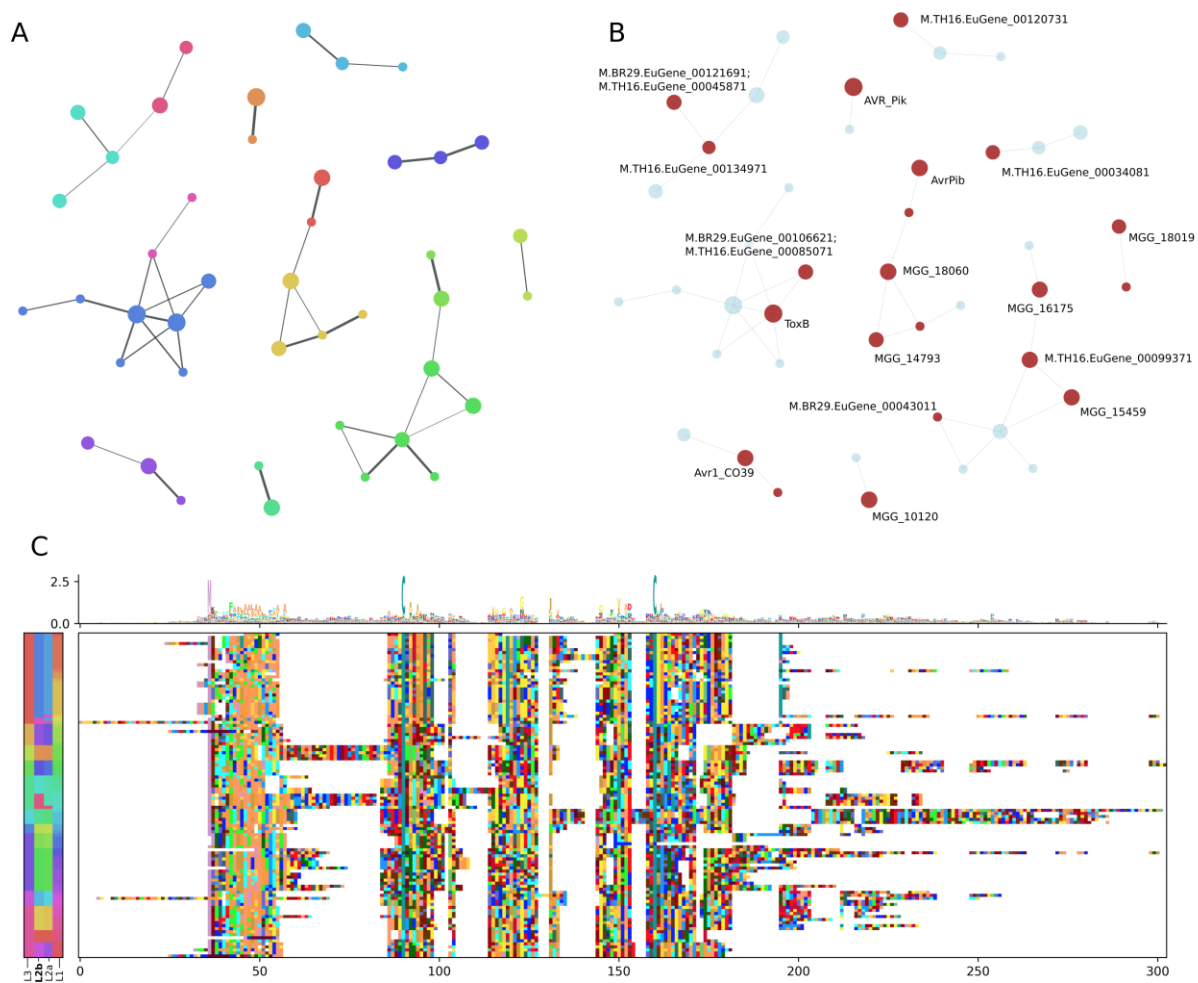

**Fig. S4.** MAX graph and alignment (unfiltered).

Supplement: Supplementary material 7 [file mgen-7-0637-s007.pdf]

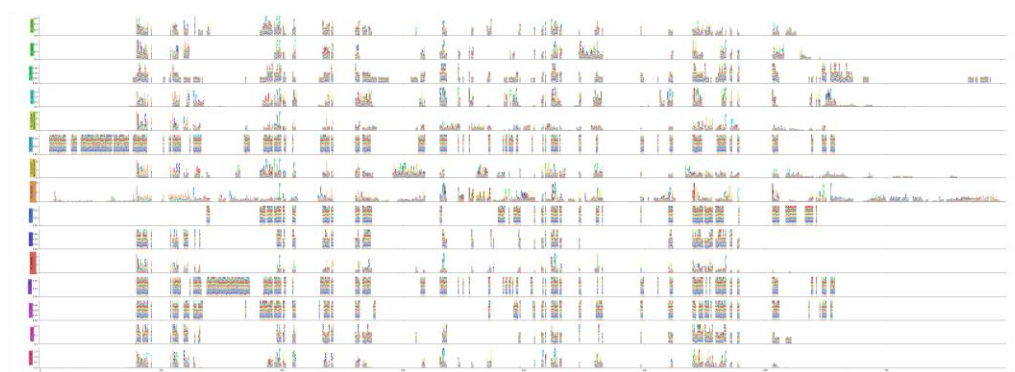

**Fig. S5.** Unfiltered logos for RNase-like effector group.

Supplement: Supplementary material 9 [file mgen-7-0637-s009.pdf]
