# Supplementary material for: Remote homology clustering identifies lowly conserved families of effector proteins in plant-pathogenic fungi
Source: Microb Genom. 2021 Sep 1;7(9):000637. doi: 10.1099/mgen.0.000637 (PMC8715435; doi:10.1099/mgen.0.000637)
Supplement: Supplementary material 12 [file mgen-7-0637-s0012.zip › supplementary_data_06-remeff_scripts/07-find_effector_clusters.html]

07-find\_effector\_clusters


In [1]:

```
import os
from shutil import rmtree
from collections import defaultdict

import numpy as np
import pandas as pd
from Bio.SeqUtils.CheckSum import seguid
from Bio.SeqRecord import SeqRecord
from Bio.Seq import Seq
from Bio import SeqIO
```

First I'll run a search of our "effector-like" sequences against the full protein dataset.

Because we want to match the exact sequence, we restrict the search matches to 90% identity, and 90% reciprocal coverage.

In [2]:

```
%%bash
set -eu

rm -rf -- tmp
mkdir -p tmp tmp/tmp

mmseqs createdb ./data/nr.fasta tmp/target
mmseqs createdb ./data/seqs_to_search.fasta tmp/query

mmseqs search tmp/query tmp/target tmp/matches tmp/tmp --min-seq-id 0.9 -c 0.9 --cov-mode 0

# Format mode 2 is BLAST-TAB + query/db length
mmseqs convertalis tmp/query tmp/target tmp/matches 07-find_effector_clusters-mmseqs_matches.tsv --format-mode 2
```

```
Process is terminated.
```

Now we load the search results and select the best match by bitscore.

In [2]:

```
matches = pd.read_csv(
    "07-find_effector_clusters-mmseqs_matches.tsv",
    sep="\t",
    names=["query", "target", "pid", "alnlen", "nmismatch",
           "ngap", "qstart", "qend", "tstart", "tend",
           "evalue", "bits", "qlen", "tlen"]
)
matches = matches.sort_values(["query", "bits"], ascending=False).groupby("query").first().reset_index()
matches = matches[["query", "target"]].rename(columns={"query": "effector", "target": "unique_sequence_id"})
matches["effector_database"] = matches["effector"].apply(lambda x: "phibase" if "#PHI:" in x else "custom")
matches
```

Out[2]:

|  | effector | unique\_sequence\_id | effector\_database |
| --- | --- | --- | --- |
| 0 | A0A016PMX1#PHI:6978 | PC\_00HIMS | phibase |
| 1 | A0A016PTV5#PHI:5393 | PC\_03ZP3E | phibase |
| 2 | A0A016PUA3#PHI:3659 | PC\_0009RR | phibase |
| 3 | A0A016PW58#PHI:3658 | PC\_03Q2UH | phibase |
| 4 | A0A016PX00#PHI:4602 | PC\_05L1QU | phibase |
| ... | ... | ... | ... |
| 3703 | bgh06532\_CSEP0264\_BEC1011 | PC\_019FR5 | custom |
| 3704 | bghG001947000001001\_CSEP0340 | PC\_042HHN | custom |
| 3705 | bghG002857000001001\_CSEP0371 | PC\_06YKL9 | custom |
| 3706 | bghG002872000001001\_CSEP0374 | PC\_029V3B | custom |
| 3707 | bghG008575000001001\_CSEP0486 | PC\_019FR5 | custom |

3708 rows × 3 columns

Cool. Now I want to check that all of the sequences with exact matches are in there, just in case there is some weird complexity filtering thing happening.

In [3]:

```
list(matches.loc[matches["effector_database"] == "custom", "effector"])
```

Out[3]:

```
['AKM21218.1',
 'ALVi.1389.11',
 'ALVi.1389.12',
 'ALVi.1389.2',
 'ALVi.1389.3',
 'ALVi.1389.4',
 'ALVi.1389.5',
 'ALVi.1389.6',
 'ALVi.1389.7',
 'ALVi.Vi1.1',
 'ALVi.Vi1.10',
 'ALVi.Vi1.13',
 'ALVi.Vi1.15',
 'ALVi.Vi1.16',
 'ALVi.Vi1.17',
 'ALVi.Vi1.18',
 'ALVi.Vi1.19',
 'ALVi.Vi1.20',
 'ALVi.Vi1.21',
 'ALVi.Vi1.22',
 'ALVi.Vi1.3',
 'ALVi.Vi1.4',
 'ALVi.Vi1.5',
 'ALVi.Vi1.6',
 'ALVi.Vi1.7',
 'ALVi.Vi1.8',
 'ALVi.Vi1.9',
 'ALVp_11032.1',
 'ALVp_11032.12',
 'ALVp_11032.13',
 'ALVp_11032.14',
 'ALVp_11032.15',
 'ALVp_11032.3',
 'ALVp_11032.4',
 'ALVp_11032.5',
 'ALVp_11032.6',
 'ALVp_11032.7',
 'BfNep1',
 'BghAvr_a1',
 'BghAvr_a13',
 'BghBEC1054',
 'BghBEC2',
 'BghCSEP0055',
 'BghCSEP0105',
 'BghCSEP0162',
 'BgtAcSP_30091',
 'BgtAvrPm2',
 'BgtAvrPm3a2f2',
 'BgtE_10117',
 'BgtE_10124',
 'BgtE_20000',
 'BgtE_20025',
 'BgtE_20026',
 'BgtE_20090',
 'BgtE_5664',
 'BgtE_5665',
 'BgtE_5842',
 'BgtE_5843',
 'BgtE_5846',
 'BgtE_5901',
 'BgtSvrPm3a1f1',
 'CCE26798.1',
 'CCE26799.1',
 'CbNip1',
 'CfAVR2',
 'CfAVR4',
 'CfAVR4E',
 'CfAVR9',
 'CfAvr5',
 'CfEcp1',
 'CfEcp2',
 'CfEcp4',
 'CfEcp5',
 'CfEcp6',
 'CfTom1',
 'CgDN3',
 'Cgfl',
 'ChEC13',
 'ChNIS1',
 'ChToxA',
 'CoDN3',
 'EKG15312.1',
 'EME45057.1',
 'EUC36307.1',
 'EUC44184.1',
 'EUN25607.1',
 'EXF72942.1',
 'FGL1',
 'FOMG_19011',
 'FoeNEP1',
 'FolSix1',
 'FolSix10',
 'FolSix11',
 'FolSix13',
 'FolSix14',
 'FolSix2',
 'FolSix3',
 'FolSix4',
 'FolSix5',
 'FolSix6',
 'FolSix7',
 'FolSix8',
 'FolSix9',
 'Fom_2',
 'GiSP7',
 'GoEC2',
 'KEQ67658.1',
 'KEQ81621.1',
 'KJY01809.1',
 'KNG46663.1',
 'KXT12958.1',
 'LmAvrLm1',
 'LmAvrLm11',
 'LmAvrLm2',
 'LmAvrLm3',
 'LmAvrLm4_7',
 'LmAvrLm6',
 'LmAvrLmJ1',
 'M.BR29.EuGene_00004921',
 'M.BR29.EuGene_00041131',
 'M.BR29.EuGene_00043011',
 'M.BR29.EuGene_00060181',
 'M.BR29.EuGene_00081821',
 'M.BR29.EuGene_00082031',
 'M.BR29.EuGene_00085071',
 'M.BR29.EuGene_00087671',
 'M.BR29.EuGene_00088411',
 'M.BR29.EuGene_00091361',
 'M.BR29.EuGene_00091681',
 'M.BR29.EuGene_00095641',
 'M.BR29.EuGene_00106461',
 'M.BR29.EuGene_00107481',
 'M.BR29.EuGene_00112111',
 'M.BR29.EuGene_00113041',
 'M.BR29.EuGene_00118801',
 'M.BR29.EuGene_00119491',
 'M.BR29.EuGene_00119511',
 'M.BR29.EuGene_00121691',
 'M.BR29.EuGene_00125811',
 'M.BR29.EuGene_00126081',
 'M.TH16.EuGene_00000541',
 'M.TH16.EuGene_00027191',
 'M.TH16.EuGene_00027411',
 'M.TH16.EuGene_00034081',
 'M.TH16.EuGene_00040131',
 'M.TH16.EuGene_00045871',
 'M.TH16.EuGene_00079081',
 'M.TH16.EuGene_00079311',
 'M.TH16.EuGene_00099371',
 'M.TH16.EuGene_00101881',
 'M.TH16.EuGene_00106621',
 'M.TH16.EuGene_00120731',
 'M.TH16.EuGene_00124981',
 'M.TH16.EuGene_00127871',
 'M.TH16.EuGene_00134971',
 'M.TH16.EuGene_00135161',
 'MGG_00821',
 'MGG_04384',
 'MGG_08482',
 'MGG_10120',
 'MGG_14793',
 'MGG_14834',
 'MGG_15207',
 'MGG_15459',
 'MGG_16058',
 'MGG_16113',
 'MGG_16175',
 'MGG_16619',
 'MGG_17132',
 'MGG_17255',
 'MGG_18019',
 'MGG_18060',
 'MIAvrM14',
 'Mg1LysM',
 'Mg3LysM',
 'MgxLysM',
 'MiSSP7_2',
 'MlAvrL2',
 'MlAvrL567_A',
 'MlAvrL567_B',
 'MlAvrL567_C',
 'MlAvrL567_D',
 'MlAvrM',
 'MlAvrP123',
 'MlpCTP1',
 'MoAVR_Pia',
 'MoAVR_Pii',
 'MoAVR_Pik',
 'MoAVR_Pita',
 'MoAVR_Pita2',
 'MoAvr1_CO39',
 'MoAvrPi9',
 'MoAvrPib',
 'MoAvrPiz_t',
 'MoBAS162',
 'MoBas1',
 'MoBas107',
 'MoBas2',
 'MoBas3',
 'MoBas4',
 'MoCDIP1',
 'MoCDIP2',
 'MoCDIP3',
 'MoCDIP4',
 'MoCDIP5',
 'MoHEG13',
 'MoIug6',
 'MoIug9',
 'MoMC69',
 'MoMSP1',
 'MoNIS1',
 'MoPwl1',
 'MoPwl2',
 'MoPwl3',
 'MoPwl4',
 'MoSPD10',
 'MoSPD2',
 'MoSPD4',
 'MoSPD5',
 'MoSPD7',
 'MoSPD9',
 'MoSlp1',
 'MpNEP1_1',
 'MpNEP1_2',
 'Mycgr3G38105',
 'OAL46779.1',
 'OCK93132.1',
 'PGTAUSPE_10_1',
 'PGTG_10537.2',
 'PGTG_16791',
 'PIIN_08944',
 'PgShr8',
 'PgShr9',
 'PgtAvrSr35',
 'PgtAvrSr50',
 'PiFGB1',
 'PnTox1',
 'PnTox3',
 'PnToxA',
 'PpCSEP-07',
 'PpCSEP-09',
 'PsShr1',
 'PsShr2',
 'PsShr3',
 'PsShr4',
 'PsShr5',
 'PsShr6',
 'PsShr7',
 'PtrToxA',
 'RiCRN1',
 'RiSIS1',
 'RsNIP1',
 'RsNIP2',
 'RsNIP3',
 'SrSAD1',
 'SrrPit2',
 'SrzPit2',
 'SsSSITL',
 'SsSSVP1',
 'UfRTP1',
 'UhAvr1',
 'UmCmu1',
 'UmPep1',
 'UmPit2',
 'UmSee1',
 'UmTin2',
 'Umeff1-1',
 'VdAve1',
 'VdNEP',
 'VdPevD1',
 'VdSCP7',
 'Vdlsc1',
 'XP003298007.1',
 'XP_001800520.1',
 'XP_001935864.1',
 'XP_007580207.1',
 'XP_007678837.1',
 'XP_007686215.1',
 'XP_007699156.1',
 'XP_008025738.1',
 'XP_013344128.1',
 'XP_013431971.1',
 'XP_014077357.1',
 'XP_016761200.1',
 'XP_018034946.1',
 'Zt103264',
 'Zt80707',
 'Zt89160',
 'ZtAvrStb6',
 'ZtNIP1',
 'ZtNIP2',
 'bgh01362_CSEP0027',
 'bgh01363_CSEP0028',
 'bgh02875_CSEP0065',
 'bgh02877_CSEP0066',
 'bgh03782_CSEP0152',
 'bgh06532_CSEP0264_BEC1011',
 'bghG001947000001001_CSEP0340',
 'bghG002857000001001_CSEP0371',
 'bghG002872000001001_CSEP0374',
 'bghG008575000001001_CSEP0486']
```

In [4]:

```
def tab_to_checksum(v):
    seq = v.strip().rstrip("*").upper()
    return seguid(seq)
```

In [5]:

```
sequences = SeqIO.to_dict(SeqIO.parse("data/seqs_to_search.fasta", "fasta"))
```

In [6]:

```
sequences_checksums = defaultdict(list)
for seq in sequences.values():
    sequences_checksums[tab_to_checksum(str(seq.seq))].append(seq.id)

sequences_checksums
```

Out[6]:

```
defaultdict(list,
            {'FM7BWC3nuHjDWZnK9THzRCXW4jM': ['PsAvrRpm1',
              'Q4ZYH0#PHI:3495__PHI:5385__PHI:5501'],
             'GaxJoKBEQnM+SQX2IIf6Vw8oZd0': ['PsAvrRps4',
              'Q52432#PHI:978__PHI:8026'],
             'zK1n8zy7kkZLAhzWVWBASc6erFM': ['BhNEP1'],
             'C+/HOG5VWLFrX4wJ6w3tRg0Yiy4': ['PaNPP1'],
             'CjDiCrqVkk50RzaU1ywNATuaoRs': ['VpNPP1'],
             'XS99vsGJH7hOuLDiMLIaG4oW04I': ['PsAvrA'],
             'Y3iulYgdEpbZ/vRi4SguvT3yxcU': ['PsAvrB1',
              'Q48B66#PHI:3493',
              'Q52378#PHI:966'],
             'Xi8JKQEP9PPcqo5h75ERY+yyLqA': ['PsAvrB2',
              'Q4ZX49#PHI:967__PHI:5384'],
             '9wdThfZrUKLtfh4bANgbHmapA7Y': ['PsAvrD',
              'Q48B68#PHI:589__PHI:3494'],
             'qFGtqOvbxSmS3cLZFoImWecw8pw': ['PsAvrE1', 'Q887C9#PHI:6377'],
             '9cRDSwXIzHv2/qwhglqiiih/rX8': ['PsAvrE2'],
             'LmoRT3s2UO4cG5loFrmPfM1rJLI': ['PsAvrE3'],
             '0sBOl4f5qrz+zjJVjnoJ6tZPMOA': ['PsAvrPto1', 'Q87Y16#PHI:975'],
             'ydGIsNlBvQIKIcDXuFusYYJn+eU': ['PsAvrPto2', 'Q4ZLM6#PHI:976'],
             'xkYNafUmDD2wGPksHNm9iaBf3RU': ['PsAvrRpt2'],
             'f9YA1+BWYLOq86USsV9h/kHe6GM': ['PsHopA'],
             'Uz4Rrfs7v1kyotH3Z7KTRLP/2Sg': ['PsHopB'],
             '1DBaPjldgKL3gNqUegNYINM94CQ': ['PsHopC'],
             'wXvakuu0zMAEAJdXFQTkdkXifHg': ['PsHopD1'],
             'lJ+D0/5uDvZzIXXCQO4Xp2S0AV4': ['PsHopD2'],
             '5gRgejx/ovvvhakbWimJNiDPHsA': ['PsHopE', 'Q87X57#PHI:6899'],
             '5vm/4k1aAzs3JxwyEGg202F6W60': ['PsHopF1', 'Q88A90#PHI:3920'],
             'w+aoXY0I7MgS1UhstNKA94uEJ7c': ['PsHopF2'],
             'fU2yFmSNGdIzrdiMRtvjgsbR6jI': ['PsHopG1', 'Q87W42#PHI:6353'],
             'tkQPPUm2BpDOwcQ66B7NpaR5MkQ': ['PsHopG2'],
             'I48vejBLp2NkOyCZSaZQIE1Y7Qo': ['PsHopH1'],
             'SjligoT59ELPa1X/m1O+EDBUga0': ['PsHopH2'],
             'wwWHEis46v9Zqx9zKEGm6mkNQdE': ['PsHopI1', 'Q87W07#PHI:7337'],
             '7j8FIwEk3t6b2RaD/U/URgPLZzA': ['PsHopI2'],
             '4a/0ikLTCTPst79GAjsU5XHMgHw': ['PsHopI3'],
             '5ItV+/KSZcTA1fItUsLNT8Yrxso': ['PsHopK',
              'Q88BH0#PHI:4018__PHI:8027'],
             'ejsbaSRxv1lKKGQ1kFszqFxcrec': ['PsHopM1',
              'Q887D0#PHI:982__PHI:4008__PHI:6378'],
             'PMdolnLOJqXIdrXeoxfBsC/lr2c': ['PsHopM2',
              'Q4ZX82#PHI:998__PHI:8430'],
             'o1p7ISv5ffRp/Xb+08p37W5wnE8': ['PsHopN', 'G3XDC5#PHI:983'],
             'HGy7wQKLKpo+IiWtll1Ugcqbb1Y': ['PsHopO', 'Q88BP8#PHI:586'],
             '3KqYNmIBBYQTlVE8UyPEcjpVJck': ['PsHopQ1',
              'Q888Y7#PHI:984__PHI:2752__PHI:2879__PHI:8329'],
             'OrFv/a6xqvELqS22rqgEKKRDw54': ['PsHopQ2'],
             'Hv6lePX/7TgEQ5CuriS494PQaaI': ['PsHopR1'],
             'KQeXTiVCFYCUvoUekOUgH0L5iyk': ['PsHopR2'],
             'Uy/r3k/fwAFvapTvk4z2FKtgy3A': ['PsHopS'],
             'iLu5Ius551hSCkaqL/dJfS7OW44': ['PsHopT'],
             'CZffg4prB5HrZ2qdYvleUOPkpr0': ['PsHopU'],
             'Nanr5V657WAVIPXk0QATHTYXYL0': ['PsHopV1'],
             'FP5CO5r8mml1gHs2YVRQEDtiPXQ': ['PsHopV2'],
             '9p7nrqMRMvnTGIAaboosZ3wB2Hg': ['PsHopW'],
             'TYE5y/TZMQi6mbOYA74/5OpheSE': ['PsHopX1'],
             '1rh3O4S2naLc+Ap+WocA95yDJGo': ['PsHopX2'],
             '5fIhanD3mYNo+bWF73UP50VkgF0': ['PsHopY'],
             'jxczTXKG3naLhhbkMtc63e2PedE': ['PsHopZ',
              'Q4ZX47#PHI:996__PHI:4968__PHI:5383__PHI:8426'],
             'Yw+qoPawJqHwtIgub3AYoyOcEw4': ['PsHopAA1', 'G3XDB9#PHI:987'],
             've4Q7HnNct5q+yAKIrHMPn/Qzks': ['PsHopAA2', 'Q4ZX85#PHI:997'],
             '5DkeTu1Xhx/Yc5xxJoFPXSPtF2U': ['PsHopAB1',
              'Q8RSY1#PHI:990__PHI:2743'],
             '9FYvE4d/c8OuraRJOu4YTALxFhY': ['PsHopAB2', 'Q9RBW3#PHI:989'],
             'd13yHVaaiJlqP4DhChY4okxLekc': ['PsHopAB3', 'Q4ZMD6#PHI:999'],
             '9kW8zn5iHdVBZmXREBkuCptWdeQ': ['PsHopAD'],
             'T19NPEIbo2wd+5+wygVxArkisEA': ['PsHopAE1'],
             'vWiSaRxiZ0+lvETGtu/CzteRxPQ': ['PsHopAE2', 'Q7PC62#PHI:995'],
             'lo2fVEUSyaGzb10m7RHxxD2Z3yQ': ['PsHopAF1', 'Q886L1#PHI:6365'],
             'EMmSNQ+nCTiJwOhI8kiQyvFEvb0': ['PsHopAF2'],
             'qzhsTSBjh/AW0///oJzBz729et4': ['PsHopAF3'],
             'VrLCSQoj317NmVGnfiBzjs/fEow': ['PsHopAG'],
             'kx6p4LNv5g6P4DcsuyLqWuTvvsw': ['PsHopAH1', 'Q888W1#PHI:581'],
             'po5P7B6VbU3JHF6VruxEqEH0H/k': ['PsHopAH2'],
             'rLzwWyOzBeLcHMdxbYdyR31G9ck': ['PsHopAI',
              'Q888W0#PHI:582__PHI:7336'],
             'NHm4nkVZFdAaZh98goChYdnkXA4': ['PsHopAL'],
             'cDZID3KnAThu222AA9rGB4iF+Ho': ['PsHopAM', 'Q52431#PHI:991'],
             'MesxihUtoyGPBXACD8SL1QzgsHE': ['PsHopAO',
              'Q79LY0#PHI:992__PHI:7237__PHI:7265'],
             'apVTPv9OP9KxGUqzo+0A+tuN3qw': ['PsHopAQ'],
             'jo5ksv+tZsmmIEQ83PoCUVgDyAI': ['PsHopAR', 'Q52430#PHI:993'],
             'YNA5euIX0SOQe3yWmddYUqnPN6g': ['PsHopAS'],
             'oNf9ZYuJdDblmLRptaoimbBI5MU': ['PsHopAT'],
             'aBrW/VS0m6Kia/XWdoqVX4P6DC0': ['PsHopAU', 'Q48BC6#PHI:590'],
             'kJ+8W1aHoLZoczNvyXowOmeB7u8': ['PsHopAV'],
             '9PBY88dL9DbSfSB/vePnFdDw5A4': ['PsHopAW'],
             'VUZLwlDUVgVk3Z//vDalS/IlfI0': ['PsHopAX', 'Q6EES5#PHI:994'],
             '5b99pNllLKCJ67akMbWVKGcnN+k': ['PsHopAY'],
             '5t/cWPP8nhgRdEF7kc3Nzn16B3g': ['PsHopAZ'],
             'G7ZPtiaoBAMvpLF3wztoQLIES0c': ['PsHopBA'],
             'ynqOS9BDDaZ6di7x0nrF+td09pQ': ['PsHopBB'],
             'lIpXgnDsdLpwEAryf95X7xhsxK0': ['PsHopBC'],
             'qkoh8zaIvhEJUSIVWiJMlAnyi+E': ['PsHopBD'],
             'KUdZbWpmsOGARFnNVyCGBHFKFhM': ['PsHopBE'],
             'g/3KsLleU/FRI2DAzeV7NH8TAuE': ['PsHopBF'],
             'JNGLeM5BAJkhWFE/NoMwE6l/NVM': ['PsHopBG'],
             'zVL5tSy0BktYvXLO26pfaXdZH/I': ['RsAvrA'],
             'swxVz3El63Vt7BukNBX0hG4mJVY': ['RsGALA', 'Q8XYF7#PHI:5134'],
             'D6kmHwsFu8YVwEkwebeqT0FVFjU': ['RsHLK1'],
             '4HTKDeZ6PF+biy6PYCqeBs5l7RU': ['RsPopA', 'Q9RBS0#PHI:5145'],
             'N7I3R8zcHyBFWveDbDYEp0v1NvU': ['RsPopB'],
             'ZhFB533ZQ3I9QaeaG1+Wc9ziCU8': ['RsPopC'],
             'ehAUFtVH7dWrkDeNNCzUAUbhNb0': ['RsPopP',
              'Q8Y125#PHI:4969__PHI:5140__PHI:6306__PHI:6336__PHI:6364'],
             'saVSwxg93ezIM6+ACdQ3nnLqSTw': ['RsPopW',
              'Q8XVQ5#PHI:2492__PHI:5144'],
             '+z19iVKm3WlJEYif+GowrCXNfNs': ['RsRipA', 'Q8XTK9#PHI:5119'],
             'hmoZzR0hwsGtcVqhNz+HH/YDAR8': ['RsRipB'],
             'DDGGdBst2YkYVNqQkXLFlFBNVrU': ['RsRipT', 'Q8XUH6#PHI:5178'],
             'YMglSLsDc4mqJG/9Kq2yE6fTSKE': ['RsSkwp1'],
             '+APRW9YaKk+vJ3D0EfjLAz5qzKY': ['RsTAL',
              'Q8XYE3#PHI:2922__PHI:5143__PHI:5179'],
             'BqcwVE0CgkXJYTxS8qCboSsxsTU': ['RSp0304', 'Q8XT13#PHI:5130'],
             'n9lOa1bnDOwoc5kOHsmD3AB5l20': ['RSp1239', 'Q8XQI6#PHI:5169'],
             'dra8uBvDiqcRhXI4ss2M5350V9I': ['RSc3369', 'Q8XU25#PHI:5131'],
             'AxfCHEFC5d0qIX51g/tTBtUUDPI': ['RSp0572', 'Q8XSA6#PHI:5127'],
             'xjNmvaZAT2Vkos74Q8vdUC0RWC8': ['RSp0842', 'Q8XRI9#PHI:5399'],
             'tpAO+5zSZZtn3iFl1Sg3m1c0o1s': ['RSp1601'],
             'LOzaMNsMgtT/YPTssAXQZy4oHMY': ['RSp0323'],
             'Lx/Cy8JVkp+NvaPU058lCUysCFs': ['RSp1281', 'Q8XQE6#PHI:4025'],
             'Fcb/nOyD/DmjC53tY/Q7a8WhvKA': ['RRSL_00326_1'],
             'JJg/pKWDqDoxpCAVzM8E2C3E19o': ['RRSL_00326_2'],
             '90ni8otlA5L360jtW13GWb0y/10': ['XAvrBs1_1', 'XAvrBs1_2'],
             'uwglxDHhUgBmK48FInxv+Bd++Io': ['XAvrBs2_1'],
             'qf+FC241n1QI+zg+92RugsyFkeU': ['XAvrBs2_2'],
             'qlRp8ITjfJ3QxjzQqV5tfitG8OI': ['XAvrBs2_3'],
             'NFQ0dxQsOV4zN9kaG4nNVrgdiCE': ['XTAL'],
             'Tf021BnTtoAoO8G6wzqOTHpJ4OU': ['XHpaA', 'Q3BYL2#PHI:6086'],
             'i7W9+dOGlutEOFN62GXFkcmmIPI': ['XopB'],
             'a90eP9OOKMUyNqYZqWJzjiEEBUY': ['XopC1'],
             'q14HFQvJmwxFz7JUDlcyHg9kRm0': ['XopC2'],
             'pANly0Yk9aW/vGOVqmm+pyOyB7o': ['XopD'],
             'rTJyZ2xALKZ26+Zpbx0iOshKnL4': ['XopE1'],
             'IvPxToGB4hoBTjolYE5CfGPS+TE': ['XopE2'],
             '0ujcTkvu/D26kpSjnjB6RLQ58g0': ['XopF1'],
             'Y6ZEhTUON0TAH2bBl6FSna2zNSs': ['XopF2'],
             'TVOos4Dv9KFc9WMacWlOUu6DRY0': ['XopG1'],
             'pluMsMSEOL3A7RtBGeJXRHqJ6hk': ['XopG2'],
             'jhMLMmiqonBwZLCs1a3S1sXNAPg': ['XopH'],
             'A2L9DIwo0OLKtZW0aR32NAokrxM': ['XopJ1', 'Q3BTM6#PHI:2973'],
             'l/Jk6r7OKAx+xjhdl7hGKS6R2Ck': ['XopJ2', 'Q8P4H6#PHI:6275'],
             'ieFTwVa5bx7Qe8dHUqsxeyyr+dw': ['XopK1'],
             'IkBN3uWZdTRgy2N5VvHDaxzkJWY': ['XopK2'],
             '3u5kCs9vW1LByIOKmL7aLnc0Cv0': ['XopK3'],
             'cBz+QCNapsFK9N64c6vvuQ5pDGk': ['XopL1', 'Q3BQL2#PHI:2688'],
             'GLF47ov/UNa8gYWDVp/Uc8T7z3U': ['XopL2'],
             'ZEJ2dL/LgkdikgC62M8TlzxE6yU': ['XopL3'],
             'bbRdyuUXRQ1ULjpo8amoOupI20U': ['XopN1'],
             'Jtd/x+4+XplKktYm8vNuyn5S4D0': ['XopN2'],
             'Gs038raOl5HFgvN9Txs77TpMrdc': ['XopN3'],
             'chdiDZvBsMohCVtHeOobrczUsHo': ['XopO'],
             'WCi7SwwIsbMjtTR0pUcnWEWzRlU': ['XopP1'],
             'bwwZOvByZkxkMS+pb7HPist6nX8': ['XopP2'],
             'KxnwmtKip4oX1JFsImBqYbwUuAQ': ['XopP3'],
             'Qug3eECEOgv/HUOWs9Vls7DKHs8': ['XopQ1',
              'Q3BM44#PHI:4009__PHI:7127'],
             'OIxG8WAgdjXogO/5xKlVza4cZEo': ['XopQ2'],
             'P5i77wvCHPPcDCJ9LToZjWVEXbo': ['XopQ3'],
             'dVGKJ7gmWYPWie/1uUSgOGF4uqI': ['XopR1'],
             'r43zP7K762zPIu5QtfwLYuYUS3I': ['XopR2'],
             'qaxCb/Wj9oefGRmEajFJKarllls': ['XopR3'],
             'D43m+QWvGhrGyQxSBcypjjHmto0': ['XopT'],
             'WjuE/CVGMK93tRo3WtFvuMQryBU': ['XopU'],
             'xGmP7P97txNpHgkkVUIxXKmqX3E': ['XopV1'],
             'magBGGFASLIspv5VWGMev6lljfs': ['XopV2'],
             'ur+sTqUYUbP6a5x5VLvO6uYFEs4': ['XopW'],
             'sXZmW9K3xZLwsjpFc19vGAicVAI': ['XopX1'],
             'NyIXF5nqQUrHH8XpXqPMZ/Q4m8Y': ['XopX2'],
             'C4AkoDOFwoDJ+L0LN0MaPGrr63M': ['XopX3'],
             'ALOIgtTQEVleFVfB/8NNmyVXJsY': ['XopY'],
             'oAHhaQTH0fqx2/SrSBlLN7thF5w': ['XopZ1'],
             'lX1B7RFD+CUBZSiEeXtcCR4stVE': ['XopZ2'],
             'WFLP068LATfySYciNBV13unbQX4': ['XopZ3'],
             'pvDxl1cdjJ2sAnE5a+T4ZKLf3kQ': ['XopAA1'],
             'tgnFovO1OnsRYcY1cxA+O4hFYhw': ['XopAA2'],
             'HGWrYYPZMT1kNr08EyzfstAlSjs': ['XopAB'],
             'm+zLqvMvUXa+311iCl6kp6pPwlQ': ['XopAC'],
             'a0n5au112NTlRJU7ESn5JZ2uqt8': ['XopAD'],
             'HmQdGZJWGWdadGv5wXXz1xI3k+w': ['XopAE'],
             'YHUA6JLYpeR8r5NlT3QGOFYivoo': ['XopAF'],
             'LZtkW+InCJQKGMAu5PeEgmpinOk': ['XopAH', 'Q4UV56#PHI:2918'],
             'wQXqa97nO3Em6UScUnlP1bqtftg': ['XopAI'],
             'qKGe+xWT/Rh2EGZvBmhH0asEEEM': ['XopAJ'],
             '7d9bZ/NXl1L1jSSDSnl5wRrsD50': ['XopAL'],
             'R9haq2RqRk5K52cdCn9mn5NQefA': ['XopAM'],
             'nJT0TzUDyhwWcKascRVrnPy6Pf8': ['SeAvrA'],
             'kDLLmhG9JmSwHe5pCYqM7k5LcUA': ['SeGogB', 'D0ZSS1#PHI:3752'],
             '6vWnSD0BMnF0eViRr3HaQbMhbFg': ['SePipB', 'D0ZTL3#PHI:3754'],
             'fkJB9FeNbgsJHGemS/FGxtsMitk': ['SeSifA', 'D0ZV82#PHI:3756'],
             'T51GYRB8ilSWwo+glNdBEDDBNZE': ['SeSifB', 'D0ZI08#PHI:3757'],
             '67BwHZbYXX7S6BewAxEEG1pJQqc': ['SeSipA',
              'P0CL52#PHI:7224__PHI:7922'],
             'DL9mioXFiXI+Qb6PtfUnQvA84Ck': ['SeSipB',
              'D0ZV22#PHI:3734',
              'E8XL13#PHI:5110'],
             'bOLPZimRIClEkkIJ5gfG4v091/c': ['SeSipC', 'D0ZV21#PHI:3735'],
             'HVMOVDdJ6YNmMWn8wRtBpvFbkAk': ['SeSipD', 'D0ZV20#PHI:3736'],
             'VcpuNuh0sBYsHVhTPglpWjVk9Nc': ['SeSlrP'],
             'HVE7zb36Q3b9GAfYxyunpbGI89M': ['SeSopA',
              'D0ZMG9#PHI:3737',
              'Q8ZNR3#PHI:7919'],
             'UQwDH+y4MXGRiHj5Fn4PM/N6afI': ['SeSopB',
              'D0ZTL7#PHI:3738',
              'O30916#PHI:6738__PHI:7236__PHI:7920__PHI:8298'],
             '3pkMq4mWiCQNAPOSkB1U5SRT83o': ['SeSopD', 'D0ZVN8#PHI:3739'],
             '4zg9vu4xTwQdifNwTgAd4RJd7X4': ['SeSopE',
              'A0A0T9VTM6#PHI:8678',
              'D0ZK57#PHI:3740',
              'Q7CQD4#PHI:6800__PHI:7921'],
             'ekMNGbN0IHIT6G0pokSRUtX5GdI': ['SeSpiC',
              'D0ZWR8#PHI:2626__PHI:3759',
              'P0CZ04#PHI:555'],
             '19HXLRiZWVMrCUCAFRkA0o0+7LQ': ['SeSptP',
              'A0A0F6B5U6#PHI:6798',
              'D0ZV15#PHI:3746'],
             'j9jQvUg9ll8oy/NYBQkbRHO6f4A': ['SeSpvB',
              'D0ZHS9#PHI:3760',
              'H9L477#PHI:663'],
             'MezetuISvipnkk4GUAaruDCjolg': ['SeSpvC',
              'D0ZHS8#PHI:3744',
              'P0A2N0#PHI:4517'],
             'z/dRlfvd+RvtrXbPxKwY+K4CHHc': ['SeSseF',
              'A0A0H3NG92#PHI:8024',
              'D0ZWT0#PHI:3764'],
             'Ewj51Zj/ZyWOzFMcnBJT0aX8sh8': ['SeSseG',
              'A0A0H3NB75#PHI:8025',
              'D0ZWT1#PHI:3765'],
             'qOkcZuAplcJ5/2O8XI5fh1JfzP4': ['SeSseI'],
             '6GFdi+/W/KwPYpF9gMheJlErYXY': ['SeSseJ', 'D0ZI42#PHI:3767'],
             '7FNkQ4owBQW+ZsKDJj59uG5DUTI': ['SeSseK1', 'D0ZQW0#PHI:3747'],
             'p5NKW0AINrcuRRQoJotoJqdKoA8': ['SeSspH1', 'D0ZVG2#PHI:3748'],
             'wwJ8vb7Nv9a+S19tFG2KUZ5rCdo': ['SeSteA', 'D0ZXR5#PHI:3749'],
             'bx4DewI3a0ZEA9vcJpWwy6cHUUI': ['SeSteB', 'D0ZI38#PHI:3750'],
             'LTCZ0GJcooxTql4Ae6zYn4jouoY': ['SeSteC', 'D0ZIB5#PHI:3772'],
             '4uX6OV/eHmUnzbxTCfagJCPkGBE': ['KEQ67658.1'],
             'RxaZ5VHoFU/U/l7TgMs/GBRDQBs': ['XP_013431971.1'],
             'K5SSCOD9BrUC3rANUT4ruAsBCAg': ['KEQ81621.1'],
             'M0n7L19OFJJ9rVuWWH1IRDVIs1I': ['XP_013344128.1'],
             'EG6LxRYbeJmvaXz8FburXScF+wk': ['XP_007678837.1'],
             'XM4fzNRsRsk2I5gCOw6UndUvjVc': ['XP_014077357.1'],
             'cTlIEBrAG3QD+W44R5JZBeUp+KI': ['AKM21218.1'],
             'GfsevG+hIo5yeXeVgOYBXYF4MQ4': ['XP_007686215.1'],
             'e3Hhgqzn+kVIgl01hL/KjT38vm0': ['EUC44184.1'],
             '9kvL86LNtXCjaq7iG8gsB75Oacg': ['XP_007699156.1'],
             'pKFS2yIDdbZAX1WuTaYjHkcP6Mg': ['EUN25607.1'],
             'vBvazmJVJ6Ze82YbwOmL+NFFFB0': ['EUC36307.1'],
             'iNQm5ClI2jSl2BDt3cvsPaFfElM': ['BghAvr_a1'],
             'odxgtappDixkIJyrWE1ObJI7Pi0': ['bgh01362_CSEP0027'],
             'L5gVZOiFqdLKr0jDId88eyvxBrA': ['bghG001947000001001_CSEP0340'],
             '/FCOwIDWvs9UA4bXr60QgBlp+S0': ['bgh01363_CSEP0028'],
             'TdBb4yw2VSA8G5jhkIje3GF6lvU': ['BghCSEP0055'],
             'GIMQXavJMR757xT3wsQ3cIxd5g4': ['bghG002872000001001_CSEP0374'],
             '0hjMOriWE49ZpFAKhX05h97DVUo': ['bghG002857000001001_CSEP0371'],
             '1KuwJnCP7GvPdHUURmdXaFYsfIA': ['BghAvr_a13'],
             'EtzN4OuPcro8cAJ2OwMS3yO5H0I': ['BghBEC4'],
             'Stq6nzuS+pJCWVaYW9x1oe5KL8U': ['BghBEC3'],
             'p5QgnYRWiNpWBGD6tv3hY462sLM': ['BghBEC2'],
             'JovZxpJe3eJBHv7vuzX/8gDZPHI': ['bgh06532_CSEP0264_BEC1011',
              'bghG008575000001001_CSEP0486',
              'N1JJX4#PHI:2898'],
             '2Xn+VWU07p5Xhnl/ZjqXr8WLFBw': ['bgh02877_CSEP0066'],
             'bTxAN7yHYnhp7dcua0qQalbb3rs': ['BghBEC1054',
              'N1JJ94#PHI:2903__PHI:8918'],
             'DIILE2duHFPDatBYH7TzPSoC7Nc': ['bgh02875_CSEP0065'],
             'C6TRC1SIxXcyVe45YidT40047rg': ['bgh03782_CSEP0152'],
             'plp9wtTu48mnBLDF4S7sC/L2jPg': ['BgtAVRa10', 'Q09QS3#PHI:758'],
             'PAS6LewVDuTttn1aJslj+4eT4Qc': ['BgtAVRk1', 'Q09QS2#PHI:759'],
             'L15GhHgGAaW4P3iH4Zv8xfnGB+U': ['BgtE_20025'],
             'P0qzCzfU59fHN8FzeZeoJYdZCxo': ['BgtE_20090'],
             't5EAqGYHAA7N3xQW1HsaBv/KyBQ': ['BgtSvrPm3a1f1'],
             'spdWUu3qzZXWL8kbICEsLD9r9s0': ['BgtAcSP_30091'],
             'unBJkROTJF/8bVRDLSwZ3gcPTCc': ['BgtE_5901'],
             'igQgAxLN4t6M3ougye2Fk3mtJkg': ['BgtE_20000'],
             'bfLc20ihmMKZnEU3Vr4h2k3AurI': ['BgtE_10117'],
             'otp2P9LhFVsfiJsQl9+TFKkE648': ['BgtE_20026'],
             'rbvfaKwwwIrsHXNq3FKT09XzZvg': ['BgtE_5664'],
             '3MkOSaD5jXME1al/NtVqlnuczWk': ['BgtE_5665'],
             'IOhHdv+SLGn39TT4vdmb2mWZ4eI': ['BgtE_5843'],
             'uCwHGKIH1lv2K3QTg7dijQt1vAY': ['BgtE_5842'],
             'inFuscow1s3uWYDnbEx17aOgclw': ['BgtAvrPm2'],
             'IT3HoX8InFDLccdhlkiEd6zZexE': ['BgtE_5846'],
             'xlLgSWAUo2dz407jS1EsvZ/rJCE': ['BgtAvrPm3a2f2'],
             'lHF45xnvC0hndh8jWXeenezROpA': ['BgtE_10124'],
             'ZW4KJZIL/fmUZj+z+vjWyEEsUa8': ['BghCSEP0105', 'N1J904#PHI:4622'],
             'uccMRbOIZLa9ctZWMDZkjOCHGsw': ['BghCSEP0162', 'N1JPG4#PHI:4623'],
             'GYHAlrGwesvYbR/lJTmzC6qVzYs': ['BghROPIP1'],
             'isJw1RdL5PI/z3eg+EcZ18cr6Cc': ['BfNep1'],
             'NemO+IqXouCzy1JbBpGg7bGNdGY': ['OCK93132.1'],
             'c7UeuM2oUoRAMj5kGKwugu1jgt8': ['CCE26798.1'],
             '13a341avohnojBGkD4i4Zri9exM': ['CCE26799.1'],
             'OwIrOnEM4phvGftZpl4hDMudGcI': ['ChToxA', 'M2URZ2#PHI:4924'],
             'zggldvd8RSiUBXytvH3V6ximW04': ['EXF72942.1'],
             'A++EN+B6jwHKpcdJafkLMR4bBgQ': ['CgEP1'],
             'beDjSdSq2uNaXj6vka2GygX86X0': ['Cgfl'],
             'jWj7sSCnL9gooqhCTSzhg61JyGQ': ['CgDN3', 'O43115#PHI:164'],
             'zeM1esmEzuCSmxKQycR3I6Ldxkw': ['ChEC13'],
             'hzsppOKmhZhTCFPhz7QCxCke5aE': ['CoNIS1'],
             'rWGpiLT/MG1afRXjgBH60iuTe1I': ['CoDN3'],
             '2KEuuV8zXYxxG661Q0Fu/8ZqUcc': ['EME45057.1'],
             'rQ7OfkxMKbCTw53QxOxxT7O0EbM': ['FoeNEP1'],
             '1ZOUPiu2+URiarWFn8/aVgrcWIU': ['FolSix1', 'Q709D8#PHI:5284'],
             'tCGQNPAXGowzC0cwFB0vEw2mEAA': ['FolSix14'],
             'avjqBowy1yXYcsyzw54FU5m04Jc': ['FolSix6'],
             'nQ8W3PlXvQSs4kZQenvLS+QWFFg': ['FolSix9'],
             'OLaXRoPHJo9j+XMmyhVhZwL1DJk': ['FolSix10'],
             'Z2MP0YiuumgZz5tPes5jOG5W59k': ['FolSix2', 'Q709E0#PHI:5285'],
             'Hi6JyEBkwcik56hcTjmi0nz143I': ['FolSix12'],
             '02OViOG+C1avDyNRzMpWSlV9NX0': ['FolSix11'],
             'oEKq3wjjp9ovRqFq00hxFX/5YmQ': ['FolSix4'],
             'ZCCkg2UIiSyTqM5NjVWnNwDCufU': ['FolSix8'],
             'CJQoqVQq/gV0fIJ5YdckF7odJjo': ['FolSix7'],
             'ZWM872RPrz9/ffOBTrVmIT5Jvg0': ['FolSix5',
              'C0LT61#PHI:4849__PHI:5286'],
             'lyyBFrcFCuL0B2RYbYW0QENR87Q': ['FolSix3',
              'Q2A0P1#PHI:4848__PHI:5283__PHI:7472'],
             'JkN8Oe4rOQW3MlyVeEnDFoJ0p0E': ['FolSix13'],
             'gxjPUjKyD5IjrIVihTuwO5NyB2M': ['Fom_2',
              'FOMG_19011',
              'W9ZT25#PHI:5067'],
             'OhBPuAXGXijdJk1XE2FMAN0JAkk': ['FGL1',
              'Q6WER3#PHI:432__PHI:4212__PHI:4242'],
             'GkWBMgyRqBd+V8oygq8jjDMFfto': ['GiSP7'],
             'ToX+2vG5VPH8PJAvcW03SXFdzdY': ['GoEC2'],
             'bWAFWAqvLQ9c+6zXv7zm8wlx86Q': ['MiSSP7_2'],
             'xBrlkawDLXxY57Uh4uCAJN6YKkE': ['LmAvrLm4_7',
              'B9ZUL0#PHI:1132__PHI:2276__PHI:5371'],
             'lsZN6G8rkTionQ6ykVOmHTIzf+0': ['LmAvrLm11'],
             'bKoDax9m8V+OGcOe9zSK2yLm6qQ': ['LmAvrLm6',
              'A4F4L2#PHI:1131__PHI:5402'],
             'rWUGaj8cIFIX9fmPK6brr+il+bE': ['LmAvrLm1',
              'Q258K5#PHI:25__PHI:2281'],
             'o747VeUyY+OXjxorYyM6LM5hiTI': ['LmAvrLmJ1'],
             'rSHzHh0yrKksaLu4hI7OEIpEIHE': ['LmAvrLm2',
              'A0A0A0S3X0#PHI:5401'],
             'oVJavUt9GYo5C1MxysEj2tEr0/o': ['LmAvrLm3'],
             'wf6r/aUMyGvG4fLgaozaw6EnXFs': ['MlpCTP1'],
             '1gwCophTVwvFhqnQSOL1lKeEKL8': ['EKG15312.1'],
             'ppZsnwSfaLWLjmhbsm7le622/tk': ['M.BR29.EuGene_00091361'],
             'KJ4tvPCQHfRsOpzHsVSzAvF9zNg': ['M.BR29.EuGene_00106461'],
             '5xWUEb8CVXH6be3JDJCH2drmB14': ['M.BR29.EuGene_00095641'],
             'Lh181Yi7w7PMML8d+b10cTyrq24': ['M.BR29.EuGene_00085071'],
             'NhK+taLKhoAHrm1a7VaZzVuI3DU': ['M.BR29.EuGene_00043011'],
             'TBsts9TAFnfLk4twL9tz0NTzrrY': ['M.BR29.EuGene_00119511'],
             'g4ynz2YeHoxcxFUJ743XpsHmhRU': ['M.BR29.EuGene_00107481'],
             'xD7G46JcPSlOwsARhD59u9YY/Oo': ['M.BR29.EuGene_00119491'],
             'Nu6WuS4GiB0grhvU/YbYBPipD0I': ['M.BR29.EuGene_00082031'],
             'a+lhzhQ2LW4tOPhzQPMvwcvHQvw': ['M.BR29.EuGene_00091681'],
             'wlCPEIaeuu4+RVXi3dTD+BUkPIU': ['M.BR29.EuGene_00112111'],
             '7T7CG81OxkisIqSBiYNZyJJhU3o': ['M.BR29.EuGene_00113041'],
             'eGwF8DnRJ+TIf3fyegObLyyO/Ww': ['M.BR29.EuGene_00125811'],
             'fAKdwE25qv1Ztr+Le8um98754Ig': ['M.BR29.EuGene_00126081'],
             'VImA/1tYs5uT6XTEzT2d6Uf8kWA': ['M.BR29.EuGene_00118801'],
             'L5q38x1te+pLTxym/N3hsmh3UuA': ['M.BR29.EuGene_00081821'],
             'wnJwMDNwWagAP8FC3iB14bVI7g8': ['M.BR29.EuGene_00041131'],
             'meVLPk3vX/2G13bMD+0YNE30dTc': ['M.BR29.EuGene_00088411'],
             'ZgqzIBVJHt5lHR+YKr11DOpxn8E': ['M.BR29.EuGene_00060181'],
             'f6N6fexl++j9Va+JplYDEs9oGak': ['M.BR29.EuGene_00121691'],
             'nMyp2BHxQo1h+nVfF0rDpGKqyh4': ['M.BR29.EuGene_00004921'],
             'kk0Jk1kO0ZS4IsYmkRuD8cvZ00w': ['M.BR29.EuGene_00087671'],
             'tw5jp8T3Uxl/YMfA4yIpiUQZJGg': ['MoBas107'],
             'wxQzhr4oUN+wVjZ2lzMa5dyFcoQ': ['MoAVR_Pia', 'R9RX08#PHI:6343'],
             'JQat5ew+p0r+oGAeEvEh0syAIck': ['MoBas1'],
             '40VeoZTLdW32iQa6G9Dumf8JAAE': ['MoMC69', 'G5EI17#PHI:3122'],
             'UrGZt+hIjQhh+X9HxYuhrrzpyt4': ['MoPwl2',
              'Q01144#PHI:42__PHI:2152'],
             'yD0gXeDFO3GiRqh3r+nk3GMow9U': ['MoPwl1', 'B9A9V1#PHI:2151'],
             'Rp5YBl+DHFXcF5a+KwF7vWBd2QI': ['MoPwl4'],
             'sMhf8WiBxNufN0z9B0kabpQ12FU': ['MoPwl3'],
             'lFrLr85UJ7mVU5lxbBcP9NauHeY': ['MoAvr1_CO39'],
             'SuXkotDIWwnfu12QyCFGXIZmpj4': ['MoAVR_Pita2'],
             'FHxjcRgWQf4QQX1oMLVrYW0l64k': ['MoAVR_Pita', 'C1KJG7#PHI:2830'],
             'jfX36mjiUhZb0X4FtwtPFJIamGA': ['MoSlp1', 'G4N906#PHI:2404'],
             '8cthQba6WFS7mmHoIsFLqFI/jbY': ['MoAvrPi9', 'G4NJP7#PHI:4526'],
             '+rk8BXfCYhPiwmy5YnyQJxUEUj8': ['MoAvrPiz_t',
              'C6ZEZ6#PHI:2064__PHI:7896__PHI:8697'],
             'RzECtctzaAHjAXgpIV+48JR7XNc': ['MoBas3'],
             'oqtPiDEb4J5zxbNTNJt4/SfcRLE': ['MoBas4'],
             'jsaliP8mivkoNSsK2hpgCB7BGcs': ['MoAVR_Pii',
              'C4B8B7#PHI:2136__PHI:4979__PHI:6500'],
             'DMqzFpvUE3GgE8wPk0qBirNqAUo': ['MoAVR_Pik',
              'C4B8B8#PHI:3104__PHI:5553'],
             'KvvVjKRBfSkZHg8PQoyDsIzwF5w': ['MoBas2'],
             'uC+m9gQ83oLc4OsiotlcF0ukrbQ': ['MoAvrPib'],
             'jxLJVdEuEAWUBO27ew4bL8I4Xig': ['MoIug9', 'A0A0A7DM22#PHI:4721'],
             'tko6AVvSMr8lbmtQ/ekf5OAbOlU': ['MoIug6', 'A0A0A7DLN4#PHI:4720'],
             'blHokzxDZwGgBgULqkSCNyl6k0k': ['MoMSP1',
              'G4MKI0#PHI:2118__PHI:5540'],
             'cyO6bi/JblRzOo8pZq1v1NwDOQo': ['MoHEG13'],
             'TbL4StNBk+8jTgLb/ulJ2Fve278': ['MoCDIP1', 'G4N8Y3#PHI:3213'],
             'i+Bl3Hhs2YYgL7OS49QaXoYZac4': ['MoCDIP2', 'G4MML4#PHI:3214'],
             'GtMXQ++M1mLXXGTO/uhj1O0KLM8': ['MoCDIP3', 'G4MX34#PHI:3215'],
             'sXSkEWt7ZQZTxPK14fyC8k6AmLY': ['MoCDIP4', 'G4MVX4#PHI:3216'],
             'HGohLt4nDRHkKp3Sf2HXHWAwK+k': ['MoCDIP5'],
             'FHnWDHjYLrqRCAHTD8Usz3Kehx8': ['MoSPD2'],
             '+0dIAF680t3GTwR3X2xD2wKMrq8': ['MoSPD4'],
             'gUzoiQi1yNwn1I48MmETi454Yw4': ['MoSPD5'],
             'hbA/VPNWPZPI+6jgxFTIGZnd0bc': ['MoSPD7'],
             'G8OiBA9a9fe1zrcwQ1taNIcEvE4': ['MoBAS162'],
             'YM4wZ2vNGGVI0o8auSjnDFBIcCw': ['MoSPD9'],
             'hd5bwqTA/FVHVkoXRE/UKYObUN0': ['MoSPD10'],
             'L89/7X9qyqqxTyf9xQ+N7ppYl2c': ['MGG_16058'],
             '8ZrJxz81mqQkD+yb5xUewkbhkXU': ['MGG_04384'],
             'yr/s/xZfqaT9NaD3XGHH4T3/7ew': ['MGG_14793'],
             'dfpl54t0rWQfeZEpahmJkR+CZRE': ['MGG_15207'],
             'U5QeIMFStabZ/PUsWNmK5TtI7DI': ['MGG_08944'],
             '6np+XmREg05oXAxv9ahUPIhEqbA': ['MGG_15459'],
             'xes8qAE8tMepjyTYelK81J35/O4': ['MGG_10120'],
             'Efb7uQ8HN+2WN3PoqsXQD7+iyWs': ['MGG_18019'],
             'Mp09K0EJbKbXRhsTas5zCy4ix/o': ['MGG_17132'],
             'tB3ViDaAlQ+2FB6w75O0l5B2K9s': ['MGG_16619'],
             'KQSc7FrkKm+nKv2PgFASoECAyyw': ['MGG_16113'],
             'MoM5IJJHhFEFkC9UPaQ7zpJu9e0': ['MGG_14834'],
             'yHE3HEYPaEaN34/+u69VCThFoUI': ['MGG_08482'],
             'PRJ5ocoLaroSmtZVl+Sc7i9Lb2w': ['MGG_17255'],
             'T/yNxRY9FLFry+AB61d3482dpAE': ['MGG_00821'],
             '/gpfOsMnSCYrVL2h8SXVLPY/ZJA': ['MGG_18060'],
             'EazUqsdy6JRuE+CQajHRff2CUDE': ['MGG_16175'],
             'uggJ3nOJfiG4Om7YLekwyWiPc38': ['M.TH16.EuGene_00000541'],
             'RcmgwXwvxTgmfahqZ7yuNKCVuHg': ['M.TH16.EuGene_00106621'],
             'hN2rrzQLOTrAvGlfcMcZBuFTt2s': ['M.TH16.EuGene_00134971'],
             '/l6Dg2QOYbwp5wz2eckEelCOTQg': ['M.TH16.EuGene_00034081'],
             'myMsKTAxYeFl2aHudjlyUODERJc': ['M.TH16.EuGene_00045871'],
             'urB/2yy0OYOAh6XYukEHyYPurZo': ['M.TH16.EuGene_00124981'],
             'XtC/TFUR55nP1khmBSU4J1gadgM': ['M.TH16.EuGene_00135161'],
             'BBLwFC3DGTljz+qlCbK859d8wVY': ['M.TH16.EuGene_00079081'],
             'LUyIAxiHKOFjkHmwQZimzcR/YCg': ['M.TH16.EuGene_00101881'],
             'OPsWTGuXRm4pqir1iaKv7xlh2HE': ['M.TH16.EuGene_00099371'],
             'S/tiZYwsCubpD01C6t5Yd0UXDvI': ['M.TH16.EuGene_00079311'],
             '6azEx4UsdXqEnossKI2MQSkVV98': ['M.TH16.EuGene_00127871'],
             '/rsxXG3g5ickPaMrDeNOzju6gZ0': ['M.TH16.EuGene_00027191'],
             'PQrxNe09wWCB6diwhvHTqu8YEiQ': ['M.TH16.EuGene_00027411'],
             'iatY9OwqBj11QFjVAeeOD0x3JGI': ['M.TH16.EuGene_00040131'],
             'YYECRxF7iZdP0wqWftBBwdnOcGo': ['M.TH16.EuGene_00120731'],
             'uJhjpJHI2ACtpzkAWU8K2jQTsPo': ['MlAvrL567_D'],
             'RI4cuHfuIwbDFc9WO2Y9L2StMT8': ['MlAvrL567_C'],
             'M+VQWHhgWhPZWe1Td+E6cMLoXJo': ['MlAvrL567_B', 'Q6R659#PHI:533'],
             'nlFttyxrh9fzrqyqPX/BNwMsMVQ': ['MlAvrL567_A', 'Q6R661#PHI:532'],
             'EFlNYvDYqebh8QM6STNdhQxQTNM': ['MlAvrP123', 'Q2MV43#PHI:537'],
             'V+BuU0AZzPIIpKcTeYiQeI/yfec': ['MlAvrP4', 'Q2MV49#PHI:536'],
             'H7IOYJlcOs9KmIL1Hir2gzEx9Wc': ['MlAvrM', 'Q2MV46#PHI:535'],
             'Na7WYuonVzW5Lt5ATylKQCqdTq0': ['MIAvrM14'],
             'YvNW8n3mMXDuP7an1TqpEEjfv9Y': ['MlAvrL2'],
             'eWOMRrePLwpH82GgZJVZFpF+8iE': ['MpNEP1_1'],
             'QhSJTXAT+XkqTww3hm8yyQ2U2LU': ['MpNEP1_2', 'A5H7H0#PHI:3128'],
             'Tw4f3sCoQSCgPTU0/Gys/ZY2KX4': ['XP_007580207.1'],
             'Lgb7bzbwimdpD3XroxgRqJDaxqQ': ['XP_018034946.1'],
             'XbqnTubpbi6bzz0celAL6mDOWws': ['XP_001800520.1'],
             '9wvnp65/PY5PLuniAewrDMkdBsk': ['CfEcp1', 'Q00364#PHI:70'],
             'DXkyCgQ7BE4c71V8Pe8VqjeHMOg': ['CfTom1'],
             'Vs5yvDSVw5y39/9eMkZPnEj2zWo': ['CfAVR4',
              'Q00363#PHI:18__PHI:5476__PHI:5487__PHI:5546__PHI:5556__PHI:5561__PHI:5567__PHI:5584'],
             'LqPopBLf6BdTlGIMf80Hgc5mwOA': ['CfAVR2',
              'Q8NID8#PHI:472__PHI:2344__PHI:5496__PHI:5541__PHI:5555__PHI:6285'],
             'ZTMd59CB6hzs7JRB7e79rwxFbko': ['CfAVR9',
              'P22287#PHI:7__PHI:2845__PHI:5473__PHI:5497__PHI:5542__PHI:5545__PHI:5558__PHI:5566'],
             'rwsdWa1KgUioeSie2fBh9jRYpdw': ['CfAvr5',
              'A0A023UJQ9#PHI:5538__PHI:5557'],
             'FrwnlxXAQWAG2RBMIos9q8Me43I': ['CfEcp2',
              'Q00365#PHI:71__PHI:5486__PHI:5544__PHI:5559'],
             '6EuOKsN/DH+OHjt3ojFlG5UEPnY': ['CfEcp5'],
             '7AQYdthBw19C5bR1TiVaQFmTCbk': ['CfAVR4E', 'Q5VB04#PHI:529'],
             'UTpNPziaj0/suUAFYlq/AGXI1Oo': ['CfEcp4'],
             'WT+QG6jXfzxmgkcq5q5E/nyVTm8': ['CfEcp6',
              'B3VBK9#PHI:5495__PHI:5543__PHI:5576'],
             'Km0QD/mXWeQH8l7wM4JyC0Z1OXw': ['PnTox3', 'C5IAW5#PHI:2264'],
             'wMl2QeklJX5Uqy5IKrdT8hSL6uc': ['PnTox1'],
             'V4v8oiiVnK/lfPD+CJqDOT7LmGs': ['PnToxA', 'Q0TVA4#PHI:1081'],
             'autERIIsuWa2MA0s5QTKSfXoVBE': ['KXT12958.1'],
             'b6LA37gbG860JExKHjhGieuBItM': ['PGTAUSPE_10_1'],
             'j1IlHx3ptI6Gx+utk9MSwudByTo': ['PgtAvrSr50'],
             '3AgHX3GB/PrUAzoxKlMkUzOW59o': ['PgtAvrSr35'],
             '4aMkFP6TGw6vabPKp+4ZHgnz36s': ['OAL46779.1'],
             'G0fGD1r4gWCZajyvvqznNr6Boik': ['XP003298007.1'],
             'EgzxKe8j9aULYvuiFQ023W6vnAY': ['PtrToxB'],
             'tPFeRim1FAE8n1/GBGxnVAFBb9Q': ['PtrToxA',
              'P78737#PHI:534__PHI:8380'],
             'tFS2gAKGFkXwbLLiqp4BUgIQUwc': ['XP_001935864.1'],
             '1GHfwrmwBtxNkTG8jkk8xW/mA7Q': ['RsNIP1',
              'Q02039#PHI:38__PHI:2698'],
             'vilbjleF8BiF7FgLo6ef5vj6c4w': ['RsNIP2', 'I1VGT7#PHI:2696'],
             'kl9MHURlGaA0U/nm1yzOByVtbQs': ['RsNIP3', 'I1VGT8#PHI:2697'],
             '2AtrfWfk4ecT3/d0d4kRdHjSA5A': ['SsSSVP1'],
             'h34K5sfGTQ3ZWUnDBZMkmQV/67Q': ['SsSSITL', 'A7F952#PHI:4482'],
             'zUmZULm5eyMyL4AdKkP0UeLBDr8': ['XP_008025738.1'],
             'NYsJjCeYBhx35rExUCoJaTfrzrU': ['XP_016761200.1'],
             'qJcRLDAIj0qaeIGyNpdx84dU9cY': ['KNG46663.1'],
             '5JyewSxmerJVzAiqApT/KRYCAHw': ['UfRTP1'],
             'ThXX+gScAfg1IF4eRfbGg/uADaA': ['UhAvr1'],
             '29p94f2g3qcvOI355EUSp5Ea3uY': ['UmCmu1',
              'A0A0D1DWQ2#PHI:8816',
              'Q4P2D2#PHI:2234'],
             'sRCrmm6ekGv3grZ3oerEmnWVY5M': ['UmPit2'],
             'AaVXlH0GeED7+C7eZm0+6RsgRQs': ['UmSee1'],
             'bmXI3IoY+pc6ogC+QWRrIDJMnQM': ['UmPep1', 'G0X7E8#PHI:3223'],
             'MYpXMpO/yc+OBr+mZHxEcCrHJDM': ['UmTin2'],
             'Upo4AE19kKlhreOXJZt/ak2TwqY': ['Umeff1-1'],
             'Vb7iBuW95hVcHKmZ6tSe8CjjkaQ': ['ALVi.1389.16'],
             '4EV/j4jgzZT0ab8rk8Lk+V65Dbw': ['ALVi.1389.4'],
             '3phGnGjwwV/c5xKxKkh7UENLvkA': ['ALVi.1389.1'],
             'g1korrWudsYRmvWl82bPMt+8ioY': ['ALVi.1389.8'],
             'PmnEbUeoF40Zggn9nCIMcWtnDto': ['ALVi.1389.15'],
             'xWgrzEt31gM0NTIudLJfDTvqr98': ['ALVi.1389.9'],
             'pmSe02pKkV1mWwjVVan6Z69HeLc': ['ALVi.1389.12'],
             'RM+8FcV5QKNtR5otVUgZmngl98Y': ['ALVi.1389.7'],
             'AaQbQktrCkswfIZZM1iyKn19MGE': ['ALVi.1389.11'],
             'zUPSdFOCv/3+hDc0j8IvM2G6TTc': ['ALVi.1389.3'],
             'cx2MwJK4Ly5+oK63G+JUj9hxZyQ': ['ALVi.1389.2'],
             '+OyldtEcbxSlAgpPGN9kRnlZ5Tg': ['ALVi.1389.6'],
             'vRJxxsy+JK53NfBXgli942DgS/8': ['ALVi.1389.10'],
             'o67o4kqCJcMol1R88+ikZflcfzM': ['ALVi.1389.14'],
             'iLrAb0Qj+pR3hVMxA8FtejIp+78': ['ALVi.1389.5', 'ALVi.Vi1.5'],
             'xopL6+trv+jo0ZrqXfU9+o4+F6o': ['ALVi.1389.13'],
             'KaH3EV9T3lGBHEI2qyHpZHCBnvA': ['ALVi.Vi1.12'],
             'WKaA4GOR5ygPkrQf325CQCIuNLY': ['ALVi.Vi1.15'],
             'U+kk0A0TcWH8hZLOfNyxJMP2ZVk': ['ALVi.Vi1.7'],
             'Dpw7SWBeWnFtxT1KRgBznNeId2c': ['ALVi.Vi1.17'],
             'mlRa+sEx5MWznJ4R/u+Z/Yq1aaQ': ['ALVi.Vi1.16'],
             'iA8+/DGHIyFUXupAHbYGumGIY50': ['ALVi.Vi1.18'],
             'uzenFNeJcBV78rZ/YHG9pVuU5Ok': ['ALVi.Vi1.11'],
             'kYTgSVW+++RLVL9VRKVvcF0zb2w': ['ALVi.Vi1.14'],
             'JCRZmhV4A4wnT0Nvw+7M409NGOo': ['ALVi.Vi1.8'],
             '7QrV/4XNCepvVGz81W3sBlnzmzo': ['ALVi.Vi1.4'],
             'XNro+UOYbg1VLuOWxFlI3g04xMM': ['ALVi.Vi1.1'],
             'dfRhC/6LpQgw8ygtFgpoFNq7eIM': ['ALVi.Vi1.22'],
             'Ihjh4s8fPkeWpI2iOkJXeZCXPuw': ['ALVi.Vi1.2'],
             'SQtNuciV+cEWGVpDIRG/taXfX1w': ['ALVi.Vi1.24'],
             '7QUVrR7zrjJq+brE2gyiHlveh2A': ['ALVi.Vi1.19'],
             'URumGcBYt8jdTm5fvQNWVnZbfsw': ['ALVi.Vi1.3'],
             'm1KhKX3ZLMIXg8DOuAR/EJOaLpQ': ['ALVi.Vi1.13'],
             '2ZiStrWRErlIGv/TOoaMUemWHGM': ['ALVi.Vi1.23'],
             'nJzeM013uDwwxqcHY+CmdLYtJtk': ['ALVi.Vi1.6'],
             'qQpfNQ+C87t47zy9LAKB9ACyZpU': ['ALVi.Vi1.21'],
             'iIqE8f0lpZM5ARXtaAoJRmN8TK4': ['ALVi.Vi1.10'],
             'Q2i09ZWI2q2WM/OBc12jCjEv9OI': ['ALVi.Vi1.9'],
             'qDBTN7n22QdFdtN4U+eOPuN6RR4': ['ALVi.Vi1.20'],
             'GPlwLGVEtSsmhla1PhlCiF/CqmA': ['ALVp_11032.11'],
             'adktYoWGtrFvrz3xAwahJVXDx1o': ['ALVp_11032.8'],
             '3WKLoBzulEqHnDzZh9tC14APff4': ['ALVp_11032.5'],
             'NOxdk/KX4uGizDRDqGNrnphHk+E': ['ALVp_11032.16'],
             'lf04/WA2/UQBIp0OR2jYbOenlCE': ['ALVp_11032.3'],
             '4g0UeQTjawtdKs791yHsAUuZioM': ['ALVp_11032.6'],
             '+jMv8KsERadZFMT/IXZBeLAK5CA': ['ALVp_11032.14'],
             'rFusyvCCguop1Oo5fo5Yz54MiF0': ['ALVp_11032.1'],
             'iA4ZIN2Pcx0ASnM0IHZm8wtZZIw': ['ALVp_11032.7'],
             '1OKnhP+tocJ9oct+qqgUmyj7PAc': ['ALVp_11032.2'],
             'B011bEhSFhEuM97cZKlWFm/QopI': ['ALVp_11032.4'],
             'OVongh5zMMNBHGsSFo/iEPC6Pkw': ['ALVp_11032.9'],
             '3rDYLHKxUoL1fIXw3EBoPCOLEVc': ['ALVp_11032.15'],
             'KwCIqZ37d/po1P52lHtjoa34HDc': ['ALVp_11032.13'],
             'ob9UNj6kZaqVjgA/WX9Ht+YARiE': ['ALVp_11032.12'],
             'pDuy6HvlitLbUCl4cR5dXMXdpho': ['ALVp_11032.10'],
             'UK0rg6ieBdPjWTRiI/zAK8w7dMk': ['VdAve1',
              'H9DUR1#PHI:2331__PHI:7988'],
             '47Ie4Kcfxa3PNv0kqi80Ld9MyrY': ['VdNEP'],
             'pSliZ+MtadUvTOEPhBEPqFxIw0I': ['Vdlsc1'],
             'uZ4KIafbNcFYGqrUoZ07wo9ybSs': ['VdSCP7', 'G2X9W2#PHI:7129'],
             'uj5GBMFVSlyxQXm1OesmtC61TG8': ['VdPevD1', 'G2WWV6#PHI:8520'],
             '2HonVjPIee4tjgiNdJ1D1N4ReA8': ['KJY01809.1'],
             'oVus/O2uOHb4BR8L8dgEAkvWnYI': ['Zt103264', 'F9X3Q1#PHI:6547'],
             'DQRABPIHZksuOV6IdahdebW1D30': ['Zt80707'],
             '5UFQ8gA4D9hqYf6yzis599PRswo': ['MgxLysM'],
             '+dF9II17BZx0oyGKK9UlvZlaFRk': ['Mg1LysM', 'F9XHX3#PHI:6495'],
             '/GJFNhxtoiQW4nWdssTa8hArnmc': ['Mg3LysM', 'F9XMT4#PHI:6494'],
             'UzuEyLUnaTjUFGydacq3C0GM4yQ': ['Mycgr3G38105'],
             'qkm8Twq6z/DWRJdzli+Ik7WOw20': ['ZtAvrStb6'],
             '696gZIbSiYQN88hov4tbctktifE': ['Zt89160'],
             'Mxz8DV7c8rnB1OtTpqkT8+Zj6VI': ['PpCSEP-07'],
             'Vj6/vB224YYqqMxLLVng/MkbLcM': ['PpCSEP-09'],
             '8slP4wRoaYGz7PNrYn/wnlhSpcU': ['SrSAD1'],
             'BEV7JenMfQSwmMLcQtm12E+rcjo': ['RiSIS1'],
             'ppxi8POFxOgGShuj+4UUz9Zu81g': ['RiCRN1'],
             '4njWt0pg7yitr2vzbXAHam78rHE': ['PIIN_08944'],
             'YleYqChbcvV+gvXOi53SKTwdjfs': ['PiFGB1'],
             'wzZYUgrQgnFmuCfAQkQxKW6BBws': ['PsShr1'],
             'QBF1YKwE4CEi3hObIhcsZfdiJ0g': ['PsShr2'],
             'QIzhvCohMO6jckKYCNCILqgsIXo': ['PsShr3'],
             '3UWGTw9v4jeGDMB30MxZoAS751I': ['PsShr4'],
             'KgYYHcdIyp4iUbypZKtOjLH7bdE': ['PsShr5'],
             'elzKQUZHQuUKyx6xkreZ0NbLxgc': ['PsShr6'],
             'rV0bhZEFSgisKVJWupLo0p9IV5s': ['PsShr7'],
             'i12H2TJEKpeclz8i/mPIbEu8224': ['PgShr8'],
             'TixFk2mnRGmH7D+isnc6NlBG8oo': ['PgShr9'],
             'Z9r7iudOStNAnD2R8sS/a0NLPo4': ['CbNip1'],
             'HFbVvhtS4NsoeBT6zrmta+FhSRQ': ['SrzPit2'],
             '8YqZEOilrh2ovcRZXVjqo3QGyew': ['SrrPit2'],
             'u0Az6FmxYdt1zZ7KAb0R9synuxY': ['Cri_9402'],
             'EraXn+bB54nvg0L2qhT/ElPmkRU': ['PGTG_10537.2'],
             'bP5qPrQfvvZedva+tosOoVYBUoU': ['PGTG_16791'],
             '/jqX1ZRn2+Bap87fD0tItRVLYxM': ['ZtNIP1'],
             'QzCxd8pBxbyyNxO6/6KrFyL966I': ['ZtNIP2'],
             'WDAjxy4HHFh8S523ep5bNKIJAww': ['ChNIS1'],
             'RLtJFuDbLiajQlEq0JjVWm3mYq8': ['MoNIS1'],
             'f2K5+VZ5SBmvs7g+WTWb2uqUrYA': ['HaATR39_1', 'H9BPR8#PHI:4771'],
             'CnD3XfrR+vVZadpcbqav62xnmiw': ['HaATR5', 'E6YE64#PHI:4735'],
             'wBBAV9CaqWIgd3Ft3nykjagjL0w': ['HaRxL44'],
             '+RVHe0wYvKqlU01E65a1b/Kc/Sg': ['HpATR13_1', 'Q5G7L3#PHI:328'],
             'CvfIBQGIowzpPuPDt5x2fPduDRU': ['HpATR13_2',
              'Q5G7K8#PHI:2402__PHI:2410__PHI:2423__PHI:4754'],
             'UIwd+GYA4T63xlzbkZUnuKUp44E': ['HpATR13_3', 'Q5G7L2#PHI:2422'],
             'YIoM8NLoQWuNfNKy8QlHA2UOKVo': ['HxATR1NdWsB_1',
              'Q4VKJ6#PHI:531__PHI:2420__PHI:4253__PHI:4254__PHI:4255__PHI:4256__PHI:4257'],
             '8HbYVc2NGpWD9KfgtaiFb7My/G4': ['HxATR1NdWsB_2',
              'Q4VKJ1#PHI:2421'],
             '+e6KIuroB2ydJtAZjI4GnepMN7g': ['HxATR1NdWsB_3'],
             'Xrx7GBJTPWQI/9gtUr7qb3lnDAM': ['PcF', 'Q94FS7#PHI:665'],
             'RaWzDdiB47ZbkO3NblHG4YCGtYY': ['PiAvr1', 'D0NVB5#PHI:5113'],
             'o9KhoNWPpCvMREoJ2HcMpb+duaQ': ['PiAvr2_1'],
             'JVwFM+UPUFooBIW4cl+nsOmxObI': ['PiAvr2_2', 'D0NDJ8#PHI:5088'],
             'PfJigl8gJJdhm09e5n2TI7iQ7B0': ['PiAvr3A',
              'Q572D3#PHI:473__PHI:4958__PHI:6374'],
             '8voyD4pGO9dcdti2T6tlkUdRdnw': ['PiAvr3b', 'D0NXM3#PHI:4206'],
             'yZata6VGzkqxyftABGHExA0s+EI': ['PiAvr4', 'B1NNT7#PHI:5098'],
             'tHti6UPHMUQ7RBQJD9+qBSidX34': ['PiAvrblb1'],
             'ZbFW2fo9E5XmI7aAwlVjRuPm4IY': ['PiAvrblb2', 'D0P1B2#PHI:4207'],
             '/UkpxXmskZ1NhuZIe1oLPCufyb8': ['PiAvrVnt1'],
             'DR7hmx0bZ09TCIZi0ULz2W1yJDo': ['Picrn1', 'Q8H6Z6#PHI:656'],
             'x7z7dEb5wuYbxYZUJNX1kR7VbNU': ['PiCRN15'],
             'ANtxFseu7ZPicJ7Ce05qMuBNSp4': ['PiCRN16'],
             'z2paiO14GAYRj+wiIBcibiNWxLM': ['Picrn2', 'Q8H6Z4#PHI:657'],
             'uvCO7Dl3XNELVtHsosVS3+6E7YI': ['PiCRN8'],
             'xWERthnzXq1FD4Si8ZffDF6Mozg': ['PiEPI1', 'Q6PQH2#PHI:4251'],
             'cnNkGRHpMBTtSQIWkeDAc6LhttQ': ['PiEPI2'],
             'bsXbJRkEN4qAy3yvVWfyZDHLdEE': ['PiEpiC1', 'D0NBV1#PHI:6286'],
             'f3S4JHbOlfwpSXRG+8iPwECteE4': ['PiEPIC2A'],
             '06EPMGYsJEz9xRT4uhgH2/M2rmg': ['PiEpiC2B', 'D0NBV3#PHI:6287'],
             'QSJmoHsQ6WtZqI/WDjIUClO4+kM': ['PiINF1',
              'Q01905#PHI:111__PHI:760'],
             'OFRhffJ71MFy6+CYdxAKFP9eIFc': ['PiINF2A', 'O42718#PHI:762'],
             'v7n6Cx7DvLJwB72xLpspBzH8/Ns': ['PiINF2B', 'O42719#PHI:764'],
             'fqVRMsQimKBw5aXUtsRUqxY35QE': ['PITG_03192', 'D0MZL5#PHI:6301'],
             'rdvTqPEvstpYS9bRaTEC8CEf2b4': ['PiNPP1'],
             'aRy4NXLP5quq2zdx7ZsBD1Ic4qk': ['PiPexRD2'],
             'jdIoaW1H7sbbRzy7yn4yOdEVui0': ['PiPexRD54'],
             '87+w1UIdOcmwCqbn/dY6ZARWzwc': ['PexRD2'],
             '0m3JmW7dmIP1pS77gBbE8A6VtPs': ['PpPSE1'],
             'XnlcyGlVuTbaoF5EUltQDLlz4jA': ['PpCBEL', 'O42830#PHI:660'],
             'wLl7EyAn35umesE4VHfpF517eSo': ['PpNPP1', 'Q9AT28#PHI:666'],
             'DXHE/H1miPiTlS5AAInXTtCyxv4': ['PsAvh18a1', 'PsPSR1'],
             'FaE57Fxx5e0CMwA7thLXEg+ssT4': ['PsAvh241', 'E0W4Y2#PHI:2727'],
             'jre2WxK2twt6B6rbVeh9XC4ioCc': ['PsAvh5', 'E0W547#PHI:2692'],
             'TGIdTs5UZ3y+bFW9bs8pLRtH4c8': ['PsAvr1a', 'A4L9T6#PHI:5107'],
             'fvLftj462rUUp/ww4i7dwX2nqrc': ['PsAVR1B_1',
              'G4ZRQ8#PHI:2891',
              'Q8L552#PHI:530'],
             'dUOcWwXcU885QzN3h+qVTo0ti4M': ['PsAvr1d',
              'L7WWG5#PHI:2941__PHI:2943'],
             'Z8RVRqxOrfbJHVYLUDSZDIa/WlE': ['PsAvr1k', 'B2C6F5#PHI:2890'],
             'vW6olfr4MQaIBdkWIAj3O8Q97eY': ['PsAVR3a', 'A5YTY8#PHI:5085'],
             'l3+E2SoldllZ6BoW4KpLYhqrvSk': ['PsAvr3b_1', 'F8UNP0#PHI:5048'],
             'eLGJKObWZ+zYBkkyb1vnJBx1NAo': ['PsAvr3b_2'],
             'jynRqRtVNZvD9l+rGOkyLS4CfBg': ['PsAvr3c', 'C0LF23#PHI:5112'],
             'jhmchK/0s6bK0ll4o5wG7ZMDv6Q': ['PsAvr4/6', 'A7L812#PHI:5101'],
             'cn5oetQO9jRYXqR3kKCAzhqyulo': ['PsCRN115'],
             'xyj51ky8hZcjZCw0l6SJqoFA82k': ['PsCRN63'],
             'oDz0GJCN0VN550kbRoWrISrWl7k': ['PsGIP1', 'Q945U0#PHI:652'],
             'sUVXmaCFwTDFWjJyEx0MyIg0ftc': ['PsGIP2', 'Q945T9#PHI:653'],
             '4DeL3NnjVPg6T5W8WMVmKuRuLWg': ['Pslsc1'],
             'kZYekzD997MRnstTABvmg8xwKwM': ['PsojNIP', 'Q9AXL8#PHI:658'],
             'KwICH127zlrijjt5TnDzuh9aJPc': ['PsPSR2', 'G5ADB3#PHI:3353'],
             'jzjboGcckcdIwpquE+Nb/b/zW7E': ['PaSD21-1', 'Q9SPD4#PHI:664'],
             'JkjowqzqpKfP2Ol2PHWpkgZfklE': ['A0A016PMX1#PHI:6978'],
             'Q+asnIHAkXU+rWcw5V5ARa/FoUs': ['A0A016PTV5#PHI:5393'],
             's+m6ykbQ4wJFWe2HKXv+jcEg+9A': ['A0A016PUA3#PHI:3659'],
             'UalS3WxULX2hDL1nxZPCUlANKcU': ['A0A016PW58#PHI:3658'],
             'pUJ5eOOdrDQwWZJxNYS7Iz9kh5c': ['A0A016PX00#PHI:4602'],
             '7O03KqqkibQqwxlKm0BfAbzNbp4': ['A0A016Q4W5#PHI:3657'],
             'UuZ5MZzLVgPjSzg6J9YrRFeiXCQ': ['A0A023H5D8#PHI:6442'],
             'jGc3iqEWB9odW9BN7qGdaFUidQ8': ['A0A023NA98#PHI:3354'],
             'aTkHqhj6v4UjEsctNpa++PsZsmE': ['A0A023Y9U3#PHI:3462'],
             'TdNxeywgyF4jlBRN1mpPZnYkZXk': ['A0A024CHY2#PHI:2935'],
             '17RyAIyvMeahaHC6NNthA8b1Voc': ['A0A024CHY4#PHI:4837'],
             'IUm3dONNX5op3L48WMhoHb4DQ4E': ['A0A024CIG8#PHI:2934'],
             'vY2UeWEHGA7L1rwZFbHSxwYrsQI': ['A0A031WAR9#PHI:4612'],
             'u2Ssz31qgot1d6D8WRkHpqNhAOs': ['A0A031WJD5#PHI:5260'],
             'Kw9lUBcfqr147u4Aptt1j/+VlZw': ['A0A034UTT8#PHI:4481',
              'D4HWX6#PHI:2501'],
             '+/N1ZAN6x/zWK5h0PWFzXHuX+B4': ['A0A034UYN5#PHI:4480'],
             'Yz4HIyYbHo9N3J6+3beZslt/uQE': ['A0A045IPH0#PHI:5351'],
             'Cc3Q6QiXcbKB8oe+MYtkCy1CeEc': ['A0A059SVJ2#PHI:4916'],
             '4ffy1yt4d4+ElnEExNHFyahov4g': ['A0A059UDR8#PHI:2897'],
             'idnw9a9lVkxZbYyqG3kpSYmonw8': ['A0A059ZQI8#PHI:7668'],
             'VoqIY5S9mx3A31EV/8msW0vJodo': ['A0A059ZR97#PHI:5240'],
             'K3ZznTj6G7q/bla4dT8hEcudmI4': ['A0A060HKF7#PHI:6439'],
             'f065qzXDTwGo4SpDU76QzuIVPFI': ['A0A060VFL5#PHI:7478'],
             'VDJ3H+yALAAnsMukKDsF3mqr2oo': ['A0A062KYV9#PHI:6493'],
             'f7104qvnT3pGWAcOrWaOx7SMFZs': ['A0A068B0X0#PHI:7257'],
             'z8HYV0uYaBsHxd4YAUnpRdzFpZ0': ['A0A068BFA5#PHI:4468'],
             '6lc6JqtUyWmCX0sS2+1tUgGZNuQ': ['A0A069B091#PHI:8776'],
             'KntyQ4KY6MBbNxbRaZK65OFs3Y0': ['A0A069B9Y9#PHI:5323'],
             'S0UF2fuWfbMGJ0WcLjiWz92179M': ['A0A069BGU6#PHI:5334'],
             'aLAi/utC6Z+3LdLirYyxsF51BPA': ['A0A075P597#PHI:3468'],
             'TTazJnI8NjxwIpLt2WpBOrtgbv8': ['A0A075TRB3#PHI:3301'],
             '2MtTXeh9e3rmrXTDsZCUsbSdSUQ': ['A0A075TRC0#PHI:3299__PHI:4505'],
             'ajwUGTkDWozFa/T2cS2ZEYvt72g': ['A0A075TXZ8#PHI:3300__PHI:4504__PHI:5352'],
             'f07mq1NKaFTPbBl+UtftevlIorU': ['A0A076LEI9#PHI:4614'],
             'd4JmCPAqt1E1J9rhi8Q/t3QGEZ8': ['A0A076ZHT9#PHI:7511'],
             'fE8/qrNCuMxmyE1CaXWCOuNbmyI': ['A0A076ZK32#PHI:7497'],
             'LVds7oJU+F4ztVmHRGo6/B2EN4o': ['A0A076ZK37#PHI:7492'],
             'jfJqABaDxGtjJViCpAyjxpk+v3Y': ['A0A076ZMY5#PHI:7520'],
             'eyvTMMXBsaNC8xUtlI6v2ssvKKM': ['A0A076ZPI5#PHI:7533'],
             '77SB818noZ3Bzr35iohZ1ohzbKI': ['A0A076ZPI8#PHI:7528'],
             '8edis3EA1TvvLJAGwZKbv90XjLk': ['A0A077D1S9#PHI:3292'],
             'ABojhWLe4HEJiHthRccBOZhaSA4': ['A0A077UVP4#PHI:6342'],
             '7Woxn4t5NLogEK8X/AAR/sp7BtA': ['A0A077XE85#PHI:6130'],
             'VCUp90FQW9dIyJyDtSLp/pfDwfc': ['A0A077XHZ4#PHI:4648'],
             'qSy/HB7xvPH3Bpacmhlckyyav3o': ['A0A085DFX7#PHI:7487'],
             '4ZgUdqDfuY6ga0ckFBI5pSsoe5o': ['A0A085TMV5#PHI:6219'],
             'g8op55mtlZ6BgWdRLunlNrIdZqA': ['A0A086IIR8#PHI:7496'],
             'rTiBeqeroOvK9vtwBKHvf+xKXyI': ['A0A086IQC3#PHI:8968'],
             'XMNdCVIeyoqGrjDXZgDdaPfn5EA': ['A0A086VCI7#PHI:6912'],
             'aSJsYXLdb7WgoxNPZxRkr1Bi7sQ': ['A0A088MWZ0#PHI:4600'],
             'zVXAgLR94PdcUEU3/rYi9S6GzX8': ['A0A088U1Z5#PHI:7250'],
             'ZCZjkDnDZg0PfUZ8Facjd9CswPQ': ['A0A094Y528#PHI:4886'],
             'T2UTIzADLFU1AXfTpRm3Y6XX82o': ['A0A095C2M1#PHI:4556'],
             'WMjjwKD77E+g0gGAQ7WqGP9QmtA': ['A0A095CFR8#PHI:3332'],
             '+56Yw802/m+vA8YiAlsHH2kt+Yo': ['A0A095CIZ4#PHI:6188'],
             'dhtSaJ3TS7SwMEfJN0Sm2ZM/9Z0': ['A0A095CJ15#PHI:4557'],
             'JGoNxGyg6yTnDeD1xXy/Pql0MiA': ['A0A095EN78#PHI:4555'],
             '3k3EshChnEZakt4jNkVRJDgyEoM': ['A0A097BQG4#PHI:5539'],
             'OH+nKd1B94Dffai36ZsZj56g68o': ['A0A097BVQ8#PHI:3463'],
             '6IdxO4x0bmNoEsn04hn8icyMJm4': ['A0A097IB07#PHI:3281__PHI:4819'],
             'Gjc8ZhDhQct7P/v7pI/3+XMx88M': ['A0A098DCR9#PHI:5838'],
             'B0eMHLf6rVViPatOKIhM1LeC5Wg': ['A0A098DG53#PHI:5847'],
             'JHuIZ1HymXVJl4pZc1wvAzAbUro': ['A0A098DK45#PHI:7123'],
             'Ac47f9twtT1nGi2LOCrxwBG9YsU': ['A0A098DM90#PHI:5864'],
             'aWnyDR8h+omngx0f2Iim8Wgg/4M': ['A0A098DMJ4#PHI:5801'],
             'CgqR1BOvyFRRs0Ww63v8ft3xUdk': ['A0A098DRE2#PHI:5846'],
             'YP7YZaxMWMQ+jLwrCi4/DSt2xR8': ['A0A098DTB5#PHI:5802'],
             'Owcl4uUQ+PFznkjQvNcYlN4nSME': ['A0A0A2HZI5#PHI:8720'],
             'O2WIWR5yPZdoQFdIz8hnoAOJduk': ['A0A0A2ILW0#PHI:7664'],
             'Qx7gbwmCqOKwkeWoQEx3OtVok0o': ['A0A0A2J5T2#PHI:8718'],
             'GgejeWLnu0UUQ6MmNtiXo4yUWNg': ['A0A0A2JB06#PHI:7667'],
             'pc+9d3wPnB55m0mMxSaDN7yA5Fw': ['A0A0A2JPI4#PHI:8719'],
             'zA7h2XkMn+ifwfQvlFh2qAiyw3Y': ['A0A0A2JVC2#PHI:7665'],
             'Hap8Ykc0qTGMZAuaSJmGIWneus4': ['A0A0A2JY92#PHI:7666'],
             'Gywpu8KkgS7wQZY1GJDtZ0qpy5w': ['A0A0A2JZB1#PHI:8722__PHI:8724'],
             'IjSzfSnhUgmzvXJJnxRsZEEQIdc': ['A0A0A2KRU1#PHI:6881'],
             'dCkg9zoO/cHpOwHKbLXOX9ifaf8': ['A0A0A2L0F6#PHI:8723'],
             'j1KM3ZP5gbZwGpYh6VPdp3ReBtw': ['A0A0A2VP56#PHI:7815'],
             'QWMlNZcsBYX+l+pBI33z2QX+8S0': ['A0A0A7DLV6#PHI:4722'],
             'H4njwQjaTSM9mL0hGGmBlbXaGss': ['A0A0B4UFC1#PHI:5439'],
             'o6gD7ggnR6UjVAzwAsIPeFfyAA8': ['A0A0B4ZUY6#PHI:6347'],
             'nuFs8k2XJMlDOPGgF0N83BIt3Fs': ['A0A0B5J4U6#PHI:4609'],
             '236smIpCrGopXHuXovZvKSUz0MU': ['A0A0B5JGW8#PHI:4607'],
             'bdqKqOtHu/rgK73pchJaGlteXnA': ['A0A0B5JJZ6#PHI:4608'],
             'OqEryFz9YJeTVbMmdlpVUJ8KlFg': ['A0A0B7LYL4#PHI:6320'],
             '+xzUOQNySMOXEcDH9ZyINcJyl3o': ['A0A0B7MDN1#PHI:6322'],
             'Jqfun5TYvvu09rCFDRlFY4N0x5Q': ['A0A0C4DHR8#PHI:7682'],
             'x1+r/AQsnYS8ZOoe4RBYObG9Nec': ['A0A0C4DHY2#PHI:4879'],
             'sSorJsUmZdn4Lk2OrL2LEg+13vM': ['A0A0C4DHY4#PHI:4880'],
             'Irk6hHNYWscd7uY9gFlS++f97tI': ['A0A0C5PM61#PHI:6589'],
             'dy6spqypdhu5ReuGbbY6SOHBA2Y': ['A0A0C6EZ24#PHI:5587'],
             'KOT4U/XjUAvG0mhuYNjAA7I/pgk': ['A0A0C6FEF7#PHI:4877'],
             'aEnhdjZ2pXTe/yUFyI8ppRN/SW0': ['A0A0C9CDU9#PHI:5108'],
             'zmrgPheuznSPKq9FYA2AybroEyE': ['A0A0D1C7C6#PHI:7046'],
             '7mfXezBZvtgkwwOlgeKKydeUfq8': ['A0A0D1DP45#PHI:6426'],
             'nBGd5djUCzhb6YbZ2I18yZguw3I': ['A0A0D1E0C2#PHI:8762'],
             'ZvoBzQtxW+JKgKGpMLI1g8XeHK4': ['A0A0D1E5A8#PHI:6346'],
             '8V7ZK4bBqi6lzaI5JHjXXDyU8Mc': ['A0A0D2X9S0#PHI:7684'],
             'rJ1Q40gqpe1ZGYGeheydfRZZ88A': ['A0A0D2XDM3#PHI:4724'],
             'XzlnCsj1Sh2FUG0h7jNdXYh06o4': ['A0A0D2XEI7#PHI:9050'],
             'namHUgH1OswN2V0ag0JU0B3YwC4': ['A0A0D2XJY4#PHI:4723'],
             'NNZaQ8NHNdd0lrXMztRiDR34hSA': ['A0A0D2XPQ9#PHI:7636'],
             'yx2tDL1E3BuEH3yrbXJjyODrhYI': ['A0A0D2XQS0#PHI:6317'],
             '0hvObfkBoTsFfRuGksRS7XqEPfs': ['A0A0D2XVZ5#PHI:5236'],
             'zA1aJdhb09pv8KfZz3hdZi5PK6o': ['A0A0D2XWJ8#PHI:4727'],
             '+VmfehxquSO90wUzD/DZwCw/cvY': ['A0A0D2XZG1#PHI:5484'],
             'J4NqVPIk1PTtTt/VHevoiZbf8q0': ['A0A0D2Y8P9#PHI:7588'],
             'vN/9rkXXtSUkI+H3atBpfa3kdfU': ['A0A0D2YC96#PHI:4725'],
             'Luwa/r0YWEZOVmXQh8mNDNslrlU': ['A0A0D2YD74#PHI:5483'],
             '5Aqu2nc7xctnIoHncmazu1Y8nbQ': ['A0A0D2YG10#PHI:6936'],
             'qNVu/RGYJfS3t4UDxT9TLpVgKSI': ['A0A0D3LX64#PHI:4618'],
             'NMpB96FArK5VSEQSSjAF2A9+5lI': ['A0A0D3LXD4#PHI:4617'],
             'dfpr3QNPYmyYo8PDEfLWAJ6Zcq4': ['A0A0D3LXF0#PHI:4619'],
             'StYwzMDD1209dD8czKOBPEmGpEo': ['A0A0D3LXG7#PHI:4620'],
             'u4/1Ilr2fwdxrIe5LpqW4JLPemA': ['A0A0D3QGV4#PHI:4687'],
             'Ry2gQyu+zfPqe0xbxEp7yCUH/zI': ['A0A0D3QGW9#PHI:4685'],
             'MrUl1JaDxp9njwuWPABt4oZtI1c': ['A0A0D3QH83#PHI:4686'],
             '3pbHMzC3a4wbh7gVvlDYj3YX+Xk': ['A0A0D5BX39#PHI:4750'],
             'CDHlVKcux/yCsLFFyCISLVN9b00': ['A0A0D5YHB7#PHI:6264'],
             'h4lZem9Tuss83USWV63JXJ7mA7o': ['A0A0D5YID2#PHI:6263__PHI:6383'],
             'GDLGy5K8FstO8ZsgU8nGmTNVWE8': ['A0A0D5YKF1#PHI:8812'],
             'qCRDpzhjo78Z6X6D7dxHxXZN3j8': ['A0A0D5YL42#PHI:7669'],
             'pzlkNlwuG9ecbLFm0sxfc1h/nrI': ['A0A0D5ZBN4#PHI:5103'],
             'm0KYIumnxxd0RHN9HtbR3RGvR3U': ['A0A0D6I3U0#PHI:5573'],
             'zRvofucNSGfBWPezflV6fTf7sl0': ['A0A0E0S4Y7#PHI:8646'],
             'zwxc1vg2rNy3PXiFA7IlWcsXs+o': ['A0A0E0S648#PHI:5780'],
             'btjZXwXGi+5Ubn0ekgcvo30U7hw': ['A0A0E0SA40#PHI:7025'],
             '9+ggmALo5TPuMugT1wLPxBhQIto': ['A0A0E0SEE4#PHI:6259'],
             '6pDoDtpKciu8UEPAGcXXFwWxs9I': ['A0A0E0USK4#PHI:7069'],
             'Md9gad0OUsBK/Ofm4G7rmvM0d0k': ['A0A0E0UVG9#PHI:6144'],
             'AvzEAoGfWmO1sAvmZIgHqQpMIUw': ['A0A0E0UVH2#PHI:5478'],
             'jP74Ltx2tz62yQ193IlKxpfY4tI': ['A0A0E0V727#PHI:6913'],
             'J+FgSL2QsSiq9Nr4P870YoudBS0': ['A0A0E2G7L7#PHI:6570'],
             '3Z2INfK+AHJglytAOER6eMi0aJc': ['A0A0E2GV55#PHI:6089'],
             'KjNEQ3nOfi4urUaHnX2HOyx/xBM': ['A0A0E2ZL83#PHI:7066'],
             'o6l2dcK4I4FB6PRDeQ/LNu7tgjY': ['A0A0E3GIM3#PHI:6379'],
             'u6PEbVm0gSpMdfFApxCbi3QpdeM': ['A0A0E3USI9#PHI:4845'],
             'TXdOLF5TBnhQmtzUMTMfnPdbqHs': ['A0A0E3X1L1#PHI:5011'],
             'IFqKVwW/97hzJ2ky9RiAKQD1QCE': ['A0A0E4D570#PHI:7862'],
             'vqHF2Zem8jyi3UQAXrtWju3fldU': ['A0A0E7TWI5#PHI:4994'],
             'ymPSnxYYJIEavHBnp+eRPNuk0dc': ['A0A0E9B8Z9#PHI:6361'],
             'ZPgBJrT25KSarGuwXGyBB10jpkI': ['A0A0F5B4S7#PHI:6595'],
             'rFgMZoBvfS45Q6U1RhWAuQTV8AU': ['A0A0F6AXS9#PHI:8808'],
             'RpdCMXrL+RxvZ5+50VKf4uhYFa4': ['A0A0F6AYC2#PHI:7263'],
             '/ankUdZBOn1THTlVWTYX7bGROqw': ['A0A0F6AZ24#PHI:7210'],
             'wuOuKZnFkIi3stDqC8nR00v136w': ['A0A0F6AZ25#PHI:7211'],
             'WnU4+z02ASkaUSw8oX2r+gmrbEs': ['A0A0F6AZ26#PHI:7212'],
             'dfMvPzsweM2YLMsY0nfYjoWtgTs': ['A0A0F6AZ27#PHI:7214'],
             'pu2HU57Ha3y98MtGiVTi2yUcSu4': ['A0A0F6AZ28#PHI:7215'],
             'BmqcMmdQ6KvXvW0caUhK/M6sAzk': ['A0A0F6AZ29#PHI:7216'],
             'Gf9rDdTXc43X7ahbxTDMDUz5PkQ': ['A0A0F6AZ30#PHI:7217__PHI:8288'],
             'm7rY4f/VLZ00MDCjm5QWLIJFawQ': ['A0A0F6B0W4#PHI:6893'],
             'NKsiQkyUnHnQfa5f+y1u2TXgL6E': ['A0A0F6B1M0#PHI:7225'],
             'LI3D0kknjGQmJ+zU6oLlPENSEwc': ['A0A0F6B1M3#PHI:7355'],
             '9wv/jliNTT44wJH5L2CUMBCQZJk': ['A0A0F6B1Q1#PHI:6982'],
             'S3Os9+Zub7rVJWfbRvrvf6T1zBA': ['A0A0F6B1W8#PHI:7660'],
             'hx4ajPMPGUskvOjZvKK3261snPY': ['A0A0F6B1W9#PHI:7659'],
             'lmFcWfblcE0IPZ+DuICcY7/kPqk': ['A0A0F6B1X0#PHI:7658'],
             'cj8kNGmR7TyKMzVuL6y5VAnifk4': ['A0A0F6B1X1#PHI:7657'],
             'bzq4DIkaK26ZJ/YrDyVe0kgBBQU': ['A0A0F6B6S3#PHI:6985'],
             'rIVfOv+GSNvbqX2ajFlyZIOMZ38': ['A0A0F6B6Z8#PHI:6984'],
             'phEPraaBCalrcCUfz4BiUSA+w/Y': ['A0A0F6B6Z9#PHI:6979'],
             '9OcP8ArnqNJwSTXjUmkYlWtNL8w': ['A0A0F6B845#PHI:6980'],
             '8AOScfVPREzgYxTN6zj1PwyUtBM': ['A0A0F6B9I4#PHI:6544'],
             '7zFrZeyRbEvKItV1MDvxvi8qBuA': ['A0A0F6BA89#PHI:7661'],
             'w3cdgnJB8Mm4D/atLIm0rd/PqXU': ['A0A0F6BB74#PHI:6981'],
             'cEjKoNWoaWeU42yo0AFZ1P36ZCQ': ['A0A0F6UGK4#PHI:6941'],
             'IFU5POnqTnI31aqx/R1sKA+PSCg': ['A0A0F7A0T2#PHI:7697'],
             'WEeZ3/4LnL3qlod4b+fOOtJiiZM': ['A0A0F7A3A1#PHI:5372'],
             'JyGjhZ0SfNwMgJ4HRAtL+7s1Uqk': ['A0A0F7R1R1#PHI:5250__PHI:5292'],
             'PtOe7sYJW6RWnnr13OM6oFDh/t8': ['A0A0F7XLK0#PHI:5469'],
             'z1WtlWXE+AU240j8ZGmAgjoXQgU': ['A0A0G3K3H0#PHI:8554'],
             'sBJdE3QAHHWPh7Hp1gqmrt2kaD4': ['A0A0G3K3W0#PHI:8870'],
             'Yop3IQa/d9WIFN2uPhtwrDUUwJw': ['A0A0G4J7R2#PHI:7735'],
             'w9YU+/5H0y2hdjpYkOMHduE+YGs': ['A0A0H2UQ73#PHI:7598'],
             'lYNxW4WNsufAdTe15sp9htwK2n4': ['A0A0H2UR33#PHI:7679'],
             'RLa8GTaZFMIkH0nHECmFsu2HOdo': ['A0A0H2URB2#PHI:7678'],
             'PYgf52vTGyPd+ny6VfojQ0omvg0': ['A0A0H2URD1#PHI:7677'],
             '9RO5k3CIBBTlA7FD2ElDy+m1klo': ['A0A0H2URH5#PHI:7599'],
             'i6oPFP5aDWwNYPMCl+bjYa+UqgE': ['A0A0H2URT4#PHI:7118'],
             'Mp3szuid8maw+Wrza1buMbVGiNw': ['A0A0H2UX24#PHI:6507'],
             'YakmcpG9ayyXX/ec12CO4mv1Kio': ['A0A0H2WA86#PHI:5320'],
             'POpPOq8ZM7fddF8ReMWL6QqjmEU': ['A0A0H2WAC2#PHI:5327'],
             'PWUCy1eauIbCvxq5op197mix7Iw': ['A0A0H2WAR1#PHI:5326'],
             'Ns53Fq++wMnMR/a92SAMi7pvYDU': ['A0A0H2WKF8#PHI:5345'],
             'enUlToJ9pyiQZ+NUAi3AXHL83/o': ['A0A0H2WXT5#PHI:6629'],
             'htY1dyOkpez/h2HAv7jJ6lmZeyA': ['A0A0H2X2G8#PHI:7781'],
             'feTOrSYH2AZ8EDgz5FctE+FVQC0': ['A0A0H2X3T0#PHI:7255'],
             'ZSWndixpuFWJ/oCmxC7vvaoLbpw': ['A0A0H2X5Q6#PHI:5547'],
             'BHvKks9lrHFlUyz/Vo9jRM4BnSw': ['A0A0H2X6Q9#PHI:7778'],
             'IY3WiUYfaq/Nk9JA3UBkmrgwnzQ': ['A0A0H2X8G8#PHI:7780'],
             'y3+8fXCdhAFfRgQfBrj6NfjWYeM': ['A0A0H2XCS3#PHI:7645'],
             '1ED0+7drFDLS1U7BVf4GoEfpAmY': ['A0A0H2XE72#PHI:6581'],
             'bH4ylbcI4heHanESJel95jDUEpo': ['A0A0H2XG07#PHI:7344'],
             'Vt6rhENKYszrdbk5Q1DJQ35xt7c': ['A0A0H2XGW2#PHI:7351'],
             'XzZqZLzJyDaajNsH0JedO32sN3w': ['A0A0H2XGW3#PHI:7589'],
             'OpJ16xogUAFtlrCZNOuG/qiCqg0': ['A0A0H2XHD4#PHI:6628'],
             'N0Xkq3/sR3txNf8xahbeC3t6jiE': ['A0A0H2XHH2#PHI:2634'],
             '8snfTs43KggX2sCf1SW7GTmFuEk': ['A0A0H2XI72#PHI:7354'],
             '5QlsnZqr5aD5Crp89sIwx8IiqzQ': ['A0A0H2XIJ3#PHI:7349'],
             'SXuH4zlqlQMINlhPhjo/7f1l/qc': ['A0A0H2XK89#PHI:7346'],
             'YqSBhGhKpdifPrcwVojgglrEELA': ['A0A0H2Z644#PHI:6705',
              'A1AJK4#PHI:3789'],
             'e+QxZHVxnteUMcKBwV8xDfZ+TiE': ['A0A0H2Z6F6#PHI:8549'],
             '4BcCsbKqQqBlTdg7NbZGlkqh0Lo': ['A0A0H2Z8S3#PHI:7290',
              'P33883#PHI:6997'],
             'O2cKXAPQ/jKerYHlcHG6bm5voJ0': ['A0A0H2Z8X0#PHI:7299'],
             'zPCAyVrprSjytimZ7LwejLpLtIQ': ['A0A0H2Z901#PHI:7291',
              'P25084#PHI:5183__PHI:7655__PHI:8640__PHI:8641__PHI:8821'],
             'O4ApXX3A6N+rAlKjcB16vLhpYOk': ['A0A0H2Z9B3#PHI:7767'],
             'ypueXRocao7Io75gOeyZwTC2xwY': ['A0A0H2Z9C8#PHI:7766'],
             'qrUEU08cTx+xo98ZtbTb449pjD8': ['A0A0H2Z9T7#PHI:7305'],
             'NylihF/mRljEvWSKgY63+ttwHrE': ['A0A0H2Z9Z1#PHI:7303'],
             'LPRrq5RDRi+VF1gU3e+th48CJ4U': ['A0A0H2ZCE5#PHI:7389'],
             '6kLHjWVG4oIl1oJMiiP5iri0sLg': ['A0A0H2ZCZ2#PHI:7827'],
             'ZxGV9dfblf7nfnkQppjWv8g/u3Y': ['A0A0H2ZDI1#PHI:7817'],
             '9H+VZAr2m1BKm4gF4BodsilUG18': ['A0A0H2ZDM9#PHI:7816'],
             'j7v0fgODAgUHP7wFhmZi+eC3xTk': ['A0A0H2ZEE6#PHI:7288__PHI:8548'],
             'pGjS2kU6OBTJLCBWT09v6uvULAo': ['A0A0H2ZEG8#PHI:7289',
              'P54292#PHI:6748__PHI:6996__PHI:7656__PHI:8547__PHI:8822'],
             'VpBovihI0vjY5pPjpCodmnZ/mxU': ['A0A0H2ZEM3#PHI:7567'],
             '0LUDmS8m1BuRikSw/qtWvaYmve4': ['A0A0H2ZF69#PHI:7295'],
             '++ySrKBeAyVtF0R23sGjjsU+WKc': ['A0A0H2ZG25#PHI:7765'],
             'XRD8RwApw7hvfzijtahHH/nyPqE': ['A0A0H2ZG56#PHI:7296'],
             'iuMlH0jAYa30nwzXILHHmsa23Vk': ['A0A0H2ZGA6#PHI:7293'],
             'Cu2S5e7uanWQ++4Ex2tOTpaUYb4': ['A0A0H2ZGB1#PHI:7298'],
             'A3gfseD9wQhqDetq7J1cb1MDbqM': ['A0A0H2ZGB8#PHI:7294'],
             '7vKHQ2V45etzkbrkfFYAwXBQ4WU': ['A0A0H2ZGH8#PHI:7292'],
             'ndPH7t/L6AXlb3qfb5aRu/4bEss': ['A0A0H2ZGI5#PHI:7297__PHI:7304'],
             'FSq6PlMKx5KGF8PB4SmR2hHCOpY': ['A0A0H2ZGK2#PHI:5443'],
             'KhzyArWrrxKznTpXSAF5f+kAILo': ['A0A0H2ZKU3#PHI:7302'],
             'BBWKHffxv3qgWaBDWSPvuNYwpPA': ['A0A0H2ZL76#PHI:7301'],
             'v+m0f4rkLQstAg2YEV71rN8LIbI': ['A0A0H2ZMA3#PHI:6406'],
             '7ntfUcIj1/P5sQx+Y5uIrjqPGzI': ['A0A0H2ZP20#PHI:7800'],
             'HhSspI1v4/dglO9A+6EEQRGe7eY': ['A0A0H2ZPC1#PHI:7005'],
             'LSYk4U4it9tYwTWv7qhAX66Qhfc': ['A0A0H2ZPU8#PHI:7798'],
             'GJGHXhduFph26LLX4tu0xRFycRw': ['A0A0H2ZQ21#PHI:6501'],
             '9gwamo28Ga/o1rVwrh8cv6kEels': ['A0A0H2ZQ64#PHI:7799'],
             'E8Wb7JD5B/d7Gb8+P9dkvng6Cjs': ['A0A0H2ZR57#PHI:7007'],
             'SU2916wVfvjA7BJeZDCcQERUogk': ['A0A0H3B1G5#PHI:7996'],
             'DXrvKEzeJ6ojOfdDksqBItig3wo': ['A0A0H3B439#PHI:5013'],
             'YA9BdYa7gIGPDrBz//0hthJoGOc': ['A0A0H3B7J5#PHI:5014'],
             '+2TXm0aY0/FInir5Cba0cLU+tkQ': ['A0A0H3C0P3#PHI:7616'],
             'Psu+4RJ713h+npYc17fqtxSMWZU': ['A0A0H3C2N0#PHI:6523'],
             '9KtMBTcXMrxjIdxzC0dXqUneYBE': ['A0A0H3HU19#PHI:5336'],
             'Y5qna9Ccyp0o2gQozfVdBXnaxtI': ['A0A0H3HU22#PHI:5339'],
             'FAgNLiaV8P8PoqdnPpCEaA1dDT0': ['A0A0H3HU52#PHI:5331'],
             'SgQk2mWWPwI38aTBfSknOiVgi4U': ['A0A0H3HVA0#PHI:5333'],
             'A2udajih6tp5I8AO3b5fK6uqWpA': ['A0A0H3HVF5#PHI:5329'],
             'LXdAB8GBCT4ixnElyCStPvnBsaU': ['A0A0H3HVK0#PHI:5335'],
             'E9L+pOrBwKWgWfSzqq0N9tsTDns': ['A0A0H3HVR2#PHI:5325'],
             'CTtZA97Rz9si1YGKYp3ME+z7wBg': ['A0A0H3HVS0#PHI:5328'],
             'hjT9thtkxddGtLJIDbEsspFJXNg': ['A0A0H3HY94#PHI:5337'],
             'zZjGoO2IBpIvHfG1ogUxqYoPavU': ['A0A0H3I0G5#PHI:5332'],
             'mmVOg/LezdoIZcyA2xd1MV+n2tQ': ['A0A0H3I0I1#PHI:5338'],
             'BglnSVr+o5wLZJtaLSSNhfT0ATM': ['A0A0H3JLK2#PHI:4960'],
             'Dj4eSEzNWSxDAEn8LFFKSlgZgEE': ['A0A0H3JRA8#PHI:6673'],
             'Tce0KwTn+WwQpjXB4ZT6Wrm+YE8': ['A0A0H3JS81#PHI:6579'],
             'YowJO1elUjJdXvnxxKdCzPq/zTo': ['A0A0H3JUP1#PHI:6580'],
             'eH0celjFivfK/oDrQASeUAAi5kc': ['A0A0H3JWX3#PHI:6876'],
             'cIqSqtOBtlxE/K8BjjKaoZ98Ef4': ['A0A0H3KBG6#PHI:7348'],
             'Etym5ujrK2pp0EWBshadJ3auKm4': ['A0A0H3KBS1#PHI:7917'],
             'k3+b1cm3ZEE9TruC834Da0eYLhQ': ['A0A0H3KEX5#PHI:5242'],
             'YwgKeNF+586TFQ/Zj3Hb3isxU28': ['A0A0H3LBW1#PHI:6073'],
             'OGk/CiRwbk1g45fUcyrCJNplRRg': ['A0A0H3MDW1#PHI:7471'],
             'JGR3hRbK8u5rhYsypU7wwjLi8NY': ['A0A0H3MFV9#PHI:8633'],
             'hNNjmP8WSvIZYFyWCv4zTYW32/0': ['A0A0H3MZ24#PHI:7249'],
             'pRZ3I4DMCwj5j9xXp81cvGEreQk': ['A0A0H3N294#PHI:6720'],
             'lS5jZbs+9Cga6Q8BH3H/vHYVMUM': ['A0A0H3N916#PHI:7307'],
             'onPepN9rf29Q1Dz7fxRakZcyWxQ': ['A0A0H3NE08#PHI:5237'],
             'IUVEiB1DIL5AdcquRl0ypI2Wl5A': ['A0A0H3NKV1#PHI:6965',
              'E1WFQ7#PHI:4498',
              'Q7CQM5#PHI:2677',
              'Q8XFU4#PHI:554'],
             'jSo69CTT3Bn3B/UybnDF96PelI0': ['A0A0H3NMH7#PHI:8023'],
             'w4aL3Lk2JhapQG1UqZJ5obQBA4A': ['A0A0H3P9Y0#PHI:7198'],
             'sgOE3Ay3sgcpPOYUhhoXGMGLU2g': ['A0A0H3PA98#PHI:7196'],
             'DPKL1zRgD/uwENTxly6TH5p0abs': ['A0A0H3PB79#PHI:7600'],
             'E6YnjfkTS8h4EGLhAuPx7moo1h8': ['A0A0H3PEF7#PHI:8613'],
             'DQpHzNn3NS0DhaCD2tn9Bt27Ed0': ['A0A0H3Q531#PHI:6700'],
             'TV2SMtIjwc4SZJXZ60X7VWPw8V0': ['A0A0H5ASD5#PHI:6549'],
             'g4CHSl9CkTSeGLwnyBkr/fCKw8I': ['A0A0H6FRN3#PHI:6701'],
             'vhh1NDffYxspSb/4CSqvNLBzQvA': ['A0A0H6NEI3#PHI:6698'],
             'SMOytqy0K6wvBAKK9C2ijwovz9Q': ['A0A0H6RQV7#PHI:7770'],
             '3dBvsb4WmIZcfQU0M2K2otz6ahM': ['A0A0H6T2F5#PHI:6699'],
             'Npd7WH04BcGg+0vRByNLRQi5RlA': ['A0A0H6ZQK4#PHI:5549'],
             'bTU8655Q6ZiDB2ykJogy6rL12CI': ['A0A0J9UIM0#PHI:7685'],
             'Om6YcnIJZDMjuxLM9NEz4CMzRNk': ['A0A0J9ULI2#PHI:7683'],
             'w4hV3VhR/jY8hsBiPZfhEzLPYho': ['A0A0J9WWX0#PHI:8986',
              'B2SME4#PHI:2730'],
             'joV2NRPbJO6rXW+sLznUVXFABew': ['A0A0J9WWX5#PHI:8985'],
             '7JYrxz7LW71pwa58NcmaWmqbhME': ['A0A0J9WWZ0#PHI:7314'],
             'ggHH/gm2O+mMnf2QURnkzYURmLs': ['A0A0J9WWZ7#PHI:6954'],
             'W1/HYt3BmbavHtAAKJ7hRGBtDrU': ['A0A0J9WWZ8#PHI:6955'],
             'BGoWclFygeyWIuOidM3eKim4zGs': ['A0A0J9WWZ9#PHI:6956'],
             'ssq/+MOIW4fJaVClN4Hs66O/5Lw': ['A0A0J9WX00#PHI:6957'],
             'LKxQdEzo/vXalg99O0/eLfNQ0k4': ['A0A0J9WX01#PHI:6958'],
             'H1xxKJMHI9QNHmUd+q+HB7W4WKg': ['A0A0J9WX31#PHI:8983'],
             'OU6eWBdChJ4O22sHHPMRZ2H4fo8': ['A0A0J9WX69#PHI:8638'],
             'Ys2dySyDFYVqo4WvlOAFwcGCtkA': ['A0A0J9WXH2#PHI:8984'],
             'Q2srsuA8SqkOAxOmH6MB3pTs9Sg': ['A0A0J9WXH4#PHI:6953'],
             'qhogev4HHsNOeTPp7GsehJM+3s0': ['A0A0J9WXI0#PHI:6959'],
             'fQJu5SVAf5L4SLqCG6liF+0wmfo': ['A0A0J9WXI1#PHI:6960'],
             'KSGOqkD/kV84nOSnBZ4sBqte7x8': ['A0A0J9WXI2#PHI:6961'],
             '0/IlUf7d8IID6jae6SEEEWctxFw': ['A0A0J9WXI3#PHI:6962'],
             'yLq7Nxp+O2XLzyz/GOVGgnwSDrs': ['A0A0K0GFP9#PHI:7970'],
             'dAowpy3dfCJUYl1Oi9Rgg69wHqE': ['A0A0K0GFV0#PHI:9011'],
             'kAbgJwXj/GCcc5ECuslBERYmRek': ['A0A0K0GFZ6#PHI:7971'],
             'BM03aqMCdP408KJ8UUbkHh7emjs': ['A0A0K0GGA9#PHI:9012__PHI:9013'],
             'y6xvcg8LC/utfYz0lBON27BJ000': ['A0A0K0GGF6#PHI:9014'],
             'kbe0SfZjbhTml1yVDKw/rRrs+vM': ['A0A0K0GH16#PHI:9015'],
             '2pkClzmPR2FmJVpf9CFsTLiE/RE': ['A0A0K0GH83#PHI:6116'],
             'MXDI8cc6Y1ZGyifu4jz1W2Gg3Lc': ['A0A0K0GHD4#PHI:6118'],
             'GhNwRIdyMf4QbsYGK+Q5fzLfrUY': ['A0A0K0GHI2#PHI:8750__PHI:9019'],
             'xdgKExvS5Gt4TwdoOGLaTa3ZX0c': ['A0A0K0GHK1#PHI:6117__PHI:9016__PHI:9017'],
             'p3RXYT46/dPgJlNiFV1U5outuxk': ['A0A0K0GHP2#PHI:9018'],
             's56/X2NypDIjlvUnp9IIMeU9dOA': ['A0A0K0GHV7#PHI:9021'],
             'HmIc/0CqHkD/Q23YgSoqX/cFG24': ['A0A0K0GI30#PHI:5062__PHI:9023'],
             'mEbINxNru1PczVyow/G6DQAyDvo': ['A0A0K0GI67#PHI:9025'],
             'Ir8WKD3PoNHVSDNYonyUJfn3DWk': ['A0A0K0GI78#PHI:9020'],
             'MLYRROXqjICHYpcnsYL8cXcdlGQ': ['A0A0K0GI94#PHI:7972'],
             '9q0gaOHdQSRr+FoyyN3GRdoUcfk': ['A0A0K0GIA5#PHI:9022'],
             'zsXeq+XOeyOEVKXQApgbnCMskCQ': ['A0A0K0GIC0#PHI:9024'],
             'ixDQVmOXyqniW11WSN1GGcQFMVM': ['A0A0K0GIH5#PHI:9027'],
             '0KKL0mbZLbizOnbMgJosSqGzWJg': ['A0A0K0GIX3#PHI:9026'],
             'aR6Zf66BIr0FR/tTRtedhJdByB8': ['A0A0K0GJ33#PHI:8976'],
             'YX+5+OKEciX8jyzRWRvn3b2M8a8': ['A0A0K0GJF6#PHI:8974'],
             'MwLpwFAX9ioCYross/j1IbNs9GI': ['A0A0K0GJK9#PHI:8975'],
             '3b0jcAeA0/8+rmbYExlxJ8WL0Is': ['A0A0K0GJQ2#PHI:8978'],
             'TBuVXZ7Dmp7bdwesr8O4YPeOgRg': ['A0A0K0GJS5#PHI:8973'],
             'emubEy1kI9cQrDNcf/vnqMcvB5U': ['A0A0K0GJS7#PHI:8977'],
             'P4f1y7eRIA32oN9aFRfUFYkHb6k': ['A0A0K0GKB7#PHI:8980'],
             'FGSPDZ4xJWmsgzlBHWF15028Kjo': ['A0A0K0GKJ8#PHI:8981'],
             'fv5sNO+6RUCvUu8xR9seEQAQhX0': ['A0A0K0GKL7#PHI:8979'],
             'H4UiP0eEIRsRGYg/0o+xPEhmCqk': ['A0A0K0GKS2#PHI:6109'],
             'RttJJ2JGPOoI9O1nQRwVMlIKfwU': ['A0A0K0GL01#PHI:8982'],
             'SHcQmiHt+jkpHHc5foGIqHtm5V8': ['A0A0K0GL88#PHI:8994'],
             '7lZ8QVKC5dtQkb5f14B+kgY/p28': ['A0A0K0GLE8#PHI:7968'],
             'JZxWAMCzGCZQe/fVHXE6xSTWXQY': ['A0A0K0GLJ4#PHI:8639'],
             '1UaQo3dc+ntss7MLo+qtDfRGLvw': ['A0A0K0GLN5#PHI:8992'],
             'Dy9vsUi+LiP2e2fxJ5YbqPHBcrw': ['A0A0K0GLP3#PHI:8995'],
             'l6OHa7U+bB7n9pTgmmMgP09l/XI': ['A0A0K0GLS1#PHI:8993'],
             '0j++tnmuswhsDERJ7pNLr+chHkk': ['A0A0K0GLV1#PHI:8991'],
             'P06aAWE48zH7mnYLHUyoL/uATZk': ['A0A0K0GLZ9#PHI:8990'],
             'ei3j3Psxs7Va9qhKkKIoGmtLf6Q': ['A0A0K0GM14#PHI:8988'],
             'nZupCGNG5NSFf8FuPJ3uawCEbq8': ['A0A0K0GM26#PHI:8989'],
             'GOddp+u9gvwRGOPmuRAUhkVA8+M': ['A0A0K0GME1#PHI:8987'],
             'CGQ7KuRjOTu3llH9S5gh00m4PA8': ['A0A0K0GN07#PHI:8996'],
             'TOKrZrpuKUI1ftHBpw7nZuWDRag': ['A0A0K0GNX3#PHI:9000',
              'Q5GWK1#PHI:8032'],
             'xSPKk0KKRsrYanzikRd8e9CLu0k': ['A0A0K0GP04#PHI:8997'],
             '71NRVoYFeIB9qNgcLOsnZ8fFLk8': ['A0A0K0GP68#PHI:8998'],
             'Gt50kCZUD/Rs6+Lw4ihTIMgjpuc': ['A0A0K0GPN6#PHI:9001'],
             'xzGDdDx9zAHcVo9TdJJVmCH4/Gk': ['A0A0K0GPS7#PHI:9002'],
             'fAaaKdbqKlV9SL02Civ0PMFcOQI': ['A0A0K0GQ25#PHI:7967'],
             'zp5+jlzDm4lvyzk7VAEsape9oIo': ['A0A0K0GQ67#PHI:9004'],
             'MNMOoN7QtsnmR2QU09cVVIBKXeQ': ['A0A0K0GQF3#PHI:9006'],
             '5L78xWMU5DLMtru3LTM1cUpHppw': ['A0A0K0GQF6#PHI:9008'],
             'D4vXM0HzkkHtJ9ctY9gxDvyruoY': ['A0A0K0GQL3#PHI:6115__PHI:9009'],
             'fCyHytWGwOmzr8+2BT+YOclTkyE': ['A0A0K0GQN3#PHI:8738'],
             '3ze54HA4cVbTFT8w/J/nAumrZrY': ['A0A0K0GQR9#PHI:9005'],
             '837p9MA6bbBcxV/O2LooGv++WHA': ['A0A0K0GR43#PHI:9003'],
             'w5ekgoKW9OFKuwnI0diddNPxKhc': ['A0A0K0GRC2#PHI:9010'],
             'GWOnkZGaL6zR8tBdlMuVwvXe4G8': ['A0A0K0GRC4#PHI:7969'],
             '8HSFnh7KQOUtnJmwHS58tuJmnX4': ['A0A0K0P1E4#PHI:7615'],
             '33JoEkNKQ4ROp8GW4mfzzewZ00A': ['A0A0K2E3Q5#PHI:7985'],
             'ATGEwiE9Ive4Sr5M4/tieU9UIUE': ['A0A0K9N568#PHI:6248'],
             'lW5Ph12p1bCpfBc+pVKCffckNEg': ['A0A0L0VB04#PHI:6412'],
             'Y/BvJdpzDZ7lXWDprAFGcGkgDo8': ['A0A0L0VYD3#PHI:6167'],
             'dKW0Sop3rlQ279B6N+DViUncDA0': ['A0A0L9JQ40#PHI:6892'],
             'FFtLUTVvzR2e3l+NHU5ZdptFBnw': ['A0A0M2J080#PHI:6894'],
             '33Fu/csuXJDbRk9XMjgQ4P6BW4s': ['A0A0M5K865#PHI:5392'],
             'Q63cumCUIjDTBUQ/YrcAQtzRPbw': ['A0A0N7IDU3#PHI:6821'],
             'J2NSXGQ0kZvGmA67M6rbmhFxTgA': ['A0A0P9Y928#PHI:6650'],
             'e4qmc04hRdej6QyB6UWCU25UAco': ['A0A0Q0B337#PHI:6649'],
             'XMDjMU5IIH+XcDtIOaen1U2hzD8': ['A0A0Q0BGR4#PHI:7957'],
             'R5AF2jaEFQuJ073rVKLqS3cr5Qg': ['A0A0R1BRS8#PHI:6090'],
             't5vfZFSm8vkjH7cqMep7FOKjhLg': ['A0A0S1LL44#PHI:6975'],
             'QsfEgFUdmqK6GYt1BLTnZarfTfM': ['A0A0T7Z0W5#PHI:6283'],
             'WGdHcuBUNH6ifHSMCQOmTDbbBio': ['A0A0T9DH93#PHI:7219'],
             '4DN75LY0u+2PU/7k3bNcjoMOuy0': ['A0A0T9ML40#PHI:7710'],
             'tamlXPMZRY1pvqC0M3zhDoMoSJ4': ['A0A0T9V9T3#PHI:7064',
              'P52616#PHI:7448__PHI:8373'],
             'DJ9W1w2wWxEj0jgLxKaMncA3big': ['A0A0T9VUV2#PHI:7065'],
             'pbDGwEys3HU8/eOZU+U+ZIBBils': ['A0A0T9W9Z0#PHI:6104'],
             'ELusakPGBjImzCQySXdD4aqasjo': ['A0A0U0B015#PHI:7120'],
             '7iLgtvAww9QFa8AWMiVro+uDGwA': ['A0A0U0HH68#PHI:6430'],
             'BzWZ73m/6TBEXfzjF9BW+3fSRXI': ['A0A0U0WTG6#PHI:7861'],
             'Rlh+0m6mMnrMESfTs2BPAvkwtUQ': ['A0A0U1FMH1#PHI:8758'],
             'tpq8/2bVuQVrh2vzL1d/E4BigwU': ['A0A0U1FZZ2#PHI:8757'],
             'AJ52zZIXAmuRHSagwEwXBpv8e5I': ['A0A0U1G333#PHI:7061__PHI:8755',
              'P07637#PHI:2624__PHI:6460__PHI:6586'],
             '4IhHlL/UrXS8nNWrL9NH4DZSYkg': ['A0A0U1GNF3#PHI:7062'],
             's6VWTfUXWdtHouSUmQQxxUSphoM': ['A0A0U1J4N7#PHI:6697'],
             '7YHhxmzspwSpwga6rJJmGdZECqM': ['A0A0U1JBY1#PHI:8759',
              'Q7CPD5#PHI:8726'],
             'Q9pCCBCDbfpoHzifef31gRpG4q4': ['A0A0U1JRP7#PHI:6745'],
             'l2aTjRzT+zJPsSQrrgB4mmDj3ik': ['A0A0U1YU79#PHI:6363'],
             '39HuHCPbde78ukLHBKSsA7gVhXM': ['A0A0U1YU94#PHI:6362'],
             'Piaaa/NycxXhkras/usdzqJakmU': ['A0A0U1YUA0#PHI:6360'],
             '2fJ5x0Gp2yeWekWJAjI9qHH+b0s': ['A0A0U2P760#PHI:8636'],
             '1EO4NGkj+SXE1cvxLBVwdalNMNA': ['A0A0U4VHH3#PHI:8637'],
             'Q9/Fnws/n+iWa/CvELxYcOuURgU': ['A0A0U5BRC2#PHI:7866'],
             'uODe9c2CsSARy0yQ/HNuiLF4jS4': ['A0A0W0C7W8#PHI:6469'],
             'M8jpE1pLHXZb2Z9sjyna7DkfkhA': ['A0A0W0D9W9#PHI:6467'],
             'pB5Yz1c2AYzKZBruaTuglbkBxRI': ['A0A0W0DKD7#PHI:6466'],
             'K4PXFmFuDbkVtMRR/2TOAob8+uM': ['A0A0Y9VPZ6#PHI:6405'],
             'FNsAEaXsT8yD2V9HGGbeR4u/PN8': ['A0A0Z9GQC2#PHI:7986'],
             'C8JeL4csAgLNe7rDxn/JGeSwbl0': ['A0A116Q7Z1#PHI:8932'],
             'hSPNCORrLTzq/ZwUz1vRxeTTsrY': ['A0A125YGQ9#PHI:8582'],
             'Ekb9dirLf426Hd8AGir7alrX+KQ': ['A0A125YJQ7#PHI:6689'],
             'bmf+yc/R1gnMjyV43bWBkp/eJRg': ['A0A125YQQ2#PHI:6691'],
             'z6cYJ0K6jNL3iFxY46gQWWAjIkY': ['A0A125YRV6#PHI:6686'],
             'J2ZyhWr/ytIXE7gVVkwJB9ukz4g': ['A0A125YUL2#PHI:7744'],
             'p0hgZo5HMY6GTLCOubt3EhB975A': ['A0A125YVB8#PHI:6693',
              'O00933#PHI:4049'],
             'hXAp+QbumX0qjXV3b3m8LRkGSa0': ['A0A125YVM0#PHI:8903'],
             'wITIcEb/bXbPx5+h5rHppj1k0IM': ['A0A125YWK4#PHI:6695__PHI:8902'],
             'nwEPc6Pp5ig69Qm0wFkxLWTCli8': ['A0A125YWL1#PHI:8802'],
             'rcRaIRAdTBNZ9O3G6tW9Vb0pU6k': ['A0A125YYI5#PHI:6694'],
             'eX/vA+sX0YRRVywOqwABj+5ethA': ['A0A125YYK6#PHI:7628__PHI:8518'],
             'kBq9ZmLPwbkOFtoS6xfCH29ZdV4': ['A0A140CZL2#PHI:6179'],
             '58gS6q2i2mnAJ1cC8nVAUek/kGk': ['A0A140CZL3#PHI:6180'],
             'U3QAyoVmbjXzl4NKpFHokHbdtZE': ['A0A140GPJ0#PHI:7728'],
             'bjgbJ1vwatMdZvrVFwTwmeCgZ5A': ['A0A140GPJ1#PHI:7729'],
             'F6QwC99teUsyjV5XtN+/rvXrGdI': ['A0A151V4F3#PHI:6391'],
             '0WN8SiLOTb4zz5XZ19azJrcZMAM': ['A0A160EFI3#PHI:6706'],
             ...})
```

Do any sequences appear more than once?

In [7]:

```
[v for k, v in sequences_checksums.items() if len(v) > 1]
```

Out[7]:

```
[['PsAvrRpm1', 'Q4ZYH0#PHI:3495__PHI:5385__PHI:5501'],
 ['PsAvrRps4', 'Q52432#PHI:978__PHI:8026'],
 ['PsAvrB1', 'Q48B66#PHI:3493', 'Q52378#PHI:966'],
 ['PsAvrB2', 'Q4ZX49#PHI:967__PHI:5384'],
 ['PsAvrD', 'Q48B68#PHI:589__PHI:3494'],
 ['PsAvrE1', 'Q887C9#PHI:6377'],
 ['PsAvrPto1', 'Q87Y16#PHI:975'],
 ['PsAvrPto2', 'Q4ZLM6#PHI:976'],
 ['PsHopE', 'Q87X57#PHI:6899'],
 ['PsHopF1', 'Q88A90#PHI:3920'],
 ['PsHopG1', 'Q87W42#PHI:6353'],
 ['PsHopI1', 'Q87W07#PHI:7337'],
 ['PsHopK', 'Q88BH0#PHI:4018__PHI:8027'],
 ['PsHopM1', 'Q887D0#PHI:982__PHI:4008__PHI:6378'],
 ['PsHopM2', 'Q4ZX82#PHI:998__PHI:8430'],
 ['PsHopN', 'G3XDC5#PHI:983'],
 ['PsHopO', 'Q88BP8#PHI:586'],
 ['PsHopQ1', 'Q888Y7#PHI:984__PHI:2752__PHI:2879__PHI:8329'],
 ['PsHopZ', 'Q4ZX47#PHI:996__PHI:4968__PHI:5383__PHI:8426'],
 ['PsHopAA1', 'G3XDB9#PHI:987'],
 ['PsHopAA2', 'Q4ZX85#PHI:997'],
 ['PsHopAB1', 'Q8RSY1#PHI:990__PHI:2743'],
 ['PsHopAB2', 'Q9RBW3#PHI:989'],
 ['PsHopAB3', 'Q4ZMD6#PHI:999'],
 ['PsHopAE2', 'Q7PC62#PHI:995'],
 ['PsHopAF1', 'Q886L1#PHI:6365'],
 ['PsHopAH1', 'Q888W1#PHI:581'],
 ['PsHopAI', 'Q888W0#PHI:582__PHI:7336'],
 ['PsHopAM', 'Q52431#PHI:991'],
 ['PsHopAO', 'Q79LY0#PHI:992__PHI:7237__PHI:7265'],
 ['PsHopAR', 'Q52430#PHI:993'],
 ['PsHopAU', 'Q48BC6#PHI:590'],
 ['PsHopAX', 'Q6EES5#PHI:994'],
 ['RsGALA', 'Q8XYF7#PHI:5134'],
 ['RsPopA', 'Q9RBS0#PHI:5145'],
 ['RsPopP', 'Q8Y125#PHI:4969__PHI:5140__PHI:6306__PHI:6336__PHI:6364'],
 ['RsPopW', 'Q8XVQ5#PHI:2492__PHI:5144'],
 ['RsRipA', 'Q8XTK9#PHI:5119'],
 ['RsRipT', 'Q8XUH6#PHI:5178'],
 ['RsTAL', 'Q8XYE3#PHI:2922__PHI:5143__PHI:5179'],
 ['RSp0304', 'Q8XT13#PHI:5130'],
 ['RSp1239', 'Q8XQI6#PHI:5169'],
 ['RSc3369', 'Q8XU25#PHI:5131'],
 ['RSp0572', 'Q8XSA6#PHI:5127'],
 ['RSp0842', 'Q8XRI9#PHI:5399'],
 ['RSp1281', 'Q8XQE6#PHI:4025'],
 ['XAvrBs1_1', 'XAvrBs1_2'],
 ['XHpaA', 'Q3BYL2#PHI:6086'],
 ['XopJ1', 'Q3BTM6#PHI:2973'],
 ['XopJ2', 'Q8P4H6#PHI:6275'],
 ['XopL1', 'Q3BQL2#PHI:2688'],
 ['XopQ1', 'Q3BM44#PHI:4009__PHI:7127'],
 ['XopAH', 'Q4UV56#PHI:2918'],
 ['SeGogB', 'D0ZSS1#PHI:3752'],
 ['SePipB', 'D0ZTL3#PHI:3754'],
 ['SeSifA', 'D0ZV82#PHI:3756'],
 ['SeSifB', 'D0ZI08#PHI:3757'],
 ['SeSipA', 'P0CL52#PHI:7224__PHI:7922'],
 ['SeSipB', 'D0ZV22#PHI:3734', 'E8XL13#PHI:5110'],
 ['SeSipC', 'D0ZV21#PHI:3735'],
 ['SeSipD', 'D0ZV20#PHI:3736'],
 ['SeSopA', 'D0ZMG9#PHI:3737', 'Q8ZNR3#PHI:7919'],
 ['SeSopB',
  'D0ZTL7#PHI:3738',
  'O30916#PHI:6738__PHI:7236__PHI:7920__PHI:8298'],
 ['SeSopD', 'D0ZVN8#PHI:3739'],
 ['SeSopE',
  'A0A0T9VTM6#PHI:8678',
  'D0ZK57#PHI:3740',
  'Q7CQD4#PHI:6800__PHI:7921'],
 ['SeSpiC', 'D0ZWR8#PHI:2626__PHI:3759', 'P0CZ04#PHI:555'],
 ['SeSptP', 'A0A0F6B5U6#PHI:6798', 'D0ZV15#PHI:3746'],
 ['SeSpvB', 'D0ZHS9#PHI:3760', 'H9L477#PHI:663'],
 ['SeSpvC', 'D0ZHS8#PHI:3744', 'P0A2N0#PHI:4517'],
 ['SeSseF', 'A0A0H3NG92#PHI:8024', 'D0ZWT0#PHI:3764'],
 ['SeSseG', 'A0A0H3NB75#PHI:8025', 'D0ZWT1#PHI:3765'],
 ['SeSseJ', 'D0ZI42#PHI:3767'],
 ['SeSseK1', 'D0ZQW0#PHI:3747'],
 ['SeSspH1', 'D0ZVG2#PHI:3748'],
 ['SeSteA', 'D0ZXR5#PHI:3749'],
 ['SeSteB', 'D0ZI38#PHI:3750'],
 ['SeSteC', 'D0ZIB5#PHI:3772'],
 ['bgh06532_CSEP0264_BEC1011',
  'bghG008575000001001_CSEP0486',
  'N1JJX4#PHI:2898'],
 ['BghBEC1054', 'N1JJ94#PHI:2903__PHI:8918'],
 ['BgtAVRa10', 'Q09QS3#PHI:758'],
 ['BgtAVRk1', 'Q09QS2#PHI:759'],
 ['BghCSEP0105', 'N1J904#PHI:4622'],
 ['BghCSEP0162', 'N1JPG4#PHI:4623'],
 ['ChToxA', 'M2URZ2#PHI:4924'],
 ['CgDN3', 'O43115#PHI:164'],
 ['FolSix1', 'Q709D8#PHI:5284'],
 ['FolSix2', 'Q709E0#PHI:5285'],
 ['FolSix5', 'C0LT61#PHI:4849__PHI:5286'],
 ['FolSix3', 'Q2A0P1#PHI:4848__PHI:5283__PHI:7472'],
 ['Fom_2', 'FOMG_19011', 'W9ZT25#PHI:5067'],
 ['FGL1', 'Q6WER3#PHI:432__PHI:4212__PHI:4242'],
 ['LmAvrLm4_7', 'B9ZUL0#PHI:1132__PHI:2276__PHI:5371'],
 ['LmAvrLm6', 'A4F4L2#PHI:1131__PHI:5402'],
 ['LmAvrLm1', 'Q258K5#PHI:25__PHI:2281'],
 ['LmAvrLm2', 'A0A0A0S3X0#PHI:5401'],
 ['MoAVR_Pia', 'R9RX08#PHI:6343'],
 ['MoMC69', 'G5EI17#PHI:3122'],
 ['MoPwl2', 'Q01144#PHI:42__PHI:2152'],
 ['MoPwl1', 'B9A9V1#PHI:2151'],
 ['MoAVR_Pita', 'C1KJG7#PHI:2830'],
 ['MoSlp1', 'G4N906#PHI:2404'],
 ['MoAvrPi9', 'G4NJP7#PHI:4526'],
 ['MoAvrPiz_t', 'C6ZEZ6#PHI:2064__PHI:7896__PHI:8697'],
 ['MoAVR_Pii', 'C4B8B7#PHI:2136__PHI:4979__PHI:6500'],
 ['MoAVR_Pik', 'C4B8B8#PHI:3104__PHI:5553'],
 ['MoIug9', 'A0A0A7DM22#PHI:4721'],
 ['MoIug6', 'A0A0A7DLN4#PHI:4720'],
 ['MoMSP1', 'G4MKI0#PHI:2118__PHI:5540'],
 ['MoCDIP1', 'G4N8Y3#PHI:3213'],
 ['MoCDIP2', 'G4MML4#PHI:3214'],
 ['MoCDIP3', 'G4MX34#PHI:3215'],
 ['MoCDIP4', 'G4MVX4#PHI:3216'],
 ['MlAvrL567_B', 'Q6R659#PHI:533'],
 ['MlAvrL567_A', 'Q6R661#PHI:532'],
 ['MlAvrP123', 'Q2MV43#PHI:537'],
 ['MlAvrP4', 'Q2MV49#PHI:536'],
 ['MlAvrM', 'Q2MV46#PHI:535'],
 ['MpNEP1_2', 'A5H7H0#PHI:3128'],
 ['CfEcp1', 'Q00364#PHI:70'],
 ['CfAVR4',
  'Q00363#PHI:18__PHI:5476__PHI:5487__PHI:5546__PHI:5556__PHI:5561__PHI:5567__PHI:5584'],
 ['CfAVR2',
  'Q8NID8#PHI:472__PHI:2344__PHI:5496__PHI:5541__PHI:5555__PHI:6285'],
 ['CfAVR9',
  'P22287#PHI:7__PHI:2845__PHI:5473__PHI:5497__PHI:5542__PHI:5545__PHI:5558__PHI:5566'],
 ['CfAvr5', 'A0A023UJQ9#PHI:5538__PHI:5557'],
 ['CfEcp2', 'Q00365#PHI:71__PHI:5486__PHI:5544__PHI:5559'],
 ['CfAVR4E', 'Q5VB04#PHI:529'],
 ['CfEcp6', 'B3VBK9#PHI:5495__PHI:5543__PHI:5576'],
 ['PnTox3', 'C5IAW5#PHI:2264'],
 ['PnToxA', 'Q0TVA4#PHI:1081'],
 ['PtrToxA', 'P78737#PHI:534__PHI:8380'],
 ['RsNIP1', 'Q02039#PHI:38__PHI:2698'],
 ['RsNIP2', 'I1VGT7#PHI:2696'],
 ['RsNIP3', 'I1VGT8#PHI:2697'],
 ['SsSSITL', 'A7F952#PHI:4482'],
 ['UmCmu1', 'A0A0D1DWQ2#PHI:8816', 'Q4P2D2#PHI:2234'],
 ['UmPep1', 'G0X7E8#PHI:3223'],
 ['ALVi.1389.5', 'ALVi.Vi1.5'],
 ['VdAve1', 'H9DUR1#PHI:2331__PHI:7988'],
 ['VdSCP7', 'G2X9W2#PHI:7129'],
 ['VdPevD1', 'G2WWV6#PHI:8520'],
 ['Zt103264', 'F9X3Q1#PHI:6547'],
 ['Mg1LysM', 'F9XHX3#PHI:6495'],
 ['Mg3LysM', 'F9XMT4#PHI:6494'],
 ['HaATR39_1', 'H9BPR8#PHI:4771'],
 ['HaATR5', 'E6YE64#PHI:4735'],
 ['HpATR13_1', 'Q5G7L3#PHI:328'],
 ['HpATR13_2', 'Q5G7K8#PHI:2402__PHI:2410__PHI:2423__PHI:4754'],
 ['HpATR13_3', 'Q5G7L2#PHI:2422'],
 ['HxATR1NdWsB_1',
  'Q4VKJ6#PHI:531__PHI:2420__PHI:4253__PHI:4254__PHI:4255__PHI:4256__PHI:4257'],
 ['HxATR1NdWsB_2', 'Q4VKJ1#PHI:2421'],
 ['PcF', 'Q94FS7#PHI:665'],
 ['PiAvr1', 'D0NVB5#PHI:5113'],
 ['PiAvr2_2', 'D0NDJ8#PHI:5088'],
 ['PiAvr3A', 'Q572D3#PHI:473__PHI:4958__PHI:6374'],
 ['PiAvr3b', 'D0NXM3#PHI:4206'],
 ['PiAvr4', 'B1NNT7#PHI:5098'],
 ['PiAvrblb2', 'D0P1B2#PHI:4207'],
 ['Picrn1', 'Q8H6Z6#PHI:656'],
 ['Picrn2', 'Q8H6Z4#PHI:657'],
 ['PiEPI1', 'Q6PQH2#PHI:4251'],
 ['PiEpiC1', 'D0NBV1#PHI:6286'],
 ['PiEpiC2B', 'D0NBV3#PHI:6287'],
 ['PiINF1', 'Q01905#PHI:111__PHI:760'],
 ['PiINF2A', 'O42718#PHI:762'],
 ['PiINF2B', 'O42719#PHI:764'],
 ['PITG_03192', 'D0MZL5#PHI:6301'],
 ['PpCBEL', 'O42830#PHI:660'],
 ['PpNPP1', 'Q9AT28#PHI:666'],
 ['PsAvh18a1', 'PsPSR1'],
 ['PsAvh241', 'E0W4Y2#PHI:2727'],
 ['PsAvh5', 'E0W547#PHI:2692'],
 ['PsAvr1a', 'A4L9T6#PHI:5107'],
 ['PsAVR1B_1', 'G4ZRQ8#PHI:2891', 'Q8L552#PHI:530'],
 ['PsAvr1d', 'L7WWG5#PHI:2941__PHI:2943'],
 ['PsAvr1k', 'B2C6F5#PHI:2890'],
 ['PsAVR3a', 'A5YTY8#PHI:5085'],
 ['PsAvr3b_1', 'F8UNP0#PHI:5048'],
 ['PsAvr3c', 'C0LF23#PHI:5112'],
 ['PsAvr4/6', 'A7L812#PHI:5101'],
 ['PsGIP1', 'Q945U0#PHI:652'],
 ['PsGIP2', 'Q945T9#PHI:653'],
 ['PsojNIP', 'Q9AXL8#PHI:658'],
 ['PsPSR2', 'G5ADB3#PHI:3353'],
 ['PaSD21-1', 'Q9SPD4#PHI:664'],
 ['A0A034UTT8#PHI:4481', 'D4HWX6#PHI:2501'],
 ['A0A0H2Z644#PHI:6705', 'A1AJK4#PHI:3789'],
 ['A0A0H2Z8S3#PHI:7290', 'P33883#PHI:6997'],
 ['A0A0H2Z901#PHI:7291',
  'P25084#PHI:5183__PHI:7655__PHI:8640__PHI:8641__PHI:8821'],
 ['A0A0H2ZEG8#PHI:7289',
  'P54292#PHI:6748__PHI:6996__PHI:7656__PHI:8547__PHI:8822'],
 ['A0A0H3NKV1#PHI:6965',
  'E1WFQ7#PHI:4498',
  'Q7CQM5#PHI:2677',
  'Q8XFU4#PHI:554'],
 ['A0A0J9WWX0#PHI:8986', 'B2SME4#PHI:2730'],
 ['A0A0K0GNX3#PHI:9000', 'Q5GWK1#PHI:8032'],
 ['A0A0T9V9T3#PHI:7064', 'P52616#PHI:7448__PHI:8373'],
 ['A0A0U1G333#PHI:7061__PHI:8755', 'P07637#PHI:2624__PHI:6460__PHI:6586'],
 ['A0A0U1JBY1#PHI:8759', 'Q7CPD5#PHI:8726'],
 ['A0A125YVB8#PHI:6693', 'O00933#PHI:4049'],
 ['A0A1D8PMK1#PHI:6837', 'Q5A1D3#PHI:107__PHI:7663'],
 ['A0A1D8PTM1#PHI:6801__PHI:6805', 'Q59X67#PHI:3505__PHI:6671'],
 ['A0A380PJQ0#PHI:8888',
  'A0A384KVC5#PHI:8887',
  'A0A386NHW9#PHI:8853',
  'A0A3N9JER8#PHI:8777'],
 ['A5A9S7#PHI:5400__PHI:8020', 'R4UG28#PHI:5348'],
 ['A9JX05#PHI:4599', 'P0C7Y0#PHI:6666'],
 ['B0BES1#PHI:1023__PHI:3444', 'M7UB04#PHI:3443'],
 ['B0XR79#PHI:4678', 'Q8TFX6#PHI:354'],
 ['B0XW64#PHI:7333', 'E9QYP0#PHI:377__PHI:486'],
 ['B0Y4S0#PHI:4677', 'Q4WQK8#PHI:6111'],
 ['B5G4W2#PHI:2262', 'Q0UI05#PHI:7001'],
 ['C1L2I8#PHI:3344', 'L8DSR3#PHI:7977'],
 ['C3LQZ4#PHI:4166', 'Q9S3S3#PHI:709'],
 ['D0ZHS7#PHI:3745', 'P0A2N3#PHI:4518', 'Q71RX8#PHI:6852'],
 ['D0ZV89#PHI:4899__PHI:6318__PHI:7356',
  'P0DM80#PHI:2675__PHI:6592',
  'P14147#PHI:3725'],
 ['D0ZV90#PHI:6110', 'P0DM78#PHI:2674__PHI:6591'],
 ['D0ZWU0#PHI:2621__PHI:6966__PHI:8303', 'P74856#PHI:8727'],
 ['D4F368#PHI:4148', 'M0QAC7#PHI:4982'],
 ['D4HVM9#PHI:5281', 'D4IAW2#PHI:2456__PHI:3672__PHI:3678'],
 ['F5HEA9#PHI:448', 'Q2TLM7#PHI:2268', 'Q6R3Q3#PHI:736', 'Q96X31#PHI:230'],
 ['F5HHD5#PHI:2266', 'Q6Y392#PHI:1085'],
 ['G3XDB0#PHI:4715', 'Q51380#PHI:4900'],
 ['G4MPQ7#PHI:2207', 'Q8NJ73#PHI:2043'],
 ['G4MSZ4#PHI:8893', 'Q2LD94#PHI:2036__PHI:2192'],
 ['G4MY14#PHI:7734', 'Q9HFU3#PHI:249'],
 ['G4N3L5#PHI:2122__PHI:2132__PHI:2164__PHI:2187', 'Q96UQ9#PHI:268'],
 ['G4NBI6#PHI:2033', 'Q0PND8#PHI:593'],
 ['G4NEB4#PHI:3173', 'Q1HGK2#PHI:1058'],
 ['G4NF05#PHI:776__PHI:802__PHI:2053', 'Q4R1B9#PHI:2065'],
 ['G4NHF4#PHI:2172', 'Q875L7#PHI:322__PHI:1064__PHI:7226'],
 ['I1RFC6#PHI:1321', 'Q2VLJ0#PHI:715'],
 ['I1RNF9#PHI:1012', 'Q96VA7#PHI:251'],
 ['I1S0S6#PHI:1196', 'Q8TG19#PHI:266'],
 ['P0A1I3#PHI:6464__PHI:6588', 'P0A1I4#PHI:647'],
 ['P0A2T6#PHI:2684', 'W9BB63#PHI:6616'],
 ['P0C1U6#PHI:3305', 'Q7A1N5#PHI:6477'],
 ['P0CT48#PHI:567', 'P0CT49#PHI:2213'],
 ['P0CY28#PHI:73', 'P0CY29#PHI:6785__PHI:6791__PHI:6813'],
 ['P22262#PHI:7470__PHI:7898',
  'Q4TVQ0#PHI:3345__PHI:3653__PHI:4151__PHI:6067__PHI:6551'],
 ['Q01928#PHI:7275', 'Q6Q475#PHI:39'],
 ['Q2FIT5#PHI:4224__PHI:4683', 'Q2G2U1#PHI:4655__PHI:8781'],
 ['Q2YKK7#PHI:3306', 'Q8YC53#PHI:3169'],
 ['Q7Z8D0#PHI:2346', 'Q8PHQ1#PHI:4028'],
 ['Q99YP4#PHI:3981', 'W0S3I8#PHI:4995']]
```

In [8]:

```
l = []

for chk, ids in sequences_checksums.items():
    for id_ in ids:
        l.append({"effector": id_, "checksum": chk})

sequences_checksums_df = pd.DataFrame(l)
del l

sequences_checksums_df["effector_database"] = sequences_checksums_df["effector"].apply(lambda x: "phibase" if "#PHI:" in x else "custom")
sequences_checksums_df
```

Out[8]:

|  | effector | checksum | effector\_database |
| --- | --- | --- | --- |
| 0 | PsAvrRpm1 | FM7BWC3nuHjDWZnK9THzRCXW4jM | custom |
| 1 | Q4ZYH0#PHI:3495\_\_PHI:5385\_\_PHI:5501 | FM7BWC3nuHjDWZnK9THzRCXW4jM | phibase |
| 2 | PsAvrRps4 | GaxJoKBEQnM+SQX2IIf6Vw8oZd0 | custom |
| 3 | Q52432#PHI:978\_\_PHI:8026 | GaxJoKBEQnM+SQX2IIf6Vw8oZd0 | phibase |
| 4 | BhNEP1 | zK1n8zy7kkZLAhzWVWBASc6erFM | custom |
| ... | ... | ... | ... |
| 6593 | X5FD66#PHI:4525 | GS7DGLfbkR9D7GpArSbvEo7mhOY | phibase |
| 6594 | X5FDR6#PHI:4558 | lPsfiENv/zkYGVflV69UGG44aB0 | phibase |
| 6595 | X5FDX7#PHI:8868 | t6GpMZjE19G00gCajmsexT855Kc | phibase |
| 6596 | X5IFG8#PHI:3025 | O0KEu96FphlqX7M+QB/hpbu7JwQ | phibase |
| 6597 | X8AYS1#PHI:3273 | hyyuWpSRU3ycXKggOfLyqSGs8fI | phibase |

6598 rows × 3 columns

Now we need to merge the checksum and search matches with the full pc records.

In [9]:

```
nr = pd.read_csv("data/nr.tsv", sep="\t", names=["pc", "checksum", "source", "seqid"])
nr = nr[["pc", "checksum"]]
nr.rename(columns={"pc": "unique_sequence_id"}, inplace=True)
nr.drop_duplicates()
nr.head()
```

Out[9]:

|  | unique\_sequence\_id | checksum |
| --- | --- | --- |
| 0 | PC\_000001 | 9BFQ0A8R/vxQldEexlL1S3OfkuA |
| 1 | PC\_000002 | NWI6881srhnUTxH0SLBlWZ42KM4 |
| 2 | PC\_000003 | HWUOPxTlipI83M3rTzMT3ZeUsVA |
| 3 | PC\_000004 | 9YUGeDuQAqE7ll9beWyXyHOUPUk |
| 4 | PC\_000005 | S0x4Y/J198jtye5aJdnM9zave5M |

Merge the checksum matches.

In [10]:

```
#nr_checksum = nr[nr.checksum.isin(sequences_checksums)]
nr_checksum = pd.merge(nr, sequences_checksums_df, on="checksum", how="inner")
nr_checksum
```

Out[10]:

|  | unique\_sequence\_id | checksum | effector | effector\_database |
| --- | --- | --- | --- | --- |
| 0 | PC\_00073G | YP7YZaxMWMQ+jLwrCi4/DSt2xR8 | A0A098DTB5#PHI:5802 | phibase |
| 1 | PC\_00073G | YP7YZaxMWMQ+jLwrCi4/DSt2xR8 | A0A098DTB5#PHI:5802 | phibase |
| 2 | PC\_0009RR | s+m6ykbQ4wJFWe2HKXv+jcEg+9A | A0A016PUA3#PHI:3659 | phibase |
| 3 | PC\_0009RR | s+m6ykbQ4wJFWe2HKXv+jcEg+9A | A0A016PUA3#PHI:3659 | phibase |
| 4 | PC\_000D77 | WFTwtyMe0Gp2K0UScEJLkrt+m5s | J9VUJ5#PHI:8018 | phibase |
| ... | ... | ... | ... | ... |
| 9142 | PC\_08GZ79 | eH0celjFivfK/oDrQASeUAAi5kc | A0A0H3JWX3#PHI:6876 | phibase |
| 9143 | PC\_08GZ8H | Vt6rhENKYszrdbk5Q1DJQ35xt7c | A0A0H2XGW2#PHI:7351 | phibase |
| 9144 | PC\_08GZB3 | wyonIdgfqQ0uyy8IlvNo/Aul8us | T1YC56#PHI:8558 | phibase |
| 9145 | PC\_08GZDN | 3iA5OAAZPWffX+kTnoqvGh5Azao | Q2G282#PHI:4991 | phibase |
| 9146 | PC\_08GZEJ | /ZS/2ecS9U2JGH0259ZsDFl1tZ8 | Q2FFI7#PHI:7345 | phibase |

9147 rows × 4 columns

In [11]:

```
list(nr_checksum.loc[nr_checksum["effector_database"] == "custom", "effector"].unique())
```

Out[11]:

```
['BgtE_20025',
 'UmCmu1',
 'MoCDIP3',
 'UmTin2',
 'CfEcp5',
 'MoAvrPib',
 'FolSix3',
 'MoSPD5',
 'PgShr9',
 'PgShr8',
 'CCE26798.1',
 'EXF72942.1',
 'KEQ81621.1',
 'KEQ67658.1',
 'MGG_10120',
 'XP_001800520.1',
 'PsShr1',
 'EUN25607.1',
 'MoAvrPi9',
 'BgtE_5665',
 'MGG_14793',
 'MoBas107',
 'FolSix1',
 'Mg1LysM',
 'BghCSEP0055',
 'PsShr2',
 'M.TH16.EuGene_00101881',
 'FGL1',
 'EUC36307.1',
 'KJY01809.1',
 'PGTG_10537.2',
 'UmSee1',
 'BghBEC4',
 'PnTox3',
 'LmAvrLm4_7',
 'CfEcp2',
 'M.BR29.EuGene_00119511',
 'PGTAUSPE_10_1',
 'MGG_18019',
 'EKG15312.1',
 'BgtE_5664',
 'ZtNIP2',
 'Mg3LysM',
 'bgh06532_CSEP0264_BEC1011',
 'bghG008575000001001_CSEP0486',
 'bgh01362_CSEP0027',
 'FolSix13',
 'ALVi.Vi1.15',
 'MoAvr1_CO39',
 'MlAvrL567_A',
 'MoPwl4',
 'MoBas1',
 'XP003298007.1',
 'M.TH16.EuGene_00135161',
 'Fom_2',
 'FOMG_19011',
 'XP_013344128.1',
 'ALVi.Vi1.18',
 'RiSIS1',
 'MGG_16175',
 'VdSCP7',
 'UmPep1',
 'UmPit2',
 'MGG_16113',
 'FolSix4',
 'SsSSITL',
 'MoAVR_Pita',
 'FolSix10',
 'Vdlsc1',
 'ChToxA',
 'CfEcp4',
 'MlAvrP4',
 'XP_001935864.1',
 'MGG_14834',
 'BghAvr_a1',
 'PgtAvrSr35',
 'CbNip1',
 'PnToxA',
 'MpNEP1_1',
 'AKM21218.1',
 'MpNEP1_2',
 'MoCDIP2',
 'MoSPD10',
 'CfAVR4E',
 'MlAvrL567_D',
 'RsNIP3',
 'EME45057.1',
 'bghG002872000001001_CSEP0374',
 'BgtE_20090',
 'BgtAVRk1',
 'CCE26799.1',
 'FolSix6',
 'MGG_08482',
 'BgtAvrPm2',
 'MoBas4',
 'M.TH16.EuGene_00079311',
 'ALVi.Vi1.10',
 'SrSAD1',
 'Cgfl',
 'MoSPD9',
 'M.TH16.EuGene_00134971',
 'bgh02875_CSEP0065',
 'Umeff1-1',
 'CfEcp1',
 'MoIug6',
 'FoeNEP1',
 'M.TH16.EuGene_00106621',
 'UfRTP1',
 'ALVi.Vi1.21',
 'KNG46663.1',
 'MoAVR_Pii',
 'ALVi.1389.5',
 'ALVi.Vi1.5',
 'OAL46779.1',
 'BgtE_5842',
 'PsShr7',
 'MoAVR_Pik',
 'BghCSEP0105',
 'XP_014077357.1',
 'VdNEP',
 'LmAvrLm6',
 'M.TH16.EuGene_00034081',
 'LmAvrLm3',
 'PiFGB1',
 'MlAvrL567_C',
 'XP_013431971.1',
 'XP_007686215.1',
 'MGG_16058',
 'CfTom1',
 'PtrToxA',
 'PsShr3',
 'BghAvr_a13',
 'MoPwl1',
 'M.TH16.EuGene_00000541',
 'XP_018034946.1',
 'PsShr4',
 'PsShr6',
 'FolSix14',
 'BgtAcSP_30091',
 'BgtE_5843',
 'MoPwl3',
 'MGG_04384',
 'MlAvrP123',
 'MoNIS1',
 'bghG001947000001001_CSEP0340',
 'CoDN3',
 'LmAvrLm1',
 'MgxLysM',
 'MGG_08944',
 'FolSix9',
 'SrzPit2',
 'BgtE_10117',
 'EUC44184.1',
 'FolSix11',
 'BgtSvrPm3a1f1',
 'CgDN3',
 'Zt103264',
 'UhAvr1',
 'bgh03782_CSEP0152',
 'FolSix2',
 'BgtE_5901',
 'MIAvrM14',
 'MoBas2',
 'BgtE_20000',
 'ALVi.Vi1.3',
 'FolSix7',
 'CfAvr5',
 'CfEcp6',
 'BghCSEP0162',
 'SsSSVP1',
 'M.BR29.EuGene_00126081',
 'M.BR29.EuGene_00087671',
 'BgtAvrPm3a2f2',
 'ALVi.Vi1.20',
 'BgtE_10124',
 'MoBas3',
 'MoPwl2',
 'MoMSP1',
 'MGG_16619',
 'VdAve1',
 'MGG_17132',
 'SrrPit2',
 'MlpCTP1',
 'MoSPD2',
 'M.BR29.EuGene_00121691',
 'PtrToxB',
 'MoSPD7',
 'MoSlp1',
 'XP_016761200.1',
 'LmAvrLm11',
 'M.BR29.EuGene_00060181',
 'MoCDIP4',
 'RsNIP1',
 'M.BR29.EuGene_00106461',
 'LmAvrLm2',
 'Mycgr3G38105',
 'MoCDIP1',
 'FolSix8',
 'PGTG_16791',
 'CfAVR2',
 'MlAvrM',
 'CfAVR4',
 'ChEC13',
 'MoHEG13',
 'MGG_15207',
 'MoAvrPiz_t',
 'XP_007580207.1',
 'MGG_18060',
 'PsShr5',
 'ChNIS1',
 'M.TH16.EuGene_00124981',
 'MGG_00821',
 'PgtAvrSr50',
 'BgtAVRa10',
 'RsNIP2',
 'MoMC69',
 'RiCRN1',
 'MoIug9',
 'MlAvrL2',
 'BghBEC1054',
 'CoNIS1',
 'MoBAS162',
 'bgh01363_CSEP0028',
 'PpCSEP-07',
 'MoAVR_Pia',
 'PIIN_08944',
 'MoAVR_Pita2',
 'BghBEC3',
 'MiSSP7_2',
 'PnTox1',
 'MGG_17255',
 'GiSP7',
 'CgEP1',
 'ALVi.Vi1.1',
 'PpCSEP-09',
 'XP_007678837.1',
 'CfAVR9',
 'bgh02877_CSEP0066',
 'LmAvrLmJ1',
 'MoSPD4',
 'MGG_15459',
 'MlAvrL567_B',
 'ZtAvrStb6',
 'FolSix5',
 'BfNep1',
 'M.BR29.EuGene_00113041',
 'MoCDIP5',
 'VdPevD1',
 'bghG002857000001001_CSEP0371',
 'XP_007699156.1',
 'BgtE_20026',
 'BgtE_5846',
 'XP_008025738.1',
 'KXT12958.1',
 'ALVi.Vi1.6',
 'ALVi.Vi1.7',
 'OCK93132.1',
 'M.BR29.EuGene_00004921',
 'M.BR29.EuGene_00041131',
 'M.BR29.EuGene_00043011',
 'M.BR29.EuGene_00081821',
 'M.BR29.EuGene_00082031',
 'M.BR29.EuGene_00085071',
 'M.BR29.EuGene_00088411',
 'M.BR29.EuGene_00091361',
 'M.BR29.EuGene_00091681',
 'M.BR29.EuGene_00095641',
 'M.BR29.EuGene_00107481',
 'M.BR29.EuGene_00112111',
 'M.BR29.EuGene_00118801',
 'M.BR29.EuGene_00119491',
 'M.BR29.EuGene_00125811',
 'ALVi.Vi1.13',
 'ALVi.Vi1.16',
 'M.TH16.EuGene_00040131',
 'M.TH16.EuGene_00045871',
 'M.TH16.EuGene_00127871',
 'M.TH16.EuGene_00027411',
 'M.TH16.EuGene_00099371',
 'M.TH16.EuGene_00027191',
 'M.TH16.EuGene_00079081',
 'M.TH16.EuGene_00120731',
 'ALVi.Vi1.4',
 'ALVi.Vi1.22',
 'ALVp_11032.7',
 'ALVp_11032.3',
 'ALVp_11032.1',
 'ALVp_11032.12',
 'ALVp_11032.4',
 'ALVp_11032.14',
 'ALVp_11032.15',
 'ALVp_11032.5',
 'ALVp_11032.6',
 'ALVp_11032.13']
```

In [12]:

```
# Matches
nr_matches = pd.merge(
    nr,
    matches[~matches["effector"].isin(set(nr_checksum["effector"]))],
    on="unique_sequence_id",
    how="inner"
)
nr_matches
```

Out[12]:

|  | unique\_sequence\_id | checksum | effector | effector\_database |
| --- | --- | --- | --- | --- |
| 0 | PC\_002TKG | bR9uKwyVZyBlBB44mIeGgt37VCM | P69996#PHI:3145 | phibase |
| 1 | PC\_0050P7 | GuXToX6jmjpRSp5HQ2YJsdrhmB0 | Q50604#PHI:3923 | phibase |
| 2 | PC\_0094VV | 3D7GhImn0FEluk9G+R6swCCwwmg | ALVi.Vi1.8 | custom |
| 3 | PC\_0094VV | 3D7GhImn0FEluk9G+R6swCCwwmg | ALVi.Vi1.8 | custom |
| 4 | PC\_0094VV | 3D7GhImn0FEluk9G+R6swCCwwmg | ALVi.Vi1.8 | custom |
| ... | ... | ... | ... | ... |
| 185 | PC\_08GZB0 | 1BEWGbVrIBkWfDprm9Tsp0yMlJo | P99075#PHI:6903 | phibase |
| 186 | PC\_08GZBD | SqCX2VF3MNUMOpVsRZ4BGrlMcgM | A8YY71#PHI:8019 | phibase |
| 187 | PC\_08GZC8 | zwz4yog+WzMmzUyEBTRN6RFJQPU | A0A0H3JLK2#PHI:4960 | phibase |
| 188 | PC\_08GZEO | /01HZvinFMgL0d0hX54HMuSQhUc | Q6GFE2#PHI:3285 | phibase |
| 189 | PC\_08JJ6L | YuA+BA7KnRq18fChmFNT6z6udZ8 | G4MW47#PHI:2049 | phibase |

190 rows × 4 columns

In [13]:

```
nr_matches["unique_sequence_id"].nunique()
```

Out[13]:

```
161
```

There are a few proteins in our query dataset that aren't matched by the checksum.

In [14]:

```
nr_merged = pd.concat([nr_checksum, nr_matches], ignore_index=True)
nr_merged.drop_duplicates(inplace=True)
```

Are there any effectors that we don't match?

In [15]:

```
len(sequences.keys())
```

Out[15]:

```
6598
```

In [16]:

```
got = set(nr_merged["effector"].unique())
missing = [s for s in sequences.keys() if (not s in got) and ("#PHI" not in s)]
missing
```

Out[16]:

```
['PsAvrRpm1',
 'PsAvrRps4',
 'BhNEP1',
 'PaNPP1',
 'VpNPP1',
 'PsAvrA',
 'PsAvrB1',
 'PsAvrB2',
 'PsAvrD',
 'PsAvrE1',
 'PsAvrE2',
 'PsAvrE3',
 'PsAvrPto1',
 'PsAvrPto2',
 'PsAvrRpt2',
 'PsHopA',
 'PsHopB',
 'PsHopC',
 'PsHopD1',
 'PsHopD2',
 'PsHopE',
 'PsHopF1',
 'PsHopF2',
 'PsHopG1',
 'PsHopG2',
 'PsHopH1',
 'PsHopH2',
 'PsHopI1',
 'PsHopI2',
 'PsHopI3',
 'PsHopK',
 'PsHopM1',
 'PsHopM2',
 'PsHopN',
 'PsHopO',
 'PsHopQ1',
 'PsHopQ2',
 'PsHopR1',
 'PsHopR2',
 'PsHopS',
 'PsHopT',
 'PsHopU',
 'PsHopV1',
 'PsHopV2',
 'PsHopW',
 'PsHopX1',
 'PsHopX2',
 'PsHopY',
 'PsHopZ',
 'PsHopAA1',
 'PsHopAA2',
 'PsHopAB1',
 'PsHopAB2',
 'PsHopAB3',
 'PsHopAD',
 'PsHopAE1',
 'PsHopAE2',
 'PsHopAF1',
 'PsHopAF2',
 'PsHopAF3',
 'PsHopAG',
 'PsHopAH1',
 'PsHopAH2',
 'PsHopAI',
 'PsHopAL',
 'PsHopAM',
 'PsHopAO',
 'PsHopAQ',
 'PsHopAR',
 'PsHopAS',
 'PsHopAT',
 'PsHopAU',
 'PsHopAV',
 'PsHopAW',
 'PsHopAX',
 'PsHopAY',
 'PsHopAZ',
 'PsHopBA',
 'PsHopBB',
 'PsHopBC',
 'PsHopBD',
 'PsHopBE',
 'PsHopBF',
 'PsHopBG',
 'RsAvrA',
 'RsGALA',
 'RsHLK1',
 'RsPopA',
 'RsPopB',
 'RsPopC',
 'RsPopP',
 'RsPopW',
 'RsRipA',
 'RsRipB',
 'RsRipT',
 'RsSkwp1',
 'RsTAL',
 'RSp0304',
 'RSp1239',
 'RSc3369',
 'RSp0572',
 'RSp0842',
 'RSp1601',
 'RSp0323',
 'RSp1281',
 'RRSL_00326_1',
 'RRSL_00326_2',
 'XAvrBs1_1',
 'XAvrBs1_2',
 'XAvrBs2_1',
 'XAvrBs2_2',
 'XAvrBs2_3',
 'XTAL',
 'XHpaA',
 'XopB',
 'XopC1',
 'XopC2',
 'XopD',
 'XopE1',
 'XopE2',
 'XopF1',
 'XopF2',
 'XopG1',
 'XopG2',
 'XopH',
 'XopJ1',
 'XopJ2',
 'XopK1',
 'XopK2',
 'XopK3',
 'XopL1',
 'XopL2',
 'XopL3',
 'XopN1',
 'XopN2',
 'XopN3',
 'XopO',
 'XopP1',
 'XopP2',
 'XopP3',
 'XopQ1',
 'XopQ2',
 'XopQ3',
 'XopR1',
 'XopR2',
 'XopR3',
 'XopT',
 'XopU',
 'XopV1',
 'XopV2',
 'XopW',
 'XopX1',
 'XopX2',
 'XopX3',
 'XopY',
 'XopZ1',
 'XopZ2',
 'XopZ3',
 'XopAA1',
 'XopAA2',
 'XopAB',
 'XopAC',
 'XopAD',
 'XopAE',
 'XopAF',
 'XopAH',
 'XopAI',
 'XopAJ',
 'XopAL',
 'XopAM',
 'SeAvrA',
 'SeGogB',
 'SePipB',
 'SeSifA',
 'SeSifB',
 'SeSipA',
 'SeSipB',
 'SeSipC',
 'SeSipD',
 'SeSlrP',
 'SeSopA',
 'SeSopB',
 'SeSopD',
 'SeSopE',
 'SeSpiC',
 'SeSptP',
 'SeSpvB',
 'SeSpvC',
 'SeSseF',
 'SeSseG',
 'SeSseI',
 'SeSseJ',
 'SeSseK1',
 'SeSspH1',
 'SeSteA',
 'SeSteB',
 'SeSteC',
 'BghROPIP1',
 'FolSix12',
 'ALVi.1389.16',
 'ALVi.1389.1',
 'ALVi.1389.8',
 'ALVi.1389.15',
 'ALVi.1389.9',
 'ALVi.1389.10',
 'ALVi.1389.14',
 'ALVi.1389.13',
 'ALVi.Vi1.12',
 'ALVi.Vi1.11',
 'ALVi.Vi1.14',
 'ALVi.Vi1.2',
 'ALVi.Vi1.24',
 'ALVi.Vi1.23',
 'ALVp_11032.11',
 'ALVp_11032.8',
 'ALVp_11032.16',
 'ALVp_11032.2',
 'ALVp_11032.9',
 'ALVp_11032.10',
 'Cri_9402',
 'HaATR39_1',
 'HaATR5',
 'HaRxL44',
 'HpATR13_1',
 'HpATR13_2',
 'HpATR13_3',
 'HxATR1NdWsB_1',
 'HxATR1NdWsB_2',
 'HxATR1NdWsB_3',
 'PcF',
 'PiAvr1',
 'PiAvr2_1',
 'PiAvr2_2',
 'PiAvr3A',
 'PiAvr3b',
 'PiAvr4',
 'PiAvrblb1',
 'PiAvrblb2',
 'PiAvrVnt1',
 'Picrn1',
 'PiCRN15',
 'PiCRN16',
 'Picrn2',
 'PiCRN8',
 'PiEPI1',
 'PiEPI2',
 'PiEpiC1',
 'PiEPIC2A',
 'PiEpiC2B',
 'PiINF1',
 'PiINF2A',
 'PiINF2B',
 'PITG_03192',
 'PiNPP1',
 'PiPexRD2',
 'PiPexRD54',
 'PexRD2',
 'PpPSE1',
 'PpCBEL',
 'PpNPP1',
 'PsAvh18a1',
 'PsAvh241',
 'PsAvh5',
 'PsAvr1a',
 'PsAVR1B_1',
 'PsAvr1d',
 'PsAvr1k',
 'PsAVR3a',
 'PsAvr3b_1',
 'PsAvr3b_2',
 'PsAvr3c',
 'PsAvr4/6',
 'PsCRN115',
 'PsCRN63',
 'PsGIP1',
 'PsGIP2',
 'Pslsc1',
 'PsojNIP',
 'PsPSR1',
 'PsPSR2',
 'PaSD21-1']
```

Most of the missing effector sequences are non-fungal.
I did spot a few fungal effectors that are missing.

I need to find out exactly which ones are missing.

In [17]:

```
effector_table = pd.read_csv("data/pclust_cluster_selection-custom_effector_sequences.tsv", sep="\t")
effector_table = effector_table[effector_table["Validated"].notnull()]
effector_table["checksum"] = effector_table["Sequence"].apply(tab_to_checksum)
effector_table.head()
```

Out[17]:

|  | ID | Kingdom | Validated | ApoCyto | Uniprot | Genbank | pdbID | Name | Species | Taxid | Notes | Reference | ReferenceLink | Sequence | checksum |
| --- | --- | --- | --- | --- | --- | --- | --- | --- | --- | --- | --- | --- | --- | --- | --- |
| 0 | KEQ67658.1 | Fungal | no | cytoplasmic | A0A074W4A2 | KEQ67658.1 | NaN | Zt6-like | Aureobasidium melanogenum | 46634 | guanyl-specific ribonuclease F1 [Aureobasidium... | Graeme J Kettles, Carlos Bayon, Caroline Spark... | http://biorxiv.org/content/early/2017/04/24/13... | MLFNSILATAMLFAASAVALPVELEARQSSTTCGNTYYSASQVSAA... | 4uX6OV/eHmUnzbxTCfagJCPkGBE |
| 1 | XP\_013431971.1 | Fungal | no | cytoplasmic | A0A074XTA9 | XP\_013431971.1 | NaN | Zt6-like | Aureobasidium namibiae | 559561 | guanyl-specific ribonuclease F1 [Aureobasidium... | Graeme J Kettles, Carlos Bayon, Caroline Spark... | http://biorxiv.org/content/early/2017/04/24/13... | MLFKSIFASAVLFAASSIALPTDLEARQQATTCGSTSYSASQVRSA... | RxaZ5VHoFU/U/l7TgMs/GBRDQBs |
| 2 | KEQ81621.1 | Fungal | no | cytoplasmic | A0A074Y426 | KEQ81621.1 | NaN | Zt6-like | Aureobasidium pullulans | 5580 | guanyl-specific ribonuclease F1 [Aureobasidium... | Graeme J Kettles, Carlos Bayon, Caroline Spark... | http://biorxiv.org/content/early/2017/04/24/13... | MLFQSVFTTAVLFVASTIALPTDLESRQSATTCGSTSYTAAQVRSA... | K5SSCOD9BrUC3rANUT4ruAsBCAg |
| 3 | XP\_013344128.1 | Fungal | no | cytoplasmic | A0A074YCY6 | XP\_013344128.1 | NaN | Zt6-like | Aureobasidium subglaciale | 1042127 | hypothetical protein AUEXF2481DRAFT\_29064 [Aur... | Graeme J Kettles, Carlos Bayon, Caroline Spark... | http://biorxiv.org/content/early/2017/04/24/13... | MLFQSIFTTAVLFAASAIALPTDLEARQQATRCGSTSYTAAQVRSA... | M0n7L19OFJJ9rVuWWH1IRDVIs1I |
| 4 | XP\_007678837.1 | Fungal | no | cytoplasmic | M2LHS5 | XP\_007678837.1 | NaN | Zt6-like | Baudoinia panamericana | 1709381 | hypothetical protein BAUCODRAFT\_205433 [Baudoi... | Graeme J Kettles, Carlos Bayon, Caroline Spark... | http://biorxiv.org/content/early/2017/04/24/13... | MRFLFATAAFFAAAAFAFPLTQRQSSTTCGKNRYTTKQVNAALNQG... | EG6LxRYbeJmvaXz8FburXScF+wk |

In [18]:

```
effector_table[(effector_table["Kingdom"] == "Fungal") & effector_table["ID"].isin(missing)]
```

Out[18]:

|  | ID | Kingdom | Validated | ApoCyto | Uniprot | Genbank | pdbID | Name | Species | Taxid | Notes | Reference | ReferenceLink | Sequence | checksum |
| --- | --- | --- | --- | --- | --- | --- | --- | --- | --- | --- | --- | --- | --- | --- | --- |
| 30 | BghROPIP1 | Fungal | yes | Cytoplasmic | NaN | NaN | NaN | ROPIP1 | Blumeria graminis f. sp. hordei | 62688 | Part of a transposon gene. Interacts with barl... | Nottensteiner et al. 2018. J. Exp. Bot | https://academic.oup.com/jxb/article/69/15/374... | MSTPVSGVPYEPPALTVESAEPRLSNNLLTSMRIPSRLRDLYRLHF... | GYHAlrGwesvYbR/lJTmzC6qVzYs |
| 64 | Cri\_9402 | Fungal | heterologous | NaN | NaN | NaN | NaN | Cri-9402 | Cronartium ribicola | 27354 | Suppresses HR upon infection with bacterial ve... | Ma and Liu, 2019. Anal. Theor. Plant. Biol. | https://apsjournals.apsnet.org/doi/10.1094/PHY... | MLLLIIAASLFLNVQANWDPATGYLWDFKPTASWLSQHKPKASSSN... | u0Az6FmxYdt1zZ7KAb0R9synuxY |
| 71 | FolSix12 | Fungal | yes | NaN | M1U296 | AGG54053 | NaN | Six12 | Fusarium oxysporum f. sp. lycopersici | 59765 | proposed non-classical secretion | Schmidt et al, 2013, BMC Genomics, MITEs in th... | http://www.biomedcentral.com/1471-2164/14/119 | MLLQVQPTLAQASSCLSVGPKGISNQNACVCGGQCVMKDLVV | Hi6JyEBkwcik56hcTjmi0nz143I |
| 261 | ALVi.1389.1 | Fungal | no | NaN | NaN | NaN | NaN | ALVi.1389.1 | Venturia inaequalis f. sp. Pyracanthae (Loquat... | 5025 | NaN | Shiller et al. 2015 Frontiers in Plant Science | http://journal.frontiersin.org/article/10.3389... | MKFTLTLLAVASSVSASTYMLCCCTQPTLPQELTDRRYYVDDPKSR... | 3phGnGjwwV/c5xKxKkh7UENLvkA |
| 262 | ALVi.1389.10 | Fungal | no | NaN | NaN | NaN | NaN | ALVi.1389.10 | Venturia inaequalis f. sp. Pyracanthae (Loquat... | 5025 | NaN | Shiller et al. 2015 Frontiers in Plant Science | http://journal.frontiersin.org/article/10.3389... | MKPFQFLLISILAASSASAKKHRLCCCAGFDACGLFSCEKDSTQSV... | vRJxxsy+JK53NfBXgli942DgS/8 |
| 265 | ALVi.1389.13 | Fungal | no | NaN | NaN | NaN | NaN | ALVi.1389.13 | Venturia inaequalis f. sp. Pyracanthae (Loquat... | 5025 | NaN | Shiller et al. 2015 Frontiers in Plant Science | http://journal.frontiersin.org/article/10.3389... | MMLSYSLLFALLAASVSAKKHRLCCCAGFNACNQFVCDDVHTQNLV... | xopL6+trv+jo0ZrqXfU9+o4+F6o |
| 266 | ALVi.1389.14 | Fungal | no | NaN | NaN | NaN | NaN | ALVi.1389.14 | Venturia inaequalis f. sp. Pyracanthae (Loquat... | 5025 | NaN | Shiller et al. 2015 Frontiers in Plant Science | http://journal.frontiersin.org/article/10.3389... | MKSLYPLLVSLLAASVSAHKHRLCCCAGYNACNQFVCDPYSAGWLV... | o67o4kqCJcMol1R88+ikZflcfzM |
| 267 | ALVi.1389.15 | Fungal | no | NaN | NaN | NaN | NaN | ALVi.1389.15 | Venturia inaequalis f. sp. Pyracanthae (Loquat... | 5025 | NaN | Shiller et al. 2015 Frontiers in Plant Science | http://journal.frontiersin.org/article/10.3389... | MKLLFTILTSILAALSVSARKHRLCCCSDFDQCGQYVCDGFATQSI... | PmnEbUeoF40Zggn9nCIMcWtnDto |
| 268 | ALVi.1389.16 | Fungal | no | NaN | NaN | NaN | NaN | ALVi.1389.16 | Venturia inaequalis f. sp. Pyracanthae (Loquat... | 5025 | NaN | Shiller et al. 2015 Frontiers in Plant Science | http://journal.frontiersin.org/article/10.3389... | MKFLYLLLISLLTTSVSAKKHRLCCCAGFNACNQFVCDADITQYLV... | Vb7iBuW95hVcHKmZ6tSe8CjjkaQ |
| 275 | ALVi.1389.8 | Fungal | no | NaN | NaN | NaN | NaN | ALVi.1389.8 | Venturia inaequalis f. sp. Pyracanthae (Loquat... | 5025 | NaN | Shiller et al. 2015 Frontiers in Plant Science | http://journal.frontiersin.org/article/10.3389... | MKLALSLLAASLATSVSAKAYELCCCSNPANPKDLHDSRYFEGLDP... | g1korrWudsYRmvWl82bPMt+8ioY |
| 276 | ALVi.1389.9 | Fungal | no | NaN | NaN | NaN | NaN | ALVi.1389.9 | Venturia inaequalis f. sp. Pyracanthae (Loquat... | 5025 | NaN | Shiller et al. 2015 Frontiers in Plant Science | http://journal.frontiersin.org/article/10.3389... | MKLLYPFLISILVTTVSSKKHRLCCCAGFDQCGLYVCDGFATQTVV... | xWgrzEt31gM0NTIudLJfDTvqr98 |
| 279 | ALVi.Vi1.11 | Fungal | no | NaN | NaN | NaN | NaN | ALVi.Vi1.11 | Venturia inaequalis MNH120/ICMP13258 | 5025 | NaN | Shiller et al. 2015 Frontiers in Plant Science | http://journal.frontiersin.org/article/10.3389... | MKLFYPLLVYLLTTSVSARRHRLCCCAGFNACNQFVCDDKSTQSII... | uzenFNeJcBV78rZ/YHG9pVuU5Ok |
| 280 | ALVi.Vi1.12 | Fungal | no | NaN | NaN | NaN | NaN | ALVi.Vi1.12 | Venturia inaequalis MNH120/ICMP13258 | 5025 | NaN | Shiller et al. 2015 Frontiers in Plant Science | http://journal.frontiersin.org/article/10.3389... | MKATLISAILLTLSAVDAKKHRLCCCYGIDEDAPGKWSDKSAVCVQ... | KaH3EV9T3lGBHEI2qyHpZHCBnvA |
| 282 | ALVi.Vi1.14 | Fungal | no | NaN | NaN | NaN | NaN | ALVi.Vi1.14 | Venturia inaequalis MNH120/ICMP13258 | 5025 | NaN | Shiller et al. 2015 Frontiers in Plant Science | http://journal.frontiersin.org/article/10.3389... | MKLLYLLPVSLLATFVSAAKHLLCCCAGFNACDQFVCDGFSTADIV... | kYTgSVW+++RLVL9VRKVvcF0zb2w |
| 288 | ALVi.Vi1.2 | Fungal | no | NaN | NaN | NaN | NaN | ALVi.Vi1.2 | Venturia inaequalis MNH120/ICMP13258 | 5025 | NaN | Shiller et al. 2015 Frontiers in Plant Science | http://journal.frontiersin.org/article/10.3389... | MKLSYPFLISILAASVSSKTHRLCCCAGFDQCGLYVCDGFATQTVV... | Ihjh4s8fPkeWpI2iOkJXeZCXPuw |
| 292 | ALVi.Vi1.23 | Fungal | no | NaN | NaN | NaN | NaN | ALVi.Vi1.23 | Venturia inaequalis MNH120/ICMP13258 | 5025 | NaN | Shiller et al. 2015 Frontiers in Plant Science | http://journal.frontiersin.org/article/10.3389... | MKLTFTLLAVSLATSVSANSYTLCCCTKPTNIQDLKDPQYWSGTPP... | 2ZiStrWRErlIGv/TOoaMUemWHGM |
| 293 | ALVi.Vi1.24 | Fungal | no | NaN | NaN | NaN | NaN | ALVi.Vi1.24 | Venturia inaequalis MNH120/ICMP13258 | 5025 | NaN | Shiller et al. 2015 Frontiers in Plant Science | http://journal.frontiersin.org/article/10.3389... | MKLTFALLAASLAALVSAKAYQLCCCVKPDEQKIGGRVFQVGTPKC... | SQtNuciV+cEWGVpDIRG/taXfX1w |
| 302 | ALVp\_11032.10 | Fungal | no | NaN | NaN | NaN | NaN | ALVp\_11032.10 | Venturia pirina 11032 | 1437871 | NaN | Shiller et al. 2015 Frontiers in Plant Science | http://journal.frontiersin.org/article/10.3389... | MRLSIALGLLAWLASVSANKHKFCCCWTKEIGCDVSSTQRIINARV... | pDuy6HvlitLbUCl4cR5dXMXdpho |
| 303 | ALVp\_11032.11 | Fungal | no | NaN | NaN | NaN | NaN | ALVp\_11032.11 | Venturia pirina 11032 | 1437871 | NaN | Shiller et al. 2015 Frontiers in Plant Science | http://journal.frontiersin.org/article/10.3389... | MKISIALSLLAYIVSTSAEPHDLCCCSSKKAKKCMNDETKLVMNNH... | GPlwLGVEtSsmhla1PhlCiF/CqmA |
| 308 | ALVp\_11032.16 | Fungal | no | NaN | NaN | NaN | NaN | ALVp\_11032.16 | Venturia pirina 11032 | 1437871 | NaN | Shiller et al. 2015 Frontiers in Plant Science | http://journal.frontiersin.org/article/10.3389... | MKISVALTTTLLAIASVSADKHDFCCCTDPGKTKCLKDETESVRTS... | NOxdk/KX4uGizDRDqGNrnphHk+E |
| 309 | ALVp\_11032.2 | Fungal | no | NaN | NaN | NaN | NaN | ALVp\_11032.2 | Venturia pirina 11032 | 1437871 | NaN | Shiller et al. 2015 Frontiers in Plant Science | http://journal.frontiersin.org/article/10.3389... | MKPAFALLIASFASSASAGRAYDLCCCCNRTTPSAAYTCNDDAGAA... | 1OKnhP+tocJ9oct+qqgUmyj7PAc |
| 315 | ALVp\_11032.8 | Fungal | no | NaN | NaN | NaN | NaN | ALVp\_11032.8 | Venturia pirina 11032 | 1437871 | NaN | Shiller et al. 2015 Frontiers in Plant Science | http://journal.frontiersin.org/article/10.3389... | MKISLAFHLSLLACIASVSADKHALCCCSTKAGCDINSTQQVINER... | adktYoWGtrFvrz3xAwahJVXDx1o |
| 316 | ALVp\_11032.9 | Fungal | no | NaN | NaN | NaN | NaN | ALVp\_11032.9 | Venturia pirina 11032 | 1437871 | NaN | Shiller et al. 2015 Frontiers in Plant Science | http://journal.frontiersin.org/article/10.3389... | MKVSTALSLFACIASISAEKHTYCCCSDKYWNTCDVEATIQVAHEH... | OVongh5zMMNBHGsSFo/iEPC6Pkw |

Ok, so the important ones that are missing are:

- BghROPIP1
- Cri\_9402
- FolSix12

A lot of the AvrLm6 homologs in Venturia are also missing, but that's ok. We've got the real one.

Now I need to merge the selected matches with the clusters.

In [19]:

```
clusters = pd.read_csv("data/profile.tsv", sep="\t")
clusters = pd.merge(clusters, nr_merged, left_on="member", right_on="unique_sequence_id", how="right")
clusters.drop(columns="member", inplace=True)
clusters
```

Out[19]:

|  | cluster | unique\_sequence\_id | checksum | effector | effector\_database |
| --- | --- | --- | --- | --- | --- |
| 0 | PC\_05YS56 | PC\_0462WT | ZYZM7Txwj1OIYzMf3IpXJnUFJH4 | I1RV71#PHI:1812 | phibase |
| 1 | PC\_060UD5 | PC\_04YGYN | xlLgSWAUo2dz407jS1EsvZ/rJCE | BgtAvrPm3a2f2 | custom |
| 2 | PC\_051JP9 | PC\_039U0B | D0sFn6jAlGJf0yj3iQ7nNMH/AwE | I1RMT5#PHI:1294 | phibase |
| 3 | PC\_071Z8Q | PC\_05QGYC | bP5qPrQfvvZedva+tosOoVYBUoU | PGTG\_16791 | custom |
| 4 | PC\_01JT40 | PC\_04SNN2 | VwysmH0hl85rMt/e4C+DxAU5uyc | I1RNY7#PHI:1181 | phibase |
| ... | ... | ... | ... | ... | ... |
| 3879 | PC\_04OICS | PC\_05LVKX | m7d+Sg6InACc/KNXhwGosbazBOY | I1RFP4#PHI:1441 | phibase |
| 3880 | PC\_00NM78 | PC\_05XX6X | 3iNhAMCF7fo678PHwfOzwTHRPQc | I1S3H6#PHI:1858 | phibase |
| 3881 | NaN | PC\_03ZM2Y | 9czEAlhlxnRX0yF5yyamB7ekjK8 | Q4WT66#PHI:2511 | phibase |
| 3882 | NaN | PC\_05DQYK | 3wpmSmrVy1zaSIdCl2tlFfOgy6k | Q6Q8B9#PHI:366 | phibase |
| 3883 | NaN | PC\_06QVYP | mPREs/XLBz4W7J/tg4SFuvmJU0Q | I1SAJ7#PHI:9042 | phibase |

3884 rows × 5 columns

Oh no! There are some sequences with no cluster assigned.
This shouldn't happen.

In [20]:

```
clusters[clusters["cluster"].isnull()]
```

Out[20]:

|  | cluster | unique\_sequence\_id | checksum | effector | effector\_database |
| --- | --- | --- | --- | --- | --- |
| 3881 | NaN | PC\_03ZM2Y | 9czEAlhlxnRX0yF5yyamB7ekjK8 | Q4WT66#PHI:2511 | phibase |
| 3882 | NaN | PC\_05DQYK | 3wpmSmrVy1zaSIdCl2tlFfOgy6k | Q6Q8B9#PHI:366 | phibase |
| 3883 | NaN | PC\_06QVYP | mPREs/XLBz4W7J/tg4SFuvmJU0Q | I1SAJ7#PHI:9042 | phibase |

We filtered some very long and short sequences from the dataset before running the first pass of clustering.
So let's just check what these are.

- PC\_03ZM2Y is super long (6,269AA), so it's ok that it's missing.
- PC\_05DQYK is super short (22 AA), so it's ok that it's missing.
- PC\_06QVYP is super long (11,197 AA).

Everything looks fine, so we'll just leave it.

In [21]:

```
clusters.to_csv("07-find_effector_clusters-effector_clusters.tsv", sep="\t", index=False)
```

Cool.

So I'll just merge the assigned clusters with the original datasets and save it for later use.

In [22]:

```
clusters
```

Out[22]:

|  | cluster | unique\_sequence\_id | checksum | effector | effector\_database |
| --- | --- | --- | --- | --- | --- |
| 0 | PC\_05YS56 | PC\_0462WT | ZYZM7Txwj1OIYzMf3IpXJnUFJH4 | I1RV71#PHI:1812 | phibase |
| 1 | PC\_060UD5 | PC\_04YGYN | xlLgSWAUo2dz407jS1EsvZ/rJCE | BgtAvrPm3a2f2 | custom |
| 2 | PC\_051JP9 | PC\_039U0B | D0sFn6jAlGJf0yj3iQ7nNMH/AwE | I1RMT5#PHI:1294 | phibase |
| 3 | PC\_071Z8Q | PC\_05QGYC | bP5qPrQfvvZedva+tosOoVYBUoU | PGTG\_16791 | custom |
| 4 | PC\_01JT40 | PC\_04SNN2 | VwysmH0hl85rMt/e4C+DxAU5uyc | I1RNY7#PHI:1181 | phibase |
| ... | ... | ... | ... | ... | ... |
| 3879 | PC\_04OICS | PC\_05LVKX | m7d+Sg6InACc/KNXhwGosbazBOY | I1RFP4#PHI:1441 | phibase |
| 3880 | PC\_00NM78 | PC\_05XX6X | 3iNhAMCF7fo678PHwfOzwTHRPQc | I1S3H6#PHI:1858 | phibase |
| 3881 | NaN | PC\_03ZM2Y | 9czEAlhlxnRX0yF5yyamB7ekjK8 | Q4WT66#PHI:2511 | phibase |
| 3882 | NaN | PC\_05DQYK | 3wpmSmrVy1zaSIdCl2tlFfOgy6k | Q6Q8B9#PHI:366 | phibase |
| 3883 | NaN | PC\_06QVYP | mPREs/XLBz4W7J/tg4SFuvmJU0Q | I1SAJ7#PHI:9042 | phibase |

3884 rows × 5 columns

In [ ]:

```

```

In [ ]:

```

```

In [ ]:

```

```

In [ ]:

```

```

In [ ]:

```

```

In [ ]:

```

```

In [ ]:

```

```

In [ ]:

```

```

In [ ]:

```

```

In [ ]:

```
effector_table_clusters = pd.merge(
    (clusters[clusters["effector_database"] == "custom"]
     .drop(columns=["effector_database", "checksum", "seqid", "source"])
     .drop_duplicates()),
    effector_table.rename(columns={"ID": "effector"}),
    on="effector"
)
effector_table_clusters.to_csv("07-find_effector_clusters-effector_clusters_custom.tsv", sep="\t", index=False)

effector_table_clusters.head()
```

In [ ]:

```

```

In [ ]:

```
phibase = pd.read_csv("data/phi-base_v4-8_2019-09-16.csv", low_memory=False)
phibase = phibase[[
    'PHI_MolConn_ID',
    'Protein ID source',
    'Protein ID',
    'Gene ID source',
    'Gene ID',
    'Sequence Strain',
    'Gene',
    'Gene/Protein modification',
    'Modified gene/protein Id',
    'Interacting partner(s)',
    'Interacting partner(s) Id',
    'Multiple mutation',
    'Pathogen ID',
    'Pathogen species',
    'Pathogen strain ID',
    'Pathogen strain',
    'Disease',
    'Host description',
    'Host ID',
    'Host species',
    'Host strain',
    'Host genotype',
    'Host genotype-Id',
    'Tissue',
    'Gene Function',
    'GO annotation',
    'Database',
    'Pathway',
    'Mutant Phenotype',
    'Mating defect',
    'Prepenetration defect',
    'Penetration defect',
    'Postpenetration defect',
    'Disease manifestation',
    'Vegetative spores',
    'Sexual spores',
    'Invitro growth',
    'Spore germination',
    'Essential gene',
    'Gene inducer',
    'Gene inducer Id',
    'Host target',
    'Host target Id',
    'Interaction phenotype',
    'Host response',
    'Exp. Technique-stable',
    'Exp. Technique-transient',
    'Species expert',
    'Entered by',
    'PMID',
    'Ref. Source',
    'DOI',
    'Ref. detail',
    'Author email',
    'Comments',
    'Author reference',
    'Year',
    'Curation details',
    'File name',
    'Batch no.',
    'Curation date',
    'Curator organization',
    'Lab',
    'FG-mycotoxin',
    'AdditionalIdentifierTypeOfGeneLocusID',
    'AdditionalGeneLocusID',
    'Anti infective agent',
    'Anti infective compound',
    'Anti infective-target site',
    'Anti infective group name',
    'Anti infective Chemical group',
    'Anti infective-Mode in planta',
    'Mode of action',
    'FRAC CODE',
    'Anti infective-comments',
    'phi3pars',
    'Horizontal gene transfer',
    'phi3_pars',
    ' Pathogen gene maps',
    'Host gene maps'
]]
```

In [ ]:

```
df = (clusters[clusters["effector_database"] == "phibase"]
 .drop(columns=["effector_database", "checksum", "seqid", "source"])
 .drop_duplicates()
 .copy())

df["Protein ID"] = df["effector"].str.split("#").apply(lambda x: x[0])
phibase_clusters = pd.merge(
    df,
    phibase,
    on="Protein ID",
    how="left"
)
phibase_clusters.to_csv("07-find_effector_clusters-effector_clusters_phibase.tsv", sep="\t", index=False)

phibase_clusters
```

In [ ]:

```
!head data/profile_stats.tsv
```

In [ ]:

```
clusters
```

In [ ]:

```
clusters
```

In [ ]:

```
df = clusters.loc[clusters["pc"].notnull(), ["pc", "effector", "effector_database"]].copy()
df["effector"] = df.apply(lambda r: f"{r['effector_database']}|{r['effector']}", axis=1)
df.drop(columns="effector_database", inplace=True)
df = (
    df
    .drop_duplicates()
    .sort_values(["pc", "effector"])
    .groupby("pc")["effector"]
    .apply(lambda x: ";".join(x))
    .reset_index()
)

profile_stats = pd.read_csv("data/profile_stats.tsv", sep="\t")
target_clusters = set(clusters["cluster"])
profile_stats = profile_stats[profile_stats["query"].isin(target_clusters)]
profile_stats = profile_stats[["query", "target", "evalue", "pident", "qstart", "qend", "qlen", "tstart", "tend", "tlen", "qcov", "tcov"]]
profile_stats.rename(
    columns={
        "query": "cluster",
        "target": "member",
        "qstart": "cluster_start",
        "qend": "cluster_end",
        "qlen": "cluster_length",
        "tstart": "member_start",
        "tend": "member_end",
        "tlen": "member_length",
        "qcov": "cluster_coverage",
        "tcov": "member_coverage",
    },
    inplace=True
)

profile_stats = pd.merge(profile_stats, df.rename(columns={"pc": "member"}), on="member", how="left")
profile_stats.to_csv("07-find_effector_clusters-effector_clusters_alignment_stats.tsv", sep="\t", index=False)
profile_stats.head()
```

In [ ]:

```
profile_stats[profile_stats["effector"].notnull()]
```

## Getting some extra data about the clusters.¶

In [ ]:

```
import csv
```

In [ ]:

```
csv.list_dialects()
```

In [ ]:

```
members_to_get = set(profile_stats["member"])
```

In [ ]:

```
ipg_metadata = []

with open("data/nr_ipg.tsv", newline="") as handle:
    reader = csv.DictReader(handle, dialect="excel-tab")
    for line in reader:
        if line["id"] in members_to_get:
            ipg_metadata.append(line)

ipg_metadata = pd.DataFrame(ipg_metadata)
ipg_metadata = (
    ipg_metadata
    .loc[ipg_metadata["db"] != "NCBIAssembly", ["id", "db", "db_id", "ncbi_taxid", "organism"]]
    .drop_duplicates()
)
ipg_metadata.head()
```

In [ ]:

```
custom_metadata = []

with open("data/nr_custom.tsv", newline="") as handle:
    reader = csv.DictReader(handle, dialect="excel-tab")
    for line in reader:
        if line["id"] in members_to_get:
            custom_metadata.append(line)

custom_metadata = pd.DataFrame(custom_metadata)
custom_metadata = custom_metadata[["id", "db", "db_id", "ncbi_taxid", "organism", "doi"]]
custom_metadata.head()
```

In [ ]:

```
uniparc_xref_metadata = []

with open("data/nr_uniparc_xrefs.tsv", newline="") as handle:
    reader = csv.DictReader(handle, dialect="excel-tab")
    for line in reader:
        if line["id"] in members_to_get:
            uniparc_xref_metadata.append(line)

uniparc_xref_metadata = pd.DataFrame(uniparc_xref_metadata)
uniparc_xref_metadata = uniparc_xref_metadata[["id", "db", "db_id", "ncbi_taxid"]]
uniparc_xref_metadata.head()
```

In [ ]:

```
uniparc_sig_metadata = []

with open("data/nr_uniparc_signatures.tsv", newline="") as handle:
    reader = csv.DictReader(handle, dialect="excel-tab")
    for line in reader:
        if line["id"] in members_to_get:
            uniparc_sig_metadata.append(line)
            
uniparc_sig_metadata = pd.DataFrame(uniparc_sig_metadata)
uniparc_sig_metadata = uniparc_sig_metadata.drop(columns="checksum")
uniparc_sig_metadata.head()
```

Now I need a way of summarising the data.

I'll combine the database cross-references.

In [ ]:

```
dbxrefs = pd.concat([ipg_metadata, custom_metadata, uniparc_xref_metadata], ignore_index=True)
dbxrefs.loc[dbxrefs["ncbi_taxid"].isnull(), "ncbi_taxid"] = "."
dbxrefs.loc[[i is None for i in dbxrefs["ncbi_taxid"]], "ncbi_taxid"] = "."
dbxrefs["ncbi_taxid"] = dbxrefs["ncbi_taxid"].astype(str)
dbxrefs.head()
```

In [ ]:

```
# Save some memory
del ipg_metadata
del custom_metadata
del uniparc_xref_metadata
```

In [ ]:

```
%%bash

mkdir -p tmp
wget -P tmp/ -c https://ftp.ncbi.nih.gov/pub/taxonomy/taxdump.tar.gz 2>/dev/null
tar -C tmp/ -zxf tmp/taxdump.tar.gz
```

In [ ]:

```
target_taxids = {int(t) for t in dbxrefs["ncbi_taxid"] if (t != ".")}

taxid_to_species = pd.read_csv(
    "tmp/names.dmp",
    sep="\t\|\t?",
    engine="python",
    names=["taxid", "name", "unique_name", "kind", "junk"]
)

taxid_to_species = (
    taxid_to_species
    .loc[(taxid_to_species["taxid"].isin(target_taxids)) & (taxid_to_species["kind"] == "scientific name")]
)

taxid_to_species["taxid"] = taxid_to_species["taxid"].astype(str)
taxid_to_species = taxid_to_species.set_index("taxid")["name"].to_dict()
```

In [ ]:

```
dbxrefs["organism"] = dbxrefs["ncbi_taxid"].apply(lambda x: taxid_to_species.get(x, "."))
dbxrefs = pd.merge(
    clusters[["cluster", "pc", "effector_database","effector"]],
    dbxrefs.rename(columns={"id": "pc"}),
    on="pc", how="right"
).drop_duplicates()

dbxrefs.sort_values(["cluster", "pc", "organism", "db"], inplace=True)
dbxrefs.to_csv("07-find_effector_clusters-effector_clusters_dbxrefs.tsv", sep="\t", index=False)
dbxrefs
```

In [ ]:

```
cl_tax = (
    dbxrefs[dbxrefs["organism"] != "."]
    .groupby("cluster")
    ["organism"]
    .unique()
    .apply(lambda x: ",".join(x))
    .reset_index()
)

cl_eff = (
    clusters[["cluster", "effector_database", "effector"]]
    .drop_duplicates()
    .apply(lambda x: pd.Series([x["cluster"], f"{x['effector_database']}|{x['effector']}"], index=["cluster", "effector"]), axis=1)
    .groupby("cluster")
    ["effector"]
    .apply(lambda x: ",".join(x))
    .reset_index()
)

def map_fun(df):
    return pd.Series(
        [
            df["member"].nunique(),
            df["evalue"].min(),
            df["pident"].min(),
            df["pident"].median(),
            df["cluster_coverage"].min(),
            df["cluster_coverage"].median(),
            df["member_coverage"].min(),
            df["member_coverage"].median(),
        ],
        index=[
            "n_unique_sequences",
            "evalue_min",
            "pident_min",
            "pident_median",
            "cluster_coverage_min",
            "cluster_coverage_median",
            "member_coverage_min",
            "member_coverage_median"
        ]
    )

cl_stats = profile_stats.groupby("cluster").apply(map_fun)

cluster_stats = pd.merge(
    pd.merge(cl_eff, cl_tax, on="cluster", how="outer"),
    cl_stats,
    on="cluster",
    how="outer",
).sort_values(["n_unique_sequences", "cluster", "effector"], ascending=False, ignore_index=True)

cluster_stats.to_csv("07-find_effector_clusters-effector_clusters_stats.tsv", sep="\t", index=False)
cluster_stats
```

Find the interpro statistics for effector sequences.

In [ ]:

```
uniparc_sig_metadata_effector = pd.merge(
    clusters[["cluster", "pc", "effector_database", "effector"]].drop_duplicates(),
    uniparc_sig_metadata.rename(columns={"id": "pc"}),
    on="pc",
    how="left",
).drop_duplicates()

uniparc_sig_metadata_effector.sort_values(["cluster", "pc", "effector_database", "effector", "db", "db_id"], inplace=True)
uniparc_sig_metadata_effector.to_csv("07-find_effector_clusters-effector_clusters_interpro.tsv", sep="\t", index=False)
uniparc_sig_metadata_effector
```

In [ ]:

```
uniparc_sig_metadata_effector[
    (uniparc_sig_metadata_effector["effector_database"] == "custom") & (uniparc_sig_metadata_effector["db"].notnull())
].head(50)
```

Ok i think that's enough for the effector clusters.
The only other thing I'd like is to get the clusters for each dbxref and interpro annotations (not just the effector ones).

In [ ]:

```
all_clusters = pd.read_csv("data/profile.tsv", sep="\t").set_index("member")["cluster"].to_dict()
print(len(all_clusters))
```

In [ ]:

```
all_taxid_to_species = pd.read_csv(
    "tmp/names.dmp",
    sep="\t\|\t?",
    engine="python",
    names=["taxid", "name", "unique_name", "kind", "junk"]
)
all_taxid_to_species["taxid"] = all_taxid_to_species["taxid"].astype(str)

all_taxid_to_species = (
    all_taxid_to_species
    [all_taxid_to_species["kind"] == "scientific name"]
    .set_index("taxid")
    ["name"]
    .to_dict()
)

len(all_taxid_to_species)
```

In [ ]:

```
with open("data/nr_ipg.tsv", newline="") as handle, \
     open("07-find_effector_clusters-all_clusters_dbxrefs.tsv", "w", newline="") as outhandle:
    reader = csv.DictReader(handle, dialect="excel-tab")
    writer = csv.DictWriter(
        outhandle,
        ["cluster", "member", "db", "db_id", "ncbi_taxid", "organism", "doi"],
        dialect="excel-tab"
    )

    writer.writeheader()
    for line in reader:
        line["member"] = line["id"]
        line["doi"] = "."
        line["organism"] = all_taxid_to_species.get(line["ncbi_taxid"], ".")
        line["cluster"] = all_clusters.get(line["member"], ".")
        line = {k: line[k] for k in ["cluster", "member", "db", "db_id", "ncbi_taxid", "organism", "doi"]}
        for k in line:
            if (line[k] == "") or (line[k] is None):
                line[k] = "."

        writer.writerow(line)
```

In [ ]:

```
with open("data/nr_custom.tsv", newline="") as handle, \
     open("07-find_effector_clusters-all_clusters_dbxrefs.tsv", "a", newline="") as outhandle:
    reader = csv.DictReader(handle, dialect="excel-tab")
    writer = csv.DictWriter(
        outhandle,
        ["cluster", "member", "db", "db_id", "ncbi_taxid", "organism", "doi"],
        dialect="excel-tab"
    )

    for line in reader:
        line["member"] = line["id"]
        line["organism"] = all_taxid_to_species.get(line["ncbi_taxid"], ".")
        line["cluster"] = all_clusters.get(line["member"], ".")
        line = {k: line[k] for k in ["cluster", "member", "db", "db_id", "ncbi_taxid", "organism", "doi"]}
        for k in line:
            if (line[k] == "") or (line[k] is None):
                line[k] = "."

        writer.writerow(line)
```

In [ ]:

```
with open("data/nr_uniparc_xrefs.tsv", newline="") as handle, \
     open("07-find_effector_clusters-all_clusters_dbxrefs.tsv", "a", newline="") as outhandle:
    reader = csv.DictReader(handle, dialect="excel-tab")
    writer = csv.DictWriter(
        outhandle,
        ["cluster", "member", "db", "db_id", "ncbi_taxid", "organism", "doi"],
        dialect="excel-tab"
    )

    for line in reader:
        line["member"] = line["id"]
        line["doi"] = "."
        line["organism"] = all_taxid_to_species.get(line["ncbi_taxid"], ".")
        line["cluster"] = all_clusters.get(line["member"], ".")
        line = {k: line[k] for k in ["cluster", "member", "db", "db_id", "ncbi_taxid", "organism", "doi"]}
        for k in line:
            if (line[k] == "") or (line[k] is None):
                line[k] = "."

        writer.writerow(line)
```

In [ ]:

```
with open("data/nr_uniparc_signatures.tsv", newline="") as handle, \
     open("07-find_effector_clusters-all_clusters_interpro.tsv", "w", newline="") as outhandle:
    reader = csv.DictReader(handle, dialect="excel-tab")
    writer = csv.DictWriter(
        outhandle,
        ["cluster", "member", "start", "end", "db", "db_id", "interpro", "name"],
        dialect="excel-tab"
    )

    writer.writeheader()
    for line in reader:
        line["member"] = line["id"]
        line["cluster"] = all_clusters.get(line["member"], ".")
        line = {k: line[k] for k in ["cluster", "member", "start", "end", "db", "db_id", "interpro", "name"]}
        for k in line:
            if (line[k] == "") or (line[k] is None):
                line[k] = "."

        writer.writerow(line)
```

In [ ]:

```

```
