# Supplementary material for: Remote homology clustering identifies lowly conserved families of effector proteins in plant-pathogenic fungi
Source: Microb Genom. 2021 Sep 1;7(9):000637. doi: 10.1099/mgen.0.000637 (PMC8715435; doi:10.1099/mgen.0.000637)
Supplement: Supplementary material 12 [file mgen-7-0637-s0012.zip › supplementary_data_06-remeff_scripts/08-full_length_clustering.html]

08-full\_length\_clustering


# Clustering to find full length homologs.¶

In [1]:

```
import random

import pandas as pd
import seaborn as sns
import matplotlib.pyplot as plt

import numpy as np
import scipy as sp
from scipy.stats import beta
import networkx as nx

import markov_clustering as mc
```

First I need to load the alignments.

In [2]:

```
alignments = pd.read_csv(
    "./06d-get_high_coverage_weighted_scores.tsv",
    sep="\t",
    names=["min_id", "max_id", "query_id", "template_id", "probability", "evalue", "score", "lqh", "norm_score", "qcov", "tcov"],
)

pairs = alignments.sort_values(["min_id", "max_id"])
pairs["first"] = pairs["query_id"] == pairs["min_id"]

pairs = (
    pairs
    [["min_id", "max_id", "first", "norm_score", "qcov", "tcov"]]
    .pivot_table(index=["min_id", "max_id"], columns="first")
)

pairs.reset_index(inplace=True) 
pairs.head()
```

Out[2]:

|  | min\_id | max\_id | norm\_score | | qcov | | tcov | |
| --- | --- | --- | --- | --- | --- | --- | --- | --- |
| first |  |  | False | True | False | True | False | True |
| 0 | PC\_000008 | PC\_01ASVG | 0.049409 | 0.054126 | 0.701 | 0.738 | 0.738 | 0.701 |
| 1 | PC\_000008 | PC\_02ZTK7 | 0.066703 | 0.074876 | 0.774 | 0.718 | 0.718 | 0.774 |
| 2 | PC\_000008 | PC\_03KIZ3 | 0.044385 | 0.056920 | 0.820 | 0.815 | 0.815 | 0.820 |
| 3 | PC\_000008 | PC\_04OBCT | 0.093649 | 0.100567 | 0.754 | 0.788 | 0.788 | 0.754 |
| 4 | PC\_000008 | PC\_067ZDP | 0.064361 | 0.072645 | 0.759 | 0.720 | 0.741 | 0.734 |

In [3]:

```
alignments.head()
```

Out[3]:

|  | min\_id | max\_id | query\_id | template\_id | probability | evalue | score | lqh | norm\_score | qcov | tcov |
| --- | --- | --- | --- | --- | --- | --- | --- | --- | --- | --- | --- |
| 0 | PC\_000008 | PC\_08DGTB | PC\_000008 | PC\_08DGTB | 99.85 | 1.500000e-27 | 235.23 | 309692 | 0.110647 | 0.756 | 0.775 |
| 1 | PC\_000008 | PC\_04OBCT | PC\_000008 | PC\_04OBCT | 99.80 | 2.600000e-25 | 220.66 | 332529 | 0.100567 | 0.788 | 0.754 |
| 2 | PC\_000008 | PC\_08K0VV | PC\_000008 | PC\_08K0VV | 99.75 | 6.300000e-24 | 208.97 | 304122 | 0.099090 | 0.732 | 0.764 |
| 3 | PC\_000008 | PC\_087M85 | PC\_000008 | PC\_087M85 | 99.78 | 6.300000e-25 | 218.83 | 349239 | 0.097587 | 0.788 | 0.708 |
| 4 | PC\_000008 | PC\_068I4K | PC\_000008 | PC\_068I4K | 99.28 | 8.200000e-17 | 156.65 | 291311 | 0.075713 | 0.720 | 0.803 |

In [4]:

```
pairs.columns = ["minid", "maxid", "lnorm_score", "rnorm_score", "qcov_maxid", "qcov_minid", "tcov_minid", "tcov_maxid"]
pairs.reset_index(drop=True, inplace=True)

pairs["score"] = pairs[["lnorm_score", "rnorm_score"]].mean(axis=1)
pairs["score"] = pairs["score"] / pairs["score"].max()

pairs["minid_cov"] = pairs[["qcov_minid", "tcov_minid"]].mean(axis=1)
pairs["maxid_cov"] = pairs[["qcov_maxid", "tcov_maxid"]].mean(axis=1)

pairs.drop(
    columns=["lnorm_score", "rnorm_score", "qcov_maxid",
             "qcov_minid", "tcov_minid", "tcov_maxid"],
    inplace=True
)

pairs.head()
```

Out[4]:

|  | minid | maxid | score | minid\_cov | maxid\_cov |
| --- | --- | --- | --- | --- | --- |
| 0 | PC\_000008 | PC\_01ASVG | 0.047714 | 0.7380 | 0.7010 |
| 1 | PC\_000008 | PC\_02ZTK7 | 0.065247 | 0.7180 | 0.7740 |
| 2 | PC\_000008 | PC\_03KIZ3 | 0.046687 | 0.8150 | 0.8200 |
| 3 | PC\_000008 | PC\_04OBCT | 0.089505 | 0.7880 | 0.7540 |
| 4 | PC\_000008 | PC\_067ZDP | 0.063139 | 0.7305 | 0.7465 |

In [5]:

```
del alignments
```

I'll give each node a numerical id so that we can save a bit of ram.

In [6]:

```
ID_TO_INT = {
    j: i for i, j
    in enumerate(np.unique(np.sort(pairs[["minid", "maxid"]].values.flatten())))
}

INT_TO_ID = np.empty(len(ID_TO_INT), dtype="U9")
for k, v in ID_TO_INT.items():
    INT_TO_ID[v] = k
```

In [7]:

```
pairs["minid_int"] = pairs["minid"].apply(ID_TO_INT.get)
pairs["maxid_int"] = pairs["maxid"].apply(ID_TO_INT.get)
pairs.head()
```

Out[7]:

|  | minid | maxid | score | minid\_cov | maxid\_cov | minid\_int | maxid\_int |
| --- | --- | --- | --- | --- | --- | --- | --- |
| 0 | PC\_000008 | PC\_01ASVG | 0.047714 | 0.7380 | 0.7010 | 0 | 35668 |
| 1 | PC\_000008 | PC\_02ZTK7 | 0.065247 | 0.7180 | 0.7740 | 0 | 81617 |
| 2 | PC\_000008 | PC\_03KIZ3 | 0.046687 | 0.8150 | 0.8200 | 0 | 97231 |
| 3 | PC\_000008 | PC\_04OBCT | 0.089505 | 0.7880 | 0.7540 | 0 | 127022 |
| 4 | PC\_000008 | PC\_067ZDP | 0.063139 | 0.7305 | 0.7465 | 0 | 168944 |

## First pass clustering.¶

The first thing that I should try is the same greedy set cover algorithm that I've been using with MMSeqs.

In [8]:

```
def get_next_greedy_cluster(nodes, pairs):
    test_centroid = nodes[(nodes["cluster"] == -1)].iloc[0]

    if test_centroid["nedges"] < 1:
        nodes.loc[
            (nodes["cluster"] == -1) & (nodes["node"] == test_centroid["node"]),
            "cluster"
        ] = test_centroid["node"]
        return
    
    test_centroid_edges = pairs.loc[
            (pairs["minid_int"] == test_centroid["node"]) | (pairs["maxid_int"] == test_centroid["node"])
    ]

    test_centroid_edges_set = {test_centroid["node"]}
    test_centroid_edges_set.update(test_centroid_edges["minid_int"])
    test_centroid_edges_set.update(test_centroid_edges["maxid_int"])
    
    nodes.loc[
        (nodes["cluster"] == -1) & nodes["node"].isin(test_centroid_edges_set),
        "cluster"
    ] = test_centroid["node"]
    return
```

In [9]:

```
def greedy_set(pairs):
    print("finding nodes")
    nodes = pd.concat([
        pairs[["minid_int", "maxid_int"]].rename(columns={"minid_int": "node", "maxid_int": "neighbor"}),
        pairs[["maxid_int", "minid_int"]].rename(columns={"maxid_int": "node", "minid_int": "neighbor"}),
    ]).drop_duplicates()

    print("finding node degrees")
    nodes = nodes.groupby("node")["neighbor"].nunique()
    nodes = nodes.reset_index().rename(columns={"neighbor": "nedges"})
    nodes.sort_values("nedges", inplace=True, ascending=False, ignore_index=True)
    nodes["cluster"] = -1

    print("finding clusters:")
    i = 1
    print_freq = 2
    while len(pairs) > 0:
        if i % print_freq == 0:
            if i >= 10000:
                print_freq = 1000
            elif i >= 2000:
                print_freq = 500
            elif i >= 1000:
                print_freq = 200
            elif i >= 500:
                print_freq = 100
            elif i >= 100:
                print_freq = 50
            elif i >= 50:
                print_freq = 20
            elif i >= 10:
                print_freq = 10

            print("- iteration:", i, ", remaining aln:", len(pairs))

        get_next_greedy_cluster(nodes, pairs)

        remaining_ids = set(nodes.loc[nodes["cluster"] == -1, "node"])
        pairs = pairs[pairs["minid_int"].isin(remaining_ids) & pairs["maxid_int"].isin(remaining_ids)]
        if len(remaining_ids) == 0:
            break

        del remaining_ids
        i += 1
        
    print("\nStopping after", i, "iterations")
    print("There are", len(nodes.loc[nodes["cluster"] == -1, "node"]), "singletons.")

    # Some singletons will be left over at the end.
    # Because all of their aligned pairs were matched to something that they weren't.
    nodes.loc[nodes["cluster"] == -1, "cluster"] = nodes.loc[nodes["cluster"] == -1, "node"]    

    print("\nReassigning pairs to best cluster")
    
    nodes["representative"] = (nodes["cluster"] == nodes["cluster"])
    representatives = set(nodes.loc[nodes["representative"], "node"])
    clusters_to_reassign = (
        pd.concat([
            (pairs
             .loc[pairs["minid_int"].isin(representatives), ["minid_int", "maxid_int", "score"]]
             .rename(columns={"minid_int": "cluster", "maxid_int": "node"})),
            (pairs
             .loc[pairs["maxid_int"].isin(representatives), ["minid_int", "maxid_int", "score"]]
             .rename(columns={"maxid_int": "cluster", "minid_int": "node"})),
        ])
        .sort_values(["node", "score"], ascending=False)
        .groupby("node")
        .first()
        ["cluster"]
        .to_dict()
    )

    nodes.loc[nodes["node"].isin(clusters_to_reassign), "cluster"] = (
        nodes
        .loc[nodes["node"].isin(clusters_to_reassign), "node"]
        .apply(clusters_to_reassign.get)
    )
    return nodes
```

In [10]:

```
clusters = greedy_set(pairs)
```

```
finding nodes
finding node degrees
finding clusters:
- iteration: 2 , remaining aln: 13259593
- iteration: 4 , remaining aln: 9604146
- iteration: 6 , remaining aln: 8294026
- iteration: 8 , remaining aln: 7513934
- iteration: 10 , remaining aln: 7201441
- iteration: 20 , remaining aln: 5998302
- iteration: 30 , remaining aln: 5525286
- iteration: 40 , remaining aln: 4915079
- iteration: 50 , remaining aln: 4300648
- iteration: 60 , remaining aln: 4197160
- iteration: 80 , remaining aln: 4022356
- iteration: 100 , remaining aln: 3644506
- iteration: 150 , remaining aln: 2991817
- iteration: 200 , remaining aln: 2764787
- iteration: 250 , remaining aln: 2373264
- iteration: 300 , remaining aln: 2166035
- iteration: 350 , remaining aln: 2072191
- iteration: 400 , remaining aln: 1911738
- iteration: 450 , remaining aln: 1778910
- iteration: 500 , remaining aln: 1624829
- iteration: 600 , remaining aln: 1407948
- iteration: 700 , remaining aln: 1278592
- iteration: 800 , remaining aln: 1169480
- iteration: 900 , remaining aln: 1057502
- iteration: 1000 , remaining aln: 939801
- iteration: 1200 , remaining aln: 816771
- iteration: 1400 , remaining aln: 674931
- iteration: 1600 , remaining aln: 606757
- iteration: 1800 , remaining aln: 548674
- iteration: 2000 , remaining aln: 496093
- iteration: 2500 , remaining aln: 397993
- iteration: 3000 , remaining aln: 335003
- iteration: 3500 , remaining aln: 279623
- iteration: 4000 , remaining aln: 240155
- iteration: 4500 , remaining aln: 208872
- iteration: 5000 , remaining aln: 184570
- iteration: 5500 , remaining aln: 163971
- iteration: 6000 , remaining aln: 148294
- iteration: 6500 , remaining aln: 133115
- iteration: 7000 , remaining aln: 119735
- iteration: 7500 , remaining aln: 107754
- iteration: 8000 , remaining aln: 96979
- iteration: 8500 , remaining aln: 87121
- iteration: 9000 , remaining aln: 79984
- iteration: 9500 , remaining aln: 73254
- iteration: 10000 , remaining aln: 67504
- iteration: 11000 , remaining aln: 58197
- iteration: 12000 , remaining aln: 49610
- iteration: 13000 , remaining aln: 42723
- iteration: 14000 , remaining aln: 37123
- iteration: 15000 , remaining aln: 32877
- iteration: 16000 , remaining aln: 28747
- iteration: 17000 , remaining aln: 25404
- iteration: 18000 , remaining aln: 22517
- iteration: 19000 , remaining aln: 20153
- iteration: 20000 , remaining aln: 18188
- iteration: 21000 , remaining aln: 16084
- iteration: 22000 , remaining aln: 14449
- iteration: 23000 , remaining aln: 13026
- iteration: 24000 , remaining aln: 11592
- iteration: 25000 , remaining aln: 10322
- iteration: 26000 , remaining aln: 9358
- iteration: 27000 , remaining aln: 8417
- iteration: 28000 , remaining aln: 7466
- iteration: 29000 , remaining aln: 6616
- iteration: 30000 , remaining aln: 5913
- iteration: 31000 , remaining aln: 5264
- iteration: 32000 , remaining aln: 4689
- iteration: 33000 , remaining aln: 4243
- iteration: 34000 , remaining aln: 3766
- iteration: 35000 , remaining aln: 3286
- iteration: 36000 , remaining aln: 2834
- iteration: 37000 , remaining aln: 2398
- iteration: 38000 , remaining aln: 1951
- iteration: 39000 , remaining aln: 1533
- iteration: 40000 , remaining aln: 1155
- iteration: 41000 , remaining aln: 840
- iteration: 42000 , remaining aln: 539
- iteration: 43000 , remaining aln: 276
- iteration: 44000 , remaining aln: 110
- iteration: 45000 , remaining aln: 7

Stopping after 45299 iterations
There are 75 singletons.

Reassigning pairs to best cluster
```

Cool so that's finished.
What kinds of sizes to we get?

In [11]:

```
clusters.groupby("cluster")["node"].count().sort_values(ascending=False).describe()
```

Out[11]:

```
count    45373.000000
mean         4.941926
std         33.487500
min          1.000000
25%          1.000000
50%          2.000000
75%          3.000000
max       3021.000000
Name: node, dtype: float64
```

That's interesting.
I guess it makes sense that you wouldn't have *that* many large groups.
I guess i was expecting to have more in the range of 6-10.

I would like to make sure that I haven't made any mistakes and assigned something to a cluster that it has no edge with.

In [12]:

```
true_alignments = set(pairs.apply(lambda x: (int(x["minid_int"]), int(x["maxid_int"])), axis=1))
true_alignments

for i, row in clusters.iterrows():
    if row["node"] == row["cluster"]:
        continue
    pf = (int(row["node"]), int(row["cluster"]))
    pr = (int(row["cluster"]), int(row["node"]))
    assert pf in true_alignments or pr in true_alignments, row
```

## Finding connected components.¶

To find more remote homologs, we can relax the requirements having direct edges connecting members.
First we'll look at connected components.

In [13]:

```
matrix = sp.sparse.lil_matrix(
    (len(INT_TO_ID), len(INT_TO_ID)),
    dtype="float32"
)

matrix[pairs["minid_int"].values, pairs["maxid_int"].values] = pairs["score"].values
matrix[pairs["maxid_int"].values, pairs["minid_int"].values] = pairs["score"].values

matrix = matrix.tocsr()
```

In [14]:

```
G = nx.from_scipy_sparse_matrix(matrix)
ccs = list(nx.connected_components(G))
```

In [15]:

```
len(ccs)
```

Out[15]:

```
6538
```

In [16]:

```
sorted((len(c) for c in ccs), reverse=True)[:10]
```

Out[16]:

```
[171346, 2057, 1864, 1700, 1025, 896, 874, 791, 754, 719]
```

Ok. Well we have one really big connected component.
The other smaller ones would probably be useful.

I think I'll use the smaller ones to figure out a good inflation parameter for markov clustering.

We'll take a random sample of medium sized connected components, and run a grid of inflation values to see what gives a good clustering score.

In [16]:

```
best = []

choices = random.choices([c for c in ccs if (len(c) < 600) and (len(c) > 300)], k=10)
for i, test_cluster in enumerate(choices):
    print(f"Running set {i} with {len(test_cluster)} members\n")
    test_graph = G.subgraph(test_cluster).copy()
    nx.draw(test_graph)
    plt.show()
    plt.close()

    test_matrix = nx.to_scipy_sparse_matrix(test_graph)
    bestq = 0
    bestinf = None

    # perform clustering using different inflation values from 0.5 and 2.0
    # for each clustering run, calculate the modularity
    for inflation in np.linspace(1.1, 2.0, 19):
        test_result = mc.run_mcl(test_matrix, inflation=inflation)
        test_result_clusters = mc.get_clusters(test_result)
        Q = mc.modularity(matrix=test_result, clusters=test_result_clusters)
        if Q > bestq:
            bestq = Q
            bestinf = inflation

        print(
            "inflation:", inflation,
            "modularity:", Q,
            "nclusters:", len(test_result_clusters)
        )

        mc.draw_graph(test_matrix, test_result_clusters, node_size=50, with_labels=False, edge_color="silver")
        plt.show()
        plt.close()

    best.append((bestq, bestinf))
```

```
Running set 0 with 331 members
```

```
inflation: 1.1 modularity: 0.0042389668695512394 nclusters: 6
```

```
inflation: 1.1500000000000001 modularity: 0.719361816704847 nclusters: 9
```

```
inflation: 1.2000000000000002 modularity: 0.7289546462701125 nclusters: 12
```

```
inflation: 1.25 modularity: 0.8119221255738805 nclusters: 18
```

```
inflation: 1.3 modularity: 0.8113197214337229 nclusters: 22
```

```
inflation: 1.35 modularity: 0.7963143819424809 nclusters: 30
```

```
inflation: 1.4000000000000001 modularity: 0.7911300554029244 nclusters: 34
```

```
inflation: 1.4500000000000002 modularity: 0.7939047653818403 nclusters: 38
```

```
inflation: 1.5 modularity: 0.7874882485555975 nclusters: 41
```

```
inflation: 1.55 modularity: 0.7910387820483553 nclusters: 44
```

```
inflation: 1.6 modularity: 0.7871687918146043 nclusters: 46
```

```
inflation: 1.65 modularity: 0.7841658984492643 nclusters: 47
```

```
inflation: 1.7000000000000002 modularity: 0.7814550798185473 nclusters: 50
```

```
inflation: 1.75 modularity: 0.7744179041812312 nclusters: 53
```

```
inflation: 1.8 modularity: 0.7697264537563537 nclusters: 55
```

```
inflation: 1.85 modularity: 0.7613201778004935 nclusters: 58
```

```
inflation: 1.9 modularity: 0.7590657259426243 nclusters: 59
```

```
inflation: 1.9500000000000002 modularity: 0.7548580242969659 nclusters: 62
```

```
inflation: 2.0 modularity: 0.7474192458995436 nclusters: 65
```

```
Running set 1 with 353 members
```

```
inflation: 1.1 modularity: 0.7778410869198882 nclusters: 7
```

```
inflation: 1.1500000000000001 modularity: 0.8151658387435935 nclusters: 10
```

```
inflation: 1.2000000000000002 modularity: 0.8188975114157103 nclusters: 13
```

```
inflation: 1.25 modularity: 0.8123089022462214 nclusters: 18
```

```
inflation: 1.3 modularity: 0.7998860435442023 nclusters: 26
```

```
inflation: 1.35 modularity: 0.7939314174738548 nclusters: 30
```

```
inflation: 1.4000000000000001 modularity: 0.7856495116725083 nclusters: 34
```

```
inflation: 1.4500000000000002 modularity: 0.7858340890304836 nclusters: 35
```

```
inflation: 1.5 modularity: 0.7989069810366807 nclusters: 42
```

```
inflation: 1.55 modularity: 0.7872224317665636 nclusters: 48
```

```
inflation: 1.6 modularity: 0.7818777134877876 nclusters: 50
```

```
inflation: 1.65 modularity: 0.7725284690511903 nclusters: 55
```

```
inflation: 1.7000000000000002 modularity: 0.7702573650378369 nclusters: 56
```

```
inflation: 1.75 modularity: 0.7709715991621785 nclusters: 61
```

```
inflation: 1.8 modularity: 0.7666380438010086 nclusters: 63
```

```
inflation: 1.85 modularity: 0.7504594371193084 nclusters: 69
```

```
inflation: 1.9 modularity: 0.750443386914267 nclusters: 69
```

```
inflation: 1.9500000000000002 modularity: 0.7479315298252929 nclusters: 70
```

```
inflation: 2.0 modularity: 0.7450344678153257 nclusters: 71
```

```
Running set 2 with 465 members
```

```
inflation: 1.1 modularity: 0.43798358191698183 nclusters: 4
```

```
inflation: 1.1500000000000001 modularity: 0.6723366863221175 nclusters: 7
```

```
inflation: 1.2000000000000002 modularity: 0.7793733379581456 nclusters: 17
```

```
inflation: 1.25 modularity: 0.7787351138859976 nclusters: 21
```

```
inflation: 1.3 modularity: 0.7876332523991217 nclusters: 31
```

```
inflation: 1.35 modularity: 0.7977060931899628 nclusters: 37
```

```
inflation: 1.4000000000000001 modularity: 0.7981731992137834 nclusters: 46
```

```
inflation: 1.4500000000000002 modularity: 0.7898161637183513 nclusters: 53
```

```
inflation: 1.5 modularity: 0.8043473233899892 nclusters: 57
```

```
inflation: 1.55 modularity: 0.8098647242455789 nclusters: 60
```

```
inflation: 1.6 modularity: 0.8009342120476358 nclusters: 65
```

```
inflation: 1.65 modularity: 0.7881003584229406 nclusters: 72
```

```
inflation: 1.7000000000000002 modularity: 0.7701098392877791 nclusters: 82
```

```
inflation: 1.75 modularity: 0.7613550699502823 nclusters: 87
```

```
inflation: 1.8 modularity: 0.7580807029714405 nclusters: 89
```

```
inflation: 1.85 modularity: 0.7522811885767137 nclusters: 93
```

```
inflation: 1.9 modularity: 0.7419771071800209 nclusters: 99
```

```
inflation: 1.9500000000000002 modularity: 0.7398265695456124 nclusters: 100
```

```
inflation: 2.0 modularity: 0.7354006243496349 nclusters: 103
```

```
Running set 3 with 353 members
```

```
inflation: 1.1 modularity: 0.7778410869198882 nclusters: 7
```

```
inflation: 1.1500000000000001 modularity: 0.8151658387435935 nclusters: 10
```

```
inflation: 1.2000000000000002 modularity: 0.8188975114157103 nclusters: 13
```

```
inflation: 1.25 modularity: 0.8123089022462214 nclusters: 18
```

```
inflation: 1.3 modularity: 0.7998860435442023 nclusters: 26
```

```
inflation: 1.35 modularity: 0.7939314174738548 nclusters: 30
```

```
inflation: 1.4000000000000001 modularity: 0.7856495116725083 nclusters: 34
```

```
inflation: 1.4500000000000002 modularity: 0.7858340890304836 nclusters: 35
```

```
inflation: 1.5 modularity: 0.7989069810366807 nclusters: 42
```

```
inflation: 1.55 modularity: 0.7872224317665636 nclusters: 48
```

```
inflation: 1.6 modularity: 0.7818777134877876 nclusters: 50
```

```
inflation: 1.65 modularity: 0.7725284690511903 nclusters: 55
```

```
inflation: 1.7000000000000002 modularity: 0.7702573650378369 nclusters: 56
```

```
inflation: 1.75 modularity: 0.7709715991621785 nclusters: 61
```

```
inflation: 1.8 modularity: 0.7666380438010086 nclusters: 63
```

```
inflation: 1.85 modularity: 0.7504594371193084 nclusters: 69
```

```
inflation: 1.9 modularity: 0.750443386914267 nclusters: 69
```

```
inflation: 1.9500000000000002 modularity: 0.7479315298252929 nclusters: 70
```

```
inflation: 2.0 modularity: 0.7450344678153257 nclusters: 71
```

```
Running set 4 with 409 members
```

```
inflation: 1.1 modularity: 0.5381364291222512 nclusters: 3
```

```
inflation: 1.1500000000000001 modularity: 0.6253489637197259 nclusters: 8
```

```
inflation: 1.2000000000000002 modularity: 0.6386977600564343 nclusters: 15
```

```
inflation: 1.25 modularity: 0.6547665305683255 nclusters: 25
```

```
inflation: 1.3 modularity: 0.6755160478476326 nclusters: 38
```

```
inflation: 1.35 modularity: 0.6810875114328586 nclusters: 48
```

```
inflation: 1.4000000000000001 modularity: 0.6794794387886245 nclusters: 59
```

```
inflation: 1.4500000000000002 modularity: 0.6740036226469248 nclusters: 65
```

```
inflation: 1.5 modularity: 0.6628965632677928 nclusters: 75
```

```
inflation: 1.55 modularity: 0.672299902559165 nclusters: 82
```

```
inflation: 1.6 modularity: 0.6647138646947357 nclusters: 91
```

```
inflation: 1.65 modularity: 0.6594412993705189 nclusters: 95
```

```
inflation: 1.7000000000000002 modularity: 0.6549578254553708 nclusters: 97
```

```
inflation: 1.75 modularity: 0.6539355934027178 nclusters: 98
```

```
inflation: 1.8 modularity: 0.6497390618181379 nclusters: 100
```

```
inflation: 1.85 modularity: 0.6384466855171823 nclusters: 107
```

```
inflation: 1.9 modularity: 0.6263891296680425 nclusters: 112
```

```
inflation: 1.9500000000000002 modularity: 0.6541986238724058 nclusters: 118
```

```
inflation: 2.0 modularity: 0.647078867295149 nclusters: 121
```

```
Running set 5 with 325 members
```

```
inflation: 1.1 modularity: 0.11139408284023646 nclusters: 5
```

```
inflation: 1.1500000000000001 modularity: 0.1875124260355032 nclusters: 9
```

```
inflation: 1.2000000000000002 modularity: 0.45304615384615265 nclusters: 14
```

```
inflation: 1.25 modularity: 0.47252071005917157 nclusters: 23
```

```
inflation: 1.3 modularity: 0.47691360946745565 nclusters: 29
```

```
inflation: 1.35 modularity: 0.60881420118343 nclusters: 39
```

```
inflation: 1.4000000000000001 modularity: 0.6643692307692302 nclusters: 45
```

```
inflation: 1.4500000000000002 modularity: 0.7258414201183432 nclusters: 48
```

```
inflation: 1.5 modularity: 0.7237585798816568 nclusters: 50
```

```
inflation: 1.55 modularity: 0.7466130177514794 nclusters: 52
```

```
inflation: 1.6 modularity: 0.7444639053254442 nclusters: 53
```

```
inflation: 1.65 modularity: 0.7400804733727817 nclusters: 54
```

```
inflation: 1.7000000000000002 modularity: 0.7252639053254445 nclusters: 59
```

```
inflation: 1.75 modularity: 0.7179644970414204 nclusters: 62
```

```
inflation: 1.8 modularity: 0.7148875739644965 nclusters: 63
```

```
inflation: 1.85 modularity: 0.7101065088757402 nclusters: 66
```

```
inflation: 1.9 modularity: 0.7028071005917158 nclusters: 69
```

```
inflation: 1.9500000000000002 modularity: 0.695526627218935 nclusters: 72
```

```
inflation: 2.0 modularity: 0.6911905325443795 nclusters: 74
```

```
Running set 6 with 597 members
```

```
inflation: 1.1 modularity: 0.7084108425993672 nclusters: 13
```

```
inflation: 1.1500000000000001 modularity: 0.7510556691890512 nclusters: 22
```

```
inflation: 1.2000000000000002 modularity: 0.7802356281687639 nclusters: 28
```

```
inflation: 1.25 modularity: 0.785437517009952 nclusters: 36
```

```
inflation: 1.3 modularity: 0.8150411465479288 nclusters: 41
```

```
inflation: 1.35 modularity: 0.8228495913402857 nclusters: 50
```

```
inflation: 1.4000000000000001 modularity: 0.8088993263357537 nclusters: 62
```

```
inflation: 1.4500000000000002 modularity: 0.7982795047263092 nclusters: 69
```

```
inflation: 1.5 modularity: 0.7993569185963314 nclusters: 71
```

```
inflation: 1.55 modularity: 0.7901063104467028 nclusters: 80
```

```
inflation: 1.6 modularity: 0.7848735581873649 nclusters: 85
```

```
inflation: 1.65 modularity: 0.7805779315337175 nclusters: 88
```

```
inflation: 1.7000000000000002 modularity: 0.7719866782264225 nclusters: 94
```

```
inflation: 1.75 modularity: 0.7637573686410858 nclusters: 99
```

```
inflation: 1.8 modularity: 0.755334461250978 nclusters: 105
```

```
inflation: 1.85 modularity: 0.7487689704805425 nclusters: 109
```

```
inflation: 1.9 modularity: 0.7426580136865218 nclusters: 113
```

```
inflation: 1.9500000000000002 modularity: 0.736552668423076 nclusters: 117
```

```
inflation: 2.0 modularity: 0.7296168166348195 nclusters: 121
```

```
Running set 7 with 465 members
```

```
inflation: 1.1 modularity: 0.43798358191698183 nclusters: 4
```

```
inflation: 1.1500000000000001 modularity: 0.6723366863221175 nclusters: 7
```

```
inflation: 1.2000000000000002 modularity: 0.7793733379581456 nclusters: 17
```

```
inflation: 1.25 modularity: 0.7787351138859976 nclusters: 21
```

```
inflation: 1.3 modularity: 0.7876332523991217 nclusters: 31
```

```
inflation: 1.35 modularity: 0.7977060931899628 nclusters: 37
```

```
inflation: 1.4000000000000001 modularity: 0.7981731992137834 nclusters: 46
```

```
inflation: 1.4500000000000002 modularity: 0.7898161637183513 nclusters: 53
```

```
inflation: 1.5 modularity: 0.8043473233899892 nclusters: 57
```

```
inflation: 1.55 modularity: 0.8098647242455789 nclusters: 60
```

```
inflation: 1.6 modularity: 0.8009342120476358 nclusters: 65
```

```
inflation: 1.65 modularity: 0.7881003584229406 nclusters: 72
```

```
inflation: 1.7000000000000002 modularity: 0.7701098392877791 nclusters: 82
```

```
inflation: 1.75 modularity: 0.7613550699502823 nclusters: 87
```

```
inflation: 1.8 modularity: 0.7580807029714405 nclusters: 89
```

```
inflation: 1.85 modularity: 0.7522811885767137 nclusters: 93
```

```
inflation: 1.9 modularity: 0.7419771071800209 nclusters: 99
```

```
inflation: 1.9500000000000002 modularity: 0.7398265695456124 nclusters: 100
```

```
inflation: 2.0 modularity: 0.7354006243496349 nclusters: 103
```

```
Running set 8 with 381 members
```

```
inflation: 1.1 modularity: 0.2172759901075359 nclusters: 7
```

```
inflation: 1.1500000000000001 modularity: 0.37601008535350433 nclusters: 11
```

```
inflation: 1.2000000000000002 modularity: 0.6453937352319173 nclusters: 23
```

```
inflation: 1.25 modularity: 0.6758977962400367 nclusters: 31
```

```
inflation: 1.3 modularity: 0.6988033976067957 nclusters: 40
```

```
inflation: 1.35 modularity: 0.6972258388961238 nclusters: 55
```

```
inflation: 1.4000000000000001 modularity: 0.6793146919627178 nclusters: 67
```

```
inflation: 1.4500000000000002 modularity: 0.676159574541372 nclusters: 71
```

```
inflation: 1.5 modularity: 0.6843987021307397 nclusters: 75
```

```
inflation: 1.55 modularity: 0.6659915542053323 nclusters: 85
```

```
inflation: 1.6 modularity: 0.6611004333119795 nclusters: 87
```

```
inflation: 1.65 modularity: 0.6579590936959647 nclusters: 91
```

```
inflation: 1.7000000000000002 modularity: 0.6446635115492462 nclusters: 97
```

```
inflation: 1.75 modularity: 0.6397172794345597 nclusters: 99
```

```
inflation: 1.8 modularity: 0.6319672639345286 nclusters: 102
```

```
inflation: 1.85 modularity: 0.6250783612678341 nclusters: 106
```

```
inflation: 1.9 modularity: 0.6250783612678341 nclusters: 106
```

```
inflation: 1.9500000000000002 modularity: 0.615041230082461 nclusters: 111
```

```
inflation: 2.0 modularity: 0.6024552049104107 nclusters: 116
```

```
Running set 9 with 497 members
```

```
inflation: 1.1 modularity: 0.7787853883866551 nclusters: 10
```

```
inflation: 1.1500000000000001 modularity: 0.7943354290734317 nclusters: 17
```

```
inflation: 1.2000000000000002 modularity: 0.8374472185224002 nclusters: 24
```

```
inflation: 1.25 modularity: 0.8412851353594422 nclusters: 28
```

```
inflation: 1.3 modularity: 0.8332044581371507 nclusters: 40
```

```
inflation: 1.35 modularity: 0.8475399681792971 nclusters: 47
```

```
inflation: 1.4000000000000001 modularity: 0.8313907590411695 nclusters: 58
```

```
inflation: 1.4500000000000002 modularity: 0.814197053548659 nclusters: 71
```

```
inflation: 1.5 modularity: 0.7982381208781851 nclusters: 81
```

```
inflation: 1.55 modularity: 0.7925419721548587 nclusters: 84
```

```
inflation: 1.6 modularity: 0.7744535624208007 nclusters: 94
```

```
inflation: 1.65 modularity: 0.7733280973567742 nclusters: 96
```

```
inflation: 1.7000000000000002 modularity: 0.7655065200053417 nclusters: 100
```

```
inflation: 1.75 modularity: 0.7669841989563126 nclusters: 103
```

```
inflation: 1.8 modularity: 0.7646523001186181 nclusters: 105
```

```
inflation: 1.85 modularity: 0.7564096854770462 nclusters: 109
```

```
inflation: 1.9 modularity: 0.7523693468659047 nclusters: 111
```

```
inflation: 1.9500000000000002 modularity: 0.7462359671105103 nclusters: 114
```

```
inflation: 2.0 modularity: 0.7462359671105103 nclusters: 114
```

By eye, about 1.2-1.5 seems to give good results.

What's the average best score.

In [17]:

```
sum(v for k, v in best) / len(best)
```

Out[17]:

```
1.3650000000000002
```

I've run this a few times, and the number usually comes close to 1.35.

In [17]:

```
mcl_result = mc.run_mcl(matrix, inflation=1.35, expansion=2)
mcl_clusters = mc.get_clusters(mcl_result)

test_clusters = sorted(mcl_clusters, reverse=True, key=lambda x: len(x))
len(test_clusters)
```

```
/home/ubuntu/.miniconda/envs/remote_homology/lib/python3.8/site-packages/scipy/sparse/_index.py:116: SparseEfficiencyWarning: Changing the sparsity structure of a csc_matrix is expensive. lil_matrix is more efficient.
  self._set_arrayXarray_sparse(i, j, x)
```

Out[17]:

```
27851
```

In [18]:

```
[len(x) for x in test_clusters][:5]
```

Out[18]:

```
[3508, 3349, 2644, 2438, 2277]
```

Cool.
So this is somewhere in-between the greedy set and connected components.

Now I need to join each of these clustered sets with the effector information, and write out a graph file with that information that I can use to visualise.

In [19]:

```
nodes = {i: {"node_id": id_, "node_int": i} for i, id_ in enumerate(INT_TO_ID)}
```

In [20]:

```
for i, row in clusters.iterrows():
    nodes[row["node"]]["nedges"] = row["nedges"]
    nodes[row["node"]]["supercluster_greedy"] = INT_TO_ID[row["cluster"]]
```

In [21]:

```
for con_comp in ccs:
    members = list()
    for n in con_comp:
        members.append(nodes[n])
    
    members.sort(key=lambda x: x["nedges"], reverse=True)
    assert len(members) > 0, members
    rep = members[0]["node_id"]
    for m in members:
        m["supercluster_component"] = rep
```

In [22]:

```
for mcl in mcl_clusters:
    members = list()
    for n in mcl:
        members.append(nodes[n])
    
    members.sort(key=lambda x: x["nedges"], reverse=True)
    assert len(members) > 0, members
    rep = members[0]["node_id"]
    for m in members:
        m["supercluster_markov"] = rep
```

In [23]:

```
nodes_df = pd.DataFrame(nodes.values())
nodes_df = nodes_df[[
    'supercluster_component', 'supercluster_markov', 'supercluster_greedy',
    'node_id', 'node_int', 'nedges'
]]
nodes_df.to_csv("08-full_length_clustering-nodes.tsv", sep="\t", index=False)
nodes_df
```

Out[23]:

|  | supercluster\_component | supercluster\_markov | supercluster\_greedy | node\_id | node\_int | nedges |
| --- | --- | --- | --- | --- | --- | --- |
| 0 | PC\_06FCV2 | PC\_000008 | PC\_08K0VV | PC\_000008 | 0 | 10 |
| 1 | PC\_06FCV2 | PC\_00000G | PC\_02Q77Y | PC\_00000G | 1 | 46 |
| 2 | PC\_08JPQU | PC\_00001B | PC\_00001B | PC\_00001B | 2 | 1 |
| 3 | PC\_06FCV2 | PC\_03KHAU | PC\_03KHAU | PC\_00002Z | 3 | 240 |
| 4 | PC\_06FCV2 | PC\_03GH3C | PC\_03GH3C | PC\_000033 | 4 | 784 |
| ... | ... | ... | ... | ... | ... | ... |
| 224225 | PC\_06FCV2 | PC\_08L6ZB | PC\_074H46 | PC\_08L6ZB | 224225 | 108 |
| 224226 | PC\_06FCV2 | PC\_0381HR | PC\_0381HR | PC\_08L6ZL | 224226 | 143 |
| 224227 | PC\_06FCV2 | PC\_074H46 | PC\_074H46 | PC\_08L705 | 224227 | 315 |
| 224228 | PC\_06FCV2 | PC\_08L70J | PC\_08L70J | PC\_08L70J | 224228 | 7 |
| 224229 | PC\_06FCV2 | PC\_074H46 | PC\_089UFN | PC\_08L712 | 224229 | 87 |

224230 rows × 6 columns

In [24]:

```
pairs.to_csv("08-full_length_clustering-edges.tsv", sep="\t", index=False)
```

In [25]:

```
for node in G.nodes:
    G.nodes[node]["supercluster_component"] = str(nodes_df.loc[node, "supercluster_component"])
    G.nodes[node]["supercluster_greedy"] = str(nodes_df.loc[node, "supercluster_greedy"])
    G.nodes[node]["supercluster_markov"] = str(nodes_df.loc[node, "supercluster_markov"])
    G.nodes[node]["cluster"] = str(nodes_df.loc[node, "node_id"])
    G.nodes[node]["nedges"] = int(nodes_df.loc[node, "nedges"])
```

In [26]:

```
for i, row in pairs.iterrows():
    e = G.edges[(row["maxid_int"], row["minid_int"])]
    e["minid_cov"] = row["minid_cov"]
    e["maxid_cov"] = row["maxid_cov"]
    e["weight"] = float(e["weight"])
```

I've tried to save this graph few times with networkx, but it seems like there must be a memory leak somewhere.
It uses like 50Gb of RAM :(.

Instead I'll convert it to graph\_tool, which is much faster.

In [27]:

```
# This cell from https://gist.github.com/bbengfort/a430d460966d64edc6cad71c502d7005
# also http://kuanbutts.com/2018/08/17/peartree-to-graph-tool/
import graph_tool as gt


def get_prop_type(value, key=None):
    """
    Performs typing and value conversion for the graph_tool PropertyMap class.
    If a key is provided, it also ensures the key is in a format that can be
    used with the PropertyMap. Returns a tuple, (type name, value, key)
    """
    # Ensure that key is returned as a str type
    if isinstance(key, bytes):
        key = key.decode()

    # Deal with the value
    if isinstance(value, bool):
        tname = 'bool'

    elif isinstance(value, int):
        tname = 'int32_t'
        value = int(value)

    elif isinstance(value, float):
        tname = 'float'

    elif isinstance(value, bytes):
        tname = 'string'
        value = value.decode()

    elif isinstance(value, dict):
        tname = 'object'

    else:
        tname = 'string'
        value = str(value)

    return tname, value, key


def nx2gt(nxG):
    """
    Converts a networkx graph to a graph-tool graph.
    """
    # Phase 0: Create a directed or undirected graph-tool Graph
    gtG = gt.Graph(directed=nxG.is_directed())

    # Add the Graph properties as "internal properties"
    for key, value in nxG.graph.items():
        # Convert the value and key into a type for graph-tool
        tname, value, key = get_prop_type(value, key)

        prop = gtG.new_graph_property(tname) # Create the PropertyMap
        gtG.graph_properties[key] = prop     # Set the PropertyMap
        gtG.graph_properties[key] = value    # Set the actual value

    # Phase 1: Add the vertex and edge property maps
    # Go through all nodes and edges and add seen properties
    # Add the node properties first
    nprops = set() # cache keys to only add properties once
    for node, data in nxG.nodes(data=True):

        # Go through all the properties if not seen and add them.
        for key, val in data.items():
            if key in nprops: continue # Skip properties already added

            # Convert the value and key into a type for graph-tool
            tname, _, key  = get_prop_type(val, key)

            prop = gtG.new_vertex_property(tname) # Create the PropertyMap
            gtG.vertex_properties[key] = prop     # Set the PropertyMap

            # Add the key to the already seen properties
            nprops.add(key)

    # Also add the node id: in NetworkX a node can be any hashable type, but
    # in graph-tool node are defined as indices. So we capture any strings
    # in a special PropertyMap called 'id' -- modify as needed!
    gtG.vertex_properties['id'] = gtG.new_vertex_property('string')

    # Add the edge properties second
    eprops = set() # cache keys to only add properties once
    for src, dst, data in nxG.edges(data=True):

        # Go through all the edge properties if not seen and add them.
        for key, val in data.items():
            if key in eprops: continue # Skip properties already added

            # Convert the value and key into a type for graph-tool
            tname, _, key = get_prop_type(val, key)

            prop = gtG.new_edge_property(tname) # Create the PropertyMap
            gtG.edge_properties[key] = prop     # Set the PropertyMap

            # Add the key to the already seen properties
            eprops.add(key)
            
    print(gtG.edge_properties)

    # Phase 2: Actually add all the nodes and vertices with their properties
    # Add the nodes
    vertices = {} # vertex mapping for tracking edges later
    for node, data in nxG.nodes(data=True):

        # Create the vertex and annotate for our edges later
        v = gtG.add_vertex(n=1)
        vertices[node] = v

        # Set the vertex properties, not forgetting the id property
        data['id'] = str(node)
        for key, value in data.items():
            tname, value, key = get_prop_type(value, key)
            gtG.vp[key][v] = value # vp is short for vertex_properties

    # Add the edges
    for src, dst, data in nxG.edges(data=True):

        # Look up the vertex structs from our vertices mapping and add edge.
        e = gtG.add_edge(vertices[src], vertices[dst])

        # Add the edge properties
        for key, value in data.items():
            try:
                gtG.ep[key][e] = value # ep is short for edge_properties
            except:
                print(e, key, value)
                raise

    # Done, finally!
    return gtG
```

In [28]:

```
converted = nx2gt(G)
```

```
{'weight': <EdgePropertyMap object with value type 'double', for Graph 0x7f3ec08ed1f0, at 0x7f3ec08ed0a0>, 'minid_cov': <EdgePropertyMap object with value type 'double', for Graph 0x7f3ec08ed1f0, at 0x7f3ec08ed880>, 'maxid_cov': <EdgePropertyMap object with value type 'double', for Graph 0x7f3ec08ed1f0, at 0x7f3ec08f0d90>}
```

In [29]:

```
converted.save("08-full_length_clustering.gt")
```

In [30]:

```
converted.save("08-full_length_clustering.xml.gz")
```

In [40]:

```
import graph_tool.all as gta
import seaborn as sns
from matplotlib.colors import ListedColormap
```

In [34]:

```
pos = gta.sfdp_layout(converted, eweight=converted.ep["weight"])
```

In [46]:

```
converted_cl = converted.new_vertex_property("int")
mapping = {k: v for v, k in enumerate(set(converted.vertex_properties["supercluster_markov"]))}
for v in converted.vertices():
    converted_cl[v] = mapping[converted.vertex_properties["supercluster_markov"][v]]
```

In [47]:

```
cmap = ListedColormap(sns.color_palette("colorblind"), N=len(mapping))
gta.graph_draw(
    converted,
    output="08-full_length_clustering_markov.png",
    pos=pos,
    output_size=(3600, 3600),
    vertex_fill_color=converted_cl,
    vcmap=cmap
)
```

Out[47]:

```
<VertexPropertyMap object with value type 'vector<double>', for Graph 0x7f3ec08ed1f0, at 0x7f41dc274c40>
```

In [44]:

```
converted_cl = converted.new_vertex_property("int")
mapping = {k: v for v, k in enumerate(set(converted.vertex_properties["supercluster_greedy"]))}
for v in converted.vertices():
    converted_cl[v] = mapping[converted.vertex_properties["supercluster_greedy"][v]]
```

In [45]:

```
cmap = ListedColormap(sns.color_palette("colorblind"), N=len(mapping))
gta.graph_draw(
    converted,
    output="08-full_length_clustering_greedy.png",
    pos=pos,
    output_size=(3600, 3600),
    vertex_fill_color=converted_cl,
    vcmap=cmap
)
```

Out[45]:

```
<VertexPropertyMap object with value type 'vector<double>', for Graph 0x7f3ec08ed1f0, at 0x7f41dc274c40>
```

In [49]:

```
converted_cl = converted.new_vertex_property("int")
mapping = {k: v for v, k in enumerate(set(converted.vertex_properties["supercluster_component"]))}
for v in converted.vertices():
    converted_cl[v] = mapping[converted.vertex_properties["supercluster_component"][v]]
```

In [50]:

```
cmap = ListedColormap(sns.color_palette("colorblind"), N=len(mapping))
gta.graph_draw(
    converted,
    output="08-full_length_clustering_component.png",
    pos=pos,
    output_size=(3600, 3600),
    vertex_fill_color=converted_cl,
    vcmap=cmap
)
```

Out[50]:

```
<VertexPropertyMap object with value type 'vector<double>', for Graph 0x7f3ec08ed1f0, at 0x7f41dc274c40>
```

In [ ]:

```

```
