# Supplementary material for: Remote homology clustering identifies lowly conserved families of effector proteins in plant-pathogenic fungi
Source: Microb Genom. 2021 Sep 1;7(9):000637. doi: 10.1099/mgen.0.000637 (PMC8715435; doi:10.1099/mgen.0.000637)
Supplement: Supplementary material 12 [file mgen-7-0637-s0012.zip › supplementary_data_06-remeff_scripts/09-truncated_clustering.html]

09-truncated\_clustering


# Clustering to find truncated and full length homologs.¶

In [1]:

```
import random

import pandas as pd
import seaborn as sns
import matplotlib.pyplot as plt

import numpy as np
import scipy as sp

import networkx as nx

import markov_clustering as mc
```

First I need to load the alignments.

In [2]:

```
alignments = pd.read_csv(
    "./06c-best_50.tsv",
    sep="\t",
    names=["min_id", "max_id", "query_id", "template_id", "probability", "evalue", "score", "lqh", "norm_score", "qcov", "tcov"],
)

pairs = alignments.sort_values(["min_id", "max_id"])
pairs["first"] = pairs["query_id"] == pairs["min_id"]

pairs = (
    pairs
    [["min_id", "max_id", "first", "norm_score", "qcov", "tcov"]]
    .pivot_table(index=["min_id", "max_id"], columns="first")
)

pairs.reset_index(inplace=True) 
pairs.head()
```

Out[2]:

|  | min\_id | max\_id | norm\_score | | qcov | | tcov | |
| --- | --- | --- | --- | --- | --- | --- | --- | --- |
| first |  |  | False | True | False | True | False | True |
| 0 | PC\_000008 | PC\_001ISM | 0.101960 | 0.111247 | 0.956 | 0.115 | 0.115 | 0.956 |
| 1 | PC\_000008 | PC\_0038EV | 0.101820 | 0.107945 | 0.824 | 0.346 | 0.348 | 0.820 |
| 2 | PC\_000008 | PC\_005RBZ | 0.093401 | 0.096911 | 0.762 | 0.429 | 0.449 | 0.717 |
| 3 | PC\_000008 | PC\_006QGB | 0.089138 | 0.096322 | 0.866 | 0.190 | 0.189 | 0.874 |
| 4 | PC\_000008 | PC\_008ZRK | 0.078557 | 0.088743 | 0.958 | 0.115 | 0.113 | 0.972 |

In [3]:

```
alignments.head()
```

Out[3]:

|  | min\_id | max\_id | query\_id | template\_id | probability | evalue | score | lqh | norm\_score | qcov | tcov |
| --- | --- | --- | --- | --- | --- | --- | --- | --- | --- | --- | --- |
| 0 | PC\_000008 | PC\_04OEQ4 | PC\_000008 | PC\_04OEQ4 | 99.74 | 9.300000e-24 | 198.03 | 182139 | 0.117895 | 0.515 | 0.920 |
| 1 | PC\_000008 | PC\_03UY5T | PC\_000008 | PC\_03UY5T | 99.75 | 6.200000e-24 | 200.16 | 197178 | 0.115040 | 0.560 | 0.890 |
| 2 | PC\_000008 | PC\_001ISM | PC\_000008 | PC\_001ISM | 98.46 | 9.100000e-12 | 93.07 | 37876 | 0.111247 | 0.115 | 0.956 |
| 3 | PC\_000008 | PC\_08DGTB | PC\_000008 | PC\_08DGTB | 99.85 | 1.500000e-27 | 235.23 | 309692 | 0.110647 | 0.756 | 0.775 |
| 4 | PC\_000008 | PC\_01CJ1V | PC\_000008 | PC\_01CJ1V | 99.74 | 1.300000e-23 | 200.18 | 225585 | 0.108380 | 0.504 | 0.743 |

In [4]:

```
pairs.columns = ["minid", "maxid", "lnorm_score", "rnorm_score", "qcov_maxid", "qcov_minid", "tcov_minid", "tcov_maxid"]
pairs.reset_index(drop=True, inplace=True)

pairs["score"] = pairs[["lnorm_score", "rnorm_score"]].mean(axis=1)
pairs["score"] = pairs["score"] / pairs["score"].max()

pairs["minid_cov"] = pairs[["qcov_minid", "tcov_minid"]].mean(axis=1)
pairs["maxid_cov"] = pairs[["qcov_maxid", "tcov_maxid"]].mean(axis=1)

pairs.drop(
    columns=["lnorm_score", "rnorm_score", "qcov_maxid",
             "qcov_minid", "tcov_minid", "tcov_maxid"],
    inplace=True
)

pairs.head()
```

Out[4]:

|  | minid | maxid | score | minid\_cov | maxid\_cov |
| --- | --- | --- | --- | --- | --- |
| 0 | PC\_000008 | PC\_001ISM | 0.098257 | 0.1150 | 0.9560 |
| 1 | PC\_000008 | PC\_0038EV | 0.096670 | 0.3470 | 0.8220 |
| 2 | PC\_000008 | PC\_005RBZ | 0.087706 | 0.4390 | 0.7395 |
| 3 | PC\_000008 | PC\_006QGB | 0.085469 | 0.1895 | 0.8700 |
| 4 | PC\_000008 | PC\_008ZRK | 0.077100 | 0.1140 | 0.9650 |

In [5]:

```
del alignments
```

I'll give each node a numerical id so that we can save a bit of ram.

In [6]:

```
ID_TO_INT = {
    j: i for i, j
    in enumerate(np.unique(np.sort(pairs[["minid", "maxid"]].values.flatten())))
}

INT_TO_ID = np.empty(len(ID_TO_INT), dtype="U9")
for k, v in ID_TO_INT.items():
    INT_TO_ID[v] = k
```

In [7]:

```
pairs["minid_int"] = pairs["minid"].apply(ID_TO_INT.get)
pairs["maxid_int"] = pairs["maxid"].apply(ID_TO_INT.get)
pairs.head()
```

Out[7]:

|  | minid | maxid | score | minid\_cov | maxid\_cov | minid\_int | maxid\_int |
| --- | --- | --- | --- | --- | --- | --- | --- |
| 0 | PC\_000008 | PC\_001ISM | 0.098257 | 0.1150 | 0.9560 | 0 | 1414 |
| 1 | PC\_000008 | PC\_0038EV | 0.096670 | 0.3470 | 0.8220 | 0 | 3084 |
| 2 | PC\_000008 | PC\_005RBZ | 0.087706 | 0.4390 | 0.7395 | 0 | 5514 |
| 3 | PC\_000008 | PC\_006QGB | 0.085469 | 0.1895 | 0.8700 | 0 | 6434 |
| 4 | PC\_000008 | PC\_008ZRK | 0.077100 | 0.1140 | 0.9650 | 0 | 8615 |

## Markov clustering¶

To find more remote homologs, we can relax the requirements having direct edges connecting members.
First we'll look at connected components.

In [8]:

```
matrix = sp.sparse.lil_matrix(
    (len(INT_TO_ID), len(INT_TO_ID)),
    dtype="float32"
)

matrix[pairs["minid_int"].values, pairs["maxid_int"].values] = pairs["score"].values
matrix[pairs["maxid_int"].values, pairs["minid_int"].values] = pairs["score"].values

matrix = matrix.tocsr()
```

In [12]:

```
G = nx.from_scipy_sparse_matrix(matrix)
ccs = list(nx.connected_components(G))
```

In [13]:

```
len(ccs)
```

Out[13]:

```
163
```

In [14]:

```
sorted((len(c) for c in ccs), reverse=True)[:10]
```

Out[14]:

```
[282333, 233, 54, 41, 37, 37, 33, 33, 29, 29]
```

Ok. Well we have one really big connected component.
The other smaller ones would probably be useful.

We'll use the optimised inflation value from the full length clustering.

In [15]:

```
mcl_result = mc.run_mcl(matrix, inflation=1.35, expansion=2)
mcl_clusters = mc.get_clusters(mcl_result)

test_clusters = sorted(mcl_clusters, reverse=True, key=lambda x: len(x))
len(test_clusters)
```

Out[15]:

```
4857
```

In [16]:

```
[len(x) for x in test_clusters][:5]
```

Out[16]:

```
[6874, 5700, 4485, 3879, 2901]
```

Now I need to join these clusters with the effector information, and write out a graph file with that information that I can use to visualise.

In [27]:

```
nodes = {i: {"node_id": id_, "node_int": i} for i, id_ in enumerate(INT_TO_ID)}
```

In [28]:

```
n = pd.concat([
    pairs[["minid_int", "maxid_int"]].rename(columns={"minid_int": "node", "maxid_int": "neighbor"}),
    pairs[["maxid_int", "minid_int"]].rename(columns={"maxid_int": "node", "minid_int": "neighbor"}),
]).drop_duplicates()

n = n.groupby("node")["neighbor"].nunique()
n = n.reset_index().rename(columns={"neighbor": "nedges"})
n.sort_values("nedges", inplace=True, ascending=False, ignore_index=True)

n
```

Out[28]:

|  | node | nedges |
| --- | --- | --- |
| 0 | 1460 | 3717 |
| 1 | 48297 | 3370 |
| 2 | 243090 | 3263 |
| 3 | 71601 | 3021 |
| 4 | 133499 | 2918 |
| ... | ... | ... |
| 283663 | 174827 | 1 |
| 283664 | 69722 | 1 |
| 283665 | 229174 | 1 |
| 283666 | 69813 | 1 |
| 283667 | 69804 | 1 |

283668 rows × 2 columns

In [29]:

```
for i, row in n.iterrows():
    nodes[row["node"]]["nedges"] = row["nedges"]
```

In [30]:

```
for mcl in mcl_clusters:
    members = list()
    for n in mcl:
        members.append(nodes[n])
    
    members.sort(key=lambda x: x["nedges"], reverse=True)
    assert len(members) > 0, members
    rep = members[0]["node_id"]
    for m in members:
        m["supercluster_markov"] = rep
```

In [31]:

```
nodes
```

Out[31]:

```
{0: {'node_id': 'PC_000008',
  'node_int': 0,
  'nedges': 50,
  'supercluster_markov': 'PC_05C4OY'},
 1: {'node_id': 'PC_00000G',
  'node_int': 1,
  'nedges': 57,
  'supercluster_markov': 'PC_03EK7O'},
 2: {'node_id': 'PC_00001B',
  'node_int': 2,
  'nedges': 97,
  'supercluster_markov': 'PC_03HSAJ'},
 3: {'node_id': 'PC_00002Z',
  'node_int': 3,
  'nedges': 58,
  'supercluster_markov': 'PC_002AG8'},
 4: {'node_id': 'PC_000033',
  'node_int': 4,
  'nedges': 101,
  'supercluster_markov': 'PC_06GTNH'},
 5: {'node_id': 'PC_000035',
  'node_int': 5,
  'nedges': 50,
  'supercluster_markov': 'PC_02HOCX'},
 6: {'node_id': 'PC_00004I',
  'node_int': 6,
  'nedges': 61,
  'supercluster_markov': 'PC_08I4HF'},
 7: {'node_id': 'PC_000054',
  'node_int': 7,
  'nedges': 125,
  'supercluster_markov': 'PC_019LQZ'},
 8: {'node_id': 'PC_000089',
  'node_int': 8,
  'nedges': 4,
  'supercluster_markov': 'PC_000089'},
 9: {'node_id': 'PC_0000B2',
  'node_int': 9,
  'nedges': 52,
  'supercluster_markov': 'PC_01VCHX'},
 10: {'node_id': 'PC_0000B5',
  'node_int': 10,
  'nedges': 71,
  'supercluster_markov': 'PC_07NEQU'},
 11: {'node_id': 'PC_0000BT',
  'node_int': 11,
  'nedges': 50,
  'supercluster_markov': 'PC_04TBA7'},
 12: {'node_id': 'PC_0000C1',
  'node_int': 12,
  'nedges': 64,
  'supercluster_markov': 'PC_0222WR'},
 13: {'node_id': 'PC_0000D7',
  'node_int': 13,
  'nedges': 50,
  'supercluster_markov': 'PC_063FB2'},
 14: {'node_id': 'PC_0000DZ',
  'node_int': 14,
  'nedges': 114,
  'supercluster_markov': 'PC_012DSI'},
 15: {'node_id': 'PC_0000EM',
  'node_int': 15,
  'nedges': 50,
  'supercluster_markov': 'PC_02XMPO'},
 16: {'node_id': 'PC_0000FD',
  'node_int': 16,
  'nedges': 52,
  'supercluster_markov': 'PC_016BFH'},
 17: {'node_id': 'PC_0000FI',
  'node_int': 17,
  'nedges': 3,
  'supercluster_markov': 'PC_05MWAZ'},
 18: {'node_id': 'PC_0000FW',
  'node_int': 18,
  'nedges': 98,
  'supercluster_markov': 'PC_048DCU'},
 19: {'node_id': 'PC_0000HW',
  'node_int': 19,
  'nedges': 50,
  'supercluster_markov': 'PC_04VQKU'},
 20: {'node_id': 'PC_0000JH',
  'node_int': 20,
  'nedges': 82,
  'supercluster_markov': 'PC_023OPN'},
 21: {'node_id': 'PC_0000MN',
  'node_int': 21,
  'nedges': 50,
  'supercluster_markov': 'PC_044XWJ'},
 22: {'node_id': 'PC_0000PW',
  'node_int': 22,
  'nedges': 50,
  'supercluster_markov': 'PC_05MOBP'},
 23: {'node_id': 'PC_0000Q3',
  'node_int': 23,
  'nedges': 50,
  'supercluster_markov': 'PC_01OCQ4'},
 24: {'node_id': 'PC_0000RJ',
  'node_int': 24,
  'nedges': 51,
  'supercluster_markov': 'PC_05VP7B'},
 25: {'node_id': 'PC_0000RV',
  'node_int': 25,
  'nedges': 61,
  'supercluster_markov': 'PC_01XK9I'},
 26: {'node_id': 'PC_0000RZ',
  'node_int': 26,
  'nedges': 96,
  'supercluster_markov': 'PC_004F9S'},
 27: {'node_id': 'PC_0000SB',
  'node_int': 27,
  'nedges': 52,
  'supercluster_markov': 'PC_02XYVD'},
 28: {'node_id': 'PC_0000SD',
  'node_int': 28,
  'nedges': 73,
  'supercluster_markov': 'PC_002AGF'},
 29: {'node_id': 'PC_0000SJ',
  'node_int': 29,
  'nedges': 76,
  'supercluster_markov': 'PC_063MEP'},
 30: {'node_id': 'PC_0000SR',
  'node_int': 30,
  'nedges': 51,
  'supercluster_markov': 'PC_04Z1TZ'},
 31: {'node_id': 'PC_0000ST',
  'node_int': 31,
  'nedges': 50,
  'supercluster_markov': 'PC_05JKKU'},
 32: {'node_id': 'PC_0000V1',
  'node_int': 32,
  'nedges': 51,
  'supercluster_markov': 'PC_06I84L'},
 33: {'node_id': 'PC_0000V7',
  'node_int': 33,
  'nedges': 59,
  'supercluster_markov': 'PC_05JLZ9'},
 34: {'node_id': 'PC_0000VE',
  'node_int': 34,
  'nedges': 6,
  'supercluster_markov': 'PC_043DQB'},
 35: {'node_id': 'PC_0000VT',
  'node_int': 35,
  'nedges': 157,
  'supercluster_markov': 'PC_04Z1TZ'},
 36: {'node_id': 'PC_0000XH',
  'node_int': 36,
  'nedges': 246,
  'supercluster_markov': 'PC_030X3X'},
 37: {'node_id': 'PC_0000XZ',
  'node_int': 37,
  'nedges': 51,
  'supercluster_markov': 'PC_04SOJA'},
 38: {'node_id': 'PC_0000ZK',
  'node_int': 38,
  'nedges': 58,
  'supercluster_markov': 'PC_03WKSI'},
 39: {'node_id': 'PC_000100',
  'node_int': 39,
  'nedges': 50,
  'supercluster_markov': 'PC_017I2K'},
 40: {'node_id': 'PC_00011X',
  'node_int': 40,
  'nedges': 88,
  'supercluster_markov': 'PC_0172Q2'},
 41: {'node_id': 'PC_000120',
  'node_int': 41,
  'nedges': 44,
  'supercluster_markov': 'PC_04VDKZ'},
 42: {'node_id': 'PC_00012H',
  'node_int': 42,
  'nedges': 54,
  'supercluster_markov': 'PC_000JRF'},
 43: {'node_id': 'PC_00012N',
  'node_int': 43,
  'nedges': 193,
  'supercluster_markov': 'PC_0172Q2'},
 44: {'node_id': 'PC_000145',
  'node_int': 44,
  'nedges': 155,
  'supercluster_markov': 'PC_03VYM3'},
 45: {'node_id': 'PC_000178',
  'node_int': 45,
  'nedges': 75,
  'supercluster_markov': 'PC_06LBG4'},
 46: {'node_id': 'PC_0001BE',
  'node_int': 46,
  'nedges': 53,
  'supercluster_markov': 'PC_00M20X'},
 47: {'node_id': 'PC_0001BP',
  'node_int': 47,
  'nedges': 32,
  'supercluster_markov': 'PC_007HXL'},
 48: {'node_id': 'PC_0001CV',
  'node_int': 48,
  'nedges': 52,
  'supercluster_markov': 'PC_05GW50'},
 49: {'node_id': 'PC_0001FZ',
  'node_int': 49,
  'nedges': 441,
  'supercluster_markov': 'PC_05MOBP'},
 50: {'node_id': 'PC_0001H6',
  'node_int': 50,
  'nedges': 207,
  'supercluster_markov': 'PC_0454I3'},
 51: {'node_id': 'PC_0001KX',
  'node_int': 51,
  'nedges': 50,
  'supercluster_markov': 'PC_02CE2K'},
 52: {'node_id': 'PC_0001N0',
  'node_int': 52,
  'nedges': 50,
  'supercluster_markov': 'PC_065PL9'},
 53: {'node_id': 'PC_0001OD',
  'node_int': 53,
  'nedges': 58,
  'supercluster_markov': 'PC_017I2K'},
 54: {'node_id': 'PC_0001P3',
  'node_int': 54,
  'nedges': 50,
  'supercluster_markov': 'PC_063MEP'},
 55: {'node_id': 'PC_0001PA',
  'node_int': 55,
  'nedges': 1,
  'supercluster_markov': 'PC_0001PA'},
 56: {'node_id': 'PC_0001T9',
  'node_int': 56,
  'nedges': 57,
  'supercluster_markov': 'PC_02MX89'},
 57: {'node_id': 'PC_0001TX',
  'node_int': 57,
  'nedges': 53,
  'supercluster_markov': 'PC_0229E6'},
 58: {'node_id': 'PC_0001V5',
  'node_int': 58,
  'nedges': 50,
  'supercluster_markov': 'PC_06RBCY'},
 59: {'node_id': 'PC_0001VI',
  'node_int': 59,
  'nedges': 164,
  'supercluster_markov': 'PC_01L48J'},
 60: {'node_id': 'PC_0001VZ',
  'node_int': 60,
  'nedges': 50,
  'supercluster_markov': 'PC_003H85'},
 61: {'node_id': 'PC_0001WS',
  'node_int': 61,
  'nedges': 50,
  'supercluster_markov': 'PC_05EWXT'},
 62: {'node_id': 'PC_00020H',
  'node_int': 62,
  'nedges': 67,
  'supercluster_markov': 'PC_03ENV6'},
 63: {'node_id': 'PC_000210',
  'node_int': 63,
  'nedges': 245,
  'supercluster_markov': 'PC_04Z1TZ'},
 64: {'node_id': 'PC_00021B',
  'node_int': 64,
  'nedges': 79,
  'supercluster_markov': 'PC_02XMPO'},
 65: {'node_id': 'PC_00021R',
  'node_int': 65,
  'nedges': 50,
  'supercluster_markov': 'PC_03ODP1'},
 66: {'node_id': 'PC_00021Y',
  'node_int': 66,
  'nedges': 93,
  'supercluster_markov': 'PC_04GVYT'},
 67: {'node_id': 'PC_00023X',
  'node_int': 67,
  'nedges': 68,
  'supercluster_markov': 'PC_05PAWE'},
 68: {'node_id': 'PC_00024N',
  'node_int': 68,
  'nedges': 56,
  'supercluster_markov': 'PC_0222WR'},
 69: {'node_id': 'PC_00024X',
  'node_int': 69,
  'nedges': 31,
  'supercluster_markov': 'PC_03F28A'},
 70: {'node_id': 'PC_00025M',
  'node_int': 70,
  'nedges': 50,
  'supercluster_markov': 'PC_03S162'},
 71: {'node_id': 'PC_00028F',
  'node_int': 71,
  'nedges': 74,
  'supercluster_markov': 'PC_02B4A4'},
 72: {'node_id': 'PC_00028Q',
  'node_int': 72,
  'nedges': 121,
  'supercluster_markov': 'PC_01MK5F'},
 73: {'node_id': 'PC_0002B2',
  'node_int': 73,
  'nedges': 67,
  'supercluster_markov': 'PC_04ZPF1'},
 74: {'node_id': 'PC_0002DL',
  'node_int': 74,
  'nedges': 51,
  'supercluster_markov': 'PC_03D1TN'},
 75: {'node_id': 'PC_0002DN',
  'node_int': 75,
  'nedges': 54,
  'supercluster_markov': 'PC_019LQZ'},
 76: {'node_id': 'PC_0002F9',
  'node_int': 76,
  'nedges': 63,
  'supercluster_markov': 'PC_01T9YZ'},
 77: {'node_id': 'PC_0002FN',
  'node_int': 77,
  'nedges': 10,
  'supercluster_markov': 'PC_01BG6A'},
 78: {'node_id': 'PC_0002G0',
  'node_int': 78,
  'nedges': 50,
  'supercluster_markov': 'PC_04LDFD'},
 79: {'node_id': 'PC_0002G4',
  'node_int': 79,
  'nedges': 50,
  'supercluster_markov': 'PC_060MBL'},
 80: {'node_id': 'PC_0002GL',
  'node_int': 80,
  'nedges': 69,
  'supercluster_markov': 'PC_04ZPF1'},
 81: {'node_id': 'PC_0002IT',
  'node_int': 81,
  'nedges': 98,
  'supercluster_markov': 'PC_04GLN4'},
 82: {'node_id': 'PC_0002JA',
  'node_int': 82,
  'nedges': 66,
  'supercluster_markov': 'PC_01F9UZ'},
 83: {'node_id': 'PC_0002K6',
  'node_int': 83,
  'nedges': 50,
  'supercluster_markov': 'PC_04G5AJ'},
 84: {'node_id': 'PC_0002KO',
  'node_int': 84,
  'nedges': 57,
  'supercluster_markov': 'PC_056DG4'},
 85: {'node_id': 'PC_0002L7',
  'node_int': 85,
  'nedges': 67,
  'supercluster_markov': 'PC_01P6AC'},
 86: {'node_id': 'PC_0002LX',
  'node_int': 86,
  'nedges': 66,
  'supercluster_markov': 'PC_004XDC'},
 87: {'node_id': 'PC_0002MN',
  'node_int': 87,
  'nedges': 65,
  'supercluster_markov': 'PC_07RDG0'},
 88: {'node_id': 'PC_0002O6',
  'node_int': 88,
  'nedges': 50,
  'supercluster_markov': 'PC_04LPRM'},
 89: {'node_id': 'PC_0002OA',
  'node_int': 89,
  'nedges': 56,
  'supercluster_markov': 'PC_00C81O'},
 90: {'node_id': 'PC_0002Q8',
  'node_int': 90,
  'nedges': 143,
  'supercluster_markov': 'PC_07NEQU'},
 91: {'node_id': 'PC_0002QB',
  'node_int': 91,
  'nedges': 50,
  'supercluster_markov': 'PC_00RPR3'},
 92: {'node_id': 'PC_0002QE',
  'node_int': 92,
  'nedges': 50,
  'supercluster_markov': 'PC_04KZQH'},
 93: {'node_id': 'PC_0002R0',
  'node_int': 93,
  'nedges': 130,
  'supercluster_markov': 'PC_08GZ9T'},
 94: {'node_id': 'PC_0002S0',
  'node_int': 94,
  'nedges': 51,
  'supercluster_markov': 'PC_08H7YK'},
 95: {'node_id': 'PC_0002TR',
  'node_int': 95,
  'nedges': 50,
  'supercluster_markov': 'PC_04Z1TZ'},
 96: {'node_id': 'PC_0002U1',
  'node_int': 96,
  'nedges': 50,
  'supercluster_markov': 'PC_00YLBJ'},
 97: {'node_id': 'PC_0002UJ',
  'node_int': 97,
  'nedges': 6,
  'supercluster_markov': 'PC_00AYEN'},
 98: {'node_id': 'PC_0002UQ',
  'node_int': 98,
  'nedges': 94,
  'supercluster_markov': 'PC_046DTC'},
 99: {'node_id': 'PC_0002VZ',
  'node_int': 99,
  'nedges': 51,
  'supercluster_markov': 'PC_03S7G4'},
 100: {'node_id': 'PC_0002X5',
  'node_int': 100,
  'nedges': 125,
  'supercluster_markov': 'PC_08GABV'},
 101: {'node_id': 'PC_0002XW',
  'node_int': 101,
  'nedges': 54,
  'supercluster_markov': 'PC_0738R9'},
 102: {'node_id': 'PC_0002Z4',
  'node_int': 102,
  'nedges': 10,
  'supercluster_markov': 'PC_0002Z4'},
 103: {'node_id': 'PC_0002ZO',
  'node_int': 103,
  'nedges': 67,
  'supercluster_markov': 'PC_01JMIR'},
 104: {'node_id': 'PC_00030B',
  'node_int': 104,
  'nedges': 52,
  'supercluster_markov': 'PC_06Q9T1'},
 105: {'node_id': 'PC_00030S',
  'node_int': 105,
  'nedges': 117,
  'supercluster_markov': 'PC_05D152'},
 106: {'node_id': 'PC_00031G',
  'node_int': 106,
  'nedges': 50,
  'supercluster_markov': 'PC_04YDES'},
 107: {'node_id': 'PC_00031N',
  'node_int': 107,
  'nedges': 173,
  'supercluster_markov': 'PC_04G5AJ'},
 108: {'node_id': 'PC_00032X',
  'node_int': 108,
  'nedges': 17,
  'supercluster_markov': 'PC_00032X'},
 109: {'node_id': 'PC_00033Y',
  'node_int': 109,
  'nedges': 61,
  'supercluster_markov': 'PC_04FX6B'},
 110: {'node_id': 'PC_000351',
  'node_int': 110,
  'nedges': 56,
  'supercluster_markov': 'PC_019S0D'},
 111: {'node_id': 'PC_000358',
  'node_int': 111,
  'nedges': 37,
  'supercluster_markov': 'PC_05MDOF'},
 112: {'node_id': 'PC_00035V',
  'node_int': 112,
  'nedges': 48,
  'supercluster_markov': 'PC_00L6KL'},
 113: {'node_id': 'PC_00035Y',
  'node_int': 113,
  'nedges': 50,
  'supercluster_markov': 'PC_018SM6'},
 114: {'node_id': 'PC_00036G',
  'node_int': 114,
  'nedges': 87,
  'supercluster_markov': 'PC_0738R9'},
 115: {'node_id': 'PC_00036I',
  'node_int': 115,
  'nedges': 54,
  'supercluster_markov': 'PC_018Q71'},
 116: {'node_id': 'PC_0003E4',
  'node_int': 116,
  'nedges': 72,
  'supercluster_markov': 'PC_06I84L'},
 117: {'node_id': 'PC_0003E9',
  'node_int': 117,
  'nedges': 61,
  'supercluster_markov': 'PC_03O094'},
 118: {'node_id': 'PC_0003EE',
  'node_int': 118,
  'nedges': 50,
  'supercluster_markov': 'PC_0229E6'},
 119: {'node_id': 'PC_0003FQ',
  'node_int': 119,
  'nedges': 57,
  'supercluster_markov': 'PC_05GH8G'},
 120: {'node_id': 'PC_0003GX',
  'node_int': 120,
  'nedges': 119,
  'supercluster_markov': 'PC_020YH4'},
 121: {'node_id': 'PC_0003J3',
  'node_int': 121,
  'nedges': 50,
  'supercluster_markov': 'PC_04YDES'},
 122: {'node_id': 'PC_0003K7',
  'node_int': 122,
  'nedges': 69,
  'supercluster_markov': 'PC_06SWHR'},
 123: {'node_id': 'PC_0003L7',
  'node_int': 123,
  'nedges': 96,
  'supercluster_markov': 'PC_05CIT8'},
 124: {'node_id': 'PC_0003LA',
  'node_int': 124,
  'nedges': 110,
  'supercluster_markov': 'PC_03NXOX'},
 125: {'node_id': 'PC_0003LF',
  'node_int': 125,
  'nedges': 59,
  'supercluster_markov': 'PC_048DCU'},
 126: {'node_id': 'PC_0003NK',
  'node_int': 126,
  'nedges': 50,
  'supercluster_markov': 'PC_05FEUB'},
 127: {'node_id': 'PC_0003OB',
  'node_int': 127,
  'nedges': 158,
  'supercluster_markov': 'PC_00G7DT'},
 128: {'node_id': 'PC_0003UA',
  'node_int': 128,
  'nedges': 109,
  'supercluster_markov': 'PC_04DVGJ'},
 129: {'node_id': 'PC_0003WZ',
  'node_int': 129,
  'nedges': 69,
  'supercluster_markov': 'PC_01JBJM'},
 130: {'node_id': 'PC_0003XI',
  'node_int': 130,
  'nedges': 42,
  'supercluster_markov': 'PC_01STXZ'},
 131: {'node_id': 'PC_0003XZ',
  'node_int': 131,
  'nedges': 76,
  'supercluster_markov': 'PC_0003XZ'},
 132: {'node_id': 'PC_0003ZC',
  'node_int': 132,
  'nedges': 131,
  'supercluster_markov': 'PC_00YLBJ'},
 133: {'node_id': 'PC_0003ZL',
  'node_int': 133,
  'nedges': 63,
  'supercluster_markov': 'PC_01D6DG'},
 134: {'node_id': 'PC_00042X',
  'node_int': 134,
  'nedges': 63,
  'supercluster_markov': 'PC_04ZPF1'},
 135: {'node_id': 'PC_000467',
  'node_int': 135,
  'nedges': 66,
  'supercluster_markov': 'PC_086AR6'},
 136: {'node_id': 'PC_00047U',
  'node_int': 136,
  'nedges': 54,
  'supercluster_markov': 'PC_06OA4L'},
 137: {'node_id': 'PC_00048X',
  'node_int': 137,
  'nedges': 65,
  'supercluster_markov': 'PC_02G17U'},
 138: {'node_id': 'PC_0004B1',
  'node_int': 138,
  'nedges': 134,
  'supercluster_markov': 'PC_0519Z5'},
 139: {'node_id': 'PC_0004BP',
  'node_int': 139,
  'nedges': 5,
  'supercluster_markov': 'PC_06F9DN'},
 140: {'node_id': 'PC_0004D5',
  'node_int': 140,
  'nedges': 103,
  'supercluster_markov': 'PC_0519Z5'},
 141: {'node_id': 'PC_0004DI',
  'node_int': 141,
  'nedges': 50,
  'supercluster_markov': 'PC_04FCEG'},
 142: {'node_id': 'PC_0004FU',
  'node_int': 142,
  'nedges': 59,
  'supercluster_markov': 'PC_034NST'},
 143: {'node_id': 'PC_0004IN',
  'node_int': 143,
  'nedges': 72,
  'supercluster_markov': 'PC_01LNCE'},
 144: {'node_id': 'PC_0004IW',
  'node_int': 144,
  'nedges': 61,
  'supercluster_markov': 'PC_0519Z5'},
 145: {'node_id': 'PC_0004JK',
  'node_int': 145,
  'nedges': 56,
  'supercluster_markov': 'PC_065ZKY'},
 146: {'node_id': 'PC_0004KJ',
  'node_int': 146,
  'nedges': 91,
  'supercluster_markov': 'PC_065PL9'},
 147: {'node_id': 'PC_0004KY',
  'node_int': 147,
  'nedges': 60,
  'supercluster_markov': 'PC_03GXDV'},
 148: {'node_id': 'PC_0004NA',
  'node_int': 148,
  'nedges': 1,
  'supercluster_markov': 'PC_05D9HF'},
 149: {'node_id': 'PC_0004PA',
  'node_int': 149,
  'nedges': 50,
  'supercluster_markov': 'PC_0861TC'},
 150: {'node_id': 'PC_0004PE',
  'node_int': 150,
  'nedges': 51,
  'supercluster_markov': 'PC_001KCV'},
 151: {'node_id': 'PC_0004PU',
  'node_int': 151,
  'nedges': 68,
  'supercluster_markov': 'PC_075ULT'},
 152: {'node_id': 'PC_0004Q8',
  'node_int': 152,
  'nedges': 64,
  'supercluster_markov': 'PC_00EXEJ'},
 153: {'node_id': 'PC_0004QS',
  'node_int': 153,
  'nedges': 89,
  'supercluster_markov': 'PC_02III9'},
 154: {'node_id': 'PC_0004SG',
  'node_int': 154,
  'nedges': 82,
  'supercluster_markov': 'PC_056GI0'},
 155: {'node_id': 'PC_0004X5',
  'node_int': 155,
  'nedges': 88,
  'supercluster_markov': 'PC_04Z1TZ'},
 156: {'node_id': 'PC_0004Y9',
  'node_int': 156,
  'nedges': 64,
  'supercluster_markov': 'PC_02GINI'},
 157: {'node_id': 'PC_0004YB',
  'node_int': 157,
  'nedges': 79,
  'supercluster_markov': 'PC_00L6KL'},
 158: {'node_id': 'PC_0004YE',
  'node_int': 158,
  'nedges': 85,
  'supercluster_markov': 'PC_004XDC'},
 159: {'node_id': 'PC_0004Z1',
  'node_int': 159,
  'nedges': 50,
  'supercluster_markov': 'PC_04Z1TZ'},
 160: {'node_id': 'PC_00051P',
  'node_int': 160,
  'nedges': 51,
  'supercluster_markov': 'PC_010T6X'},
 161: {'node_id': 'PC_00052H',
  'node_int': 161,
  'nedges': 68,
  'supercluster_markov': 'PC_0229E6'},
 162: {'node_id': 'PC_00052X',
  'node_int': 162,
  'nedges': 56,
  'supercluster_markov': 'PC_040LIN'},
 163: {'node_id': 'PC_000546',
  'node_int': 163,
  'nedges': 56,
  'supercluster_markov': 'PC_04SRDC'},
 164: {'node_id': 'PC_00054T',
  'node_int': 164,
  'nedges': 55,
  'supercluster_markov': 'PC_04Z1TZ'},
 165: {'node_id': 'PC_00057G',
  'node_int': 165,
  'nedges': 53,
  'supercluster_markov': 'PC_027WLR'},
 166: {'node_id': 'PC_0005AJ',
  'node_int': 166,
  'nedges': 48,
  'supercluster_markov': 'PC_01K8SG'},
 167: {'node_id': 'PC_0005BF',
  'node_int': 167,
  'nedges': 75,
  'supercluster_markov': 'PC_03L3BY'},
 168: {'node_id': 'PC_0005BJ',
  'node_int': 168,
  'nedges': 52,
  'supercluster_markov': 'PC_08CJSM'},
 169: {'node_id': 'PC_0005BX',
  'node_int': 169,
  'nedges': 37,
  'supercluster_markov': 'PC_08KGXK'},
 170: {'node_id': 'PC_0005CY',
  'node_int': 170,
  'nedges': 30,
  'supercluster_markov': 'PC_0824YU'},
 171: {'node_id': 'PC_0005FN',
  'node_int': 171,
  'nedges': 50,
  'supercluster_markov': 'PC_013S4V'},
 172: {'node_id': 'PC_0005KU',
  'node_int': 172,
  'nedges': 51,
  'supercluster_markov': 'PC_03HDK3'},
 173: {'node_id': 'PC_0005MO',
  'node_int': 173,
  'nedges': 51,
  'supercluster_markov': 'PC_02GINI'},
 174: {'node_id': 'PC_0005MR',
  'node_int': 174,
  'nedges': 50,
  'supercluster_markov': 'PC_02OA60'},
 175: {'node_id': 'PC_0005ND',
  'node_int': 175,
  'nedges': 51,
  'supercluster_markov': 'PC_03NTZD'},
 176: {'node_id': 'PC_0005NG',
  'node_int': 176,
  'nedges': 50,
  'supercluster_markov': 'PC_06F4U8'},
 177: {'node_id': 'PC_0005P0',
  'node_int': 177,
  'nedges': 81,
  'supercluster_markov': 'PC_055Y72'},
 178: {'node_id': 'PC_0005T5',
  'node_int': 178,
  'nedges': 51,
  'supercluster_markov': 'PC_02VXG7'},
 179: {'node_id': 'PC_0005TC',
  'node_int': 179,
  'nedges': 54,
  'supercluster_markov': 'PC_01RCLA'},
 180: {'node_id': 'PC_0005TJ',
  'node_int': 180,
  'nedges': 10,
  'supercluster_markov': 'PC_0029MW'},
 181: {'node_id': 'PC_0005WL',
  'node_int': 181,
  'nedges': 51,
  'supercluster_markov': 'PC_009P46'},
 182: {'node_id': 'PC_0005WO',
  'node_int': 182,
  'nedges': 34,
  'supercluster_markov': 'PC_037PVL'},
 183: {'node_id': 'PC_00061S',
  'node_int': 183,
  'nedges': 28,
  'supercluster_markov': 'PC_04HPIB'},
 184: {'node_id': 'PC_000628',
  'node_int': 184,
  'nedges': 51,
  'supercluster_markov': 'PC_02GINI'},
 185: {'node_id': 'PC_00065H',
  'node_int': 185,
  'nedges': 78,
  'supercluster_markov': 'PC_032Q1F'},
 186: {'node_id': 'PC_00068Y',
  'node_int': 186,
  'nedges': 51,
  'supercluster_markov': 'PC_03QZRR'},
 187: {'node_id': 'PC_0006A4',
  'node_int': 187,
  'nedges': 50,
  'supercluster_markov': 'PC_04YDES'},
 188: {'node_id': 'PC_0006M1',
  'node_int': 188,
  'nedges': 58,
  'supercluster_markov': 'PC_03S7G4'},
 189: {'node_id': 'PC_0006MJ',
  'node_int': 189,
  'nedges': 50,
  'supercluster_markov': 'PC_0312H6'},
 190: {'node_id': 'PC_0006MU',
  'node_int': 190,
  'nedges': 58,
  'supercluster_markov': 'PC_022MR1'},
 191: {'node_id': 'PC_0006N6',
  'node_int': 191,
  'nedges': 50,
  'supercluster_markov': 'PC_05GT7C'},
 192: {'node_id': 'PC_0006NR',
  'node_int': 192,
  'nedges': 50,
  'supercluster_markov': 'PC_04DVGJ'},
 193: {'node_id': 'PC_0006NZ',
  'node_int': 193,
  'nedges': 50,
  'supercluster_markov': 'PC_04RUJN'},
 194: {'node_id': 'PC_0006OH',
  'node_int': 194,
  'nedges': 56,
  'supercluster_markov': 'PC_03CQ5G'},
 195: {'node_id': 'PC_0006OT',
  'node_int': 195,
  'nedges': 50,
  'supercluster_markov': 'PC_05HBLX'},
 196: {'node_id': 'PC_0006PA',
  'node_int': 196,
  'nedges': 50,
  'supercluster_markov': 'PC_0006PA'},
 197: {'node_id': 'PC_0006QQ',
  'node_int': 197,
  'nedges': 143,
  'supercluster_markov': 'PC_075ULT'},
 198: {'node_id': 'PC_0006S1',
  'node_int': 198,
  'nedges': 51,
  'supercluster_markov': 'PC_04ZPF1'},
 199: {'node_id': 'PC_0006TR',
  'node_int': 199,
  'nedges': 80,
  'supercluster_markov': 'PC_04GLN4'},
 200: {'node_id': 'PC_0006U4',
  'node_int': 200,
  'nedges': 51,
  'supercluster_markov': 'PC_04RUJN'},
 201: {'node_id': 'PC_0006U5',
  'node_int': 201,
  'nedges': 50,
  'supercluster_markov': 'PC_04OW50'},
 202: {'node_id': 'PC_0006U8',
  'node_int': 202,
  'nedges': 51,
  'supercluster_markov': 'PC_009GXC'},
 203: {'node_id': 'PC_0006UN',
  'node_int': 203,
  'nedges': 12,
  'supercluster_markov': 'PC_0006UN'},
 204: {'node_id': 'PC_0006UQ',
  'node_int': 204,
  'nedges': 256,
  'supercluster_markov': 'PC_04RUJN'},
 205: {'node_id': 'PC_0006VB',
  'node_int': 205,
  'nedges': 50,
  'supercluster_markov': 'PC_04RUJN'},
 206: {'node_id': 'PC_0006WK',
  'node_int': 206,
  'nedges': 74,
  'supercluster_markov': 'PC_001ISM'},
 207: {'node_id': 'PC_0006XQ',
  'node_int': 207,
  'nedges': 64,
  'supercluster_markov': 'PC_00Q1IH'},
 208: {'node_id': 'PC_0006YH',
  'node_int': 208,
  'nedges': 22,
  'supercluster_markov': 'PC_03ENDL'},
 209: {'node_id': 'PC_00071M',
  'node_int': 209,
  'nedges': 64,
  'supercluster_markov': 'PC_0544K4'},
 210: {'node_id': 'PC_000722',
  'node_int': 210,
  'nedges': 50,
  'supercluster_markov': 'PC_04YDES'},
 211: {'node_id': 'PC_000726',
  'node_int': 211,
  'nedges': 54,
  'supercluster_markov': 'PC_087NYJ'},
 212: {'node_id': 'PC_00074W',
  'node_int': 212,
  'nedges': 119,
  'supercluster_markov': 'PC_00074W'},
 213: {'node_id': 'PC_00076J',
  'node_int': 213,
  'nedges': 18,
  'supercluster_markov': 'PC_05K6RJ'},
 214: {'node_id': 'PC_000799',
  'node_int': 214,
  'nedges': 57,
  'supercluster_markov': 'PC_03S7G4'},
 215: {'node_id': 'PC_00079B',
  'node_int': 215,
  'nedges': 11,
  'supercluster_markov': 'PC_00079B'},
 216: {'node_id': 'PC_00079M',
  'node_int': 216,
  'nedges': 50,
  'supercluster_markov': 'PC_00THHH'},
 217: {'node_id': 'PC_0007A2',
  'node_int': 217,
  'nedges': 115,
  'supercluster_markov': 'PC_0378ND'},
 218: {'node_id': 'PC_0007A4',
  'node_int': 218,
  'nedges': 109,
  'supercluster_markov': 'PC_02I8J5'},
 219: {'node_id': 'PC_0007A5',
  'node_int': 219,
  'nedges': 54,
  'supercluster_markov': 'PC_04VPV5'},
 220: {'node_id': 'PC_0007AQ',
  'node_int': 220,
  'nedges': 1028,
  'supercluster_markov': 'PC_0229E6'},
 221: {'node_id': 'PC_0007B2',
  'node_int': 221,
  'nedges': 51,
  'supercluster_markov': 'PC_0519Z5'},
 222: {'node_id': 'PC_0007BP',
  'node_int': 222,
  'nedges': 200,
  'supercluster_markov': 'PC_0229E6'},
 223: {'node_id': 'PC_0007C1',
  'node_int': 223,
  'nedges': 50,
  'supercluster_markov': 'PC_0483K9'},
 224: {'node_id': 'PC_0007E6',
  'node_int': 224,
  'nedges': 50,
  'supercluster_markov': 'PC_041LAD'},
 225: {'node_id': 'PC_0007FR',
  'node_int': 225,
  'nedges': 60,
  'supercluster_markov': 'PC_04SWP7'},
 226: {'node_id': 'PC_0007H1',
  'node_int': 226,
  'nedges': 57,
  'supercluster_markov': 'PC_07BJIL'},
 227: {'node_id': 'PC_0007KE',
  'node_int': 227,
  'nedges': 50,
  'supercluster_markov': 'PC_06I84L'},
 228: {'node_id': 'PC_0007KZ',
  'node_int': 228,
  'nedges': 53,
  'supercluster_markov': 'PC_056GI0'},
 229: {'node_id': 'PC_0007OT',
  'node_int': 229,
  'nedges': 51,
  'supercluster_markov': 'PC_02HSBP'},
 230: {'node_id': 'PC_0007S5',
  'node_int': 230,
  'nedges': 97,
  'supercluster_markov': 'PC_003N2B'},
 231: {'node_id': 'PC_0007SN',
  'node_int': 231,
  'nedges': 71,
  'supercluster_markov': 'PC_04XZUL'},
 232: {'node_id': 'PC_0007TE',
  'node_int': 232,
  'nedges': 8,
  'supercluster_markov': 'PC_0270V9'},
 233: {'node_id': 'PC_0007UK',
  'node_int': 233,
  'nedges': 51,
  'supercluster_markov': 'PC_017I2K'},
 234: {'node_id': 'PC_0007W8',
  'node_int': 234,
  'nedges': 53,
  'supercluster_markov': 'PC_06DIZZ'},
 235: {'node_id': 'PC_0007WY',
  'node_int': 235,
  'nedges': 50,
  'supercluster_markov': 'PC_03B9OL'},
 236: {'node_id': 'PC_0007WZ',
  'node_int': 236,
  'nedges': 92,
  'supercluster_markov': 'PC_05GH8G'},
 237: {'node_id': 'PC_0007XV',
  'node_int': 237,
  'nedges': 50,
  'supercluster_markov': 'PC_016G2J'},
 238: {'node_id': 'PC_0007Y0',
  'node_int': 238,
  'nedges': 50,
  'supercluster_markov': 'PC_04G5AJ'},
 239: {'node_id': 'PC_00080U',
  'node_int': 239,
  'nedges': 55,
  'supercluster_markov': 'PC_01V515'},
 240: {'node_id': 'PC_000826',
  'node_int': 240,
  'nedges': 51,
  'supercluster_markov': 'PC_02G2TC'},
 241: {'node_id': 'PC_00083D',
  'node_int': 241,
  'nedges': 31,
  'supercluster_markov': 'PC_01KO6L'},
 242: {'node_id': 'PC_00083G',
  'node_int': 242,
  'nedges': 50,
  'supercluster_markov': 'PC_03HSAJ'},
 243: {'node_id': 'PC_000855',
  'node_int': 243,
  'nedges': 59,
  'supercluster_markov': 'PC_02XEG8'},
 244: {'node_id': 'PC_000856',
  'node_int': 244,
  'nedges': 81,
  'supercluster_markov': 'PC_025SRL'},
 245: {'node_id': 'PC_00089N',
  'node_int': 245,
  'nedges': 59,
  'supercluster_markov': 'PC_00SM5I'},
 246: {'node_id': 'PC_0008AN',
  'node_int': 246,
  'nedges': 70,
  'supercluster_markov': 'PC_013Y2D'},
 247: {'node_id': 'PC_0008B1',
  'node_int': 247,
  'nedges': 82,
  'supercluster_markov': 'PC_07NFD1'},
 248: {'node_id': 'PC_0008CB',
  'node_int': 248,
  'nedges': 62,
  'supercluster_markov': 'PC_034NST'},
 249: {'node_id': 'PC_0008CG',
  'node_int': 249,
  'nedges': 108,
  'supercluster_markov': 'PC_07OBRT'},
 250: {'node_id': 'PC_0008DA',
  'node_int': 250,
  'nedges': 65,
  'supercluster_markov': 'PC_02QK72'},
 251: {'node_id': 'PC_0008FW',
  'node_int': 251,
  'nedges': 74,
  'supercluster_markov': 'PC_017I2K'},
 252: {'node_id': 'PC_0008FZ',
  'node_int': 252,
  'nedges': 50,
  'supercluster_markov': 'PC_041LAD'},
 253: {'node_id': 'PC_0008H8',
  'node_int': 253,
  'nedges': 23,
  'supercluster_markov': 'PC_03J6LS'},
 254: {'node_id': 'PC_0008K4',
  'node_int': 254,
  'nedges': 52,
  'supercluster_markov': 'PC_019E1I'},
 255: {'node_id': 'PC_0008O0',
  'node_int': 255,
  'nedges': 60,
  'supercluster_markov': 'PC_08ICAO'},
 256: {'node_id': 'PC_0008PR',
  'node_int': 256,
  'nedges': 87,
  'supercluster_markov': 'PC_00OZYJ'},
 257: {'node_id': 'PC_0008PS',
  'node_int': 257,
  'nedges': 77,
  'supercluster_markov': 'PC_00NP4B'},
 258: {'node_id': 'PC_0008Q2',
  'node_int': 258,
  'nedges': 50,
  'supercluster_markov': 'PC_01T9YZ'},
 259: {'node_id': 'PC_0008RZ',
  'node_int': 259,
  'nedges': 24,
  'supercluster_markov': 'PC_08JW8U'},
 260: {'node_id': 'PC_0008S8',
  'node_int': 260,
  'nedges': 50,
  'supercluster_markov': 'PC_059NL8'},
 261: {'node_id': 'PC_0008SO',
  'node_int': 261,
  'nedges': 57,
  'supercluster_markov': 'PC_086FA1'},
 262: {'node_id': 'PC_0008SZ',
  'node_int': 262,
  'nedges': 31,
  'supercluster_markov': 'PC_02I8J5'},
 263: {'node_id': 'PC_0008X0',
  'node_int': 263,
  'nedges': 50,
  'supercluster_markov': 'PC_00YLBJ'},
 264: {'node_id': 'PC_0008YT',
  'node_int': 264,
  'nedges': 114,
  'supercluster_markov': 'PC_035MOV'},
 265: {'node_id': 'PC_00091C',
  'node_int': 265,
  'nedges': 50,
  'supercluster_markov': 'PC_04Z1TZ'},
 266: {'node_id': 'PC_000920',
  'node_int': 266,
  'nedges': 112,
  'supercluster_markov': 'PC_044QTV'},
 267: {'node_id': 'PC_00092U',
  'node_int': 267,
  'nedges': 50,
  'supercluster_markov': 'PC_0229E6'},
 268: {'node_id': 'PC_00096F',
  'node_int': 268,
  'nedges': 51,
  'supercluster_markov': 'PC_04K9G7'},
 269: {'node_id': 'PC_000972',
  'node_int': 269,
  'nedges': 87,
  'supercluster_markov': 'PC_011PFM'},
 270: {'node_id': 'PC_000985',
  'node_int': 270,
  'nedges': 54,
  'supercluster_markov': 'PC_040773'},
 271: {'node_id': 'PC_000990',
  'node_int': 271,
  'nedges': 58,
  'supercluster_markov': 'PC_03IVVO'},
 272: {'node_id': 'PC_00099T',
  'node_int': 272,
  'nedges': 71,
  'supercluster_markov': 'PC_011RZ1'},
 273: {'node_id': 'PC_0009BQ',
  'node_int': 273,
  'nedges': 71,
  'supercluster_markov': 'PC_08GZF3'},
 274: {'node_id': 'PC_0009C0',
  'node_int': 274,
  'nedges': 77,
  'supercluster_markov': 'PC_06NSK3'},
 275: {'node_id': 'PC_0009DC',
  'node_int': 275,
  'nedges': 46,
  'supercluster_markov': 'PC_047OWQ'},
 276: {'node_id': 'PC_0009EF',
  'node_int': 276,
  'nedges': 50,
  'supercluster_markov': 'PC_0009EF'},
 277: {'node_id': 'PC_0009EM',
  'node_int': 277,
  'nedges': 74,
  'supercluster_markov': 'PC_0312H6'},
 278: {'node_id': 'PC_0009FH',
  'node_int': 278,
  'nedges': 50,
  'supercluster_markov': 'PC_00TAOF'},
 279: {'node_id': 'PC_0009GP',
  'node_int': 279,
  'nedges': 26,
  'supercluster_markov': 'PC_08JIDK'},
 280: {'node_id': 'PC_0009NO',
  'node_int': 280,
  'nedges': 76,
  'supercluster_markov': 'PC_00TAOF'},
 281: {'node_id': 'PC_0009PC',
  'node_int': 281,
  'nedges': 79,
  'supercluster_markov': 'PC_02GINI'},
 282: {'node_id': 'PC_0009QR',
  'node_int': 282,
  'nedges': 50,
  'supercluster_markov': 'PC_04YDES'},
 283: {'node_id': 'PC_0009RO',
  'node_int': 283,
  'nedges': 193,
  'supercluster_markov': 'PC_0009RO'},
 284: {'node_id': 'PC_0009RS',
  'node_int': 284,
  'nedges': 52,
  'supercluster_markov': 'PC_04Z1TZ'},
 285: {'node_id': 'PC_0009SX',
  'node_int': 285,
  'nedges': 58,
  'supercluster_markov': 'PC_0100QV'},
 286: {'node_id': 'PC_0009TY',
  'node_int': 286,
  'nedges': 69,
  'supercluster_markov': 'PC_03L0DV'},
 287: {'node_id': 'PC_0009TZ',
  'node_int': 287,
  'nedges': 57,
  'supercluster_markov': 'PC_00XHO7'},
 288: {'node_id': 'PC_0009U2',
  'node_int': 288,
  'nedges': 69,
  'supercluster_markov': 'PC_020SRS'},
 289: {'node_id': 'PC_0009UU',
  'node_int': 289,
  'nedges': 54,
  'supercluster_markov': 'PC_03FBY4'},
 290: {'node_id': 'PC_0009WG',
  'node_int': 290,
  'nedges': 122,
  'supercluster_markov': 'PC_0009WG'},
 291: {'node_id': 'PC_0009YU',
  'node_int': 291,
  'nedges': 50,
  'supercluster_markov': 'PC_04ZPF1'},
 292: {'node_id': 'PC_000A02',
  'node_int': 292,
  'nedges': 58,
  'supercluster_markov': 'PC_04DVGJ'},
 293: {'node_id': 'PC_000A06',
  'node_int': 293,
  'nedges': 51,
  'supercluster_markov': 'PC_054B1U'},
 294: {'node_id': 'PC_000A0J',
  'node_int': 294,
  'nedges': 569,
  'supercluster_markov': 'PC_0229E6'},
 295: {'node_id': 'PC_000A0W',
  'node_int': 295,
  'nedges': 172,
  'supercluster_markov': 'PC_02WI85'},
 296: {'node_id': 'PC_000A2T',
  'node_int': 296,
  'nedges': 322,
  'supercluster_markov': 'PC_06UH99'},
 297: {'node_id': 'PC_000A3M',
  'node_int': 297,
  'nedges': 76,
  'supercluster_markov': 'PC_016G2J'},
 298: {'node_id': 'PC_000A3P',
  'node_int': 298,
  'nedges': 57,
  'supercluster_markov': 'PC_02QK72'},
 299: {'node_id': 'PC_000A86',
  'node_int': 299,
  'nedges': 10,
  'supercluster_markov': 'PC_043ORU'},
 300: {'node_id': 'PC_000A8A',
  'node_int': 300,
  'nedges': 50,
  'supercluster_markov': 'PC_07NIQK'},
 301: {'node_id': 'PC_000A8B',
  'node_int': 301,
  'nedges': 50,
  'supercluster_markov': 'PC_001KCV'},
 302: {'node_id': 'PC_000AA6',
  'node_int': 302,
  'nedges': 54,
  'supercluster_markov': 'PC_06SWHR'},
 303: {'node_id': 'PC_000AAN',
  'node_int': 303,
  'nedges': 55,
  'supercluster_markov': 'PC_06VD8W'},
 304: {'node_id': 'PC_000AAO',
  'node_int': 304,
  'nedges': 55,
  'supercluster_markov': 'PC_0285D9'},
 305: {'node_id': 'PC_000ACB',
  'node_int': 305,
  'nedges': 50,
  'supercluster_markov': 'PC_05U9ZT'},
 306: {'node_id': 'PC_000AE4',
  'node_int': 306,
  'nedges': 67,
  'supercluster_markov': 'PC_08GZ9T'},
 307: {'node_id': 'PC_000AEB',
  'node_int': 307,
  'nedges': 115,
  'supercluster_markov': 'PC_01B4MJ'},
 308: {'node_id': 'PC_000AET',
  'node_int': 308,
  'nedges': 51,
  'supercluster_markov': 'PC_0009RO'},
 309: {'node_id': 'PC_000AFH',
  'node_int': 309,
  'nedges': 66,
  'supercluster_markov': 'PC_02IVE7'},
 310: {'node_id': 'PC_000AFV',
  'node_int': 310,
  'nedges': 23,
  'supercluster_markov': 'PC_069Q0C'},
 311: {'node_id': 'PC_000AG2',
  'node_int': 311,
  'nedges': 18,
  'supercluster_markov': 'PC_02C8PH'},
 312: {'node_id': 'PC_000AHB',
  'node_int': 312,
  'nedges': 52,
  'supercluster_markov': 'PC_041LAD'},
 313: {'node_id': 'PC_000ALT',
  'node_int': 313,
  'nedges': 50,
  'supercluster_markov': 'PC_017I2K'},
 314: {'node_id': 'PC_000AP3',
  'node_int': 314,
  'nedges': 50,
  'supercluster_markov': 'PC_017I2K'},
 315: {'node_id': 'PC_000ASE',
  'node_int': 315,
  'nedges': 83,
  'supercluster_markov': 'PC_033V7U'},
 316: {'node_id': 'PC_000AV9',
  'node_int': 316,
  'nedges': 37,
  'supercluster_markov': 'PC_00LC00'},
 317: {'node_id': 'PC_000AVG',
  'node_int': 317,
  'nedges': 53,
  'supercluster_markov': 'PC_032Q1F'},
 318: {'node_id': 'PC_000AVV',
  'node_int': 318,
  'nedges': 51,
  'supercluster_markov': 'PC_04KZQH'},
 319: {'node_id': 'PC_000B13',
  'node_int': 319,
  'nedges': 72,
  'supercluster_markov': 'PC_01OCQ4'},
 320: {'node_id': 'PC_000B19',
  'node_int': 320,
  'nedges': 52,
  'supercluster_markov': 'PC_02XYVD'},
 321: {'node_id': 'PC_000B2V',
  'node_int': 321,
  'nedges': 54,
  'supercluster_markov': 'PC_00URTR'},
 322: {'node_id': 'PC_000B3W',
  'node_int': 322,
  'nedges': 54,
  'supercluster_markov': 'PC_068OU9'},
 323: {'node_id': 'PC_000B7D',
  'node_int': 323,
  'nedges': 52,
  'supercluster_markov': 'PC_05FEUB'},
 324: {'node_id': 'PC_000B85',
  'node_int': 324,
  'nedges': 53,
  'supercluster_markov': 'PC_01LOK6'},
 325: {'node_id': 'PC_000B8O',
  'node_int': 325,
  'nedges': 31,
  'supercluster_markov': 'PC_03VYM3'},
 326: {'node_id': 'PC_000BA2',
  'node_int': 326,
  'nedges': 60,
  'supercluster_markov': 'PC_03YP5C'},
 327: {'node_id': 'PC_000BB4',
  'node_int': 327,
  'nedges': 50,
  'supercluster_markov': 'PC_00G7DT'},
 328: {'node_id': 'PC_000BCY',
  'node_int': 328,
  'nedges': 8,
  'supercluster_markov': 'PC_0502JE'},
 329: {'node_id': 'PC_000BE0',
  'node_int': 329,
  'nedges': 50,
  'supercluster_markov': 'PC_00YLBJ'},
 330: {'node_id': 'PC_000BF9',
  'node_int': 330,
  'nedges': 50,
  'supercluster_markov': 'PC_052V2R'},
 331: {'node_id': 'PC_000BG2',
  'node_int': 331,
  'nedges': 50,
  'supercluster_markov': 'PC_04FX6B'},
 332: {'node_id': 'PC_000BL3',
  'node_int': 332,
  'nedges': 51,
  'supercluster_markov': 'PC_02AKK7'},
 333: {'node_id': 'PC_000BLW',
  'node_int': 333,
  'nedges': 50,
  'supercluster_markov': 'PC_019LQZ'},
 334: {'node_id': 'PC_000BN9',
  'node_int': 334,
  'nedges': 86,
  'supercluster_markov': 'PC_046DTC'},
 335: {'node_id': 'PC_000BNC',
  'node_int': 335,
  'nedges': 72,
  'supercluster_markov': 'PC_05UMJN'},
 336: {'node_id': 'PC_000BNR',
  'node_int': 336,
  'nedges': 50,
  'supercluster_markov': 'PC_04RUJN'},
 337: {'node_id': 'PC_000BPC',
  'node_int': 337,
  'nedges': 57,
  'supercluster_markov': 'PC_065ZKY'},
 338: {'node_id': 'PC_000BQW',
  'node_int': 338,
  'nedges': 78,
  'supercluster_markov': 'PC_05FHTA'},
 339: {'node_id': 'PC_000BSC',
  'node_int': 339,
  'nedges': 27,
  'supercluster_markov': 'PC_02Z69R'},
 340: {'node_id': 'PC_000BT4',
  'node_int': 340,
  'nedges': 64,
  'supercluster_markov': 'PC_0229E6'},
 341: {'node_id': 'PC_000BTS',
  'node_int': 341,
  'nedges': 66,
  'supercluster_markov': 'PC_04Z1TZ'},
 342: {'node_id': 'PC_000BZC',
  'node_int': 342,
  'nedges': 50,
  'supercluster_markov': 'PC_058Q5G'},
 343: {'node_id': 'PC_000C0U',
  'node_int': 343,
  'nedges': 50,
  'supercluster_markov': 'PC_02GINI'},
 344: {'node_id': 'PC_000C13',
  'node_int': 344,
  'nedges': 58,
  'supercluster_markov': 'PC_086AR6'},
 345: {'node_id': 'PC_000C4B',
  'node_int': 345,
  'nedges': 189,
  'supercluster_markov': 'PC_04CNN8'},
 346: {'node_id': 'PC_000C5K',
  'node_int': 346,
  'nedges': 50,
  'supercluster_markov': 'PC_02ETCM'},
 347: {'node_id': 'PC_000C5R',
  'node_int': 347,
  'nedges': 51,
  'supercluster_markov': 'PC_056GI0'},
 348: {'node_id': 'PC_000C6G',
  'node_int': 348,
  'nedges': 69,
  'supercluster_markov': 'PC_054BZB'},
 349: {'node_id': 'PC_000C8R',
  'node_int': 349,
  'nedges': 51,
  'supercluster_markov': 'PC_0229E6'},
 350: {'node_id': 'PC_000C98',
  'node_int': 350,
  'nedges': 50,
  'supercluster_markov': 'PC_003H85'},
 351: {'node_id': 'PC_000CC1',
  'node_int': 351,
  'nedges': 69,
  'supercluster_markov': 'PC_0029MW'},
 352: {'node_id': 'PC_000CCG',
  'node_int': 352,
  'nedges': 50,
  'supercluster_markov': 'PC_00DE50'},
 353: {'node_id': 'PC_000CDW',
  'node_int': 353,
  'nedges': 55,
  'supercluster_markov': 'PC_01CG4S'},
 354: {'node_id': 'PC_000CEN',
  'node_int': 354,
  'nedges': 63,
  'supercluster_markov': 'PC_06WPC5'},
 355: {'node_id': 'PC_000CGS',
  'node_int': 355,
  'nedges': 54,
  'supercluster_markov': 'PC_03D1TN'},
 356: {'node_id': 'PC_000CGU',
  'node_int': 356,
  'nedges': 115,
  'supercluster_markov': 'PC_07Y7YR'},
 357: {'node_id': 'PC_000CH1',
  'node_int': 357,
  'nedges': 140,
  'supercluster_markov': 'PC_02OSDN'},
 358: {'node_id': 'PC_000CLJ',
  'node_int': 358,
  'nedges': 55,
  'supercluster_markov': 'PC_04VPV5'},
 359: {'node_id': 'PC_000CLP',
  'node_int': 359,
  'nedges': 97,
  'supercluster_markov': 'PC_03YZJK'},
 360: {'node_id': 'PC_000CNI',
  'node_int': 360,
  'nedges': 166,
  'supercluster_markov': 'PC_043BXQ'},
 361: {'node_id': 'PC_000COP',
  'node_int': 361,
  'nedges': 57,
  'supercluster_markov': 'PC_02PZ3F'},
 362: {'node_id': 'PC_000CPH',
  'node_int': 362,
  'nedges': 67,
  'supercluster_markov': 'PC_00TAOF'},
 363: {'node_id': 'PC_000CQ3',
  'node_int': 363,
  'nedges': 68,
  'supercluster_markov': 'PC_04KZQH'},
 364: {'node_id': 'PC_000CQ5',
  'node_int': 364,
  'nedges': 51,
  'supercluster_markov': 'PC_059BWH'},
 365: {'node_id': 'PC_000CR4',
  'node_int': 365,
  'nedges': 51,
  'supercluster_markov': 'PC_019LQZ'},
 366: {'node_id': 'PC_000CYW',
  'node_int': 366,
  'nedges': 50,
  'supercluster_markov': 'PC_025D3C'},
 367: {'node_id': 'PC_000CYX',
  'node_int': 367,
  'nedges': 50,
  'supercluster_markov': 'PC_05GW50'},
 368: {'node_id': 'PC_000CZF',
  'node_int': 368,
  'nedges': 50,
  'supercluster_markov': 'PC_0813A5'},
 369: {'node_id': 'PC_000D2T',
  'node_int': 369,
  'nedges': 65,
  'supercluster_markov': 'PC_06S0FQ'},
 370: {'node_id': 'PC_000D43',
  'node_int': 370,
  'nedges': 51,
  'supercluster_markov': 'PC_018T19'},
 371: {'node_id': 'PC_000D9V',
  'node_int': 371,
  'nedges': 52,
  'supercluster_markov': 'PC_012DSI'},
 372: {'node_id': 'PC_000D9X',
  'node_int': 372,
  'nedges': 215,
  'supercluster_markov': 'PC_017I2K'},
 373: {'node_id': 'PC_000DAY',
  'node_int': 373,
  'nedges': 131,
  'supercluster_markov': 'PC_0544K4'},
 374: {'node_id': 'PC_000DBM',
  'node_int': 374,
  'nedges': 68,
  'supercluster_markov': 'PC_02NYGI'},
 375: {'node_id': 'PC_000DCV',
  'node_int': 375,
  'nedges': 53,
  'supercluster_markov': 'PC_07PF9W'},
 376: {'node_id': 'PC_000DD5',
  'node_int': 376,
  'nedges': 330,
  'supercluster_markov': 'PC_017I2K'},
 377: {'node_id': 'PC_000DE8',
  'node_int': 377,
  'nedges': 56,
  'supercluster_markov': 'PC_06I84L'},
 378: {'node_id': 'PC_000DEE',
  'node_int': 378,
  'nedges': 53,
  'supercluster_markov': 'PC_08IPTF'},
 379: {'node_id': 'PC_000DHG',
  'node_int': 379,
  'nedges': 51,
  'supercluster_markov': 'PC_02NPL8'},
 380: {'node_id': 'PC_000DIL',
  'node_int': 380,
  'nedges': 53,
  'supercluster_markov': 'PC_04IGFB'},
 381: {'node_id': 'PC_000DJ8',
  'node_int': 381,
  'nedges': 50,
  'supercluster_markov': 'PC_08GZF3'},
 382: {'node_id': 'PC_000DJX',
  'node_int': 382,
  'nedges': 50,
  'supercluster_markov': 'PC_07ZKK3'},
 383: {'node_id': 'PC_000DM3',
  'node_int': 383,
  'nedges': 113,
  'supercluster_markov': 'PC_008E3F'},
 384: {'node_id': 'PC_000DMF',
  'node_int': 384,
  'nedges': 110,
  'supercluster_markov': 'PC_0229E6'},
 385: {'node_id': 'PC_000DMJ',
  'node_int': 385,
  'nedges': 63,
  'supercluster_markov': 'PC_086N1M'},
 386: {'node_id': 'PC_000DND',
  'node_int': 386,
  'nedges': 52,
  'supercluster_markov': 'PC_01D6DG'},
 387: {'node_id': 'PC_000DQL',
  'node_int': 387,
  'nedges': 52,
  'supercluster_markov': 'PC_03IFH0'},
 388: {'node_id': 'PC_000DSN',
  'node_int': 388,
  'nedges': 51,
  'supercluster_markov': 'PC_05SY89'},
 389: {'node_id': 'PC_000DST',
  'node_int': 389,
  'nedges': 89,
  'supercluster_markov': 'PC_0210B7'},
 390: {'node_id': 'PC_000DT1',
  'node_int': 390,
  'nedges': 60,
  'supercluster_markov': 'PC_08KGXK'},
 391: {'node_id': 'PC_000DUN',
  'node_int': 391,
  'nedges': 50,
  'supercluster_markov': 'PC_001KCV'},
 392: {'node_id': 'PC_000DV1',
  'node_int': 392,
  'nedges': 58,
  'supercluster_markov': 'PC_06Q9T1'},
 393: {'node_id': 'PC_000E0A',
  'node_int': 393,
  'nedges': 55,
  'supercluster_markov': 'PC_06MCAB'},
 394: {'node_id': 'PC_000E1N',
  'node_int': 394,
  'nedges': 57,
  'supercluster_markov': 'PC_00YLBJ'},
 395: {'node_id': 'PC_000E3P',
  'node_int': 395,
  'nedges': 6,
  'supercluster_markov': 'PC_022Q3I'},
 396: {'node_id': 'PC_000E4K',
  'node_int': 396,
  'nedges': 79,
  'supercluster_markov': 'PC_03DZ2X'},
 397: {'node_id': 'PC_000E58',
  'node_int': 397,
  'nedges': 79,
  'supercluster_markov': 'PC_04ZPF1'},
 398: {'node_id': 'PC_000E6I',
  'node_int': 398,
  'nedges': 186,
  'supercluster_markov': 'PC_04ZPF1'},
 399: {'node_id': 'PC_000E6R',
  'node_int': 399,
  'nedges': 54,
  'supercluster_markov': 'PC_020SRS'},
 400: {'node_id': 'PC_000E76',
  'node_int': 400,
  'nedges': 111,
  'supercluster_markov': 'PC_05CIT8'},
 401: {'node_id': 'PC_000E7Q',
  'node_int': 401,
  'nedges': 50,
  'supercluster_markov': 'PC_00DE50'},
 402: {'node_id': 'PC_000E8B',
  'node_int': 402,
  'nedges': 51,
  'supercluster_markov': 'PC_017I2K'},
 403: {'node_id': 'PC_000E8T',
  'node_int': 403,
  'nedges': 184,
  'supercluster_markov': 'PC_00TVEO'},
 404: {'node_id': 'PC_000EAG',
  'node_int': 404,
  'nedges': 50,
  'supercluster_markov': 'PC_040773'},
 405: {'node_id': 'PC_000EAK',
  'node_int': 405,
  'nedges': 50,
  'supercluster_markov': 'PC_06OA4L'},
 406: {'node_id': 'PC_000EC2',
  'node_int': 406,
  'nedges': 51,
  'supercluster_markov': 'PC_03NMIF'},
 407: {'node_id': 'PC_000ECE',
  'node_int': 407,
  'nedges': 52,
  'supercluster_markov': 'PC_024Q2O'},
 408: {'node_id': 'PC_000EDN',
  'node_int': 408,
  'nedges': 52,
  'supercluster_markov': 'PC_056GI0'},
 409: {'node_id': 'PC_000EHC',
  'node_int': 409,
  'nedges': 43,
  'supercluster_markov': 'PC_069RPH'},
 410: {'node_id': 'PC_000EHL',
  'node_int': 410,
  'nedges': 67,
  'supercluster_markov': 'PC_0229E6'},
 411: {'node_id': 'PC_000EHZ',
  'node_int': 411,
  'nedges': 50,
  'supercluster_markov': 'PC_06GTNH'},
 412: {'node_id': 'PC_000EJ0',
  'node_int': 412,
  'nedges': 159,
  'supercluster_markov': 'PC_03L0DV'},
 413: {'node_id': 'PC_000ENH',
  'node_int': 413,
  'nedges': 50,
  'supercluster_markov': 'PC_04YDES'},
 414: {'node_id': 'PC_000ENL',
  'node_int': 414,
  'nedges': 51,
  'supercluster_markov': 'PC_05PAWE'},
 415: {'node_id': 'PC_000EOT',
  'node_int': 415,
  'nedges': 50,
  'supercluster_markov': 'PC_001KCV'},
 416: {'node_id': 'PC_000ERW',
  'node_int': 416,
  'nedges': 51,
  'supercluster_markov': 'PC_04YDES'},
 417: {'node_id': 'PC_000ES7',
  'node_int': 417,
  'nedges': 50,
  'supercluster_markov': 'PC_01EDDL'},
 418: {'node_id': 'PC_000ESX',
  'node_int': 418,
  'nedges': 76,
  'supercluster_markov': 'PC_0544K4'},
 419: {'node_id': 'PC_000EUY',
  'node_int': 419,
  'nedges': 45,
  'supercluster_markov': 'PC_04JJLC'},
 420: {'node_id': 'PC_000EW0',
  'node_int': 420,
  'nedges': 67,
  'supercluster_markov': 'PC_01V515'},
 421: {'node_id': 'PC_000EYI',
  'node_int': 421,
  'nedges': 77,
  'supercluster_markov': 'PC_00ZXSI'},
 422: {'node_id': 'PC_000EZ4',
  'node_int': 422,
  'nedges': 50,
  'supercluster_markov': 'PC_06II73'},
 423: {'node_id': 'PC_000EZA',
  'node_int': 423,
  'nedges': 67,
  'supercluster_markov': 'PC_04Z1TZ'},
 424: {'node_id': 'PC_000EZG',
  'node_int': 424,
  'nedges': 52,
  'supercluster_markov': 'PC_068NSV'},
 425: {'node_id': 'PC_000F10',
  'node_int': 425,
  'nedges': 58,
  'supercluster_markov': 'PC_012SAL'},
 426: {'node_id': 'PC_000F5I',
  'node_int': 426,
  'nedges': 86,
  'supercluster_markov': 'PC_03VGCN'},
 427: {'node_id': 'PC_000F5L',
  'node_int': 427,
  'nedges': 52,
  'supercluster_markov': 'PC_04DYYD'},
 428: {'node_id': 'PC_000F5M',
  'node_int': 428,
  'nedges': 67,
  'supercluster_markov': 'PC_01HMLV'},
 429: {'node_id': 'PC_000F6U',
  'node_int': 429,
  'nedges': 139,
  'supercluster_markov': 'PC_04FX6B'},
 430: {'node_id': 'PC_000F82',
  'node_int': 430,
  'nedges': 147,
  'supercluster_markov': 'PC_04DYYD'},
 431: {'node_id': 'PC_000FE3',
  'node_int': 431,
  'nedges': 76,
  'supercluster_markov': 'PC_05W84L'},
 432: {'node_id': 'PC_000FEZ',
  'node_int': 432,
  'nedges': 52,
  'supercluster_markov': 'PC_04Z1TZ'},
 433: {'node_id': 'PC_000FG1',
  'node_int': 433,
  'nedges': 56,
  'supercluster_markov': 'PC_00THHH'},
 434: {'node_id': 'PC_000FG6',
  'node_int': 434,
  'nedges': 53,
  'supercluster_markov': 'PC_01I7SG'},
 435: {'node_id': 'PC_000FI2',
  'node_int': 435,
  'nedges': 52,
  'supercluster_markov': 'PC_0222WR'},
 436: {'node_id': 'PC_000FIF',
  'node_int': 436,
  'nedges': 20,
  'supercluster_markov': 'PC_01QFAP'},
 437: {'node_id': 'PC_000FJJ',
  'node_int': 437,
  'nedges': 81,
  'supercluster_markov': 'PC_041LAD'},
 438: {'node_id': 'PC_000FLI',
  'node_int': 438,
  'nedges': 51,
  'supercluster_markov': 'PC_00Q1IH'},
 439: {'node_id': 'PC_000FO5',
  'node_int': 439,
  'nedges': 55,
  'supercluster_markov': 'PC_03NIRP'},
 440: {'node_id': 'PC_000FR0',
  'node_int': 440,
  'nedges': 12,
  'supercluster_markov': 'PC_04ENOZ'},
 441: {'node_id': 'PC_000FS3',
  'node_int': 441,
  'nedges': 31,
  'supercluster_markov': 'PC_01B4MJ'},
 442: {'node_id': 'PC_000FSM',
  'node_int': 442,
  'nedges': 51,
  'supercluster_markov': 'PC_017I2K'},
 443: {'node_id': 'PC_000FTQ',
  'node_int': 443,
  'nedges': 82,
  'supercluster_markov': 'PC_06OA4L'},
 444: {'node_id': 'PC_000FTV',
  'node_int': 444,
  'nedges': 64,
  'supercluster_markov': 'PC_03IXCU'},
 445: {'node_id': 'PC_000FTZ',
  'node_int': 445,
  'nedges': 50,
  'supercluster_markov': 'PC_017I2K'},
 446: {'node_id': 'PC_000FVJ',
  'node_int': 446,
  'nedges': 54,
  'supercluster_markov': 'PC_04BIJZ'},
 447: {'node_id': 'PC_000FVO',
  'node_int': 447,
  'nedges': 105,
  'supercluster_markov': 'PC_07GIR5'},
 448: {'node_id': 'PC_000FVY',
  'node_int': 448,
  'nedges': 54,
  'supercluster_markov': 'PC_040LIN'},
 449: {'node_id': 'PC_000G27',
  'node_int': 449,
  'nedges': 62,
  'supercluster_markov': 'PC_03FX59'},
 450: {'node_id': 'PC_000G5L',
  'node_int': 450,
  'nedges': 426,
  'supercluster_markov': 'PC_05T7HM'},
 451: {'node_id': 'PC_000G7U',
  'node_int': 451,
  'nedges': 55,
  'supercluster_markov': 'PC_002R3M'},
 452: {'node_id': 'PC_000GAR',
  'node_int': 452,
  'nedges': 25,
  'supercluster_markov': 'PC_03AOMU'},
 453: {'node_id': 'PC_000GBY',
  'node_int': 453,
  'nedges': 76,
  'supercluster_markov': 'PC_07NDZC'},
 454: {'node_id': 'PC_000GD9',
  'node_int': 454,
  'nedges': 71,
  'supercluster_markov': 'PC_032Q1C'},
 455: {'node_id': 'PC_000GDB',
  'node_int': 455,
  'nedges': 50,
  'supercluster_markov': 'PC_03BU82'},
 456: {'node_id': 'PC_000GDR',
  'node_int': 456,
  'nedges': 50,
  'supercluster_markov': 'PC_04FX6B'},
 457: {'node_id': 'PC_000GIH',
  'node_int': 457,
  'nedges': 62,
  'supercluster_markov': 'PC_05JLZ9'},
 458: {'node_id': 'PC_000GK4',
  'node_int': 458,
  'nedges': 81,
  'supercluster_markov': 'PC_05W84L'},
 459: {'node_id': 'PC_000GLL',
  'node_int': 459,
  'nedges': 93,
  'supercluster_markov': 'PC_06F9DN'},
 460: {'node_id': 'PC_000GM0',
  'node_int': 460,
  'nedges': 263,
  'supercluster_markov': 'PC_02NPL8'},
 461: {'node_id': 'PC_000GN1',
  'node_int': 461,
  'nedges': 84,
  'supercluster_markov': 'PC_03VJQG'},
 462: {'node_id': 'PC_000GN6',
  'node_int': 462,
  'nedges': 75,
  'supercluster_markov': 'PC_043431'},
 463: {'node_id': 'PC_000GQ6',
  'node_int': 463,
  'nedges': 61,
  'supercluster_markov': 'PC_05GW50'},
 464: {'node_id': 'PC_000GUM',
  'node_int': 464,
  'nedges': 52,
  'supercluster_markov': 'PC_026DGP'},
 465: {'node_id': 'PC_000GWH',
  'node_int': 465,
  'nedges': 97,
  'supercluster_markov': 'PC_02H654'},
 466: {'node_id': 'PC_000GZ2',
  'node_int': 466,
  'nedges': 79,
  'supercluster_markov': 'PC_04CAM3'},
 467: {'node_id': 'PC_000GZZ',
  'node_int': 467,
  'nedges': 95,
  'supercluster_markov': 'PC_01AMXQ'},
 468: {'node_id': 'PC_000H0F',
  'node_int': 468,
  'nedges': 25,
  'supercluster_markov': 'PC_06UH99'},
 469: {'node_id': 'PC_000H1O',
  'node_int': 469,
  'nedges': 58,
  'supercluster_markov': 'PC_083S7Q'},
 470: {'node_id': 'PC_000H4E',
  'node_int': 470,
  'nedges': 35,
  'supercluster_markov': 'PC_01GP9M'},
 471: {'node_id': 'PC_000H4S',
  'node_int': 471,
  'nedges': 73,
  'supercluster_markov': 'PC_05JXJP'},
 472: {'node_id': 'PC_000H5O',
  'node_int': 472,
  'nedges': 50,
  'supercluster_markov': 'PC_070747'},
 473: {'node_id': 'PC_000H5V',
  'node_int': 473,
  'nedges': 102,
  'supercluster_markov': 'PC_00G7DT'},
 474: {'node_id': 'PC_000H66',
  'node_int': 474,
  'nedges': 50,
  'supercluster_markov': 'PC_045LJ7'},
 475: {'node_id': 'PC_000H6J',
  'node_int': 475,
  'nedges': 111,
  'supercluster_markov': 'PC_06SWHR'},
 476: {'node_id': 'PC_000H87',
  'node_int': 476,
  'nedges': 62,
  'supercluster_markov': 'PC_02T7WU'},
 477: {'node_id': 'PC_000H9X',
  'node_int': 477,
  'nedges': 61,
  'supercluster_markov': 'PC_04Z1TZ'},
 478: {'node_id': 'PC_000H9Z',
  'node_int': 478,
  'nedges': 52,
  'supercluster_markov': 'PC_04Z1TZ'},
 479: {'node_id': 'PC_000HAV',
  'node_int': 479,
  'nedges': 136,
  'supercluster_markov': 'PC_020SRS'},
 480: {'node_id': 'PC_000HB4',
  'node_int': 480,
  'nedges': 61,
  'supercluster_markov': 'PC_068OU9'},
 481: {'node_id': 'PC_000HDL',
  'node_int': 481,
  'nedges': 34,
  'supercluster_markov': 'PC_000HDL'},
 482: {'node_id': 'PC_000HFT',
  'node_int': 482,
  'nedges': 59,
  'supercluster_markov': 'PC_06MYJ6'},
 483: {'node_id': 'PC_000HG7',
  'node_int': 483,
  'nedges': 141,
  'supercluster_markov': 'PC_030X3X'},
 484: {'node_id': 'PC_000HH3',
  'node_int': 484,
  'nedges': 145,
  'supercluster_markov': 'PC_01MQNN'},
 485: {'node_id': 'PC_000HOJ',
  'node_int': 485,
  'nedges': 56,
  'supercluster_markov': 'PC_03DSG4'},
 486: {'node_id': 'PC_000HOQ',
  'node_int': 486,
  'nedges': 59,
  'supercluster_markov': 'PC_01JMIR'},
 487: {'node_id': 'PC_000HVK',
  'node_int': 487,
  'nedges': 101,
  'supercluster_markov': 'PC_02CE2K'},
 488: {'node_id': 'PC_000HWY',
  'node_int': 488,
  'nedges': 81,
  'supercluster_markov': 'PC_009UTY'},
 489: {'node_id': 'PC_000HY4',
  'node_int': 489,
  'nedges': 51,
  'supercluster_markov': 'PC_049GIO'},
 490: {'node_id': 'PC_000HZO',
  'node_int': 490,
  'nedges': 51,
  'supercluster_markov': 'PC_02HK5E'},
 491: {'node_id': 'PC_000I01',
  'node_int': 491,
  'nedges': 50,
  'supercluster_markov': 'PC_05QCZN'},
 492: {'node_id': 'PC_000I1E',
  'node_int': 492,
  'nedges': 50,
  'supercluster_markov': 'PC_04RUJN'},
 493: {'node_id': 'PC_000I1G',
  'node_int': 493,
  'nedges': 10,
  'supercluster_markov': 'PC_01O94B'},
 494: {'node_id': 'PC_000I3G',
  'node_int': 494,
  'nedges': 53,
  'supercluster_markov': 'PC_04JRL2'},
 495: {'node_id': 'PC_000I49',
  'node_int': 495,
  'nedges': 105,
  'supercluster_markov': 'PC_01O6FX'},
 496: {'node_id': 'PC_000I4D',
  'node_int': 496,
  'nedges': 347,
  'supercluster_markov': 'PC_018SM6'},
 497: {'node_id': 'PC_000I8G',
  'node_int': 497,
  'nedges': 34,
  'supercluster_markov': 'PC_05OH0N'},
 498: {'node_id': 'PC_000I9Z',
  'node_int': 498,
  'nedges': 91,
  'supercluster_markov': 'PC_05C1EU'},
 499: {'node_id': 'PC_000IA8',
  'node_int': 499,
  'nedges': 51,
  'supercluster_markov': 'PC_010T6X'},
 500: {'node_id': 'PC_000ID1',
  'node_int': 500,
  'nedges': 29,
  'supercluster_markov': 'PC_07XRZ6'},
 501: {'node_id': 'PC_000IGB',
  'node_int': 501,
  'nedges': 77,
  'supercluster_markov': 'PC_048JCR'},
 502: {'node_id': 'PC_000IGF',
  'node_int': 502,
  'nedges': 50,
  'supercluster_markov': 'PC_02CE2K'},
 503: {'node_id': 'PC_000IGX',
  'node_int': 503,
  'nedges': 27,
  'supercluster_markov': 'PC_06OA4L'},
 504: {'node_id': 'PC_000IIE',
  'node_int': 504,
  'nedges': 50,
  'supercluster_markov': 'PC_0327T9'},
 505: {'node_id': 'PC_000IKX',
  'node_int': 505,
  'nedges': 50,
  'supercluster_markov': 'PC_05MOBP'},
 506: {'node_id': 'PC_000INR',
  'node_int': 506,
  'nedges': 22,
  'supercluster_markov': 'PC_067B1D'},
 507: {'node_id': 'PC_000IOW',
  'node_int': 507,
  'nedges': 72,
  'supercluster_markov': 'PC_05MVTJ'},
 508: {'node_id': 'PC_000IQH',
  'node_int': 508,
  'nedges': 53,
  'supercluster_markov': 'PC_04Z1TZ'},
 509: {'node_id': 'PC_000IR5',
  'node_int': 509,
  'nedges': 62,
  'supercluster_markov': 'PC_055Y72'},
 510: {'node_id': 'PC_000IRR',
  'node_int': 510,
  'nedges': 114,
  'supercluster_markov': 'PC_0285D9'},
 511: {'node_id': 'PC_000ITC',
  'node_int': 511,
  'nedges': 55,
  'supercluster_markov': 'PC_04DO9I'},
 512: {'node_id': 'PC_000ITH',
  'node_int': 512,
  'nedges': 55,
  'supercluster_markov': 'PC_04FL8M'},
 513: {'node_id': 'PC_000ITL',
  'node_int': 513,
  'nedges': 50,
  'supercluster_markov': 'PC_001KCV'},
 514: {'node_id': 'PC_000ITQ',
  'node_int': 514,
  'nedges': 132,
  'supercluster_markov': 'PC_04G1T3'},
 515: {'node_id': 'PC_000IW3',
  'node_int': 515,
  'nedges': 67,
  'supercluster_markov': 'PC_06DIZZ'},
 516: {'node_id': 'PC_000IWV',
  'node_int': 516,
  'nedges': 70,
  'supercluster_markov': 'PC_06OA4L'},
 517: {'node_id': 'PC_000IXY',
  'node_int': 517,
  'nedges': 50,
  'supercluster_markov': 'PC_05JLZ9'},
 518: {'node_id': 'PC_000J0J',
  'node_int': 518,
  'nedges': 62,
  'supercluster_markov': 'PC_0229E6'},
 519: {'node_id': 'PC_000J2X',
  'node_int': 519,
  'nedges': 50,
  'supercluster_markov': 'PC_04Z1TZ'},
 520: {'node_id': 'PC_000J37',
  'node_int': 520,
  'nedges': 60,
  'supercluster_markov': 'PC_001ISM'},
 521: {'node_id': 'PC_000J4Y',
  'node_int': 521,
  'nedges': 50,
  'supercluster_markov': 'PC_04IGFB'},
 522: {'node_id': 'PC_000J71',
  'node_int': 522,
  'nedges': 106,
  'supercluster_markov': 'PC_01ZMFZ'},
 523: {'node_id': 'PC_000J74',
  'node_int': 523,
  'nedges': 15,
  'supercluster_markov': 'PC_02UUUD'},
 524: {'node_id': 'PC_000J9K',
  'node_int': 524,
  'nedges': 55,
  'supercluster_markov': 'PC_05U9ZT'},
 525: {'node_id': 'PC_000JB2',
  'node_int': 525,
  'nedges': 62,
  'supercluster_markov': 'PC_065PL9'},
 526: {'node_id': 'PC_000JC0',
  'node_int': 526,
  'nedges': 106,
  'supercluster_markov': 'PC_02O0RA'},
 527: {'node_id': 'PC_000JD6',
  'node_int': 527,
  'nedges': 50,
  'supercluster_markov': 'PC_003VWA'},
 528: {'node_id': 'PC_000JD9',
  'node_int': 528,
  'nedges': 64,
  'supercluster_markov': 'PC_006K1D'},
 529: {'node_id': 'PC_000JDQ',
  'node_int': 529,
  'nedges': 62,
  'supercluster_markov': 'PC_02ETCM'},
 530: {'node_id': 'PC_000JJL',
  'node_int': 530,
  'nedges': 50,
  'supercluster_markov': 'PC_075U70'},
 531: {'node_id': 'PC_000JJZ',
  'node_int': 531,
  'nedges': 107,
  'supercluster_markov': 'PC_08GZ9T'},
 532: {'node_id': 'PC_000JK1',
  'node_int': 532,
  'nedges': 1144,
  'supercluster_markov': 'PC_001KCV'},
 533: {'node_id': 'PC_000JKG',
  'node_int': 533,
  'nedges': 52,
  'supercluster_markov': 'PC_04Z1TZ'},
 534: {'node_id': 'PC_000JN7',
  'node_int': 534,
  'nedges': 50,
  'supercluster_markov': 'PC_07NZVJ'},
 535: {'node_id': 'PC_000JOO',
  'node_int': 535,
  'nedges': 55,
  'supercluster_markov': 'PC_00RQHP'},
 536: {'node_id': 'PC_000JP4',
  'node_int': 536,
  'nedges': 141,
  'supercluster_markov': 'PC_03SUJL'},
 537: {'node_id': 'PC_000JPJ',
  'node_int': 537,
  'nedges': 56,
  'supercluster_markov': 'PC_01GVBK'},
 538: {'node_id': 'PC_000JQQ',
  'node_int': 538,
  'nedges': 87,
  'supercluster_markov': 'PC_06P41T'},
 539: {'node_id': 'PC_000JRF',
  'node_int': 539,
  'nedges': 142,
  'supercluster_markov': 'PC_000JRF'},
 540: {'node_id': 'PC_000JTS',
  'node_int': 540,
  'nedges': 52,
  'supercluster_markov': 'PC_03TRQM'},
 541: {'node_id': 'PC_000JUN',
  'node_int': 541,
  'nedges': 50,
  'supercluster_markov': 'PC_04CNN8'},
 542: {'node_id': 'PC_000JVH',
  'node_int': 542,
  'nedges': 70,
  'supercluster_markov': 'PC_02XEG8'},
 543: {'node_id': 'PC_000K18',
  'node_int': 543,
  'nedges': 121,
  'supercluster_markov': 'PC_06NSK3'},
 544: {'node_id': 'PC_000K1G',
  'node_int': 544,
  'nedges': 64,
  'supercluster_markov': 'PC_03TSDQ'},
 545: {'node_id': 'PC_000K2T',
  'node_int': 545,
  'nedges': 50,
  'supercluster_markov': 'PC_05E542'},
 546: {'node_id': 'PC_000K9S',
  'node_int': 546,
  'nedges': 54,
  'supercluster_markov': 'PC_04Z1TZ'},
 547: {'node_id': 'PC_000KA4',
  'node_int': 547,
  'nedges': 536,
  'supercluster_markov': 'PC_03WKSI'},
 548: {'node_id': 'PC_000KD3',
  'node_int': 548,
  'nedges': 90,
  'supercluster_markov': 'PC_01SJJ6'},
 549: {'node_id': 'PC_000KDY',
  'node_int': 549,
  'nedges': 50,
  'supercluster_markov': 'PC_00SM5I'},
 550: {'node_id': 'PC_000KEX',
  'node_int': 550,
  'nedges': 51,
  'supercluster_markov': 'PC_048JCR'},
 551: {'node_id': 'PC_000KF6',
  'node_int': 551,
  'nedges': 128,
  'supercluster_markov': 'PC_04BE6S'},
 552: {'node_id': 'PC_000KFF',
  'node_int': 552,
  'nedges': 40,
  'supercluster_markov': 'PC_04ENOZ'},
 553: {'node_id': 'PC_000KFH',
  'node_int': 553,
  'nedges': 123,
  'supercluster_markov': 'PC_020SRS'},
 554: {'node_id': 'PC_000KH7',
  'node_int': 554,
  'nedges': 50,
  'supercluster_markov': 'PC_05Y6BT'},
 555: {'node_id': 'PC_000KKL',
  'node_int': 555,
  'nedges': 152,
  'supercluster_markov': 'PC_061MZC'},
 556: {'node_id': 'PC_000KNC',
  'node_int': 556,
  'nedges': 9,
  'supercluster_markov': 'PC_027UX3'},
 557: {'node_id': 'PC_000KPL',
  'node_int': 557,
  'nedges': 50,
  'supercluster_markov': 'PC_00YLBJ'},
 558: {'node_id': 'PC_000KSC',
  'node_int': 558,
  'nedges': 66,
  'supercluster_markov': 'PC_05FHTA'},
 559: {'node_id': 'PC_000KTS',
  'node_int': 559,
  'nedges': 56,
  'supercluster_markov': 'PC_00AAGL'},
 560: {'node_id': 'PC_000KU0',
  'node_int': 560,
  'nedges': 54,
  'supercluster_markov': 'PC_07NZVJ'},
 561: {'node_id': 'PC_000KX5',
  'node_int': 561,
  'nedges': 56,
  'supercluster_markov': 'PC_01CG4S'},
 562: {'node_id': 'PC_000KYR',
  'node_int': 562,
  'nedges': 48,
  'supercluster_markov': 'PC_06R811'},
 563: {'node_id': 'PC_000L4A',
  'node_int': 563,
  'nedges': 125,
  'supercluster_markov': 'PC_04CY8Y'},
 564: {'node_id': 'PC_000L4K',
  'node_int': 564,
  'nedges': 23,
  'supercluster_markov': 'PC_032Q1C'},
 565: {'node_id': 'PC_000L4T',
  'node_int': 565,
  'nedges': 97,
  'supercluster_markov': 'PC_0861TC'},
 566: {'node_id': 'PC_000L5T',
  'node_int': 566,
  'nedges': 57,
  'supercluster_markov': 'PC_01STXZ'},
 567: {'node_id': 'PC_000L6L',
  'node_int': 567,
  'nedges': 84,
  'supercluster_markov': 'PC_02OA60'},
 568: {'node_id': 'PC_000L7I',
  'node_int': 568,
  'nedges': 51,
  'supercluster_markov': 'PC_00H7PF'},
 569: {'node_id': 'PC_000L8O',
  'node_int': 569,
  'nedges': 287,
  'supercluster_markov': 'PC_000L8O'},
 570: {'node_id': 'PC_000L9L',
  'node_int': 570,
  'nedges': 69,
  'supercluster_markov': 'PC_03S7G4'},
 571: {'node_id': 'PC_000LAO',
  'node_int': 571,
  'nedges': 12,
  'supercluster_markov': 'PC_00S70S'},
 572: {'node_id': 'PC_000LCA',
  'node_int': 572,
  'nedges': 141,
  'supercluster_markov': 'PC_05GW50'},
 573: {'node_id': 'PC_000LE5',
  'node_int': 573,
  'nedges': 57,
  'supercluster_markov': 'PC_06SWHR'},
 574: {'node_id': 'PC_000LGR',
  'node_int': 574,
  'nedges': 51,
  'supercluster_markov': 'PC_00H391'},
 575: {'node_id': 'PC_000LKY',
  'node_int': 575,
  'nedges': 34,
  'supercluster_markov': 'PC_024HG3'},
 576: {'node_id': 'PC_000LL9',
  'node_int': 576,
  'nedges': 57,
  'supercluster_markov': 'PC_06QKPS'},
 577: {'node_id': 'PC_000LRJ',
  'node_int': 577,
  'nedges': 460,
  'supercluster_markov': 'PC_04ZPF1'},
 578: {'node_id': 'PC_000LSW',
  'node_int': 578,
  'nedges': 50,
  'supercluster_markov': 'PC_065PL9'},
 579: {'node_id': 'PC_000LVE',
  'node_int': 579,
  'nedges': 61,
  'supercluster_markov': 'PC_038JWP'},
 580: {'node_id': 'PC_000LVI',
  'node_int': 580,
  'nedges': 51,
  'supercluster_markov': 'PC_034NST'},
 581: {'node_id': 'PC_000LVW',
  'node_int': 581,
  'nedges': 58,
  'supercluster_markov': 'PC_049GIO'},
 582: {'node_id': 'PC_000LZ5',
  'node_int': 582,
  'nedges': 65,
  'supercluster_markov': 'PC_03BVUQ'},
 583: {'node_id': 'PC_000M03',
  'node_int': 583,
  'nedges': 52,
  'supercluster_markov': 'PC_016ML3'},
 584: {'node_id': 'PC_000M0H',
  'node_int': 584,
  'nedges': 55,
  'supercluster_markov': 'PC_04ZXML'},
 585: {'node_id': 'PC_000M1G',
  'node_int': 585,
  'nedges': 62,
  'supercluster_markov': 'PC_022MR1'},
 586: {'node_id': 'PC_000M28',
  'node_int': 586,
  'nedges': 65,
  'supercluster_markov': 'PC_03SBNI'},
 587: {'node_id': 'PC_000M3C',
  'node_int': 587,
  'nedges': 51,
  'supercluster_markov': 'PC_04FCEG'},
 588: {'node_id': 'PC_000M3Q',
  'node_int': 588,
  'nedges': 90,
  'supercluster_markov': 'PC_048JCR'},
 589: {'node_id': 'PC_000M46',
  'node_int': 589,
  'nedges': 83,
  'supercluster_markov': 'PC_04WHZJ'},
 590: {'node_id': 'PC_000M5P',
  'node_int': 590,
  'nedges': 140,
  'supercluster_markov': 'PC_034PGU'},
 591: {'node_id': 'PC_000M6M',
  'node_int': 591,
  'nedges': 52,
  'supercluster_markov': 'PC_058MJ7'},
 592: {'node_id': 'PC_000M6O',
  'node_int': 592,
  'nedges': 50,
  'supercluster_markov': 'PC_04Z1TZ'},
 593: {'node_id': 'PC_000MBB',
  'node_int': 593,
  'nedges': 65,
  'supercluster_markov': 'PC_02R861'},
 594: {'node_id': 'PC_000MC5',
  'node_int': 594,
  'nedges': 175,
  'supercluster_markov': 'PC_07WC9J'},
 595: {'node_id': 'PC_000MCG',
  'node_int': 595,
  'nedges': 50,
  'supercluster_markov': 'PC_02F4JY'},
 596: {'node_id': 'PC_000MDT',
  'node_int': 596,
  'nedges': 90,
  'supercluster_markov': 'PC_03NN63'},
 597: {'node_id': 'PC_000ML3',
  'node_int': 597,
  'nedges': 50,
  'supercluster_markov': 'PC_06WPC5'},
 598: {'node_id': 'PC_000MM2',
  'node_int': 598,
  'nedges': 54,
  'supercluster_markov': 'PC_04GOD5'},
 599: {'node_id': 'PC_000MMQ',
  'node_int': 599,
  'nedges': 87,
  'supercluster_markov': 'PC_0071QL'},
 600: {'node_id': 'PC_000MSC',
  'node_int': 600,
  'nedges': 57,
  'supercluster_markov': 'PC_0210B7'},
 601: {'node_id': 'PC_000MSY',
  'node_int': 601,
  'nedges': 56,
  'supercluster_markov': 'PC_03SBOF'},
 602: {'node_id': 'PC_000MWQ',
  'node_int': 602,
  'nedges': 71,
  'supercluster_markov': 'PC_04ZPF1'},
 603: {'node_id': 'PC_000MZN',
  'node_int': 603,
  'nedges': 75,
  'supercluster_markov': 'PC_07TD20'},
 604: {'node_id': 'PC_000N0Q',
  'node_int': 604,
  'nedges': 50,
  'supercluster_markov': 'PC_06IID3'},
 605: {'node_id': 'PC_000N0V',
  'node_int': 605,
  'nedges': 50,
  'supercluster_markov': 'PC_04Z1TZ'},
 606: {'node_id': 'PC_000N1R',
  'node_int': 606,
  'nedges': 18,
  'supercluster_markov': 'PC_04UD62'},
 607: {'node_id': 'PC_000N1X',
  'node_int': 607,
  'nedges': 360,
  'supercluster_markov': 'PC_05JLZ9'},
 608: {'node_id': 'PC_000N45',
  'node_int': 608,
  'nedges': 61,
  'supercluster_markov': 'PC_01LNCE'},
 609: {'node_id': 'PC_000N8Z',
  'node_int': 609,
  'nedges': 39,
  'supercluster_markov': 'PC_05RBTN'},
 610: {'node_id': 'PC_000N92',
  'node_int': 610,
  'nedges': 18,
  'supercluster_markov': 'PC_000N92'},
 611: {'node_id': 'PC_000N9H',
  'node_int': 611,
  'nedges': 62,
  'supercluster_markov': 'PC_01BD6G'},
 612: {'node_id': 'PC_000NB5',
  'node_int': 612,
  'nedges': 51,
  'supercluster_markov': 'PC_06F0BO'},
 613: {'node_id': 'PC_000ND2',
  'node_int': 613,
  'nedges': 72,
  'supercluster_markov': 'PC_07Y7YR'},
 614: {'node_id': 'PC_000NE6',
  'node_int': 614,
  'nedges': 50,
  'supercluster_markov': 'PC_04Z1TZ'},
 615: {'node_id': 'PC_000NH1',
  'node_int': 615,
  'nedges': 169,
  'supercluster_markov': 'PC_025D3C'},
 616: {'node_id': 'PC_000NHD',
  'node_int': 616,
  'nedges': 51,
  'supercluster_markov': 'PC_011WBU'},
 617: {'node_id': 'PC_000NHU',
  'node_int': 617,
  'nedges': 188,
  'supercluster_markov': 'PC_02GINI'},
 618: {'node_id': 'PC_000NIM',
  'node_int': 618,
  'nedges': 75,
  'supercluster_markov': 'PC_04DYYD'},
 619: {'node_id': 'PC_000NJ4',
  'node_int': 619,
  'nedges': 108,
  'supercluster_markov': 'PC_06MYJ6'},
 620: {'node_id': 'PC_000NJH',
  'node_int': 620,
  'nedges': 101,
  'supercluster_markov': 'PC_01KO6L'},
 621: {'node_id': 'PC_000NKL',
  'node_int': 621,
  'nedges': 63,
  'supercluster_markov': 'PC_06UH99'},
 622: {'node_id': 'PC_000NMF',
  'node_int': 622,
  'nedges': 65,
  'supercluster_markov': 'PC_06N7LU'},
 623: {'node_id': 'PC_000NML',
  'node_int': 623,
  'nedges': 50,
  'supercluster_markov': 'PC_0150TZ'},
 624: {'node_id': 'PC_000NOX',
  'node_int': 624,
  'nedges': 96,
  'supercluster_markov': 'PC_00ZXSI'},
 625: {'node_id': 'PC_000NQF',
  'node_int': 625,
  'nedges': 8,
  'supercluster_markov': 'PC_06C1DD'},
 626: {'node_id': 'PC_000NRS',
  'node_int': 626,
  'nedges': 206,
  'supercluster_markov': 'PC_01CG4S'},
 627: {'node_id': 'PC_000NTQ',
  'node_int': 627,
  'nedges': 54,
  'supercluster_markov': 'PC_04ZPF1'},
 628: {'node_id': 'PC_000NVJ',
  'node_int': 628,
  'nedges': 79,
  'supercluster_markov': 'PC_07NZVJ'},
 629: {'node_id': 'PC_000NYQ',
  'node_int': 629,
  'nedges': 53,
  'supercluster_markov': 'PC_01O6FX'},
 630: {'node_id': 'PC_000NZ5',
  'node_int': 630,
  'nedges': 62,
  'supercluster_markov': 'PC_01SJJ6'},
 631: {'node_id': 'PC_000O2V',
  'node_int': 631,
  'nedges': 51,
  'supercluster_markov': 'PC_0738R9'},
 632: {'node_id': 'PC_000O2Y',
  'node_int': 632,
  'nedges': 50,
  'supercluster_markov': 'PC_00T39A'},
 633: {'node_id': 'PC_000O4Z',
  'node_int': 633,
  'nedges': 158,
  'supercluster_markov': 'PC_055Y72'},
 634: {'node_id': 'PC_000O5G',
  'node_int': 634,
  'nedges': 10,
  'supercluster_markov': 'PC_07PNVL'},
 635: {'node_id': 'PC_000O6N',
  'node_int': 635,
  'nedges': 51,
  'supercluster_markov': 'PC_056GI0'},
 636: {'node_id': 'PC_000O7V',
  'node_int': 636,
  'nedges': 50,
  'supercluster_markov': 'PC_02NPL8'},
 637: {'node_id': 'PC_000OCX',
  'node_int': 637,
  'nedges': 51,
  'supercluster_markov': 'PC_07WC9J'},
 638: {'node_id': 'PC_000OD0',
  'node_int': 638,
  'nedges': 52,
  'supercluster_markov': 'PC_01B991'},
 639: {'node_id': 'PC_000OEF',
  'node_int': 639,
  'nedges': 84,
  'supercluster_markov': 'PC_04CAM3'},
 640: {'node_id': 'PC_000OFO',
  'node_int': 640,
  'nedges': 61,
  'supercluster_markov': 'PC_012DSI'},
 641: {'node_id': 'PC_000OGI',
  'node_int': 641,
  'nedges': 78,
  'supercluster_markov': 'PC_08CJSM'},
 642: {'node_id': 'PC_000OI3',
  'node_int': 642,
  'nedges': 51,
  'supercluster_markov': 'PC_02XYB2'},
 643: {'node_id': 'PC_000OJ8',
  'node_int': 643,
  'nedges': 42,
  'supercluster_markov': 'PC_06PT0B'},
 644: {'node_id': 'PC_000OLX',
  'node_int': 644,
  'nedges': 67,
  'supercluster_markov': 'PC_01GVBK'},
 645: {'node_id': 'PC_000ON0',
  'node_int': 645,
  'nedges': 57,
  'supercluster_markov': 'PC_04FX6B'},
 646: {'node_id': 'PC_000OQ2',
  'node_int': 646,
  'nedges': 59,
  'supercluster_markov': 'PC_06GTNH'},
 647: {'node_id': 'PC_000OR9',
  'node_int': 647,
  'nedges': 50,
  'supercluster_markov': 'PC_04Z1TZ'},
 648: {'node_id': 'PC_000OSL',
  'node_int': 648,
  'nedges': 53,
  'supercluster_markov': 'PC_05FEUB'},
 649: {'node_id': 'PC_000OUL',
  'node_int': 649,
  'nedges': 52,
  'supercluster_markov': 'PC_0009RO'},
 650: {'node_id': 'PC_000P3P',
  'node_int': 650,
  'nedges': 50,
  'supercluster_markov': 'PC_048JCR'},
 651: {'node_id': 'PC_000P4M',
  'node_int': 651,
  'nedges': 54,
  'supercluster_markov': 'PC_04LRLB'},
 652: {'node_id': 'PC_000P4Y',
  'node_int': 652,
  'nedges': 93,
  'supercluster_markov': 'PC_04SOJA'},
 653: {'node_id': 'PC_000P59',
  'node_int': 653,
  'nedges': 81,
  'supercluster_markov': 'PC_03YZJK'},
 654: {'node_id': 'PC_000P5F',
  'node_int': 654,
  'nedges': 98,
  'supercluster_markov': 'PC_05JE7J'},
 655: {'node_id': 'PC_000P5N',
  'node_int': 655,
  'nedges': 83,
  'supercluster_markov': 'PC_07ZHMV'},
 656: {'node_id': 'PC_000P6R',
  'node_int': 656,
  'nedges': 88,
  'supercluster_markov': 'PC_07XRZ6'},
 657: {'node_id': 'PC_000P8V',
  'node_int': 657,
  'nedges': 50,
  'supercluster_markov': 'PC_06AMI3'},
 658: {'node_id': 'PC_000P9H',
  'node_int': 658,
  'nedges': 50,
  'supercluster_markov': 'PC_00XW9P'},
 659: {'node_id': 'PC_000PC9',
  'node_int': 659,
  'nedges': 91,
  'supercluster_markov': 'PC_04Z1TZ'},
 660: {'node_id': 'PC_000PCA',
  'node_int': 660,
  'nedges': 52,
  'supercluster_markov': 'PC_00SM5I'},
 661: {'node_id': 'PC_000PG4',
  'node_int': 661,
  'nedges': 115,
  'supercluster_markov': 'PC_06WQB1'},
 662: {'node_id': 'PC_000PGY',
  'node_int': 662,
  'nedges': 68,
  'supercluster_markov': 'PC_00074W'},
 663: {'node_id': 'PC_000PIM',
  'node_int': 663,
  'nedges': 32,
  'supercluster_markov': 'PC_06AMI3'},
 664: {'node_id': 'PC_000PKD',
  'node_int': 664,
  'nedges': 134,
  'supercluster_markov': 'PC_01SJJ6'},
 665: {'node_id': 'PC_000PKQ',
  'node_int': 665,
  'nedges': 50,
  'supercluster_markov': 'PC_06Z4LR'},
 666: {'node_id': 'PC_000PLQ',
  'node_int': 666,
  'nedges': 82,
  'supercluster_markov': 'PC_00DE50'},
 667: {'node_id': 'PC_000PMI',
  'node_int': 667,
  'nedges': 50,
  'supercluster_markov': 'PC_043ORU'},
 668: {'node_id': 'PC_000PMM',
  'node_int': 668,
  'nedges': 54,
  'supercluster_markov': 'PC_041B58'},
 669: {'node_id': 'PC_000PMZ',
  'node_int': 669,
  'nedges': 82,
  'supercluster_markov': 'PC_03DZ2X'},
 670: {'node_id': 'PC_000PN0',
  'node_int': 670,
  'nedges': 13,
  'supercluster_markov': 'PC_01CSA2'},
 671: {'node_id': 'PC_000PNN',
  'node_int': 671,
  'nedges': 55,
  'supercluster_markov': 'PC_04CNN8'},
 672: {'node_id': 'PC_000PUQ',
  'node_int': 672,
  'nedges': 60,
  'supercluster_markov': 'PC_0570D3'},
 673: {'node_id': 'PC_000PXE',
  'node_int': 673,
  'nedges': 53,
  'supercluster_markov': 'PC_019LQZ'},
 674: {'node_id': 'PC_000PYG',
  'node_int': 674,
  'nedges': 51,
  'supercluster_markov': 'PC_001ISM'},
 675: {'node_id': 'PC_000Q13',
  'node_int': 675,
  'nedges': 74,
  'supercluster_markov': 'PC_027UX3'},
 676: {'node_id': 'PC_000Q1M',
  'node_int': 676,
  'nedges': 12,
  'supercluster_markov': 'PC_00GB0F'},
 677: {'node_id': 'PC_000Q40',
  'node_int': 677,
  'nedges': 92,
  'supercluster_markov': 'PC_04JRL2'},
 678: {'node_id': 'PC_000Q5I',
  'node_int': 678,
  'nedges': 52,
  'supercluster_markov': 'PC_063GLA'},
 679: {'node_id': 'PC_000Q5J',
  'node_int': 679,
  'nedges': 50,
  'supercluster_markov': 'PC_00N5RS'},
 680: {'node_id': 'PC_000Q5R',
  'node_int': 680,
  'nedges': 47,
  'supercluster_markov': 'PC_040IOT'},
 681: {'node_id': 'PC_000Q7X',
  'node_int': 681,
  'nedges': 11,
  'supercluster_markov': 'PC_000Q7X'},
 682: {'node_id': 'PC_000Q8O',
  'node_int': 682,
  'nedges': 61,
  'supercluster_markov': 'PC_0150TZ'},
 683: {'node_id': 'PC_000Q90',
  'node_int': 683,
  'nedges': 47,
  'supercluster_markov': 'PC_04BE6S'},
 684: {'node_id': 'PC_000QCK',
  'node_int': 684,
  'nedges': 50,
  'supercluster_markov': 'PC_03BVUQ'},
 685: {'node_id': 'PC_000QCV',
  'node_int': 685,
  'nedges': 175,
  'supercluster_markov': 'PC_068NSV'},
 686: {'node_id': 'PC_000QFD',
  'node_int': 686,
  'nedges': 70,
  'supercluster_markov': 'PC_06HYJV'},
 687: {'node_id': 'PC_000QFO',
  'node_int': 687,
  'nedges': 51,
  'supercluster_markov': 'PC_058MJ7'},
 688: {'node_id': 'PC_000QFU',
  'node_int': 688,
  'nedges': 54,
  'supercluster_markov': 'PC_06F4U8'},
 689: {'node_id': 'PC_000QGU',
  'node_int': 689,
  'nedges': 51,
  'supercluster_markov': 'PC_022MR1'},
 690: {'node_id': 'PC_000QHH',
  'node_int': 690,
  'nedges': 50,
  'supercluster_markov': 'PC_001XWY'},
 691: {'node_id': 'PC_000QL4',
  'node_int': 691,
  'nedges': 26,
  'supercluster_markov': 'PC_034PGU'},
 692: {'node_id': 'PC_000QNS',
  'node_int': 692,
  'nedges': 366,
  'supercluster_markov': 'PC_02BX3J'},
 693: {'node_id': 'PC_000QSW',
  'node_int': 693,
  'nedges': 87,
  'supercluster_markov': 'PC_07ZHMV'},
 694: {'node_id': 'PC_000QTG',
  'node_int': 694,
  'nedges': 61,
  'supercluster_markov': 'PC_068NSV'},
 695: {'node_id': 'PC_000QVI',
  'node_int': 695,
  'nedges': 50,
  'supercluster_markov': 'PC_0229E6'},
 696: {'node_id': 'PC_000QWF',
  'node_int': 696,
  'nedges': 101,
  'supercluster_markov': 'PC_03A2BU'},
 697: {'node_id': 'PC_000QY0',
  'node_int': 697,
  'nedges': 74,
  'supercluster_markov': 'PC_03GXDV'},
 698: {'node_id': 'PC_000QYL',
  'node_int': 698,
  'nedges': 222,
  'supercluster_markov': 'PC_03MUEB'},
 699: {'node_id': 'PC_000QYW',
  'node_int': 699,
  'nedges': 65,
  'supercluster_markov': 'PC_069X1U'},
 700: {'node_id': 'PC_000QZD',
  'node_int': 700,
  'nedges': 82,
  'supercluster_markov': 'PC_01B991'},
 701: {'node_id': 'PC_000R03',
  'node_int': 701,
  'nedges': 59,
  'supercluster_markov': 'PC_0483K9'},
 702: {'node_id': 'PC_000R13',
  'node_int': 702,
  'nedges': 59,
  'supercluster_markov': 'PC_04Z1TZ'},
 703: {'node_id': 'PC_000R3L',
  'node_int': 703,
  'nedges': 91,
  'supercluster_markov': 'PC_00KTEK'},
 704: {'node_id': 'PC_000R3O',
  'node_int': 704,
  'nedges': 78,
  'supercluster_markov': 'PC_08D8IU'},
 705: {'node_id': 'PC_000R41',
  'node_int': 705,
  'nedges': 50,
  'supercluster_markov': 'PC_01B7AN'},
 706: {'node_id': 'PC_000R61',
  'node_int': 706,
  'nedges': 70,
  'supercluster_markov': 'PC_00N879'},
 707: {'node_id': 'PC_000R7X',
  'node_int': 707,
  'nedges': 321,
  'supercluster_markov': 'PC_048JCR'},
 708: {'node_id': 'PC_000R86',
  'node_int': 708,
  'nedges': 87,
  'supercluster_markov': 'PC_01T9YZ'},
 709: {'node_id': 'PC_000R9Y',
  'node_int': 709,
  'nedges': 51,
  'supercluster_markov': 'PC_03TOIJ'},
 710: {'node_id': 'PC_000RBH',
  'node_int': 710,
  'nedges': 52,
  'supercluster_markov': 'PC_03DSG4'},
 711: {'node_id': 'PC_000RBZ',
  'node_int': 711,
  'nedges': 126,
  'supercluster_markov': 'PC_0378ND'},
 712: {'node_id': 'PC_000RCU',
  'node_int': 712,
  'nedges': 50,
  'supercluster_markov': 'PC_04IBHT'},
 713: {'node_id': 'PC_000RGS',
  'node_int': 713,
  'nedges': 46,
  'supercluster_markov': 'PC_0259Z4'},
 714: {'node_id': 'PC_000RK6',
  'node_int': 714,
  'nedges': 174,
  'supercluster_markov': 'PC_04IGFB'},
 715: {'node_id': 'PC_000RKB',
  'node_int': 715,
  'nedges': 95,
  'supercluster_markov': 'PC_01I3EE'},
 716: {'node_id': 'PC_000RKC',
  'node_int': 716,
  'nedges': 54,
  'supercluster_markov': 'PC_06Z4LR'},
 717: {'node_id': 'PC_000RLC',
  'node_int': 717,
  'nedges': 51,
  'supercluster_markov': 'PC_05W84L'},
 718: {'node_id': 'PC_000RLU',
  'node_int': 718,
  'nedges': 50,
  'supercluster_markov': 'PC_04Z1TZ'},
 719: {'node_id': 'PC_000RN2',
  'node_int': 719,
  'nedges': 50,
  'supercluster_markov': 'PC_04Z1TZ'},
 720: {'node_id': 'PC_000RN3',
  'node_int': 720,
  'nedges': 17,
  'supercluster_markov': 'PC_000RN3'},
 721: {'node_id': 'PC_000RNA',
  'node_int': 721,
  'nedges': 52,
  'supercluster_markov': 'PC_02X0GZ'},
 722: {'node_id': 'PC_000RQF',
  'node_int': 722,
  'nedges': 502,
  'supercluster_markov': 'PC_04Z1TZ'},
 723: {'node_id': 'PC_000RS6',
  'node_int': 723,
  'nedges': 58,
  'supercluster_markov': 'PC_006ICR'},
 724: {'node_id': 'PC_000RVP',
  'node_int': 724,
  'nedges': 50,
  'supercluster_markov': 'PC_04GLN4'},
 725: {'node_id': 'PC_000RVY',
  'node_int': 725,
  'nedges': 13,
  'supercluster_markov': 'PC_01BG6A'},
 726: {'node_id': 'PC_000RZU',
  'node_int': 726,
  'nedges': 96,
  'supercluster_markov': 'PC_04TLUV'},
 727: {'node_id': 'PC_000S63',
  'node_int': 727,
  'nedges': 16,
  'supercluster_markov': 'PC_00GBAC'},
 728: {'node_id': 'PC_000S6Y',
  'node_int': 728,
  'nedges': 50,
  'supercluster_markov': 'PC_02VGIH'},
 729: {'node_id': 'PC_000S7U',
  'node_int': 729,
  'nedges': 394,
  'supercluster_markov': 'PC_02NPL8'},
 730: {'node_id': 'PC_000SA4',
  'node_int': 730,
  'nedges': 84,
  'supercluster_markov': 'PC_01KR2J'},
 731: {'node_id': 'PC_000SBO',
  'node_int': 731,
  'nedges': 50,
  'supercluster_markov': 'PC_06N7LU'},
 732: {'node_id': 'PC_000SE2',
  'node_int': 732,
  'nedges': 65,
  'supercluster_markov': 'PC_0222WR'},
 733: {'node_id': 'PC_000SH0',
  'node_int': 733,
  'nedges': 62,
  'supercluster_markov': 'PC_01EOWC'},
 734: {'node_id': 'PC_000SH2',
  'node_int': 734,
  'nedges': 18,
  'supercluster_markov': 'PC_00H391'},
 735: {'node_id': 'PC_000SHN',
  'node_int': 735,
  'nedges': 51,
  'supercluster_markov': 'PC_04Y53H'},
 736: {'node_id': 'PC_000SIT',
  'node_int': 736,
  'nedges': 52,
  'supercluster_markov': 'PC_034H78'},
 737: {'node_id': 'PC_000SLN',
  'node_int': 737,
  'nedges': 103,
  'supercluster_markov': 'PC_03NN63'},
 738: {'node_id': 'PC_000SMO',
  'node_int': 738,
  'nedges': 50,
  'supercluster_markov': 'PC_00YLBJ'},
 739: {'node_id': 'PC_000SN8',
  'node_int': 739,
  'nedges': 3,
  'supercluster_markov': 'PC_02MN9C'},
 740: {'node_id': 'PC_000SPD',
  'node_int': 740,
  'nedges': 65,
  'supercluster_markov': 'PC_048JCR'},
 741: {'node_id': 'PC_000SQB',
  'node_int': 741,
  'nedges': 50,
  'supercluster_markov': 'PC_00YLBJ'},
 742: {'node_id': 'PC_000SRI',
  'node_int': 742,
  'nedges': 51,
  'supercluster_markov': 'PC_07RDG0'},
 743: {'node_id': 'PC_000STO',
  'node_int': 743,
  'nedges': 51,
  'supercluster_markov': 'PC_017I2K'},
 744: {'node_id': 'PC_000STQ',
  'node_int': 744,
  'nedges': 108,
  'supercluster_markov': 'PC_01UCVE'},
 745: {'node_id': 'PC_000STV',
  'node_int': 745,
  'nedges': 92,
  'supercluster_markov': 'PC_082OPC'},
 746: {'node_id': 'PC_000SUD',
  'node_int': 746,
  'nedges': 61,
  'supercluster_markov': 'PC_02IVE7'},
 747: {'node_id': 'PC_000SUS',
  'node_int': 747,
  'nedges': 77,
  'supercluster_markov': 'PC_017I2K'},
 748: {'node_id': 'PC_000T1G',
  'node_int': 748,
  'nedges': 52,
  'supercluster_markov': 'PC_00DE50'},
 749: {'node_id': 'PC_000T3D',
  'node_int': 749,
  'nedges': 173,
  'supercluster_markov': 'PC_02CE2K'},
 750: {'node_id': 'PC_000T3R',
  'node_int': 750,
  'nedges': 65,
  'supercluster_markov': 'PC_01UCVE'},
 751: {'node_id': 'PC_000T85',
  'node_int': 751,
  'nedges': 63,
  'supercluster_markov': 'PC_03O094'},
 752: {'node_id': 'PC_000TBH',
  'node_int': 752,
  'nedges': 59,
  'supercluster_markov': 'PC_065PL9'},
 753: {'node_id': 'PC_000TBS',
  'node_int': 753,
  'nedges': 81,
  'supercluster_markov': 'PC_04FCEG'},
 754: {'node_id': 'PC_000TCZ',
  'node_int': 754,
  'nedges': 57,
  'supercluster_markov': 'PC_05VP7B'},
 755: {'node_id': 'PC_000TD5',
  'node_int': 755,
  'nedges': 51,
  'supercluster_markov': 'PC_017I2K'},
 756: {'node_id': 'PC_000TDB',
  'node_int': 756,
  'nedges': 50,
  'supercluster_markov': 'PC_002AG8'},
 757: {'node_id': 'PC_000TDQ',
  'node_int': 757,
  'nedges': 50,
  'supercluster_markov': 'PC_017I2K'},
 758: {'node_id': 'PC_000TDZ',
  'node_int': 758,
  'nedges': 1026,
  'supercluster_markov': 'PC_017I2K'},
 759: {'node_id': 'PC_000TEE',
  'node_int': 759,
  'nedges': 64,
  'supercluster_markov': 'PC_05GSLY'},
 760: {'node_id': 'PC_000THB',
  'node_int': 760,
  'nedges': 88,
  'supercluster_markov': 'PC_01SJJ6'},
 761: {'node_id': 'PC_000TKU',
  'node_int': 761,
  'nedges': 52,
  'supercluster_markov': 'PC_018Q71'},
 762: {'node_id': 'PC_000TL7',
  'node_int': 762,
  'nedges': 190,
  'supercluster_markov': 'PC_04KZQH'},
 763: {'node_id': 'PC_000TL9',
  'node_int': 763,
  'nedges': 50,
  'supercluster_markov': 'PC_00M20X'},
 764: {'node_id': 'PC_000TLF',
  'node_int': 764,
  'nedges': 59,
  'supercluster_markov': 'PC_02Y3X2'},
 765: {'node_id': 'PC_000TNY',
  'node_int': 765,
  'nedges': 52,
  'supercluster_markov': 'PC_063MEP'},
 766: {'node_id': 'PC_000TNZ',
  'node_int': 766,
  'nedges': 50,
  'supercluster_markov': 'PC_04ZPF1'},
 767: {'node_id': 'PC_000TOD',
  'node_int': 767,
  'nedges': 50,
  'supercluster_markov': 'PC_001KCV'},
 768: {'node_id': 'PC_000TSK',
  'node_int': 768,
  'nedges': 70,
  'supercluster_markov': 'PC_01B7AN'},
 769: {'node_id': 'PC_000TXL',
  'node_int': 769,
  'nedges': 61,
  'supercluster_markov': 'PC_07RVVX'},
 770: {'node_id': 'PC_000U1D',
  'node_int': 770,
  'nedges': 50,
  'supercluster_markov': 'PC_03NMIF'},
 771: {'node_id': 'PC_000U2P',
  'node_int': 771,
  'nedges': 81,
  'supercluster_markov': 'PC_05ZMNN'},
 772: {'node_id': 'PC_000U2Q',
  'node_int': 772,
  'nedges': 806,
  'supercluster_markov': 'PC_00YLBJ'},
 773: {'node_id': 'PC_000U37',
  'node_int': 773,
  'nedges': 50,
  'supercluster_markov': 'PC_08KP0V'},
 774: {'node_id': 'PC_000U4O',
  'node_int': 774,
  'nedges': 77,
  'supercluster_markov': 'PC_030X3X'},
 775: {'node_id': 'PC_000U5F',
  'node_int': 775,
  'nedges': 54,
  'supercluster_markov': 'PC_05MDOF'},
 776: {'node_id': 'PC_000U5N',
  'node_int': 776,
  'nedges': 51,
  'supercluster_markov': 'PC_02ZCN2'},
 777: {'node_id': 'PC_000U8J',
  'node_int': 777,
  'nedges': 28,
  'supercluster_markov': 'PC_01SSAS'},
 778: {'node_id': 'PC_000U9T',
  'node_int': 778,
  'nedges': 51,
  'supercluster_markov': 'PC_056GI0'},
 779: {'node_id': 'PC_000UBY',
  'node_int': 779,
  'nedges': 50,
  'supercluster_markov': 'PC_07ZHMV'},
 780: {'node_id': 'PC_000UCN',
  'node_int': 780,
  'nedges': 52,
  'supercluster_markov': 'PC_020SRS'},
 781: {'node_id': 'PC_000UCO',
  'node_int': 781,
  'nedges': 88,
  'supercluster_markov': 'PC_04RUJN'},
 782: {'node_id': 'PC_000UDN',
  'node_int': 782,
  'nedges': 87,
  'supercluster_markov': 'PC_0348W7'},
 783: {'node_id': 'PC_000UES',
  'node_int': 783,
  'nedges': 59,
  'supercluster_markov': 'PC_06TQEP'},
 784: {'node_id': 'PC_000UFO',
  'node_int': 784,
  'nedges': 76,
  'supercluster_markov': 'PC_07BJ3H'},
 785: {'node_id': 'PC_000UIU',
  'node_int': 785,
  'nedges': 6,
  'supercluster_markov': 'PC_05ZVDM'},
 786: {'node_id': 'PC_000UL9',
  'node_int': 786,
  'nedges': 433,
  'supercluster_markov': 'PC_04Z1TZ'},
 787: {'node_id': 'PC_000UO4',
  'node_int': 787,
  'nedges': 51,
  'supercluster_markov': 'PC_08GZ9T'},
 788: {'node_id': 'PC_000UP6',
  'node_int': 788,
  'nedges': 79,
  'supercluster_markov': 'PC_081XPX'},
 789: {'node_id': 'PC_000UPZ',
  'node_int': 789,
  'nedges': 50,
  'supercluster_markov': 'PC_04ZPF1'},
 790: {'node_id': 'PC_000UQK',
  'node_int': 790,
  'nedges': 59,
  'supercluster_markov': 'PC_012DSI'},
 791: {'node_id': 'PC_000UQU',
  'node_int': 791,
  'nedges': 144,
  'supercluster_markov': 'PC_03B9OL'},
 792: {'node_id': 'PC_000UTM',
  'node_int': 792,
  'nedges': 13,
  'supercluster_markov': 'PC_00CGI5'},
 793: {'node_id': 'PC_000UUA',
  'node_int': 793,
  'nedges': 177,
  'supercluster_markov': 'PC_04D8YQ'},
 794: {'node_id': 'PC_000UWZ',
  'node_int': 794,
  'nedges': 51,
  'supercluster_markov': 'PC_015SBK'},
 795: {'node_id': 'PC_000UXY',
  'node_int': 795,
  'nedges': 52,
  'supercluster_markov': 'PC_01X5Z2'},
 796: {'node_id': 'PC_000UY0',
  'node_int': 796,
  'nedges': 69,
  'supercluster_markov': 'PC_015SBK'},
 797: {'node_id': 'PC_000UZW',
  'node_int': 797,
  'nedges': 53,
  'supercluster_markov': 'PC_04Z1TZ'},
 798: {'node_id': 'PC_000V0G',
  'node_int': 798,
  'nedges': 54,
  'supercluster_markov': 'PC_044FPA'},
 799: {'node_id': 'PC_000V0O',
  'node_int': 799,
  'nedges': 102,
  'supercluster_markov': 'PC_048JCR'},
 800: {'node_id': 'PC_000V12',
  'node_int': 800,
  'nedges': 57,
  'supercluster_markov': 'PC_04KCDR'},
 801: {'node_id': 'PC_000V3V',
  'node_int': 801,
  'nedges': 74,
  'supercluster_markov': 'PC_02R861'},
 802: {'node_id': 'PC_000V4N',
  'node_int': 802,
  'nedges': 62,
  'supercluster_markov': 'PC_07F7XA'},
 803: {'node_id': 'PC_000V5G',
  'node_int': 803,
  'nedges': 50,
  'supercluster_markov': 'PC_08HMGX'},
 804: {'node_id': 'PC_000V75',
  'node_int': 804,
  'nedges': 50,
  'supercluster_markov': 'PC_04Z1TZ'},
 805: {'node_id': 'PC_000V7Z',
  'node_int': 805,
  'nedges': 150,
  'supercluster_markov': 'PC_01RCLA'},
 806: {'node_id': 'PC_000V8Z',
  'node_int': 806,
  'nedges': 50,
  'supercluster_markov': 'PC_04ZPF1'},
 807: {'node_id': 'PC_000VC2',
  'node_int': 807,
  'nedges': 50,
  'supercluster_markov': 'PC_00P9ED'},
 808: {'node_id': 'PC_000VD7',
  'node_int': 808,
  'nedges': 50,
  'supercluster_markov': 'PC_00YLBJ'},
 809: {'node_id': 'PC_000VE7',
  'node_int': 809,
  'nedges': 70,
  'supercluster_markov': 'PC_04GZQ2'},
 810: {'node_id': 'PC_000VJ5',
  'node_int': 810,
  'nedges': 21,
  'supercluster_markov': 'PC_04ZXML'},
 811: {'node_id': 'PC_000VM6',
  'node_int': 811,
  'nedges': 257,
  'supercluster_markov': 'PC_019LQZ'},
 812: {'node_id': 'PC_000VN3',
  'node_int': 812,
  'nedges': 50,
  'supercluster_markov': 'PC_01L8KT'},
 813: {'node_id': 'PC_000VO0',
  'node_int': 813,
  'nedges': 59,
  'supercluster_markov': 'PC_017KCR'},
 814: {'node_id': 'PC_000VOC',
  'node_int': 814,
  'nedges': 188,
  'supercluster_markov': 'PC_04GLN4'},
 815: {'node_id': 'PC_000VOY',
  'node_int': 815,
  'nedges': 13,
  'supercluster_markov': 'PC_08GYWS'},
 816: {'node_id': 'PC_000VP3',
  'node_int': 816,
  'nedges': 127,
  'supercluster_markov': 'PC_012C2G'},
 817: {'node_id': 'PC_000VQT',
  'node_int': 817,
  'nedges': 60,
  'supercluster_markov': 'PC_0172Q2'},
 818: {'node_id': 'PC_000VS6',
  'node_int': 818,
  'nedges': 164,
  'supercluster_markov': 'PC_01APJR'},
 819: {'node_id': 'PC_000VUH',
  'node_int': 819,
  'nedges': 10,
  'supercluster_markov': 'PC_07O53H'},
 820: {'node_id': 'PC_000W2H',
  'node_int': 820,
  'nedges': 15,
  'supercluster_markov': 'PC_03TPT3'},
 821: {'node_id': 'PC_000W3M',
  'node_int': 821,
  'nedges': 61,
  'supercluster_markov': 'PC_030X3X'},
 822: {'node_id': 'PC_000W7U',
  'node_int': 822,
  'nedges': 88,
  'supercluster_markov': 'PC_05CIT8'},
 823: {'node_id': 'PC_000W9S',
  'node_int': 823,
  'nedges': 68,
  'supercluster_markov': 'PC_02NYGI'},
 824: {'node_id': 'PC_000W9T',
  'node_int': 824,
  'nedges': 178,
  'supercluster_markov': 'PC_010T6X'},
 825: {'node_id': 'PC_000WAF',
  'node_int': 825,
  'nedges': 51,
  'supercluster_markov': 'PC_04PABL'},
 826: {'node_id': 'PC_000WAJ',
  'node_int': 826,
  'nedges': 84,
  'supercluster_markov': 'PC_05VP7B'},
 827: {'node_id': 'PC_000WCI',
  'node_int': 827,
  'nedges': 93,
  'supercluster_markov': 'PC_03GXDV'},
 828: {'node_id': 'PC_000WKK',
  'node_int': 828,
  'nedges': 51,
  'supercluster_markov': 'PC_017I2K'},
 829: {'node_id': 'PC_000WKN',
  'node_int': 829,
  'nedges': 50,
  'supercluster_markov': 'PC_02KM4V'},
 830: {'node_id': 'PC_000WL4',
  'node_int': 830,
  'nedges': 60,
  'supercluster_markov': 'PC_04GOD5'},
 831: {'node_id': 'PC_000WLX',
  'node_int': 831,
  'nedges': 50,
  'supercluster_markov': 'PC_017I2K'},
 832: {'node_id': 'PC_000WMA',
  'node_int': 832,
  'nedges': 428,
  'supercluster_markov': 'PC_04LRLB'},
 833: {'node_id': 'PC_000WRC',
  'node_int': 833,
  'nedges': 57,
  'supercluster_markov': 'PC_02QK72'},
 834: {'node_id': 'PC_000WRE',
  'node_int': 834,
  'nedges': 50,
  'supercluster_markov': 'PC_03BEHE'},
 835: {'node_id': 'PC_000WRG',
  'node_int': 835,
  'nedges': 50,
  'supercluster_markov': 'PC_001KCV'},
 836: {'node_id': 'PC_000WTD',
  'node_int': 836,
  'nedges': 51,
  'supercluster_markov': 'PC_001KCV'},
 837: {'node_id': 'PC_000WW5',
  'node_int': 837,
  'nedges': 98,
  'supercluster_markov': 'PC_02DN65'},
 838: {'node_id': 'PC_000X08',
  'node_int': 838,
  'nedges': 61,
  'supercluster_markov': 'PC_01V515'},
 839: {'node_id': 'PC_000X0H',
  'node_int': 839,
  'nedges': 85,
  'supercluster_markov': 'PC_01B7AN'},
 840: {'node_id': 'PC_000X4Q',
  'node_int': 840,
  'nedges': 52,
  'supercluster_markov': 'PC_01Y6QU'},
 841: {'node_id': 'PC_000X4Z',
  'node_int': 841,
  'nedges': 52,
  'supercluster_markov': 'PC_07POIQ'},
 842: {'node_id': 'PC_000X59',
  'node_int': 842,
  'nedges': 51,
  'supercluster_markov': 'PC_017I2K'},
 843: {'node_id': 'PC_000X6D',
  'node_int': 843,
  'nedges': 51,
  'supercluster_markov': 'PC_03UPZM'},
 844: {'node_id': 'PC_000X6M',
  'node_int': 844,
  'nedges': 108,
  'supercluster_markov': 'PC_06NSK3'},
 845: {'node_id': 'PC_000X72',
  'node_int': 845,
  'nedges': 57,
  'supercluster_markov': 'PC_02HOCX'},
 846: {'node_id': 'PC_000X75',
  'node_int': 846,
  'nedges': 50,
  'supercluster_markov': 'PC_02KM4V'},
 847: {'node_id': 'PC_000X7N',
  'node_int': 847,
  'nedges': 70,
  'supercluster_markov': 'PC_04Z1TZ'},
 848: {'node_id': 'PC_000X8Z',
  'node_int': 848,
  'nedges': 51,
  'supercluster_markov': 'PC_017I2K'},
 849: {'node_id': 'PC_000XFP',
  'node_int': 849,
  'nedges': 54,
  'supercluster_markov': 'PC_04Z1TZ'},
 850: {'node_id': 'PC_000XHH',
  'node_int': 850,
  'nedges': 57,
  'supercluster_markov': 'PC_055Y72'},
 851: {'node_id': 'PC_000XHN',
  'node_int': 851,
  'nedges': 379,
  'supercluster_markov': 'PC_02NPL8'},
 852: {'node_id': 'PC_000XID',
  'node_int': 852,
  'nedges': 139,
  'supercluster_markov': 'PC_003H85'},
 853: {'node_id': 'PC_000XKA',
  'node_int': 853,
  'nedges': 55,
  'supercluster_markov': 'PC_017I2K'},
 854: {'node_id': 'PC_000XKU',
  'node_int': 854,
  'nedges': 60,
  'supercluster_markov': 'PC_07GIR5'},
 855: {'node_id': 'PC_000XMQ',
  'node_int': 855,
  'nedges': 86,
  'supercluster_markov': 'PC_06QKPS'},
 856: {'node_id': 'PC_000XN1',
  'node_int': 856,
  'nedges': 50,
  'supercluster_markov': 'PC_0285D9'},
 857: {'node_id': 'PC_000XNN',
  'node_int': 857,
  'nedges': 55,
  'supercluster_markov': 'PC_000XNN'},
 858: {'node_id': 'PC_000XOI',
  'node_int': 858,
  'nedges': 39,
  'supercluster_markov': 'PC_01QCOF'},
 859: {'node_id': 'PC_000XPT',
  'node_int': 859,
  'nedges': 187,
  'supercluster_markov': 'PC_063MEP'},
 860: {'node_id': 'PC_000XQQ',
  'node_int': 860,
  'nedges': 108,
  'supercluster_markov': 'PC_01CO2B'},
 861: {'node_id': 'PC_000XRE',
  'node_int': 861,
  'nedges': 131,
  'supercluster_markov': 'PC_02NYGI'},
 862: {'node_id': 'PC_000XSI',
  'node_int': 862,
  'nedges': 78,
  'supercluster_markov': 'PC_013Y2D'},
 863: {'node_id': 'PC_000XSY',
  'node_int': 863,
  'nedges': 50,
  'supercluster_markov': 'PC_005IKK'},
 864: {'node_id': 'PC_000XT0',
  'node_int': 864,
  'nedges': 51,
  'supercluster_markov': 'PC_04G5AJ'},
 865: {'node_id': 'PC_000XU9',
  'node_int': 865,
  'nedges': 51,
  'supercluster_markov': 'PC_034IQ4'},
 866: {'node_id': 'PC_000XZ2',
  'node_int': 866,
  'nedges': 120,
  'supercluster_markov': 'PC_04D8YQ'},
 867: {'node_id': 'PC_000XZK',
  'node_int': 867,
  'nedges': 50,
  'supercluster_markov': 'PC_04Z1TZ'},
 868: {'node_id': 'PC_000Y01',
  'node_int': 868,
  'nedges': 50,
  'supercluster_markov': 'PC_0172Q2'},
 869: {'node_id': 'PC_000Y0D',
  'node_int': 869,
  'nedges': 45,
  'supercluster_markov': 'PC_048HVT'},
 870: {'node_id': 'PC_000Y2X',
  'node_int': 870,
  'nedges': 53,
  'supercluster_markov': 'PC_01D6DG'},
 871: {'node_id': 'PC_000Y35',
  'node_int': 871,
  'nedges': 57,
  'supercluster_markov': 'PC_00TVEO'},
 872: {'node_id': 'PC_000Y5X',
  'node_int': 872,
  'nedges': 177,
  'supercluster_markov': 'PC_012C2G'},
 873: {'node_id': 'PC_000Y5Y',
  'node_int': 873,
  'nedges': 28,
  'supercluster_markov': 'PC_07X1BJ'},
 874: {'node_id': 'PC_000Y6Q',
  'node_int': 874,
  'nedges': 63,
  'supercluster_markov': 'PC_030YZB'},
 875: {'node_id': 'PC_000Y7Z',
  'node_int': 875,
  'nedges': 68,
  'supercluster_markov': 'PC_04LZKI'},
 876: {'node_id': 'PC_000Y8P',
  'node_int': 876,
  'nedges': 55,
  'supercluster_markov': 'PC_04CJAG'},
 877: {'node_id': 'PC_000Y9L',
  'node_int': 877,
  'nedges': 99,
  'supercluster_markov': 'PC_06LE4A'},
 878: {'node_id': 'PC_000YA9',
  'node_int': 878,
  'nedges': 51,
  'supercluster_markov': 'PC_00DE50'},
 879: {'node_id': 'PC_000YAX',
  'node_int': 879,
  'nedges': 52,
  'supercluster_markov': 'PC_063MEP'},
 880: {'node_id': 'PC_000YC8',
  'node_int': 880,
  'nedges': 54,
  'supercluster_markov': 'PC_06MVYI'},
 881: {'node_id': 'PC_000YG7',
  'node_int': 881,
  'nedges': 50,
  'supercluster_markov': 'PC_001KCV'},
 882: {'node_id': 'PC_000YGO',
  'node_int': 882,
  'nedges': 51,
  'supercluster_markov': 'PC_0229E6'},
 883: {'node_id': 'PC_000YGU',
  'node_int': 883,
  'nedges': 50,
  'supercluster_markov': 'PC_02HSBP'},
 884: {'node_id': 'PC_000YLZ',
  'node_int': 884,
  'nedges': 53,
  'supercluster_markov': 'PC_02IVE7'},
 885: {'node_id': 'PC_000YMQ',
  'node_int': 885,
  'nedges': 51,
  'supercluster_markov': 'PC_04PABL'},
 886: {'node_id': 'PC_000YOO',
  'node_int': 886,
  'nedges': 51,
  'supercluster_markov': 'PC_02XEG8'},
 887: {'node_id': 'PC_000YOY',
  'node_int': 887,
  'nedges': 56,
  'supercluster_markov': 'PC_02WI85'},
 888: {'node_id': 'PC_000YT0',
  'node_int': 888,
  'nedges': 433,
  'supercluster_markov': 'PC_017I2K'},
 889: {'node_id': 'PC_000YTV',
  'node_int': 889,
  'nedges': 79,
  'supercluster_markov': 'PC_041LAD'},
 890: {'node_id': 'PC_000YU8',
  'node_int': 890,
  'nedges': 54,
  'supercluster_markov': 'PC_055Y72'},
 891: {'node_id': 'PC_000YUG',
  'node_int': 891,
  'nedges': 50,
  'supercluster_markov': 'PC_06GFLZ'},
 892: {'node_id': 'PC_000YV4',
  'node_int': 892,
  'nedges': 51,
  'supercluster_markov': 'PC_02FBX8'},
 893: {'node_id': 'PC_000YVR',
  'node_int': 893,
  'nedges': 50,
  'supercluster_markov': 'PC_02V0X3'},
 894: {'node_id': 'PC_000YWM',
  'node_int': 894,
  'nedges': 65,
  'supercluster_markov': 'PC_010T6X'},
 895: {'node_id': 'PC_000YWW',
  'node_int': 895,
  'nedges': 54,
  'supercluster_markov': 'PC_06B3ON'},
 896: {'node_id': 'PC_000YYP',
  'node_int': 896,
  'nedges': 55,
  'supercluster_markov': 'PC_05I2V5'},
 897: {'node_id': 'PC_000Z0Q',
  'node_int': 897,
  'nedges': 52,
  'supercluster_markov': 'PC_030X3X'},
 898: {'node_id': 'PC_000Z1P',
  'node_int': 898,
  'nedges': 93,
  'supercluster_markov': 'PC_017I2K'},
 899: {'node_id': 'PC_000Z2Q',
  'node_int': 899,
  'nedges': 42,
  'supercluster_markov': 'PC_08CK32'},
 900: {'node_id': 'PC_000Z2X',
  'node_int': 900,
  'nedges': 52,
  'supercluster_markov': 'PC_04IGFB'},
 901: {'node_id': 'PC_000Z3H',
  'node_int': 901,
  'nedges': 53,
  'supercluster_markov': 'PC_034NST'},
 902: {'node_id': 'PC_000Z84',
  'node_int': 902,
  'nedges': 78,
  'supercluster_markov': 'PC_056GI0'},
 903: {'node_id': 'PC_000Z8L',
  'node_int': 903,
  'nedges': 46,
  'supercluster_markov': 'PC_00GBAC'},
 904: {'node_id': 'PC_000ZAP',
  'node_int': 904,
  'nedges': 38,
  'supercluster_markov': 'PC_08GZES'},
 905: {'node_id': 'PC_000ZC7',
  'node_int': 905,
  'nedges': 50,
  'supercluster_markov': 'PC_030WXE'},
 906: {'node_id': 'PC_000ZHF',
  'node_int': 906,
  'nedges': 53,
  'supercluster_markov': 'PC_03PCDG'},
 907: {'node_id': 'PC_000ZIA',
  'node_int': 907,
  'nedges': 51,
  'supercluster_markov': 'PC_01LOWK'},
 908: {'node_id': 'PC_000ZM0',
  'node_int': 908,
  'nedges': 349,
  'supercluster_markov': 'PC_05JLZ9'},
 909: {'node_id': 'PC_000ZO0',
  'node_int': 909,
  'nedges': 58,
  'supercluster_markov': 'PC_04YWEL'},
 910: {'node_id': 'PC_000ZO6',
  'node_int': 910,
  'nedges': 130,
  'supercluster_markov': 'PC_03S7G4'},
 911: {'node_id': 'PC_000ZOS',
  'node_int': 911,
  'nedges': 52,
  'supercluster_markov': 'PC_01JBJM'},
 912: {'node_id': 'PC_000ZQC',
  'node_int': 912,
  'nedges': 72,
  'supercluster_markov': 'PC_011PFM'},
 913: {'node_id': 'PC_000ZQT',
  'node_int': 913,
  'nedges': 50,
  'supercluster_markov': 'PC_017I2K'},
 914: {'node_id': 'PC_000ZRK',
  'node_int': 914,
  'nedges': 50,
  'supercluster_markov': 'PC_07ZHMV'},
 915: {'node_id': 'PC_000ZTY',
  'node_int': 915,
  'nedges': 72,
  'supercluster_markov': 'PC_011WBU'},
 916: {'node_id': 'PC_000ZUJ',
  'node_int': 916,
  'nedges': 50,
  'supercluster_markov': 'PC_00Q1S8'},
 917: {'node_id': 'PC_000ZUS',
  'node_int': 917,
  'nedges': 86,
  'supercluster_markov': 'PC_04ZF7E'},
 918: {'node_id': 'PC_000ZV8',
  'node_int': 918,
  'nedges': 65,
  'supercluster_markov': 'PC_065PL9'},
 919: {'node_id': 'PC_000ZVS',
  'node_int': 919,
  'nedges': 51,
  'supercluster_markov': 'PC_03S7G4'},
 920: {'node_id': 'PC_000ZYH',
  'node_int': 920,
  'nedges': 72,
  'supercluster_markov': 'PC_003N2B'},
 921: {'node_id': 'PC_000ZZU',
  'node_int': 921,
  'nedges': 17,
  'supercluster_markov': 'PC_02I8J5'},
 922: {'node_id': 'PC_00101A',
  'node_int': 922,
  'nedges': 215,
  'supercluster_markov': 'PC_02VGIH'},
 923: {'node_id': 'PC_00103B',
  'node_int': 923,
  'nedges': 59,
  'supercluster_markov': 'PC_04GOD5'},
 924: {'node_id': 'PC_001059',
  'node_int': 924,
  'nedges': 51,
  'supercluster_markov': 'PC_01SJJ6'},
 925: {'node_id': 'PC_00105Y',
  'node_int': 925,
  'nedges': 50,
  'supercluster_markov': 'PC_011RZ1'},
 926: {'node_id': 'PC_0010BN',
  'node_int': 926,
  'nedges': 51,
  'supercluster_markov': 'PC_04Z1TZ'},
 927: {'node_id': 'PC_0010CZ',
  'node_int': 927,
  'nedges': 49,
  'supercluster_markov': 'PC_060U0G'},
 928: {'node_id': 'PC_0010DA',
  'node_int': 928,
  'nedges': 31,
  'supercluster_markov': 'PC_069JHU'},
 929: {'node_id': 'PC_0010HG',
  'node_int': 929,
  'nedges': 52,
  'supercluster_markov': 'PC_047UYC'},
 930: {'node_id': 'PC_0010K4',
  'node_int': 930,
  'nedges': 51,
  'supercluster_markov': 'PC_02XYVD'},
 931: {'node_id': 'PC_0010M4',
  'node_int': 931,
  'nedges': 71,
  'supercluster_markov': 'PC_01OCQ4'},
 932: {'node_id': 'PC_0010MF',
  'node_int': 932,
  'nedges': 50,
  'supercluster_markov': 'PC_06DIZZ'},
 933: {'node_id': 'PC_0010MM',
  'node_int': 933,
  'nedges': 52,
  'supercluster_markov': 'PC_00EXEJ'},
 934: {'node_id': 'PC_0010MT',
  'node_int': 934,
  'nedges': 61,
  'supercluster_markov': 'PC_01AQ9R'},
 935: {'node_id': 'PC_0010OJ',
  'node_int': 935,
  'nedges': 60,
  'supercluster_markov': 'PC_034PGU'},
 936: {'node_id': 'PC_0010QD',
  'node_int': 936,
  'nedges': 51,
  'supercluster_markov': 'PC_063MEP'},
 937: {'node_id': 'PC_0010QI',
  'node_int': 937,
  'nedges': 50,
  'supercluster_markov': 'PC_04SOJA'},
 938: {'node_id': 'PC_0010RL',
  'node_int': 938,
  'nedges': 136,
  'supercluster_markov': 'PC_05FOR2'},
 939: {'node_id': 'PC_0010T8',
  'node_int': 939,
  'nedges': 55,
  'supercluster_markov': 'PC_07110D'},
 940: {'node_id': 'PC_0010U4',
  'node_int': 940,
  'nedges': 41,
  'supercluster_markov': 'PC_06OA4L'},
 941: {'node_id': 'PC_0010X2',
  'node_int': 941,
  'nedges': 60,
  'supercluster_markov': 'PC_020YH4'},
 942: {'node_id': 'PC_0010XG',
  'node_int': 942,
  'nedges': 55,
  'supercluster_markov': 'PC_00WD4L'},
 943: {'node_id': 'PC_001134',
  'node_int': 943,
  'nedges': 59,
  'supercluster_markov': 'PC_02OA60'},
 944: {'node_id': 'PC_00114L',
  'node_int': 944,
  'nedges': 132,
  'supercluster_markov': 'PC_05D9O6'},
 945: {'node_id': 'PC_00114Y',
  'node_int': 945,
  'nedges': 50,
  'supercluster_markov': 'PC_03UPZM'},
 946: {'node_id': 'PC_001158',
  'node_int': 946,
  'nedges': 54,
  'supercluster_markov': 'PC_00JS7X'},
 947: {'node_id': 'PC_00115S',
  'node_int': 947,
  'nedges': 50,
  'supercluster_markov': 'PC_04RUJN'},
 948: {'node_id': 'PC_00117L',
  'node_int': 948,
  'nedges': 51,
  'supercluster_markov': 'PC_044QTV'},
 949: {'node_id': 'PC_00118C',
  'node_int': 949,
  'nedges': 4,
  'supercluster_markov': 'PC_00118C'},
 950: {'node_id': 'PC_0011BV',
  'node_int': 950,
  'nedges': 54,
  'supercluster_markov': 'PC_01I3EE'},
 951: {'node_id': 'PC_0011C1',
  'node_int': 951,
  'nedges': 50,
  'supercluster_markov': 'PC_04ZPF1'},
 952: {'node_id': 'PC_0011D4',
  'node_int': 952,
  'nedges': 37,
  'supercluster_markov': 'PC_086EZG'},
 953: {'node_id': 'PC_0011DG',
  'node_int': 953,
  'nedges': 50,
  'supercluster_markov': 'PC_05HQOC'},
 954: {'node_id': 'PC_0011FJ',
  'node_int': 954,
  'nedges': 50,
  'supercluster_markov': 'PC_034NST'},
 955: {'node_id': 'PC_0011FM',
  'node_int': 955,
  'nedges': 52,
  'supercluster_markov': 'PC_086CHL'},
 956: {'node_id': 'PC_0011FQ',
  'node_int': 956,
  'nedges': 67,
  'supercluster_markov': 'PC_05SBEA'},
 957: {'node_id': 'PC_0011GM',
  'node_int': 957,
  'nedges': 54,
  'supercluster_markov': 'PC_017I2K'},
 958: {'node_id': 'PC_0011IK',
  'node_int': 958,
  'nedges': 164,
  'supercluster_markov': 'PC_06GTNH'},
 959: {'node_id': 'PC_0011L7',
  'node_int': 959,
  'nedges': 54,
  'supercluster_markov': 'PC_07VNQT'},
 960: {'node_id': 'PC_0011LP',
  'node_int': 960,
  'nedges': 50,
  'supercluster_markov': 'PC_03N0NA'},
 961: {'node_id': 'PC_0011P5',
  'node_int': 961,
  'nedges': 67,
  'supercluster_markov': 'PC_050QUH'},
 962: {'node_id': 'PC_0011Q3',
  'node_int': 962,
  'nedges': 6,
  'supercluster_markov': 'PC_08BE85'},
 963: {'node_id': 'PC_0011RK',
  'node_int': 963,
  'nedges': 55,
  'supercluster_markov': 'PC_0011RK'},
 964: {'node_id': 'PC_0011RP',
  'node_int': 964,
  'nedges': 50,
  'supercluster_markov': 'PC_04IGFB'},
 965: {'node_id': 'PC_0011TF',
  'node_int': 965,
  'nedges': 50,
  'supercluster_markov': 'PC_05MOBP'},
 966: {'node_id': 'PC_0011UC',
  'node_int': 966,
  'nedges': 67,
  'supercluster_markov': 'PC_04LDFD'},
 967: {'node_id': 'PC_0011VW',
  'node_int': 967,
  'nedges': 52,
  'supercluster_markov': 'PC_04YDES'},
 968: {'node_id': 'PC_0011WS',
  'node_int': 968,
  'nedges': 50,
  'supercluster_markov': 'PC_04LRLB'},
 969: {'node_id': 'PC_0011Y5',
  'node_int': 969,
  'nedges': 45,
  'supercluster_markov': 'PC_02XYVD'},
 970: {'node_id': 'PC_00120B',
  'node_int': 970,
  'nedges': 50,
  'supercluster_markov': 'PC_06MCAB'},
 971: {'node_id': 'PC_00120F',
  'node_int': 971,
  'nedges': 52,
  'supercluster_markov': 'PC_033V7U'},
 972: {'node_id': 'PC_00126F',
  'node_int': 972,
  'nedges': 25,
  'supercluster_markov': 'PC_037RQE'},
 973: {'node_id': 'PC_00128R',
  'node_int': 973,
  'nedges': 56,
  'supercluster_markov': 'PC_06I84L'},
 974: {'node_id': 'PC_001299',
  'node_int': 974,
  'nedges': 67,
  'supercluster_markov': 'PC_045LJ7'},
 975: {'node_id': 'PC_0012DW',
  'node_int': 975,
  'nedges': 50,
  'supercluster_markov': 'PC_04VN26'},
 976: {'node_id': 'PC_0012FY',
  'node_int': 976,
  'nedges': 84,
  'supercluster_markov': 'PC_000JRF'},
 977: {'node_id': 'PC_0012I9',
  'node_int': 977,
  'nedges': 79,
  'supercluster_markov': 'PC_01LNCE'},
 978: {'node_id': 'PC_0012IQ',
  'node_int': 978,
  'nedges': 50,
  'supercluster_markov': 'PC_04KZQH'},
 979: {'node_id': 'PC_0012K8',
  'node_int': 979,
  'nedges': 53,
  'supercluster_markov': 'PC_01DU6J'},
 980: {'node_id': 'PC_0012L0',
  'node_int': 980,
  'nedges': 199,
  'supercluster_markov': 'PC_054B1U'},
 981: {'node_id': 'PC_0012L5',
  'node_int': 981,
  'nedges': 53,
  'supercluster_markov': 'PC_03MUEB'},
 982: {'node_id': 'PC_0012M8',
  'node_int': 982,
  'nedges': 55,
  'supercluster_markov': 'PC_02DN65'},
 983: {'node_id': 'PC_0012NC',
  'node_int': 983,
  'nedges': 51,
  'supercluster_markov': 'PC_0519Z5'},
 984: {'node_id': 'PC_0012OH',
  'node_int': 984,
  'nedges': 9,
  'supercluster_markov': 'PC_05KIFB'},
 985: {'node_id': 'PC_0012OK',
  'node_int': 985,
  'nedges': 71,
  'supercluster_markov': 'PC_0012OK'},
 986: {'node_id': 'PC_0012OT',
  'node_int': 986,
  'nedges': 44,
  'supercluster_markov': 'PC_06XYC4'},
 987: {'node_id': 'PC_0012PS',
  'node_int': 987,
  'nedges': 152,
  'supercluster_markov': 'PC_011RZ1'},
 988: {'node_id': 'PC_0012Q9',
  'node_int': 988,
  'nedges': 83,
  'supercluster_markov': 'PC_01DR6B'},
 989: {'node_id': 'PC_0012QJ',
  'node_int': 989,
  'nedges': 50,
  'supercluster_markov': 'PC_001KCV'},
 990: {'node_id': 'PC_0012RG',
  'node_int': 990,
  'nedges': 68,
  'supercluster_markov': 'PC_01CO2B'},
 991: {'node_id': 'PC_0012S0',
  'node_int': 991,
  'nedges': 89,
  'supercluster_markov': 'PC_06BAI6'},
 992: {'node_id': 'PC_0012VT',
  'node_int': 992,
  'nedges': 50,
  'supercluster_markov': 'PC_02AYBU'},
 993: {'node_id': 'PC_0012X9',
  'node_int': 993,
  'nedges': 75,
  'supercluster_markov': 'PC_04MW87'},
 994: {'node_id': 'PC_0012ZT',
  'node_int': 994,
  'nedges': 119,
  'supercluster_markov': 'PC_01P6AC'},
 995: {'node_id': 'PC_00133A',
  'node_int': 995,
  'nedges': 50,
  'supercluster_markov': 'PC_044FPA'},
 996: {'node_id': 'PC_001356',
  'node_int': 996,
  'nedges': 53,
  'supercluster_markov': 'PC_08DCNT'},
 997: {'node_id': 'PC_00135T',
  'node_int': 997,
  'nedges': 118,
  'supercluster_markov': 'PC_003VWA'},
 998: {'node_id': 'PC_00135Z',
  'node_int': 998,
  'nedges': 165,
  'supercluster_markov': 'PC_02G2TC'},
 999: {'node_id': 'PC_00136N',
  'node_int': 999,
  'nedges': 50,
  'supercluster_markov': 'PC_04CNN8'},
 ...}
```

In [32]:

```
nodes_df = pd.DataFrame(nodes.values())
nodes_df = nodes_df[['supercluster_markov', 'node_id', 'node_int', 'nedges']]
nodes_df
```

Out[32]:

|  | supercluster\_markov | node\_id | node\_int | nedges |
| --- | --- | --- | --- | --- |
| 0 | PC\_05C4OY | PC\_000008 | 0 | 50 |
| 1 | PC\_03EK7O | PC\_00000G | 1 | 57 |
| 2 | PC\_03HSAJ | PC\_00001B | 2 | 97 |
| 3 | PC\_002AG8 | PC\_00002Z | 3 | 58 |
| 4 | PC\_06GTNH | PC\_000033 | 4 | 101 |
| ... | ... | ... | ... | ... |
| 283663 | PC\_06VD2C | PC\_08L6ZL | 283663 | 98 |
| 283664 | PC\_016G2J | PC\_08L6ZT | 283664 | 50 |
| 283665 | PC\_048DCU | PC\_08L705 | 283665 | 122 |
| 283666 | PC\_011RZ1 | PC\_08L70J | 283666 | 50 |
| 283667 | PC\_0229E6 | PC\_08L712 | 283667 | 52 |

283668 rows × 4 columns

In [33]:

```
effector_df = (
    pd.read_csv("./07-find_effector_clusters-effector_clusters.tsv", sep="\t")
    [["cluster", "pc", "effector", "effector_database"]]
    .drop_duplicates()
)

effector_df = pd.merge(
    nodes_df.rename(columns={"node_id": "cluster", "node_int": "cluster_int"}),
    effector_df,
    on="cluster",
    how="outer"
)

effector_df.sort_values(["supercluster_markov", "cluster", "nedges"], inplace=True)
effector_df.to_csv("09-truncated_clustering-nodes.tsv", sep="\t", index=False)
effector_df
```

Out[33]:

|  | supercluster\_markov | cluster | cluster\_int | nedges | pc | effector | effector\_database |
| --- | --- | --- | --- | --- | --- | --- | --- |
| 8 | PC\_000089 | PC\_000089 | 8.0 | 4.0 | NaN | NaN | NaN |
| 55 | PC\_0001PA | PC\_0001PA | 55.0 | 1.0 | NaN | NaN | NaN |
| 102 | PC\_0002Z4 | PC\_0002Z4 | 102.0 | 10.0 | NaN | NaN | NaN |
| 140266 | PC\_0002Z4 | PC\_0432AV | 140005.0 | 5.0 | NaN | NaN | NaN |
| 204440 | PC\_0002Z4 | PC\_05Y1QN | 204038.0 | 10.0 | NaN | NaN | NaN |
| ... | ... | ... | ... | ... | ... | ... | ... |
| 284682 | NaN | PC\_08L2AF | NaN | NaN | PC\_00FYLP | G4NF22#PHI:5662 | phibase |
| 285024 | NaN | PC\_08L65P | NaN | NaN | PC\_02590L | Q5AJ90#PHI:3688 | phibase |
| 286489 | NaN | NaN | NaN | NaN | PC\_03ZM2Y | Q4WT66#PHI:2511 | phibase |
| 286490 | NaN | NaN | NaN | NaN | PC\_05DQYK | Q6Q8B9#PHI:366 | phibase |
| 286491 | NaN | NaN | NaN | NaN | PC\_06QVYP | I1SAJ7#PHI:9042 | phibase |

286492 rows × 7 columns

In [45]:

```
pairs.to_csv("09-truncated_clustering-edges.tsv", sep="\t", index=False)
```

In [34]:

```
df = effector_df.copy()
df.sort_values(
    ['supercluster_markov', 'cluster', 'nedges', "effector_database", "effector"],
    inplace=True
)
df.loc[df["effector"].notnull(), "effector"] = df[df["effector"].notnull()].apply(lambda x: f"{x['effector_database']}|{x['effector']}", axis=1)

df.loc[df["effector"].isnull(), "effector"] = "."

df = (
    df
    .groupby(['supercluster_markov', 'cluster', 'cluster_int', 'nedges'])
    ["effector"]
    .apply(lambda x: ",".join(xi for xi in x if xi != "."))
    .reset_index()
)

df["cluster_int"] = df["cluster_int"].astype(int)
df["nedges"] = df["nedges"].astype(int)
df.loc[df["effector"] == "", "effector"] = None

df.to_csv("09-truncated_clustering-nodes_grouped.tsv", sep="\t", index=False)
nodes = df

nodes.set_index("cluster_int", inplace=True)
nodes.sort_index(inplace=True)
nodes
```

Out[34]:

|  | supercluster\_markov | cluster | nedges | effector |
| --- | --- | --- | --- | --- |
| cluster\_int |  |  |  |  |
| 0 | PC\_05C4OY | PC\_000008 | 50 | None |
| 1 | PC\_03EK7O | PC\_00000G | 57 | None |
| 2 | PC\_03HSAJ | PC\_00001B | 97 | None |
| 3 | PC\_002AG8 | PC\_00002Z | 58 | None |
| 4 | PC\_06GTNH | PC\_000033 | 101 | None |
| ... | ... | ... | ... | ... |
| 283663 | PC\_06VD2C | PC\_08L6ZL | 98 | None |
| 283664 | PC\_016G2J | PC\_08L6ZT | 50 | None |
| 283665 | PC\_048DCU | PC\_08L705 | 122 | None |
| 283666 | PC\_011RZ1 | PC\_08L70J | 50 | None |
| 283667 | PC\_0229E6 | PC\_08L712 | 52 | None |

283668 rows × 4 columns

In [35]:

```
nodes[nodes["effector"].notnull()]
```

Out[35]:

|  | supercluster\_markov | cluster | nedges | effector |
| --- | --- | --- | --- | --- |
| cluster\_int |  |  |  |  |
| 249 | PC\_07OBRT | PC\_0008CG | 108 | phibase|O74238#PHI:3221 |
| 491 | PC\_05QCZN | PC\_000I01 | 50 | phibase|I1RB21#PHI:1966 |
| 569 | PC\_000L8O | PC\_000L8O | 287 | phibase|I1RPX8#PHI:1581 |
| 751 | PC\_03O094 | PC\_000T85 | 63 | phibase|G4MYY5#PHI:7993 |
| 826 | PC\_05VP7B | PC\_000WAJ | 84 | phibase|I1SAE7#PHI:1725 |
| ... | ... | ... | ... | ... |
| 281482 | PC\_019LQZ | PC\_08K18Q | 251 | phibase|D2SZX7#PHI:2837 |
| 283375 | PC\_040773 | PC\_08KUF0 | 56 | phibase|I1RJM9#PHI:1221 |
| 283401 | PC\_037RQE | PC\_08KWX7 | 95 | phibase|F9XJV5#PHI:2441 |
| 283435 | PC\_027WLR | PC\_08KYY1 | 60 | phibase|E9EWM2#PHI:5430,phibase|H1VKZ0#PHI:671... |
| 283506 | PC\_07O88I | PC\_08L1R9 | 3 | custom|M.BR29.EuGene\_00087671,custom|MGG\_18060 |

1060 rows × 4 columns

In [38]:

```
for node in G.nodes:
    G.nodes[node]["supercluster_markov"] = str(nodes.loc[node, "supercluster_markov"])
    G.nodes[node]["cluster"] = str(nodes.loc[node, "cluster"])
    G.nodes[node]["nedges"] = int(nodes.loc[node, "nedges"])
    G.nodes[node]["effector"] = str(nodes.loc[node, "effector"]) if nodes.loc[node, "effector"] is not None else ""
```

In [39]:

```
G.nodes[0]
```

Out[39]:

```
{'supercluster_markov': 'PC_05C4OY',
 'cluster': 'PC_000008',
 'nedges': 50,
 'effector': ''}
```

In [42]:

```
for i, row in pairs.iterrows():
    e = G.edges[(row["maxid_int"], row["minid_int"])]
    e["minid_cov"] = row["minid_cov"]
    e["maxid_cov"] = row["maxid_cov"]
```

In [43]:

```
subset_nodes = nodes[nodes["supercluster_markov"].isin(set(nodes.loc[nodes["effector"].notnull(), "supercluster_markov"]))].index
G_sub = nx.subgraph(G, subset_nodes)
nx.write_graphml(G_sub, "09-truncated_clustering_subset.graphml")
```

In [44]:

```
nx.write_graphml(G, "09-truncated_clustering.graphml")
```

In [ ]:

```

```

In [ ]:

```

```
