# Supplementary material for: Remote homology clustering identifies lowly conserved families of effector proteins in plant-pathogenic fungi
Source: Microb Genom. 2021 Sep 1;7(9):000637. doi: 10.1099/mgen.0.000637 (PMC8715435; doi:10.1099/mgen.0.000637)
Supplement: Supplementary material 12 [file mgen-7-0637-s0012.zip › supplementary_data_06-remeff_scripts/06a-normalise_scores.html]

06a-normalise\_scores


We're trying to find a normalised score that we can use for ranking the matches.
It needs to be independent of HMM-length and we want to find a way of combining scores so that matched pairs can have a single score.

In [1]:

```
from math import ceil

import pandas as pd
import numpy as np
import seaborn as sns
import scipy as sp
from scipy.stats import linregress
import matplotlib.pyplot as plt
```

In [2]:

```
b10 = pd.read_csv(
    "./05b-best_10.tsv",
    sep="\t",
    names=[
        "min_id",
        "max_id",
        "query_id",
        "template_id",
        "probability",
        "evalue",
        "score",
        "query_start",
        "query_end",
        "query_length",
        "query_cov",
        "template_start",
        "template_end",
        "template_length",
        "template_cov",
    ]
)[["min_id", "max_id", "query_id", "template_id", "query_length", "template_length", "probability", "score"]]

b10["lqh"] = b10["query_length"] * b10["template_length"]
b10["log_lqh"] = np.log10(b10["lqh"])

b10["log_score"] = np.log10(b10["score"])

b10["cut"] = np.arange(len(b10)) // 1000

b10.sort_values("log_lqh", ascending=False, inplace=True)

b10.head()
```

Out[2]:

|  | min\_id | max\_id | query\_id | template\_id | query\_length | template\_length | probability | score | lqh | log\_lqh | log\_score | cut |
| --- | --- | --- | --- | --- | --- | --- | --- | --- | --- | --- | --- | --- |
| 4206636 | PC\_03V69G | PC\_07CY3I | PC\_07CY3I | PC\_03V69G | 3268 | 3239 | 100.0 | 5974.23 | 10585052 | 7.024693 | 3.776282 | 4206 |
| 2266054 | PC\_03V69G | PC\_07CY3I | PC\_03V69G | PC\_07CY3I | 3239 | 3268 | 100.0 | 5961.17 | 10585052 | 7.024693 | 3.775332 | 2266 |
| 4206698 | PC\_03HL8P | PC\_07CY3I | PC\_07CY3I | PC\_03HL8P | 3268 | 3235 | 100.0 | 4018.43 | 10571980 | 7.024156 | 3.604056 | 4206 |
| 2045989 | PC\_03HL8P | PC\_07CY3I | PC\_03HL8P | PC\_07CY3I | 3235 | 3268 | 100.0 | 4012.13 | 10571980 | 7.024156 | 3.603375 | 2045 |
| 4206632 | PC\_05KLV7 | PC\_07CY3I | PC\_07CY3I | PC\_05KLV7 | 3268 | 3234 | 100.0 | 6604.16 | 10568712 | 7.024022 | 3.819818 | 4206 |

In [3]:

```
bself = pd.read_csv(
    "./04a-select_reciprocal_hits-self.tsv",
    sep="\t",
    names=[
        "min_id",
        "max_id",
        "query_id",
        "template_id",
        "probability",
        "evalue",
        "score",
        "query_start",
        "query_end",
        "query_length",
        "query_cov",
        "template_start",
        "template_end",
        "template_length",
        "template_cov",
    ]
)[["min_id", "max_id", "query_id", "template_id", "query_length", "template_length", "probability", "score"]]


bself["lqh"] = bself["query_length"] * bself["template_length"]
bself["log_lqh"] = np.log10(bself["lqh"])

bself["log_score"] = np.log10(bself["score"])

bself["cut"] = np.arange(len(bself)) // 1000

bself.sort_values("log_lqh", ascending=False, inplace=True)

bself.head()
```

Out[3]:

|  | min\_id | max\_id | query\_id | template\_id | query\_length | template\_length | probability | score | lqh | log\_lqh | log\_score | cut |
| --- | --- | --- | --- | --- | --- | --- | --- | --- | --- | --- | --- | --- |
| 108737 | PC\_036O1W | PC\_036O1W | PC\_036O1W | PC\_036O1W | 3279 | 3279 | 100.0 | 10983.52 | 10751841 | 7.031483 | 4.040742 | 108 |
| 175696 | PC\_055J9A | PC\_055J9A | PC\_055J9A | PC\_055J9A | 3278 | 3278 | 100.0 | 10693.89 | 10745284 | 7.031218 | 4.029136 | 175 |
| 24123 | PC\_00P9BY | PC\_00P9BY | PC\_00P9BY | PC\_00P9BY | 3273 | 3273 | 100.0 | 10873.78 | 10712529 | 7.029892 | 4.036381 | 24 |
| 102375 | PC\_02ZW9K | PC\_02ZW9K | PC\_02ZW9K | PC\_02ZW9K | 3272 | 3272 | 100.0 | 10933.41 | 10705984 | 7.029627 | 4.038756 | 102 |
| 276527 | PC\_08H5WR | PC\_08H5WR | PC\_08H5WR | PC\_08H5WR | 3271 | 3271 | 100.0 | 11613.38 | 10699441 | 7.029361 | 4.064959 | 276 |

So the data are prepared. Lets look at the distributions of our candidate score strategies.
Probability, score, and score / qlen

In [4]:

```
b10.sample(n=10000)["probability"].plot.kde()
```

Out[4]:

```
<matplotlib.axes._subplots.AxesSubplot at 0x7f3c9b2289a0>
```

In [5]:

```
bself.sample(n=10000)["probability"].plot.kde()
```

Out[5]:

```
<matplotlib.axes._subplots.AxesSubplot at 0x7f3c990f0a00>
```

This is highly skewed (probably as a result of our using the "top"-50 matches as input.
I suspect that probability won't give enough separation of the very high scores.

In [6]:

```
b10.sample(n=10000)["score"].plot.kde()
```

Out[6]:

```
<matplotlib.axes._subplots.AxesSubplot at 0x7f3c990db3d0>
```

In [7]:

```
bself.sample(n=10000)["score"].plot.kde()
```

Out[7]:

```
<matplotlib.axes._subplots.AxesSubplot at 0x7f3c99031670>
```

This score is skewed in the other direction, though i suppose that's closer to what we want really.

In [8]:

```
b10.sample(n=10000)["log_score"].plot.kde()
```

Out[8]:

```
<matplotlib.axes._subplots.AxesSubplot at 0x7f3c99003130>
```

In [9]:

```
bself.sample(n=10000)["log_score"].plot.kde()
```

Out[9]:

```
<matplotlib.axes._subplots.AxesSubplot at 0x7f3c98fc3430>
```

The log score does seem to be roughly normally distributed, which might give us some nice properties.

In [10]:

```
(b10["score"] / b10["query_length"]).sample(n=10000).plot.kde()
```

Out[10]:

```
<matplotlib.axes._subplots.AxesSubplot at 0x7f3c98f3c1f0>
```

In [11]:

```
(b10["score"] / b10["template_length"]).sample(n=10000).plot.kde()
```

Out[11]:

```
<matplotlib.axes._subplots.AxesSubplot at 0x7f3c98f3c190>
```

Both length normalised scores are skewed and might be a bit difficult to work with.

In [12]:

```
(b10["score"] * b10["probability"]).sample(n=10000).plot.kde()
```

Out[12]:

```
<matplotlib.axes._subplots.AxesSubplot at 0x7f3c98ed5df0>
```

It might be a bit dicey to combine the probability and score, since probability is derived from score.
But anyway.

In [13]:

```
b10.sample(n=10000).plot("log_score", "probability", kind="scatter")
```

Out[13]:

```
<matplotlib.axes._subplots.AxesSubplot at 0x7f3c98e3db80>
```

The probability and score seem to generally give similar information.
Probability seems to give better discrimination until we get to 100%, where the probability has maxed out and the logscore continues to be useful.
We know from the density plot that most probabilities are very close to 100%.

## Length dependence on scores.¶

In [14]:

```
b10.sample(n=10000).plot("lqh", "score", kind="scatter")
```

Out[14]:

```
<matplotlib.axes._subplots.AxesSubplot at 0x7f3c98e12700>
```

In [15]:

```
bself.sample(n=10000).plot("lqh", "score", kind="scatter")
```

Out[15]:

```
<matplotlib.axes._subplots.AxesSubplot at 0x7f3c98d725e0>
```

This is the raw score vs the product of the two lengths.
It seems that there's a log log relationship, which is what we'd expect based on orthofinders normalisation.

In [16]:

```
b10.sample(n=10000).plot("log_lqh", "log_score", kind="scatter")
```

Out[16]:

```
<matplotlib.axes._subplots.AxesSubplot at 0x7f3c98d5aa60>
```

In [17]:

```
bself.sample(n=10000).plot("log_lqh", "log_score", kind="scatter")
```

Out[17]:

```
<matplotlib.axes._subplots.AxesSubplot at 0x7f3c98e1df10>
```

Yes there's a definite dependence of score on length.
It is linear in the log-log plot.

In [18]:

```
b10.sample(n=10000).plot("log_lqh", "probability", kind="scatter")
```

Out[18]:

```
<matplotlib.axes._subplots.AxesSubplot at 0x7f3c98d87a30>
```

In [19]:

```
bself.sample(n=10000).plot("log_lqh", "probability", kind="scatter")
```

Out[19]:

```
<matplotlib.axes._subplots.AxesSubplot at 0x7f3c98c453d0>
```

There doesn't appear to be a dependence on length for the probability.

## Normalising the score by length¶

Here we're replicating the method used by orthofinder.
We've already sorted alignments the product of both lengths, we bin the whole dataframe into 1000 alignment sized chunks, then take the top 5% matches by score.
We then regress the log length product against the log score to find the correction factors.

I think we'll use the self matches for this.

In [20]:

```
def mapfun(df):
    n = len(df)
    return df.nlargest(ceil(0.05 * n), "log_score")

bself_best = bself.groupby("cut").apply(mapfun)
```

In [21]:

```
reg = linregress(bself_best["log_lqh"], bself_best["log_score"])
reg
```

Out[21]:

```
LinregressResult(slope=0.44383741340701377, intercept=0.8904714660279858, rvalue=0.9101929186244985, pvalue=0.0, stderr=0.0017005829380550086)
```

Now we can apply the transformation to get the normalised score.

In [22]:

```
bself["norm_score"] = bself["score"] / ((10 ** reg.intercept) * (bself["lqh"] ** reg.slope))
bself
```

Out[22]:

|  | min\_id | max\_id | query\_id | template\_id | query\_length | template\_length | probability | score | lqh | log\_lqh | log\_score | cut | norm\_score |
| --- | --- | --- | --- | --- | --- | --- | --- | --- | --- | --- | --- | --- | --- |
| 108737 | PC\_036O1W | PC\_036O1W | PC\_036O1W | PC\_036O1W | 3279 | 3279 | 100.00 | 10983.52 | 10751841 | 7.031483 | 4.040742 | 108 | 1.070126 |
| 175696 | PC\_055J9A | PC\_055J9A | PC\_055J9A | PC\_055J9A | 3278 | 3278 | 100.00 | 10693.89 | 10745284 | 7.031218 | 4.029136 | 175 | 1.042189 |
| 24123 | PC\_00P9BY | PC\_00P9BY | PC\_00P9BY | PC\_00P9BY | 3273 | 3273 | 100.00 | 10873.78 | 10712529 | 7.029892 | 4.036381 | 24 | 1.061158 |
| 102375 | PC\_02ZW9K | PC\_02ZW9K | PC\_02ZW9K | PC\_02ZW9K | 3272 | 3272 | 100.00 | 10933.41 | 10705984 | 7.029627 | 4.038756 | 102 | 1.067267 |
| 276527 | PC\_08H5WR | PC\_08H5WR | PC\_08H5WR | PC\_08H5WR | 3271 | 3271 | 100.00 | 11613.38 | 10699441 | 7.029361 | 4.064959 | 276 | 1.133950 |
| ... | ... | ... | ... | ... | ... | ... | ... | ... | ... | ... | ... | ... | ... |
| 12877 | PC\_00DKGG | PC\_00DKGG | PC\_00DKGG | PC\_00DKGG | 32 | 32 | 100.00 | 152.02 | 1024 | 3.010300 | 2.181901 | 12 | 0.902289 |
| 178105 | PC\_057ZMK | PC\_057ZMK | PC\_057ZMK | PC\_057ZMK | 32 | 32 | 99.92 | 128.83 | 1024 | 3.010300 | 2.110017 | 178 | 0.764648 |
| 224578 | PC\_06L1OP | PC\_06L1OP | PC\_06L1OP | PC\_06L1OP | 31 | 31 | 99.92 | 126.15 | 961 | 2.982723 | 2.100887 | 224 | 0.770143 |
| 123567 | PC\_03MAS8 | PC\_03MAS8 | PC\_03MAS8 | PC\_03MAS8 | 31 | 31 | 99.85 | 114.38 | 961 | 2.982723 | 2.058350 | 123 | 0.698288 |
| 29020 | PC\_00UCED | PC\_00UCED | PC\_00UCED | PC\_00UCED | 31 | 31 | 99.86 | 115.40 | 961 | 2.982723 | 2.062206 | 29 | 0.704515 |

282123 rows × 13 columns

In [23]:

```
b10["norm_score"] = b10["score"] / ((10 ** reg.intercept) * (b10["lqh"] ** reg.slope))
b10
```

Out[23]:

|  | min\_id | max\_id | query\_id | template\_id | query\_length | template\_length | probability | score | lqh | log\_lqh | log\_score | cut | norm\_score |
| --- | --- | --- | --- | --- | --- | --- | --- | --- | --- | --- | --- | --- | --- |
| 4206636 | PC\_03V69G | PC\_07CY3I | PC\_07CY3I | PC\_03V69G | 3268 | 3239 | 100.00 | 5974.23 | 10585052 | 7.024693 | 3.776282 | 4206 | 0.586123 |
| 2266054 | PC\_03V69G | PC\_07CY3I | PC\_03V69G | PC\_07CY3I | 3239 | 3268 | 100.00 | 5961.17 | 10585052 | 7.024693 | 3.775332 | 2266 | 0.584842 |
| 4206698 | PC\_03HL8P | PC\_07CY3I | PC\_07CY3I | PC\_03HL8P | 3268 | 3235 | 100.00 | 4018.43 | 10571980 | 7.024156 | 3.604056 | 4206 | 0.394459 |
| 2045989 | PC\_03HL8P | PC\_07CY3I | PC\_03HL8P | PC\_07CY3I | 3235 | 3268 | 100.00 | 4012.13 | 10571980 | 7.024156 | 3.603375 | 2045 | 0.393840 |
| 4206632 | PC\_05KLV7 | PC\_07CY3I | PC\_07CY3I | PC\_05KLV7 | 3268 | 3234 | 100.00 | 6604.16 | 10568712 | 7.024022 | 3.819818 | 4206 | 0.648369 |
| ... | ... | ... | ... | ... | ... | ... | ... | ... | ... | ... | ... | ... | ... |
| 2600741 | PC\_00L4QY | PC\_04FXZ9 | PC\_04FXZ9 | PC\_00L4QY | 36 | 51 | 99.80 | 117.60 | 1836 | 3.263873 | 2.070407 | 2600 | 0.538650 |
| 4153474 | PC\_04H0SU | PC\_074C3D | PC\_074C3D | PC\_04H0SU | 38 | 41 | 99.67 | 102.28 | 1558 | 3.192567 | 2.009791 | 4153 | 0.503893 |
| 2618095 | PC\_04H0SU | PC\_074C3D | PC\_04H0SU | PC\_074C3D | 41 | 38 | 99.68 | 102.68 | 1558 | 3.192567 | 2.011486 | 2618 | 0.505864 |
| 4153477 | PC\_00NYII | PC\_074C3D | PC\_074C3D | PC\_00NYII | 38 | 33 | 99.46 | 86.33 | 1254 | 3.098298 | 1.936162 | 4153 | 0.468328 |
| 388557 | PC\_00NYII | PC\_074C3D | PC\_00NYII | PC\_074C3D | 33 | 38 | 99.58 | 92.73 | 1254 | 3.098298 | 1.967220 | 388 | 0.503047 |

4816938 rows × 13 columns

In [24]:

```
b10.sample(n=10000).plot("log_lqh", "norm_score", kind="scatter")
```

Out[24]:

```
<matplotlib.axes._subplots.AxesSubplot at 0x7f3c98c1b880>
```

So we've corrected for the length bias, but we still have increasing variance with length.
It's still better than we had before.

In [25]:

```
b10.sample(n=10000).plot("norm_score", "probability", kind="scatter")
```

Out[25]:

```
<matplotlib.axes._subplots.AxesSubplot at 0x7f3c98b389d0>
```

The normalised score doesn't fit the probability quite as well as the un-normalised score.

In [26]:

```
b10.sample(n=10000).plot("log_score", "probability", kind="scatter")
```

Out[26]:

```
<matplotlib.axes._subplots.AxesSubplot at 0x7f3c98ba79d0>
```

It's a bit hard to say what is better to be honest.
We really want more separation of the low scores, but since we have so many alignments with very high probabilities we also need a way of prioritising them.

In [27]:

```
bself.sample(n=10000)["norm_score"].plot.kde()
```

Out[27]:

```
<matplotlib.axes._subplots.AxesSubplot at 0x7f3c98a33130>
```

In [28]:

```
b10.sample(n=10000)["norm_score"].plot.kde()
```

Out[28]:

```
<matplotlib.axes._subplots.AxesSubplot at 0x7f3c989e3340>
```

In [29]:

```
(bself["norm_score"] * bself["probability"]).sample(n=10000).plot.kde()
```

Out[29]:

```
<matplotlib.axes._subplots.AxesSubplot at 0x7f3c98aecc10>
```

In [30]:

```
(bself["norm_score"] * bself["probability"]).sample(n=10000).plot.kde()
```

Out[30]:

```
<matplotlib.axes._subplots.AxesSubplot at 0x7f3c988e8d60>
```

## Finding paired scores¶

We need to find a way of combining two scores for pairs of reciprocal matches.
The obvious one is to use the mean (i.e the midpoint between the two), but the product might work as well.

In [31]:

```
bself["max_id"].nunique() == len(bself["max_id"])
```

Out[31]:

```
True
```

In [32]:

```
pairs = b10.sort_values(["min_id", "max_id"])
pairs["first"] = pairs["query_id"] == pairs["min_id"]
```

In [33]:

```
pairs = (
    pairs
    [["min_id", "max_id", "first", "query_length", "lqh", "log_lqh", "score", "norm_score", "probability"]]
    .pivot_table(index=["min_id", "max_id"], columns="first")
)

pairs.reset_index(inplace=True)
pairs
```

Out[33]:

|  | min\_id | max\_id | log\_lqh | | lqh | | norm\_score | | probability | | query\_length | | score | |
| --- | --- | --- | --- | --- | --- | --- | --- | --- | --- | --- | --- | --- | --- | --- |
| first |  |  | False | True | False | True | False | True | False | True | False | True | False | True |
| 0 | PC\_000008 | PC\_004KTP | 5.763306 | 5.763306 | 579837 | 579837 | 0.073867 | 0.078112 | 99.68 | 99.75 | 1041 | 557 | 207.44 | 219.36 |
| 1 | PC\_000008 | PC\_01B7M5 | 5.436051 | 5.436051 | 272930 | 272930 | 0.092304 | 0.104503 | 99.61 | 99.77 | 490 | 557 | 185.53 | 210.05 |
| 2 | PC\_000008 | PC\_036WQD | 5.840326 | 5.840326 | 692351 | 692351 | 0.068552 | 0.069674 | 99.67 | 99.70 | 1243 | 557 | 208.28 | 211.69 |
| 3 | PC\_000008 | PC\_04OBCT | 5.521830 | 5.521830 | 332529 | 332529 | 0.093649 | 0.100567 | 99.72 | 99.80 | 597 | 557 | 205.48 | 220.66 |
| 4 | PC\_000008 | PC\_064DGH | 5.819207 | 5.819207 | 659488 | 659488 | 0.069466 | 0.073216 | 99.66 | 99.73 | 1184 | 557 | 206.55 | 217.70 |
| ... | ... | ... | ... | ... | ... | ... | ... | ... | ... | ... | ... | ... | ... | ... |
| 2408464 | PC\_08KVJS | PC\_08KWTP | 6.042667 | 6.042667 | 1103232 | 1103232 | 0.384053 | 0.391165 | 100.00 | 100.00 | 1632 | 676 | 1434.91 | 1461.48 |
| 2408465 | PC\_08KY6J | PC\_08L2R6 | 5.473272 | 5.473272 | 297353 | 297353 | 0.469112 | 0.467886 | 100.00 | 100.00 | 749 | 397 | 979.47 | 976.91 |
| 2408466 | PC\_08KZ7E | PC\_08L2L8 | 4.063033 | 4.063033 | 11562 | 11562 | 0.646535 | 0.641718 | 100.00 | 100.00 | 123 | 94 | 319.44 | 317.06 |
| 2408467 | PC\_08L0U1 | PC\_08L2M7 | 4.240724 | 4.240724 | 17407 | 17407 | 0.488112 | 0.487302 | 100.00 | 100.00 | 103 | 169 | 289.19 | 288.71 |
| 2408468 | PC\_08L25F | PC\_08L25H | 5.111246 | 5.111246 | 129195 | 129195 | 0.279013 | 0.278084 | 100.00 | 100.00 | 165 | 783 | 402.40 | 401.06 |

2408469 rows × 14 columns

In [34]:

```
pairs.columns = ["min_id", "max_id", "log_lqh", "log_lqh_2", "lqh", "lqh_2", "lnorm_score", "rnorm_score", "lprob", "rprob", "llen", "rlen", "lscore", "rscore"]
pairs.drop(columns=["log_lqh_2", "lqh_2"], inplace=True)

pairs = pd.merge(
    pd.merge(
        pairs,
        bself[["min_id", "norm_score"]].rename(columns={"norm_score": "min_id_norm_score"}),
        on="min_id",
        how="left",
    ),
    bself[["max_id", "norm_score"]].rename(columns={"norm_score": "max_id_norm_score"}),
    on="max_id",
    how="left",
)

pairs.head()
```

Out[34]:

|  | min\_id | max\_id | log\_lqh | lqh | lnorm\_score | rnorm\_score | lprob | rprob | llen | rlen | lscore | rscore | min\_id\_norm\_score | max\_id\_norm\_score |
| --- | --- | --- | --- | --- | --- | --- | --- | --- | --- | --- | --- | --- | --- | --- |
| 0 | PC\_000008 | PC\_004KTP | 5.763306 | 579837 | 0.073867 | 0.078112 | 99.68 | 99.75 | 1041 | 557 | 207.44 | 219.36 | 0.998661 | 0.922086 |
| 1 | PC\_000008 | PC\_01B7M5 | 5.436051 | 272930 | 0.092304 | 0.104503 | 99.61 | 99.77 | 490 | 557 | 185.53 | 210.05 | 0.998661 | 0.654780 |
| 2 | PC\_000008 | PC\_036WQD | 5.840326 | 692351 | 0.068552 | 0.069674 | 99.67 | 99.70 | 1243 | 557 | 208.28 | 211.69 | 0.998661 | 0.990437 |
| 3 | PC\_000008 | PC\_04OBCT | 5.521830 | 332529 | 0.093649 | 0.100567 | 99.72 | 99.80 | 597 | 557 | 205.48 | 220.66 | 0.998661 | 0.697262 |
| 4 | PC\_000008 | PC\_064DGH | 5.819207 | 659488 | 0.069466 | 0.073216 | 99.66 | 99.73 | 1184 | 557 | 206.55 | 217.70 | 0.998661 | 0.889741 |

In [35]:

```
pairs["llen_norm"] = pairs["lscore"] / pairs["llen"]
pairs["rlen_norm"] = pairs["rscore"] / pairs["rlen"]
pairs["llen_normnorm"] = pairs["lnorm_score"] / pairs["llen"]
pairs["rlen_normnorm"] = pairs["rnorm_score"] / pairs["rlen"]
pairs["mid_norm"] = (pairs["lnorm_score"] + pairs["rnorm_score"]) / 2
pairs["mid"] = (pairs["lscore"] + pairs["rscore"]) / 2
pairs["mid_prob"] = (pairs["lprob"] + pairs["rprob"]) / 2
pairs["prod_norm"] = pairs["lnorm_score"] * pairs["rnorm_score"]
pairs["prod"] = pairs["lscore"] * pairs["rscore"]
pairs["prod_prob"] = pairs["lprob"] * pairs["rprob"]

pairs["ln_prob"] = (pairs["lprob"] / 100.) * pairs["lnorm_score"]
pairs["rn_prob"] = (pairs["rprob"] / 100.) * pairs["rnorm_score"]
pairs["mid_rn_prob"] = (pairs["ln_prob"] + pairs["rn_prob"]) / 2
pairs["prod_rn_prob"] = pairs["ln_prob"] * pairs["rn_prob"]
pairs
```

Out[35]:

|  | min\_id | max\_id | log\_lqh | lqh | lnorm\_score | rnorm\_score | lprob | rprob | llen | rlen | ... | mid\_norm | mid | mid\_prob | prod\_norm | prod | prod\_prob | ln\_prob | rn\_prob | mid\_rn\_prob | prod\_rn\_prob |
| --- | --- | --- | --- | --- | --- | --- | --- | --- | --- | --- | --- | --- | --- | --- | --- | --- | --- | --- | --- | --- | --- |
| 0 | PC\_000008 | PC\_004KTP | 5.763306 | 579837 | 0.073867 | 0.078112 | 99.68 | 99.75 | 1041 | 557 | ... | 0.075989 | 213.400 | 99.715 | 0.005770 | 4.550404e+04 | 9943.0800 | 0.073631 | 0.077916 | 0.075773 | 0.005737 |
| 1 | PC\_000008 | PC\_01B7M5 | 5.436051 | 272930 | 0.092304 | 0.104503 | 99.61 | 99.77 | 490 | 557 | ... | 0.098403 | 197.790 | 99.690 | 0.009646 | 3.897058e+04 | 9938.0897 | 0.091944 | 0.104263 | 0.098103 | 0.009586 |
| 2 | PC\_000008 | PC\_036WQD | 5.840326 | 692351 | 0.068552 | 0.069674 | 99.67 | 99.70 | 1243 | 557 | ... | 0.069113 | 209.985 | 99.685 | 0.004776 | 4.409079e+04 | 9937.0990 | 0.068326 | 0.069465 | 0.068896 | 0.004746 |
| 3 | PC\_000008 | PC\_04OBCT | 5.521830 | 332529 | 0.093649 | 0.100567 | 99.72 | 99.80 | 597 | 557 | ... | 0.097108 | 213.070 | 99.760 | 0.009418 | 4.534122e+04 | 9952.0560 | 0.093387 | 0.100366 | 0.096877 | 0.009373 |
| 4 | PC\_000008 | PC\_064DGH | 5.819207 | 659488 | 0.069466 | 0.073216 | 99.66 | 99.73 | 1184 | 557 | ... | 0.071341 | 212.125 | 99.695 | 0.005086 | 4.496593e+04 | 9939.0918 | 0.069230 | 0.073018 | 0.071124 | 0.005055 |
| ... | ... | ... | ... | ... | ... | ... | ... | ... | ... | ... | ... | ... | ... | ... | ... | ... | ... | ... | ... | ... | ... |
| 2408464 | PC\_08KVJS | PC\_08KWTP | 6.042667 | 1103232 | 0.384053 | 0.391165 | 100.00 | 100.00 | 1632 | 676 | ... | 0.387609 | 1448.195 | 100.000 | 0.150228 | 2.097092e+06 | 10000.0000 | 0.384053 | 0.391165 | 0.387609 | 0.150228 |
| 2408465 | PC\_08KY6J | PC\_08L2R6 | 5.473272 | 297353 | 0.469112 | 0.467886 | 100.00 | 100.00 | 749 | 397 | ... | 0.468499 | 978.190 | 100.000 | 0.219491 | 9.568540e+05 | 10000.0000 | 0.469112 | 0.467886 | 0.468499 | 0.219491 |
| 2408466 | PC\_08KZ7E | PC\_08L2L8 | 4.063033 | 11562 | 0.646535 | 0.641718 | 100.00 | 100.00 | 123 | 94 | ... | 0.644126 | 318.250 | 100.000 | 0.414893 | 1.012816e+05 | 10000.0000 | 0.646535 | 0.641718 | 0.644126 | 0.414893 |
| 2408467 | PC\_08L0U1 | PC\_08L2M7 | 4.240724 | 17407 | 0.488112 | 0.487302 | 100.00 | 100.00 | 103 | 169 | ... | 0.487707 | 288.950 | 100.000 | 0.237858 | 8.349204e+04 | 10000.0000 | 0.488112 | 0.487302 | 0.487707 | 0.237858 |
| 2408468 | PC\_08L25F | PC\_08L25H | 5.111246 | 129195 | 0.279013 | 0.278084 | 100.00 | 100.00 | 165 | 783 | ... | 0.278548 | 401.730 | 100.000 | 0.077589 | 1.613865e+05 | 10000.0000 | 0.279013 | 0.278084 | 0.278548 | 0.077589 |

2408469 rows × 28 columns

Cool so we have the paired scores together, how well corellated are they?

In [36]:

```
pairs.sample(n=10000).plot("lprob", "rprob", kind="scatter")
```

Out[36]:

```
<matplotlib.axes._subplots.AxesSubplot at 0x7f3c98b98610>
```

Probability looks pretty symmetrical.

In [37]:

```
pairs.sample(n=10000).plot("lscore", "rscore", kind="scatter")
```

Out[37]:

```
<matplotlib.axes._subplots.AxesSubplot at 0x7f3c988a80d0>
```

The raw scores are very closely corellated, which is promising.

In [38]:

```
pairs.sample(n=10000).plot("lnorm_score", "rnorm_score", kind="scatter")
```

Out[38]:

```
<matplotlib.axes._subplots.AxesSubplot at 0x7f3c98875790>
```

Normalised scores are also pretty good.

In [39]:

```
pairs.sample(n=10000).plot("ln_prob", "rn_prob", kind="scatter")
```

Out[39]:

```
<matplotlib.axes._subplots.AxesSubplot at 0x7f3c98843be0>
```

Unsurprisingly the product of normalised score and probability is also fine.
But how about simpler methods?
Could we just divide the score by the length?

In [40]:

```
pairs.sample(n=10000).plot("llen_norm", "rlen_norm", kind="scatter")
```

Out[40]:

```
<matplotlib.axes._subplots.AxesSubplot at 0x7f3c98813be0>
```

In [41]:

```
pairs.sample(n=10000).plot("llen_normnorm", "rlen_normnorm", kind="scatter")
```

Out[41]:

```
<matplotlib.axes._subplots.AxesSubplot at 0x7f3c987d4430>
```

Oof, no corellation, so taking a mid-point of the two scores would be super unreliable.

## Checking normalised scores vs length again¶

In [42]:

```
# Un normalised mid-points
pairs.sample(n=10000).plot("log_lqh", "mid", kind="scatter")
```

Out[42]:

```
<matplotlib.axes._subplots.AxesSubplot at 0x7f3c98739130>
```

In [43]:

```
# normalised mid-points
pairs.sample(n=10000).plot("log_lqh", "mid_norm", kind="scatter")
```

Out[43]:

```
<matplotlib.axes._subplots.AxesSubplot at 0x7f3c9871bc70>
```

In [44]:

```
pairs.sample(n=10000).plot("log_lqh", "mid_prob", kind="scatter")
```

Out[44]:

```
<matplotlib.axes._subplots.AxesSubplot at 0x7f3c9866abe0>
```

In [45]:

```
# Un normalised product
pairs.sample(n=10000).plot("log_lqh", "prod", kind="scatter")
```

Out[45]:

```
<matplotlib.axes._subplots.AxesSubplot at 0x7f3c9869d490>
```

In [46]:

```
# normalised product
pairs.sample(n=10000).plot("log_lqh", "prod_norm", kind="scatter")
```

Out[46]:

```
<matplotlib.axes._subplots.AxesSubplot at 0x7f3c986029a0>
```

In [47]:

```
pairs.sample(n=10000).plot("log_lqh", "prod_prob", kind="scatter")
```

Out[47]:

```
<matplotlib.axes._subplots.AxesSubplot at 0x7f3c985727f0>
```

In [48]:

```
pairs.sample(n=10000).plot("log_lqh", "mid_rn_prob", kind="scatter")
```

Out[48]:

```
<matplotlib.axes._subplots.AxesSubplot at 0x7f3c9855aee0>
```

In [49]:

```
pairs.sample(n=10000).plot("log_lqh", "prod_rn_prob", kind="scatter")
```

Out[49]:

```
<matplotlib.axes._subplots.AxesSubplot at 0x7f3c98527af0>
```

In [50]:

```
pairs.sample(n=10000).plot("mid", "rlen_norm", kind="scatter")
```

Out[50]:

```
<matplotlib.axes._subplots.AxesSubplot at 0x7f3c9847dfd0>
```

In [51]:

```
pairs.sample(n=10000).plot("mid_prob", "mid_norm", kind="scatter")
```

Out[51]:

```
<matplotlib.axes._subplots.AxesSubplot at 0x7f3c984544f0>
```

In [52]:

```
pairs.sample(n=10000).plot("mid_rn_prob", "mid_prob", kind="scatter")
```

Out[52]:

```
<matplotlib.axes._subplots.AxesSubplot at 0x7f3c98459cd0>
```

In [53]:

```
pairs.sample(n=10000).plot("log_lqh", "mid_rn_prob", kind="scatter")
```

Out[53]:

```
<matplotlib.axes._subplots.AxesSubplot at 0x7f3c987311f0>
```

In [54]:

```
pairs.sample(n=10000)["mid"].plot.kde()
```

Out[54]:

```
<matplotlib.axes._subplots.AxesSubplot at 0x7f3c9841cd30>
```

In [55]:

```
pairs.sample(n=10000)["mid_norm"].plot.kde()
```

Out[55]:

```
<matplotlib.axes._subplots.AxesSubplot at 0x7f3c982fabe0>
```

In [56]:

```
pairs.sample(n=10000)["mid_prob"].plot.kde()
```

Out[56]:

```
<matplotlib.axes._subplots.AxesSubplot at 0x7f3c982d3a00>
```

In [57]:

```
pairs.sample(n=10000)["mid_rn_prob"].plot.kde()
```

Out[57]:

```
<matplotlib.axes._subplots.AxesSubplot at 0x7f3c982357f0>
```

In [58]:

```
pairs.sample(n=10000)["prod"].plot.kde()
```

Out[58]:

```
<matplotlib.axes._subplots.AxesSubplot at 0x7f3c98206160>
```

In [59]:

```
pairs.sample(n=10000)["prod_norm"].plot.kde()
```

Out[59]:

```
<matplotlib.axes._subplots.AxesSubplot at 0x7f3c981dd2e0>
```

In [60]:

```
pairs.sample(n=10000)["prod_prob"].plot.kde()
```

Out[60]:

```
<matplotlib.axes._subplots.AxesSubplot at 0x7f3c9813d1c0>
```

In [61]:

```
pairs.sample(n=10000)["prod_rn_prob"].plot.kde()
```

Out[61]:

```
<matplotlib.axes._subplots.AxesSubplot at 0x7f3c981216d0>
```

In [62]:

```
pairs["mid_norm"].max()
```

Out[62]:

```
1.084946448298163
```

In [63]:

```
pairs["mid_norm"].min()
```

Out[63]:

```
0.022130032630388832
```

In [64]:

```
pairs["mid_norm"].sample(10000).plot.kde()
```

Out[64]:

```
<matplotlib.axes._subplots.AxesSubplot at 0x7f3c981392b0>
```

In [65]:

```
(pairs["mid_norm"] / pairs["mid_norm"].max()).sample(10000).plot.kde()
```

Out[65]:

```
<matplotlib.axes._subplots.AxesSubplot at 0x7f3c98033c40>
```

In [66]:

```
pairs_self = pairs[pairs["min_id"].notnull() & pairs["max_id"].notnull()].copy()

# Ok this name is ridiculous but it's the mid norm score / mid norm score of self matches. 
pairs_self["norm_mid_norm"] = pairs_self["mid_norm"] / ((pairs_self["min_id_norm_score"] + pairs_self["min_id_norm_score"]) / 2)
pairs_self.head()
```

Out[66]:

|  | min\_id | max\_id | log\_lqh | lqh | lnorm\_score | rnorm\_score | lprob | rprob | llen | rlen | ... | mid | mid\_prob | prod\_norm | prod | prod\_prob | ln\_prob | rn\_prob | mid\_rn\_prob | prod\_rn\_prob | norm\_mid\_norm |
| --- | --- | --- | --- | --- | --- | --- | --- | --- | --- | --- | --- | --- | --- | --- | --- | --- | --- | --- | --- | --- | --- |
| 0 | PC\_000008 | PC\_004KTP | 5.763306 | 579837 | 0.073867 | 0.078112 | 99.68 | 99.75 | 1041 | 557 | ... | 213.400 | 99.715 | 0.005770 | 45504.0384 | 9943.0800 | 0.073631 | 0.077916 | 0.075773 | 0.005737 | 0.076091 |
| 1 | PC\_000008 | PC\_01B7M5 | 5.436051 | 272930 | 0.092304 | 0.104503 | 99.61 | 99.77 | 490 | 557 | ... | 197.790 | 99.690 | 0.009646 | 38970.5765 | 9938.0897 | 0.091944 | 0.104263 | 0.098103 | 0.009586 | 0.098535 |
| 2 | PC\_000008 | PC\_036WQD | 5.840326 | 692351 | 0.068552 | 0.069674 | 99.67 | 99.70 | 1243 | 557 | ... | 209.985 | 99.685 | 0.004776 | 44090.7932 | 9937.0990 | 0.068326 | 0.069465 | 0.068896 | 0.004746 | 0.069206 |
| 3 | PC\_000008 | PC\_04OBCT | 5.521830 | 332529 | 0.093649 | 0.100567 | 99.72 | 99.80 | 597 | 557 | ... | 213.070 | 99.760 | 0.009418 | 45341.2168 | 9952.0560 | 0.093387 | 0.100366 | 0.096877 | 0.009373 | 0.097239 |
| 4 | PC\_000008 | PC\_064DGH | 5.819207 | 659488 | 0.069466 | 0.073216 | 99.66 | 99.73 | 1184 | 557 | ... | 212.125 | 99.695 | 0.005086 | 44965.9350 | 9939.0918 | 0.069230 | 0.073018 | 0.071124 | 0.005055 | 0.071437 |

5 rows × 29 columns

In [67]:

```
pairs_self["norm_mid_norm"].sample(10000).plot.kde()
```

Out[67]:

```
<matplotlib.axes._subplots.AxesSubplot at 0x7f3c980197f0>
```

This normalises things well, but isn't too different to dividing by the global max norm score.
Since not all clusters had a self match, we can't use them I guess.
We could actually just align them all without running it as a search command with hhsuite.
Possibly for a later date.

Let's just check what the other relationships look like for this normalised version.

In [68]:

```
# normalised mid-points
pairs_self.sample(n=10000).plot("log_lqh", "norm_mid_norm", kind="scatter")
```

Out[68]:

```
<matplotlib.axes._subplots.AxesSubplot at 0x7f3c980cf880>
```

It's a lovely big ball.

In [69]:

```
# normalised mid-points
pairs.sample(n=10000).plot("log_lqh", "mid_norm", kind="scatter")
```

Out[69]:

```
<matplotlib.axes._subplots.AxesSubplot at 0x7f3c98df0880>
```

Comparing the extra normalised score, we have a slightly more uniform distribution, and a spread that is closer to 1.

What does it look like if we divide by the max mid norm?

In [70]:

```
pairs["div_max_mid_norm"] = (pairs["mid_norm"] / pairs["mid_norm"].max())

# normalised mid-points
pairs.sample(n=10000).plot("log_lqh", "div_max_mid_norm", kind="scatter")
```

Out[70]:

```
<matplotlib.axes._subplots.AxesSubplot at 0x7f3c97f056d0>
```

It hasn't really changed the distribution, but at least we're guaranteed a value between 0 and 1.

Can we manually scale this value?

In [71]:

```
pairs["sqrt_midnorm"] = np.sqrt(pairs["mid_norm"])

# normalised mid-points
pairs.sample(n=10000).plot("log_lqh", "sqrt_midnorm", kind="scatter")
```

Out[71]:

```
<matplotlib.axes._subplots.AxesSubplot at 0x7f3c97ee7520>
```

In [72]:

```
m = pairs["mid_norm"].max()
pairs["fancy_max_mid_norm"] = 2 * (pairs["div_max_mid_norm"] * m) / (pairs["div_max_mid_norm"] + m)

pairs.sample(n=10000).plot("log_lqh", "fancy_max_mid_norm", kind="scatter")
```

Out[72]:

```
<matplotlib.axes._subplots.AxesSubplot at 0x7f3c97e3fc10>
```

In [73]:

```
pairs["sqrt_midnorm"].sample(10000).plot.kde()
```

Out[73]:

```
<matplotlib.axes._subplots.AxesSubplot at 0x7f3c97e157f0>
```

In [74]:

```
pairs["div_max_mid_norm"].sample(10000).plot.kde()
```

Out[74]:

```
<matplotlib.axes._subplots.AxesSubplot at 0x7f3c97dc9580>
```

In [75]:

```
pairs["fancy_max_mid_norm"].sample(10000).plot.kde()
```

Out[75]:

```
<matplotlib.axes._subplots.AxesSubplot at 0x7f3c97da0280>
```

In [76]:

```
pairs.sample(n=10000).plot("mid_prob", "sqrt_midnorm", kind="scatter")
```

Out[76]:

```
<matplotlib.axes._subplots.AxesSubplot at 0x7f3c97d13b80>
```

To my eyes the "fancy" method looks good.
It kind of normalises to have a mode around 0.5 and is quite symmetrical.

In [77]:

```
from scipy.stats import beta
```

In [78]:

```
pairs["mid_beta_cdf"] = beta(a=1, b=3).cdf(pairs["mid_norm"])
```

In [79]:

```
pairs.sample(n=10000).plot("log_lqh", "mid_beta_cdf", kind="scatter")
```

Out[79]:

```
<matplotlib.axes._subplots.AxesSubplot at 0x7f3c9822f850>
```

In [80]:

```
pairs.sample(n=10000).plot("mid_norm", "mid_beta_cdf", kind="scatter")
```

Out[80]:

```
<matplotlib.axes._subplots.AxesSubplot at 0x7f3c98b5a340>
```

In [81]:

```
pairs["mid_beta_cdf"].sample(n=10000).plot.kde()
```

Out[81]:

```
<matplotlib.axes._subplots.AxesSubplot at 0x7f3c97c55d90>
```

I think I will transform the data to a Beta 1 3 distribution.
Because it's a monotonic function, I can transform the data in awk, select the top matches and then run the beta
transformation in python.

## Making some plots.¶

In [82]:

```
sns.set_palette("deep")
sns.set_context("paper")
sns.set_style("ticks", rc={"edgecolor": "none", 'patch.force_edgecolor': False})
```

In [83]:

```
fig, ax = plt.subplots(figsize=(3, 3))

sns.scatterplot("lqh", "score", data=b10.sample(n=10000), ax=ax, edgecolor=None, s=3)
ax.set_yscale("log")
ax.set_xscale("log")

ax.set_xlabel(r"score")
ax.set_xlabel(r"query $\times$ target HMM lengths")
fig.savefig("06a-score_vs_lqh.pdf")
fig.savefig("06a-score_vs_lqh.png")
```

In [84]:

```
fig, ax = plt.subplots(figsize=(3, 3))

sns.scatterplot("lqh", "score", data=bself.sample(n=10000), ax=ax, edgecolor=None, s=3)
ax.set_yscale("log")
ax.set_xscale("log")

ax.set_xlabel(r"score")
ax.set_xlabel(r"query $\times$ target HMM lengths")
fig.savefig("06a-self_score_vs_lqh.pdf")
fig.savefig("06a-self_score_vs_lqh.png")
```

In [85]:

```
fig, ax = plt.subplots(figsize=(3, 3))

sns.scatterplot("lqh", "norm_score", data=b10.sample(n=10000), ax=ax, edgecolor=None, s=3)
ax.set_xscale("log")

ax.set_ylabel(r"normalised score")
ax.set_xlabel(r"query $\times$ target HMM lengths")
fig.savefig("06a-normscore_vs_lqh.pdf")
fig.savefig("06a-normscore_vs_lqh.png")
```

In [86]:

```
fig, ax = plt.subplots(figsize=(3, 3))

sns.scatterplot("lnorm_score", "rnorm_score", data=pairs.sample(n=10000), ax=ax, edgecolor=None, s=3)
ax.set_ylabel("target normalised score")
ax.set_xlabel("query normalised score")
fig.savefig("06a-lnormscore_vs_rnormscore.pdf")
fig.savefig("06a-lnormscore_vs_rnormscore.png")
```

In [87]:

```
ax = sns.jointplot("lqh", "mid_norm", kind="scatter", data=pairs.sample(n=10000), s=3)
ax.ax_marg_x.set_xscale("log")
ax.ax_joint.set_xscale("log")
ax.fig.set_size_inches(4, 4)

ax.ax_joint.set_ylabel("mean normalised score")
ax.ax_joint.set_xlabel(r"query $\times$ target HMM lengths")
fig.savefig("06a-midnormscore_vs_lqh.pdf")
fig.savefig("06a-midnormscore_vs_lqh.png")
```

In [88]:

```
ax = sns.jointplot("lqh", "mid_beta_cdf", kind="scatter", data=pairs.sample(n=10000), s=3)
ax.ax_marg_x.set_xscale("log")
ax.ax_joint.set_xscale("log")
ax.fig.set_size_inches(4, 4)

ax.ax_joint.set_ylabel(r"$Beta(1, 3)$ CDF(mean normalised score)")
ax.ax_joint.set_xlabel(r"query $\times$ target HMM lengths")
fig.savefig("06a-mid_beta_cdf_vs_lqh.pdf")
fig.savefig("06a-mid_beta_cdf_vs_lqh.png")
```

In [89]:

```
fig, ax = plt.subplots(figsize=(3, 3))

sns.scatterplot("mid_norm", "mid_beta_cdf", data=pairs.sample(n=10000), ax=ax, edgecolor=None, s=3)

ax.set_xlabel("mean normalised score")
ax.set_ylabel(r"$Beta(1, 3)$ CDF(mean normalised score)")
fig.savefig("06a-mid_beta_cdf_vs_midnormscore.pdf")
fig.savefig("06a-mid_beta_cdf_vs_midnormscore.png")
```

In [ ]:

```

```
