# Supplementary material for: Remote homology clustering identifies lowly conserved families of effector proteins in plant-pathogenic fungi
Source: Microb Genom. 2021 Sep 1;7(9):000637. doi: 10.1099/mgen.0.000637 (PMC8715435; doi:10.1099/mgen.0.000637)
Supplement: Supplementary material 12 [file mgen-7-0637-s0012.zip › supplementary_data_06-remeff_scripts/11-align_hmms.html]

11-align\_hmms


# In this notebook we want to plot the interesting subgraphs and align the HMMs.¶

In [1]:

```
import os
from os.path import join as pjoin
import graph_tool.all as gt

import pandas as pd

import matplotlib
import matplotlib.pyplot as plt
from matplotlib import cm

import seaborn as sns
from matplotlib.colors import ListedColormap

import ffdb
from ffdb.ffindex import FFDB

import subprocess
from tempfile import NamedTemporaryFile

import logomaker as lm

from io import StringIO, BytesIO
from Bio import AlignIO
from Bio import SeqIO
```

```
/home/ubuntu/.miniconda/envs/remote_homology/lib/python3.7/site-packages/graph_tool/draw/cairo_draw.py:1494: RuntimeWarning: Error importing Gtk module: No module named 'gi'; GTK+ drawing will not work.
  warnings.warn(msg, RuntimeWarning)
```

In [2]:

```
OUTDIR="11-align_hmms"
PALETTE="hls"
LOGO_PALETTE="skylign_protein"
```

In [3]:

```
os.makedirs(OUTDIR)
```

```
---------------------------------------------------------------------------
FileExistsError                           Traceback (most recent call last)
<ipython-input-3-d725c6a3d27b> in <module>
----> 1 os.makedirs(OUTDIR)

~/.miniconda/envs/remote_homology/lib/python3.7/os.py in makedirs(name, mode, exist_ok)
    221             return
    222     try:
--> 223         mkdir(name, mode)
    224     except OSError:
    225         # Cannot rely on checking for EEXIST, since the operating system

FileExistsError: [Errno 17] File exists: '11-align_hmms'
```

First I need to convert the original (non-enriched) MSAs to A3M format, and remove the consensus sequences.

We'll use these later.

```
bin/add_fasta_id_as_ffindex_id.py \
  -i 11-msas_renamed_index.ffindex \
  --strip-consensus \
  data/msas/db_fasta.ff{data,index}
```

```
ffindex_apply \
  data/msas/db_fasta.ffdata \
  11-msas_renamed_index.ffindex \
  -d 11-msas_a3m.ffdata \
  -i 11-msas_a3m.ffindex \
  -- \
  bin/run_fas_to_a3m.sh
```

## Load and prepare the graph.¶

We need to add some properties to the graph before we start taking subgraphs.

In [5]:

```
G = gt.load_graph("08-full_length_clustering.gt")
```

In [6]:

```
eff = pd.read_csv("10-cluster_summarisation-cluster_taxon.tsv", sep="\t", na_values=".", low_memory=False)
eff
```

Out[6]:

|  | supercluster\_component | supercluster\_markov | supercluster\_greedy | cluster | effectors | phibase | organisms | lca\_rank | lca | cluster\_size | cluster\_length |
| --- | --- | --- | --- | --- | --- | --- | --- | --- | --- | --- | --- |
| 0 | PC\_0000B2 | PC\_0000B2 | PC\_0000B2 | PC\_0000B2 | NaN | NaN | Pseudogymnoascus sp. VKM F-4519 (FW-2642) | species | Pseudogymnoascus sp. VKM F-4519 (FW-2642) | 1 | 1103 |
| 1 | PC\_0000B2 | PC\_0000B2 | PC\_0000B2 | PC\_07SB5M | NaN | NaN | Epichloe bromicola;Epichloe uncinata | genus | Epichloe | 2 | 1128 |
| 2 | PC\_0000RV | PC\_0000RV | PC\_0000RV | PC\_0000RV | NaN | NaN | Phialophora attinorum | species | Phialophora attinorum | 1 | 237 |
| 3 | PC\_0000RV | PC\_0000RV | PC\_0000RV | PC\_057HPB | NaN | NaN | Colletotrichum orbiculare MAFF 240422 | strain | Colletotrichum orbiculare MAFF 240422 | 1 | 281 |
| 4 | PC\_0001BP | PC\_0001BP | PC\_0001BP | PC\_0001BP | NaN | NaN | Pseudogymnoascus sp. VKM F-4246 | species | Pseudogymnoascus sp. VKM F-4246 | 1 | 750 |
| ... | ... | ... | ... | ... | ... | ... | ... | ... | ... | ... | ... |
| 286507 | NaN | NaN | NaN | PC\_08L65P | NaN | Q5AJ90#PHI:3688 | Candida albicans P60002;Candida albicans P7804... | species | Candida albicans | 1 | 412 |
| 286508 | NaN | NaN | NaN | PC\_08L68V | NaN | NaN | Alternaria mali | species | Alternaria mali | 1 | 311 |
| 286509 | NaN | NaN | NaN | PC\_08L6K4 | NaN | NaN | Alternaria mali | species | Alternaria mali | 1 | 956 |
| 286510 | NaN | NaN | NaN | PC\_08L6Q0 | NaN | NaN | Alternaria destruens;Alternaria dauci;Alternar... | genus | Alternaria | 3 | 204 |
| 286511 | NaN | NaN | NaN | PC\_08L6ZT | NaN | NaN | Serendipita vermifera MAFF 305830 | strain | Serendipita vermifera MAFF 305830 | 1 | 1777 |

286512 rows × 11 columns

Add the Effector names to the nodes.

In [8]:

```
eff_map = eff[eff["effectors"].notnull()].set_index("cluster")["effectors"].to_dict()
effector_vp = G.new_vertex_property("string")
for v in G.vertices():
    cl = G.vp["cluster"][v]
    if cl in eff_map:
        effector_vp[v] = eff_map[cl]
        
G.vp["effectors"] = effector_vp
```

Add the cluster MSA/HMM lengths to the graph.

In [9]:

```
len_map = eff.set_index("cluster")["cluster_length"].to_dict()
len_vp = G.new_vertex_property("int")
for v in G.vertices():
    cl = G.vp["cluster"][v]
    if cl in len_map:
        len_vp[v] = len_map[cl]
        
G.vp["length"] = len_vp
```

In [10]:

```
size_map = eff.set_index("cluster")["cluster_size"].to_dict()
size_vp = G.new_vertex_property("int")
for v in G.vertices():
    cl = G.vp["cluster"][v]
    if cl in size_map:
        size_vp[v] = size_map[cl]
        
G.vp["size"] = size_vp
```

Add the inverted edge weights to the graph.
We need this for later, so that we can get the maximum spanning tree of the weights.

In [11]:

```
neg_ep = G.new_edge_property("float")
for e in G.edges():
    w = G.ep["weight"][e]
    neg_ep[e] = 1 - w

G.ep["neg"] = neg_ep
```

And here is a handy tool to collect a subgraph given the nodes.

In [12]:

```
def get_subgraph(G, nodes):
    mask = G.new_vertex_property("bool")
    for v in G.vertices():
        cl = G.vp["cluster"][v]
        mask[v] = cl in nodes
        
    return gt.GraphView(G, vfilt=mask)
```

In [ ]:

### Plotting AvrLm6¶

As an example, we'll plot the AvrLm6 component because it was one of the more interesting ones.

In [13]:

```
lm6_clusters = set(
    eff
    .loc[
        eff["supercluster_component"] == eff.loc[
            eff["effectors"] == "LmAvrLm6",
            "supercluster_component"
        ].iloc[0],
        "cluster"
    ]
)
```

In [ ]:

In [14]:

```
subG = get_subgraph(G, lm6_clusters)

pos = gt.sfdp_layout(subG, eweight=subG.edge_properties["weight"])
esize = gt.prop_to_size(subG.edge_properties["weight"], mi=0.5, ma=3)
vsize = gt.prop_to_size(subG.vp["size"], mi=10, ma=20, log=True)

vcolour = subG.new_vertex_property("int")
mapping = {k: v for v, k in enumerate(set(subG.vertex_properties["supercluster_markov"]))}

cmap = ListedColormap(sns.color_palette(PALETTE, len(mapping)), N=len(mapping))

for v in subG.vertices():
    vcolour[v] = mapping[subG.vertex_properties["supercluster_markov"][v]]
```

In [15]:

```
gt.graph_draw(
    subG,
    pos=pos,
    edge_pen_width=esize,
    inline=True,
    vcmap=cmap,
    vertex_size=vsize,
    vertex_fill_color=vcolour,
    vertex_text=subG.vertex_properties["effectors"],
    vertex_text_position=0,
    vertex_text_offset=[-1, 1],
    vertex_pen_width=0.0,
)
```

Out[15]:

```
<VertexPropertyMap object with value type 'vector<double>', for Graph 0x7f74e96645d0, at 0x7f74e9673710>
```

This looks great.
But it's a bit hard to see which ones are the actual effectors.
I'll do a plot that just colours by whether it matches a name.

In [16]:

```
eff_colour = subG.new_vertex_property("vector<float>")

for v in subG.vertices():
    e = subG.vp["effectors"][v]
    if e is None or e == "":
        eff_colour[v] =  [91/256, 176/256, 201/256, 0.3] # light blue
    else:
        eff_colour[v] = [158 / 256,  17/256, 17/256, 0.8] # dark red
```

In [17]:

```
gt.graph_draw(
    subG,
    pos=pos,
    edge_pen_width=0.1,
    inline=True,
    vcmap=cmap,
    vertex_size=vsize,
    vertex_fill_color=eff_colour,
    vertex_text=subG.vertex_properties["effectors"],
    vertex_text_position=0,
    vertex_text_offset=[-1, 1],
    vertex_pen_width=0.0,
)
```

Out[17]:

```
<VertexPropertyMap object with value type 'vector<double>', for Graph 0x7f74e96645d0, at 0x7f74e9673710>
```

Ok, it's much clearer like that :)

## Aligning the clusters.¶

Now I need to align the clusters together to see if they have a genuine relationship.

My plan is to:

1. Find the maximum spanning tree
2. For each edge in the MST, pairwise align the nodes using `hhalign` in descending order of edge weight.
   i.e. merge the profiles, merging closer ones before more distant ones.
3. Take that MSA and use it as a profile to align to using ClustalO.

The reason we don't just take 2 as the final alignment is because I'll be using the enriched sequences to combine them, but we want the un-enriched versions to visualise.
Also, hhalign throws out sequences and sometimes fails to extend alignments well.

Here's how we get the MST

In [18]:

```
# Remember that neg contains the 1 - weights. so the min tree of this is the max tree of 'weight'.
mst = gt.min_spanning_tree(subG, weights=subG.edge_properties["neg"])
subGMst = gt.GraphView(subG, efilt=mst)
```

In [19]:

```
gt.graph_draw(
    subGMst,
    pos=pos,
    edge_pen_width=esize,
    inline=True,
    vcmap=cmap,
    vertex_size=vsize,
    vertex_fill_color=vcolour,
    vertex_text=subGMst.vertex_properties["effectors"],
    vertex_text_position=0,
    vertex_text_offset=[-1, 1],
    vertex_pen_width=0.0,
)
```

Out[19]:

```
<VertexPropertyMap object with value type 'vector<double>', for Graph 0x7f74e96645d0, at 0x7f74e9673710>
```

Now i need to be able to get the cluster MSAs.

In the `data/` folder is the enriched msas, and we prepared the un-enriched msas earlier.

In [20]:

```
data = open("data/hhself_subset/db_a3m.ffdata", "rb")

with open("data/hhself_subset/db_a3m.ffindex", "rb") as handle:
    enriched = FFDB.from_file(data, handle)

print(enriched[b"PC_0000RV"][:200].decode())
```

```
>PC_0000RV_consensus
MPDRPKVSTDVTPVRGPQSAKQKPLSQEEVQAFKDVFAMFDKDGSGTITANELGDIMRSLGQKPSDSELEDMINEVDADQSGAIDFDEFLKMMSTSVKSA
DIEHETRAAFAVFDKDGSGSISADELRQVMKSLGENLTDREIDEMIAEADKDRNGTIDCVFSRDQLVIIWFLTPRNAA
```

In [21]:

```
data = open("11-msas_a3m.ffdata", "rb")

with open("11-msas_a3m.ffindex", "rb") as handle:
    unenriched = FFDB.from_file(data, handle) 

print(unenriched[b"PC_0000RV"][:200].decode())
```

```
>PC_0000RV 
MPDRPKVSTDVTPVRGPQSAKQKPLSQEEVQAFKDVFAMFDKDGSGTITANELGDIMRSLGQKPSDSELEDMINEVDADQSGAIDFDEFLKMMSTSVKSA
DIEHETRAAFAVFDKDGSGSISADELRQVMKSLGENLTDREIDEMIAEADKDRNGTIDCVFSRDQLVIIWFLTPRNAAAGTRTNGDV
```

Note the difference in size. Even clusters with only 1 member can form good HMMs.

Now I need a way to align them.

In [22]:

```
def hhalign(left, right, verbose=False):
    """ Align two a3m files using hhalign.
    If the alignment fails (does not cover both sequences, this will return None.
    """

    with NamedTemporaryFile() as lhandle, NamedTemporaryFile() as rhandle, NamedTemporaryFile('r+b') as outhandle:
        lhandle.write(left)
        lhandle.seek(0)
        rhandle.write(right)
        rhandle.seek(0)

        ps = subprocess.run(
            [
                "hhalign",
                "-i", lhandle.name,
                "-t", rhandle.name,
                "-M", "a3m",
                "-o", "/dev/null",
                "-oa3m", outhandle.name,
                "-v", "2",
                "-id", "90",
                "-diff", "500",
            ],
            capture_output=True,
            check=True
        )
        
        if verbose:
            print("stdout")
            print(ps.stdout.decode())
            print("stderr")
            print(ps.stderr.decode())

        if os.path.exists(f"{lhandle.name}.hhr"):
            os.remove(f"{lhandle.name}.hhr")
        if os.path.exists(f"{rhandle.name}.hhr"):
            os.remove(f"{rhandle.name}.hhr")

        outhandle.seek(0)
        result = outhandle.read()

    left_ids = {s.strip() for s in left.split(b"\n") if s.strip().startswith(b">")}
    right_ids = {s.strip() for s in right.split(b"\n") if s.strip().startswith(b">")}
    result_ids = {s.strip() for s in result.split(b"\n") if s.strip().startswith(b">")}
    
    left_ok = len(result_ids.intersection(left_ids)) > 0
    right_ok = len(result_ids.intersection(right_ids)) > 0    

    if left_ok and right_ok:
        return result
    else:
        if verbose:
            print("Alignment failed to combine hmms")
            print(left_ok, right_ok)
            print("\nleft")
            print(left.decode())
            
            print("\nright")
            print(right.decode())
            
            print("\nresult")
            print(result.decode())
        return None


def a3m_to_fas(a3m):
    with NamedTemporaryFile() as inhandle, NamedTemporaryFile('r+b') as outhandle:
        inhandle.write(a3m)
        inhandle.seek(0)

        ps = subprocess.run(
            " ".join([
                "${CONDA_PREFIX}/scripts/reformat.pl",
                "a3m", "fas",
                inhandle.name,
                outhandle.name,
            ]),
            shell=True,
            capture_output=True,
            check=True
        )

        outhandle.seek(0)
        result = outhandle.read()
    return result


def a3m_to_a2m(a3m):
    with NamedTemporaryFile() as inhandle, NamedTemporaryFile('r+b') as outhandle:
        inhandle.write(a3m)
        inhandle.seek(0)

        ps = subprocess.run(
            " ".join([
                "${CONDA_PREFIX}/scripts/reformat.pl",
                "a3m", "a2m",
                inhandle.name,
                outhandle.name,
            ]),
            shell=True,
            capture_output=True,
            check=True
        )

        outhandle.seek(0)
        result = outhandle.read()
    return result


def a3m_to_sto(a3m):
    with NamedTemporaryFile() as inhandle, NamedTemporaryFile('r+b') as outhandle:
        inhandle.write(a3m)
        inhandle.seek(0)

        ps = subprocess.run(
            " ".join([
                "${CONDA_PREFIX}/scripts/reformat.pl",
                "a3m", "sto",
                inhandle.name,
                outhandle.name,
            ]),
            shell=True,
            capture_output=True,
            check=True
        )

        outhandle.seek(0)
        result = outhandle.read()
    return result


def fas_to_a3m(fas):
    with NamedTemporaryFile() as inhandle, NamedTemporaryFile('r+b') as outhandle:
        inhandle.write(fas)
        inhandle.seek(0)

        ps = subprocess.run(
            " ".join([
                "${CONDA_PREFIX}/scripts/reformat.pl",
                "-M", "50",
                "fas", "a3m",
                inhandle.name,
                outhandle.name,
            ]),
            shell=True,
            capture_output=True,
            check=True
        )

        outhandle.seek(0)
        result = outhandle.read()
    return result


def fas_to_sto(fas):
    with NamedTemporaryFile() as inhandle, NamedTemporaryFile('r+b') as outhandle:
        inhandle.write(fas)
        inhandle.seek(0)

        ps = subprocess.run(
            " ".join([
                "${CONDA_PREFIX}/scripts/reformat.pl",
                "-M", "50",
                "fas", "sto",
                inhandle.name,
                outhandle.name,
            ]),
            shell=True,
            capture_output=True,
            check=True
        )

        outhandle.seek(0)
        result = outhandle.read()
    return result
```

In [23]:

```
def get_edges(G, mst):
    """ This outputs a sorted list of the edges to align from the MST."""
    out = []
    for e in G.edges():
        l, r = e
        if mst[e]:
            weight = G.ep["weight"][e]
            lid = G.vp["cluster"][l]
            rid = G.vp["cluster"][r]
            out.append((lid, rid, weight))
            
    out.sort(key=lambda t: t[2], reverse=True)
    return out
```

In [24]:

```
def progressive(G, mst, msas, verbose=False, semiverbose=True, ep="neg"):
    """ Progressively aligns the MST edges. """
    
    from random import shuffle
    from collections import defaultdict, deque
    from copy import copy

    # Remember that neg contains the 1 - weights. so the min tree of this is the max tree of 'weight'.
    # mst = gt.min_spanning_tree(G, weights=G.edge_properties[ep])
    # subG = gt.GraphView(G, efilt=mst)
    edges = get_edges(G, mst)
    
    i = 0
    
    e_to_done = defaultdict(list)
    done_to_e = defaultdict(list)
    done = copy(msas)
    edges = deque(copy(edges))

    iter_since_change = 0
    while len(edges) > 0:
        if iter_since_change > len(edges) + 1:
            break
        
        l, r, w = edges.popleft()

        if verbose:
            print("Edge", l, r, w)
        
        if l in e_to_done:
            lkey = e_to_done[l]
            if verbose:
                print("lkey", lkey)
            lkey_clusters = done_to_e.pop(lkey)
            if verbose:
                print("lkey_clusters", lkey_clusters)
        else:
            lkey = l
            lkey_clusters = [lkey]

        if r in e_to_done:
            rkey = e_to_done[r]
            rkey_clusters = done_to_e.pop(rkey)
        else:
            rkey = r
            rkey_clusters = [rkey]

        lmsa = done.pop(lkey)
        rmsa = done.pop(rkey)

        msa1 = hhalign(lmsa, rmsa)
        msa2 = hhalign(rmsa, lmsa)
        # Sometimes hhalign doesn't include both alignments.
        # We run both alignments (q vs r and r vs q), and pick the
        # one that succeeded, or has more sequences.
        # If both fail, we add both clusters back into the mix to retry aligning later.
        if (msa1 is None) and (not msa2 is None):
            msa = msa2
        elif (msa2 is None) and (not msa1 is None):
            msa = msa1
        elif (msa1 is None) and (msa2 is None):
            msa = None
        elif lmsa.count(b">") < rmsa.count(b">"):
            msa = msa1
        else:
            msa = msa2

        del msa1, msa2

        if msa is None:
            if verbose:
                print("MSA failed")

            done[lkey] = lmsa
            done[rkey] = rmsa
            done_to_e[lkey] = lkey_clusters
            done_to_e[rkey] = rkey_clusters
            
            edges.append((l, r, w))
            iter_since_change += 1
        else:
            if verbose:
                print("MSA succeeded")
            all_nodes = set()
            all_nodes.update(lkey_clusters)
            all_nodes.update(rkey_clusters)
            done[i] = msa
            done_to_e[i] = all_nodes

            for n in all_nodes:
                e_to_done[n] = i
            
            iter_since_change = 0

            i += 1

    # If we're trying to align multiple connected components
    # they won't have edges to guide the alignment.
    # Essentially we just try aligning and the align a few random permutations.
    components = deque(done.values())

    iter_since_change = 0
    while len(components) > 0:
        if len(components) == 1:
            break
        # In this case we've tried enough.
        elif iter_since_change > (10 * len(components)):
            break
        elif iter_since_change > len(components):
            tmp = list(components)
            shuffle(tmp)
            components = deque(tmp)
            del tmp

        lmsa = components.pop()
        rmsa = components.pop()
        msa1 = hhalign(lmsa, rmsa)
        msa2 = hhalign(rmsa, lmsa)

        if (msa1 is None) and (not msa2 is None):
            msa = msa2
        elif (msa2 is None) and (not msa1 is None):
            msa = msa1
        elif (msa1 is None) and (msa2 is None):
            msa = None
        elif lmsa.count(b">") < rmsa.count(b">"):
            msa = msa1
        else:
            msa = msa2

        del msa1, msa2
        
        if msa is None:
            components.append(lmsa)
            components.append(rmsa)
            iter_since_change += 1
        else:
            components.append(msa)
        
    result = list(sorted(components, key=lambda x: x.count(b">"), reverse=True))
    
    lens = ', '.join([str(r.count(b">")) for r in result])
    print(f"there are {len(result)} alignments. Each with {lens} sequences.")
    return result
```

Ok so we wrote the first few bits into a function "progressive".
It gets the MST, and progressively aligns the hmms.
At the end we'll get a list of alignments.
If some sub graphs just can't be aligned, that's ok.

In [25]:

```
# I'll also get the individual alignments as a dict for some convenience.
lm6_msas = {c: enriched[c.encode()][:-1] for c in lm6_clusters}

res = progressive(subG, mst, lm6_msas, verbose=True)
```

```
Edge PC_02OR95 PC_05KKBN 0.6076353788375854
MSA succeeded
Edge PC_04RWF3 PC_084044 0.6033158302307129
MSA succeeded
Edge PC_01GL0Z PC_06UG6Z 0.5812585949897766
MSA succeeded
Edge PC_02OR95 PC_0687YG 0.5703802108764648
lkey 0
lkey_clusters {'PC_05KKBN', 'PC_02OR95'}
MSA succeeded
Edge PC_02OR95 PC_0617QI 0.5647053122520447
lkey 3
lkey_clusters {'PC_05KKBN', 'PC_0687YG', 'PC_02OR95'}
MSA succeeded
Edge PC_04OD9C PC_08CQAE 0.5587312579154968
MSA succeeded
Edge PC_00G5J7 PC_02VR38 0.5528545379638672
MSA succeeded
Edge PC_06GC2O PC_073F8B 0.5459896922111511
MSA succeeded
Edge PC_05PCSX PC_06SD31 0.5416982173919678
MSA succeeded
Edge PC_00EVW7 PC_06I2IK 0.5379717946052551
MSA succeeded
Edge PC_00UG2O PC_03MDGJ 0.5347541570663452
MSA succeeded
Edge PC_00UG2O PC_06GC2O 0.5320342183113098
lkey 10
lkey_clusters {'PC_03MDGJ', 'PC_00UG2O'}
MSA succeeded
Edge PC_07TWLV PC_08AM85 0.5278814435005188
MSA succeeded
Edge PC_03V7A5 PC_073F8B 0.5200820565223694
MSA succeeded
Edge PC_04OD9C PC_08JYGI 0.5000483989715576
lkey 5
lkey_clusters {'PC_08CQAE', 'PC_04OD9C'}
MSA succeeded
Edge PC_04IZL7 PC_06SD31 0.4920383393764496
MSA succeeded
Edge PC_00EVW7 PC_033XMO 0.4873012602329254
lkey 9
lkey_clusters {'PC_06I2IK', 'PC_00EVW7'}
MSA succeeded
Edge PC_03MDGJ PC_0475IM 0.4819457232952118
lkey 13
lkey_clusters {'PC_06GC2O', 'PC_03MDGJ', 'PC_00UG2O', 'PC_073F8B', 'PC_03V7A5'}
MSA succeeded
Edge PC_02S3U6 PC_0622N2 0.4789322018623352
MSA succeeded
Edge PC_02YGO1 PC_03MDGJ 0.4653546214103699
MSA succeeded
Edge PC_014XUC PC_01AVAY 0.46165308356285095
MSA succeeded
Edge PC_00LU2L PC_029JDT 0.4603760540485382
MSA succeeded
Edge PC_02TF7C PC_07UDSR 0.4580874741077423
MSA succeeded
Edge PC_01204B PC_08JYGI 0.44878336787223816
MSA succeeded
Edge PC_06K5VR PC_085WBH 0.4451412558555603
MSA succeeded
Edge PC_029JDT PC_07VAQ1 0.44200101494789124
lkey 21
lkey_clusters {'PC_00LU2L', 'PC_029JDT'}
MSA succeeded
Edge PC_02PZHW PC_02YGO1 0.43246784806251526
MSA succeeded
Edge PC_01204B PC_0803KN 0.42920365929603577
lkey 23
lkey_clusters {'PC_01204B', 'PC_04OD9C', 'PC_08CQAE', 'PC_08JYGI'}
MSA succeeded
Edge PC_04OSH3 PC_073F8B 0.4291285276412964
MSA succeeded
Edge PC_06K5VR PC_085TUP 0.4244011342525482
lkey 24
lkey_clusters {'PC_085WBH', 'PC_06K5VR'}
MSA succeeded
Edge PC_00L9FI PC_0211IV 0.4178187847137451
MSA succeeded
Edge PC_04HM3F PC_073F8B 0.4127996563911438
MSA succeeded
Edge PC_01204B PC_06K5VR 0.4075726866722107
lkey 27
lkey_clusters {'PC_04OD9C', 'PC_08CQAE', 'PC_01204B', 'PC_08JYGI', 'PC_0803KN'}
MSA succeeded
Edge PC_02E9AS PC_0683F7 0.4048532247543335
MSA succeeded
Edge PC_01204B PC_05ZYD4 0.4037858843803406
lkey 32
lkey_clusters {'PC_01204B', 'PC_08JYGI', 'PC_085TUP', 'PC_04OD9C', 'PC_085WBH', 'PC_06K5VR', 'PC_0803KN', 'PC_08CQAE'}
MSA succeeded
Edge PC_00TUNG PC_06O7HH 0.3986985683441162
MSA succeeded
Edge PC_00L9FI PC_06O7HH 0.39267706871032715
lkey 30
lkey_clusters {'PC_00L9FI', 'PC_0211IV'}
MSA succeeded
Edge PC_0879T3 PC_08BIP7 0.37536707520484924
MSA succeeded
Edge PC_0877SL PC_0879T3 0.3747767210006714
MSA succeeded
Edge PC_00LU2L PC_07A69X 0.36399880051612854
lkey 25
lkey_clusters {'PC_00LU2L', 'PC_029JDT', 'PC_07VAQ1'}
MSA succeeded
Edge PC_02YGO1 PC_063YDL 0.3519645929336548
lkey 31
lkey_clusters {'PC_03MDGJ', 'PC_00UG2O', 'PC_04OSH3', 'PC_073F8B', 'PC_02PZHW', 'PC_0475IM', 'PC_04HM3F', 'PC_02YGO1', 'PC_06GC2O', 'PC_03V7A5'}
MSA succeeded
Edge PC_007WD7 PC_02E9AS 0.35185861587524414
MSA succeeded
Edge PC_00QDN6 PC_0879T3 0.3508147895336151
MSA succeeded
Edge PC_0879T3 PC_087AH5 0.34897691011428833
lkey 42
lkey_clusters {'PC_08BIP7', 'PC_0879T3', 'PC_0877SL', 'PC_00QDN6'}
MSA succeeded
Edge PC_004AOC PC_02E9AS 0.34323838353157043
MSA succeeded
Edge PC_02PZHW PC_03J9TR 0.33959031105041504
lkey 40
lkey_clusters {'PC_063YDL', 'PC_03MDGJ', 'PC_00UG2O', 'PC_04OSH3', 'PC_073F8B', 'PC_02PZHW', 'PC_0475IM', 'PC_04HM3F', 'PC_02YGO1', 'PC_06GC2O', 'PC_03V7A5'}
MSA succeeded
Edge PC_04IZL7 PC_06YSDT 0.3012208044528961
lkey 15
lkey_clusters {'PC_04IZL7', 'PC_05PCSX', 'PC_06SD31'}
MSA succeeded
Edge PC_01204B PC_08GN0W 0.2988732159137726
lkey 34
lkey_clusters {'PC_05ZYD4', 'PC_04OD9C', 'PC_085WBH', 'PC_08CQAE', 'PC_01204B', 'PC_08JYGI', 'PC_085TUP', 'PC_06K5VR', 'PC_0803KN'}
MSA succeeded
Edge PC_00LU2L PC_07UDSR 0.2923669219017029
lkey 39
lkey_clusters {'PC_00LU2L', 'PC_07A69X', 'PC_029JDT', 'PC_07VAQ1'}
MSA succeeded
Edge PC_02VR38 PC_06O7HH 0.28206413984298706
lkey 6
lkey_clusters {'PC_00G5J7', 'PC_02VR38'}
MSA succeeded
Edge PC_00LU2L PC_05PCSX 0.2652103900909424
lkey 48
lkey_clusters {'PC_00LU2L', 'PC_02TF7C', 'PC_029JDT', 'PC_07A69X', 'PC_07VAQ1', 'PC_07UDSR'}
MSA succeeded
Edge PC_00MV3G PC_0617QI 0.24672473967075348
MSA succeeded
Edge PC_02BPO1 PC_06O7HH 0.24493801593780518
MSA succeeded
Edge PC_004AOC PC_00WOKE 0.23818056285381317
lkey 44
lkey_clusters {'PC_0683F7', 'PC_02E9AS', 'PC_004AOC', 'PC_007WD7'}
MSA succeeded
Edge PC_007WD7 PC_01AVAY 0.2270980179309845
lkey 53
lkey_clusters {'PC_00WOKE', 'PC_02E9AS', 'PC_004AOC', 'PC_0683F7', 'PC_007WD7'}
MSA succeeded
Edge PC_01204B PC_04Y56R 0.21280565857887268
lkey 47
lkey_clusters {'PC_05ZYD4', 'PC_04OD9C', 'PC_085WBH', 'PC_08CQAE', 'PC_01204B', 'PC_08JYGI', 'PC_085TUP', 'PC_06K5VR', 'PC_0803KN', 'PC_08GN0W'}
MSA succeeded
Edge PC_07N5OJ PC_0803KN 0.20192405581474304
MSA succeeded
Edge PC_06O7HH PC_087AH5 0.19200515747070312
lkey 52
lkey_clusters {'PC_00TUNG', 'PC_00L9FI', 'PC_02VR38', 'PC_06O7HH', 'PC_00G5J7', 'PC_02BPO1', 'PC_0211IV'}
MSA succeeded
Edge PC_03MDGJ PC_07OCCM 0.19086456298828125
lkey 45
lkey_clusters {'PC_03J9TR', 'PC_063YDL', 'PC_03MDGJ', 'PC_00UG2O', 'PC_04OSH3', 'PC_073F8B', 'PC_02PZHW', 'PC_0475IM', 'PC_04HM3F', 'PC_02YGO1', 'PC_06GC2O', 'PC_03V7A5'}
MSA succeeded
Edge PC_01AVAY PC_0617QI 0.18304207921028137
lkey 54
lkey_clusters {'PC_00WOKE', 'PC_02E9AS', 'PC_004AOC', 'PC_014XUC', 'PC_0683F7', 'PC_01AVAY', 'PC_007WD7'}
MSA succeeded
Edge PC_06O7HH PC_087ABI 0.17540070414543152
lkey 57
lkey_clusters {'PC_00L9FI', 'PC_02VR38', 'PC_06O7HH', 'PC_02BPO1', 'PC_0211IV', 'PC_00QDN6', 'PC_00TUNG', 'PC_08BIP7', 'PC_00G5J7', 'PC_087AH5', 'PC_0879T3', 'PC_0877SL'}
MSA succeeded
Edge PC_00R3TP PC_03MDGJ 0.17051522433757782
MSA succeeded
Edge PC_0803KN PC_08GKAA 0.16953915357589722
lkey 56
lkey_clusters {'PC_05ZYD4', 'PC_04OD9C', 'PC_085WBH', 'PC_04Y56R', 'PC_08CQAE', 'PC_01204B', 'PC_07N5OJ', 'PC_08JYGI', 'PC_085TUP', 'PC_06K5VR', 'PC_0803KN', 'PC_08GN0W'}
MSA succeeded
Edge PC_00EVW7 PC_08JYGI 0.1662445217370987
lkey 16
lkey_clusters {'PC_06I2IK', 'PC_033XMO', 'PC_00EVW7'}
MSA succeeded
Edge PC_06K5VR PC_084044 0.1640601009130478
lkey 63
lkey_clusters {'PC_05ZYD4', 'PC_04OD9C', 'PC_08GKAA', 'PC_08CQAE', 'PC_033XMO', 'PC_01204B', 'PC_06I2IK', 'PC_07N5OJ', 'PC_08GN0W', 'PC_085WBH', 'PC_04Y56R', 'PC_08JYGI', 'PC_085TUP', 'PC_06K5VR', 'PC_0803KN', 'PC_00EVW7'}
MSA succeeded
Edge PC_00G5J7 PC_06SD31 0.1574072539806366
lkey 60
lkey_clusters {'PC_00L9FI', 'PC_02VR38', 'PC_06O7HH', 'PC_02BPO1', 'PC_0211IV', 'PC_00QDN6', 'PC_00TUNG', 'PC_08BIP7', 'PC_00G5J7', 'PC_087AH5', 'PC_0879T3', 'PC_0877SL', 'PC_087ABI'}
MSA succeeded
Edge PC_0617QI PC_07A69X 0.15488731861114502
lkey 59
lkey_clusters {'PC_0617QI', 'PC_00WOKE', 'PC_02E9AS', 'PC_014XUC', 'PC_0687YG', 'PC_01AVAY', 'PC_00MV3G', 'PC_05KKBN', 'PC_004AOC', 'PC_0683F7', 'PC_007WD7', 'PC_02OR95'}
MSA succeeded
Edge PC_033XMO PC_08AM85 0.15040139853954315
lkey 64
lkey_clusters {'PC_05ZYD4', 'PC_04OD9C', 'PC_08GKAA', 'PC_08CQAE', 'PC_033XMO', 'PC_01204B', 'PC_084044', 'PC_06I2IK', 'PC_07N5OJ', 'PC_08GN0W', 'PC_04RWF3', 'PC_085WBH', 'PC_04Y56R', 'PC_08JYGI', 'PC_085TUP', 'PC_06K5VR', 'PC_0803KN', 'PC_00EVW7'}
MSA succeeded
Edge PC_007WD7 PC_08GQ6T 0.14426471292972565
lkey 66
lkey_clusters {'PC_07A69X', 'PC_0211IV', 'PC_05PCSX', 'PC_08BIP7', 'PC_02TF7C', 'PC_0683F7', 'PC_087AH5', 'PC_087ABI', 'PC_07VAQ1', 'PC_00QDN6', 'PC_06YSDT', 'PC_0879T3', 'PC_07UDSR', 'PC_02E9AS', 'PC_014XUC', 'PC_06O7HH', 'PC_00LU2L', 'PC_00MV3G', 'PC_06SD31', 'PC_00G5J7', 'PC_007WD7', 'PC_02OR95', 'PC_0617QI', 'PC_00L9FI', 'PC_04IZL7', 'PC_02VR38', 'PC_00WOKE', 'PC_0687YG', 'PC_029JDT', 'PC_01AVAY', 'PC_02BPO1', 'PC_00TUNG', 'PC_05KKBN', 'PC_004AOC', 'PC_0877SL'}
MSA succeeded
Edge PC_00VSX6 PC_02ITW3 0.14256232976913452
MSA succeeded
Edge PC_01GL0Z PC_08JYGI 0.13551683723926544
lkey 2
lkey_clusters {'PC_06UG6Z', 'PC_01GL0Z'}
MSA succeeded
Edge PC_01204B PC_084AW6 0.13054610788822174
lkey 70
lkey_clusters {'PC_05ZYD4', 'PC_04OD9C', 'PC_08GKAA', 'PC_08CQAE', 'PC_033XMO', 'PC_01204B', 'PC_06UG6Z', 'PC_084044', 'PC_06I2IK', 'PC_07N5OJ', 'PC_07TWLV', 'PC_01GL0Z', 'PC_08GN0W', 'PC_06K5VR', 'PC_04RWF3', 'PC_085WBH', 'PC_04Y56R', 'PC_08JYGI', 'PC_085TUP', 'PC_08AM85', 'PC_0803KN', 'PC_00EVW7'}
MSA succeeded
Edge PC_029JDT PC_084044 0.12624453008174896
lkey 68
lkey_clusters {'PC_07A69X', 'PC_0211IV', 'PC_05PCSX', 'PC_08BIP7', 'PC_02TF7C', 'PC_0683F7', 'PC_087AH5', 'PC_087ABI', 'PC_07VAQ1', 'PC_00QDN6', 'PC_08GQ6T', 'PC_06YSDT', 'PC_0879T3', 'PC_07UDSR', 'PC_02E9AS', 'PC_014XUC', 'PC_06O7HH', 'PC_00LU2L', 'PC_00MV3G', 'PC_06SD31', 'PC_00G5J7', 'PC_007WD7', 'PC_02OR95', 'PC_0617QI', 'PC_00L9FI', 'PC_04IZL7', 'PC_02VR38', 'PC_00WOKE', 'PC_0687YG', 'PC_029JDT', 'PC_01AVAY', 'PC_02BPO1', 'PC_00TUNG', 'PC_05KKBN', 'PC_004AOC', 'PC_0877SL'}
MSA succeeded
Edge PC_02ITW3 PC_04Y56R 0.12166597694158554
lkey 69
lkey_clusters {'PC_02ITW3', 'PC_00VSX6'}
MSA succeeded
Edge PC_00VSX6 PC_04IL0O 0.12055901437997818
lkey 73
lkey_clusters {'PC_05ZYD4', 'PC_07A69X', 'PC_08GKAA', 'PC_0211IV', 'PC_05PCSX', 'PC_08BIP7', 'PC_02TF7C', 'PC_08CQAE', 'PC_01204B', 'PC_084044', 'PC_07N5OJ', 'PC_0683F7', 'PC_01GL0Z', 'PC_087AH5', 'PC_087ABI', 'PC_08GN0W', 'PC_00VSX6', 'PC_04Y56R', 'PC_07VAQ1', 'PC_00QDN6', 'PC_02ITW3', 'PC_08GQ6T', 'PC_08JYGI', 'PC_06YSDT', 'PC_0879T3', 'PC_06K5VR', 'PC_08AM85', 'PC_07UDSR', 'PC_0803KN', 'PC_02E9AS', 'PC_014XUC', 'PC_06O7HH', 'PC_04OD9C', 'PC_033XMO', 'PC_00LU2L', 'PC_00MV3G', 'PC_06UG6Z', 'PC_06I2IK', 'PC_06SD31', 'PC_00G5J7', 'PC_084AW6', 'PC_07TWLV', 'PC_007WD7', 'PC_02OR95', 'PC_0617QI', 'PC_00L9FI', 'PC_04IZL7', 'PC_04RWF3', 'PC_02VR38', 'PC_00WOKE', 'PC_0687YG', 'PC_029JDT', 'PC_085WBH', 'PC_01AVAY', 'PC_02BPO1', 'PC_00TUNG', 'PC_05KKBN', 'PC_004AOC', 'PC_085TUP', 'PC_0877SL', 'PC_00EVW7'}
MSA succeeded
Edge PC_00LU2L PC_03MDGJ 0.1190008744597435
lkey 74
lkey_clusters {'PC_05ZYD4', 'PC_07A69X', 'PC_08GKAA', 'PC_0211IV', 'PC_05PCSX', 'PC_08BIP7', 'PC_02TF7C', 'PC_08CQAE', 'PC_01204B', 'PC_084044', 'PC_07N5OJ', 'PC_0683F7', 'PC_01GL0Z', 'PC_087AH5', 'PC_087ABI', 'PC_08GN0W', 'PC_00VSX6', 'PC_04Y56R', 'PC_07VAQ1', 'PC_00QDN6', 'PC_02ITW3', 'PC_08GQ6T', 'PC_08JYGI', 'PC_06YSDT', 'PC_0879T3', 'PC_06K5VR', 'PC_08AM85', 'PC_07UDSR', 'PC_0803KN', 'PC_02E9AS', 'PC_014XUC', 'PC_06O7HH', 'PC_04OD9C', 'PC_033XMO', 'PC_00LU2L', 'PC_00MV3G', 'PC_06UG6Z', 'PC_06I2IK', 'PC_06SD31', 'PC_00G5J7', 'PC_084AW6', 'PC_07TWLV', 'PC_04IL0O', 'PC_007WD7', 'PC_02OR95', 'PC_0617QI', 'PC_00L9FI', 'PC_04IZL7', 'PC_04RWF3', 'PC_02VR38', 'PC_00WOKE', 'PC_0687YG', 'PC_029JDT', 'PC_085WBH', 'PC_01AVAY', 'PC_02BPO1', 'PC_00TUNG', 'PC_05KKBN', 'PC_004AOC', 'PC_085TUP', 'PC_0877SL', 'PC_00EVW7'}
MSA succeeded
Edge PC_01DK5W PC_05ZYD4 0.1187262088060379
MSA succeeded
Edge PC_05ZYD4 PC_07NG3S 0.11581962555646896
lkey 76
lkey_clusters {'PC_07A69X', 'PC_03MDGJ', 'PC_08GKAA', 'PC_0211IV', 'PC_08CQAE', 'PC_08BIP7', 'PC_01204B', 'PC_0683F7', 'PC_087AH5', 'PC_087ABI', 'PC_04Y56R', 'PC_07VAQ1', 'PC_00QDN6', 'PC_04HM3F', 'PC_06YSDT', 'PC_0879T3', 'PC_08AM85', 'PC_0803KN', 'PC_07UDSR', 'PC_03V7A5', 'PC_07OCCM', 'PC_02E9AS', 'PC_014XUC', 'PC_06O7HH', 'PC_033XMO', 'PC_00LU2L', 'PC_06UG6Z', 'PC_06I2IK', 'PC_06SD31', 'PC_084AW6', 'PC_04IL0O', 'PC_02OR95', 'PC_04IZL7', 'PC_02VR38', 'PC_0687YG', 'PC_085WBH', 'PC_02BPO1', 'PC_073F8B', 'PC_05KKBN', 'PC_0475IM', 'PC_085TUP', 'PC_01DK5W', 'PC_05ZYD4', 'PC_05PCSX', 'PC_04OSH3', 'PC_02TF7C', 'PC_084044', 'PC_07N5OJ', 'PC_02YGO1', 'PC_01GL0Z', 'PC_08GN0W', 'PC_03J9TR', 'PC_063YDL', 'PC_00VSX6', 'PC_02ITW3', 'PC_08GQ6T', 'PC_08JYGI', 'PC_00R3TP', 'PC_06K5VR', 'PC_04OD9C', 'PC_00MV3G', 'PC_02PZHW', 'PC_00G5J7', 'PC_07TWLV', 'PC_00UG2O', 'PC_007WD7', 'PC_0617QI', 'PC_00L9FI', 'PC_04RWF3', 'PC_029JDT', 'PC_00WOKE', 'PC_01AVAY', 'PC_00TUNG', 'PC_004AOC', 'PC_06GC2O', 'PC_0877SL', 'PC_00EVW7'}
MSA succeeded
Edge PC_084YSZ PC_08GKAA 0.11386284977197647
MSA succeeded
Edge PC_007UXN PC_05PCSX 0.11298953741788864
MSA succeeded
Edge PC_007UXN PC_0622N2 0.10779482871294022
lkey 79
lkey_clusters {'PC_07A69X', 'PC_03MDGJ', 'PC_08GKAA', 'PC_0211IV', 'PC_08CQAE', 'PC_08BIP7', 'PC_01204B', 'PC_0683F7', 'PC_087AH5', 'PC_087ABI', 'PC_04Y56R', 'PC_07VAQ1', 'PC_00QDN6', 'PC_04HM3F', 'PC_06YSDT', 'PC_0879T3', 'PC_08AM85', 'PC_0803KN', 'PC_07UDSR', 'PC_03V7A5', 'PC_07OCCM', 'PC_02E9AS', 'PC_014XUC', 'PC_06O7HH', 'PC_033XMO', 'PC_00LU2L', 'PC_06UG6Z', 'PC_06I2IK', 'PC_06SD31', 'PC_084AW6', 'PC_04IL0O', 'PC_02OR95', 'PC_04IZL7', 'PC_02VR38', 'PC_0687YG', 'PC_07NG3S', 'PC_085WBH', 'PC_02BPO1', 'PC_073F8B', 'PC_05KKBN', 'PC_0475IM', 'PC_085TUP', 'PC_01DK5W', 'PC_05ZYD4', 'PC_05PCSX', 'PC_04OSH3', 'PC_02TF7C', 'PC_084044', 'PC_07N5OJ', 'PC_02YGO1', 'PC_01GL0Z', 'PC_08GN0W', 'PC_03J9TR', 'PC_063YDL', 'PC_00VSX6', 'PC_02ITW3', 'PC_08GQ6T', 'PC_08JYGI', 'PC_00R3TP', 'PC_06K5VR', 'PC_04OD9C', 'PC_00MV3G', 'PC_084YSZ', 'PC_02PZHW', 'PC_00G5J7', 'PC_07TWLV', 'PC_00UG2O', 'PC_007WD7', 'PC_0617QI', 'PC_00L9FI', 'PC_04RWF3', 'PC_029JDT', 'PC_00WOKE', 'PC_007UXN', 'PC_01AVAY', 'PC_00TUNG', 'PC_004AOC', 'PC_06GC2O', 'PC_0877SL', 'PC_00EVW7'}
MSA succeeded
there are 1 alignments. Each with 107 sequences.
```

I won't print out the verbose things every time, but it's good to know what it's doing. :)

From this, I need to build a HMMER hmm, and align the sequences to it using ClustalO

In [26]:

```
def hmmbuild(profile, name=None, verbose=True):
    #profile = a3m_to_a2m(profile)
    with NamedTemporaryFile() as ihandle, NamedTemporaryFile() as ohandle:
        ihandle.write(profile)
        ihandle.seek(0)

        cmd = [
            "hmmbuild",
            "--amino",
            "--informat", "a2m",
        ]
        if name is not None:
            cmd.extend(["-n", name])

        cmd.extend([
            ohandle.name,
            ihandle.name,
        ])
        ps = subprocess.run(
            cmd,
            capture_output=True,
            check=False
        )
        
        if verbose:
            print(ps.stdout.decode())
            print(ps.stderr.decode())

        ohandle.seek(0)
        
        result = ohandle.read()
    return result
```

In [27]:

```
def clustalo(profile, seqs):
    # This is a quicky a3m to unaligned fasta conversion.
    seqs = SeqIO.parse(
        StringIO(
            seqs.replace(b"-", b"").replace(b".", b"").upper().decode()
        ),
        "fasta"
    )
    
    # Remove any duplicate sequences just in case.
    seen = set()
    seqs2 = []
    for seq in seqs:
        if seq.id not in seen:
            seqs2.append(seq)
            seen.add(seq.id)
    seqs = seqs2
    del seqs2
    del seen

    with NamedTemporaryFile() as phandle, NamedTemporaryFile(mode="w+") as shandle:
        phandle.write(profile)
        phandle.seek(0)
        
        SeqIO.write(seqs, shandle, "fasta")
        shandle.seek(0)
        try:
            ps = subprocess.run(
                [
                    "clustalo",
                    "--in", shandle.name,
                    "--hmm-in", phandle.name,
                    "--dealign",
                    "--infmt", "fas",
                    "--outfmt", "a2m",               
                ],
                capture_output=True,
                check=False
            )
        except Exception as e:
            print(ps.stdout.decode())
            print(ps.stderr.decode())
            raise e

    return ps.stdout
```

In [28]:

```
hmm = hmmbuild(res[0], verbose=True)
```

```
# hmmbuild :: profile HMM construction from multiple sequence alignments
# HMMER 3.3 (Nov 2019); http://hmmer.org/
# Copyright (C) 2019 Howard Hughes Medical Institute.
# Freely distributed under the BSD open source license.
# - - - - - - - - - - - - - - - - - - - - - - - - - - - - - - - - - - - -
# input alignment file:             /tmp/tmpyqcyo696
# output HMM file:                  /tmp/tmpf8sd_lb3
# input alignment is asserted as:   protein
# - - - - - - - - - - - - - - - - - - - - - - - - - - - - - - - - - - - -

# idx name                  nseq  alen  mlen eff_nseq re/pos description
#---- -------------------- ----- ----- ----- -------- ------ -----------
1     tmpyqcyo696            107   238   108     4.71  0.590 

# CPU time: 0.05u 0.00s 00:00:00.05 Elapsed: 00:00:00.05
```

In [29]:

```
# I'll also get the individual alignments as a dict for some convenience.
lm6_msas2 = {c: unenriched[c.encode()][:-1] for c in lm6_clusters}

a = clustalo(hmm, b"\n".join(lm6_msas2.values()))
print(a[:5000].decode())
```

```
>PC_01204B
------------------------------------------------------------
-------------------------------------------------------MRLLI
T--------LM-----------------------------------------------PF
-LLPVMASDHKQ--C--DCQVNNG------------------------------KGWEYD
---WQL--TFNACVD--------NY-S---RT---------------------------A
EY--------------------------------DTGAGRCIANP-------------HT
RLDGDR------------------FY----------------NN----------------
--CKFL--A-------K--------NG------YYPVVNGAIDTTQPK-L-------TAQ
QG-------GSGCYN---------------------------------------------
---------------
>PC_03T3PM
------------------------------------------------------------
--------------------------------------------------MKSIAILAA-
----FGL--L-PFSDV-------------------------------------L----AD
------SQSWAQ--C--SCSVWTPSGG-----------------------------WGYD
---WQL--TYNACIN--------NY-D---GA--------------------------IA
KY-DHTLG-------------------------------RCTGT-------------AGN
TIDGAR------------------WF----------------GD----------------
--CQIQ--A--------------S-DG------YFPVTYG--F--ID--M-----TQPVM
TEEA--DNVRGYCTNSS-------------------------------------------
---------------
>PC_050115
------------------------------------------------------------
--------------------------------------------------MKFSASTVL-
----FAA--LGVFFAP-------------------------------------G----VA
------ADPHYE--C--SCSTWNG-RG-----------------------------WTYD
---WQL--TFNACKN--------NY-E---GE---------------------------A
NY-NHGQG-------------------------------RCKWF-------------SHK
RVDGDD------------------WN----------------RV----------------
--CEAQ--A--------------R-DG------YYPVAND--V--ID--S-----TQPKI
TGK----SGHGFCKR---------------------------------------------
---------------
>PC_00VHHZ
------------------------------------------------------------
--------------------------------------------------MKFSASTLL-
----FAA--IGAFFAP-------------------------------------G----VA
------ADPHYE--C--SCSTWNG-RG-----------------------------WTYD
---WQL--TFNACKN--------NY-E---GE---------------------------A
NY-NHGQG-------------------------------RCKWF-------------SHK
RVDGDD------------------WN----------------HV----------------
--CEAQ--A--------------R-DG------YYPVAND--V--ID--S-----TQPKI
TGK----SGHGFCKR---------------------------------------------
---------------
>PC_01E5YS
------------------------------------------------------------
--------------------------------------------------MKFSASTIL-
----FAA--LGAFFAP-------------------------------------G----VA
------ADPHYE--C--SCSTWNG-RG-----------------------------WTYD
---WQL--TFNACKN--------NY-E---GE---------------------------A
NY-NHGQG-------------------------------RCKWF-------------SHR
RVEGDD------------------WN----------------HV----------------
--CEAQ--A--------------R-DG------YYPVAND--V--ID--S-----TQPKI
TGK----SGHGFCKR---------------------------------------------
---------------
>PC_085T9E
------------------------------------------------------------
---------------------------------------------------------MR-
----AFS--LLAFLLP-------------------------------------F----VA
------AAEHRQ--C--SCQTRTTGQD-----------------------------WIHN
---ANL--TRIVCGL--------NF-Y---DQ---------------------------A
KW-DDGSE-------------------------------RCIAF-------------SHQ
RIDGDL------------------WN----------------GD----------------
--CIQH--G-------V------V--N------GYHTFTD-GY--LD--E-----TTPLR
HVD---WAV-GDCFHT--------------------------------------------
---------------
>PC_02HO2Q
------------------------------------------------------------
-------------------------------------------------------MRFAI
T--------LL-----------------------------------------------PF
-ILPVLASDHKQ--C--DCQINNG------------------------------NGWKYD
---WQL--TFNVCAD--------NY-E---KT---------------------------A
EY--------------------------------DNGAGRCIANP-------------HV
RLDGDR------------------FY----------------QN----------------
--CKDL--A-------K--------KG------WYPVVNGAVDTTQAK-I-------YAK
QG-------GSGCYN---------------------------------------------
---------------
>PC_00T7ET
------------------------------------------------------------
-------------------------------------------------------MRLLI
T--------LM-----------------------------------------------PF
-LLPVMASDHKQ--C--DCQVNNG------------------------------KGWEYD
---WQL--TFNACVD--------NY-S---RT---------------------------A
EY--------------------------------DTGAGRCIANP-------------HT
RLDGDR------------------FY----------------NN----------------
--CKFL--A-------K--------NG------YYQLLMALSIPLNLS-L-------LPN
KAALVAITSGVVVLGIEL------------------------------------------
---------------
>PC_03200O
------------------------------------------------------------
-------------------------------------------------------MRFAI
T--------LL-----------------------------------------------PF
-ILPVLASDHKQ--C--DCQINNG------------------------------DGWKYD
---WQL--TFNVCAD--------NY-E---KT---------------------------A
EY--------------------------------DNGAGRCIANP-------------HT
RLDGDR---------
```

I know that there is some redundancy in the input datasets.
So let's try removing similar proteins, and convert it to a3m.

In [30]:

```
def hhfilter(fas, verbose=False):
    ps = subprocess.run(
        [
            "hhfilter",
            "-i", "/dev/stdin",
            "-o", "/dev/stdout",
            "-v", "0",
            "-M", "50",
            "-id", "90",
        ],
        input=fas,
        capture_output=True,
        check=False
    )
    if verbose:
        print(ps.stderr.decode())

    return ps.stdout
```

Now I would like to plot a logo of this.
I will try to use the HMMER hhlogo tool for this, because it gives some nice properies.

In [31]:

```
def read_logo(logo):
    res = []
    indel = []

    is_resblock = False
    is_indelblock = False
    for line in logo.decode().split("\n"):
        line = line.strip()
        if line.startswith("Residue heights"):
            is_resblock = True
            continue
        elif line.startswith("Indel values"):
            is_resblock = False
            is_indelblock = True
            continue
            
        if is_resblock:
            res.append(line.rstrip(")"))
        elif is_indelblock:
            indel.append(line)
    
    names = ['profile']+list('ACDEFGHIKLMNPQRSTVWY-')
    sep = '\s+\(\s*|:?\s+'
    resdf = pd.read_csv(
        StringIO("\n".join(res)),
        index_col=0,
        header=None,
        sep=sep,
        engine='python',
        names=names
    )
    #resdf.dropna(axis='columns', inplace=True)
    
    names = ["ins_prob", "ins_len", "occupancy"]
    indeldf = pd.read_csv(
        StringIO("\n".join(indel)),
        index_col=0,
        header=None,
        sep=sep,
        engine='python',
        names=names
    )
    return resdf, indeldf


def hmmlogo(a2m, verbose=False):
    hmm = hmmbuild(a2m, verbose=False)
    ps = subprocess.run(
        [
            "hmmlogo",
            "--height_relent_all",
            #"--height_relent_abovebg",
            "/dev/stdin",
        ],
        input=hmm,
        capture_output=True,
        check=False
    )
    if verbose:
        print(ps.stderr.decode())

    res, indel = read_logo(ps.stdout)
    res.drop(columns="-", inplace=True)
    return res, indel
```

Here we plot the logo of the HMM match states.
This corresponds to the Skylign HMM plot version showing all characters (not just those above background).

In [32]:

```
residues, indel = hmmlogo(hhfilter(a))
```

In [33]:

```
lm.Logo(residues, figsize=(30, 5), color_scheme=LOGO_PALETTE)
```

Out[33]:

```
<logomaker.src.Logo.Logo at 0x7f753f0d4450>
```

Here's the ordinary way of doing it just with information content.

In [34]:

```
seqs = list(SeqIO.parse(StringIO(a3m_to_fas(hhfilter(a)).decode()), "fasta"))
str_seqs = [str(s.seq).upper() for s in seqs]

seqs_mat = lm.alignment_to_matrix(str_seqs, characters_to_ignore=".-X", to_type="information")

lm.Logo(seqs_mat, figsize=(30, 5), color_scheme=LOGO_PALETTE)
```

Out[34]:

```
<logomaker.src.Logo.Logo at 0x7f747467f210>
```

And if we exclude some positions with too many gaps.

In [35]:

```
lm.Logo(
    seqs_mat[seqs_mat.sum(axis=1) >= 0.5].reset_index(drop=True),
    figsize=(30, 5),
    color_scheme=LOGO_PALETTE
)
```

Out[35]:

```
<logomaker.src.Logo.Logo at 0x7f7464ef9550>
```

This is really neat.

We do get slightly different results between the HMM and regular versions, but essentially they show the same thing.

I'm pleased that the regular version filtered to only include positions with > 50% matches works well.
This means we can do multiple logos and apply the same filter.

I also want a way to plot the full sequencem but we'll do that later.

In [36]:

```
eff_with_unique = pd.read_csv("data/profile.tsv", sep="\t")
eff_with_unique = pd.merge(eff, eff_with_unique, on="cluster", how="left")
eff_with_unique
```

Out[36]:

|  | supercluster\_component | supercluster\_markov | supercluster\_greedy | cluster | effectors | phibase | organisms | lca\_rank | lca | cluster\_size | cluster\_length | member |
| --- | --- | --- | --- | --- | --- | --- | --- | --- | --- | --- | --- | --- |
| 0 | PC\_0000B2 | PC\_0000B2 | PC\_0000B2 | PC\_0000B2 | NaN | NaN | Pseudogymnoascus sp. VKM F-4519 (FW-2642) | species | Pseudogymnoascus sp. VKM F-4519 (FW-2642) | 1 | 1103 | PC\_0000B2 |
| 1 | PC\_0000B2 | PC\_0000B2 | PC\_0000B2 | PC\_07SB5M | NaN | NaN | Epichloe bromicola;Epichloe uncinata | genus | Epichloe | 2 | 1128 | PC\_07SB5M |
| 2 | PC\_0000B2 | PC\_0000B2 | PC\_0000B2 | PC\_07SB5M | NaN | NaN | Epichloe bromicola;Epichloe uncinata | genus | Epichloe | 2 | 1128 | PC\_07QFU0 |
| 3 | PC\_0000RV | PC\_0000RV | PC\_0000RV | PC\_0000RV | NaN | NaN | Phialophora attinorum | species | Phialophora attinorum | 1 | 237 | PC\_0000RV |
| 4 | PC\_0000RV | PC\_0000RV | PC\_0000RV | PC\_057HPB | NaN | NaN | Colletotrichum orbiculare MAFF 240422 | strain | Colletotrichum orbiculare MAFF 240422 | 1 | 281 | PC\_057HPB |
| ... | ... | ... | ... | ... | ... | ... | ... | ... | ... | ... | ... | ... |
| 3224490 | NaN | NaN | NaN | PC\_08L6K4 | NaN | NaN | Alternaria mali | species | Alternaria mali | 1 | 956 | PC\_08L6K4 |
| 3224491 | NaN | NaN | NaN | PC\_08L6Q0 | NaN | NaN | Alternaria destruens;Alternaria dauci;Alternar... | genus | Alternaria | 3 | 204 | PC\_08L6Q0 |
| 3224492 | NaN | NaN | NaN | PC\_08L6Q0 | NaN | NaN | Alternaria destruens;Alternaria dauci;Alternar... | genus | Alternaria | 3 | 204 | PC\_08KBT3 |
| 3224493 | NaN | NaN | NaN | PC\_08L6Q0 | NaN | NaN | Alternaria destruens;Alternaria dauci;Alternar... | genus | Alternaria | 3 | 204 | PC\_07VSXE |
| 3224494 | NaN | NaN | NaN | PC\_08L6ZT | NaN | NaN | Serendipita vermifera MAFF 305830 | strain | Serendipita vermifera MAFF 305830 | 1 | 1777 | PC\_08L6ZT |

3224495 rows × 12 columns

In [ ]:

## Wrapping it all together¶

I need to be able to plot the graph, get the MSA, get the logos, and the logos for each supercluster in the clusters of interest.

In [37]:

```
def plot_graph(
    name,
    G,
    pos,
    esize,
    vsize,
    df,
    cluster_column,
    supercluster_column,
    palette,
    outdir,
):

    vcolour = G.new_vertex_property("vector<float>")
    mapping = {
        k: v
        for v, k
        in enumerate(
            df
            .sort_values("effectors", ascending=False)
            [supercluster_column]
            .unique()
        )
    }

    cpalette = sns.color_palette(palette, len(mapping))
    cmap = ListedColormap(cpalette, N=len(mapping))

    colourmap = {k: cmap(v) for k, v in mapping.items()}
    
    for v in G.vertices():
        vcolour[v] = colourmap[G.vertex_properties[supercluster_column][v]]

    for ext in ["svg", "png"]:
        gt.graph_draw(
            G,
            output=os.path.join(outdir, f"{name}-subgraph_{supercluster_column}-labelled.{ext}"),
            pos=pos,
            edge_pen_width=esize,
            inline=True,
            vertex_size=vsize,
            vertex_fill_color=vcolour,
            vertex_text=subG.vertex_properties["effectors"],
            vertex_text_position=0,
            vertex_text_offset=[-1, 1],
            vertex_pen_width=0.0,
        )

    for ext in ["svg", "png"]:
        gt.graph_draw(
            G,
            output=os.path.join(outdir, f"{name}-subgraph_{supercluster_column}-unlabelled.{ext}"),
            pos=pos,
            edge_pen_width=esize,
            inline=True,
            vertex_size=vsize,
            vertex_fill_color=vcolour,
            vertex_pen_width=0.0,
        )
    
    gt.graph_draw(
        G,
        pos=pos,
        edge_pen_width=esize,
        inline=True,
        vertex_size=vsize,
        vertex_fill_color=vcolour,
        vertex_text=subG.vertex_properties["effectors"],
        vertex_text_position=0,
        vertex_text_offset=[-1, 1],
        vertex_pen_width=0.0,
    )    
    
    return colourmap
```

In [38]:

```
def runit(
    name,
    G,
    df,
    enriched,
    nonenriched,
    super_column="supercluster_markov",
    cluster_column="cluster",
    sequence_column="member",
    outdir="11-align_hmms",
    palette=PALETTE
):
    """ """
    os.makedirs(outdir, exist_ok=True)
    subG = get_subgraph(G, df[cluster_column].unique())
    pos = gt.sfdp_layout(subG, eweight=subG.edge_properties["weight"])
    esize = gt.prop_to_size(subG.edge_properties["weight"], mi=0.5, ma=3)
    vsize = gt.prop_to_size(subG.vp["size"], mi=10, ma=20, log=True)

    print("markov")
    markov_colourmap = plot_graph(
        name,
        subG,
        pos,
        esize,
        vsize,
        df,
        cluster_column,
        "supercluster_markov",
        palette,
        outdir,
    )

    print("greedy")
    greedy_colourmap = plot_graph(
        name,
        subG,
        pos,
        esize,
        vsize,
        df,
        cluster_column,
        "supercluster_greedy",
        palette,
        outdir,
    )
    
    # Plot one with just the effector names, transparent nodes.
    eff_colour = subG.new_vertex_property("vector<float>")

    for v in subG.vertices():
        e = subG.vp["effectors"][v]
        if e is None or e == "":
            eff_colour[v] =  [91/256, 176/256, 201/256, 0.3]
        else:
            eff_colour[v] = [158 / 256,  17/256, 17/256, 0.8]

    gt.graph_draw(
        subG,
        pos=pos,
        edge_pen_width=0.1,
        output=os.path.join(outdir, f"{name}-subgraph_effectors.svg"),
        inline=True,
        vcmap=cmap,
        vertex_size=vsize,
        vertex_fill_color=eff_colour,
        vertex_text=subG.vertex_properties["effectors"],
        vertex_text_position=0,
        vertex_text_offset=[-1, 1],
        vertex_pen_width=0.0,
    )

    # Remember that neg contains the 1 - weights. so the min tree of this is the max tree of 'weight'.
    mst = gt.min_spanning_tree(subG, weights=subG.edge_properties["neg"])
    subGMst = gt.GraphView(subG, efilt=mst)

    print("markov mst")
    plot_graph(
        f"{name}-mst",
        subGMst,
        pos,
        esize,
        vsize,
        df,
        cluster_column,
        "supercluster_markov",
        palette,
        outdir,
    )

    print("greedy mst")
    plot_graph(
        f"{name}-mst",
        subGMst,
        pos,
        esize,
        vsize,
        df,
        cluster_column,
        "supercluster_greedy",
        palette,
        outdir,
    )
    
    cluster_msas = {c: enriched[c.encode()][:-1] for c in df[cluster_column].unique()}
    prog_msas = progressive(subG, mst, cluster_msas, verbose=False)

    hmms = [hmmbuild(m, name=name, verbose=True) for m in prog_msas]
    
    original_msas = b"\n".join(nonenriched[c.encode()][:-1] for c in df[cluster_column].unique())
    remsas = [clustalo(h, original_msas) for h in hmms]

    seqmsas = [SeqIO.to_dict(SeqIO.parse(StringIO(m.decode()), "fasta"))
               for m in remsas]
    seqdfs = []
    for m in seqmsas:
        this_df = df.copy()
        this_df["alignment"] = this_df[sequence_column].apply(lambda k: str(m.get(k).seq).upper())
        seqdfs.append(this_df)

    filteredmsas = [SeqIO.to_dict(SeqIO.parse(StringIO(a3m_to_fas(hhfilter(m)).decode()), "fasta"))
                    for m in remsas]

    filtered_dfs = []
    for m in filteredmsas:
        this_df = df.copy()
        this_df["alignment"] = this_df[sequence_column].apply(m.get).apply(lambda s: pd.NA if s is None else str(s.seq).upper())
        this_df = this_df[this_df["alignment"].notnull()]
        filtered_dfs.append(this_df)

    return hmms, remsas, seqdfs, filtered_dfs, markov_colourmap, greedy_colourmap
```

In [ ]:

In [39]:

```
avrlm6_df = eff_with_unique[
    eff_with_unique["supercluster_component"]
    .isin(eff_with_unique.loc[eff_with_unique["effectors"] == "LmAvrLm6", "supercluster_component"])
]
```

In [40]:

```
hmms, msas, unfiltered_dfs, filtered_dfs, markov_cmap, greedy_cmap = runit(
    "avrlm6_cbnip1_mobas4_mospd5_folsix5",
    G,
    avrlm6_df,
    enriched,
    unenriched,
)
```

```
markov
```

```
greedy
```

```
markov mst
```

```
greedy mst
```

```
there are 1 alignments. Each with 107 sequences.
# hmmbuild :: profile HMM construction from multiple sequence alignments
# HMMER 3.3 (Nov 2019); http://hmmer.org/
# Copyright (C) 2019 Howard Hughes Medical Institute.
# Freely distributed under the BSD open source license.
# - - - - - - - - - - - - - - - - - - - - - - - - - - - - - - - - - - - -
# input alignment file:             /tmp/tmpi5vhew1a
# output HMM file:                  /tmp/tmp5kg4n2kb
# name (the single) HMM:            avrlm6_cbnip1_mobas4_mospd5_folsix5
# input alignment is asserted as:   protein
# - - - - - - - - - - - - - - - - - - - - - - - - - - - - - - - - - - - -

# idx name                  nseq  alen  mlen eff_nseq re/pos description
#---- -------------------- ----- ----- ----- -------- ------ -----------
1     avrlm6_cbnip1_mobas4_mospd5_folsix5   107   238   108     4.71  0.590 

# CPU time: 0.05u 0.00s 00:00:00.05 Elapsed: 00:00:00.05
```

And we want to get logos of those too please.

In [41]:

```
def fasttree(fas, verbose=False):
    ps = subprocess.run(
        [
            "fasttree",
        ],
        input=fas,
        capture_output=True,
        check=False
    )
    if verbose:
        print(ps.stderr.decode())

    return ps.stdout
```

In [ ]:

In [230]:

```
def plot_logo(
    df,
    name,
    max_gap=1,
    outdir=OUTDIR,
    base_width=0.5,
    width_per_res=0.1,
    height=2.5,
    alignment_col="alignment",
    font="DejaVu Sans",
    logo_palette=LOGO_PALETTE,
    show=True,
):
    mat_info = lm.alignment_to_matrix(
        df[alignment_col].str.upper(),
        characters_to_ignore=".-X",
        to_type="information"
    )

    if max_gap < 1:
        mat_counts = lm.alignment_to_matrix(
            df[alignment_col],
            characters_to_ignore=".-X",
            to_type="counts",
            pseudocount=0
        )
        mat_info = (
            mat_info[(1 - (mat_counts.sum(axis=1) / len(df[alignment_col]))) <= max_gap]
            .reset_index(drop=True)
        )
    # This font is more open.
    # cmss10
    
    fig, ax = plt.subplots(figsize=(base_width + width_per_res * len(mat_info), height))
    l = lm.Logo(mat_info, color_scheme=logo_palette, ax=ax, font_name=font)
    l.style_spines(spines=("top", "right"), visible=False)
    ax.set_xlim(-1, len(mat_info))

    fig.savefig(os.path.join(outdir, f"{name}-total_logo.svg"))
    fig.savefig(os.path.join(outdir, f"{name}-total_logo.png"), dpi=300)

    if show:
        plt.show()
    else:
        plt.close()

    return l


l = plot_logo(filtered_dfs[0], "test", max_gap=0.5, outdir=OUTDIR)
```

In [231]:

```
def plot_logo_group(
    df,
    name,
    cluster_cmap,
    row_order=None,
    min_size=0,
    max_gap=1,
    outdir=OUTDIR,
    base_width=0.5,
    width_per_res=0.1,
    height=2.0,
    supercluster_col="supercluster_markov",
    alignment_col="alignment",
    sharey=False,
    show=True,
    logo_palette=LOGO_PALETTE,
):    
    if min_size > 0:
        if row_order is None:
            x = df.groupby(supercluster_col)[alignment_col].count()
        else:
            x = (
                df[df[supercluster_col].isin(row_order)]
                .groupby(supercluster_col)
                [alignment_col]
                .count()
            )

        x = set(x[x > 10].index)
        df = df[df[supercluster_col].isin(x)]

    if max_gap < 1:
        mat_counts = lm.alignment_to_matrix(
            df[alignment_col],
            characters_to_ignore=".-X",
            to_type="counts",
            pseudocount=0
        )
        filter_ = (1 - (mat_counts.sum(axis=1) / len(df[alignment_col]))) <= max_gap
        ncols = filter_.astype(int).sum()
    else:
        ncols = len(df.iloc[0][alignment_col])

    ncl = df[supercluster_col].nunique()
    if df[supercluster_col].isnull().any():
        ncl += 1

    fig, axes = plt.subplots(
        nrows=ncl,
        sharex=True,
        sharey=sharey,
        figsize=(base_width + width_per_res * ncols, height * ncl)
    )
    
    if ncl == 1:
        axes = [axes]
    
    max_y = 0
    if row_order is None:
        row_order = df[supercluster_col].unique()
    
    for i, group in enumerate(row_order):
        if pd.isnull(group):
            assert df.loc[df[supercluster_col].isnull(), "cluster"].nunique() == 1, i
            subdf = df[df[supercluster_col].isnull()]
            group = subdf["cluster"].unique()[0]
        else:
            subdf = df[df[supercluster_col] == group]

        try:
            mat_info = lm.alignment_to_matrix(
                subdf[alignment_col].str.upper(),
                characters_to_ignore=".-X",
                to_type="information"
            )
        except Exception:
            # Logomaker doesn't make their errors public as far as I can tell.
            print("Group could not be plotted", group)
            continue

        if max_gap < 1:
            mat_info = mat_info[filter_].reset_index(drop=True)
    
        #if mat_info.sum() == 0:
        # This one is more open.
        # cmss10  

        l = lm.Logo(mat_info, color_scheme=logo_palette, ax=axes[i], font_name="DejaVu Sans")
        l.style_spines(spines=("top", "right"), visible=False)
        axes[i].set_xlim(-1, ncols)
        
        effectors = ";".join(subdf.loc[subdf["effectors"].notnull(), "effectors"].unique())
        if len(effectors) > 0:
            yl = group + "\n" + effectors
        else:
            yl = group

        axes[i].set_ylabel(yl, backgroundcolor=cluster_cmap.get(group))
        l, h = axes[i].get_ylim()
        
        if h > max_y:
            max_y = h

    if sharey:
        axes[0].set_ylim(0, max_y)
    fig.savefig(os.path.join(outdir, f"{name}-grouped_logo.svg"))
    fig.savefig(os.path.join(outdir, f"{name}-grouped_logo.png"), dpi=300)

    if show:
        plt.show()
    else:
        plt.close()

    return

plot_logo_group(filtered_dfs[0], "test", markov_cmap, max_gap=0.5, min_size=10, outdir=OUTDIR)
```

In [232]:

```
def alnlogo(
    name,
    df,
    greedy_cmap,
    markov_cmap,
    alignment_col="alignment",
    max_gap=1,
    cpalette=PALETTE,
    logo_palette=LOGO_PALETTE,
    base_width=2,
    width_per_base=0.15,
    base_height=2.5,
    height_per_seq=0.01,
    hratio=[0.2, 1],
    wratio=[0.01, 0.01, 0.01, 0.01, 1],
    outdir=OUTDIR,
    show=True,
):
    skylign_colours = {
        'F': [.16, .99, .18, 1],
        'Y': [.04, .40, .05, 1],
        'L': [.99, .60, .25, 1],
        'V': [1.0, .80, .27, 1],
        'I': [.80, .60, .24, 1],
        'H': [.40, .02, .20, 1],
        'W': [.42, .79, .42, 1],
        'A': [.99, .60, .42, 1],
        'S': [.04, .14, .98, 1],
        'T': [.17, 1.0, 1.0, 1],
        'M': [.80, .60, .80, 1],
        'N': [.21, .40, .40, 1],
        'Q': [.40, .41, .79, 1],
        'R': [.59, .02, .04, 1],
        'K': [.40, .20, .03, 1],
        'E': [.79, .04, .22, 1],
        'G': [.95, .94, .22, 1],
        'D': [.99, .05, .11, 1],
        'P': [.10, .61, .99, 1],
        'C': [.09, .60, .60, 1],
    }
    
    import numpy as np
    aa_to_int = {a: i for i, a in enumerate(skylign_colours)}
    alignmentint_to_colour = np.array([
        skylign_colours[a]
        for a, i
        in sorted(aa_to_int.items(), key=lambda t: t[1])
    ])

    def mapfn(f):
        seq = []
        index = []
        for i, b in enumerate(f):
            index.append(i)
            seq.append(aa_to_int.get(b, np.nan))
        return pd.Series(np.array(seq), index=index)

    alignment = df["alignment"].str.upper().apply(mapfn)
    
    greedy_to_int = {c: i for i, c in enumerate(greedy_cmap)}
    greedyint_to_colour = np.array([
        greedy_cmap[a]
        for a, i
        in sorted(greedy_to_int.items(), key=lambda t: t[1])
    ])

    def mapfn(f):
        return np.array(greedy_to_int[f])

    greedy_mat = df["supercluster_greedy"].apply(mapfn).values.reshape([-1, 1])
    
    markov_to_int = {c: i for i, c in enumerate(markov_cmap)}
    markovint_to_colour = np.array([
        markov_cmap[a]
        for a, i
        in sorted(markov_to_int.items(), key=lambda t: t[1])
    ])

    def mapfn(f):
        return np.array(markov_to_int[f])

    markov_mat = df["supercluster_markov"].apply(mapfn).values.reshape([-1, 1])

    
    cluster_colourmap = ListedColormap(
        sns.color_palette(cpalette, df["cluster"].nunique()),
        N=df["cluster"].nunique()
    )
    cluster_intmap = {c: i for i, c in enumerate(df["cluster"].unique())}
    cluster_mat = df["cluster"].apply(cluster_intmap.get).values.reshape([-1, 1])

    component_colourmap = ListedColormap(
        sns.color_palette(cpalette, df["supercluster_component"].nunique()),
        N=df["supercluster_component"].nunique()
    )
    component_intmap = {c: i for i, c in enumerate(df["supercluster_component"].unique())}
    component_mat = df["supercluster_component"].apply(component_intmap.get).values.reshape([-1, 1])
    
    mat_info = lm.alignment_to_matrix(
        df[alignment_col].str.upper(),
        characters_to_ignore=".-X",
        to_type="information"
    )

    if max_gap < 1:
        mat_counts = lm.alignment_to_matrix(
            df[alignment_col],
            characters_to_ignore=".-X",
            to_type="counts",
            pseudocount=0
        )
        filter_ = (1 - (mat_counts.sum(axis=1) / len(df[alignment_col]))) <= max_gap
        del mat_counts
        
        mat_info = mat_info.loc[filter_, :].reset_index(drop=True)
        alignment = alignment.loc[:, filter_]        

    fig = plt.figure(
        figsize=(
            base_width + (width_per_base * len(mat_info)),
            base_height + (height_per_seq * len(df))
        ),
        constrained_layout=False
    )

    gs = fig.add_gridspec(
        nrows=2,
        ncols=5,
        width_ratios=wratio,
        height_ratios=hratio,
        wspace=0.05,
        hspace=0.05,
    )
    axlogo = fig.add_subplot(gs[0, -1])  # Top right
    #axlogo.set_xticklabels(["" for _ in ax1.get_xticklabels()])

    axcomp = fig.add_subplot(gs[1, 0])  # Bottom left
    axcomp.set_xticklabels([])
    axcomp.set_yticklabels([])
    axcomp.set_xticks([])
    axcomp.set_yticks([])

    axmarkov = fig.add_subplot(gs[1, 1], sharey=axcomp)  # Bottom left
    axmarkov.set_xticklabels([])
    axmarkov.set_xticks([])

    axgreedy = fig.add_subplot(gs[1, 2], sharey=axcomp)  # Bottom left
    axgreedy.set_xticklabels([])
    axgreedy.set_xticks([])

    axcluster = fig.add_subplot(gs[1, 3], sharey=axcomp)  # Bottom left
    axcluster.set_xticklabels([])
    axcluster.set_xticks([])

    axali = fig.add_subplot(gs[1, 4], sharex=axlogo, sharey=axcomp)  # Bottom right
    axali.set_yticklabels([])

    l = lm.Logo(mat_info, color_scheme=logo_palette, ax=axlogo, font_name="DejaVu Sans")
    l.style_spines(spines=("top", "right"), visible=False)
    
    axali.imshow(alignment, cmap=ListedColormap(alignmentint_to_colour), interpolation="none", aspect="auto")
    axcomp.imshow(component_mat, cmap=component_colourmap, aspect="auto", interpolation="none")
    axmarkov.imshow(markov_mat, cmap=ListedColormap(markovint_to_colour), aspect="auto", interpolation="none")
    axgreedy.imshow(greedy_mat, cmap=ListedColormap(greedyint_to_colour), aspect="auto", interpolation="none")
    axcluster.imshow(cluster_mat, cmap=cluster_colourmap, aspect="auto", interpolation="none")
    
    fig.savefig(os.path.join(outdir, f"{name}-alignment_logo.svg"))
    fig.savefig(os.path.join(outdir, f"{name}-alignment_logo.png"), dpi=450)
    
    if show:
        plt.show()
    else:
        plt.close()
    return

alnlogo("test", filtered_dfs[0], greedy_cmap, markov_cmap, max_gap=0.5, outdir=OUTDIR)
```

In [45]:

```
def get_plots(
    name,
    index,
    hmms,
    msas,
    unfiltered_dfs,
    filtered_dfs,
    markov_cmap,
    greedy_cmap, 
    outdir=OUTDIR,
    palette=PALETTE,
    logo_palette=LOGO_PALETTE,
    run_fastree=True,
    show=False,
):
    hmm = hmms[index]
    msa = msas[index]
    unfiltered_df = unfiltered_dfs[index]
    filtered_df = filtered_dfs[index]
    
    filtered_df.to_csv(os.path.join(outdir, f"{name}-filtered.tsv"), sep="\t", na_rep=".")
    unfiltered_df.to_csv(os.path.join(outdir, f"{name}-unfiltered.tsv"), sep="\t", na_rep=".")

    with open(os.path.join(outdir, f"{name}_enriched.hmm"), "wb") as handle:
        handle.write(hmm)

    with open(os.path.join(outdir, f"{name}.a3m"), "wb") as handle:
        handle.write(hhfilter(msa))

    with open(os.path.join(outdir, f"{name}.hmm"), "wb") as handle:
        handle.write(hmmbuild(hhfilter(msa), name=name, verbose=True))

    fas = a3m_to_fas(msa)
    with open(os.path.join(outdir, f"{name}.fasta"), "wb") as handle:
        handle.write(fas)

    if run_fastree:
        nwk = fasttree(fas)
        with open(os.path.join(outdir, f"{name}.nwk"), "wb") as handle:
            handle.write(nwk)

    n = f"{name}-unfiltered_logo"
    print(n)
    l = plot_logo(unfiltered_df, n, show=show, outdir=outdir)
    n = f"{name}-unfiltered_logo-maxgap0.5"
    print(n)
    l = plot_logo(unfiltered_df, n, max_gap=0.5, show=show, outdir=outdir)

    n = f"{name}-filtered_logo"
    print(n)
    l = plot_logo(filtered_df, n, show=show, outdir=outdir)
    
    n = f"{name}-filtered_logo-maxgap0.5"
    print(n)
    l = plot_logo(filtered_df, n, max_gap=0.5, show=show, outdir=outdir)
                  
    n = f"{name}-filtered_logo_group"
    print(n)
    l = plot_logo_group(filtered_df, n, markov_cmap, show=show, outdir=outdir)
    n = f"{name}-filtered_logo_group-maxgap0.5"
    print(n)
    l = plot_logo_group(filtered_df, n, markov_cmap, max_gap=0.5, show=show, outdir=outdir)
    n = f"{name}-filtered_logo_group-maxgap0.5minsize10"
    print(n)
    l = plot_logo_group(filtered_df, n,
                        markov_cmap, max_gap=0.5, min_size=10, show=show, outdir=outdir)

    
    n = f"{name}-unfiltered_logo_group"
    print(n)
    l = plot_logo_group(unfiltered_df, n, markov_cmap, show=show, outdir=outdir)
    n = f"{name}-unfiltered_logo_group-maxgap0.5"
    print(n)
    l = plot_logo_group(unfiltered_df, n, markov_cmap, max_gap=0.5, show=show, outdir=outdir)
    n = f"{name}-unfiltered_logo_group-maxgap0.5minsize10"
    print(n)
    l = plot_logo_group(unfiltered_df, n, markov_cmap, max_gap=0.5, min_size=10, show=show, outdir=outdir)

    n = f"{name}-unfiltered_markov"
    print(n)
    alnlogo(
        n,
        unfiltered_df.sort_values(["supercluster_markov", "supercluster_greedy", "cluster"]),
        greedy_cmap,
        markov_cmap,
        max_gap=1,
        show=show,
        outdir=outdir,
    )

    n = f"{name}-unfiltered_greedy"
    print(n)
    alnlogo(
        n,
        unfiltered_df.sort_values(["supercluster_greedy", "supercluster_markov", "cluster"]),
        greedy_cmap,
        markov_cmap,
        max_gap=1,
        show=show,
        outdir=outdir,
    )
    
    n = f"{name}-filtered_markov"
    print(n)
    alnlogo(
        n,
        filtered_df.sort_values(["supercluster_markov", "supercluster_greedy", "cluster"]),
        greedy_cmap,
        markov_cmap,
        max_gap=1,
        show=show,
        outdir=outdir,
    )

    n = f"{name}-filtered_greedy"
    print(n)
    alnlogo(
        n,
        filtered_df.sort_values(["supercluster_greedy", "supercluster_markov", "cluster"]),
        greedy_cmap,
        markov_cmap,
        max_gap=1,
        show=show,
        outdir=outdir,
    )

    n = f"{name}-unfiltered_markov-maxgap0.5"
    print(n)
    alnlogo(
        n,
        unfiltered_df.sort_values(["supercluster_markov", "supercluster_greedy", "cluster"]),
        greedy_cmap,
        markov_cmap,
        max_gap=0.5,
        show=show,
        outdir=outdir,
    )

    n = f"{name}-unfiltered_greedy-maxgap0.5"
    print(n)
    alnlogo(
        n,
        unfiltered_df.sort_values(["supercluster_greedy", "supercluster_markov", "cluster"]),
        greedy_cmap,
        markov_cmap,
        max_gap=0.5,
        show=show,
        outdir=outdir,
    )
    
    n = f"{name}-filtered_markov-maxgap0.5"
    print(n)
    alnlogo(
        n,
        filtered_df.sort_values(["supercluster_markov", "supercluster_greedy", "cluster"]),
        greedy_cmap,
        markov_cmap,
        max_gap=0.5,
        show=show,
        outdir=outdir,
    )

    n = f"{name}-filtered_greedy-maxgap0.5"
    print(n)
    alnlogo(
        n,
        filtered_df.sort_values(["supercluster_greedy", "supercluster_markov", "cluster"]),
        greedy_cmap,
        markov_cmap,
        max_gap=0.5,
        show=show,
        outdir=outdir,
    )
    
    return
```

In [46]:

```
get_plots(
    "avrlm6_cbnip1_mobas4_mospd5_folsix5",
    0,
    hmms,
    msas,
    unfiltered_dfs,
    filtered_dfs,
    markov_cmap,
    greedy_cmap,
    run_fastree=True,
    show=True,
)
```

```
# hmmbuild :: profile HMM construction from multiple sequence alignments
# HMMER 3.3 (Nov 2019); http://hmmer.org/
# Copyright (C) 2019 Howard Hughes Medical Institute.
# Freely distributed under the BSD open source license.
# - - - - - - - - - - - - - - - - - - - - - - - - - - - - - - - - - - - -
# input alignment file:             /tmp/tmp5hyz1hjd
# output HMM file:                  /tmp/tmpq4l34bdt
# name (the single) HMM:            avrlm6_cbnip1_mobas4_mospd5_folsix5
# input alignment is asserted as:   protein
# - - - - - - - - - - - - - - - - - - - - - - - - - - - - - - - - - - - -

# idx name                  nseq  alen  mlen eff_nseq re/pos description
#---- -------------------- ----- ----- ----- -------- ------ -----------
1     avrlm6_cbnip1_mobas4_mospd5_folsix5   617   509   262    11.68  0.590 

# CPU time: 0.14u 0.00s 00:00:00.14 Elapsed: 00:00:00.14


avrlm6_cbnip1_mobas4_mospd5_folsix5-unfiltered_logo
```

```
avrlm6_cbnip1_mobas4_mospd5_folsix5-unfiltered_logo-maxgap0.5
```

```
avrlm6_cbnip1_mobas4_mospd5_folsix5-filtered_logo
```

```
avrlm6_cbnip1_mobas4_mospd5_folsix5-filtered_logo-maxgap0.5
```

```
avrlm6_cbnip1_mobas4_mospd5_folsix5-filtered_logo_group
```

```
avrlm6_cbnip1_mobas4_mospd5_folsix5-filtered_logo_group-maxgap0.5
```

```
avrlm6_cbnip1_mobas4_mospd5_folsix5-filtered_logo_group-maxgap0.5minsize10
```

```
avrlm6_cbnip1_mobas4_mospd5_folsix5-unfiltered_logo_group
```

```
avrlm6_cbnip1_mobas4_mospd5_folsix5-unfiltered_logo_group-maxgap0.5
```

```
avrlm6_cbnip1_mobas4_mospd5_folsix5-unfiltered_logo_group-maxgap0.5minsize10
```

```
avrlm6_cbnip1_mobas4_mospd5_folsix5-unfiltered_markov
```

```
avrlm6_cbnip1_mobas4_mospd5_folsix5-unfiltered_greedy
```

```
avrlm6_cbnip1_mobas4_mospd5_folsix5-filtered_markov
```

```
avrlm6_cbnip1_mobas4_mospd5_folsix5-filtered_greedy
```

```
avrlm6_cbnip1_mobas4_mospd5_folsix5-unfiltered_markov-maxgap0.5
```

```
avrlm6_cbnip1_mobas4_mospd5_folsix5-unfiltered_greedy-maxgap0.5
```

```
avrlm6_cbnip1_mobas4_mospd5_folsix5-filtered_markov-maxgap0.5
```

```
avrlm6_cbnip1_mobas4_mospd5_folsix5-filtered_greedy-maxgap0.5
```

I like this!!!!!

While I'm here i'll get some of these that are suitable for main figures (just some resizing).

In [98]:

```
alnlogo(
    "figure-avrlm6_cbnip1_mobas4_mospd5_folsix5",
    filtered_dfs[0],
    greedy_cmap,
    markov_cmap,
    max_gap=0.5,
    outdir=OUTDIR,
    width_per_base=0.07,
    wratio=[0.000, 0.015, 0.015, 0.015, 1],
    hratio=[0.1, 1]
)
```

In [99]:

```
plot_logo_group(
    filtered_dfs[0],
    "figure-avrlm6_cbnip1_mobas4_mospd5_folsix5",
    markov_cmap,
    max_gap=0.5,
    min_size=10,
    outdir=OUTDIR,
    width_per_res=0.09,
    height=1.5
)
```

In [ ]:

In [ ]:

In [ ]:

## Churn out the plots please.¶

I need to also run the same for other groups.

In [62]:

```
toxa_fom2_df = eff_with_unique[
    eff_with_unique["supercluster_component"]
    .isin(eff_with_unique.loc[
        eff_with_unique["effectors"].isin(['ChToxA;PtrToxA;PnToxA', 'Fom_2;FOMG_19011']), "supercluster_component"])
]
toxa_fom2_df.head()
```

Out[62]:

|  | supercluster\_component | supercluster\_markov | supercluster\_greedy | cluster | effectors | phibase | organisms | lca\_rank | lca | cluster\_size | cluster\_length | member |
| --- | --- | --- | --- | --- | --- | --- | --- | --- | --- | --- | --- | --- |
| 195035 | PC\_03B2DN | PC\_01UOBI | PC\_03B2DN | PC\_01UOBI | ChToxA;PtrToxA;PnToxA | P78737#PHI:534\_\_PHI:8380;Q0TVA4#PHI:1081;M2URZ... | Pyrenophora teres f. maculata;Parastagonospora... | suborder | Pleosporineae | 41 | 150 | PC\_01UOBI |
| 195036 | PC\_03B2DN | PC\_01UOBI | PC\_03B2DN | PC\_01UOBI | ChToxA;PtrToxA;PnToxA | P78737#PHI:534\_\_PHI:8380;Q0TVA4#PHI:1081;M2URZ... | Pyrenophora teres f. maculata;Parastagonospora... | suborder | Pleosporineae | 41 | 150 | PC\_00IPRC |
| 195037 | PC\_03B2DN | PC\_01UOBI | PC\_03B2DN | PC\_01UOBI | ChToxA;PtrToxA;PnToxA | P78737#PHI:534\_\_PHI:8380;Q0TVA4#PHI:1081;M2URZ... | Pyrenophora teres f. maculata;Parastagonospora... | suborder | Pleosporineae | 41 | 150 | PC\_022CB0 |
| 195038 | PC\_03B2DN | PC\_01UOBI | PC\_03B2DN | PC\_01UOBI | ChToxA;PtrToxA;PnToxA | P78737#PHI:534\_\_PHI:8380;Q0TVA4#PHI:1081;M2URZ... | Pyrenophora teres f. maculata;Parastagonospora... | suborder | Pleosporineae | 41 | 150 | PC\_01VEQU |
| 195039 | PC\_03B2DN | PC\_01UOBI | PC\_03B2DN | PC\_01UOBI | ChToxA;PtrToxA;PnToxA | P78737#PHI:534\_\_PHI:8380;Q0TVA4#PHI:1081;M2URZ... | Pyrenophora teres f. maculata;Parastagonospora... | suborder | Pleosporineae | 41 | 150 | PC\_00NLG8 |

In [94]:

```
toxa_fom2_df["effectors"].unique()
```

Out[94]:

```
array(['ChToxA;PtrToxA;PnToxA', nan, 'Fom_2;FOMG_19011',
       'EXF72942.1;XP003298007.1',
       'EUC36307.1;ChEC13;EUC44184.1;EUN25607.1;EKG15312.1',
       'CCE26798.1;CCE26799.1'], dtype=object)
```

In [63]:

```
toxa_hmms, toxa_msas, toxa_unfiltered_dfs, toxa_filtered_dfs, toxa_markov_cmap, toxa_greedy_cmap = runit(
    "toxa_fom2",
    G,
    toxa_fom2_df,
    enriched,
    unenriched,
)
```

```
markov
```

```
greedy
```

```
markov mst
```

```
greedy mst
```

```
there are 1 alignments. Each with 69 sequences.
# hmmbuild :: profile HMM construction from multiple sequence alignments
# HMMER 3.3 (Nov 2019); http://hmmer.org/
# Copyright (C) 2019 Howard Hughes Medical Institute.
# Freely distributed under the BSD open source license.
# - - - - - - - - - - - - - - - - - - - - - - - - - - - - - - - - - - - -
# input alignment file:             /tmp/tmpdc4mq38z
# output HMM file:                  /tmp/tmpgi96x7d0
# name (the single) HMM:            toxa_fom2
# input alignment is asserted as:   protein
# - - - - - - - - - - - - - - - - - - - - - - - - - - - - - - - - - - - -

# idx name                  nseq  alen  mlen eff_nseq re/pos description
#---- -------------------- ----- ----- ----- -------- ------ -----------
1     toxa_fom2               69   222   121     3.26  0.590 

# CPU time: 0.05u 0.00s 00:00:00.05 Elapsed: 00:00:00.05
```

In [64]:

```
get_plots(
    "toxa_fom2",
    0,
    toxa_hmms,
    toxa_msas,
    toxa_unfiltered_dfs,
    toxa_filtered_dfs,
    toxa_markov_cmap,
    toxa_greedy_cmap,
    run_fastree=True,
    show=True,
)
```

```
# hmmbuild :: profile HMM construction from multiple sequence alignments
# HMMER 3.3 (Nov 2019); http://hmmer.org/
# Copyright (C) 2019 Howard Hughes Medical Institute.
# Freely distributed under the BSD open source license.
# - - - - - - - - - - - - - - - - - - - - - - - - - - - - - - - - - - - -
# input alignment file:             /tmp/tmpxznjykvt
# output HMM file:                  /tmp/tmp34_m7i1g
# name (the single) HMM:            toxa_fom2
# input alignment is asserted as:   protein
# - - - - - - - - - - - - - - - - - - - - - - - - - - - - - - - - - - - -

# idx name                  nseq  alen  mlen eff_nseq re/pos description
#---- -------------------- ----- ----- ----- -------- ------ -----------
1     toxa_fom2               71   368   189     5.40  0.590 

# CPU time: 0.08u 0.01s 00:00:00.09 Elapsed: 00:00:00.08


toxa_fom2-unfiltered_logo
```

```
toxa_fom2-unfiltered_logo-maxgap0.5
```

```
toxa_fom2-filtered_logo
```

```
toxa_fom2-filtered_logo-maxgap0.5
```

```
toxa_fom2-filtered_logo_group
```

```
toxa_fom2-filtered_logo_group-maxgap0.5
```

```
toxa_fom2-filtered_logo_group-maxgap0.5minsize10
```

```
toxa_fom2-unfiltered_logo_group
```

```
toxa_fom2-unfiltered_logo_group-maxgap0.5
```

```
toxa_fom2-unfiltered_logo_group-maxgap0.5minsize10
```

```
toxa_fom2-unfiltered_markov
```

```
toxa_fom2-unfiltered_greedy
```

```
toxa_fom2-filtered_markov
```

```
toxa_fom2-filtered_greedy
```

```
toxa_fom2-unfiltered_markov-maxgap0.5
```

```
toxa_fom2-unfiltered_greedy-maxgap0.5
```

```
toxa_fom2-filtered_markov-maxgap0.5
```

```
toxa_fom2-filtered_greedy-maxgap0.5
```

In [176]:

```
alnlogo(
    "figure-toxa_fom2-filtered",
    toxa_filtered_dfs[0],
    toxa_greedy_cmap,
    toxa_markov_cmap,
    max_gap=0.5,
    outdir=OUTDIR,
    width_per_base=0.07,
    wratio=[0.01, 0.01, 0.01, 0.01, 1],
    hratio=[0.2, 1]
)
```

In [181]:

```
plot_logo_group(
    toxa_filtered_dfs[0],
    "figure-toxa_fom2-filtered",
    toxa_markov_cmap,
    max_gap=0.5,
    min_size=0,
    outdir=OUTDIR,
    width_per_res=0.09,
    height=1.5
)
```

In [182]:

```
avrlm2_df = eff_with_unique[
    eff_with_unique["supercluster_component"]
    .isin(eff_with_unique.loc[
        eff_with_unique["effectors"].isin(['LmAvrLm2']), "supercluster_component"])
]
avrlm2_df.head()
```

Out[182]:

|  | supercluster\_component | supercluster\_markov | supercluster\_greedy | cluster | effectors | phibase | organisms | lca\_rank | lca | cluster\_size | cluster\_length | member |
| --- | --- | --- | --- | --- | --- | --- | --- | --- | --- | --- | --- | --- |
| 381711 | PC\_053NVO | PC\_02XUK5 | PC\_03DVFF | PC\_03DVFF | NaN | NaN | Fusarium oxysporum | species | Fusarium oxysporum | 1 | 154 | PC\_03DVFF |
| 381712 | PC\_053NVO | PC\_02XUK5 | PC\_053NVO | PC\_01LZTX | FolSix1 | X0J1D8#PHI:8628;Q709E2#PHI:379;Q709D8#PHI:5284 | Colletotrichum trifolii;Fusarium oxysporum f. ... | subclass | Hypocreomycetidae | 40 | 272 | PC\_01LZTX |
| 381713 | PC\_053NVO | PC\_02XUK5 | PC\_053NVO | PC\_01LZTX | FolSix1 | X0J1D8#PHI:8628;Q709E2#PHI:379;Q709D8#PHI:5284 | Colletotrichum trifolii;Fusarium oxysporum f. ... | subclass | Hypocreomycetidae | 40 | 272 | PC\_06245F |
| 381714 | PC\_053NVO | PC\_02XUK5 | PC\_053NVO | PC\_01LZTX | FolSix1 | X0J1D8#PHI:8628;Q709E2#PHI:379;Q709D8#PHI:5284 | Colletotrichum trifolii;Fusarium oxysporum f. ... | subclass | Hypocreomycetidae | 40 | 272 | PC\_003OT8 |
| 381715 | PC\_053NVO | PC\_02XUK5 | PC\_053NVO | PC\_01LZTX | FolSix1 | X0J1D8#PHI:8628;Q709E2#PHI:379;Q709D8#PHI:5284 | Colletotrichum trifolii;Fusarium oxysporum f. ... | subclass | Hypocreomycetidae | 40 | 272 | PC\_06TP87 |

In [183]:

```
avrlm2_hmms, avrlm2_msas, avrlm2_unfiltered_dfs, avrlm2_filtered_dfs, avrlm2_markov_cmap, avrlm2_greedy_cmap = runit(
    "avrlm2",
    G,
    avrlm2_df,
    enriched,
    unenriched,
)
```

```
markov
```

```
greedy
```

```
markov mst
```

```
greedy mst
```

```
there are 1 alignments. Each with 62 sequences.
# hmmbuild :: profile HMM construction from multiple sequence alignments
# HMMER 3.3 (Nov 2019); http://hmmer.org/
# Copyright (C) 2019 Howard Hughes Medical Institute.
# Freely distributed under the BSD open source license.
# - - - - - - - - - - - - - - - - - - - - - - - - - - - - - - - - - - - -
# input alignment file:             /tmp/tmpbqxwf0fq
# output HMM file:                  /tmp/tmp6fc1yt2j
# name (the single) HMM:            avrlm2
# input alignment is asserted as:   protein
# - - - - - - - - - - - - - - - - - - - - - - - - - - - - - - - - - - - -

# idx name                  nseq  alen  mlen eff_nseq re/pos description
#---- -------------------- ----- ----- ----- -------- ------ -----------
1     avrlm2                  62   342   235     2.93  0.590 

# CPU time: 0.10u 0.00s 00:00:00.10 Elapsed: 00:00:00.10
```

In [184]:

```
get_plots(
    "avrlm2",
    0,
    avrlm2_hmms,
    avrlm2_msas,
    avrlm2_unfiltered_dfs,
    avrlm2_filtered_dfs,
    avrlm2_markov_cmap,
    avrlm2_greedy_cmap,
    run_fastree=True,
    show=True,
)
```

```
# hmmbuild :: profile HMM construction from multiple sequence alignments
# HMMER 3.3 (Nov 2019); http://hmmer.org/
# Copyright (C) 2019 Howard Hughes Medical Institute.
# Freely distributed under the BSD open source license.
# - - - - - - - - - - - - - - - - - - - - - - - - - - - - - - - - - - - -
# input alignment file:             /tmp/tmpgu42isj3
# output HMM file:                  /tmp/tmp3g5htcf7
# name (the single) HMM:            avrlm2
# input alignment is asserted as:   protein
# - - - - - - - - - - - - - - - - - - - - - - - - - - - - - - - - - - - -

# idx name                  nseq  alen  mlen eff_nseq re/pos description
#---- -------------------- ----- ----- ----- -------- ------ -----------
1     avrlm2                  37   431   258     3.18  0.590 

# CPU time: 0.10u 0.01s 00:00:00.11 Elapsed: 00:00:00.11


avrlm2-unfiltered_logo
```

```
avrlm2-unfiltered_logo-maxgap0.5
```

```
avrlm2-filtered_logo
```

```
avrlm2-filtered_logo-maxgap0.5
```

```
avrlm2-filtered_logo_group
```

```
avrlm2-filtered_logo_group-maxgap0.5
```

```
avrlm2-filtered_logo_group-maxgap0.5minsize10
```

```
avrlm2-unfiltered_logo_group
```

```
avrlm2-unfiltered_logo_group-maxgap0.5
```

```
avrlm2-unfiltered_logo_group-maxgap0.5minsize10
```

```
avrlm2-unfiltered_markov
```

```
avrlm2-unfiltered_greedy
```

```
avrlm2-filtered_markov
```

```
avrlm2-filtered_greedy
```

```
avrlm2-unfiltered_markov-maxgap0.5
```

```
avrlm2-unfiltered_greedy-maxgap0.5
```

```
avrlm2-filtered_markov-maxgap0.5
```

```
avrlm2-filtered_greedy-maxgap0.5
```

In [ ]:

In [ ]:

In [70]:

```
avrpii_df = eff_with_unique[
    eff_with_unique["supercluster_component"]
    .isin(eff_with_unique.loc[
        eff_with_unique["effectors"].isin(['MoAVR_Pii', "MoAvrPi9"]), "supercluster_component"])
]
avrpii_df.head()
```

Out[70]:

|  | supercluster\_component | supercluster\_markov | supercluster\_greedy | cluster | effectors | phibase | organisms | lca\_rank | lca | cluster\_size | cluster\_length | member |
| --- | --- | --- | --- | --- | --- | --- | --- | --- | --- | --- | --- | --- |
| 220027 | PC\_03PRB1 | PC\_00DRHD | PC\_04RYG0 | PC\_04RYG0 | NaN | NaN | Pyricularia grisea | species | Pyricularia grisea | 1 | 124 | PC\_04RYG0 |
| 220028 | PC\_03PRB1 | PC\_00DRHD | PC\_05DMGK | PC\_00DRHD | NaN | NaN | Pyricularia oryzae;Pyricularia sp. CBS 133598;... | genus | Pyricularia | 7 | 88 | PC\_00DRHD |
| 220029 | PC\_03PRB1 | PC\_00DRHD | PC\_05DMGK | PC\_00DRHD | NaN | NaN | Pyricularia oryzae;Pyricularia sp. CBS 133598;... | genus | Pyricularia | 7 | 88 | PC\_0142UF |
| 220030 | PC\_03PRB1 | PC\_00DRHD | PC\_05DMGK | PC\_00DRHD | NaN | NaN | Pyricularia oryzae;Pyricularia sp. CBS 133598;... | genus | Pyricularia | 7 | 88 | PC\_06CRT8 |
| 220031 | PC\_03PRB1 | PC\_00DRHD | PC\_05DMGK | PC\_00DRHD | NaN | NaN | Pyricularia oryzae;Pyricularia sp. CBS 133598;... | genus | Pyricularia | 7 | 88 | PC\_08JO33 |

In [71]:

```
avrpii_hmms, avrpii_msas, avrpii_unfiltered_dfs, avrpii_filtered_dfs, avrpii_markov_cmap, avrpii_greedy_cmap = runit(
    "avrpii_avrpi9",
    G,
    avrpii_df,
    enriched,
    unenriched,
)
```

```
markov
```

```
greedy
```

```
markov mst
```

```
greedy mst
```

```
there are 1 alignments. Each with 55 sequences.
# hmmbuild :: profile HMM construction from multiple sequence alignments
# HMMER 3.3 (Nov 2019); http://hmmer.org/
# Copyright (C) 2019 Howard Hughes Medical Institute.
# Freely distributed under the BSD open source license.
# - - - - - - - - - - - - - - - - - - - - - - - - - - - - - - - - - - - -
# input alignment file:             /tmp/tmpdokgrd6x
# output HMM file:                  /tmp/tmpo0mjpxua
# name (the single) HMM:            avrpii_avrpi9
# input alignment is asserted as:   protein
# - - - - - - - - - - - - - - - - - - - - - - - - - - - - - - - - - - - -

# idx name                  nseq  alen  mlen eff_nseq re/pos description
#---- -------------------- ----- ----- ----- -------- ------ -----------
1     avrpii_avrpi9           55   176   107     2.88  0.590 

# CPU time: 0.05u 0.00s 00:00:00.05 Elapsed: 00:00:00.05
```

In [72]:

```
get_plots(
    "avrpii_avrpi9",
    0,
    avrpii_hmms,
    avrpii_msas,
    avrpii_unfiltered_dfs,
    avrpii_filtered_dfs,
    avrpii_markov_cmap,
    avrpii_greedy_cmap,
    run_fastree=True,
    show=True,
)
```

```
# hmmbuild :: profile HMM construction from multiple sequence alignments
# HMMER 3.3 (Nov 2019); http://hmmer.org/
# Copyright (C) 2019 Howard Hughes Medical Institute.
# Freely distributed under the BSD open source license.
# - - - - - - - - - - - - - - - - - - - - - - - - - - - - - - - - - - - -
# input alignment file:             /tmp/tmp_d5xdpg5
# output HMM file:                  /tmp/tmpyy9_bu41
# name (the single) HMM:            avrpii_avrpi9
# input alignment is asserted as:   protein
# - - - - - - - - - - - - - - - - - - - - - - - - - - - - - - - - - - - -

# idx name                  nseq  alen  mlen eff_nseq re/pos description
#---- -------------------- ----- ----- ----- -------- ------ -----------
1     avrpii_avrpi9           68   246   118     6.35  0.590 

# CPU time: 0.05u 0.00s 00:00:00.05 Elapsed: 00:00:00.05


avrpii_avrpi9-unfiltered_logo
```

```
avrpii_avrpi9-unfiltered_logo-maxgap0.5
```

```
avrpii_avrpi9-filtered_logo
```

```
avrpii_avrpi9-filtered_logo-maxgap0.5
```

```
avrpii_avrpi9-filtered_logo_group
```

```
avrpii_avrpi9-filtered_logo_group-maxgap0.5
```

```
avrpii_avrpi9-filtered_logo_group-maxgap0.5minsize10
```

```
avrpii_avrpi9-unfiltered_logo_group
```

```
avrpii_avrpi9-unfiltered_logo_group-maxgap0.5
```

```
avrpii_avrpi9-unfiltered_logo_group-maxgap0.5minsize10
```

```
avrpii_avrpi9-unfiltered_markov
```

```
avrpii_avrpi9-unfiltered_greedy
```

```
avrpii_avrpi9-filtered_markov
```

```
avrpii_avrpi9-filtered_greedy
```

```
avrpii_avrpi9-unfiltered_markov-maxgap0.5
```

```
avrpii_avrpi9-unfiltered_greedy-maxgap0.5
```

```
avrpii_avrpi9-filtered_markov-maxgap0.5
```

```
avrpii_avrpi9-filtered_greedy-maxgap0.5
```

In [73]:

```
ecp2_df = eff_with_unique[
    eff_with_unique["supercluster_markov"]
    .isin(eff_with_unique.loc[
        eff_with_unique["effectors"].isin(['CfEcp2']), "supercluster_markov"]) | \
    eff_with_unique["supercluster_greedy"]
    .isin(eff_with_unique.loc[
        eff_with_unique["effectors"].isin(['CfEcp2']), "supercluster_greedy"])
]
ecp2_df.head()
```

Out[73]:

|  | supercluster\_component | supercluster\_markov | supercluster\_greedy | cluster | effectors | phibase | organisms | lca\_rank | lca | cluster\_size | cluster\_length | member |
| --- | --- | --- | --- | --- | --- | --- | --- | --- | --- | --- | --- | --- |
| 567631 | PC\_06FCV2 | PC\_008GAD | PC\_02URNV | PC\_008GAD | NaN | NaN | Valsa malicola;Valsa sordida;Valsa mali var. p... | family | Valsaceae | 10 | 178 | PC\_008GAD |
| 567632 | PC\_06FCV2 | PC\_008GAD | PC\_02URNV | PC\_008GAD | NaN | NaN | Valsa malicola;Valsa sordida;Valsa mali var. p... | family | Valsaceae | 10 | 178 | PC\_044J4G |
| 567633 | PC\_06FCV2 | PC\_008GAD | PC\_02URNV | PC\_008GAD | NaN | NaN | Valsa malicola;Valsa sordida;Valsa mali var. p... | family | Valsaceae | 10 | 178 | PC\_00PCCG |
| 567634 | PC\_06FCV2 | PC\_008GAD | PC\_02URNV | PC\_008GAD | NaN | NaN | Valsa malicola;Valsa sordida;Valsa mali var. p... | family | Valsaceae | 10 | 178 | PC\_05H9KD |
| 567635 | PC\_06FCV2 | PC\_008GAD | PC\_02URNV | PC\_008GAD | NaN | NaN | Valsa malicola;Valsa sordida;Valsa mali var. p... | family | Valsaceae | 10 | 178 | PC\_063Z99 |

In [74]:

```
ecp2_hmms, ecp2_msas, ecp2_unfiltered_dfs, ecp2_filtered_dfs, ecp2_markov_cmap, ecp2_greedy_cmap = runit(
    "ecp2_ztnip1",
    G,
    ecp2_df,
    enriched,
    unenriched,
)
```

```
markov
```

```
greedy
```

```
markov mst
```

```
greedy mst
```

```
there are 1 alignments. Each with 160 sequences.
# hmmbuild :: profile HMM construction from multiple sequence alignments
# HMMER 3.3 (Nov 2019); http://hmmer.org/
# Copyright (C) 2019 Howard Hughes Medical Institute.
# Freely distributed under the BSD open source license.
# - - - - - - - - - - - - - - - - - - - - - - - - - - - - - - - - - - - -
# input alignment file:             /tmp/tmp61eeq74n
# output HMM file:                  /tmp/tmp5g0kf18h
# name (the single) HMM:            ecp2_ztnip1
# input alignment is asserted as:   protein
# - - - - - - - - - - - - - - - - - - - - - - - - - - - - - - - - - - - -

# idx name                  nseq  alen  mlen eff_nseq re/pos description
#---- -------------------- ----- ----- ----- -------- ------ -----------
1     ecp2_ztnip1            160   401   165     3.60  0.590 

# CPU time: 0.07u 0.00s 00:00:00.07 Elapsed: 00:00:00.08
```

In [75]:

```
get_plots(
    "ecp2_ztnip1",
    0,
    ecp2_hmms,
    ecp2_msas,
    ecp2_unfiltered_dfs,
    ecp2_filtered_dfs,
    ecp2_markov_cmap,
    ecp2_greedy_cmap,
    run_fastree=True,
    show=True,
)
```

```
# hmmbuild :: profile HMM construction from multiple sequence alignments
# HMMER 3.3 (Nov 2019); http://hmmer.org/
# Copyright (C) 2019 Howard Hughes Medical Institute.
# Freely distributed under the BSD open source license.
# - - - - - - - - - - - - - - - - - - - - - - - - - - - - - - - - - - - -
# input alignment file:             /tmp/tmp5ge1fjvj
# output HMM file:                  /tmp/tmpjyewc46x
# name (the single) HMM:            ecp2_ztnip1
# input alignment is asserted as:   protein
# - - - - - - - - - - - - - - - - - - - - - - - - - - - - - - - - - - - -

# idx name                  nseq  alen  mlen eff_nseq re/pos description
#---- -------------------- ----- ----- ----- -------- ------ -----------
1     ecp2_ztnip1            245   510   261    12.01  0.590 

# CPU time: 0.13u 0.00s 00:00:00.13 Elapsed: 00:00:00.13


ecp2_ztnip1-unfiltered_logo
```

```
ecp2_ztnip1-unfiltered_logo-maxgap0.5
```

```
ecp2_ztnip1-filtered_logo
```

```
ecp2_ztnip1-filtered_logo-maxgap0.5
```

```
ecp2_ztnip1-filtered_logo_group
```

```
ecp2_ztnip1-filtered_logo_group-maxgap0.5
```

```
ecp2_ztnip1-filtered_logo_group-maxgap0.5minsize10
```

```
ecp2_ztnip1-unfiltered_logo_group
```

```
ecp2_ztnip1-unfiltered_logo_group-maxgap0.5
```

```
ecp2_ztnip1-unfiltered_logo_group-maxgap0.5minsize10
```

```
ecp2_ztnip1-unfiltered_markov
```

```
ecp2_ztnip1-unfiltered_greedy
```

```
ecp2_ztnip1-filtered_markov
```

```
ecp2_ztnip1-filtered_greedy
```

```
ecp2_ztnip1-unfiltered_markov-maxgap0.5
```

```
ecp2_ztnip1-unfiltered_greedy-maxgap0.5
```

```
ecp2_ztnip1-filtered_markov-maxgap0.5
```

```
ecp2_ztnip1-filtered_greedy-maxgap0.5
```

In [76]:

```
ave1_df = eff_with_unique[
    eff_with_unique["supercluster_markov"]
    .isin(eff_with_unique.loc[
        eff_with_unique["effectors"].isin(['VdAve1', 'MoMSP1']), "supercluster_markov"]) | \
    eff_with_unique["supercluster_greedy"]
    .isin(eff_with_unique.loc[
        eff_with_unique["effectors"].isin(['VdAve1', 'MoMSP1']), "supercluster_greedy"])
]
ave1_df.head()
```

Out[76]:

|  | supercluster\_component | supercluster\_markov | supercluster\_greedy | cluster | effectors | phibase | organisms | lca\_rank | lca | cluster\_size | cluster\_length | member |
| --- | --- | --- | --- | --- | --- | --- | --- | --- | --- | --- | --- | --- |
| 573202 | PC\_06FCV2 | PC\_009YZN | PC\_029W2K | PC\_04MIWA | NaN | NaN | Amanita muscaria Koide BX008 | strain | Amanita muscaria Koide BX008 | 1 | 158 | PC\_04MIWA |
| 729477 | PC\_06FCV2 | PC\_00VO2T | PC\_00FID0 | PC\_00FID0 | NaN | NaN | Fusarium sp. FIESC\_5 CS3069 | strain | Fusarium sp. FIESC\_5 CS3069 | 1 | 127 | PC\_00FID0 |
| 729478 | PC\_06FCV2 | PC\_00VO2T | PC\_00FID0 | PC\_04UG3A | NaN | NaN | Fusarium oxysporum f. sp. vasinfectum 25433;Fu... | species | Fusarium oxysporum | 4 | 118 | PC\_04UG3A |
| 729479 | PC\_06FCV2 | PC\_00VO2T | PC\_00FID0 | PC\_04UG3A | NaN | NaN | Fusarium oxysporum f. sp. vasinfectum 25433;Fu... | species | Fusarium oxysporum | 4 | 118 | PC\_05PDU3 |
| 729480 | PC\_06FCV2 | PC\_00VO2T | PC\_00FID0 | PC\_04UG3A | NaN | NaN | Fusarium oxysporum f. sp. vasinfectum 25433;Fu... | species | Fusarium oxysporum | 4 | 118 | PC\_02O22R |

In [77]:

```
ave1_hmms, ave1_msas, ave1_unfiltered_dfs, ave1_filtered_dfs, ave1_markov_cmap, ave1_greedy_cmap = runit(
    "ave1_momsp1",
    G,
    ave1_df,
    enriched,
    unenriched,
)
```

```
markov
```

```
greedy
```

```
markov mst
```

```
greedy mst
```

```
there are 2 alignments. Each with 120, 115 sequences.
# hmmbuild :: profile HMM construction from multiple sequence alignments
# HMMER 3.3 (Nov 2019); http://hmmer.org/
# Copyright (C) 2019 Howard Hughes Medical Institute.
# Freely distributed under the BSD open source license.
# - - - - - - - - - - - - - - - - - - - - - - - - - - - - - - - - - - - -
# input alignment file:             /tmp/tmpmgwpw2xg
# output HMM file:                  /tmp/tmpwcxcwqh_
# name (the single) HMM:            ave1_momsp1
# input alignment is asserted as:   protein
# - - - - - - - - - - - - - - - - - - - - - - - - - - - - - - - - - - - -

# idx name                  nseq  alen  mlen eff_nseq re/pos description
#---- -------------------- ----- ----- ----- -------- ------ -----------
1     ave1_momsp1            120   257   151     4.45  0.590 

# CPU time: 0.07u 0.00s 00:00:00.07 Elapsed: 00:00:00.07


# hmmbuild :: profile HMM construction from multiple sequence alignments
# HMMER 3.3 (Nov 2019); http://hmmer.org/
# Copyright (C) 2019 Howard Hughes Medical Institute.
# Freely distributed under the BSD open source license.
# - - - - - - - - - - - - - - - - - - - - - - - - - - - - - - - - - - - -
# input alignment file:             /tmp/tmpzse2rir2
# output HMM file:                  /tmp/tmppcc0shop
# name (the single) HMM:            ave1_momsp1
# input alignment is asserted as:   protein
# - - - - - - - - - - - - - - - - - - - - - - - - - - - - - - - - - - - -

# idx name                  nseq  alen  mlen eff_nseq re/pos description
#---- -------------------- ----- ----- ----- -------- ------ -----------
1     ave1_momsp1            115   167   146     1.79  0.589 

# CPU time: 0.07u 0.00s 00:00:00.07 Elapsed: 00:00:00.07
```

It doesn't look like we can merge msp and ave1.

In [78]:

```
ave1_df = eff_with_unique[
    eff_with_unique["supercluster_markov"]
    .isin(eff_with_unique.loc[
        eff_with_unique["effectors"].isin(['VdAve1']), "supercluster_markov"]) | \
    eff_with_unique["supercluster_greedy"]
    .isin(eff_with_unique.loc[
        eff_with_unique["effectors"].isin(['VdAve1']), "supercluster_greedy"])
]
ave1_df.head()
```

Out[78]:

|  | supercluster\_component | supercluster\_markov | supercluster\_greedy | cluster | effectors | phibase | organisms | lca\_rank | lca | cluster\_size | cluster\_length | member |
| --- | --- | --- | --- | --- | --- | --- | --- | --- | --- | --- | --- | --- |
| 573202 | PC\_06FCV2 | PC\_009YZN | PC\_029W2K | PC\_04MIWA | NaN | NaN | Amanita muscaria Koide BX008 | strain | Amanita muscaria Koide BX008 | 1 | 158 | PC\_04MIWA |
| 1096826 | PC\_06FCV2 | PC\_029W2K | PC\_004RGQ | PC\_03STYR | NaN | NaN | Phellinidium pouzarii | species | Phellinidium pouzarii | 1 | 144 | PC\_03STYR |
| 1096827 | PC\_06FCV2 | PC\_029W2K | PC\_006USE | PC\_006USE | NaN | NaN | Acaromyces ingoldii | species | Acaromyces ingoldii | 1 | 136 | PC\_006USE |
| 1096828 | PC\_06FCV2 | PC\_029W2K | PC\_006USE | PC\_03FE05 | NaN | NaN | Zymoseptoria tritici ST99CH\_1A5 | strain | Zymoseptoria tritici ST99CH\_1A5 | 1 | 143 | PC\_03FE05 |
| 1096829 | PC\_06FCV2 | PC\_029W2K | PC\_006USE | PC\_03SKZH | NaN | NaN | Colletotrichum fructicola Nara gc5 | strain | Colletotrichum fructicola Nara gc5 | 1 | 165 | PC\_03SKZH |

In [106]:

```
ave1_hmms, ave1_msas, ave1_unfiltered_dfs, ave1_filtered_dfs, ave1_markov_cmap, ave1_greedy_cmap = runit(
    "ave1",
    G,
    ave1_df,
    enriched,
    unenriched,
)
```

```
markov
```

```
greedy
```

```
markov mst
```

```
greedy mst
```

```
there are 1 alignments. Each with 120 sequences.
# hmmbuild :: profile HMM construction from multiple sequence alignments
# HMMER 3.3 (Nov 2019); http://hmmer.org/
# Copyright (C) 2019 Howard Hughes Medical Institute.
# Freely distributed under the BSD open source license.
# - - - - - - - - - - - - - - - - - - - - - - - - - - - - - - - - - - - -
# input alignment file:             /tmp/tmpdugme7w7
# output HMM file:                  /tmp/tmpvyi9hd7_
# name (the single) HMM:            ave1
# input alignment is asserted as:   protein
# - - - - - - - - - - - - - - - - - - - - - - - - - - - - - - - - - - - -

# idx name                  nseq  alen  mlen eff_nseq re/pos description
#---- -------------------- ----- ----- ----- -------- ------ -----------
1     ave1                   120   257   151     4.45  0.590 

# CPU time: 0.07u 0.00s 00:00:00.07 Elapsed: 00:00:00.07
```

In [107]:

```
get_plots(
    "ave1",
    0,
    ave1_hmms,
    ave1_msas,
    ave1_unfiltered_dfs,
    ave1_filtered_dfs,
    ave1_markov_cmap,
    ave1_greedy_cmap,
    run_fastree=True,
    show=True,
)
```

```
# hmmbuild :: profile HMM construction from multiple sequence alignments
# HMMER 3.3 (Nov 2019); http://hmmer.org/
# Copyright (C) 2019 Howard Hughes Medical Institute.
# Freely distributed under the BSD open source license.
# - - - - - - - - - - - - - - - - - - - - - - - - - - - - - - - - - - - -
# input alignment file:             /tmp/tmpjg3kt1mj
# output HMM file:                  /tmp/tmpe78cmo7d
# name (the single) HMM:            ave1
# input alignment is asserted as:   protein
# - - - - - - - - - - - - - - - - - - - - - - - - - - - - - - - - - - - -

# idx name                  nseq  alen  mlen eff_nseq re/pos description
#---- -------------------- ----- ----- ----- -------- ------ -----------
1     ave1                  1779   851   302    10.48  0.590 

# CPU time: 0.21u 0.01s 00:00:00.22 Elapsed: 00:00:00.22


ave1-unfiltered_logo
```

```
ave1-unfiltered_logo-maxgap0.5
```

```
ave1-filtered_logo
```

```
ave1-filtered_logo-maxgap0.5
```

```
ave1-filtered_logo_group
```

```
ave1-filtered_logo_group-maxgap0.5
```

```
ave1-filtered_logo_group-maxgap0.5minsize10
```

```
ave1-unfiltered_logo_group
```

```
ave1-unfiltered_logo_group-maxgap0.5
```

```
ave1-unfiltered_logo_group-maxgap0.5minsize10
```

```
ave1-unfiltered_markov
```

```
---------------------------------------------------------------------------
ValueError                                Traceback (most recent call last)
<ipython-input-107-b212e0aa52c8> in <module>
      9     ave1_greedy_cmap,
     10     run_fastree=True,
---> 11     show=True,
     12 )

<ipython-input-45-faf363bbac27> in get_plots(name, index, hmms, msas, unfiltered_dfs, filtered_dfs, markov_cmap, greedy_cmap, outdir, palette, logo_palette, run_fastree, show)
     86         max_gap=1,
     87         show=show,
---> 88         outdir=outdir,
     89     )
     90 

<ipython-input-44-2c72ccb9f635> in alnlogo(name, df, greedy_cmap, markov_cmap, alignment_col, max_gap, cpalette, logo_palette, base_width, width_per_base, base_height, height_per_seq, hratio, wratio, outdir, show)
    166 
    167     fig.savefig(os.path.join(outdir, f"{name}-alignment_logo.svg"))
--> 168     fig.savefig(os.path.join(outdir, f"{name}-alignment_logo.png"), dpi=600)
    169 
    170     if show:

~/.miniconda/envs/remote_homology/lib/python3.7/site-packages/matplotlib/figure.py in savefig(self, fname, transparent, **kwargs)
   2201             self.patch.set_visible(frameon)
   2202 
-> 2203         self.canvas.print_figure(fname, **kwargs)
   2204 
   2205         if frameon:

~/.miniconda/envs/remote_homology/lib/python3.7/site-packages/matplotlib/backend_bases.py in print_figure(self, filename, dpi, facecolor, edgecolor, orientation, format, bbox_inches, **kwargs)
   2124                     orientation=orientation,
   2125                     bbox_inches_restore=_bbox_inches_restore,
-> 2126                     **kwargs)
   2127             finally:
   2128                 if bbox_inches and restore_bbox:

~/.miniconda/envs/remote_homology/lib/python3.7/site-packages/matplotlib/backends/backend_agg.py in print_png(self, filename_or_obj, metadata, pil_kwargs, *args, **kwargs)
    512         }
    513 
--> 514         FigureCanvasAgg.draw(self)
    515         if pil_kwargs is not None:
    516             from PIL import Image

~/.miniconda/envs/remote_homology/lib/python3.7/site-packages/matplotlib/backends/backend_agg.py in draw(self)
    386         Draw the figure using the renderer.
    387         """
--> 388         self.renderer = self.get_renderer(cleared=True)
    389         # Acquire a lock on the shared font cache.
    390         with RendererAgg.lock, \

~/.miniconda/envs/remote_homology/lib/python3.7/site-packages/matplotlib/backends/backend_agg.py in get_renderer(self, cleared)
    402                           and getattr(self, "_lastKey", None) == key)
    403         if not reuse_renderer:
--> 404             self.renderer = RendererAgg(w, h, self.figure.dpi)
    405             self._lastKey = key
    406         elif cleared:

~/.miniconda/envs/remote_homology/lib/python3.7/site-packages/matplotlib/backends/backend_agg.py in __init__(self, width, height, dpi)
     90         self.width = width
     91         self.height = height
---> 92         self._renderer = _RendererAgg(int(width), int(height), dpi)
     93         self._filter_renderers = []
     94 

ValueError: Image size of 87779x16212 pixels is too large. It must be less than 2^16 in each direction.
```

In [108]:

```
alnlogo(
    "figure-ave1-filtered-gt0.5",
    ave1_filtered_dfs[0],
    ave1_greedy_cmap,
    ave1_markov_cmap,
    max_gap=0.5,
    outdir=OUTDIR,
    width_per_base=0.05,
    height_per_seq=0.005,
    wratio=[0.015, 0.015, 0.015, 0.015, 1],
    hratio=[0.06, 1]
)
```

In [109]:

```
alnlogo(
    "figure-ave1-filtered",
    ave1_filtered_dfs[0],
    ave1_greedy_cmap,
    ave1_markov_cmap,
    max_gap=1,
    outdir=OUTDIR,
    width_per_base=0.05,
    height_per_seq=0.005,
    wratio=[0.01, 0.01, 0.01, 0.01, 1],
    hratio=[0.06, 1]
)
```

In [ ]:

In [110]:

```
msp1_df = eff_with_unique[
    eff_with_unique["supercluster_markov"]
    .isin(eff_with_unique.loc[
        eff_with_unique["effectors"].isin(['MoMSP1']), "supercluster_markov"]) | \
    eff_with_unique["supercluster_greedy"]
    .isin(eff_with_unique.loc[
        eff_with_unique["effectors"].isin(['MoMSP1']), "supercluster_greedy"])
]
msp1_df.head()
```

Out[110]:

|  | supercluster\_component | supercluster\_markov | supercluster\_greedy | cluster | effectors | phibase | organisms | lca\_rank | lca | cluster\_size | cluster\_length | member |
| --- | --- | --- | --- | --- | --- | --- | --- | --- | --- | --- | --- | --- |
| 729477 | PC\_06FCV2 | PC\_00VO2T | PC\_00FID0 | PC\_00FID0 | NaN | NaN | Fusarium sp. FIESC\_5 CS3069 | strain | Fusarium sp. FIESC\_5 CS3069 | 1 | 127 | PC\_00FID0 |
| 729478 | PC\_06FCV2 | PC\_00VO2T | PC\_00FID0 | PC\_04UG3A | NaN | NaN | Fusarium oxysporum f. sp. vasinfectum 25433;Fu... | species | Fusarium oxysporum | 4 | 118 | PC\_04UG3A |
| 729479 | PC\_06FCV2 | PC\_00VO2T | PC\_00FID0 | PC\_04UG3A | NaN | NaN | Fusarium oxysporum f. sp. vasinfectum 25433;Fu... | species | Fusarium oxysporum | 4 | 118 | PC\_05PDU3 |
| 729480 | PC\_06FCV2 | PC\_00VO2T | PC\_00FID0 | PC\_04UG3A | NaN | NaN | Fusarium oxysporum f. sp. vasinfectum 25433;Fu... | species | Fusarium oxysporum | 4 | 118 | PC\_02O22R |
| 729481 | PC\_06FCV2 | PC\_00VO2T | PC\_00FID0 | PC\_04UG3A | NaN | NaN | Fusarium oxysporum f. sp. vasinfectum 25433;Fu... | species | Fusarium oxysporum | 4 | 118 | PC\_06LEW1 |

In [111]:

```
msp1_hmms, msp1_msas, msp1_unfiltered_dfs, msp1_filtered_dfs, msp1_markov_cmap, msp1_greedy_cmap = runit(
    "momsp1",
    G,
    msp1_df,
    enriched,
    unenriched,
)
```

```
markov
```

```
greedy
```

```
markov mst
```

```
greedy mst
```

```
there are 1 alignments. Each with 115 sequences.
# hmmbuild :: profile HMM construction from multiple sequence alignments
# HMMER 3.3 (Nov 2019); http://hmmer.org/
# Copyright (C) 2019 Howard Hughes Medical Institute.
# Freely distributed under the BSD open source license.
# - - - - - - - - - - - - - - - - - - - - - - - - - - - - - - - - - - - -
# input alignment file:             /tmp/tmpp9zls2dg
# output HMM file:                  /tmp/tmp60x25_hi
# name (the single) HMM:            momsp1
# input alignment is asserted as:   protein
# - - - - - - - - - - - - - - - - - - - - - - - - - - - - - - - - - - - -

# idx name                  nseq  alen  mlen eff_nseq re/pos description
#---- -------------------- ----- ----- ----- -------- ------ -----------
1     momsp1                 115   167   146     1.79  0.589 

# CPU time: 0.07u 0.00s 00:00:00.07 Elapsed: 00:00:00.07
```

In [112]:

```
get_plots(
    "momsp1",
    0,
    msp1_hmms,
    msp1_msas,
    msp1_unfiltered_dfs,
    msp1_filtered_dfs,
    msp1_markov_cmap,
    msp1_greedy_cmap,
    run_fastree=True,
    show=True,
)
```

```
# hmmbuild :: profile HMM construction from multiple sequence alignments
# HMMER 3.3 (Nov 2019); http://hmmer.org/
# Copyright (C) 2019 Howard Hughes Medical Institute.
# Freely distributed under the BSD open source license.
# - - - - - - - - - - - - - - - - - - - - - - - - - - - - - - - - - - - -
# input alignment file:             /tmp/tmpwtwfue33
# output HMM file:                  /tmp/tmpifseyixr
# name (the single) HMM:            momsp1
# input alignment is asserted as:   protein
# - - - - - - - - - - - - - - - - - - - - - - - - - - - - - - - - - - - -

# idx name                  nseq  alen  mlen eff_nseq re/pos description
#---- -------------------- ----- ----- ----- -------- ------ -----------
1     momsp1                 891   698   324     8.72  0.590 

# CPU time: 0.19u 0.01s 00:00:00.20 Elapsed: 00:00:00.19


momsp1-unfiltered_logo
```

```
momsp1-unfiltered_logo-maxgap0.5
```

```
momsp1-filtered_logo
```

```
momsp1-filtered_logo-maxgap0.5
```

```
momsp1-filtered_logo_group
```

```
momsp1-filtered_logo_group-maxgap0.5
```

```
momsp1-filtered_logo_group-maxgap0.5minsize10
```

```
momsp1-unfiltered_logo_group
```

```
momsp1-unfiltered_logo_group-maxgap0.5
```

```
momsp1-unfiltered_logo_group-maxgap0.5minsize10
```

```
momsp1-unfiltered_markov
```

```
---------------------------------------------------------------------------
ValueError                                Traceback (most recent call last)
<ipython-input-112-1dd834c06c6e> in <module>
      9     msp1_greedy_cmap,
     10     run_fastree=True,
---> 11     show=True,
     12 )

<ipython-input-45-faf363bbac27> in get_plots(name, index, hmms, msas, unfiltered_dfs, filtered_dfs, markov_cmap, greedy_cmap, outdir, palette, logo_palette, run_fastree, show)
     86         max_gap=1,
     87         show=show,
---> 88         outdir=outdir,
     89     )
     90 

<ipython-input-44-2c72ccb9f635> in alnlogo(name, df, greedy_cmap, markov_cmap, alignment_col, max_gap, cpalette, logo_palette, base_width, width_per_base, base_height, height_per_seq, hratio, wratio, outdir, show)
    166 
    167     fig.savefig(os.path.join(outdir, f"{name}-alignment_logo.svg"))
--> 168     fig.savefig(os.path.join(outdir, f"{name}-alignment_logo.png"), dpi=600)
    169 
    170     if show:

~/.miniconda/envs/remote_homology/lib/python3.7/site-packages/matplotlib/figure.py in savefig(self, fname, transparent, **kwargs)
   2201             self.patch.set_visible(frameon)
   2202 
-> 2203         self.canvas.print_figure(fname, **kwargs)
   2204 
   2205         if frameon:

~/.miniconda/envs/remote_homology/lib/python3.7/site-packages/matplotlib/backend_bases.py in print_figure(self, filename, dpi, facecolor, edgecolor, orientation, format, bbox_inches, **kwargs)
   2124                     orientation=orientation,
   2125                     bbox_inches_restore=_bbox_inches_restore,
-> 2126                     **kwargs)
   2127             finally:
   2128                 if bbox_inches and restore_bbox:

~/.miniconda/envs/remote_homology/lib/python3.7/site-packages/matplotlib/backends/backend_agg.py in print_png(self, filename_or_obj, metadata, pil_kwargs, *args, **kwargs)
    512         }
    513 
--> 514         FigureCanvasAgg.draw(self)
    515         if pil_kwargs is not None:
    516             from PIL import Image

~/.miniconda/envs/remote_homology/lib/python3.7/site-packages/matplotlib/backends/backend_agg.py in draw(self)
    386         Draw the figure using the renderer.
    387         """
--> 388         self.renderer = self.get_renderer(cleared=True)
    389         # Acquire a lock on the shared font cache.
    390         with RendererAgg.lock, \

~/.miniconda/envs/remote_homology/lib/python3.7/site-packages/matplotlib/backends/backend_agg.py in get_renderer(self, cleared)
    402                           and getattr(self, "_lastKey", None) == key)
    403         if not reuse_renderer:
--> 404             self.renderer = RendererAgg(w, h, self.figure.dpi)
    405             self._lastKey = key
    406         elif cleared:

~/.miniconda/envs/remote_homology/lib/python3.7/site-packages/matplotlib/backends/backend_agg.py in __init__(self, width, height, dpi)
     90         self.width = width
     91         self.height = height
---> 92         self._renderer = _RendererAgg(int(width), int(height), dpi)
     93         self._filter_renderers = []
     94 

ValueError: Image size of 68520x9930 pixels is too large. It must be less than 2^16 in each direction.
```

In [113]:

```
alnlogo(
    "figure-momsp1-filtered-gt0.5",
    msp1_filtered_dfs[0],
    msp1_greedy_cmap,
    msp1_markov_cmap,
    max_gap=0.5,
    outdir=OUTDIR,
    width_per_base=0.05,
    height_per_seq=0.005,
    wratio=[0.015, 0.015, 0.015, 0.015, 1],
    hratio=[0.06, 1]
)
```

In [114]:

```
alnlogo(
    "figure-momsp1-filtered",
    msp1_filtered_dfs[0],
    msp1_greedy_cmap,
    msp1_markov_cmap,
    max_gap=1,
    outdir=OUTDIR,
    width_per_base=0.05,
    height_per_seq=0.005,
    wratio=[0.01, 0.01, 0.01, 0.01, 1],
    hratio=[0.06, 1]
)
```

In [ ]:

In [225]:

```
nep_df = eff_with_unique[eff_with_unique["supercluster_component"] == "PC_06S6A1"]
nep_df.head()
```

Out[225]:

|  | supercluster\_component | supercluster\_markov | supercluster\_greedy | cluster | effectors | phibase | organisms | lca\_rank | lca | cluster\_size | cluster\_length | member |
| --- | --- | --- | --- | --- | --- | --- | --- | --- | --- | --- | --- | --- |
| 2441438 | PC\_06S6A1 | PC\_00KWEY | PC\_007HTT | PC\_007HTT | NaN | NaN | Magnaporthiopsis poae ATCC 64411 | strain | Magnaporthiopsis poae ATCC 64411 | 1 | 138 | PC\_007HTT |
| 2441439 | PC\_06S6A1 | PC\_00KWEY | PC\_007HTT | PC\_07UWJJ | NaN | NaN | Epichloe bromicola | species | Epichloe bromicola | 1 | 147 | PC\_07UWJJ |
| 2441440 | PC\_06S6A1 | PC\_00KWEY | PC\_007HTT | PC\_08G70O | NaN | NaN | Alternaria gaisen | species | Alternaria gaisen | 1 | 122 | PC\_08G70O |
| 2441441 | PC\_06S6A1 | PC\_00KWEY | PC\_00KWEY | PC\_00KWEY | NaN | NaN | Botrytis sp. D10\_B\_F3I05;Botrytis sp. D10\_B\_F1... | subkingdom | Dikarya | 14 | 163 | PC\_00KWEY |
| 2441442 | PC\_06S6A1 | PC\_00KWEY | PC\_00KWEY | PC\_00KWEY | NaN | NaN | Botrytis sp. D10\_B\_F3I05;Botrytis sp. D10\_B\_F1... | subkingdom | Dikarya | 14 | 163 | PC\_06VMON |

In [227]:

```
nep_hmms, nep_msas, nep_unfiltered_dfs, nep_filtered_dfs, nep_markov_cmap, nep_greedy_cmap = runit(
    "nep",
    G,
    nep_df,
    enriched,
    unenriched,
)
```

```
markov
```

```
greedy
```

```
markov mst
```

```
greedy mst
```

```
there are 2 alignments. Each with 201, 166 sequences.
# hmmbuild :: profile HMM construction from multiple sequence alignments
# HMMER 3.3 (Nov 2019); http://hmmer.org/
# Copyright (C) 2019 Howard Hughes Medical Institute.
# Freely distributed under the BSD open source license.
# - - - - - - - - - - - - - - - - - - - - - - - - - - - - - - - - - - - -
# input alignment file:             /tmp/tmp6e_jhk5p
# output HMM file:                  /tmp/tmpc_fh7san
# name (the single) HMM:            nep
# input alignment is asserted as:   protein
# - - - - - - - - - - - - - - - - - - - - - - - - - - - - - - - - - - - -

# idx name                  nseq  alen  mlen eff_nseq re/pos description
#---- -------------------- ----- ----- ----- -------- ------ -----------
1     nep                    201   183   141     2.76  0.590 

# CPU time: 0.07u 0.00s 00:00:00.07 Elapsed: 00:00:00.07


# hmmbuild :: profile HMM construction from multiple sequence alignments
# HMMER 3.3 (Nov 2019); http://hmmer.org/
# Copyright (C) 2019 Howard Hughes Medical Institute.
# Freely distributed under the BSD open source license.
# - - - - - - - - - - - - - - - - - - - - - - - - - - - - - - - - - - - -
# input alignment file:             /tmp/tmpd8r1ibod
# output HMM file:                  /tmp/tmp_gfmmeis
# name (the single) HMM:            nep
# input alignment is asserted as:   protein
# - - - - - - - - - - - - - - - - - - - - - - - - - - - - - - - - - - - -

# idx name                  nseq  alen  mlen eff_nseq re/pos description
#---- -------------------- ----- ----- ----- -------- ------ -----------
1     nep                    166   437   336     3.25  0.590 

# CPU time: 0.16u 0.00s 00:00:00.16 Elapsed: 00:00:00.16
```

In [233]:

```
get_plots(
    "nep_1",
    0,
    nep_hmms,
    nep_msas,
    nep_unfiltered_dfs,
    nep_filtered_dfs,
    nep_markov_cmap,
    nep_greedy_cmap,
    run_fastree=False,
    show=True,
)
```

```
# hmmbuild :: profile HMM construction from multiple sequence alignments
# HMMER 3.3 (Nov 2019); http://hmmer.org/
# Copyright (C) 2019 Howard Hughes Medical Institute.
# Freely distributed under the BSD open source license.
# - - - - - - - - - - - - - - - - - - - - - - - - - - - - - - - - - - - -
# input alignment file:             /tmp/tmp6kevphv2
# output HMM file:                  /tmp/tmps73iby2d
# name (the single) HMM:            nep_1
# input alignment is asserted as:   protein
# - - - - - - - - - - - - - - - - - - - - - - - - - - - - - - - - - - - -

# idx name                  nseq  alen  mlen eff_nseq re/pos description
#---- -------------------- ----- ----- ----- -------- ------ -----------
1     nep_1                  735  1477   625    11.40  0.590 

# CPU time: 0.35u 0.01s 00:00:00.36 Elapsed: 00:00:00.36


nep_1-unfiltered_logo
```

```
nep_1-unfiltered_logo-maxgap0.5
```

```
nep_1-filtered_logo
```

```
nep_1-filtered_logo-maxgap0.5
```

```
nep_1-filtered_logo_group
```

```
nep_1-filtered_logo_group-maxgap0.5
```

```
nep_1-filtered_logo_group-maxgap0.5minsize10
```

```
nep_1-unfiltered_logo_group
```

```
nep_1-unfiltered_logo_group-maxgap0.5
```

```
nep_1-unfiltered_logo_group-maxgap0.5minsize10
```

```
nep_1-unfiltered_markov
```

```
---------------------------------------------------------------------------
ValueError                                Traceback (most recent call last)
<ipython-input-233-c498b2ab31c8> in <module>
      9     nep_greedy_cmap,
     10     run_fastree=False,
---> 11     show=True,
     12 )

<ipython-input-45-faf363bbac27> in get_plots(name, index, hmms, msas, unfiltered_dfs, filtered_dfs, markov_cmap, greedy_cmap, outdir, palette, logo_palette, run_fastree, show)
     86         max_gap=1,
     87         show=show,
---> 88         outdir=outdir,
     89     )
     90 

<ipython-input-232-6267afd8f8bc> in alnlogo(name, df, greedy_cmap, markov_cmap, alignment_col, max_gap, cpalette, logo_palette, base_width, width_per_base, base_height, height_per_seq, hratio, wratio, outdir, show)
    166 
    167     fig.savefig(os.path.join(outdir, f"{name}-alignment_logo.svg"))
--> 168     fig.savefig(os.path.join(outdir, f"{name}-alignment_logo.png"), dpi=450)
    169 
    170     if show:

~/.miniconda/envs/remote_homology/lib/python3.7/site-packages/matplotlib/figure.py in savefig(self, fname, transparent, **kwargs)
   2201             self.patch.set_visible(frameon)
   2202 
-> 2203         self.canvas.print_figure(fname, **kwargs)
   2204 
   2205         if frameon:

~/.miniconda/envs/remote_homology/lib/python3.7/site-packages/matplotlib/backend_bases.py in print_figure(self, filename, dpi, facecolor, edgecolor, orientation, format, bbox_inches, **kwargs)
   2124                     orientation=orientation,
   2125                     bbox_inches_restore=_bbox_inches_restore,
-> 2126                     **kwargs)
   2127             finally:
   2128                 if bbox_inches and restore_bbox:

~/.miniconda/envs/remote_homology/lib/python3.7/site-packages/matplotlib/backends/backend_agg.py in print_png(self, filename_or_obj, metadata, pil_kwargs, *args, **kwargs)
    512         }
    513 
--> 514         FigureCanvasAgg.draw(self)
    515         if pil_kwargs is not None:
    516             from PIL import Image

~/.miniconda/envs/remote_homology/lib/python3.7/site-packages/matplotlib/backends/backend_agg.py in draw(self)
    386         Draw the figure using the renderer.
    387         """
--> 388         self.renderer = self.get_renderer(cleared=True)
    389         # Acquire a lock on the shared font cache.
    390         with RendererAgg.lock, \

~/.miniconda/envs/remote_homology/lib/python3.7/site-packages/matplotlib/backends/backend_agg.py in get_renderer(self, cleared)
    402                           and getattr(self, "_lastKey", None) == key)
    403         if not reuse_renderer:
--> 404             self.renderer = RendererAgg(w, h, self.figure.dpi)
    405             self._lastKey = key
    406         elif cleared:

~/.miniconda/envs/remote_homology/lib/python3.7/site-packages/matplotlib/backends/backend_agg.py in __init__(self, width, height, dpi)
     90         self.width = width
     91         self.height = height
---> 92         self._renderer = _RendererAgg(int(width), int(height), dpi)
     93         self._filter_renderers = []
     94 

ValueError: Image size of 105929x8590 pixels is too large. It must be less than 2^16 in each direction.
```

In [ ]:

```
get_plots(
    "nep_2",
    1,
    nep_hmms,
    nep_msas,
    nep_unfiltered_dfs,
    nep_filtered_dfs,
    nep_markov_cmap,
    nep_greedy_cmap,
    run_fastree=False,
    show=True,
)
```

In [ ]:

In [ ]:

In [80]:

```
# PC_05OMCN is M.BR29.EuGene_00106461;M.TH16.EuGene_00040131;MoAVR_Pia
max_df = eff_with_unique[
    eff_with_unique["supercluster_component"]
    .isin(eff_with_unique.loc[
        eff_with_unique["effectors"].isin([
            'MoAvrPib', 'M.TH16.EuGene_00120731', 'MGG_16175',
            'M.TH16.EuGene_00134971', 'MoAVR_Pik', 'MoAvrPiz_t',
            'MGG_10120', 'MGG_18019', 'M.TH16.EuGene_00034081', 'MGG_14834',
            'M.BR29.EuGene_00119491;M.TH16.EuGene_00127871;MoAvr1_CO39',
            "M.BR29.EuGene_00126081;M.TH16.EuGene_00135161;PtrToxB",
        ]), "supercluster_component"]) |
    (eff_with_unique["cluster"] == "PC_05OMCN")
]
max_df.head()
```

Out[80]:

|  | supercluster\_component | supercluster\_markov | supercluster\_greedy | cluster | effectors | phibase | organisms | lca\_rank | lca | cluster\_size | cluster\_length | member |
| --- | --- | --- | --- | --- | --- | --- | --- | --- | --- | --- | --- | --- |
| 53100 | PC\_00ZF7V | PC\_00ZF7V | PC\_00ZF7V | PC\_00NUN6 | NaN | NaN | Colletotrichum gloeosporioides Cg-14 | strain | Colletotrichum gloeosporioides Cg-14 | 1 | 59 | PC\_00NUN6 |
| 53101 | PC\_00ZF7V | PC\_00ZF7V | PC\_00ZF7V | PC\_00ZF7V | NaN | NaN | Colletotrichum fructicola;Colletotrichum orbic... | clade | sordariomyceta | 22 | 87 | PC\_00ZF7V |
| 53102 | PC\_00ZF7V | PC\_00ZF7V | PC\_00ZF7V | PC\_00ZF7V | NaN | NaN | Colletotrichum fructicola;Colletotrichum orbic... | clade | sordariomyceta | 22 | 87 | PC\_048LF8 |
| 53103 | PC\_00ZF7V | PC\_00ZF7V | PC\_00ZF7V | PC\_00ZF7V | NaN | NaN | Colletotrichum fructicola;Colletotrichum orbic... | clade | sordariomyceta | 22 | 87 | PC\_030V50 |
| 53104 | PC\_00ZF7V | PC\_00ZF7V | PC\_00ZF7V | PC\_00ZF7V | NaN | NaN | Colletotrichum fructicola;Colletotrichum orbic... | clade | sordariomyceta | 22 | 87 | PC\_060C7X |

In [81]:

```
max_hmms, max_msas, max_unfiltered_dfs, max_filtered_dfs, max_markov_cmap, max_greedy_cmap = runit(
    "max",
    G,
    max_df,
    enriched,
    unenriched,
)
```

```
markov
```

```
greedy
```

```
markov mst
```

```
greedy mst
```

```
there are 2 alignments. Each with 70, 8 sequences.
# hmmbuild :: profile HMM construction from multiple sequence alignments
# HMMER 3.3 (Nov 2019); http://hmmer.org/
# Copyright (C) 2019 Howard Hughes Medical Institute.
# Freely distributed under the BSD open source license.
# - - - - - - - - - - - - - - - - - - - - - - - - - - - - - - - - - - - -
# input alignment file:             /tmp/tmpxiuu565p
# output HMM file:                  /tmp/tmpw_eq49e7
# name (the single) HMM:            max
# input alignment is asserted as:   protein
# - - - - - - - - - - - - - - - - - - - - - - - - - - - - - - - - - - - -

# idx name                  nseq  alen  mlen eff_nseq re/pos description
#---- -------------------- ----- ----- ----- -------- ------ -----------
1     max                     70   163    95     4.99  0.602 

# CPU time: 0.04u 0.00s 00:00:00.04 Elapsed: 00:00:00.04


# hmmbuild :: profile HMM construction from multiple sequence alignments
# HMMER 3.3 (Nov 2019); http://hmmer.org/
# Copyright (C) 2019 Howard Hughes Medical Institute.
# Freely distributed under the BSD open source license.
# - - - - - - - - - - - - - - - - - - - - - - - - - - - - - - - - - - - -
# input alignment file:             /tmp/tmp2uhvozu2
# output HMM file:                  /tmp/tmpd_ljuo02
# name (the single) HMM:            max
# input alignment is asserted as:   protein
# - - - - - - - - - - - - - - - - - - - - - - - - - - - - - - - - - - - -

# idx name                  nseq  alen  mlen eff_nseq re/pos description
#---- -------------------- ----- ----- ----- -------- ------ -----------
1     max                      8    82    75     1.31  0.752 

# CPU time: 0.03u 0.01s 00:00:00.04 Elapsed: 00:00:00.03
```

We don't seem to be able to merge all of the max groups.

Let's find out what the actual groups are.

In [ ]:

In [82]:

```
maxG = get_subgraph(G, max_df["cluster"].unique())
max_mst = gt.min_spanning_tree(maxG, weights=maxG.edge_properties["neg"])
max_cluster_msas = {c: enriched[c.encode()][:-1] for c in max_df["cluster"].unique()}

max_prog_msas = progressive(maxG, max_mst, max_cluster_msas, verbose=False)
```

```
there are 3 alignments. Each with 70, 21, 13 sequences.
```

In [83]:

```
max_cluster_ids = []

for i, cluster in enumerate(max_prog_msas):
    this = []
    seqs = SeqIO.parse(StringIO(cluster.decode()), "fasta")
    for seq in seqs:
        if seq.id.startswith("PC_") and seq.id.endswith("_consensus"):
            this.append(seq.id[:-len("_consensus")])
        elif seq.id.startswith("PC_"):
            this.append(seq.id)
    max_cluster_ids.append(this)
    
    this_comps = max_df.loc[max_df["member"].isin(set(this)), "supercluster_component"].unique()
    this_effectors = max_df.loc[max_df["member"].isin(set(this)), "effectors"].unique()
    
    print("Cluster", i, "components:", this_comps, ", effectors:", this_effectors)
```

```
Cluster 0 components: ['PC_00ZF7V' 'PC_01DKY2' 'PC_022BD9' 'PC_02W1ZZ' 'PC_07OBWM' 'PC_08JMY0'
 'PC_08L1R9'] , effectors: [nan 'M.TH16.EuGene_00106621;M.BR29.EuGene_00085071'
 'M.BR29.EuGene_00126081;M.TH16.EuGene_00135161;PtrToxB'
 'M.BR29.EuGene_00119491;M.TH16.EuGene_00127871;MoAvr1_CO39' 'MoAVR_Pik'
 'MoAvrPiz_t' 'MGG_18019' 'M.BR29.EuGene_00082031'
 'M.TH16.EuGene_00120731' 'MGG_14793' 'M.BR29.EuGene_00004921'
 'MGG_18060;M.BR29.EuGene_00087671' 'MoAvrPib' 'M.BR29.EuGene_00091361']
Cluster 1 components: ['PC_02LJVJ' 'PC_02O8L1' 'PC_049RVC' 'PC_05VEPP'] , effectors: [nan 'M.TH16.EuGene_00034081' 'M.TH16.EuGene_00134971' 'MGG_10120']
Cluster 2 components: [nan] , effectors: ['M.BR29.EuGene_00106461;M.TH16.EuGene_00040131;MoAVR_Pia']
```

It seems like we have 3 clusters with bona-fide effectors in them, so I'll try them separately and see what's up.

First up is ToxB

In [84]:

```
toxb_avr1co39_avrpik_avrpib_avrpia_df = eff_with_unique[
    eff_with_unique["supercluster_component"].isin([
        'PC_00ZF7V', 'PC_01DKY2', 'PC_022BD9', 'PC_02LJVJ', 'PC_02O8L1',
        'PC_05VEPP', 'PC_07OBWM', 'PC_08JJK6', 'PC_08JMY0', 'PC_08L1R9']) | \
    (eff_with_unique["cluster"] == "PC_05OMCN")
]
toxb_avr1co39_avrpik_avrpib_avrpia_df.head()
```

Out[84]:

|  | supercluster\_component | supercluster\_markov | supercluster\_greedy | cluster | effectors | phibase | organisms | lca\_rank | lca | cluster\_size | cluster\_length | member |
| --- | --- | --- | --- | --- | --- | --- | --- | --- | --- | --- | --- | --- |
| 53100 | PC\_00ZF7V | PC\_00ZF7V | PC\_00ZF7V | PC\_00NUN6 | NaN | NaN | Colletotrichum gloeosporioides Cg-14 | strain | Colletotrichum gloeosporioides Cg-14 | 1 | 59 | PC\_00NUN6 |
| 53101 | PC\_00ZF7V | PC\_00ZF7V | PC\_00ZF7V | PC\_00ZF7V | NaN | NaN | Colletotrichum fructicola;Colletotrichum orbic... | clade | sordariomyceta | 22 | 87 | PC\_00ZF7V |
| 53102 | PC\_00ZF7V | PC\_00ZF7V | PC\_00ZF7V | PC\_00ZF7V | NaN | NaN | Colletotrichum fructicola;Colletotrichum orbic... | clade | sordariomyceta | 22 | 87 | PC\_048LF8 |
| 53103 | PC\_00ZF7V | PC\_00ZF7V | PC\_00ZF7V | PC\_00ZF7V | NaN | NaN | Colletotrichum fructicola;Colletotrichum orbic... | clade | sordariomyceta | 22 | 87 | PC\_030V50 |
| 53104 | PC\_00ZF7V | PC\_00ZF7V | PC\_00ZF7V | PC\_00ZF7V | NaN | NaN | Colletotrichum fructicola;Colletotrichum orbic... | clade | sordariomyceta | 22 | 87 | PC\_060C7X |

In [86]:

```
toxb_hmms, toxb_msas, toxb_unfiltered_dfs, toxb_filtered_dfs, toxb_markov_cmap, toxb_greedy_cmap = runit(
    "toxb_avr1co39_avrpik_avrpib_avrpia",
    G,
    toxb_avr1co39_avrpik_avrpib_avrpia_df,
    enriched,
    unenriched,
)
```

```
markov
```

```
greedy
```

```
markov mst
```

```
greedy mst
```

```
there are 1 alignments. Each with 110 sequences.
# hmmbuild :: profile HMM construction from multiple sequence alignments
# HMMER 3.3 (Nov 2019); http://hmmer.org/
# Copyright (C) 2019 Howard Hughes Medical Institute.
# Freely distributed under the BSD open source license.
# - - - - - - - - - - - - - - - - - - - - - - - - - - - - - - - - - - - -
# input alignment file:             /tmp/tmpaljtvy0j
# output HMM file:                  /tmp/tmpxsztj3lq
# name (the single) HMM:            toxb_avr1co39_avrpik_avrpib_avrpia
# input alignment is asserted as:   protein
# - - - - - - - - - - - - - - - - - - - - - - - - - - - - - - - - - - - -

# idx name                  nseq  alen  mlen eff_nseq re/pos description
#---- -------------------- ----- ----- ----- -------- ------ -----------
1     toxb_avr1co39_avrpik_avrpib_avrpia   110   190    84     8.48  0.676 

# CPU time: 0.04u 0.00s 00:00:00.04 Elapsed: 00:00:00.04
```

In [138]:

```
get_plots(
    "toxb_avr1co39_avrpik_avrpib_avrpia",
    0,
    toxb_hmms,
    toxb_msas,
    toxb_unfiltered_dfs,
    toxb_filtered_dfs,
    toxb_markov_cmap,
    toxb_greedy_cmap,
    run_fastree=True,
    show=True,
)
```

```
# hmmbuild :: profile HMM construction from multiple sequence alignments
# HMMER 3.3 (Nov 2019); http://hmmer.org/
# Copyright (C) 2019 Howard Hughes Medical Institute.
# Freely distributed under the BSD open source license.
# - - - - - - - - - - - - - - - - - - - - - - - - - - - - - - - - - - - -
# input alignment file:             /tmp/tmph8gvv1vn
# output HMM file:                  /tmp/tmp8nh3zfmi
# name (the single) HMM:            toxb_avr1co39_avrpik_avrpib_avrpia
# input alignment is asserted as:   protein
# - - - - - - - - - - - - - - - - - - - - - - - - - - - - - - - - - - - -

# idx name                  nseq  alen  mlen eff_nseq re/pos description
#---- -------------------- ----- ----- ----- -------- ------ -----------
1     toxb_avr1co39_avrpik_avrpib_avrpia   107   303   179    11.88  0.590 

# CPU time: 0.08u 0.00s 00:00:00.08 Elapsed: 00:00:00.08


toxb_avr1co39_avrpik_avrpib_avrpia-unfiltered_logo
```

```
toxb_avr1co39_avrpik_avrpib_avrpia-unfiltered_logo-maxgap0.5
```

```
toxb_avr1co39_avrpik_avrpib_avrpia-filtered_logo
```

```
toxb_avr1co39_avrpik_avrpib_avrpia-filtered_logo-maxgap0.5
```

```
toxb_avr1co39_avrpik_avrpib_avrpia-filtered_logo_group
```

```
toxb_avr1co39_avrpik_avrpib_avrpia-filtered_logo_group-maxgap0.5
```

```
toxb_avr1co39_avrpik_avrpib_avrpia-filtered_logo_group-maxgap0.5minsize10
```

```
toxb_avr1co39_avrpik_avrpib_avrpia-unfiltered_logo_group
```

```
toxb_avr1co39_avrpik_avrpib_avrpia-unfiltered_logo_group-maxgap0.5
```

```
toxb_avr1co39_avrpik_avrpib_avrpia-unfiltered_logo_group-maxgap0.5minsize10
```

```
toxb_avr1co39_avrpik_avrpib_avrpia-unfiltered_markov
```

```
toxb_avr1co39_avrpik_avrpib_avrpia-unfiltered_greedy
```

```
toxb_avr1co39_avrpik_avrpib_avrpia-filtered_markov
```

```
toxb_avr1co39_avrpik_avrpib_avrpia-filtered_greedy
```

```
toxb_avr1co39_avrpik_avrpib_avrpia-unfiltered_markov-maxgap0.5
```

```
toxb_avr1co39_avrpik_avrpib_avrpia-unfiltered_greedy-maxgap0.5
```

```
toxb_avr1co39_avrpik_avrpib_avrpia-filtered_markov-maxgap0.5
```

```
toxb_avr1co39_avrpik_avrpib_avrpia-filtered_greedy-maxgap0.5
```

In [88]:

```
alnlogo(
    "figure-toxb_avr1co39_avrpik_avrpib_avrpia-filtered-gt0.5",
    toxb_filtered_dfs[0],
    toxb_greedy_cmap,
    toxb_markov_cmap,
    max_gap=0.5,
    outdir=OUTDIR,
    width_per_base=0.06,
    height_per_seq=0.03,
    wratio=[0.015, 0.015, 0.015, 0.015, 1],
    hratio=[0.15, 1]
)
```

In [89]:

```
alnlogo(
    "figure-toxb_avr1co39_avrpik_avrpib_avrpia-filtered",
    toxb_filtered_dfs[0],
    toxb_greedy_cmap,
    toxb_markov_cmap,
    max_gap=1,
    outdir=OUTDIR,
    width_per_base=0.05,
    height_per_seq=0.03,
    wratio=[0.01, 0.01, 0.01, 0.01, 1],
    hratio=[0.15, 1]
)
```

In [90]:

```
plot_logo_group(
    toxb_unfiltered_dfs[0],
    "figure-toxb_avr1co39_avrpik_avrpib_avrpia-unfiltered-gt0.5gt10",
    toxb_markov_cmap,
    max_gap=0.5,
    min_size=10,
    outdir=OUTDIR,
    width_per_res=0.09,
    height=1.5
)
```

In [135]:

```
plot_logo_group(
    toxb_unfiltered_dfs[0],
    "figure-toxb_avr1co39_avrpik_avrpib_avrpia-unfiltered-gt0.5",
    toxb_markov_cmap,
    max_gap=0.5,
    outdir=OUTDIR,
    width_per_res=0.09,
    height=1.5
)
```

In [136]:

```
plot_logo_group(
    toxb_filtered_dfs[0],
    "figure-toxb_avr1co39_avrpik_avrpib_avrpia-filtered-gt0.5gt10",
    toxb_markov_cmap,
    max_gap=0.5,
    min_size=10,
    outdir=OUTDIR,
    width_per_res=0.09,
    height=1.5
)
```

In [137]:

```
plot_logo_group(
    toxb_filtered_dfs[0],
    "figure-toxb_avr1co39_avrpik_avrpib_avrpia-filtered-gt0.5",
    toxb_markov_cmap,
    max_gap=0.5,
    outdir=OUTDIR,
    width_per_res=0.09,
    height=1.5
)
```

In [ ]:

In [ ]:

In [ ]:

In [139]:

```
avrpizt_df = eff_with_unique[
    eff_with_unique["supercluster_component"].isin(["PC_02W1ZZ"])
]
avrpizt_df.head()
```

Out[139]:

|  | supercluster\_component | supercluster\_markov | supercluster\_greedy | cluster | effectors | phibase | organisms | lca\_rank | lca | cluster\_size | cluster\_length | member |
| --- | --- | --- | --- | --- | --- | --- | --- | --- | --- | --- | --- | --- |
| 175448 | PC\_02W1ZZ | PC\_02W1ZZ | PC\_04M1RR | PC\_02W1ZZ | NaN | NaN | Pyricularia oryzae | species | Pyricularia oryzae | 1 | 80 | PC\_02W1ZZ |
| 175449 | PC\_02W1ZZ | PC\_02W1ZZ | PC\_04M1RR | PC\_04M1RR | MoAvrPiz\_t | C6ZEZ6#PHI:2064\_\_PHI:7896\_\_PHI:8697 | Pyricularia oryzae Y34;Pyricularia oryzae 70-1... | genus | Pyricularia | 14 | 108 | PC\_04M1RR |
| 175450 | PC\_02W1ZZ | PC\_02W1ZZ | PC\_04M1RR | PC\_04M1RR | MoAvrPiz\_t | C6ZEZ6#PHI:2064\_\_PHI:7896\_\_PHI:8697 | Pyricularia oryzae Y34;Pyricularia oryzae 70-1... | genus | Pyricularia | 14 | 108 | PC\_08JNGG |
| 175451 | PC\_02W1ZZ | PC\_02W1ZZ | PC\_04M1RR | PC\_04M1RR | MoAvrPiz\_t | C6ZEZ6#PHI:2064\_\_PHI:7896\_\_PHI:8697 | Pyricularia oryzae Y34;Pyricularia oryzae 70-1... | genus | Pyricularia | 14 | 108 | PC\_05XX8W |
| 175452 | PC\_02W1ZZ | PC\_02W1ZZ | PC\_04M1RR | PC\_04M1RR | MoAvrPiz\_t | C6ZEZ6#PHI:2064\_\_PHI:7896\_\_PHI:8697 | Pyricularia oryzae Y34;Pyricularia oryzae 70-1... | genus | Pyricularia | 14 | 108 | PC\_0363GG |

In [140]:

```
avrpizt_hmms, avrpizt_msas, avrpizt_unfiltered_dfs, avrpizt_filtered_dfs, avrpizt_markov_cmap, avrpizt_greedy_cmap = runit(
    "avrpizt",
    G,
    avrpizt_df,
    enriched,
    unenriched,
)
```

```
markov
```

```
greedy
```

```
markov mst
```

```
greedy mst
```

```
there are 1 alignments. Each with 8 sequences.
# hmmbuild :: profile HMM construction from multiple sequence alignments
# HMMER 3.3 (Nov 2019); http://hmmer.org/
# Copyright (C) 2019 Howard Hughes Medical Institute.
# Freely distributed under the BSD open source license.
# - - - - - - - - - - - - - - - - - - - - - - - - - - - - - - - - - - - -
# input alignment file:             /tmp/tmp6bformeg
# output HMM file:                  /tmp/tmpy4ebjm19
# name (the single) HMM:            avrpizt
# input alignment is asserted as:   protein
# - - - - - - - - - - - - - - - - - - - - - - - - - - - - - - - - - - - -

# idx name                  nseq  alen  mlen eff_nseq re/pos description
#---- -------------------- ----- ----- ----- -------- ------ -----------
1     avrpizt                  8    82    75     1.31  0.752 

# CPU time: 0.03u 0.00s 00:00:00.03 Elapsed: 00:00:00.03
```

In [141]:

```
get_plots(
    "avrpizt",
    0,
    avrpizt_hmms,
    avrpizt_msas,
    avrpizt_unfiltered_dfs,
    avrpizt_filtered_dfs,
    avrpizt_markov_cmap,
    avrpizt_greedy_cmap,
    run_fastree=True,
    show=True,
)
```

```
# hmmbuild :: profile HMM construction from multiple sequence alignments
# HMMER 3.3 (Nov 2019); http://hmmer.org/
# Copyright (C) 2019 Howard Hughes Medical Institute.
# Freely distributed under the BSD open source license.
# - - - - - - - - - - - - - - - - - - - - - - - - - - - - - - - - - - - -
# input alignment file:             /tmp/tmp6nzbaf5x
# output HMM file:                  /tmp/tmpxn092fuc
# name (the single) HMM:            avrpizt
# input alignment is asserted as:   protein
# - - - - - - - - - - - - - - - - - - - - - - - - - - - - - - - - - - - -

# idx name                  nseq  alen  mlen eff_nseq re/pos description
#---- -------------------- ----- ----- ----- -------- ------ -----------
1     avrpizt                  7   113   102     0.89  0.591 

# CPU time: 0.04u 0.00s 00:00:00.04 Elapsed: 00:00:00.04


avrpizt-unfiltered_logo
```

```
avrpizt-unfiltered_logo-maxgap0.5
```

```
avrpizt-filtered_logo
```

```
avrpizt-filtered_logo-maxgap0.5
```

```
avrpizt-filtered_logo_group
```

```
avrpizt-filtered_logo_group-maxgap0.5
```

```
avrpizt-filtered_logo_group-maxgap0.5minsize10
```

```
---------------------------------------------------------------------------
LogomakerError                            Traceback (most recent call last)
<ipython-input-141-3a6560ed2042> in <module>
      9     avrpizt_greedy_cmap,
     10     run_fastree=True,
---> 11     show=True,
     12 )

<ipython-input-45-faf363bbac27> in get_plots(name, index, hmms, msas, unfiltered_dfs, filtered_dfs, markov_cmap, greedy_cmap, outdir, palette, logo_palette, run_fastree, show)
     64     print(n)
     65     l = plot_logo_group(filtered_df, n,
---> 66                         markov_cmap, max_gap=0.5, min_size=10, show=show, outdir=outdir)
     67 
     68 

<ipython-input-134-7f485e5fc5a3> in plot_logo_group(df, name, cluster_cmap, row_order, min_size, max_gap, outdir, base_width, width_per_res, height, supercluster_col, alignment_col, sharey, show, logo_palette)
     35             characters_to_ignore=".-X",
     36             to_type="counts",
---> 37             pseudocount=0
     38         )
     39         filter_ = (1 - (mat_counts.sum(axis=1) / len(df[alignment_col]))) <= max_gap

~/.miniconda/envs/remote_homology/lib/python3.7/site-packages/logomaker/src/error_handling.py in wrapped_func(*args, **kwargs)
    132             # Otherwise, raise error
    133             else:
--> 134                 raise e
    135 
    136         # If not in debug mode, and no error was detected above,

~/.miniconda/envs/remote_homology/lib/python3.7/site-packages/logomaker/src/error_handling.py in wrapped_func(*args, **kwargs)
    100         try:
    101             # Execute function
--> 102             result = func(*args, **kwargs)
    103 
    104             # If running functional test and expect to fail

~/.miniconda/envs/remote_homology/lib/python3.7/site-packages/logomaker/src/matrix.py in alignment_to_matrix(sequences, counts, to_type, background, characters_to_ignore, center_weights, pseudocount)
    519 
    520     # Make sure sequences has at least 1 element
--> 521     check(len(sequences) > 0, 'sequences must have length > 0.')
    522 
    523     # Make sure all elements are sequences

~/.miniconda/envs/remote_homology/lib/python3.7/site-packages/logomaker/src/error_handling.py in check(condition, message)
     54 
     55     if not condition:
---> 56         raise LogomakerError(message)
     57 
     58 

LogomakerError: sequences must have length > 0.
```

In [189]:

```
alnlogo(
    "figure-avrpizt-filtered",
    avrpizt_filtered_dfs[0],
    avrpizt_greedy_cmap,
    avrpizt_markov_cmap,
    max_gap=1,
    outdir=OUTDIR,
    width_per_base=0.07,
    height_per_seq=0.1,
    wratio=[0.01, 0.01, 0.01, 0.01, 1],
    hratio=[0.2, 1]
)
```

In [ ]:

In [142]:

```
rnase_df = eff_with_unique[
    eff_with_unique["supercluster_component"]
    .isin(eff_with_unique.loc[
        eff_with_unique["effectors"].isin(['BgtAvrPm3a2f2']), "supercluster_component"])
]
rnase_df.head()
```

Out[142]:

|  | supercluster\_component | supercluster\_markov | supercluster\_greedy | cluster | effectors | phibase | organisms | lca\_rank | lca | cluster\_size | cluster\_length | member |
| --- | --- | --- | --- | --- | --- | --- | --- | --- | --- | --- | --- | --- |
| 181806 | PC\_032CKH | PC\_00595C | PC\_00595C | PC\_00595C | NaN | NaN | Blumeria graminis f. sp. hordei;Blumeria grami... | species | Blumeria graminis | 40 | 135 | PC\_00595C |
| 181807 | PC\_032CKH | PC\_00595C | PC\_00595C | PC\_00595C | NaN | NaN | Blumeria graminis f. sp. hordei;Blumeria grami... | species | Blumeria graminis | 40 | 135 | PC\_052368 |
| 181808 | PC\_032CKH | PC\_00595C | PC\_00595C | PC\_00595C | NaN | NaN | Blumeria graminis f. sp. hordei;Blumeria grami... | species | Blumeria graminis | 40 | 135 | PC\_017HDF |
| 181809 | PC\_032CKH | PC\_00595C | PC\_00595C | PC\_00595C | NaN | NaN | Blumeria graminis f. sp. hordei;Blumeria grami... | species | Blumeria graminis | 40 | 135 | PC\_039J9Z |
| 181810 | PC\_032CKH | PC\_00595C | PC\_00595C | PC\_00595C | NaN | NaN | Blumeria graminis f. sp. hordei;Blumeria grami... | species | Blumeria graminis | 40 | 135 | PC\_0693MJ |

In [143]:

```
rnase_hmms, rnase_msas, rnase_unfiltered_dfs, rnase_filtered_dfs, rnase_markov_cmap, rnase_greedy_cmap = runit(
    "rnase",
    G,
    rnase_df,
    enriched,
    unenriched,
)
```

```
markov
```

```
greedy
```

```
markov mst
```

```
greedy mst
```

```
there are 4 alignments. Each with 111, 36, 12, 6 sequences.
# hmmbuild :: profile HMM construction from multiple sequence alignments
# HMMER 3.3 (Nov 2019); http://hmmer.org/
# Copyright (C) 2019 Howard Hughes Medical Institute.
# Freely distributed under the BSD open source license.
# - - - - - - - - - - - - - - - - - - - - - - - - - - - - - - - - - - - -
# input alignment file:             /tmp/tmply0a605m
# output HMM file:                  /tmp/tmp29ltdcg1
# name (the single) HMM:            rnase
# input alignment is asserted as:   protein
# - - - - - - - - - - - - - - - - - - - - - - - - - - - - - - - - - - - -

# idx name                  nseq  alen  mlen eff_nseq re/pos description
#---- -------------------- ----- ----- ----- -------- ------ -----------
1     rnase                  111    94    80     5.28  0.709 

# CPU time: 0.04u 0.00s 00:00:00.04 Elapsed: 00:00:00.04


# hmmbuild :: profile HMM construction from multiple sequence alignments
# HMMER 3.3 (Nov 2019); http://hmmer.org/
# Copyright (C) 2019 Howard Hughes Medical Institute.
# Freely distributed under the BSD open source license.
# - - - - - - - - - - - - - - - - - - - - - - - - - - - - - - - - - - - -
# input alignment file:             /tmp/tmpl6sqx8bz
# output HMM file:                  /tmp/tmp32vhsmow
# name (the single) HMM:            rnase
# input alignment is asserted as:   protein
# - - - - - - - - - - - - - - - - - - - - - - - - - - - - - - - - - - - -

# idx name                  nseq  alen  mlen eff_nseq re/pos description
#---- -------------------- ----- ----- ----- -------- ------ -----------
1     rnase                   36   121   101     1.74  0.591 

# CPU time: 0.04u 0.01s 00:00:00.05 Elapsed: 00:00:00.04


# hmmbuild :: profile HMM construction from multiple sequence alignments
# HMMER 3.3 (Nov 2019); http://hmmer.org/
# Copyright (C) 2019 Howard Hughes Medical Institute.
# Freely distributed under the BSD open source license.
# - - - - - - - - - - - - - - - - - - - - - - - - - - - - - - - - - - - -
# input alignment file:             /tmp/tmpx7jfahzv
# output HMM file:                  /tmp/tmpf_si_zef
# name (the single) HMM:            rnase
# input alignment is asserted as:   protein
# - - - - - - - - - - - - - - - - - - - - - - - - - - - - - - - - - - - -

# idx name                  nseq  alen  mlen eff_nseq re/pos description
#---- -------------------- ----- ----- ----- -------- ------ -----------
1     rnase                   12   167   123     1.91  0.591 

# CPU time: 0.05u 0.00s 00:00:00.05 Elapsed: 00:00:00.05


# hmmbuild :: profile HMM construction from multiple sequence alignments
# HMMER 3.3 (Nov 2019); http://hmmer.org/
# Copyright (C) 2019 Howard Hughes Medical Institute.
# Freely distributed under the BSD open source license.
# - - - - - - - - - - - - - - - - - - - - - - - - - - - - - - - - - - - -
# input alignment file:             /tmp/tmp40naex6x
# output HMM file:                  /tmp/tmpjtmkf5s7
# name (the single) HMM:            rnase
# input alignment is asserted as:   protein
# - - - - - - - - - - - - - - - - - - - - - - - - - - - - - - - - - - - -

# idx name                  nseq  alen  mlen eff_nseq re/pos description
#---- -------------------- ----- ----- ----- -------- ------ -----------
1     rnase                    6   171   169     0.57  0.590 

# CPU time: 0.06u 0.00s 00:00:00.06 Elapsed: 00:00:00.06
```

The rnases also fall into multiple groups that can't be merged.
What are they?

In [144]:

```
rnaseG = get_subgraph(G, rnase_df["cluster"].unique())
rnase_mst = gt.min_spanning_tree(rnaseG, weights=rnaseG.edge_properties["neg"])
rnase_cluster_msas = {c: enriched[c.encode()][:-1] for c in rnase_df["cluster"].unique()}

rnase_prog_msas = progressive(rnaseG, rnase_mst, rnase_cluster_msas, verbose=True)
```

```
Edge PC_02UBXP PC_060QAC 0.6877759099006653
MSA succeeded
Edge PC_012M5T PC_01OCWE 0.6773021817207336
MSA succeeded
Edge PC_01WPL7 PC_04HZ79 0.6600525379180908
MSA succeeded
Edge PC_060TP7 PC_072DWQ 0.6518558263778687
MSA succeeded
Edge PC_02GYSI PC_0306GU 0.6499834060668945
MSA succeeded
Edge PC_02P3RH PC_06SE34 0.6492133140563965
MSA succeeded
Edge PC_04KM1I PC_06UZ06 0.6451504826545715
MSA succeeded
Edge PC_0278ZT PC_07TV5W 0.6450186371803284
MSA succeeded
Edge PC_0091OE PC_07TUTP 0.6372565627098083
MSA succeeded
Edge PC_0085EC PC_02BNS6 0.6349347829818726
MSA succeeded
Edge PC_009BF9 PC_01CVKZ 0.630730926990509
MSA succeeded
Edge PC_009BF9 PC_02QUA0 0.6298078298568726
lkey 10
lkey_clusters {'PC_01CVKZ', 'PC_009BF9'}
MSA succeeded
Edge PC_02GRBP PC_02K5A5 0.6274924874305725
MSA succeeded
Edge PC_016FRE PC_03PSAN 0.6207807064056396
MSA succeeded
Edge PC_01OH6B PC_04KM1I 0.6156569719314575
MSA succeeded
Edge PC_0109ZP PC_01VGHN 0.6145864129066467
MSA succeeded
Edge PC_009BF9 PC_06WVB2 0.6071569919586182
lkey 11
lkey_clusters {'PC_02QUA0', 'PC_01CVKZ', 'PC_009BF9'}
MSA succeeded
Edge PC_022NID PC_02R0M8 0.6019706130027771
MSA succeeded
Edge PC_07UIKR PC_08H11O 0.6013807058334351
MSA succeeded
Edge PC_011LAW PC_03EHDR 0.6003285646438599
MSA succeeded
Edge PC_016FRE PC_06WVB2 0.5963689684867859
lkey 13
lkey_clusters {'PC_016FRE', 'PC_03PSAN'}
MSA succeeded
Edge PC_00LSXD PC_0837FY 0.5895354747772217
MSA succeeded
Edge PC_01QQ1T PC_073XSY 0.5884699821472168
MSA succeeded
Edge PC_01FQRU PC_074GF9 0.5826748013496399
MSA succeeded
Edge PC_018DM6 PC_0278ZT 0.5820120573043823
MSA succeeded
Edge PC_02OSJL PC_03813O 0.5796492695808411
MSA succeeded
Edge PC_05N2GV PC_0753N9 0.5784077644348145
MSA succeeded
Edge PC_04MR4A PC_07TV5W 0.5755965709686279
MSA succeeded
Edge PC_04OIFF PC_07UIKR 0.5738135576248169
MSA succeeded
Edge PC_018GH8 PC_06MGDC 0.5736923217773438
MSA succeeded
Edge PC_045E9O PC_04R5US 0.5725969076156616
MSA succeeded
Edge PC_039ZP9 PC_05FXKZ 0.5718848705291748
MSA succeeded
Edge PC_00M7NJ PC_04OIFF 0.5693345069885254
MSA succeeded
Edge PC_04M1B3 PC_07TUTP 0.5690626502037048
MSA succeeded
Edge PC_0306GU PC_05L7US 0.5673298239707947
lkey 4
lkey_clusters {'PC_0306GU', 'PC_02GYSI'}
MSA succeeded
Edge PC_03HGF9 PC_074DQF 0.5632222890853882
MSA succeeded
Edge PC_042YRE PC_07UIKR 0.5535540580749512
MSA succeeded
Edge PC_01FQRU PC_03813O 0.5532559156417847
lkey 23
lkey_clusters {'PC_01FQRU', 'PC_074GF9'}
MSA succeeded
Edge PC_002I3M PC_01OH6B 0.551506519317627
MSA succeeded
Edge PC_02OSJL PC_05TZZ7 0.5509747266769409
lkey 37
lkey_clusters {'PC_01FQRU', 'PC_02OSJL', 'PC_074GF9', 'PC_03813O'}
MSA succeeded
Edge PC_00A3UY PC_02GRBP 0.5501709580421448
MSA succeeded
Edge PC_00NN46 PC_07AGJQ 0.5499635934829712
MSA succeeded
Edge PC_0654DH PC_06EWH0 0.5494096279144287
MSA succeeded
Edge PC_03EHDR PC_05F9H4 0.5486013293266296
lkey 19
lkey_clusters {'PC_03EHDR', 'PC_011LAW'}
MSA succeeded
Edge PC_02QIDH PC_07TV4U 0.5483132600784302
MSA succeeded
Edge PC_03PSAN PC_04D7VO 0.5461366772651672
lkey 20
lkey_clusters {'PC_01CVKZ', 'PC_02QUA0', 'PC_009BF9', 'PC_016FRE', 'PC_06WVB2', 'PC_03PSAN'}
MSA succeeded
Edge PC_02JM6C PC_07UIKR 0.5426766276359558
MSA succeeded
Edge PC_02K5A5 PC_07TVM3 0.542299211025238
lkey 40
lkey_clusters {'PC_02K5A5', 'PC_00A3UY', 'PC_02GRBP'}
MSA succeeded
Edge PC_057CGE PC_07TVM3 0.5386976599693298
MSA succeeded
Edge PC_059TG9 PC_06MGDC 0.5380460023880005
MSA succeeded
Edge PC_01296N PC_07AGJQ 0.5379142165184021
MSA succeeded
Edge PC_06PIV6 PC_07TUWD 0.5364763140678406
MSA succeeded
Edge PC_03970R PC_05W9ZE 0.5338674783706665
MSA succeeded
Edge PC_00595C PC_00646F 0.53367018699646
MSA succeeded
Edge PC_008806 PC_02V4AV 0.5327125787734985
MSA succeeded
Edge PC_00NE3P PC_04THYL 0.5325245261192322
MSA succeeded
Edge PC_01FQRU PC_02D1GK 0.5314738154411316
lkey 39
lkey_clusters {'PC_03813O', 'PC_05TZZ7', 'PC_01FQRU', 'PC_02OSJL', 'PC_074GF9'}
MSA succeeded
Edge PC_07TV8B PC_07TVM3 0.5311857461929321
MSA succeeded
Edge PC_01YHQS PC_05KG1N 0.5295529961585999
MSA succeeded
Edge PC_001RDT PC_02OSJL 0.5287690758705139
MSA succeeded
Edge PC_05XT7X PC_06WVB2 0.5283653736114502
MSA succeeded
Edge PC_002I3M PC_016FRE 0.528316080570221
lkey 38
lkey_clusters {'PC_06UZ06', 'PC_04KM1I', 'PC_002I3M', 'PC_01OH6B'}
MSA succeeded
Edge PC_02KEHU PC_03813O 0.5272538065910339
MSA succeeded
Edge PC_029YIZ PC_03FKVQ 0.5268643498420715
MSA succeeded
Edge PC_01296N PC_087UOG 0.5259629487991333
lkey 50
lkey_clusters {'PC_00NN46', 'PC_07AGJQ', 'PC_01296N'}
MSA succeeded
Edge PC_02OSJL PC_032CKH 0.5247979164123535
lkey 62
lkey_clusters {'PC_02D1GK', 'PC_03813O', 'PC_001RDT', 'PC_02KEHU', 'PC_05TZZ7', 'PC_02OSJL', 'PC_01FQRU', 'PC_074GF9'}
MSA succeeded
Edge PC_057VS9 PC_06K136 0.5228093266487122
MSA succeeded
Edge PC_0109ZP PC_05VZ2W 0.5201234817504883
lkey 15
lkey_clusters {'PC_0109ZP', 'PC_01VGHN'}
MSA succeeded
Edge PC_017F5K PC_07AGJQ 0.5199341177940369
MSA succeeded
Edge PC_02OSJL PC_03PEL5 0.5180040597915649
lkey 65
lkey_clusters {'PC_02D1GK', 'PC_03813O', 'PC_032CKH', 'PC_001RDT', 'PC_02KEHU', 'PC_05TZZ7', 'PC_02OSJL', 'PC_01FQRU', 'PC_074GF9'}
MSA succeeded
Edge PC_05RS5N PC_0654DH 0.5176547169685364
MSA succeeded
Edge PC_01EAHK PC_060UD5 0.5170606970787048
MSA succeeded
Edge PC_026AIN PC_03XWEE 0.5163021087646484
MSA succeeded
Edge PC_00QNZJ PC_07TUWD 0.5158467888832092
MSA succeeded
Edge PC_009036 PC_03813O 0.515701174736023
MSA succeeded
Edge PC_03PEL5 PC_07076R 0.5150887370109558
lkey 74
lkey_clusters {'PC_02D1GK', 'PC_03813O', 'PC_032CKH', 'PC_009036', 'PC_001RDT', 'PC_02KEHU', 'PC_05TZZ7', 'PC_02OSJL', 'PC_01FQRU', 'PC_03PEL5', 'PC_074GF9'}
MSA succeeded
Edge PC_03AMPS PC_0490I5 0.5146458148956299
MSA succeeded
Edge PC_02V4LH PC_081PV4 0.5123651027679443
MSA succeeded
Edge PC_0370DG PC_087UOG 0.5122812390327454
MSA succeeded
Edge PC_002I4B PC_004U8P 0.5117825865745544
MSA succeeded
Edge PC_00LBZX PC_06RYIX 0.5108935832977295
MSA succeeded
Edge PC_051C8B PC_06K136 0.510413408279419
MSA succeeded
Edge PC_01YHQS PC_06J307 0.5103392004966736
lkey 58
lkey_clusters {'PC_05KG1N', 'PC_01YHQS'}
MSA succeeded
Edge PC_01FQRU PC_06FIXN 0.5100414752960205
lkey 75
lkey_clusters {'PC_07076R', 'PC_02D1GK', 'PC_03813O', 'PC_032CKH', 'PC_009036', 'PC_001RDT', 'PC_02KEHU', 'PC_05TZZ7', 'PC_02OSJL', 'PC_01FQRU', 'PC_03PEL5', 'PC_074GF9'}
MSA succeeded
Edge PC_04M1B3 PC_074DQF 0.5093894004821777
lkey 33
lkey_clusters {'PC_04M1B3', 'PC_07TUTP', 'PC_0091OE'}
MSA succeeded
Edge PC_0753N9 PC_0843Z9 0.5089358687400818
lkey 26
lkey_clusters {'PC_0753N9', 'PC_05N2GV'}
MSA succeeded
Edge PC_016TKH PC_02T3ZI 0.5068883299827576
MSA succeeded
Edge PC_013NPU PC_03792B 0.5068206191062927
MSA succeeded
Edge PC_003XGF PC_07TUWD 0.5057196021080017
MSA succeeded
Edge PC_00DU1R PC_06K136 0.5035116672515869
MSA succeeded
Edge PC_0306GU PC_04D7VO 0.5034434795379639
lkey 34
lkey_clusters {'PC_0306GU', 'PC_05L7US', 'PC_02GYSI'}
MSA succeeded
Edge PC_02OSJL PC_04DQ0W 0.5030863285064697
lkey 83
lkey_clusters {'PC_07076R', 'PC_06FIXN', 'PC_02D1GK', 'PC_03813O', 'PC_032CKH', 'PC_009036', 'PC_001RDT', 'PC_02KEHU', 'PC_05TZZ7', 'PC_02OSJL', 'PC_01FQRU', 'PC_03PEL5', 'PC_074GF9'}
MSA succeeded
Edge PC_00QNZJ PC_0519TF 0.5026710629463196
lkey 88
lkey_clusters {'PC_003XGF', 'PC_00QNZJ', 'PC_07TUWD', 'PC_06PIV6'}
MSA succeeded
Edge PC_02C8S3 PC_02QUA0 0.5003723502159119
MSA succeeded
Edge PC_00S0UG PC_05AVFK 0.49957001209259033
MSA succeeded
Edge PC_00S0UG PC_016TKH 0.4993741512298584
lkey 94
lkey_clusters {'PC_05AVFK', 'PC_00S0UG'}
MSA succeeded
Edge PC_01FJ7Z PC_02V4LH 0.49520808458328247
MSA succeeded
Edge PC_030G1S PC_05OR8I 0.4945029616355896
MSA succeeded
Edge PC_03AMPS PC_045E9O 0.4932720363140106
lkey 76
lkey_clusters {'PC_0490I5', 'PC_03AMPS'}
MSA succeeded
Edge PC_06BP65 PC_0804W2 0.4895142614841461
MSA succeeded
Edge PC_02V4AV PC_06J307 0.4894751012325287
lkey 54
lkey_clusters {'PC_008806', 'PC_02V4AV'}
MSA succeeded
Edge PC_00915C PC_0804W2 0.4884128272533417
MSA succeeded
Edge PC_007Z8T PC_0804W2 0.4876667261123657
MSA succeeded
Edge PC_02QW19 PC_04SFDZ 0.4876174032688141
MSA succeeded
Edge PC_027RDL PC_05MI4Z 0.4871160089969635
MSA succeeded
Edge PC_00641O PC_029RUP 0.48447486758232117
MSA succeeded
Edge PC_02WCO0 PC_05ILWE 0.4842674732208252
MSA succeeded
Edge PC_057VS9 PC_06RYIX 0.4836794435977936
lkey 89
lkey_clusters {'PC_057VS9', 'PC_051C8B', 'PC_06K136', 'PC_00DU1R'}
MSA succeeded
Edge PC_01Y0R1 PC_05W6XP 0.4830918610095978
MSA succeeded
Edge PC_03PEL5 PC_05QB64 0.48257383704185486
lkey 91
lkey_clusters {'PC_07076R', 'PC_06FIXN', 'PC_02D1GK', 'PC_03813O', 'PC_032CKH', 'PC_009036', 'PC_04DQ0W', 'PC_001RDT', 'PC_02KEHU', 'PC_05TZZ7', 'PC_02OSJL', 'PC_01FQRU', 'PC_03PEL5', 'PC_074GF9'}
MSA succeeded
Edge PC_00595C PC_073XSY 0.4825042486190796
lkey 53
lkey_clusters {'PC_00646F', 'PC_00595C'}
MSA succeeded
Edge PC_02T3ZI PC_0490I5 0.4818899631500244
lkey 95
lkey_clusters {'PC_05AVFK', 'PC_00S0UG', 'PC_016TKH', 'PC_02T3ZI'}
MSA succeeded
Edge PC_00QNZJ PC_0287UU 0.48177748918533325
lkey 92
lkey_clusters {'PC_0519TF', 'PC_06PIV6', 'PC_003XGF', 'PC_00QNZJ', 'PC_07TUWD'}
MSA succeeded
Edge PC_00UWAQ PC_081PV4 0.4813807010650635
MSA succeeded
Edge PC_01IC1B PC_06J307 0.47727128863334656
MSA succeeded
Edge PC_03813O PC_0860OA 0.4749937653541565
lkey 109
lkey_clusters {'PC_07076R', 'PC_06FIXN', 'PC_02D1GK', 'PC_03813O', 'PC_032CKH', 'PC_009036', 'PC_04DQ0W', 'PC_001RDT', 'PC_02KEHU', 'PC_05TZZ7', 'PC_02OSJL', 'PC_01FQRU', 'PC_05QB64', 'PC_03PEL5', 'PC_074GF9'}
MSA succeeded
Edge PC_00QDYY PC_00QNZJ 0.4748527705669403
MSA succeeded
Edge PC_05OR8I PC_073NJA 0.4743748605251312
lkey 97
lkey_clusters {'PC_030G1S', 'PC_05OR8I'}
MSA succeeded
Edge PC_032CKH PC_03JFL5 0.4731172025203705
lkey 115
lkey_clusters {'PC_07076R', 'PC_06FIXN', 'PC_02D1GK', 'PC_03813O', 'PC_032CKH', 'PC_009036', 'PC_04DQ0W', 'PC_001RDT', 'PC_02KEHU', 'PC_05TZZ7', 'PC_02OSJL', 'PC_01FQRU', 'PC_05QB64', 'PC_03PEL5', 'PC_0860OA', 'PC_074GF9'}
MSA succeeded
Edge PC_060UD5 PC_063IUN 0.47238489985466003
lkey 71
lkey_clusters {'PC_060UD5', 'PC_01EAHK'}
MSA succeeded
Edge PC_016TKH PC_01J1VJ 0.4723544716835022
lkey 111
lkey_clusters {'PC_02T3ZI', 'PC_05AVFK', 'PC_0490I5', 'PC_03AMPS', 'PC_04R5US', 'PC_00S0UG', 'PC_016TKH', 'PC_045E9O'}
MSA succeeded
Edge PC_00772B PC_02KEHU 0.4718433916568756
MSA succeeded
Edge PC_007Z8T PC_05K9FR 0.4708157181739807
lkey 102
lkey_clusters {'PC_0804W2', 'PC_00915C', 'PC_007Z8T', 'PC_06BP65'}
MSA succeeded
Edge PC_00915C PC_06IRIY 0.47055578231811523
lkey 122
lkey_clusters {'PC_007Z8T', 'PC_06BP65', 'PC_05K9FR', 'PC_0804W2', 'PC_00915C'}
MSA succeeded
Edge PC_002I4B PC_05MVJQ 0.46961748600006104
lkey 79
lkey_clusters {'PC_004U8P', 'PC_002I4B'}
MSA succeeded
Edge PC_02OSJL PC_072EU7 0.46925848722457886
lkey 121
lkey_clusters {'PC_02D1GK', 'PC_032CKH', 'PC_001RDT', 'PC_05TZZ7', 'PC_02OSJL', 'PC_01FQRU', 'PC_03JFL5', 'PC_00772B', 'PC_07076R', 'PC_06FIXN', 'PC_03813O', 'PC_009036', 'PC_04DQ0W', 'PC_05QB64', 'PC_02KEHU', 'PC_0860OA', 'PC_03PEL5', 'PC_074GF9'}
MSA succeeded
Edge PC_05QB64 PC_081PV4 0.4689939618110657
lkey 125
lkey_clusters {'PC_02D1GK', 'PC_032CKH', 'PC_001RDT', 'PC_05TZZ7', 'PC_02OSJL', 'PC_01FQRU', 'PC_03JFL5', 'PC_00772B', 'PC_072EU7', 'PC_07076R', 'PC_06FIXN', 'PC_03813O', 'PC_009036', 'PC_04DQ0W', 'PC_05QB64', 'PC_02KEHU', 'PC_0860OA', 'PC_03PEL5', 'PC_074GF9'}
MSA succeeded
Edge PC_03PEL5 PC_053J9J 0.4689340591430664
lkey 126
lkey_clusters {'PC_00UWAQ', 'PC_02D1GK', 'PC_081PV4', 'PC_032CKH', 'PC_001RDT', 'PC_05TZZ7', 'PC_02OSJL', 'PC_01FQRU', 'PC_03JFL5', 'PC_00772B', 'PC_072EU7', 'PC_01FJ7Z', 'PC_07076R', 'PC_06FIXN', 'PC_03813O', 'PC_009036', 'PC_04DQ0W', 'PC_02V4LH', 'PC_05QB64', 'PC_02KEHU', 'PC_0860OA', 'PC_03PEL5', 'PC_074GF9'}
MSA succeeded
Edge PC_041GAL PC_05X98I 0.46636343002319336
MSA succeeded
Edge PC_002I4B PC_00NE3P 0.4655693769454956
lkey 124
lkey_clusters {'PC_004U8P', 'PC_002I4B', 'PC_05MVJQ'}
MSA succeeded
Edge PC_01R1KR PC_06RYIX 0.4651573896408081
MSA succeeded
Edge PC_04DUFN PC_072EU7 0.46493616700172424
MSA succeeded
Edge PC_02YR8I PC_03GTZD 0.46361953020095825
MSA succeeded
Edge PC_03G9RN PC_05PFLL 0.46223142743110657
MSA succeeded
Edge PC_03NC9U PC_06IRIY 0.46153002977371216
MSA succeeded
Edge PC_02KEHU PC_07VAB2 0.46003732085227966
lkey 131
lkey_clusters {'PC_00UWAQ', 'PC_02D1GK', 'PC_081PV4', 'PC_032CKH', 'PC_001RDT', 'PC_05TZZ7', 'PC_02OSJL', 'PC_01FQRU', 'PC_03JFL5', 'PC_053J9J', 'PC_00772B', 'PC_072EU7', 'PC_01FJ7Z', 'PC_07076R', 'PC_06FIXN', 'PC_03813O', 'PC_009036', 'PC_04DQ0W', 'PC_02V4LH', 'PC_05QB64', 'PC_02KEHU', 'PC_0860OA', 'PC_03PEL5', 'PC_04DUFN', 'PC_074GF9'}
MSA succeeded
Edge PC_00UWAQ PC_05FIVY 0.4573242962360382
lkey 135
lkey_clusters {'PC_00UWAQ', 'PC_02D1GK', 'PC_081PV4', 'PC_032CKH', 'PC_001RDT', 'PC_05TZZ7', 'PC_02OSJL', 'PC_01FQRU', 'PC_03JFL5', 'PC_07VAB2', 'PC_053J9J', 'PC_00772B', 'PC_072EU7', 'PC_01FJ7Z', 'PC_07076R', 'PC_06FIXN', 'PC_03813O', 'PC_009036', 'PC_04DQ0W', 'PC_02V4LH', 'PC_05QB64', 'PC_02KEHU', 'PC_0860OA', 'PC_03PEL5', 'PC_04DUFN', 'PC_074GF9'}
MSA succeeded
Edge PC_04D7VO PC_05MHF3 0.45633944869041443
lkey 93
lkey_clusters {'PC_06UZ06', 'PC_05L7US', 'PC_04KM1I', 'PC_06WVB2', 'PC_02GYSI', 'PC_01OH6B', 'PC_016FRE', 'PC_02QUA0', 'PC_0306GU', 'PC_01CVKZ', 'PC_002I3M', 'PC_04D7VO', 'PC_02C8S3', 'PC_03PSAN', 'PC_009BF9', 'PC_05XT7X'}
MSA succeeded
Edge PC_02EV3G PC_053J9J 0.4563247263431549
MSA succeeded
Edge PC_007Z8T PC_05W661 0.4559740126132965
lkey 134
lkey_clusters {'PC_0804W2', 'PC_05K9FR', 'PC_007Z8T', 'PC_00915C', 'PC_06BP65', 'PC_03NC9U', 'PC_06IRIY'}
MSA succeeded
Edge PC_02WCO0 PC_04V0PU 0.4559173285961151
lkey 106
lkey_clusters {'PC_05ILWE', 'PC_02WCO0'}
MSA succeeded
Edge PC_03EHDR PC_05FXKZ 0.4548112750053406
lkey 43
lkey_clusters {'PC_03EHDR', 'PC_011LAW', 'PC_05F9H4'}
MSA succeeded
Edge PC_00D5EQ PC_00QNZJ 0.45392921566963196
MSA succeeded
Edge PC_04FB23 PC_063JSC 0.45391538739204407
MSA succeeded
Edge PC_03KYEF PC_07088H 0.45355546474456787
MSA succeeded
Edge PC_01PMZK PC_029RUP 0.4518945515155792
MSA succeeded
Edge PC_00QJR6 PC_07088H 0.45094889402389526
MSA succeeded
Edge PC_03813O PC_06IRIY 0.4505769908428192
lkey 138
lkey_clusters {'PC_00UWAQ', 'PC_02D1GK', 'PC_081PV4', 'PC_032CKH', 'PC_001RDT', 'PC_05TZZ7', 'PC_02OSJL', 'PC_01FQRU', 'PC_03JFL5', 'PC_07VAB2', 'PC_053J9J', 'PC_00772B', 'PC_072EU7', 'PC_01FJ7Z', 'PC_07076R', 'PC_06FIXN', 'PC_03813O', 'PC_009036', 'PC_04DQ0W', 'PC_02V4LH', 'PC_05QB64', 'PC_02KEHU', 'PC_0860OA', 'PC_03PEL5', 'PC_04DUFN', 'PC_05FIVY', 'PC_02EV3G', 'PC_074GF9'}
MSA succeeded
Edge PC_05OR8I PC_06ZU26 0.44951149821281433
lkey 117
lkey_clusters {'PC_073NJA', 'PC_030G1S', 'PC_05OR8I'}
MSA succeeded
Edge PC_02UI5W PC_03PEL5 0.44948291778564453
MSA succeeded
Edge PC_01EZOK PC_04ULOH 0.44897231459617615
MSA succeeded
Edge PC_001RDT PC_06CM2C 0.4486616849899292
lkey 149
lkey_clusters {'PC_06BP65', 'PC_001RDT', 'PC_02OSJL', 'PC_07VAB2', 'PC_03JFL5', 'PC_00772B', 'PC_072EU7', 'PC_06FIXN', 'PC_03813O', 'PC_009036', 'PC_05K9FR', 'PC_03NC9U', 'PC_05QB64', 'PC_0860OA', 'PC_0804W2', 'PC_03PEL5', 'PC_04DUFN', 'PC_02UI5W', 'PC_074GF9', 'PC_00UWAQ', 'PC_02D1GK', 'PC_081PV4', 'PC_032CKH', 'PC_007Z8T', 'PC_05TZZ7', 'PC_01FQRU', 'PC_06IRIY', 'PC_05W661', 'PC_053J9J', 'PC_00915C', 'PC_01FJ7Z', 'PC_07076R', 'PC_04DQ0W', 'PC_02V4LH', 'PC_02KEHU', 'PC_05FIVY', 'PC_02EV3G'}
MSA succeeded
Edge PC_03PEL5 PC_06OK2P 0.4463288486003876
lkey 151
lkey_clusters {'PC_06BP65', 'PC_001RDT', 'PC_02OSJL', 'PC_07VAB2', 'PC_03JFL5', 'PC_06CM2C', 'PC_00772B', 'PC_072EU7', 'PC_06FIXN', 'PC_03813O', 'PC_009036', 'PC_05K9FR', 'PC_03NC9U', 'PC_05QB64', 'PC_0860OA', 'PC_0804W2', 'PC_03PEL5', 'PC_04DUFN', 'PC_02UI5W', 'PC_074GF9', 'PC_00UWAQ', 'PC_02D1GK', 'PC_081PV4', 'PC_032CKH', 'PC_007Z8T', 'PC_05TZZ7', 'PC_01FQRU', 'PC_06IRIY', 'PC_05W661', 'PC_053J9J', 'PC_00915C', 'PC_01FJ7Z', 'PC_07076R', 'PC_04DQ0W', 'PC_02V4LH', 'PC_02KEHU', 'PC_05FIVY', 'PC_02EV3G'}
MSA succeeded
Edge PC_018DM6 PC_06UZ3U 0.44270059466362
lkey 27
lkey_clusters {'PC_04MR4A', 'PC_018DM6', 'PC_0278ZT', 'PC_07TV5W'}
MSA succeeded
Edge PC_02Q6QH PC_06PIV6 0.43916448950767517
MSA succeeded
Edge PC_05AJOI PC_07WEO1 0.4379432201385498
MSA succeeded
Edge PC_030ACC PC_05F9H4 0.43380892276763916
MSA succeeded
Edge PC_03G9RN PC_03GU7O 0.431063175201416
lkey 133
lkey_clusters {'PC_03G9RN', 'PC_05PFLL'}
MSA succeeded
Edge PC_01WTO6 PC_032CKH 0.43103691935539246
MSA succeeded
Edge PC_07XIBT PC_0860OA 0.42854323983192444
MSA succeeded
Edge PC_04TBPN PC_06187I 0.42805567383766174
MSA succeeded
Edge PC_01D3OM PC_07TV9U 0.42493388056755066
MSA succeeded
Edge PC_02OSJL PC_0384LZ 0.4245997369289398
lkey 159
lkey_clusters {'PC_06BP65', 'PC_001RDT', 'PC_02OSJL', 'PC_07VAB2', 'PC_03JFL5', 'PC_06CM2C', 'PC_00772B', 'PC_072EU7', 'PC_07XIBT', 'PC_06FIXN', 'PC_03813O', 'PC_009036', 'PC_05K9FR', 'PC_03NC9U', 'PC_05QB64', 'PC_0860OA', 'PC_0804W2', 'PC_03PEL5', 'PC_04DUFN', 'PC_02UI5W', 'PC_074GF9', 'PC_00UWAQ', 'PC_02D1GK', 'PC_081PV4', 'PC_032CKH', 'PC_007Z8T', 'PC_05TZZ7', 'PC_01FQRU', 'PC_06IRIY', 'PC_05W661', 'PC_053J9J', 'PC_00915C', 'PC_06OK2P', 'PC_01FJ7Z', 'PC_07076R', 'PC_04DQ0W', 'PC_02V4LH', 'PC_01WTO6', 'PC_02KEHU', 'PC_05FIVY', 'PC_02EV3G'}
MSA succeeded
Edge PC_036385 PC_06BP65 0.4232964515686035
MSA succeeded
Edge PC_02QMV0 PC_063IUN 0.4232393205165863
MSA succeeded
Edge PC_03PEL5 PC_04V0PU 0.4182344675064087
lkey 163
lkey_clusters {'PC_06BP65', 'PC_001RDT', 'PC_02OSJL', 'PC_07VAB2', 'PC_03JFL5', 'PC_06CM2C', 'PC_0384LZ', 'PC_00772B', 'PC_072EU7', 'PC_07XIBT', 'PC_06FIXN', 'PC_03813O', 'PC_009036', 'PC_05K9FR', 'PC_03NC9U', 'PC_05QB64', 'PC_0860OA', 'PC_0804W2', 'PC_03PEL5', 'PC_04DUFN', 'PC_02UI5W', 'PC_074GF9', 'PC_00UWAQ', 'PC_02D1GK', 'PC_081PV4', 'PC_032CKH', 'PC_007Z8T', 'PC_05TZZ7', 'PC_01FQRU', 'PC_06IRIY', 'PC_05W661', 'PC_053J9J', 'PC_00915C', 'PC_06OK2P', 'PC_01FJ7Z', 'PC_036385', 'PC_07076R', 'PC_04DQ0W', 'PC_02V4LH', 'PC_01WTO6', 'PC_02KEHU', 'PC_05FIVY', 'PC_02EV3G'}
MSA succeeded
Edge PC_030G1S PC_06J307 0.41714227199554443
lkey 148
lkey_clusters {'PC_073NJA', 'PC_06ZU26', 'PC_030G1S', 'PC_05OR8I'}
MSA succeeded
Edge PC_03NC9U PC_05LDC6 0.41551870107650757
lkey 165
lkey_clusters {'PC_05ILWE', 'PC_06BP65', 'PC_02WCO0', 'PC_001RDT', 'PC_02OSJL', 'PC_07VAB2', 'PC_03JFL5', 'PC_06CM2C', 'PC_0384LZ', 'PC_00772B', 'PC_072EU7', 'PC_07XIBT', 'PC_04V0PU', 'PC_06FIXN', 'PC_03813O', 'PC_009036', 'PC_05K9FR', 'PC_03NC9U', 'PC_05QB64', 'PC_0860OA', 'PC_0804W2', 'PC_03PEL5', 'PC_04DUFN', 'PC_02UI5W', 'PC_074GF9', 'PC_00UWAQ', 'PC_02D1GK', 'PC_081PV4', 'PC_032CKH', 'PC_007Z8T', 'PC_05TZZ7', 'PC_01FQRU', 'PC_06IRIY', 'PC_05W661', 'PC_053J9J', 'PC_00915C', 'PC_06OK2P', 'PC_01FJ7Z', 'PC_036385', 'PC_07076R', 'PC_04DQ0W', 'PC_02V4LH', 'PC_01WTO6', 'PC_02KEHU', 'PC_05FIVY', 'PC_02EV3G'}
MSA succeeded
Edge PC_024FEH PC_02Q6QH 0.4139457941055298
MSA succeeded
Edge PC_00LSXD PC_021V0R 0.41323286294937134
lkey 21
lkey_clusters {'PC_0837FY', 'PC_00LSXD'}
MSA succeeded
Edge PC_0100UP PC_05F9H4 0.4130324125289917
MSA succeeded
Edge PC_087UOG PC_088DQF 0.41103968024253845
lkey 78
lkey_clusters {'PC_087UOG', 'PC_07AGJQ', 'PC_0370DG', 'PC_00NN46', 'PC_017F5K', 'PC_01296N'}
MSA succeeded
Edge PC_03813O PC_03K305 0.41013041138648987
lkey 167
lkey_clusters {'PC_05ILWE', 'PC_06BP65', 'PC_02WCO0', 'PC_001RDT', 'PC_02OSJL', 'PC_07VAB2', 'PC_03JFL5', 'PC_06CM2C', 'PC_0384LZ', 'PC_00772B', 'PC_05LDC6', 'PC_072EU7', 'PC_07XIBT', 'PC_04V0PU', 'PC_06FIXN', 'PC_03813O', 'PC_009036', 'PC_05K9FR', 'PC_03NC9U', 'PC_05QB64', 'PC_0860OA', 'PC_0804W2', 'PC_03PEL5', 'PC_04DUFN', 'PC_02UI5W', 'PC_074GF9', 'PC_00UWAQ', 'PC_02D1GK', 'PC_081PV4', 'PC_032CKH', 'PC_007Z8T', 'PC_05TZZ7', 'PC_01FQRU', 'PC_06IRIY', 'PC_05W661', 'PC_053J9J', 'PC_00915C', 'PC_06OK2P', 'PC_01FJ7Z', 'PC_036385', 'PC_07076R', 'PC_04DQ0W', 'PC_02V4LH', 'PC_01WTO6', 'PC_02KEHU', 'PC_05FIVY', 'PC_02EV3G'}
MSA succeeded
Edge PC_02Q6QH PC_05X98I 0.4094294607639313
lkey 168
lkey_clusters {'PC_00QDYY', 'PC_0519TF', 'PC_024FEH', 'PC_00D5EQ', 'PC_06PIV6', 'PC_003XGF', 'PC_00QNZJ', 'PC_07TUWD', 'PC_0287UU', 'PC_02Q6QH'}
MSA succeeded
Edge PC_032CKH PC_07UMX8 0.4092511236667633
lkey 172
lkey_clusters {'PC_05ILWE', 'PC_06BP65', 'PC_02WCO0', 'PC_001RDT', 'PC_02OSJL', 'PC_07VAB2', 'PC_03JFL5', 'PC_06CM2C', 'PC_0384LZ', 'PC_00772B', 'PC_05LDC6', 'PC_072EU7', 'PC_07XIBT', 'PC_04V0PU', 'PC_06FIXN', 'PC_03813O', 'PC_009036', 'PC_05K9FR', 'PC_03NC9U', 'PC_05QB64', 'PC_0860OA', 'PC_0804W2', 'PC_03PEL5', 'PC_04DUFN', 'PC_02UI5W', 'PC_074GF9', 'PC_00UWAQ', 'PC_02D1GK', 'PC_081PV4', 'PC_032CKH', 'PC_007Z8T', 'PC_05TZZ7', 'PC_01FQRU', 'PC_06IRIY', 'PC_05W661', 'PC_053J9J', 'PC_00915C', 'PC_06OK2P', 'PC_03K305', 'PC_01FJ7Z', 'PC_036385', 'PC_07076R', 'PC_04DQ0W', 'PC_02V4LH', 'PC_01WTO6', 'PC_02KEHU', 'PC_05FIVY', 'PC_02EV3G'}
MSA succeeded
Edge PC_024L0V PC_063TMD 0.40653854608535767
MSA succeeded
Edge PC_02NATZ PC_05X98I 0.40606755018234253
MSA succeeded
Edge PC_01IC1B PC_021SYL 0.40122172236442566
lkey 166
lkey_clusters {'PC_06ZU26', 'PC_030G1S', 'PC_008806', 'PC_01IC1B', 'PC_06J307', 'PC_073NJA', 'PC_05KG1N', 'PC_01YHQS', 'PC_05OR8I', 'PC_02V4AV'}
MSA succeeded
Edge PC_02V4LH PC_06RYNI 0.4003461003303528
lkey 174
lkey_clusters {'PC_05ILWE', 'PC_06BP65', 'PC_02WCO0', 'PC_001RDT', 'PC_02OSJL', 'PC_07VAB2', 'PC_03JFL5', 'PC_06CM2C', 'PC_0384LZ', 'PC_00772B', 'PC_05LDC6', 'PC_072EU7', 'PC_07XIBT', 'PC_04V0PU', 'PC_06FIXN', 'PC_03813O', 'PC_009036', 'PC_05K9FR', 'PC_03NC9U', 'PC_05QB64', 'PC_0860OA', 'PC_0804W2', 'PC_03PEL5', 'PC_04DUFN', 'PC_02UI5W', 'PC_074GF9', 'PC_00UWAQ', 'PC_02D1GK', 'PC_081PV4', 'PC_032CKH', 'PC_007Z8T', 'PC_05TZZ7', 'PC_01FQRU', 'PC_06IRIY', 'PC_05W661', 'PC_053J9J', 'PC_00915C', 'PC_06OK2P', 'PC_03K305', 'PC_01FJ7Z', 'PC_036385', 'PC_07076R', 'PC_07UMX8', 'PC_04DQ0W', 'PC_02V4LH', 'PC_01WTO6', 'PC_02KEHU', 'PC_05FIVY', 'PC_02EV3G'}
MSA succeeded
Edge PC_04E4JE PC_06BL39 0.39843541383743286
MSA succeeded
Edge PC_01GNRM PC_02JM6C 0.39519748091697693
MSA succeeded
Edge PC_01K875 PC_0384LZ 0.3950430750846863
MSA succeeded
Edge PC_00646F PC_01KTUJ 0.39132171869277954
lkey 110
lkey_clusters {'PC_073XSY', 'PC_01QQ1T', 'PC_00646F', 'PC_00595C'}
MSA succeeded
Edge PC_02HVML PC_032CKH 0.38930365443229675
MSA succeeded
Edge PC_0294CS PC_03792B 0.38905709981918335
MSA succeeded
Edge PC_02OSJL PC_08I1Q6 0.38833171129226685
lkey 183
lkey_clusters {'PC_05ILWE', 'PC_06BP65', 'PC_02WCO0', 'PC_001RDT', 'PC_02OSJL', 'PC_07VAB2', 'PC_03JFL5', 'PC_06CM2C', 'PC_0384LZ', 'PC_00772B', 'PC_01K875', 'PC_05LDC6', 'PC_072EU7', 'PC_07XIBT', 'PC_04V0PU', 'PC_06FIXN', 'PC_03813O', 'PC_009036', 'PC_05K9FR', 'PC_03NC9U', 'PC_05QB64', 'PC_0860OA', 'PC_0804W2', 'PC_03PEL5', 'PC_04DUFN', 'PC_02UI5W', 'PC_074GF9', 'PC_00UWAQ', 'PC_02D1GK', 'PC_081PV4', 'PC_032CKH', 'PC_007Z8T', 'PC_02HVML', 'PC_05TZZ7', 'PC_01FQRU', 'PC_06IRIY', 'PC_05W661', 'PC_053J9J', 'PC_00915C', 'PC_06RYNI', 'PC_06OK2P', 'PC_03K305', 'PC_01FJ7Z', 'PC_036385', 'PC_07076R', 'PC_07UMX8', 'PC_04DQ0W', 'PC_02V4LH', 'PC_01WTO6', 'PC_02KEHU', 'PC_05FIVY', 'PC_02EV3G'}
MSA succeeded
Edge PC_00QJR6 PC_08K2VM 0.38609105348587036
lkey 146
lkey_clusters {'PC_00QJR6', 'PC_07088H', 'PC_03KYEF'}
MSA succeeded
Edge PC_021EBE PC_02QUA0 0.3859021067619324
MSA succeeded
Edge PC_06AI9C PC_07076R 0.3858228623867035
MSA succeeded
Edge PC_01WTO6 PC_07VHB5 0.3836725056171417
lkey 188
lkey_clusters {'PC_05ILWE', 'PC_06BP65', 'PC_02WCO0', 'PC_001RDT', 'PC_02OSJL', 'PC_07VAB2', 'PC_03JFL5', 'PC_06CM2C', 'PC_0384LZ', 'PC_00772B', 'PC_01K875', 'PC_05LDC6', 'PC_072EU7', 'PC_07XIBT', 'PC_04V0PU', 'PC_06FIXN', 'PC_03813O', 'PC_009036', 'PC_05K9FR', 'PC_03NC9U', 'PC_05QB64', 'PC_0860OA', 'PC_0804W2', 'PC_03PEL5', 'PC_04DUFN', 'PC_08I1Q6', 'PC_02UI5W', 'PC_074GF9', 'PC_00UWAQ', 'PC_02D1GK', 'PC_081PV4', 'PC_032CKH', 'PC_007Z8T', 'PC_02HVML', 'PC_05TZZ7', 'PC_01FQRU', 'PC_06IRIY', 'PC_05W661', 'PC_06AI9C', 'PC_053J9J', 'PC_00915C', 'PC_06RYNI', 'PC_06OK2P', 'PC_03K305', 'PC_01FJ7Z', 'PC_036385', 'PC_07076R', 'PC_07UMX8', 'PC_04DQ0W', 'PC_02V4LH', 'PC_01WTO6', 'PC_02KEHU', 'PC_05FIVY', 'PC_02EV3G'}
MSA succeeded
Edge PC_009BF9 PC_06HSND 0.3835393190383911
lkey 187
lkey_clusters {'PC_021EBE', 'PC_06UZ06', 'PC_05L7US', 'PC_04KM1I', 'PC_06WVB2', 'PC_02GYSI', 'PC_01OH6B', 'PC_016FRE', 'PC_02QUA0', 'PC_0306GU', 'PC_01CVKZ', 'PC_002I3M', 'PC_04D7VO', 'PC_02C8S3', 'PC_03PSAN', 'PC_05MHF3', 'PC_009BF9', 'PC_05XT7X'}
MSA succeeded
Edge PC_03PEL5 PC_046RD9 0.3835098445415497
lkey 189
lkey_clusters {'PC_05ILWE', 'PC_06BP65', 'PC_02WCO0', 'PC_001RDT', 'PC_02OSJL', 'PC_07VAB2', 'PC_03JFL5', 'PC_06CM2C', 'PC_0384LZ', 'PC_00772B', 'PC_01K875', 'PC_05LDC6', 'PC_072EU7', 'PC_07XIBT', 'PC_04V0PU', 'PC_06FIXN', 'PC_03813O', 'PC_009036', 'PC_05K9FR', 'PC_03NC9U', 'PC_05QB64', 'PC_0860OA', 'PC_0804W2', 'PC_03PEL5', 'PC_04DUFN', 'PC_08I1Q6', 'PC_02UI5W', 'PC_074GF9', 'PC_00UWAQ', 'PC_02D1GK', 'PC_081PV4', 'PC_032CKH', 'PC_007Z8T', 'PC_02HVML', 'PC_07VHB5', 'PC_05TZZ7', 'PC_01FQRU', 'PC_06IRIY', 'PC_05W661', 'PC_06AI9C', 'PC_053J9J', 'PC_00915C', 'PC_06RYNI', 'PC_06OK2P', 'PC_03K305', 'PC_01FJ7Z', 'PC_036385', 'PC_07076R', 'PC_07UMX8', 'PC_04DQ0W', 'PC_02V4LH', 'PC_01WTO6', 'PC_02KEHU', 'PC_05FIVY', 'PC_02EV3G'}
MSA succeeded
Edge PC_05AOUJ PC_05MVJQ 0.38324254751205444
MSA succeeded
Edge PC_00NE3P PC_01CVKZ 0.38157379627227783
lkey 192
lkey_clusters {'PC_05AOUJ', 'PC_05MVJQ', 'PC_004U8P', 'PC_002I4B', 'PC_04THYL', 'PC_00NE3P'}
MSA succeeded
Edge PC_04IINX PC_04N53M 0.3796308636665344
MSA succeeded
Edge PC_001GNR PC_001RDT 0.377686083316803
MSA succeeded
Edge PC_06J307 PC_085L07 0.3739706873893738
lkey 177
lkey_clusters {'PC_06ZU26', 'PC_030G1S', 'PC_008806', 'PC_01IC1B', 'PC_06J307', 'PC_073NJA', 'PC_05KG1N', 'PC_01YHQS', 'PC_05OR8I', 'PC_021SYL', 'PC_02V4AV'}
MSA succeeded
Edge PC_02OSJL PC_02VB7G 0.3739093840122223
lkey 195
lkey_clusters {'PC_05ILWE', 'PC_06BP65', 'PC_02WCO0', 'PC_001RDT', 'PC_02OSJL', 'PC_07VAB2', 'PC_03JFL5', 'PC_06CM2C', 'PC_001GNR', 'PC_0384LZ', 'PC_00772B', 'PC_01K875', 'PC_05LDC6', 'PC_072EU7', 'PC_07XIBT', 'PC_04V0PU', 'PC_06FIXN', 'PC_046RD9', 'PC_03813O', 'PC_009036', 'PC_05K9FR', 'PC_03NC9U', 'PC_05QB64', 'PC_0860OA', 'PC_0804W2', 'PC_03PEL5', 'PC_04DUFN', 'PC_08I1Q6', 'PC_02UI5W', 'PC_074GF9', 'PC_00UWAQ', 'PC_02D1GK', 'PC_081PV4', 'PC_032CKH', 'PC_007Z8T', 'PC_02HVML', 'PC_07VHB5', 'PC_05TZZ7', 'PC_01FQRU', 'PC_06IRIY', 'PC_05W661', 'PC_06AI9C', 'PC_053J9J', 'PC_00915C', 'PC_06RYNI', 'PC_06OK2P', 'PC_03K305', 'PC_01FJ7Z', 'PC_036385', 'PC_07076R', 'PC_07UMX8', 'PC_04DQ0W', 'PC_02V4LH', 'PC_01WTO6', 'PC_02KEHU', 'PC_05FIVY', 'PC_02EV3G'}
MSA succeeded
Edge PC_04HZ79 PC_07TV02 0.37386929988861084
lkey 2
lkey_clusters {'PC_04HZ79', 'PC_01WPL7'}
MSA succeeded
Edge PC_03QNGV PC_07TV6Z 0.3715599775314331
MSA succeeded
Edge PC_05K4L1 PC_06WVB2 0.36788883805274963
MSA succeeded
Edge PC_01FQRU PC_02CEZD 0.36539748311042786
lkey 197
lkey_clusters {'PC_05ILWE', 'PC_06BP65', 'PC_02WCO0', 'PC_001RDT', 'PC_02OSJL', 'PC_07VAB2', 'PC_03JFL5', 'PC_06CM2C', 'PC_001GNR', 'PC_0384LZ', 'PC_00772B', 'PC_01K875', 'PC_05LDC6', 'PC_072EU7', 'PC_07XIBT', 'PC_04V0PU', 'PC_06FIXN', 'PC_046RD9', 'PC_03813O', 'PC_009036', 'PC_05K9FR', 'PC_03NC9U', 'PC_05QB64', 'PC_0860OA', 'PC_0804W2', 'PC_03PEL5', 'PC_04DUFN', 'PC_08I1Q6', 'PC_02UI5W', 'PC_074GF9', 'PC_00UWAQ', 'PC_02D1GK', 'PC_081PV4', 'PC_032CKH', 'PC_007Z8T', 'PC_02HVML', 'PC_07VHB5', 'PC_05TZZ7', 'PC_01FQRU', 'PC_06IRIY', 'PC_05W661', 'PC_06AI9C', 'PC_053J9J', 'PC_00915C', 'PC_06RYNI', 'PC_02VB7G', 'PC_06OK2P', 'PC_03K305', 'PC_01FJ7Z', 'PC_036385', 'PC_07076R', 'PC_07UMX8', 'PC_04DQ0W', 'PC_02V4LH', 'PC_01WTO6', 'PC_02KEHU', 'PC_05FIVY', 'PC_02EV3G'}
MSA succeeded
Edge PC_06AI9C PC_07QLAB 0.36337020993232727
lkey 201
lkey_clusters {'PC_05ILWE', 'PC_06BP65', 'PC_02WCO0', 'PC_001RDT', 'PC_02OSJL', 'PC_07VAB2', 'PC_03JFL5', 'PC_06CM2C', 'PC_001GNR', 'PC_0384LZ', 'PC_00772B', 'PC_01K875', 'PC_05LDC6', 'PC_072EU7', 'PC_07XIBT', 'PC_04V0PU', 'PC_06FIXN', 'PC_046RD9', 'PC_03813O', 'PC_009036', 'PC_05K9FR', 'PC_03NC9U', 'PC_05QB64', 'PC_0860OA', 'PC_0804W2', 'PC_03PEL5', 'PC_04DUFN', 'PC_08I1Q6', 'PC_02UI5W', 'PC_074GF9', 'PC_00UWAQ', 'PC_02D1GK', 'PC_081PV4', 'PC_032CKH', 'PC_007Z8T', 'PC_02HVML', 'PC_07VHB5', 'PC_05TZZ7', 'PC_01FQRU', 'PC_06IRIY', 'PC_05W661', 'PC_06AI9C', 'PC_053J9J', 'PC_00915C', 'PC_06RYNI', 'PC_02VB7G', 'PC_02CEZD', 'PC_06OK2P', 'PC_03K305', 'PC_01FJ7Z', 'PC_036385', 'PC_07076R', 'PC_07UMX8', 'PC_04DQ0W', 'PC_02V4LH', 'PC_01WTO6', 'PC_02KEHU', 'PC_05FIVY', 'PC_02EV3G'}
MSA succeeded
Edge PC_00HGKK PC_07UMX8 0.36278536915779114
MSA succeeded
Edge PC_01R1KR PC_03970R 0.36142265796661377
lkey 130
lkey_clusters {'PC_057VS9', 'PC_06RYIX', 'PC_06K136', 'PC_00DU1R', 'PC_051C8B', 'PC_00LBZX', 'PC_01R1KR'}
MSA succeeded
Edge PC_02EMSJ PC_08H9YG 0.36096686124801636
MSA succeeded
Edge PC_00ZQQF PC_030G1S 0.36039772629737854
MSA succeeded
Edge PC_01FFWH PC_03GTZD 0.3603401184082031
MSA succeeded
Edge PC_01GLB1 PC_02T3ZI 0.3568468689918518
MSA succeeded
Edge PC_029RUP PC_04PM5Y 0.35428866744041443
lkey 145
lkey_clusters {'PC_01PMZK', 'PC_029RUP', 'PC_00641O'}
MSA succeeded
Edge PC_005N2L PC_07076R 0.35381078720092773
MSA succeeded
Edge PC_027RDL PC_05QB64 0.35022398829460144
lkey 104
lkey_clusters {'PC_027RDL', 'PC_05MI4Z'}
MSA succeeded
Edge PC_00QNZJ PC_012M5T 0.3493455946445465
lkey 176
lkey_clusters {'PC_00QDYY', 'PC_0519TF', 'PC_024FEH', 'PC_02NATZ', 'PC_00D5EQ', 'PC_06PIV6', 'PC_05X98I', 'PC_003XGF', 'PC_00QNZJ', 'PC_07TUWD', 'PC_0287UU', 'PC_041GAL', 'PC_02Q6QH'}
MSA succeeded
Edge PC_01D3OM PC_022NID 0.34870731830596924
lkey 161
lkey_clusters {'PC_01D3OM', 'PC_07TV9U'}
MSA succeeded
Edge PC_03XWEE PC_06187I 0.3481367826461792
lkey 72
lkey_clusters {'PC_026AIN', 'PC_03XWEE'}
MSA succeeded
Edge PC_032CKH PC_04VBNR 0.3390423655509949
lkey 211
lkey_clusters {'PC_06BP65', 'PC_001RDT', 'PC_02OSJL', 'PC_001GNR', 'PC_0384LZ', 'PC_00772B', 'PC_01K875', 'PC_07XIBT', 'PC_04V0PU', 'PC_06FIXN', 'PC_009036', 'PC_05QB64', 'PC_0804W2', 'PC_04DUFN', 'PC_08I1Q6', 'PC_07QLAB', 'PC_074GF9', 'PC_081PV4', 'PC_02D1GK', 'PC_007Z8T', 'PC_032CKH', 'PC_02HVML', 'PC_07VHB5', 'PC_05TZZ7', 'PC_01FQRU', 'PC_05W661', 'PC_005N2L', 'PC_06RYNI', 'PC_02VB7G', 'PC_02CEZD', 'PC_06OK2P', 'PC_03K305', 'PC_05MI4Z', 'PC_027RDL', 'PC_036385', 'PC_07UMX8', 'PC_04DQ0W', 'PC_02V4LH', 'PC_02KEHU', 'PC_05ILWE', 'PC_02WCO0', 'PC_07VAB2', 'PC_06CM2C', 'PC_03JFL5', 'PC_05LDC6', 'PC_072EU7', 'PC_046RD9', 'PC_00HGKK', 'PC_03813O', 'PC_05K9FR', 'PC_03NC9U', 'PC_0860OA', 'PC_03PEL5', 'PC_02UI5W', 'PC_00UWAQ', 'PC_06IRIY', 'PC_06AI9C', 'PC_053J9J', 'PC_00915C', 'PC_01FJ7Z', 'PC_07076R', 'PC_01WTO6', 'PC_05FIVY', 'PC_02EV3G'}
MSA succeeded
Edge PC_00UFF9 PC_036HDQ 0.3387584686279297
MSA succeeded
Edge PC_00VTNG PC_05TFVL 0.3380095958709717
MSA succeeded
Edge PC_08I1Q6 PC_08I3IM 0.3375031054019928
lkey 215
lkey_clusters {'PC_06BP65', 'PC_001RDT', 'PC_02OSJL', 'PC_001GNR', 'PC_0384LZ', 'PC_00772B', 'PC_01K875', 'PC_07XIBT', 'PC_04V0PU', 'PC_06FIXN', 'PC_009036', 'PC_05QB64', 'PC_0804W2', 'PC_04DUFN', 'PC_08I1Q6', 'PC_07QLAB', 'PC_074GF9', 'PC_081PV4', 'PC_02D1GK', 'PC_007Z8T', 'PC_032CKH', 'PC_02HVML', 'PC_07VHB5', 'PC_05TZZ7', 'PC_01FQRU', 'PC_05W661', 'PC_005N2L', 'PC_06RYNI', 'PC_02VB7G', 'PC_02CEZD', 'PC_06OK2P', 'PC_03K305', 'PC_05MI4Z', 'PC_027RDL', 'PC_036385', 'PC_07UMX8', 'PC_04DQ0W', 'PC_02V4LH', 'PC_02KEHU', 'PC_05ILWE', 'PC_02WCO0', 'PC_07VAB2', 'PC_06CM2C', 'PC_03JFL5', 'PC_04VBNR', 'PC_05LDC6', 'PC_072EU7', 'PC_046RD9', 'PC_00HGKK', 'PC_03813O', 'PC_05K9FR', 'PC_03NC9U', 'PC_0860OA', 'PC_03PEL5', 'PC_02UI5W', 'PC_00UWAQ', 'PC_06IRIY', 'PC_06AI9C', 'PC_053J9J', 'PC_00915C', 'PC_01FJ7Z', 'PC_07076R', 'PC_01WTO6', 'PC_05FIVY', 'PC_02EV3G'}
MSA succeeded
Edge PC_00HGKK PC_04LTCQ 0.33570119738578796
lkey 218
lkey_clusters {'PC_06BP65', 'PC_001RDT', 'PC_02OSJL', 'PC_001GNR', 'PC_0384LZ', 'PC_00772B', 'PC_01K875', 'PC_07XIBT', 'PC_04V0PU', 'PC_06FIXN', 'PC_009036', 'PC_08I3IM', 'PC_05QB64', 'PC_0804W2', 'PC_04DUFN', 'PC_08I1Q6', 'PC_07QLAB', 'PC_074GF9', 'PC_081PV4', 'PC_02D1GK', 'PC_007Z8T', 'PC_032CKH', 'PC_02HVML', 'PC_07VHB5', 'PC_05TZZ7', 'PC_01FQRU', 'PC_05W661', 'PC_005N2L', 'PC_06RYNI', 'PC_02VB7G', 'PC_02CEZD', 'PC_06OK2P', 'PC_03K305', 'PC_05MI4Z', 'PC_027RDL', 'PC_036385', 'PC_07UMX8', 'PC_04DQ0W', 'PC_02V4LH', 'PC_02KEHU', 'PC_05ILWE', 'PC_02WCO0', 'PC_07VAB2', 'PC_06CM2C', 'PC_03JFL5', 'PC_04VBNR', 'PC_05LDC6', 'PC_072EU7', 'PC_046RD9', 'PC_00HGKK', 'PC_03813O', 'PC_05K9FR', 'PC_03NC9U', 'PC_0860OA', 'PC_03PEL5', 'PC_02UI5W', 'PC_00UWAQ', 'PC_06IRIY', 'PC_06AI9C', 'PC_053J9J', 'PC_00915C', 'PC_01FJ7Z', 'PC_07076R', 'PC_01WTO6', 'PC_05FIVY', 'PC_02EV3G'}
MSA succeeded
Edge PC_0085EC PC_02QMV0 0.3329360783100128
lkey 9
lkey_clusters {'PC_0085EC', 'PC_02BNS6'}
MSA succeeded
Edge PC_05X98I PC_064DUW 0.33155813813209534
lkey 212
lkey_clusters {'PC_00QDYY', 'PC_0519TF', 'PC_024FEH', 'PC_02NATZ', 'PC_00D5EQ', 'PC_06PIV6', 'PC_05X98I', 'PC_003XGF', 'PC_00QNZJ', 'PC_07TUWD', 'PC_0287UU', 'PC_041GAL', 'PC_01OCWE', 'PC_02Q6QH', 'PC_012M5T'}
MSA succeeded
Edge PC_01FQRU PC_07OCLR 0.3276141881942749
lkey 219
lkey_clusters {'PC_06BP65', 'PC_001RDT', 'PC_02OSJL', 'PC_001GNR', 'PC_0384LZ', 'PC_00772B', 'PC_01K875', 'PC_07XIBT', 'PC_04V0PU', 'PC_06FIXN', 'PC_009036', 'PC_08I3IM', 'PC_05QB64', 'PC_0804W2', 'PC_04DUFN', 'PC_08I1Q6', 'PC_07QLAB', 'PC_074GF9', 'PC_081PV4', 'PC_02D1GK', 'PC_007Z8T', 'PC_032CKH', 'PC_02HVML', 'PC_07VHB5', 'PC_05TZZ7', 'PC_01FQRU', 'PC_05W661', 'PC_005N2L', 'PC_06RYNI', 'PC_02VB7G', 'PC_02CEZD', 'PC_06OK2P', 'PC_03K305', 'PC_05MI4Z', 'PC_027RDL', 'PC_036385', 'PC_07UMX8', 'PC_04DQ0W', 'PC_02V4LH', 'PC_02KEHU', 'PC_05ILWE', 'PC_02WCO0', 'PC_07VAB2', 'PC_06CM2C', 'PC_03JFL5', 'PC_04VBNR', 'PC_05LDC6', 'PC_072EU7', 'PC_046RD9', 'PC_00HGKK', 'PC_03813O', 'PC_05K9FR', 'PC_03NC9U', 'PC_0860OA', 'PC_04LTCQ', 'PC_03PEL5', 'PC_02UI5W', 'PC_00UWAQ', 'PC_06IRIY', 'PC_06AI9C', 'PC_053J9J', 'PC_00915C', 'PC_01FJ7Z', 'PC_07076R', 'PC_01WTO6', 'PC_05FIVY', 'PC_02EV3G'}
MSA succeeded
Edge PC_02KEHU PC_060QAC 0.32723212242126465
lkey 222
lkey_clusters {'PC_06BP65', 'PC_001RDT', 'PC_02OSJL', 'PC_001GNR', 'PC_0384LZ', 'PC_00772B', 'PC_01K875', 'PC_07XIBT', 'PC_04V0PU', 'PC_06FIXN', 'PC_009036', 'PC_08I3IM', 'PC_05QB64', 'PC_0804W2', 'PC_04DUFN', 'PC_08I1Q6', 'PC_07QLAB', 'PC_074GF9', 'PC_081PV4', 'PC_02D1GK', 'PC_007Z8T', 'PC_032CKH', 'PC_02HVML', 'PC_07VHB5', 'PC_05TZZ7', 'PC_01FQRU', 'PC_05W661', 'PC_005N2L', 'PC_06RYNI', 'PC_02VB7G', 'PC_02CEZD', 'PC_06OK2P', 'PC_03K305', 'PC_05MI4Z', 'PC_027RDL', 'PC_036385', 'PC_07UMX8', 'PC_04DQ0W', 'PC_02V4LH', 'PC_02KEHU', 'PC_05ILWE', 'PC_02WCO0', 'PC_07VAB2', 'PC_06CM2C', 'PC_03JFL5', 'PC_04VBNR', 'PC_05LDC6', 'PC_072EU7', 'PC_046RD9', 'PC_00HGKK', 'PC_03813O', 'PC_05K9FR', 'PC_03NC9U', 'PC_0860OA', 'PC_04LTCQ', 'PC_03PEL5', 'PC_02UI5W', 'PC_00UWAQ', 'PC_06IRIY', 'PC_06AI9C', 'PC_053J9J', 'PC_00915C', 'PC_01FJ7Z', 'PC_07076R', 'PC_01WTO6', 'PC_07OCLR', 'PC_05FIVY', 'PC_02EV3G'}
MSA succeeded
Edge PC_02RGUO PC_05X98I 0.3268556296825409
MSA succeeded
Edge PC_02OSJL PC_05TX0J 0.32331904768943787
lkey 223
lkey_clusters {'PC_06BP65', 'PC_001RDT', 'PC_02OSJL', 'PC_001GNR', 'PC_0384LZ', 'PC_00772B', 'PC_060QAC', 'PC_01K875', 'PC_07XIBT', 'PC_04V0PU', 'PC_06FIXN', 'PC_009036', 'PC_08I3IM', 'PC_05QB64', 'PC_0804W2', 'PC_04DUFN', 'PC_08I1Q6', 'PC_07QLAB', 'PC_074GF9', 'PC_081PV4', 'PC_02D1GK', 'PC_007Z8T', 'PC_032CKH', 'PC_02HVML', 'PC_07VHB5', 'PC_05TZZ7', 'PC_01FQRU', 'PC_05W661', 'PC_005N2L', 'PC_06RYNI', 'PC_02VB7G', 'PC_02CEZD', 'PC_06OK2P', 'PC_03K305', 'PC_05MI4Z', 'PC_027RDL', 'PC_036385', 'PC_07UMX8', 'PC_04DQ0W', 'PC_02V4LH', 'PC_02KEHU', 'PC_05ILWE', 'PC_02WCO0', 'PC_07VAB2', 'PC_06CM2C', 'PC_03JFL5', 'PC_04VBNR', 'PC_05LDC6', 'PC_072EU7', 'PC_046RD9', 'PC_00HGKK', 'PC_03813O', 'PC_05K9FR', 'PC_03NC9U', 'PC_0860OA', 'PC_04LTCQ', 'PC_03PEL5', 'PC_02UI5W', 'PC_00UWAQ', 'PC_06IRIY', 'PC_06AI9C', 'PC_053J9J', 'PC_00915C', 'PC_01FJ7Z', 'PC_07076R', 'PC_02UBXP', 'PC_01WTO6', 'PC_07OCLR', 'PC_05FIVY', 'PC_02EV3G'}
MSA succeeded
Edge PC_045HU3 PC_06EWH0 0.31701138615608215
MSA succeeded
Edge PC_02XK62 PC_06280D 0.31657448410987854
MSA succeeded
Edge PC_02OSJL PC_063TMD 0.3124024271965027
lkey 225
lkey_clusters {'PC_06BP65', 'PC_001RDT', 'PC_02OSJL', 'PC_001GNR', 'PC_0384LZ', 'PC_00772B', 'PC_060QAC', 'PC_01K875', 'PC_07XIBT', 'PC_04V0PU', 'PC_06FIXN', 'PC_009036', 'PC_08I3IM', 'PC_05QB64', 'PC_0804W2', 'PC_04DUFN', 'PC_08I1Q6', 'PC_07QLAB', 'PC_074GF9', 'PC_081PV4', 'PC_02D1GK', 'PC_007Z8T', 'PC_032CKH', 'PC_02HVML', 'PC_07VHB5', 'PC_05TZZ7', 'PC_01FQRU', 'PC_05W661', 'PC_005N2L', 'PC_06RYNI', 'PC_02VB7G', 'PC_02CEZD', 'PC_06OK2P', 'PC_03K305', 'PC_05MI4Z', 'PC_027RDL', 'PC_036385', 'PC_07UMX8', 'PC_04DQ0W', 'PC_02V4LH', 'PC_02KEHU', 'PC_05TX0J', 'PC_05ILWE', 'PC_02WCO0', 'PC_07VAB2', 'PC_06CM2C', 'PC_03JFL5', 'PC_04VBNR', 'PC_05LDC6', 'PC_072EU7', 'PC_046RD9', 'PC_00HGKK', 'PC_03813O', 'PC_05K9FR', 'PC_03NC9U', 'PC_0860OA', 'PC_04LTCQ', 'PC_03PEL5', 'PC_02UI5W', 'PC_00UWAQ', 'PC_06IRIY', 'PC_06AI9C', 'PC_053J9J', 'PC_00915C', 'PC_01FJ7Z', 'PC_07076R', 'PC_02UBXP', 'PC_01WTO6', 'PC_07OCLR', 'PC_05FIVY', 'PC_02EV3G'}
MSA succeeded
Edge PC_07WEO1 PC_08H11O 0.3107548654079437
lkey 155
lkey_clusters {'PC_05AJOI', 'PC_07WEO1'}
MSA succeeded
Edge PC_04U1L7 PC_05FXKZ 0.31004148721694946
MSA succeeded
Edge PC_04PJ1H PC_060UD5 0.30934327840805054
MSA succeeded
Edge PC_007Z8T PC_05R7KZ 0.3008981943130493
lkey 228
lkey_clusters {'PC_06BP65', 'PC_001RDT', 'PC_02OSJL', 'PC_001GNR', 'PC_0384LZ', 'PC_00772B', 'PC_060QAC', 'PC_01K875', 'PC_07XIBT', 'PC_04V0PU', 'PC_06FIXN', 'PC_009036', 'PC_08I3IM', 'PC_05QB64', 'PC_0804W2', 'PC_04DUFN', 'PC_08I1Q6', 'PC_07QLAB', 'PC_074GF9', 'PC_081PV4', 'PC_02D1GK', 'PC_007Z8T', 'PC_032CKH', 'PC_02HVML', 'PC_07VHB5', 'PC_05TZZ7', 'PC_01FQRU', 'PC_05W661', 'PC_005N2L', 'PC_06RYNI', 'PC_02VB7G', 'PC_02CEZD', 'PC_06OK2P', 'PC_03K305', 'PC_05MI4Z', 'PC_027RDL', 'PC_036385', 'PC_07UMX8', 'PC_04DQ0W', 'PC_02V4LH', 'PC_02KEHU', 'PC_05TX0J', 'PC_05ILWE', 'PC_02WCO0', 'PC_07VAB2', 'PC_06CM2C', 'PC_03JFL5', 'PC_04VBNR', 'PC_05LDC6', 'PC_072EU7', 'PC_046RD9', 'PC_00HGKK', 'PC_03813O', 'PC_05K9FR', 'PC_03NC9U', 'PC_063TMD', 'PC_0860OA', 'PC_04LTCQ', 'PC_03PEL5', 'PC_02UI5W', 'PC_00UWAQ', 'PC_06IRIY', 'PC_06AI9C', 'PC_053J9J', 'PC_00915C', 'PC_01FJ7Z', 'PC_07076R', 'PC_02UBXP', 'PC_01WTO6', 'PC_07OCLR', 'PC_024L0V', 'PC_05FIVY', 'PC_02EV3G'}
MSA succeeded
Edge PC_04ZZ0B PC_05FXKZ 0.3006322979927063
MSA succeeded
Edge PC_04U1L7 PC_07WEO1 0.29874923825263977
lkey 233
lkey_clusters {'PC_04ZZ0B', 'PC_011LAW', 'PC_05F9H4', 'PC_030ACC', 'PC_03EHDR', 'PC_0100UP', 'PC_04U1L7', 'PC_05FXKZ', 'PC_039ZP9'}
MSA succeeded
Edge PC_01FQRU PC_04PSFQ 0.29829853773117065
lkey 232
lkey_clusters {'PC_06BP65', 'PC_001RDT', 'PC_02OSJL', 'PC_001GNR', 'PC_0384LZ', 'PC_00772B', 'PC_060QAC', 'PC_01K875', 'PC_07XIBT', 'PC_04V0PU', 'PC_06FIXN', 'PC_009036', 'PC_08I3IM', 'PC_05QB64', 'PC_0804W2', 'PC_04DUFN', 'PC_08I1Q6', 'PC_07QLAB', 'PC_074GF9', 'PC_081PV4', 'PC_02D1GK', 'PC_007Z8T', 'PC_032CKH', 'PC_02HVML', 'PC_07VHB5', 'PC_05TZZ7', 'PC_01FQRU', 'PC_05W661', 'PC_005N2L', 'PC_06RYNI', 'PC_02VB7G', 'PC_02CEZD', 'PC_06OK2P', 'PC_03K305', 'PC_05MI4Z', 'PC_027RDL', 'PC_036385', 'PC_07UMX8', 'PC_04DQ0W', 'PC_02V4LH', 'PC_02KEHU', 'PC_05TX0J', 'PC_05ILWE', 'PC_02WCO0', 'PC_07VAB2', 'PC_06CM2C', 'PC_03JFL5', 'PC_04VBNR', 'PC_05R7KZ', 'PC_05LDC6', 'PC_072EU7', 'PC_046RD9', 'PC_00HGKK', 'PC_03813O', 'PC_05K9FR', 'PC_03NC9U', 'PC_063TMD', 'PC_0860OA', 'PC_04LTCQ', 'PC_03PEL5', 'PC_02UI5W', 'PC_00UWAQ', 'PC_06IRIY', 'PC_06AI9C', 'PC_053J9J', 'PC_00915C', 'PC_01FJ7Z', 'PC_07076R', 'PC_02UBXP', 'PC_01WTO6', 'PC_07OCLR', 'PC_024L0V', 'PC_05FIVY', 'PC_02EV3G'}
MSA succeeded
Edge PC_03T6PW PC_087UOG 0.29583483934402466
MSA succeeded
Edge PC_029YIZ PC_02RGUO 0.2917341887950897
lkey 63
lkey_clusters {'PC_03FKVQ', 'PC_029YIZ'}
MSA succeeded
Edge PC_02V4LH PC_0473PG 0.2888838052749634
lkey 235
lkey_clusters {'PC_06BP65', 'PC_001RDT', 'PC_02OSJL', 'PC_001GNR', 'PC_0384LZ', 'PC_00772B', 'PC_060QAC', 'PC_01K875', 'PC_07XIBT', 'PC_04V0PU', 'PC_06FIXN', 'PC_009036', 'PC_08I3IM', 'PC_05QB64', 'PC_0804W2', 'PC_04DUFN', 'PC_08I1Q6', 'PC_07QLAB', 'PC_074GF9', 'PC_081PV4', 'PC_02D1GK', 'PC_007Z8T', 'PC_032CKH', 'PC_02HVML', 'PC_07VHB5', 'PC_05TZZ7', 'PC_01FQRU', 'PC_05W661', 'PC_005N2L', 'PC_06RYNI', 'PC_02VB7G', 'PC_02CEZD', 'PC_06OK2P', 'PC_03K305', 'PC_05MI4Z', 'PC_027RDL', 'PC_036385', 'PC_07UMX8', 'PC_04DQ0W', 'PC_02V4LH', 'PC_02KEHU', 'PC_05TX0J', 'PC_05ILWE', 'PC_02WCO0', 'PC_07VAB2', 'PC_06CM2C', 'PC_03JFL5', 'PC_04VBNR', 'PC_05R7KZ', 'PC_05LDC6', 'PC_072EU7', 'PC_046RD9', 'PC_00HGKK', 'PC_03813O', 'PC_05K9FR', 'PC_03NC9U', 'PC_063TMD', 'PC_0860OA', 'PC_04LTCQ', 'PC_03PEL5', 'PC_02UI5W', 'PC_04PSFQ', 'PC_00UWAQ', 'PC_06IRIY', 'PC_06AI9C', 'PC_053J9J', 'PC_00915C', 'PC_01FJ7Z', 'PC_07076R', 'PC_02UBXP', 'PC_01WTO6', 'PC_07OCLR', 'PC_024L0V', 'PC_05FIVY', 'PC_02EV3G'}
MSA succeeded
Edge PC_04BA1R PC_0804W2 0.2837305963039398
MSA failed
Edge PC_02EMSJ PC_06CM2C 0.28368082642555237
lkey 205
lkey_clusters {'PC_08H9YG', 'PC_02EMSJ'}
MSA succeeded
Edge PC_0181PE PC_02KEHU 0.2819480299949646
MSA succeeded
Edge PC_04USMD PC_04VBNR 0.27842390537261963
MSA succeeded
Edge PC_04SK9M PC_06EWH0 0.27728742361068726
MSA succeeded
Edge PC_01CDCN PC_073NJA 0.2747020721435547
MSA succeeded
Edge PC_00MO21 PC_03JFL5 0.2724088728427887
MSA succeeded
Edge PC_02W8LT PC_04USMD 0.27057376503944397
MSA succeeded
Edge PC_02QMV0 PC_07TUPN 0.26580995321273804
lkey 231
lkey_clusters {'PC_04PJ1H', 'PC_01EAHK', 'PC_02BNS6', 'PC_060UD5', 'PC_0085EC', 'PC_063IUN', 'PC_02QMV0'}
MSA succeeded
Edge PC_007Z8T PC_04N53M 0.2607945203781128
lkey 245
lkey_clusters {'PC_05FIVY', 'PC_06BP65', 'PC_001RDT', 'PC_02OSJL', 'PC_001GNR', 'PC_0384LZ', 'PC_00772B', 'PC_060QAC', 'PC_01K875', 'PC_07XIBT', 'PC_04V0PU', 'PC_06FIXN', 'PC_009036', 'PC_08I3IM', 'PC_05QB64', 'PC_0804W2', 'PC_04DUFN', 'PC_08I1Q6', 'PC_08H9YG', 'PC_07QLAB', 'PC_074GF9', 'PC_081PV4', 'PC_02D1GK', 'PC_007Z8T', 'PC_032CKH', 'PC_02HVML', 'PC_07VHB5', 'PC_05TZZ7', 'PC_01FQRU', 'PC_05W661', 'PC_02W8LT', 'PC_005N2L', 'PC_06RYNI', 'PC_02VB7G', 'PC_02CEZD', 'PC_06OK2P', 'PC_03K305', 'PC_05MI4Z', 'PC_00MO21', 'PC_027RDL', 'PC_036385', 'PC_07UMX8', 'PC_04DQ0W', 'PC_02V4LH', 'PC_02KEHU', 'PC_05ILWE', 'PC_02WCO0', 'PC_07VAB2', 'PC_06CM2C', 'PC_03JFL5', 'PC_04VBNR', 'PC_05R7KZ', 'PC_05LDC6', 'PC_072EU7', 'PC_046RD9', 'PC_00HGKK', 'PC_03813O', 'PC_05K9FR', 'PC_03NC9U', 'PC_063TMD', 'PC_0473PG', 'PC_0860OA', 'PC_04LTCQ', 'PC_03PEL5', 'PC_02UI5W', 'PC_0181PE', 'PC_04PSFQ', 'PC_00UWAQ', 'PC_02EMSJ', 'PC_06IRIY', 'PC_06AI9C', 'PC_053J9J', 'PC_00915C', 'PC_01FJ7Z', 'PC_07076R', 'PC_02UBXP', 'PC_04USMD', 'PC_01WTO6', 'PC_07OCLR', 'PC_024L0V', 'PC_05TX0J', 'PC_02EV3G'}
MSA succeeded
Edge PC_02EV3G PC_05MNXH 0.255221426486969
lkey 247
lkey_clusters {'PC_05FIVY', 'PC_06BP65', 'PC_001RDT', 'PC_02OSJL', 'PC_04IINX', 'PC_001GNR', 'PC_0384LZ', 'PC_00772B', 'PC_060QAC', 'PC_01K875', 'PC_07XIBT', 'PC_04V0PU', 'PC_06FIXN', 'PC_009036', 'PC_08I3IM', 'PC_05QB64', 'PC_0804W2', 'PC_04DUFN', 'PC_08I1Q6', 'PC_08H9YG', 'PC_07QLAB', 'PC_074GF9', 'PC_081PV4', 'PC_02D1GK', 'PC_007Z8T', 'PC_032CKH', 'PC_02HVML', 'PC_07VHB5', 'PC_05TZZ7', 'PC_01FQRU', 'PC_05W661', 'PC_02W8LT', 'PC_005N2L', 'PC_06RYNI', 'PC_02VB7G', 'PC_02CEZD', 'PC_06OK2P', 'PC_03K305', 'PC_05MI4Z', 'PC_00MO21', 'PC_027RDL', 'PC_036385', 'PC_07UMX8', 'PC_04DQ0W', 'PC_02V4LH', 'PC_02KEHU', 'PC_05ILWE', 'PC_02WCO0', 'PC_07VAB2', 'PC_06CM2C', 'PC_03JFL5', 'PC_04VBNR', 'PC_05R7KZ', 'PC_05LDC6', 'PC_072EU7', 'PC_046RD9', 'PC_00HGKK', 'PC_03813O', 'PC_05K9FR', 'PC_03NC9U', 'PC_063TMD', 'PC_0473PG', 'PC_0860OA', 'PC_04LTCQ', 'PC_03PEL5', 'PC_02UI5W', 'PC_0181PE', 'PC_04PSFQ', 'PC_00UWAQ', 'PC_02EMSJ', 'PC_06IRIY', 'PC_06AI9C', 'PC_053J9J', 'PC_00915C', 'PC_04N53M', 'PC_01FJ7Z', 'PC_07076R', 'PC_02UBXP', 'PC_04USMD', 'PC_01WTO6', 'PC_07OCLR', 'PC_024L0V', 'PC_05TX0J', 'PC_02EV3G'}
MSA succeeded
Edge PC_01296N PC_02EV3G 0.25369325280189514
lkey 236
lkey_clusters {'PC_088DQF', 'PC_00NN46', 'PC_01296N', 'PC_087UOG', 'PC_07AGJQ', 'PC_0370DG', 'PC_03T6PW', 'PC_017F5K'}
MSA succeeded
Edge PC_02QW19 PC_07TUPN 0.2530005872249603
lkey 103
lkey_clusters {'PC_02QW19', 'PC_04SFDZ'}
MSA succeeded
Edge PC_01W301 PC_04U1L7 0.2521600127220154
MSA succeeded
Edge PC_05VZ2W PC_07TV6Z 0.25179868936538696
lkey 67
lkey_clusters {'PC_0109ZP', 'PC_05VZ2W', 'PC_01VGHN'}
MSA succeeded
Edge PC_07AWUL PC_07WEO1 0.2509147822856903
MSA succeeded
Edge PC_00UFF9 PC_04V0PU 0.2495640367269516
lkey 216
lkey_clusters {'PC_00UFF9', 'PC_036HDQ'}
MSA succeeded
Edge PC_02OSJL PC_07D4R9 0.2487017810344696
lkey 254
lkey_clusters {'PC_05FIVY', 'PC_06BP65', 'PC_001RDT', 'PC_02OSJL', 'PC_04IINX', 'PC_001GNR', 'PC_0384LZ', 'PC_00772B', 'PC_060QAC', 'PC_01K875', 'PC_017F5K', 'PC_07XIBT', 'PC_04V0PU', 'PC_06FIXN', 'PC_009036', 'PC_08I3IM', 'PC_05QB64', 'PC_01296N', 'PC_0804W2', 'PC_04DUFN', 'PC_08I1Q6', 'PC_08H9YG', 'PC_07QLAB', 'PC_074GF9', 'PC_00UFF9', 'PC_081PV4', 'PC_02D1GK', 'PC_007Z8T', 'PC_032CKH', 'PC_05MNXH', 'PC_02HVML', 'PC_07VHB5', 'PC_05TZZ7', 'PC_01FQRU', 'PC_05W661', 'PC_02W8LT', 'PC_005N2L', 'PC_06RYNI', 'PC_02VB7G', 'PC_02CEZD', 'PC_06OK2P', 'PC_03K305', 'PC_05MI4Z', 'PC_00MO21', 'PC_027RDL', 'PC_036385', 'PC_07UMX8', 'PC_04DQ0W', 'PC_02V4LH', 'PC_02KEHU', 'PC_07AGJQ', 'PC_05ILWE', 'PC_088DQF', 'PC_036HDQ', 'PC_02WCO0', 'PC_07VAB2', 'PC_06CM2C', 'PC_03JFL5', 'PC_04VBNR', 'PC_05R7KZ', 'PC_05LDC6', 'PC_072EU7', 'PC_03T6PW', 'PC_046RD9', 'PC_00HGKK', 'PC_03813O', 'PC_05K9FR', 'PC_00NN46', 'PC_03NC9U', 'PC_063TMD', 'PC_0473PG', 'PC_0860OA', 'PC_04LTCQ', 'PC_087UOG', 'PC_03PEL5', 'PC_02UI5W', 'PC_0370DG', 'PC_0181PE', 'PC_04PSFQ', 'PC_00UWAQ', 'PC_02EMSJ', 'PC_06IRIY', 'PC_06AI9C', 'PC_053J9J', 'PC_00915C', 'PC_04N53M', 'PC_01FJ7Z', 'PC_07076R', 'PC_02UBXP', 'PC_04USMD', 'PC_01WTO6', 'PC_07OCLR', 'PC_024L0V', 'PC_05TX0J', 'PC_02EV3G'}
MSA succeeded
Edge PC_032CKH PC_07VJ0Q 0.24770864844322205
lkey 255
lkey_clusters {'PC_05FIVY', 'PC_06BP65', 'PC_001RDT', 'PC_02OSJL', 'PC_04IINX', 'PC_001GNR', 'PC_0384LZ', 'PC_00772B', 'PC_060QAC', 'PC_01K875', 'PC_017F5K', 'PC_07XIBT', 'PC_04V0PU', 'PC_06FIXN', 'PC_009036', 'PC_08I3IM', 'PC_05QB64', 'PC_01296N', 'PC_0804W2', 'PC_04DUFN', 'PC_08I1Q6', 'PC_08H9YG', 'PC_07QLAB', 'PC_074GF9', 'PC_00UFF9', 'PC_081PV4', 'PC_02D1GK', 'PC_007Z8T', 'PC_032CKH', 'PC_05MNXH', 'PC_02HVML', 'PC_07VHB5', 'PC_05TZZ7', 'PC_01FQRU', 'PC_05W661', 'PC_02W8LT', 'PC_005N2L', 'PC_07D4R9', 'PC_06RYNI', 'PC_02VB7G', 'PC_02CEZD', 'PC_06OK2P', 'PC_03K305', 'PC_05MI4Z', 'PC_00MO21', 'PC_027RDL', 'PC_036385', 'PC_07UMX8', 'PC_04DQ0W', 'PC_02V4LH', 'PC_02KEHU', 'PC_07AGJQ', 'PC_05ILWE', 'PC_088DQF', 'PC_036HDQ', 'PC_02WCO0', 'PC_07VAB2', 'PC_06CM2C', 'PC_03JFL5', 'PC_04VBNR', 'PC_05R7KZ', 'PC_05LDC6', 'PC_072EU7', 'PC_03T6PW', 'PC_046RD9', 'PC_00HGKK', 'PC_03813O', 'PC_05K9FR', 'PC_00NN46', 'PC_03NC9U', 'PC_063TMD', 'PC_0473PG', 'PC_0860OA', 'PC_04LTCQ', 'PC_087UOG', 'PC_03PEL5', 'PC_02UI5W', 'PC_0370DG', 'PC_0181PE', 'PC_04PSFQ', 'PC_00UWAQ', 'PC_02EMSJ', 'PC_06IRIY', 'PC_06AI9C', 'PC_053J9J', 'PC_00915C', 'PC_04N53M', 'PC_01FJ7Z', 'PC_07076R', 'PC_02UBXP', 'PC_04USMD', 'PC_01WTO6', 'PC_07OCLR', 'PC_024L0V', 'PC_05TX0J', 'PC_02EV3G'}
MSA succeeded
Edge PC_02EMSJ PC_0843Z9 0.24690723419189453
lkey 256
lkey_clusters {'PC_05FIVY', 'PC_06BP65', 'PC_001RDT', 'PC_02OSJL', 'PC_04IINX', 'PC_001GNR', 'PC_0384LZ', 'PC_00772B', 'PC_060QAC', 'PC_01K875', 'PC_017F5K', 'PC_07XIBT', 'PC_04V0PU', 'PC_06FIXN', 'PC_009036', 'PC_08I3IM', 'PC_05QB64', 'PC_01296N', 'PC_0804W2', 'PC_04DUFN', 'PC_08I1Q6', 'PC_08H9YG', 'PC_07QLAB', 'PC_074GF9', 'PC_00UFF9', 'PC_081PV4', 'PC_02D1GK', 'PC_007Z8T', 'PC_032CKH', 'PC_05MNXH', 'PC_02HVML', 'PC_07VHB5', 'PC_05TZZ7', 'PC_01FQRU', 'PC_05W661', 'PC_02W8LT', 'PC_005N2L', 'PC_07D4R9', 'PC_06RYNI', 'PC_02VB7G', 'PC_02CEZD', 'PC_06OK2P', 'PC_03K305', 'PC_05MI4Z', 'PC_00MO21', 'PC_027RDL', 'PC_036385', 'PC_07UMX8', 'PC_04DQ0W', 'PC_02V4LH', 'PC_02KEHU', 'PC_07AGJQ', 'PC_05ILWE', 'PC_088DQF', 'PC_036HDQ', 'PC_02WCO0', 'PC_07VAB2', 'PC_06CM2C', 'PC_03JFL5', 'PC_04VBNR', 'PC_05R7KZ', 'PC_07VJ0Q', 'PC_05LDC6', 'PC_072EU7', 'PC_03T6PW', 'PC_046RD9', 'PC_00HGKK', 'PC_03813O', 'PC_05K9FR', 'PC_00NN46', 'PC_03NC9U', 'PC_063TMD', 'PC_0473PG', 'PC_0860OA', 'PC_04LTCQ', 'PC_087UOG', 'PC_03PEL5', 'PC_02UI5W', 'PC_0370DG', 'PC_0181PE', 'PC_04PSFQ', 'PC_00UWAQ', 'PC_02EMSJ', 'PC_06IRIY', 'PC_06AI9C', 'PC_053J9J', 'PC_00915C', 'PC_04N53M', 'PC_01FJ7Z', 'PC_07076R', 'PC_02UBXP', 'PC_04USMD', 'PC_01WTO6', 'PC_07OCLR', 'PC_024L0V', 'PC_05TX0J', 'PC_02EV3G'}
MSA succeeded
Edge PC_00LSXD PC_05FXKZ 0.24619752168655396
lkey 169
lkey_clusters {'PC_0837FY', 'PC_00LSXD', 'PC_021V0R'}
MSA succeeded
Edge PC_0181PE PC_023QTD 0.24538688361644745
lkey 257
lkey_clusters {'PC_05FIVY', 'PC_06BP65', 'PC_001RDT', 'PC_02OSJL', 'PC_04IINX', 'PC_001GNR', 'PC_0384LZ', 'PC_00772B', 'PC_060QAC', 'PC_01K875', 'PC_017F5K', 'PC_07XIBT', 'PC_04V0PU', 'PC_06FIXN', 'PC_009036', 'PC_08I3IM', 'PC_05QB64', 'PC_01296N', 'PC_0804W2', 'PC_04DUFN', 'PC_08I1Q6', 'PC_08H9YG', 'PC_0843Z9', 'PC_07QLAB', 'PC_074GF9', 'PC_00UFF9', 'PC_081PV4', 'PC_02D1GK', 'PC_007Z8T', 'PC_032CKH', 'PC_05MNXH', 'PC_0753N9', 'PC_02HVML', 'PC_07VHB5', 'PC_05TZZ7', 'PC_01FQRU', 'PC_05N2GV', 'PC_05W661', 'PC_02W8LT', 'PC_005N2L', 'PC_07D4R9', 'PC_06RYNI', 'PC_02VB7G', 'PC_02CEZD', 'PC_06OK2P', 'PC_03K305', 'PC_05MI4Z', 'PC_00MO21', 'PC_027RDL', 'PC_036385', 'PC_07UMX8', 'PC_04DQ0W', 'PC_02V4LH', 'PC_02KEHU', 'PC_07AGJQ', 'PC_05ILWE', 'PC_088DQF', 'PC_036HDQ', 'PC_02WCO0', 'PC_07VAB2', 'PC_06CM2C', 'PC_03JFL5', 'PC_04VBNR', 'PC_05R7KZ', 'PC_07VJ0Q', 'PC_05LDC6', 'PC_072EU7', 'PC_03T6PW', 'PC_046RD9', 'PC_00HGKK', 'PC_03813O', 'PC_05K9FR', 'PC_00NN46', 'PC_03NC9U', 'PC_063TMD', 'PC_0473PG', 'PC_0860OA', 'PC_04LTCQ', 'PC_087UOG', 'PC_03PEL5', 'PC_02UI5W', 'PC_0370DG', 'PC_0181PE', 'PC_04PSFQ', 'PC_00UWAQ', 'PC_02EMSJ', 'PC_06IRIY', 'PC_06AI9C', 'PC_053J9J', 'PC_00915C', 'PC_04N53M', 'PC_01FJ7Z', 'PC_07076R', 'PC_02UBXP', 'PC_04USMD', 'PC_01WTO6', 'PC_07OCLR', 'PC_024L0V', 'PC_05TX0J', 'PC_02EV3G'}
MSA succeeded
Edge PC_02BG1Z PC_07UMX8 0.2393054962158203
MSA succeeded
Edge PC_04UWXG PC_05RVL4 0.2372058629989624
MSA succeeded
Edge PC_00UAB7 PC_04E4JE 0.23556247353553772
MSA succeeded
Edge PC_011686 PC_0804W2 0.23292087018489838
MSA succeeded
Edge PC_00UAB7 PC_04UWXG 0.22930088639259338
lkey 262
lkey_clusters {'PC_04E4JE', 'PC_06BL39', 'PC_00UAB7'}
MSA succeeded
Edge PC_02CC4R PC_02QMV0 0.22842665016651154
MSA succeeded
Edge PC_02NISQ PC_03G9RN 0.2280837893486023
MSA succeeded
Edge PC_030G1S PC_07VAB2 0.22640259563922882
lkey 243
lkey_clusters {'PC_06ZU26', 'PC_030G1S', 'PC_085L07', 'PC_008806', 'PC_01IC1B', 'PC_06J307', 'PC_073NJA', 'PC_01CDCN', 'PC_05KG1N', 'PC_00ZQQF', 'PC_01YHQS', 'PC_05OR8I', 'PC_021SYL', 'PC_02V4AV'}
MSA succeeded
Edge PC_001RDT PC_02G1YM 0.22013825178146362
lkey 267
lkey_clusters {'PC_085L07', 'PC_06BP65', 'PC_001RDT', 'PC_02OSJL', 'PC_04IINX', 'PC_001GNR', 'PC_073NJA', 'PC_0384LZ', 'PC_00772B', 'PC_060QAC', 'PC_01K875', 'PC_07XIBT', 'PC_04V0PU', 'PC_06ZU26', 'PC_06FIXN', 'PC_009036', 'PC_08I3IM', 'PC_05QB64', 'PC_01296N', 'PC_0804W2', 'PC_04DUFN', 'PC_00ZQQF', 'PC_021SYL', 'PC_08I1Q6', 'PC_08H9YG', 'PC_011686', 'PC_0843Z9', 'PC_07QLAB', 'PC_074GF9', 'PC_00UFF9', 'PC_081PV4', 'PC_02D1GK', 'PC_007Z8T', 'PC_032CKH', 'PC_05MNXH', 'PC_0753N9', 'PC_02HVML', 'PC_07VHB5', 'PC_05TZZ7', 'PC_01FQRU', 'PC_05N2GV', 'PC_05W661', 'PC_02W8LT', 'PC_05KG1N', 'PC_005N2L', 'PC_023QTD', 'PC_07D4R9', 'PC_06RYNI', 'PC_02VB7G', 'PC_02CEZD', 'PC_06OK2P', 'PC_03K305', 'PC_05MI4Z', 'PC_00MO21', 'PC_027RDL', 'PC_036385', 'PC_07UMX8', 'PC_04DQ0W', 'PC_02V4LH', 'PC_02KEHU', 'PC_05TX0J', 'PC_07AGJQ', 'PC_05OR8I', 'PC_05ILWE', 'PC_088DQF', 'PC_030G1S', 'PC_036HDQ', 'PC_02WCO0', 'PC_07VAB2', 'PC_06CM2C', 'PC_03JFL5', 'PC_04VBNR', 'PC_01CDCN', 'PC_05R7KZ', 'PC_07VJ0Q', 'PC_02V4AV', 'PC_05LDC6', 'PC_072EU7', 'PC_03T6PW', 'PC_046RD9', 'PC_00HGKK', 'PC_03813O', 'PC_05K9FR', 'PC_00NN46', 'PC_03NC9U', 'PC_063TMD', 'PC_0473PG', 'PC_0860OA', 'PC_04LTCQ', 'PC_06J307', 'PC_087UOG', 'PC_03PEL5', 'PC_02UI5W', 'PC_0370DG', 'PC_0181PE', 'PC_04PSFQ', 'PC_00UWAQ', 'PC_02EMSJ', 'PC_02BG1Z', 'PC_008806', 'PC_06IRIY', 'PC_06AI9C', 'PC_053J9J', 'PC_01YHQS', 'PC_00915C', 'PC_02EV3G', 'PC_04N53M', 'PC_01FJ7Z', 'PC_07076R', 'PC_02UBXP', 'PC_04USMD', 'PC_01WTO6', 'PC_01IC1B', 'PC_07OCLR', 'PC_024L0V', 'PC_05FIVY', 'PC_017F5K'}
MSA succeeded
Edge PC_001RDT PC_0708R1 0.21551914513111115
lkey 268
lkey_clusters {'PC_085L07', 'PC_06BP65', 'PC_001RDT', 'PC_02OSJL', 'PC_04IINX', 'PC_001GNR', 'PC_073NJA', 'PC_0384LZ', 'PC_00772B', 'PC_060QAC', 'PC_01K875', 'PC_07XIBT', 'PC_04V0PU', 'PC_06ZU26', 'PC_06FIXN', 'PC_009036', 'PC_08I3IM', 'PC_05QB64', 'PC_01296N', 'PC_0804W2', 'PC_04DUFN', 'PC_00ZQQF', 'PC_021SYL', 'PC_08I1Q6', 'PC_08H9YG', 'PC_011686', 'PC_0843Z9', 'PC_07QLAB', 'PC_074GF9', 'PC_00UFF9', 'PC_081PV4', 'PC_02D1GK', 'PC_007Z8T', 'PC_032CKH', 'PC_05MNXH', 'PC_0753N9', 'PC_02HVML', 'PC_07VHB5', 'PC_05TZZ7', 'PC_01FQRU', 'PC_05N2GV', 'PC_05W661', 'PC_02W8LT', 'PC_05KG1N', 'PC_005N2L', 'PC_023QTD', 'PC_07D4R9', 'PC_06RYNI', 'PC_02VB7G', 'PC_02CEZD', 'PC_06OK2P', 'PC_03K305', 'PC_05MI4Z', 'PC_00MO21', 'PC_027RDL', 'PC_036385', 'PC_07UMX8', 'PC_04DQ0W', 'PC_02V4LH', 'PC_02KEHU', 'PC_05TX0J', 'PC_07AGJQ', 'PC_05OR8I', 'PC_05ILWE', 'PC_088DQF', 'PC_030G1S', 'PC_036HDQ', 'PC_02WCO0', 'PC_07VAB2', 'PC_06CM2C', 'PC_03JFL5', 'PC_04VBNR', 'PC_01CDCN', 'PC_05R7KZ', 'PC_07VJ0Q', 'PC_02V4AV', 'PC_05LDC6', 'PC_072EU7', 'PC_03T6PW', 'PC_046RD9', 'PC_00HGKK', 'PC_03813O', 'PC_05K9FR', 'PC_00NN46', 'PC_03NC9U', 'PC_063TMD', 'PC_0473PG', 'PC_0860OA', 'PC_04LTCQ', 'PC_06J307', 'PC_087UOG', 'PC_03PEL5', 'PC_02UI5W', 'PC_0370DG', 'PC_0181PE', 'PC_04PSFQ', 'PC_00UWAQ', 'PC_02EMSJ', 'PC_02BG1Z', 'PC_02G1YM', 'PC_008806', 'PC_06IRIY', 'PC_06AI9C', 'PC_053J9J', 'PC_01YHQS', 'PC_00915C', 'PC_02EV3G', 'PC_04N53M', 'PC_01FJ7Z', 'PC_07076R', 'PC_02UBXP', 'PC_04USMD', 'PC_01WTO6', 'PC_01IC1B', 'PC_07OCLR', 'PC_024L0V', 'PC_05FIVY', 'PC_017F5K'}
MSA succeeded
Edge PC_02P3RH PC_06EWH0 0.21165214478969574
lkey 5
lkey_clusters {'PC_02P3RH', 'PC_06SE34'}
MSA succeeded
Edge PC_00595C PC_05VOPA 0.20122264325618744
lkey 182
lkey_clusters {'PC_00595C', 'PC_01QQ1T', 'PC_01KTUJ', 'PC_00646F', 'PC_073XSY'}
MSA succeeded
Edge PC_018DM6 PC_07TV5U 0.2005576342344284
lkey 153
lkey_clusters {'PC_018DM6', 'PC_04MR4A', 'PC_0278ZT', 'PC_07TV5W', 'PC_06UZ3U'}
MSA succeeded
Edge PC_018GH8 PC_05W6XP 0.2000497728586197
lkey 49
lkey_clusters {'PC_018GH8', 'PC_059TG9', 'PC_06MGDC'}
MSA succeeded
Edge PC_04L2GD PC_05X98I 0.19951380789279938
MSA succeeded
Edge PC_01FGFJ PC_02KYVT 0.1965625137090683
MSA succeeded
Edge PC_00HTJV PC_05QB64 0.19620397686958313
MSA succeeded
Edge PC_01D3OM PC_07TUPN 0.1953260451555252
lkey 213
lkey_clusters {'PC_01D3OM', 'PC_02R0M8', 'PC_07TV9U', 'PC_022NID'}
MSA succeeded
Edge PC_0393P5 PC_060UD5 0.1914254128932953
MSA succeeded
Edge PC_00641O PC_05MHF3 0.18913820385932922
lkey 209
lkey_clusters {'PC_01PMZK', 'PC_04PM5Y', 'PC_029RUP', 'PC_00641O'}
MSA succeeded
Edge PC_00YPBK PC_05VOPA 0.1840716153383255
MSA succeeded
Edge PC_02LGPA PC_04SK9M 0.18143923580646515
MSA succeeded
Edge PC_032CKH PC_05FXKZ 0.17991381883621216
lkey 276
lkey_clusters {'PC_085L07', 'PC_05FIVY', 'PC_06BP65', 'PC_001RDT', 'PC_02OSJL', 'PC_04IINX', 'PC_001GNR', 'PC_073NJA', 'PC_0384LZ', 'PC_00772B', 'PC_060QAC', 'PC_01K875', 'PC_0708R1', 'PC_017F5K', 'PC_07XIBT', 'PC_04V0PU', 'PC_06ZU26', 'PC_06FIXN', 'PC_009036', 'PC_08I3IM', 'PC_05QB64', 'PC_01296N', 'PC_0804W2', 'PC_04DUFN', 'PC_00ZQQF', 'PC_021SYL', 'PC_08I1Q6', 'PC_08H9YG', 'PC_011686', 'PC_0843Z9', 'PC_07QLAB', 'PC_074GF9', 'PC_00UFF9', 'PC_081PV4', 'PC_02D1GK', 'PC_007Z8T', 'PC_032CKH', 'PC_05MNXH', 'PC_0753N9', 'PC_02HVML', 'PC_07VHB5', 'PC_05TZZ7', 'PC_01FQRU', 'PC_05N2GV', 'PC_05W661', 'PC_02W8LT', 'PC_05KG1N', 'PC_005N2L', 'PC_023QTD', 'PC_07D4R9', 'PC_06RYNI', 'PC_02VB7G', 'PC_02CEZD', 'PC_06OK2P', 'PC_03K305', 'PC_05MI4Z', 'PC_00MO21', 'PC_027RDL', 'PC_036385', 'PC_07UMX8', 'PC_04DQ0W', 'PC_02V4LH', 'PC_02KEHU', 'PC_07AGJQ', 'PC_05OR8I', 'PC_05ILWE', 'PC_088DQF', 'PC_030G1S', 'PC_036HDQ', 'PC_02WCO0', 'PC_07VAB2', 'PC_06CM2C', 'PC_03JFL5', 'PC_04VBNR', 'PC_01CDCN', 'PC_05R7KZ', 'PC_07VJ0Q', 'PC_02V4AV', 'PC_05LDC6', 'PC_072EU7', 'PC_03T6PW', 'PC_00HTJV', 'PC_046RD9', 'PC_00HGKK', 'PC_03813O', 'PC_05K9FR', 'PC_00NN46', 'PC_03NC9U', 'PC_063TMD', 'PC_0473PG', 'PC_0860OA', 'PC_04LTCQ', 'PC_06J307', 'PC_087UOG', 'PC_03PEL5', 'PC_02UI5W', 'PC_0370DG', 'PC_0181PE', 'PC_04PSFQ', 'PC_00UWAQ', 'PC_02EMSJ', 'PC_02BG1Z', 'PC_02G1YM', 'PC_008806', 'PC_06IRIY', 'PC_06AI9C', 'PC_053J9J', 'PC_01YHQS', 'PC_00915C', 'PC_04N53M', 'PC_01FJ7Z', 'PC_07076R', 'PC_02UBXP', 'PC_04USMD', 'PC_01WTO6', 'PC_01IC1B', 'PC_07OCLR', 'PC_024L0V', 'PC_05TX0J', 'PC_02EV3G'}
MSA succeeded
Edge PC_05KEYA PC_07TUPN 0.1787593960762024
MSA succeeded
Edge PC_009LO1 PC_03870X 0.1777118742465973
MSA succeeded
Edge PC_02DAU3 PC_05VOPA 0.1763565093278885
MSA succeeded
Edge PC_03870X PC_07TUPN 0.17483800649642944
lkey 284
lkey_clusters {'PC_009LO1', 'PC_03870X'}
MSA succeeded
Edge PC_05O6MK PC_05TX0J 0.17454399168491364
MSA succeeded
Edge PC_00595C PC_02QIDH 0.1743154078722
lkey 285
lkey_clusters {'PC_00595C', 'PC_02DAU3', 'PC_01QQ1T', 'PC_01KTUJ', 'PC_00YPBK', 'PC_05VOPA', 'PC_00646F', 'PC_073XSY'}
MSA succeeded
Edge PC_02WCO0 PC_03E0CS 0.1724577099084854
lkey 287
lkey_clusters {'PC_06BP65', 'PC_04IINX', 'PC_001GNR', 'PC_0384LZ', 'PC_00772B', 'PC_060QAC', 'PC_01GNRM', 'PC_01K875', 'PC_042YRE', 'PC_04V0PU', 'PC_05QB64', 'PC_01296N', 'PC_04DUFN', 'PC_00ZQQF', 'PC_08I1Q6', 'PC_08H9YG', 'PC_0843Z9', 'PC_07QLAB', 'PC_039ZP9', 'PC_081PV4', 'PC_007Z8T', 'PC_032CKH', 'PC_05MNXH', 'PC_02HVML', 'PC_07VHB5', 'PC_01FQRU', 'PC_02W8LT', 'PC_05KG1N', 'PC_005N2L', 'PC_06RYNI', 'PC_02CEZD', 'PC_07WEO1', 'PC_06OK2P', 'PC_00MO21', 'PC_04DQ0W', 'PC_04U1L7', 'PC_05OR8I', 'PC_05FXKZ', 'PC_07AWUL', 'PC_088DQF', 'PC_036HDQ', 'PC_07VAB2', 'PC_04VBNR', 'PC_01CDCN', 'PC_05R7KZ', 'PC_02V4AV', 'PC_05LDC6', 'PC_072EU7', 'PC_00HTJV', 'PC_046RD9', 'PC_00HGKK', 'PC_03813O', 'PC_05K9FR', 'PC_03NC9U', 'PC_0860OA', 'PC_04LTCQ', 'PC_0370DG', 'PC_04PSFQ', 'PC_00UWAQ', 'PC_02EMSJ', 'PC_02BG1Z', 'PC_008806', 'PC_06IRIY', 'PC_053J9J', 'PC_02EV3G', 'PC_04N53M', 'PC_08H11O', 'PC_02UBXP', 'PC_05F9H4', 'PC_021V0R', 'PC_01WTO6', 'PC_07OCLR', 'PC_05FIVY', 'PC_085L07', 'PC_001RDT', 'PC_02OSJL', 'PC_073NJA', 'PC_0708R1', 'PC_00LSXD', 'PC_07XIBT', 'PC_06ZU26', 'PC_06FIXN', 'PC_009036', 'PC_08I3IM', 'PC_05AJOI', 'PC_0804W2', 'PC_021SYL', 'PC_011686', 'PC_0837FY', 'PC_074GF9', 'PC_00UFF9', 'PC_02D1GK', 'PC_05N2GV', 'PC_0753N9', 'PC_02G1YM', 'PC_03EHDR', 'PC_05TZZ7', 'PC_023QTD', 'PC_05W661', 'PC_07D4R9', 'PC_02VB7G', 'PC_05O6MK', 'PC_03K305', 'PC_05MI4Z', 'PC_027RDL', 'PC_036385', 'PC_011LAW', 'PC_04OIFF', 'PC_07UMX8', 'PC_030ACC', 'PC_02V4LH', 'PC_02KEHU', 'PC_07AGJQ', 'PC_01W301', 'PC_02JM6C', 'PC_05ILWE', 'PC_030G1S', 'PC_02WCO0', 'PC_06CM2C', 'PC_03JFL5', 'PC_07VJ0Q', 'PC_03T6PW', 'PC_07UIKR', 'PC_0473PG', 'PC_00NN46', 'PC_063TMD', 'PC_06J307', 'PC_087UOG', 'PC_03PEL5', 'PC_02UI5W', 'PC_0181PE', 'PC_0100UP', 'PC_06AI9C', 'PC_00M7NJ', 'PC_01YHQS', 'PC_00915C', 'PC_01FJ7Z', 'PC_04ZZ0B', 'PC_07076R', 'PC_04USMD', 'PC_01IC1B', 'PC_024L0V', 'PC_05TX0J', 'PC_017F5K'}
MSA succeeded
Edge PC_01WPL7 PC_05DIQW 0.16989123821258545
lkey 198
lkey_clusters {'PC_04HZ79', 'PC_01WPL7', 'PC_07TV02'}
MSA succeeded
Edge PC_02AR9H PC_081PV4 0.16931332647800446
MSA succeeded
Edge PC_05D714 PC_0654DH 0.1687280535697937
MSA succeeded
Edge PC_06SL6B PC_07UMX8 0.1644914448261261
MSA succeeded
Edge PC_03PBKY PC_06J307 0.16342411935329437
MSA succeeded
Edge PC_02LGPA PC_07TVA5 0.16170145571231842
lkey 292
lkey_clusters {'PC_02P3RH', 'PC_05RS5N', 'PC_045HU3', 'PC_06EWH0', 'PC_0654DH', 'PC_02LGPA', 'PC_06SE34', 'PC_05D714', 'PC_04SK9M'}
MSA succeeded
Edge PC_032CKH PC_07NWKC 0.15928982198238373
lkey 294
lkey_clusters {'PC_06BP65', 'PC_04IINX', 'PC_001GNR', 'PC_0384LZ', 'PC_00772B', 'PC_060QAC', 'PC_01GNRM', 'PC_01K875', 'PC_042YRE', 'PC_017F5K', 'PC_04V0PU', 'PC_05QB64', 'PC_01296N', 'PC_04DUFN', 'PC_00ZQQF', 'PC_08I1Q6', 'PC_08H9YG', 'PC_0843Z9', 'PC_07QLAB', 'PC_039ZP9', 'PC_081PV4', 'PC_007Z8T', 'PC_032CKH', 'PC_05MNXH', 'PC_02HVML', 'PC_07VHB5', 'PC_01FQRU', 'PC_02W8LT', 'PC_05KG1N', 'PC_005N2L', 'PC_06RYNI', 'PC_02CEZD', 'PC_07WEO1', 'PC_06OK2P', 'PC_00MO21', 'PC_04DQ0W', 'PC_04U1L7', 'PC_05OR8I', 'PC_05FXKZ', 'PC_07AWUL', 'PC_088DQF', 'PC_036HDQ', 'PC_07VAB2', 'PC_04VBNR', 'PC_01CDCN', 'PC_05R7KZ', 'PC_02V4AV', 'PC_05LDC6', 'PC_072EU7', 'PC_00HTJV', 'PC_046RD9', 'PC_03E0CS', 'PC_00HGKK', 'PC_03813O', 'PC_05K9FR', 'PC_03NC9U', 'PC_0860OA', 'PC_04LTCQ', 'PC_0370DG', 'PC_04PSFQ', 'PC_00UWAQ', 'PC_02EMSJ', 'PC_02BG1Z', 'PC_008806', 'PC_06IRIY', 'PC_053J9J', 'PC_04N53M', 'PC_08H11O', 'PC_02UBXP', 'PC_05F9H4', 'PC_021V0R', 'PC_01WTO6', 'PC_07OCLR', 'PC_05FIVY', 'PC_085L07', 'PC_001RDT', 'PC_02OSJL', 'PC_03PBKY', 'PC_073NJA', 'PC_0708R1', 'PC_00LSXD', 'PC_07XIBT', 'PC_06ZU26', 'PC_06FIXN', 'PC_009036', 'PC_08I3IM', 'PC_05AJOI', 'PC_0804W2', 'PC_021SYL', 'PC_011686', 'PC_02AR9H', 'PC_0837FY', 'PC_074GF9', 'PC_00UFF9', 'PC_02D1GK', 'PC_05N2GV', 'PC_0753N9', 'PC_02G1YM', 'PC_03EHDR', 'PC_05TZZ7', 'PC_023QTD', 'PC_05W661', 'PC_07D4R9', 'PC_02VB7G', 'PC_05O6MK', 'PC_03K305', 'PC_05MI4Z', 'PC_027RDL', 'PC_036385', 'PC_011LAW', 'PC_04OIFF', 'PC_07UMX8', 'PC_030ACC', 'PC_02V4LH', 'PC_02KEHU', 'PC_07AGJQ', 'PC_01W301', 'PC_02JM6C', 'PC_05ILWE', 'PC_030G1S', 'PC_02WCO0', 'PC_06CM2C', 'PC_03JFL5', 'PC_07VJ0Q', 'PC_03T6PW', 'PC_07UIKR', 'PC_0473PG', 'PC_00NN46', 'PC_063TMD', 'PC_06J307', 'PC_087UOG', 'PC_03PEL5', 'PC_02UI5W', 'PC_0181PE', 'PC_06SL6B', 'PC_0100UP', 'PC_06AI9C', 'PC_00M7NJ', 'PC_01YHQS', 'PC_00915C', 'PC_01FJ7Z', 'PC_04ZZ0B', 'PC_07076R', 'PC_04USMD', 'PC_01IC1B', 'PC_024L0V', 'PC_05TX0J', 'PC_02EV3G'}
MSA succeeded
Edge PC_06RYIX PC_074DQF 0.15545877814292908
lkey 204
lkey_clusters {'PC_057VS9', 'PC_06RYIX', 'PC_00LBZX', 'PC_01R1KR', 'PC_03970R', 'PC_06K136', 'PC_00DU1R', 'PC_051C8B', 'PC_05W9ZE'}
MSA succeeded
Edge PC_02JQ3K PC_074DQF 0.15465320646762848
MSA succeeded
Edge PC_01IC1B PC_022ZLX 0.15180838108062744
lkey 296
lkey_clusters {'PC_06BP65', 'PC_04IINX', 'PC_001GNR', 'PC_0384LZ', 'PC_00772B', 'PC_060QAC', 'PC_01GNRM', 'PC_01K875', 'PC_042YRE', 'PC_017F5K', 'PC_04V0PU', 'PC_05QB64', 'PC_01296N', 'PC_04DUFN', 'PC_00ZQQF', 'PC_08I1Q6', 'PC_08H9YG', 'PC_0843Z9', 'PC_07QLAB', 'PC_039ZP9', 'PC_081PV4', 'PC_007Z8T', 'PC_032CKH', 'PC_05MNXH', 'PC_02HVML', 'PC_07VHB5', 'PC_01FQRU', 'PC_02W8LT', 'PC_05KG1N', 'PC_005N2L', 'PC_06RYNI', 'PC_02CEZD', 'PC_07WEO1', 'PC_06OK2P', 'PC_00MO21', 'PC_04DQ0W', 'PC_04U1L7', 'PC_05OR8I', 'PC_05FXKZ', 'PC_07AWUL', 'PC_088DQF', 'PC_036HDQ', 'PC_07VAB2', 'PC_04VBNR', 'PC_01CDCN', 'PC_05R7KZ', 'PC_02V4AV', 'PC_05LDC6', 'PC_072EU7', 'PC_00HTJV', 'PC_046RD9', 'PC_03E0CS', 'PC_00HGKK', 'PC_03813O', 'PC_05K9FR', 'PC_03NC9U', 'PC_0860OA', 'PC_04LTCQ', 'PC_0370DG', 'PC_04PSFQ', 'PC_00UWAQ', 'PC_02EMSJ', 'PC_02BG1Z', 'PC_008806', 'PC_06IRIY', 'PC_053J9J', 'PC_04N53M', 'PC_08H11O', 'PC_02UBXP', 'PC_05F9H4', 'PC_021V0R', 'PC_01WTO6', 'PC_07OCLR', 'PC_05FIVY', 'PC_085L07', 'PC_001RDT', 'PC_02OSJL', 'PC_03PBKY', 'PC_073NJA', 'PC_0708R1', 'PC_00LSXD', 'PC_07XIBT', 'PC_06ZU26', 'PC_06FIXN', 'PC_009036', 'PC_08I3IM', 'PC_05AJOI', 'PC_0804W2', 'PC_021SYL', 'PC_011686', 'PC_02AR9H', 'PC_0837FY', 'PC_074GF9', 'PC_00UFF9', 'PC_02D1GK', 'PC_05N2GV', 'PC_0753N9', 'PC_02G1YM', 'PC_03EHDR', 'PC_05TZZ7', 'PC_023QTD', 'PC_05W661', 'PC_07D4R9', 'PC_02VB7G', 'PC_05O6MK', 'PC_03K305', 'PC_05MI4Z', 'PC_027RDL', 'PC_036385', 'PC_011LAW', 'PC_04OIFF', 'PC_07UMX8', 'PC_030ACC', 'PC_02V4LH', 'PC_02KEHU', 'PC_07AGJQ', 'PC_01W301', 'PC_02JM6C', 'PC_05ILWE', 'PC_030G1S', 'PC_07NWKC', 'PC_02WCO0', 'PC_06CM2C', 'PC_03JFL5', 'PC_07VJ0Q', 'PC_03T6PW', 'PC_07UIKR', 'PC_0473PG', 'PC_00NN46', 'PC_063TMD', 'PC_06J307', 'PC_087UOG', 'PC_03PEL5', 'PC_02UI5W', 'PC_0181PE', 'PC_06SL6B', 'PC_0100UP', 'PC_06AI9C', 'PC_00M7NJ', 'PC_01YHQS', 'PC_00915C', 'PC_01FJ7Z', 'PC_04ZZ0B', 'PC_07076R', 'PC_04USMD', 'PC_01IC1B', 'PC_024L0V', 'PC_05TX0J', 'PC_02EV3G'}
MSA succeeded
Edge PC_032CKH PC_07A9N7 0.1516880989074707
lkey 299
lkey_clusters {'PC_06BP65', 'PC_04IINX', 'PC_001GNR', 'PC_0384LZ', 'PC_00772B', 'PC_060QAC', 'PC_01GNRM', 'PC_01K875', 'PC_042YRE', 'PC_017F5K', 'PC_04V0PU', 'PC_05QB64', 'PC_01296N', 'PC_04DUFN', 'PC_00ZQQF', 'PC_08I1Q6', 'PC_08H9YG', 'PC_0843Z9', 'PC_07QLAB', 'PC_039ZP9', 'PC_081PV4', 'PC_007Z8T', 'PC_032CKH', 'PC_05MNXH', 'PC_02HVML', 'PC_07VHB5', 'PC_01FQRU', 'PC_02W8LT', 'PC_05KG1N', 'PC_005N2L', 'PC_06RYNI', 'PC_02CEZD', 'PC_07WEO1', 'PC_06OK2P', 'PC_00MO21', 'PC_04DQ0W', 'PC_04U1L7', 'PC_05OR8I', 'PC_05FXKZ', 'PC_07AWUL', 'PC_088DQF', 'PC_036HDQ', 'PC_07VAB2', 'PC_04VBNR', 'PC_01CDCN', 'PC_05R7KZ', 'PC_02V4AV', 'PC_05LDC6', 'PC_072EU7', 'PC_00HTJV', 'PC_046RD9', 'PC_03E0CS', 'PC_00HGKK', 'PC_03813O', 'PC_05K9FR', 'PC_03NC9U', 'PC_0860OA', 'PC_04LTCQ', 'PC_0370DG', 'PC_04PSFQ', 'PC_00UWAQ', 'PC_02EMSJ', 'PC_02BG1Z', 'PC_008806', 'PC_06IRIY', 'PC_053J9J', 'PC_04N53M', 'PC_08H11O', 'PC_02UBXP', 'PC_05F9H4', 'PC_022ZLX', 'PC_021V0R', 'PC_01WTO6', 'PC_07OCLR', 'PC_05FIVY', 'PC_085L07', 'PC_001RDT', 'PC_02OSJL', 'PC_03PBKY', 'PC_073NJA', 'PC_0708R1', 'PC_00LSXD', 'PC_07XIBT', 'PC_06ZU26', 'PC_06FIXN', 'PC_009036', 'PC_08I3IM', 'PC_05AJOI', 'PC_0804W2', 'PC_021SYL', 'PC_011686', 'PC_02AR9H', 'PC_0837FY', 'PC_074GF9', 'PC_00UFF9', 'PC_02D1GK', 'PC_05N2GV', 'PC_0753N9', 'PC_02G1YM', 'PC_03EHDR', 'PC_05TZZ7', 'PC_023QTD', 'PC_05W661', 'PC_07D4R9', 'PC_02VB7G', 'PC_05O6MK', 'PC_03K305', 'PC_05MI4Z', 'PC_027RDL', 'PC_036385', 'PC_011LAW', 'PC_04OIFF', 'PC_07UMX8', 'PC_030ACC', 'PC_02V4LH', 'PC_02KEHU', 'PC_07AGJQ', 'PC_01W301', 'PC_02JM6C', 'PC_05ILWE', 'PC_030G1S', 'PC_07NWKC', 'PC_02WCO0', 'PC_06CM2C', 'PC_03JFL5', 'PC_07VJ0Q', 'PC_03T6PW', 'PC_07UIKR', 'PC_0473PG', 'PC_00NN46', 'PC_063TMD', 'PC_06J307', 'PC_087UOG', 'PC_03PEL5', 'PC_02UI5W', 'PC_0181PE', 'PC_06SL6B', 'PC_0100UP', 'PC_06AI9C', 'PC_00M7NJ', 'PC_01YHQS', 'PC_00915C', 'PC_01FJ7Z', 'PC_04ZZ0B', 'PC_07076R', 'PC_04USMD', 'PC_01IC1B', 'PC_024L0V', 'PC_05TX0J', 'PC_02EV3G'}
MSA succeeded
Edge PC_04U1L7 PC_062QHL 0.14806488156318665
lkey 300
lkey_clusters {'PC_06BP65', 'PC_07A9N7', 'PC_04IINX', 'PC_001GNR', 'PC_0384LZ', 'PC_00772B', 'PC_060QAC', 'PC_01GNRM', 'PC_01K875', 'PC_042YRE', 'PC_017F5K', 'PC_04V0PU', 'PC_05QB64', 'PC_01296N', 'PC_04DUFN', 'PC_00ZQQF', 'PC_08I1Q6', 'PC_08H9YG', 'PC_0843Z9', 'PC_07QLAB', 'PC_039ZP9', 'PC_081PV4', 'PC_007Z8T', 'PC_032CKH', 'PC_05MNXH', 'PC_02HVML', 'PC_07VHB5', 'PC_01FQRU', 'PC_02W8LT', 'PC_05KG1N', 'PC_005N2L', 'PC_06RYNI', 'PC_02CEZD', 'PC_07WEO1', 'PC_06OK2P', 'PC_00MO21', 'PC_04DQ0W', 'PC_04U1L7', 'PC_05OR8I', 'PC_05FXKZ', 'PC_07AWUL', 'PC_088DQF', 'PC_036HDQ', 'PC_07VAB2', 'PC_04VBNR', 'PC_01CDCN', 'PC_05R7KZ', 'PC_02V4AV', 'PC_05LDC6', 'PC_072EU7', 'PC_00HTJV', 'PC_046RD9', 'PC_03E0CS', 'PC_00HGKK', 'PC_03813O', 'PC_05K9FR', 'PC_03NC9U', 'PC_0860OA', 'PC_04LTCQ', 'PC_0370DG', 'PC_04PSFQ', 'PC_00UWAQ', 'PC_02EMSJ', 'PC_02BG1Z', 'PC_008806', 'PC_06IRIY', 'PC_053J9J', 'PC_04N53M', 'PC_08H11O', 'PC_02UBXP', 'PC_05F9H4', 'PC_022ZLX', 'PC_021V0R', 'PC_01WTO6', 'PC_07OCLR', 'PC_05FIVY', 'PC_085L07', 'PC_001RDT', 'PC_02OSJL', 'PC_03PBKY', 'PC_073NJA', 'PC_0708R1', 'PC_00LSXD', 'PC_07XIBT', 'PC_06ZU26', 'PC_06FIXN', 'PC_009036', 'PC_08I3IM', 'PC_05AJOI', 'PC_0804W2', 'PC_021SYL', 'PC_011686', 'PC_02AR9H', 'PC_0837FY', 'PC_074GF9', 'PC_00UFF9', 'PC_02D1GK', 'PC_05N2GV', 'PC_0753N9', 'PC_02G1YM', 'PC_03EHDR', 'PC_05TZZ7', 'PC_023QTD', 'PC_05W661', 'PC_07D4R9', 'PC_02VB7G', 'PC_05O6MK', 'PC_03K305', 'PC_05MI4Z', 'PC_027RDL', 'PC_036385', 'PC_011LAW', 'PC_04OIFF', 'PC_07UMX8', 'PC_030ACC', 'PC_02V4LH', 'PC_02KEHU', 'PC_07AGJQ', 'PC_01W301', 'PC_02JM6C', 'PC_05ILWE', 'PC_030G1S', 'PC_07NWKC', 'PC_02WCO0', 'PC_06CM2C', 'PC_03JFL5', 'PC_07VJ0Q', 'PC_03T6PW', 'PC_07UIKR', 'PC_0473PG', 'PC_00NN46', 'PC_063TMD', 'PC_06J307', 'PC_087UOG', 'PC_03PEL5', 'PC_02UI5W', 'PC_0181PE', 'PC_06SL6B', 'PC_0100UP', 'PC_06AI9C', 'PC_00M7NJ', 'PC_01YHQS', 'PC_00915C', 'PC_01FJ7Z', 'PC_04ZZ0B', 'PC_07076R', 'PC_04USMD', 'PC_01IC1B', 'PC_024L0V', 'PC_05TX0J', 'PC_02EV3G'}
MSA succeeded
Edge PC_063JSC PC_0654DH 0.14728374779224396
lkey 143
lkey_clusters {'PC_04FB23', 'PC_063JSC'}
MSA succeeded
Edge PC_06ADE4 PC_07UIKR 0.14687128365039825
MSA succeeded
Edge PC_032CKH PC_079ZWS 0.14491404592990875
lkey 303
lkey_clusters {'PC_06BP65', 'PC_07A9N7', 'PC_04IINX', 'PC_001GNR', 'PC_0384LZ', 'PC_00772B', 'PC_060QAC', 'PC_01GNRM', 'PC_01K875', 'PC_042YRE', 'PC_04V0PU', 'PC_05QB64', 'PC_01296N', 'PC_04DUFN', 'PC_00ZQQF', 'PC_08I1Q6', 'PC_08H9YG', 'PC_0843Z9', 'PC_07QLAB', 'PC_039ZP9', 'PC_081PV4', 'PC_007Z8T', 'PC_032CKH', 'PC_05MNXH', 'PC_02HVML', 'PC_07VHB5', 'PC_01FQRU', 'PC_02W8LT', 'PC_05KG1N', 'PC_005N2L', 'PC_06RYNI', 'PC_02CEZD', 'PC_07WEO1', 'PC_06OK2P', 'PC_00MO21', 'PC_04DQ0W', 'PC_04U1L7', 'PC_05OR8I', 'PC_05FXKZ', 'PC_07AWUL', 'PC_088DQF', 'PC_036HDQ', 'PC_07VAB2', 'PC_04VBNR', 'PC_01CDCN', 'PC_05R7KZ', 'PC_02V4AV', 'PC_05LDC6', 'PC_072EU7', 'PC_00HTJV', 'PC_046RD9', 'PC_03E0CS', 'PC_00HGKK', 'PC_03813O', 'PC_05K9FR', 'PC_03NC9U', 'PC_0860OA', 'PC_04LTCQ', 'PC_0370DG', 'PC_04PSFQ', 'PC_00UWAQ', 'PC_02EMSJ', 'PC_02BG1Z', 'PC_008806', 'PC_06IRIY', 'PC_053J9J', 'PC_02EV3G', 'PC_04N53M', 'PC_08H11O', 'PC_02UBXP', 'PC_05F9H4', 'PC_022ZLX', 'PC_021V0R', 'PC_01WTO6', 'PC_07OCLR', 'PC_05FIVY', 'PC_085L07', 'PC_001RDT', 'PC_02OSJL', 'PC_03PBKY', 'PC_073NJA', 'PC_062QHL', 'PC_0708R1', 'PC_00LSXD', 'PC_07XIBT', 'PC_06ZU26', 'PC_06FIXN', 'PC_009036', 'PC_08I3IM', 'PC_05AJOI', 'PC_0804W2', 'PC_021SYL', 'PC_011686', 'PC_02AR9H', 'PC_0837FY', 'PC_074GF9', 'PC_00UFF9', 'PC_02D1GK', 'PC_05N2GV', 'PC_0753N9', 'PC_02G1YM', 'PC_03EHDR', 'PC_05TZZ7', 'PC_023QTD', 'PC_05W661', 'PC_07D4R9', 'PC_02VB7G', 'PC_05O6MK', 'PC_03K305', 'PC_05MI4Z', 'PC_027RDL', 'PC_036385', 'PC_011LAW', 'PC_04OIFF', 'PC_07UMX8', 'PC_030ACC', 'PC_02V4LH', 'PC_02KEHU', 'PC_07AGJQ', 'PC_01W301', 'PC_02JM6C', 'PC_05ILWE', 'PC_030G1S', 'PC_07NWKC', 'PC_02WCO0', 'PC_06CM2C', 'PC_03JFL5', 'PC_07VJ0Q', 'PC_03T6PW', 'PC_06ADE4', 'PC_07UIKR', 'PC_0473PG', 'PC_00NN46', 'PC_063TMD', 'PC_06J307', 'PC_087UOG', 'PC_03PEL5', 'PC_02UI5W', 'PC_0181PE', 'PC_06SL6B', 'PC_0100UP', 'PC_06AI9C', 'PC_00M7NJ', 'PC_01YHQS', 'PC_00915C', 'PC_01FJ7Z', 'PC_04ZZ0B', 'PC_07076R', 'PC_04USMD', 'PC_01IC1B', 'PC_024L0V', 'PC_05TX0J', 'PC_017F5K'}
MSA succeeded
Edge PC_00A54M PC_0843Z9 0.14392875134944916
MSA succeeded
Edge PC_00QNZJ PC_03GTZD 0.14373934268951416
lkey 274
lkey_clusters {'PC_04L2GD', 'PC_0519TF', 'PC_024FEH', 'PC_02RGUO', 'PC_02NATZ', 'PC_05X98I', 'PC_07TUWD', 'PC_041GAL', 'PC_02Q6QH', 'PC_012M5T', 'PC_029YIZ', 'PC_00QDYY', 'PC_03FKVQ', 'PC_00D5EQ', 'PC_06PIV6', 'PC_003XGF', 'PC_00QNZJ', 'PC_0287UU', 'PC_01OCWE', 'PC_064DUW'}
MSA succeeded
Edge PC_048XFW PC_07TVM3 0.1436084657907486
MSA succeeded
Edge PC_057FE7 PC_06EWH0 0.143285870552063
MSA succeeded
Edge PC_00646F PC_03AR0I 0.14265266060829163
lkey 288
lkey_clusters {'PC_02QIDH', 'PC_00595C', 'PC_02DAU3', 'PC_07TV4U', 'PC_01QQ1T', 'PC_01KTUJ', 'PC_00YPBK', 'PC_05VOPA', 'PC_00646F', 'PC_073XSY'}
MSA succeeded
Edge PC_05VZ2W PC_074DQF 0.14168763160705566
lkey 252
lkey_clusters {'PC_03QNGV', 'PC_07TV6Z', 'PC_0109ZP', 'PC_05VZ2W', 'PC_01VGHN'}
MSA succeeded
Edge PC_00A3UY PC_0654DH 0.14116087555885315
lkey 307
lkey_clusters {'PC_02K5A5', 'PC_00A3UY', 'PC_02GRBP', 'PC_07TVM3', 'PC_048XFW', 'PC_057CGE', 'PC_07TV8B'}
MSA succeeded
Edge PC_05W661 PC_080PLJ 0.1405811309814453
lkey 305
lkey_clusters {'PC_06BP65', 'PC_07A9N7', 'PC_04IINX', 'PC_001GNR', 'PC_0384LZ', 'PC_00772B', 'PC_060QAC', 'PC_01GNRM', 'PC_01K875', 'PC_042YRE', 'PC_017F5K', 'PC_04V0PU', 'PC_05QB64', 'PC_01296N', 'PC_04DUFN', 'PC_00ZQQF', 'PC_08I1Q6', 'PC_08H9YG', 'PC_0843Z9', 'PC_07QLAB', 'PC_039ZP9', 'PC_081PV4', 'PC_007Z8T', 'PC_032CKH', 'PC_05MNXH', 'PC_02HVML', 'PC_07VHB5', 'PC_01FQRU', 'PC_02W8LT', 'PC_05KG1N', 'PC_005N2L', 'PC_06RYNI', 'PC_02CEZD', 'PC_07WEO1', 'PC_06OK2P', 'PC_00MO21', 'PC_04DQ0W', 'PC_04U1L7', 'PC_05OR8I', 'PC_05FXKZ', 'PC_07AWUL', 'PC_088DQF', 'PC_036HDQ', 'PC_07VAB2', 'PC_04VBNR', 'PC_01CDCN', 'PC_05R7KZ', 'PC_02V4AV', 'PC_05LDC6', 'PC_072EU7', 'PC_00HTJV', 'PC_046RD9', 'PC_03E0CS', 'PC_00HGKK', 'PC_03813O', 'PC_05K9FR', 'PC_03NC9U', 'PC_0860OA', 'PC_04LTCQ', 'PC_00A54M', 'PC_0370DG', 'PC_04PSFQ', 'PC_00UWAQ', 'PC_02EMSJ', 'PC_02BG1Z', 'PC_008806', 'PC_06IRIY', 'PC_053J9J', 'PC_04N53M', 'PC_08H11O', 'PC_02UBXP', 'PC_05F9H4', 'PC_022ZLX', 'PC_021V0R', 'PC_01WTO6', 'PC_07OCLR', 'PC_05FIVY', 'PC_085L07', 'PC_001RDT', 'PC_02OSJL', 'PC_03PBKY', 'PC_073NJA', 'PC_062QHL', 'PC_0708R1', 'PC_00LSXD', 'PC_07XIBT', 'PC_06ZU26', 'PC_06FIXN', 'PC_009036', 'PC_08I3IM', 'PC_05AJOI', 'PC_0804W2', 'PC_021SYL', 'PC_011686', 'PC_02AR9H', 'PC_0837FY', 'PC_074GF9', 'PC_00UFF9', 'PC_02D1GK', 'PC_05N2GV', 'PC_0753N9', 'PC_02G1YM', 'PC_03EHDR', 'PC_05TZZ7', 'PC_023QTD', 'PC_05W661', 'PC_07D4R9', 'PC_02VB7G', 'PC_05O6MK', 'PC_03K305', 'PC_05MI4Z', 'PC_027RDL', 'PC_036385', 'PC_011LAW', 'PC_04OIFF', 'PC_07UMX8', 'PC_030ACC', 'PC_02V4LH', 'PC_02KEHU', 'PC_07AGJQ', 'PC_01W301', 'PC_02JM6C', 'PC_05ILWE', 'PC_030G1S', 'PC_07NWKC', 'PC_02WCO0', 'PC_079ZWS', 'PC_06CM2C', 'PC_03JFL5', 'PC_07VJ0Q', 'PC_03T6PW', 'PC_06ADE4', 'PC_07UIKR', 'PC_0473PG', 'PC_00NN46', 'PC_063TMD', 'PC_06J307', 'PC_087UOG', 'PC_03PEL5', 'PC_02UI5W', 'PC_0181PE', 'PC_06SL6B', 'PC_0100UP', 'PC_06AI9C', 'PC_00M7NJ', 'PC_01YHQS', 'PC_00915C', 'PC_01FJ7Z', 'PC_04ZZ0B', 'PC_07076R', 'PC_04USMD', 'PC_01IC1B', 'PC_024L0V', 'PC_05TX0J', 'PC_02EV3G'}
MSA succeeded
Edge PC_04Z3ZQ PC_05RVL4 0.13914650678634644
MSA succeeded
Edge PC_032CKH PC_06K75W 0.13733305037021637
lkey 312
lkey_clusters {'PC_06BP65', 'PC_07A9N7', 'PC_04IINX', 'PC_001GNR', 'PC_0384LZ', 'PC_00772B', 'PC_060QAC', 'PC_01GNRM', 'PC_01K875', 'PC_042YRE', 'PC_017F5K', 'PC_04V0PU', 'PC_05QB64', 'PC_01296N', 'PC_04DUFN', 'PC_00ZQQF', 'PC_08I1Q6', 'PC_08H9YG', 'PC_0843Z9', 'PC_07QLAB', 'PC_039ZP9', 'PC_081PV4', 'PC_007Z8T', 'PC_032CKH', 'PC_05MNXH', 'PC_02HVML', 'PC_07VHB5', 'PC_01FQRU', 'PC_02W8LT', 'PC_05KG1N', 'PC_005N2L', 'PC_06RYNI', 'PC_02CEZD', 'PC_07WEO1', 'PC_06OK2P', 'PC_00MO21', 'PC_04DQ0W', 'PC_04U1L7', 'PC_05OR8I', 'PC_05FXKZ', 'PC_07AWUL', 'PC_088DQF', 'PC_036HDQ', 'PC_07VAB2', 'PC_04VBNR', 'PC_01CDCN', 'PC_05R7KZ', 'PC_02V4AV', 'PC_05LDC6', 'PC_072EU7', 'PC_00HTJV', 'PC_046RD9', 'PC_03E0CS', 'PC_00HGKK', 'PC_03813O', 'PC_05K9FR', 'PC_03NC9U', 'PC_0860OA', 'PC_04LTCQ', 'PC_00A54M', 'PC_0370DG', 'PC_080PLJ', 'PC_04PSFQ', 'PC_00UWAQ', 'PC_02EMSJ', 'PC_02BG1Z', 'PC_008806', 'PC_06IRIY', 'PC_053J9J', 'PC_04N53M', 'PC_08H11O', 'PC_02UBXP', 'PC_05F9H4', 'PC_022ZLX', 'PC_021V0R', 'PC_01WTO6', 'PC_07OCLR', 'PC_05FIVY', 'PC_085L07', 'PC_001RDT', 'PC_02OSJL', 'PC_03PBKY', 'PC_073NJA', 'PC_062QHL', 'PC_0708R1', 'PC_00LSXD', 'PC_07XIBT', 'PC_06ZU26', 'PC_06FIXN', 'PC_009036', 'PC_08I3IM', 'PC_05AJOI', 'PC_0804W2', 'PC_021SYL', 'PC_011686', 'PC_02AR9H', 'PC_0837FY', 'PC_074GF9', 'PC_00UFF9', 'PC_02D1GK', 'PC_05N2GV', 'PC_0753N9', 'PC_02G1YM', 'PC_03EHDR', 'PC_05TZZ7', 'PC_023QTD', 'PC_05W661', 'PC_07D4R9', 'PC_02VB7G', 'PC_05O6MK', 'PC_03K305', 'PC_05MI4Z', 'PC_027RDL', 'PC_036385', 'PC_011LAW', 'PC_04OIFF', 'PC_07UMX8', 'PC_030ACC', 'PC_02V4LH', 'PC_02KEHU', 'PC_07AGJQ', 'PC_01W301', 'PC_02JM6C', 'PC_05ILWE', 'PC_030G1S', 'PC_07NWKC', 'PC_02WCO0', 'PC_079ZWS', 'PC_06CM2C', 'PC_03JFL5', 'PC_07VJ0Q', 'PC_03T6PW', 'PC_06ADE4', 'PC_07UIKR', 'PC_0473PG', 'PC_00NN46', 'PC_063TMD', 'PC_06J307', 'PC_087UOG', 'PC_03PEL5', 'PC_02UI5W', 'PC_0181PE', 'PC_06SL6B', 'PC_0100UP', 'PC_06AI9C', 'PC_00M7NJ', 'PC_01YHQS', 'PC_00915C', 'PC_01FJ7Z', 'PC_04ZZ0B', 'PC_07076R', 'PC_04USMD', 'PC_01IC1B', 'PC_024L0V', 'PC_05TX0J', 'PC_02EV3G'}
MSA succeeded
Edge PC_031XPV PC_05RVL4 0.13721001148223877
MSA succeeded
Edge PC_05K9FR PC_06LOIO 0.13677358627319336
lkey 314
lkey_clusters {'PC_06BP65', 'PC_07A9N7', 'PC_04IINX', 'PC_001GNR', 'PC_0384LZ', 'PC_00772B', 'PC_060QAC', 'PC_01GNRM', 'PC_01K875', 'PC_042YRE', 'PC_017F5K', 'PC_04V0PU', 'PC_05QB64', 'PC_01296N', 'PC_04DUFN', 'PC_00ZQQF', 'PC_08I1Q6', 'PC_08H9YG', 'PC_0843Z9', 'PC_07QLAB', 'PC_039ZP9', 'PC_081PV4', 'PC_007Z8T', 'PC_032CKH', 'PC_05MNXH', 'PC_02HVML', 'PC_07VHB5', 'PC_01FQRU', 'PC_02W8LT', 'PC_05KG1N', 'PC_005N2L', 'PC_06RYNI', 'PC_02CEZD', 'PC_07WEO1', 'PC_06OK2P', 'PC_00MO21', 'PC_04DQ0W', 'PC_04U1L7', 'PC_05OR8I', 'PC_05FXKZ', 'PC_07AWUL', 'PC_088DQF', 'PC_036HDQ', 'PC_07VAB2', 'PC_04VBNR', 'PC_01CDCN', 'PC_05R7KZ', 'PC_02V4AV', 'PC_05LDC6', 'PC_072EU7', 'PC_00HTJV', 'PC_046RD9', 'PC_03E0CS', 'PC_00HGKK', 'PC_03813O', 'PC_05K9FR', 'PC_03NC9U', 'PC_0860OA', 'PC_04LTCQ', 'PC_00A54M', 'PC_0370DG', 'PC_080PLJ', 'PC_04PSFQ', 'PC_00UWAQ', 'PC_02EMSJ', 'PC_02BG1Z', 'PC_008806', 'PC_06IRIY', 'PC_053J9J', 'PC_04N53M', 'PC_08H11O', 'PC_02UBXP', 'PC_05F9H4', 'PC_022ZLX', 'PC_021V0R', 'PC_01WTO6', 'PC_07OCLR', 'PC_05FIVY', 'PC_085L07', 'PC_001RDT', 'PC_02OSJL', 'PC_03PBKY', 'PC_073NJA', 'PC_062QHL', 'PC_0708R1', 'PC_00LSXD', 'PC_07XIBT', 'PC_06ZU26', 'PC_06FIXN', 'PC_009036', 'PC_08I3IM', 'PC_05AJOI', 'PC_0804W2', 'PC_021SYL', 'PC_011686', 'PC_02AR9H', 'PC_0837FY', 'PC_074GF9', 'PC_00UFF9', 'PC_06K75W', 'PC_02D1GK', 'PC_05N2GV', 'PC_0753N9', 'PC_02G1YM', 'PC_03EHDR', 'PC_05TZZ7', 'PC_023QTD', 'PC_05W661', 'PC_07D4R9', 'PC_02VB7G', 'PC_05O6MK', 'PC_03K305', 'PC_05MI4Z', 'PC_027RDL', 'PC_036385', 'PC_011LAW', 'PC_04OIFF', 'PC_07UMX8', 'PC_030ACC', 'PC_02V4LH', 'PC_02KEHU', 'PC_07AGJQ', 'PC_01W301', 'PC_02JM6C', 'PC_05ILWE', 'PC_030G1S', 'PC_07NWKC', 'PC_02WCO0', 'PC_079ZWS', 'PC_06CM2C', 'PC_03JFL5', 'PC_07VJ0Q', 'PC_03T6PW', 'PC_06ADE4', 'PC_07UIKR', 'PC_0473PG', 'PC_00NN46', 'PC_063TMD', 'PC_06J307', 'PC_087UOG', 'PC_03PEL5', 'PC_02UI5W', 'PC_0181PE', 'PC_06SL6B', 'PC_0100UP', 'PC_06AI9C', 'PC_00M7NJ', 'PC_01YHQS', 'PC_00915C', 'PC_01FJ7Z', 'PC_04ZZ0B', 'PC_07076R', 'PC_04USMD', 'PC_01IC1B', 'PC_024L0V', 'PC_05TX0J', 'PC_02EV3G'}
MSA succeeded
Edge PC_03G9RN PC_07088H 0.13483431935310364
lkey 266
lkey_clusters {'PC_03G9RN', 'PC_02NISQ', 'PC_03GU7O', 'PC_05PFLL'}
MSA succeeded
Edge PC_00SR1W PC_06J307 0.1345154196023941
MSA succeeded
Edge PC_0164QS PC_01BZCN 0.13437025249004364
MSA succeeded
Edge PC_018DM6 PC_06WRVL 0.13435550034046173
lkey 272
lkey_clusters {'PC_018DM6', 'PC_07TV5U', 'PC_07TV5W', 'PC_06UZ3U', 'PC_04MR4A', 'PC_0278ZT'}
MSA succeeded
Edge PC_01D3OM PC_047570 0.13255633413791656
lkey 286
lkey_clusters {'PC_03870X', 'PC_07TUPN', 'PC_02CC4R', 'PC_009LO1', 'PC_0085EC', 'PC_063IUN', 'PC_02BNS6', 'PC_022NID', 'PC_02QW19', 'PC_01EAHK', 'PC_0393P5', 'PC_060UD5', 'PC_02R0M8', 'PC_07TV9U', 'PC_04SFDZ', 'PC_05KEYA', 'PC_04PJ1H', 'PC_01D3OM', 'PC_02QMV0'}
MSA succeeded
Edge PC_013NPU PC_05RVL4 0.13192221522331238
lkey 184
lkey_clusters {'PC_0294CS', 'PC_013NPU', 'PC_03792B'}
MSA succeeded
Edge PC_04V0PU PC_07A5YP 0.131278395652771
lkey 318
lkey_clusters {'PC_06BP65', 'PC_07A9N7', 'PC_04IINX', 'PC_001GNR', 'PC_0384LZ', 'PC_00772B', 'PC_060QAC', 'PC_01GNRM', 'PC_01K875', 'PC_042YRE', 'PC_04V0PU', 'PC_05QB64', 'PC_01296N', 'PC_04DUFN', 'PC_00ZQQF', 'PC_08I1Q6', 'PC_08H9YG', 'PC_0843Z9', 'PC_07QLAB', 'PC_039ZP9', 'PC_081PV4', 'PC_007Z8T', 'PC_032CKH', 'PC_05MNXH', 'PC_02HVML', 'PC_07VHB5', 'PC_01FQRU', 'PC_02W8LT', 'PC_05KG1N', 'PC_005N2L', 'PC_06RYNI', 'PC_02CEZD', 'PC_07WEO1', 'PC_06OK2P', 'PC_00MO21', 'PC_04DQ0W', 'PC_04U1L7', 'PC_05OR8I', 'PC_05FXKZ', 'PC_07AWUL', 'PC_088DQF', 'PC_036HDQ', 'PC_07VAB2', 'PC_04VBNR', 'PC_01CDCN', 'PC_05R7KZ', 'PC_02V4AV', 'PC_05LDC6', 'PC_072EU7', 'PC_00HTJV', 'PC_046RD9', 'PC_03E0CS', 'PC_00HGKK', 'PC_03813O', 'PC_05K9FR', 'PC_03NC9U', 'PC_0860OA', 'PC_04LTCQ', 'PC_00A54M', 'PC_0370DG', 'PC_080PLJ', 'PC_04PSFQ', 'PC_00UWAQ', 'PC_02EMSJ', 'PC_02BG1Z', 'PC_008806', 'PC_06IRIY', 'PC_053J9J', 'PC_02EV3G', 'PC_04N53M', 'PC_08H11O', 'PC_02UBXP', 'PC_05F9H4', 'PC_022ZLX', 'PC_021V0R', 'PC_01WTO6', 'PC_07OCLR', 'PC_05FIVY', 'PC_085L07', 'PC_001RDT', 'PC_02OSJL', 'PC_03PBKY', 'PC_073NJA', 'PC_062QHL', 'PC_0708R1', 'PC_00LSXD', 'PC_07XIBT', 'PC_06ZU26', 'PC_06FIXN', 'PC_009036', 'PC_08I3IM', 'PC_05AJOI', 'PC_0804W2', 'PC_021SYL', 'PC_011686', 'PC_02AR9H', 'PC_0837FY', 'PC_074GF9', 'PC_00SR1W', 'PC_00UFF9', 'PC_06K75W', 'PC_02D1GK', 'PC_05N2GV', 'PC_0753N9', 'PC_02G1YM', 'PC_03EHDR', 'PC_05TZZ7', 'PC_023QTD', 'PC_05W661', 'PC_07D4R9', 'PC_02VB7G', 'PC_05O6MK', 'PC_03K305', 'PC_05MI4Z', 'PC_027RDL', 'PC_036385', 'PC_011LAW', 'PC_04OIFF', 'PC_07UMX8', 'PC_030ACC', 'PC_02V4LH', 'PC_02KEHU', 'PC_07AGJQ', 'PC_01W301', 'PC_02JM6C', 'PC_05ILWE', 'PC_030G1S', 'PC_07NWKC', 'PC_02WCO0', 'PC_079ZWS', 'PC_06CM2C', 'PC_03JFL5', 'PC_07VJ0Q', 'PC_03T6PW', 'PC_06ADE4', 'PC_07UIKR', 'PC_0473PG', 'PC_00NN46', 'PC_063TMD', 'PC_06J307', 'PC_087UOG', 'PC_03PEL5', 'PC_02UI5W', 'PC_0181PE', 'PC_06SL6B', 'PC_0100UP', 'PC_06AI9C', 'PC_00M7NJ', 'PC_01YHQS', 'PC_00915C', 'PC_01FJ7Z', 'PC_04ZZ0B', 'PC_07076R', 'PC_04USMD', 'PC_01IC1B', 'PC_06LOIO', 'PC_024L0V', 'PC_05TX0J', 'PC_017F5K'}
MSA failed
Edge PC_02OSJL PC_03AMPS 0.1304636150598526
lkey 318
lkey_clusters {'PC_06BP65', 'PC_07A9N7', 'PC_04IINX', 'PC_001GNR', 'PC_0384LZ', 'PC_00772B', 'PC_060QAC', 'PC_01GNRM', 'PC_01K875', 'PC_042YRE', 'PC_04V0PU', 'PC_05QB64', 'PC_01296N', 'PC_04DUFN', 'PC_00ZQQF', 'PC_08I1Q6', 'PC_08H9YG', 'PC_0843Z9', 'PC_07QLAB', 'PC_039ZP9', 'PC_081PV4', 'PC_007Z8T', 'PC_032CKH', 'PC_05MNXH', 'PC_02HVML', 'PC_07VHB5', 'PC_01FQRU', 'PC_02W8LT', 'PC_05KG1N', 'PC_005N2L', 'PC_06RYNI', 'PC_02CEZD', 'PC_07WEO1', 'PC_06OK2P', 'PC_00MO21', 'PC_04DQ0W', 'PC_04U1L7', 'PC_05OR8I', 'PC_05FXKZ', 'PC_07AWUL', 'PC_088DQF', 'PC_036HDQ', 'PC_07VAB2', 'PC_04VBNR', 'PC_01CDCN', 'PC_05R7KZ', 'PC_02V4AV', 'PC_05LDC6', 'PC_072EU7', 'PC_00HTJV', 'PC_046RD9', 'PC_03E0CS', 'PC_00HGKK', 'PC_03813O', 'PC_05K9FR', 'PC_03NC9U', 'PC_0860OA', 'PC_04LTCQ', 'PC_00A54M', 'PC_0370DG', 'PC_080PLJ', 'PC_04PSFQ', 'PC_00UWAQ', 'PC_02EMSJ', 'PC_02BG1Z', 'PC_008806', 'PC_06IRIY', 'PC_053J9J', 'PC_02EV3G', 'PC_04N53M', 'PC_08H11O', 'PC_02UBXP', 'PC_05F9H4', 'PC_022ZLX', 'PC_021V0R', 'PC_01WTO6', 'PC_07OCLR', 'PC_05FIVY', 'PC_085L07', 'PC_001RDT', 'PC_02OSJL', 'PC_03PBKY', 'PC_073NJA', 'PC_062QHL', 'PC_0708R1', 'PC_00LSXD', 'PC_07XIBT', 'PC_06ZU26', 'PC_06FIXN', 'PC_009036', 'PC_08I3IM', 'PC_05AJOI', 'PC_0804W2', 'PC_021SYL', 'PC_011686', 'PC_02AR9H', 'PC_0837FY', 'PC_074GF9', 'PC_00SR1W', 'PC_00UFF9', 'PC_06K75W', 'PC_02D1GK', 'PC_05N2GV', 'PC_0753N9', 'PC_02G1YM', 'PC_03EHDR', 'PC_05TZZ7', 'PC_023QTD', 'PC_05W661', 'PC_07D4R9', 'PC_02VB7G', 'PC_05O6MK', 'PC_03K305', 'PC_05MI4Z', 'PC_027RDL', 'PC_036385', 'PC_011LAW', 'PC_04OIFF', 'PC_07UMX8', 'PC_030ACC', 'PC_02V4LH', 'PC_02KEHU', 'PC_07AGJQ', 'PC_01W301', 'PC_02JM6C', 'PC_05ILWE', 'PC_030G1S', 'PC_07NWKC', 'PC_02WCO0', 'PC_079ZWS', 'PC_06CM2C', 'PC_03JFL5', 'PC_07VJ0Q', 'PC_03T6PW', 'PC_06ADE4', 'PC_07UIKR', 'PC_0473PG', 'PC_00NN46', 'PC_063TMD', 'PC_06J307', 'PC_087UOG', 'PC_03PEL5', 'PC_02UI5W', 'PC_0181PE', 'PC_06SL6B', 'PC_0100UP', 'PC_06AI9C', 'PC_00M7NJ', 'PC_01YHQS', 'PC_00915C', 'PC_01FJ7Z', 'PC_04ZZ0B', 'PC_07076R', 'PC_04USMD', 'PC_01IC1B', 'PC_06LOIO', 'PC_024L0V', 'PC_05TX0J', 'PC_017F5K'}
MSA succeeded
Edge PC_01J10M PC_060QAC 0.1295534372329712
MSA succeeded
Edge PC_04SSH3 PC_06RYIX 0.12714411318302155
MSA succeeded
Edge PC_01KP4T PC_0843Z9 0.12539885938167572
MSA failed
Edge PC_04OJOB PC_05TX0J 0.1240568682551384
MSA succeeded
Edge PC_0654DH PC_0804W2 0.1235881820321083
lkey 311
lkey_clusters {'PC_045HU3', 'PC_07TVA5', 'PC_0654DH', 'PC_00A3UY', 'PC_02LGPA', 'PC_02GRBP', 'PC_06SE34', 'PC_05D714', 'PC_057FE7', 'PC_02K5A5', 'PC_02P3RH', 'PC_05RS5N', 'PC_04FB23', 'PC_04SK9M', 'PC_063JSC', 'PC_06EWH0', 'PC_07TV8B', 'PC_07TVM3', 'PC_048XFW', 'PC_057CGE'}
MSA succeeded
Edge PC_01KBB8 PC_05VOPA 0.12352873384952545
MSA succeeded
Edge PC_00P0Q4 PC_03XWEE 0.12260472774505615
MSA succeeded
Edge PC_01BZCN PC_02EMSJ 0.12115166336297989
lkey 319
lkey_clusters {'PC_01BZCN', 'PC_0164QS'}
MSA succeeded
Edge PC_06DDSH PC_07TUPN 0.11897322535514832
MSA succeeded
Edge PC_02T3ZI PC_05JFQY 0.11883174628019333
lkey 330
lkey_clusters {'PC_06BP65', 'PC_07A9N7', 'PC_04IINX', 'PC_001GNR', 'PC_0384LZ', 'PC_00772B', 'PC_060QAC', 'PC_01GNRM', 'PC_01K875', 'PC_042YRE', 'PC_04V0PU', 'PC_048XFW', 'PC_05QB64', 'PC_01296N', 'PC_04DUFN', 'PC_00ZQQF', 'PC_08I1Q6', 'PC_08H9YG', 'PC_0843Z9', 'PC_07QLAB', 'PC_039ZP9', 'PC_081PV4', 'PC_007Z8T', 'PC_032CKH', 'PC_05MNXH', 'PC_02HVML', 'PC_07VHB5', 'PC_01FQRU', 'PC_02W8LT', 'PC_05KG1N', 'PC_005N2L', 'PC_06RYNI', 'PC_02LGPA', 'PC_02CEZD', 'PC_07WEO1', 'PC_06OK2P', 'PC_00MO21', 'PC_063JSC', 'PC_04DQ0W', 'PC_0490I5', 'PC_06EWH0', 'PC_04U1L7', 'PC_05OR8I', 'PC_05FXKZ', 'PC_04SK9M', 'PC_07AWUL', 'PC_088DQF', 'PC_036HDQ', 'PC_07VAB2', 'PC_04VBNR', 'PC_01CDCN', 'PC_05R7KZ', 'PC_02V4AV', 'PC_05LDC6', 'PC_057FE7', 'PC_072EU7', 'PC_00HTJV', 'PC_046RD9', 'PC_03E0CS', 'PC_01J10M', 'PC_02T3ZI', 'PC_00HGKK', 'PC_03813O', 'PC_05K9FR', 'PC_03NC9U', 'PC_0860OA', 'PC_04LTCQ', 'PC_00A54M', 'PC_0370DG', 'PC_080PLJ', 'PC_057CGE', 'PC_04PSFQ', 'PC_045HU3', 'PC_00UWAQ', 'PC_02EMSJ', 'PC_02BG1Z', 'PC_008806', 'PC_06IRIY', 'PC_03AMPS', 'PC_01BZCN', 'PC_053J9J', 'PC_02EV3G', 'PC_04N53M', 'PC_08H11O', 'PC_02UBXP', 'PC_05F9H4', 'PC_022ZLX', 'PC_021V0R', 'PC_01WTO6', 'PC_07OCLR', 'PC_05FIVY', 'PC_00S0UG', 'PC_016TKH', 'PC_085L07', 'PC_07TVA5', 'PC_01GLB1', 'PC_001RDT', 'PC_02OSJL', 'PC_03PBKY', 'PC_04OJOB', 'PC_073NJA', 'PC_062QHL', 'PC_06SE34', 'PC_0708R1', 'PC_02K5A5', 'PC_00LSXD', 'PC_05RS5N', 'PC_07XIBT', 'PC_06ZU26', 'PC_06FIXN', 'PC_009036', 'PC_08I3IM', 'PC_05AJOI', 'PC_0804W2', 'PC_021SYL', 'PC_011686', 'PC_07TVM3', 'PC_02AR9H', 'PC_0837FY', 'PC_074GF9', 'PC_00SR1W', 'PC_00UFF9', 'PC_06K75W', 'PC_02D1GK', 'PC_05N2GV', 'PC_0753N9', 'PC_02G1YM', 'PC_03EHDR', 'PC_05TZZ7', 'PC_023QTD', 'PC_05W661', 'PC_07D4R9', 'PC_02VB7G', 'PC_02GRBP', 'PC_05O6MK', 'PC_03K305', 'PC_05MI4Z', 'PC_027RDL', 'PC_036385', 'PC_011LAW', 'PC_04OIFF', 'PC_07UMX8', 'PC_030ACC', 'PC_02V4LH', 'PC_02KEHU', 'PC_07AGJQ', 'PC_01W301', 'PC_02JM6C', 'PC_045E9O', 'PC_05ILWE', 'PC_030G1S', 'PC_07NWKC', 'PC_02WCO0', 'PC_079ZWS', 'PC_06CM2C', 'PC_03JFL5', 'PC_07VJ0Q', 'PC_0654DH', 'PC_00A3UY', 'PC_03T6PW', 'PC_06ADE4', 'PC_02P3RH', 'PC_07UIKR', 'PC_04FB23', 'PC_0473PG', 'PC_00NN46', 'PC_063TMD', 'PC_07TV8B', 'PC_06J307', 'PC_087UOG', 'PC_03PEL5', 'PC_04R5US', 'PC_02UI5W', 'PC_0181PE', 'PC_06SL6B', 'PC_05AVFK', 'PC_0164QS', 'PC_0100UP', 'PC_06AI9C', 'PC_00M7NJ', 'PC_01YHQS', 'PC_00915C', 'PC_01J1VJ', 'PC_01FJ7Z', 'PC_04ZZ0B', 'PC_07076R', 'PC_04USMD', 'PC_01IC1B', 'PC_06LOIO', 'PC_024L0V', 'PC_05D714', 'PC_05TX0J', 'PC_017F5K'}
MSA succeeded
Edge PC_007Z8T PC_03ALOO 0.11853956431150436
lkey 332
lkey_clusters {'PC_06BP65', 'PC_07A9N7', 'PC_04IINX', 'PC_001GNR', 'PC_0384LZ', 'PC_00772B', 'PC_060QAC', 'PC_01GNRM', 'PC_01K875', 'PC_042YRE', 'PC_04V0PU', 'PC_048XFW', 'PC_05QB64', 'PC_01296N', 'PC_04DUFN', 'PC_00ZQQF', 'PC_08I1Q6', 'PC_08H9YG', 'PC_0843Z9', 'PC_07QLAB', 'PC_039ZP9', 'PC_081PV4', 'PC_007Z8T', 'PC_032CKH', 'PC_05MNXH', 'PC_02HVML', 'PC_07VHB5', 'PC_01FQRU', 'PC_02W8LT', 'PC_05KG1N', 'PC_005N2L', 'PC_06RYNI', 'PC_02LGPA', 'PC_02CEZD', 'PC_07WEO1', 'PC_06OK2P', 'PC_00MO21', 'PC_063JSC', 'PC_04DQ0W', 'PC_0490I5', 'PC_06EWH0', 'PC_04U1L7', 'PC_05OR8I', 'PC_05FXKZ', 'PC_04SK9M', 'PC_07AWUL', 'PC_088DQF', 'PC_036HDQ', 'PC_07VAB2', 'PC_04VBNR', 'PC_01CDCN', 'PC_05R7KZ', 'PC_02V4AV', 'PC_05LDC6', 'PC_057FE7', 'PC_072EU7', 'PC_05JFQY', 'PC_00HTJV', 'PC_046RD9', 'PC_03E0CS', 'PC_01J10M', 'PC_02T3ZI', 'PC_00HGKK', 'PC_03813O', 'PC_05K9FR', 'PC_03NC9U', 'PC_0860OA', 'PC_04LTCQ', 'PC_00A54M', 'PC_0370DG', 'PC_080PLJ', 'PC_057CGE', 'PC_04PSFQ', 'PC_045HU3', 'PC_00UWAQ', 'PC_02EMSJ', 'PC_02BG1Z', 'PC_008806', 'PC_06IRIY', 'PC_03AMPS', 'PC_01BZCN', 'PC_053J9J', 'PC_02EV3G', 'PC_04N53M', 'PC_08H11O', 'PC_02UBXP', 'PC_05F9H4', 'PC_022ZLX', 'PC_021V0R', 'PC_01WTO6', 'PC_07OCLR', 'PC_05FIVY', 'PC_00S0UG', 'PC_016TKH', 'PC_085L07', 'PC_07TVA5', 'PC_01GLB1', 'PC_001RDT', 'PC_02OSJL', 'PC_03PBKY', 'PC_04OJOB', 'PC_073NJA', 'PC_062QHL', 'PC_06SE34', 'PC_0708R1', 'PC_02K5A5', 'PC_00LSXD', 'PC_05RS5N', 'PC_07XIBT', 'PC_06ZU26', 'PC_06FIXN', 'PC_009036', 'PC_08I3IM', 'PC_05AJOI', 'PC_0804W2', 'PC_021SYL', 'PC_011686', 'PC_07TVM3', 'PC_02AR9H', 'PC_0837FY', 'PC_074GF9', 'PC_00SR1W', 'PC_00UFF9', 'PC_06K75W', 'PC_02D1GK', 'PC_05N2GV', 'PC_0753N9', 'PC_02G1YM', 'PC_03EHDR', 'PC_05TZZ7', 'PC_023QTD', 'PC_05W661', 'PC_07D4R9', 'PC_02VB7G', 'PC_02GRBP', 'PC_05O6MK', 'PC_03K305', 'PC_05MI4Z', 'PC_027RDL', 'PC_036385', 'PC_011LAW', 'PC_04OIFF', 'PC_07UMX8', 'PC_030ACC', 'PC_02V4LH', 'PC_02KEHU', 'PC_07AGJQ', 'PC_01W301', 'PC_02JM6C', 'PC_045E9O', 'PC_05ILWE', 'PC_030G1S', 'PC_07NWKC', 'PC_02WCO0', 'PC_079ZWS', 'PC_06CM2C', 'PC_03JFL5', 'PC_07VJ0Q', 'PC_0654DH', 'PC_00A3UY', 'PC_03T6PW', 'PC_06ADE4', 'PC_02P3RH', 'PC_07UIKR', 'PC_04FB23', 'PC_0473PG', 'PC_00NN46', 'PC_063TMD', 'PC_07TV8B', 'PC_06J307', 'PC_087UOG', 'PC_03PEL5', 'PC_04R5US', 'PC_02UI5W', 'PC_0181PE', 'PC_06SL6B', 'PC_05AVFK', 'PC_0164QS', 'PC_0100UP', 'PC_06AI9C', 'PC_00M7NJ', 'PC_01YHQS', 'PC_00915C', 'PC_01J1VJ', 'PC_01FJ7Z', 'PC_04ZZ0B', 'PC_07076R', 'PC_04USMD', 'PC_01IC1B', 'PC_06LOIO', 'PC_024L0V', 'PC_05D714', 'PC_05TX0J', 'PC_017F5K'}
MSA failed
Edge PC_05PJ7O PC_05TX0J 0.1185031533241272
MSA succeeded
Edge PC_03GTZD PC_04SK9M 0.11814968287944794
lkey 306
lkey_clusters {'PC_03GTZD', 'PC_04L2GD', 'PC_0519TF', 'PC_024FEH', 'PC_02RGUO', 'PC_02NATZ', 'PC_05X98I', 'PC_07TUWD', 'PC_02YR8I', 'PC_041GAL', 'PC_02Q6QH', 'PC_012M5T', 'PC_029YIZ', 'PC_00QDYY', 'PC_01FFWH', 'PC_03FKVQ', 'PC_00D5EQ', 'PC_06PIV6', 'PC_003XGF', 'PC_00QNZJ', 'PC_0287UU', 'PC_01OCWE', 'PC_064DUW'}
MSA failed
Edge PC_00641O PC_04HZ79 0.1163896918296814
lkey 279
lkey_clusters {'PC_05AOUJ', 'PC_021EBE', 'PC_06UZ06', 'PC_05L7US', 'PC_04KM1I', 'PC_05K4L1', 'PC_06WVB2', 'PC_02GYSI', 'PC_00641O', 'PC_01OH6B', 'PC_016FRE', 'PC_02QUA0', 'PC_04THYL', 'PC_06HSND', 'PC_04PM5Y', 'PC_0306GU', 'PC_01CVKZ', 'PC_002I3M', 'PC_04D7VO', 'PC_004U8P', 'PC_01PMZK', 'PC_002I4B', 'PC_029RUP', 'PC_00NE3P', 'PC_02C8S3', 'PC_03PSAN', 'PC_05MHF3', 'PC_05MVJQ', 'PC_009BF9', 'PC_05XT7X'}
MSA succeeded
Edge PC_02D1GK PC_06MGDC 0.115432508289814
lkey 333
lkey_clusters {'PC_06BP65', 'PC_07A9N7', 'PC_04IINX', 'PC_001GNR', 'PC_0384LZ', 'PC_00772B', 'PC_060QAC', 'PC_01GNRM', 'PC_01K875', 'PC_042YRE', 'PC_017F5K', 'PC_04V0PU', 'PC_05QB64', 'PC_01296N', 'PC_04DUFN', 'PC_00ZQQF', 'PC_08I1Q6', 'PC_08H9YG', 'PC_0843Z9', 'PC_07QLAB', 'PC_039ZP9', 'PC_081PV4', 'PC_007Z8T', 'PC_032CKH', 'PC_05MNXH', 'PC_02HVML', 'PC_07VHB5', 'PC_01FQRU', 'PC_02W8LT', 'PC_05KG1N', 'PC_005N2L', 'PC_06RYNI', 'PC_02LGPA', 'PC_02CEZD', 'PC_07WEO1', 'PC_06OK2P', 'PC_00MO21', 'PC_063JSC', 'PC_04DQ0W', 'PC_0490I5', 'PC_06EWH0', 'PC_04U1L7', 'PC_05OR8I', 'PC_05FXKZ', 'PC_04SK9M', 'PC_07AWUL', 'PC_088DQF', 'PC_036HDQ', 'PC_07VAB2', 'PC_04VBNR', 'PC_01CDCN', 'PC_05R7KZ', 'PC_02V4AV', 'PC_05LDC6', 'PC_057FE7', 'PC_072EU7', 'PC_05JFQY', 'PC_00HTJV', 'PC_046RD9', 'PC_03E0CS', 'PC_01J10M', 'PC_02T3ZI', 'PC_00HGKK', 'PC_03813O', 'PC_05K9FR', 'PC_03NC9U', 'PC_0860OA', 'PC_04LTCQ', 'PC_00A54M', 'PC_0370DG', 'PC_080PLJ', 'PC_057CGE', 'PC_04PSFQ', 'PC_045HU3', 'PC_00UWAQ', 'PC_02EMSJ', 'PC_02BG1Z', 'PC_008806', 'PC_06IRIY', 'PC_03AMPS', 'PC_01BZCN', 'PC_053J9J', 'PC_02EV3G', 'PC_04N53M', 'PC_08H11O', 'PC_02UBXP', 'PC_05F9H4', 'PC_022ZLX', 'PC_021V0R', 'PC_01WTO6', 'PC_07OCLR', 'PC_05FIVY', 'PC_00S0UG', 'PC_016TKH', 'PC_085L07', 'PC_07TVA5', 'PC_01GLB1', 'PC_001RDT', 'PC_02OSJL', 'PC_03PBKY', 'PC_04OJOB', 'PC_073NJA', 'PC_062QHL', 'PC_06SE34', 'PC_0708R1', 'PC_02K5A5', 'PC_00LSXD', 'PC_05RS5N', 'PC_07XIBT', 'PC_06ZU26', 'PC_06FIXN', 'PC_009036', 'PC_08I3IM', 'PC_05AJOI', 'PC_0804W2', 'PC_021SYL', 'PC_011686', 'PC_07TVM3', 'PC_02AR9H', 'PC_0837FY', 'PC_074GF9', 'PC_00SR1W', 'PC_00UFF9', 'PC_06K75W', 'PC_02D1GK', 'PC_05N2GV', 'PC_0753N9', 'PC_02G1YM', 'PC_03EHDR', 'PC_05TZZ7', 'PC_023QTD', 'PC_05W661', 'PC_07D4R9', 'PC_02VB7G', 'PC_02GRBP', 'PC_05O6MK', 'PC_03K305', 'PC_05MI4Z', 'PC_027RDL', 'PC_036385', 'PC_011LAW', 'PC_04OIFF', 'PC_07UMX8', 'PC_030ACC', 'PC_02V4LH', 'PC_02KEHU', 'PC_07AGJQ', 'PC_01W301', 'PC_02JM6C', 'PC_045E9O', 'PC_05ILWE', 'PC_030G1S', 'PC_07NWKC', 'PC_02WCO0', 'PC_079ZWS', 'PC_06CM2C', 'PC_03JFL5', 'PC_07VJ0Q', 'PC_0654DH', 'PC_00A3UY', 'PC_03T6PW', 'PC_06ADE4', 'PC_02P3RH', 'PC_07UIKR', 'PC_04FB23', 'PC_0473PG', 'PC_00NN46', 'PC_063TMD', 'PC_07TV8B', 'PC_06J307', 'PC_087UOG', 'PC_03PEL5', 'PC_04R5US', 'PC_02UI5W', 'PC_0181PE', 'PC_06SL6B', 'PC_05PJ7O', 'PC_05AVFK', 'PC_0164QS', 'PC_0100UP', 'PC_06AI9C', 'PC_00M7NJ', 'PC_01YHQS', 'PC_00915C', 'PC_01J1VJ', 'PC_01FJ7Z', 'PC_04ZZ0B', 'PC_07076R', 'PC_04USMD', 'PC_01IC1B', 'PC_06LOIO', 'PC_024L0V', 'PC_05D714', 'PC_05TX0J', 'PC_048XFW'}
MSA succeeded
Edge PC_02R0M8 PC_07TUNO 0.1146790161728859
lkey 331
lkey_clusters {'PC_03870X', 'PC_047570', 'PC_07TUPN', 'PC_02CC4R', 'PC_009LO1', 'PC_0085EC', 'PC_063IUN', 'PC_02BNS6', 'PC_022NID', 'PC_02QW19', 'PC_01EAHK', 'PC_06DDSH', 'PC_0393P5', 'PC_060UD5', 'PC_02R0M8', 'PC_07TV9U', 'PC_04SFDZ', 'PC_05KEYA', 'PC_04PJ1H', 'PC_01D3OM', 'PC_02QMV0'}
MSA succeeded
Edge PC_03TCZP PC_05TX0J 0.11416839808225632
MSA failed
Edge PC_0060XJ PC_07TVF3 0.11401170492172241
MSA succeeded
Edge PC_031XPV PC_07OCLR 0.11221623420715332
lkey 322
lkey_clusters {'PC_031XPV', 'PC_0294CS', 'PC_06BL39', 'PC_00UAB7', 'PC_013NPU', 'PC_03792B', 'PC_04Z3ZQ', 'PC_05RVL4', 'PC_04UWXG', 'PC_04E4JE'}
MSA succeeded
Edge PC_01FGFJ PC_07TV02 0.10786948353052139
lkey 275
lkey_clusters {'PC_02KYVT', 'PC_01FGFJ'}
MSA succeeded
Edge PC_0060XJ PC_07TVM3 0.10621042549610138
lkey 337
lkey_clusters {'PC_0060XJ', 'PC_07TVF3'}
MSA succeeded
Edge PC_04HZ79 PC_06280D 0.1059034988284111
lkey 339
lkey_clusters {'PC_05AOUJ', 'PC_04KM1I', 'PC_05K4L1', 'PC_06WVB2', 'PC_00641O', 'PC_01OH6B', 'PC_016FRE', 'PC_04PM5Y', 'PC_0306GU', 'PC_01CVKZ', 'PC_002I3M', 'PC_01PMZK', 'PC_002I4B', 'PC_029RUP', 'PC_01FGFJ', 'PC_00NE3P', 'PC_02C8S3', 'PC_05XT7X', 'PC_009BF9', 'PC_05DIQW', 'PC_02KYVT', 'PC_021EBE', 'PC_06UZ06', 'PC_05L7US', 'PC_07TV02', 'PC_02GYSI', 'PC_02QUA0', 'PC_04THYL', 'PC_06HSND', 'PC_04HZ79', 'PC_04D7VO', 'PC_004U8P', 'PC_03PSAN', 'PC_05MHF3', 'PC_05MVJQ', 'PC_01WPL7'}
MSA succeeded
Edge PC_045HU3 PC_047570 0.10430342704057693
lkey 340
lkey_clusters {'PC_06BP65', 'PC_07A9N7', 'PC_04IINX', 'PC_001GNR', 'PC_0384LZ', 'PC_00772B', 'PC_060QAC', 'PC_01GNRM', 'PC_01K875', 'PC_042YRE', 'PC_017F5K', 'PC_04V0PU', 'PC_05QB64', 'PC_01296N', 'PC_013NPU', 'PC_04DUFN', 'PC_00ZQQF', 'PC_08I1Q6', 'PC_08H9YG', 'PC_0843Z9', 'PC_07QLAB', 'PC_04E4JE', 'PC_039ZP9', 'PC_081PV4', 'PC_007Z8T', 'PC_032CKH', 'PC_05MNXH', 'PC_018GH8', 'PC_02HVML', 'PC_07VHB5', 'PC_01FQRU', 'PC_02W8LT', 'PC_05KG1N', 'PC_005N2L', 'PC_06RYNI', 'PC_02LGPA', 'PC_02CEZD', 'PC_04Z3ZQ', 'PC_07WEO1', 'PC_06OK2P', 'PC_00MO21', 'PC_063JSC', 'PC_04DQ0W', 'PC_0490I5', 'PC_059TG9', 'PC_06EWH0', 'PC_04U1L7', 'PC_05OR8I', 'PC_05FXKZ', 'PC_06BL39', 'PC_04SK9M', 'PC_07AWUL', 'PC_088DQF', 'PC_036HDQ', 'PC_07VAB2', 'PC_04VBNR', 'PC_01CDCN', 'PC_05R7KZ', 'PC_01Y0R1', 'PC_02V4AV', 'PC_05LDC6', 'PC_05RVL4', 'PC_057FE7', 'PC_072EU7', 'PC_05JFQY', 'PC_00HTJV', 'PC_046RD9', 'PC_03E0CS', 'PC_01J10M', 'PC_02T3ZI', 'PC_00HGKK', 'PC_03813O', 'PC_0060XJ', 'PC_05K9FR', 'PC_03NC9U', 'PC_0860OA', 'PC_04LTCQ', 'PC_00A54M', 'PC_0370DG', 'PC_04UWXG', 'PC_080PLJ', 'PC_057CGE', 'PC_04PSFQ', 'PC_045HU3', 'PC_00UWAQ', 'PC_02EMSJ', 'PC_02BG1Z', 'PC_008806', 'PC_06IRIY', 'PC_03AMPS', 'PC_01BZCN', 'PC_053J9J', 'PC_05W6XP', 'PC_02EV3G', 'PC_04N53M', 'PC_08H11O', 'PC_02UBXP', 'PC_05F9H4', 'PC_022ZLX', 'PC_021V0R', 'PC_01WTO6', 'PC_07TVF3', 'PC_07OCLR', 'PC_05FIVY', 'PC_00S0UG', 'PC_016TKH', 'PC_085L07', 'PC_07TVA5', 'PC_01GLB1', 'PC_001RDT', 'PC_02OSJL', 'PC_03PBKY', 'PC_04OJOB', 'PC_073NJA', 'PC_062QHL', 'PC_06SE34', 'PC_0708R1', 'PC_02K5A5', 'PC_00LSXD', 'PC_05RS5N', 'PC_07XIBT', 'PC_06ZU26', 'PC_06FIXN', 'PC_009036', 'PC_08I3IM', 'PC_05AJOI', 'PC_0804W2', 'PC_021SYL', 'PC_011686', 'PC_07TVM3', 'PC_02AR9H', 'PC_0837FY', 'PC_074GF9', 'PC_00SR1W', 'PC_00UFF9', 'PC_06K75W', 'PC_02D1GK', 'PC_05N2GV', 'PC_0753N9', 'PC_02G1YM', 'PC_03EHDR', 'PC_05TZZ7', 'PC_023QTD', 'PC_05W661', 'PC_07D4R9', 'PC_02VB7G', 'PC_02GRBP', 'PC_05O6MK', 'PC_03K305', 'PC_05MI4Z', 'PC_031XPV', 'PC_027RDL', 'PC_036385', 'PC_011LAW', 'PC_04OIFF', 'PC_07UMX8', 'PC_030ACC', 'PC_02V4LH', 'PC_02KEHU', 'PC_03792B', 'PC_07AGJQ', 'PC_01W301', 'PC_02JM6C', 'PC_045E9O', 'PC_05ILWE', 'PC_030G1S', 'PC_07NWKC', 'PC_02WCO0', 'PC_079ZWS', 'PC_06CM2C', 'PC_03JFL5', 'PC_07VJ0Q', 'PC_0654DH', 'PC_00A3UY', 'PC_03T6PW', 'PC_06ADE4', 'PC_02P3RH', 'PC_07UIKR', 'PC_04FB23', 'PC_0294CS', 'PC_0473PG', 'PC_00NN46', 'PC_063TMD', 'PC_07TV8B', 'PC_06J307', 'PC_087UOG', 'PC_03PEL5', 'PC_04R5US', 'PC_06MGDC', 'PC_02UI5W', 'PC_0181PE', 'PC_06SL6B', 'PC_05PJ7O', 'PC_05AVFK', 'PC_0164QS', 'PC_0100UP', 'PC_06AI9C', 'PC_00M7NJ', 'PC_01YHQS', 'PC_00915C', 'PC_01J1VJ', 'PC_01FJ7Z', 'PC_04ZZ0B', 'PC_07076R', 'PC_04USMD', 'PC_00UAB7', 'PC_01IC1B', 'PC_06LOIO', 'PC_024L0V', 'PC_05D714', 'PC_05TX0J', 'PC_048XFW'}
MSA succeeded
Edge PC_032XC4 PC_042YRE 0.10313240438699722
MSA succeeded
Edge PC_05TFVL PC_07TV5W 0.1006479561328888
lkey 217
lkey_clusters {'PC_00VTNG', 'PC_05TFVL'}
MSA succeeded
Edge PC_02QUA0 PC_06RYIX 0.10023457556962967
lkey 341
lkey_clusters {'PC_05AOUJ', 'PC_04KM1I', 'PC_05K4L1', 'PC_06WVB2', 'PC_00641O', 'PC_01OH6B', 'PC_016FRE', 'PC_04PM5Y', 'PC_0306GU', 'PC_01CVKZ', 'PC_002I3M', 'PC_01PMZK', 'PC_002I4B', 'PC_029RUP', 'PC_01FGFJ', 'PC_00NE3P', 'PC_02C8S3', 'PC_05XT7X', 'PC_009BF9', 'PC_05DIQW', 'PC_02KYVT', 'PC_021EBE', 'PC_06UZ06', 'PC_05L7US', 'PC_07TV02', 'PC_02GYSI', 'PC_02QUA0', 'PC_04THYL', 'PC_06HSND', 'PC_04HZ79', 'PC_04D7VO', 'PC_004U8P', 'PC_03PSAN', 'PC_05MHF3', 'PC_05MVJQ', 'PC_06280D', 'PC_02XK62', 'PC_01WPL7'}
MSA succeeded
Edge PC_06280D PC_072DWQ 0.09929443895816803
lkey 345
lkey_clusters {'PC_05AOUJ', 'PC_057VS9', 'PC_04KM1I', 'PC_04M1B3', 'PC_05K4L1', 'PC_06WVB2', 'PC_00641O', 'PC_01OH6B', 'PC_016FRE', 'PC_04PM5Y', 'PC_0306GU', 'PC_01CVKZ', 'PC_0091OE', 'PC_06RYIX', 'PC_03QNGV', 'PC_002I3M', 'PC_07TV6Z', 'PC_01PMZK', 'PC_002I4B', 'PC_00LBZX', 'PC_029RUP', 'PC_01FGFJ', 'PC_00NE3P', 'PC_02C8S3', 'PC_05XT7X', 'PC_06K136', 'PC_009BF9', 'PC_05VZ2W', 'PC_05DIQW', 'PC_02KYVT', 'PC_021EBE', 'PC_06UZ06', 'PC_05L7US', 'PC_04SSH3', 'PC_07TV02', 'PC_03HGF9', 'PC_02GYSI', 'PC_01VGHN', 'PC_01R1KR', 'PC_03970R', 'PC_074DQF', 'PC_02QUA0', 'PC_04THYL', 'PC_06HSND', 'PC_04HZ79', 'PC_02JQ3K', 'PC_05W9ZE', 'PC_04D7VO', 'PC_004U8P', 'PC_07TUTP', 'PC_03PSAN', 'PC_05MHF3', 'PC_05MVJQ', 'PC_00DU1R', 'PC_0109ZP', 'PC_06280D', 'PC_02XK62', 'PC_01WPL7', 'PC_051C8B'}
MSA succeeded
Edge PC_05VOPA PC_06UZ3U 0.09888658672571182
lkey 328
lkey_clusters {'PC_02QIDH', 'PC_01KBB8', 'PC_00595C', 'PC_02DAU3', 'PC_01QQ1T', 'PC_073XSY', 'PC_01KTUJ', 'PC_00YPBK', 'PC_05VOPA', 'PC_03AR0I', 'PC_00646F', 'PC_07TV4U'}
MSA succeeded
Edge PC_01QQ1T PC_04DUFN 0.0965680480003357
lkey 347
lkey_clusters {'PC_04MR4A', 'PC_01QQ1T', 'PC_0278ZT', 'PC_07TV5U', 'PC_05VOPA', 'PC_03AR0I', 'PC_05TFVL', 'PC_073XSY', 'PC_00VTNG', 'PC_018DM6', 'PC_02QIDH', 'PC_01KBB8', 'PC_00595C', 'PC_02DAU3', 'PC_01KTUJ', 'PC_00YPBK', 'PC_07TV5W', 'PC_06WRVL', 'PC_00646F', 'PC_07TV4U', 'PC_06UZ3U'}
MSA succeeded
Edge PC_02WPLI PC_04V0PU 0.09385459870100021
MSA failed
Edge PC_018GH8 PC_0574V4 0.09232421219348907
lkey 348
lkey_clusters {'PC_060QAC', 'PC_01K875', 'PC_04V0PU', 'PC_01KBB8', 'PC_01296N', 'PC_013NPU', 'PC_00ZQQF', 'PC_08H9YG', 'PC_081PV4', 'PC_007Z8T', 'PC_032CKH', 'PC_018GH8', 'PC_02HVML', 'PC_01FQRU', 'PC_02W8LT', 'PC_005N2L', 'PC_07WEO1', 'PC_04Z3ZQ', 'PC_06OK2P', 'PC_00MO21', 'PC_04DQ0W', 'PC_059TG9', 'PC_06EWH0', 'PC_04U1L7', 'PC_07TV5W', 'PC_07AWUL', 'PC_036HDQ', 'PC_07VAB2', 'PC_01CDCN', 'PC_05R7KZ', 'PC_02V4AV', 'PC_05LDC6', 'PC_072EU7', 'PC_01J10M', 'PC_02T3ZI', 'PC_0060XJ', 'PC_05K9FR', 'PC_03NC9U', 'PC_02R0M8', 'PC_04LTCQ', 'PC_00A54M', 'PC_0370DG', 'PC_080PLJ', 'PC_057CGE', 'PC_02EMSJ', 'PC_06IRIY', 'PC_03AMPS', 'PC_07TV5U', 'PC_05VOPA', 'PC_053J9J', 'PC_04N53M', 'PC_022NID', 'PC_05F9H4', 'PC_022ZLX', 'PC_01WTO6', 'PC_07OCLR', 'PC_05FIVY', 'PC_085L07', 'PC_03870X', 'PC_01GLB1', 'PC_001RDT', 'PC_02OSJL', 'PC_04OJOB', 'PC_073NJA', 'PC_062QHL', 'PC_0708R1', 'PC_00LSXD', 'PC_063IUN', 'PC_05RS5N', 'PC_009036', 'PC_08I3IM', 'PC_05AJOI', 'PC_011686', 'PC_02AR9H', 'PC_0837FY', 'PC_074GF9', 'PC_02D1GK', 'PC_0753N9', 'PC_03EHDR', 'PC_023QTD', 'PC_02GRBP', 'PC_05O6MK', 'PC_027RDL', 'PC_02V4LH', 'PC_02KEHU', 'PC_01W301', 'PC_02JM6C', 'PC_05ILWE', 'PC_030G1S', 'PC_079ZWS', 'PC_02WCO0', 'PC_047570', 'PC_03JFL5', 'PC_07VJ0Q', 'PC_00A3UY', 'PC_03T6PW', 'PC_06ADE4', 'PC_02P3RH', 'PC_0294CS', 'PC_0473PG', 'PC_0393P5', 'PC_063TMD', 'PC_07TV9U', 'PC_07TV8B', 'PC_06J307', 'PC_087UOG', 'PC_06MGDC', 'PC_02UI5W', 'PC_0181PE', 'PC_05PJ7O', 'PC_0164QS', 'PC_0100UP', 'PC_02CC4R', 'PC_06AI9C', 'PC_00M7NJ', 'PC_03AR0I', 'PC_009LO1', 'PC_0085EC', 'PC_02BNS6', 'PC_01FJ7Z', 'PC_04ZZ0B', 'PC_07076R', 'PC_04USMD', 'PC_00UAB7', 'PC_01IC1B', 'PC_06LOIO', 'PC_05TX0J', 'PC_02EV3G', 'PC_017F5K', 'PC_02QMV0', 'PC_06BP65', 'PC_07A9N7', 'PC_04IINX', 'PC_001GNR', 'PC_0384LZ', 'PC_00772B', 'PC_05TFVL', 'PC_01GNRM', 'PC_042YRE', 'PC_07TUNO', 'PC_06DDSH', 'PC_05QB64', 'PC_04DUFN', 'PC_08I1Q6', 'PC_0843Z9', 'PC_07QLAB', 'PC_04E4JE', 'PC_039ZP9', 'PC_05MNXH', 'PC_07VHB5', 'PC_05KG1N', 'PC_06RYNI', 'PC_02LGPA', 'PC_02CEZD', 'PC_063JSC', 'PC_0490I5', 'PC_05OR8I', 'PC_05FXKZ', 'PC_07TV4U', 'PC_01D3OM', 'PC_06BL39', 'PC_04SK9M', 'PC_088DQF', 'PC_07TUPN', 'PC_04VBNR', 'PC_01Y0R1', 'PC_05RVL4', 'PC_057FE7', 'PC_05JFQY', 'PC_00HTJV', 'PC_02QIDH', 'PC_046RD9', 'PC_03E0CS', 'PC_00HGKK', 'PC_03813O', 'PC_01KTUJ', 'PC_0860OA', 'PC_04UWXG', 'PC_04PSFQ', 'PC_045HU3', 'PC_00UWAQ', 'PC_02BG1Z', 'PC_008806', 'PC_0278ZT', 'PC_01BZCN', 'PC_05W6XP', 'PC_073XSY', 'PC_08H11O', 'PC_02QW19', 'PC_01EAHK', 'PC_02UBXP', 'PC_02DAU3', 'PC_021V0R', 'PC_07TVF3', 'PC_00S0UG', 'PC_016TKH', 'PC_07TVA5', 'PC_03PBKY', 'PC_06SE34', 'PC_02K5A5', 'PC_07XIBT', 'PC_06ZU26', 'PC_06FIXN', 'PC_00595C', 'PC_05KEYA', 'PC_0804W2', 'PC_021SYL', 'PC_06WRVL', 'PC_06UZ3U', 'PC_07TVM3', 'PC_00SR1W', 'PC_00UFF9', 'PC_06K75W', 'PC_05N2GV', 'PC_04MR4A', 'PC_02G1YM', 'PC_01QQ1T', 'PC_05TZZ7', 'PC_05W661', 'PC_07D4R9', 'PC_02VB7G', 'PC_00VTNG', 'PC_03K305', 'PC_05MI4Z', 'PC_018DM6', 'PC_031XPV', 'PC_036385', 'PC_011LAW', 'PC_04OIFF', 'PC_07UMX8', 'PC_030ACC', 'PC_04SFDZ', 'PC_00YPBK', 'PC_04PJ1H', 'PC_03792B', 'PC_07AGJQ', 'PC_045E9O', 'PC_032XC4', 'PC_07NWKC', 'PC_06CM2C', 'PC_0654DH', 'PC_07UIKR', 'PC_04FB23', 'PC_00NN46', 'PC_04R5US', 'PC_03PEL5', 'PC_06SL6B', 'PC_05AVFK', 'PC_01YHQS', 'PC_00915C', 'PC_01J1VJ', 'PC_060UD5', 'PC_00646F', 'PC_024L0V', 'PC_05D714', 'PC_048XFW'}
MSA failed
Edge PC_03G9RN PC_072EU7 0.08931116759777069
lkey 317
lkey_clusters {'PC_03KYEF', 'PC_05PFLL', 'PC_08K2VM', 'PC_02NISQ', 'PC_03GU7O', 'PC_00QJR6', 'PC_07088H', 'PC_03G9RN'}
MSA failed
Edge PC_02EMSJ PC_06BZLT 0.08916065096855164
lkey 348
lkey_clusters {'PC_060QAC', 'PC_01K875', 'PC_04V0PU', 'PC_01KBB8', 'PC_01296N', 'PC_013NPU', 'PC_00ZQQF', 'PC_08H9YG', 'PC_081PV4', 'PC_007Z8T', 'PC_032CKH', 'PC_018GH8', 'PC_02HVML', 'PC_01FQRU', 'PC_02W8LT', 'PC_005N2L', 'PC_07WEO1', 'PC_04Z3ZQ', 'PC_06OK2P', 'PC_00MO21', 'PC_04DQ0W', 'PC_059TG9', 'PC_06EWH0', 'PC_04U1L7', 'PC_07TV5W', 'PC_07AWUL', 'PC_036HDQ', 'PC_07VAB2', 'PC_01CDCN', 'PC_05R7KZ', 'PC_02V4AV', 'PC_05LDC6', 'PC_072EU7', 'PC_01J10M', 'PC_02T3ZI', 'PC_0060XJ', 'PC_05K9FR', 'PC_03NC9U', 'PC_02R0M8', 'PC_04LTCQ', 'PC_00A54M', 'PC_0370DG', 'PC_080PLJ', 'PC_057CGE', 'PC_02EMSJ', 'PC_06IRIY', 'PC_03AMPS', 'PC_07TV5U', 'PC_05VOPA', 'PC_053J9J', 'PC_04N53M', 'PC_022NID', 'PC_05F9H4', 'PC_022ZLX', 'PC_01WTO6', 'PC_07OCLR', 'PC_05FIVY', 'PC_085L07', 'PC_03870X', 'PC_01GLB1', 'PC_001RDT', 'PC_02OSJL', 'PC_04OJOB', 'PC_073NJA', 'PC_062QHL', 'PC_0708R1', 'PC_00LSXD', 'PC_063IUN', 'PC_05RS5N', 'PC_009036', 'PC_08I3IM', 'PC_05AJOI', 'PC_011686', 'PC_02AR9H', 'PC_0837FY', 'PC_074GF9', 'PC_02D1GK', 'PC_0753N9', 'PC_03EHDR', 'PC_023QTD', 'PC_02GRBP', 'PC_05O6MK', 'PC_027RDL', 'PC_02V4LH', 'PC_02KEHU', 'PC_01W301', 'PC_02JM6C', 'PC_05ILWE', 'PC_030G1S', 'PC_079ZWS', 'PC_02WCO0', 'PC_047570', 'PC_03JFL5', 'PC_07VJ0Q', 'PC_00A3UY', 'PC_03T6PW', 'PC_06ADE4', 'PC_02P3RH', 'PC_0294CS', 'PC_0473PG', 'PC_0393P5', 'PC_063TMD', 'PC_07TV9U', 'PC_07TV8B', 'PC_06J307', 'PC_087UOG', 'PC_06MGDC', 'PC_02UI5W', 'PC_0181PE', 'PC_05PJ7O', 'PC_0164QS', 'PC_0100UP', 'PC_02CC4R', 'PC_06AI9C', 'PC_00M7NJ', 'PC_03AR0I', 'PC_009LO1', 'PC_0085EC', 'PC_02BNS6', 'PC_01FJ7Z', 'PC_04ZZ0B', 'PC_07076R', 'PC_04USMD', 'PC_00UAB7', 'PC_01IC1B', 'PC_06LOIO', 'PC_05TX0J', 'PC_02EV3G', 'PC_017F5K', 'PC_02QMV0', 'PC_06BP65', 'PC_07A9N7', 'PC_04IINX', 'PC_001GNR', 'PC_0384LZ', 'PC_00772B', 'PC_05TFVL', 'PC_01GNRM', 'PC_042YRE', 'PC_07TUNO', 'PC_06DDSH', 'PC_05QB64', 'PC_04DUFN', 'PC_08I1Q6', 'PC_0843Z9', 'PC_07QLAB', 'PC_04E4JE', 'PC_039ZP9', 'PC_05MNXH', 'PC_07VHB5', 'PC_05KG1N', 'PC_06RYNI', 'PC_02LGPA', 'PC_02CEZD', 'PC_063JSC', 'PC_0490I5', 'PC_05OR8I', 'PC_05FXKZ', 'PC_07TV4U', 'PC_01D3OM', 'PC_06BL39', 'PC_04SK9M', 'PC_088DQF', 'PC_07TUPN', 'PC_04VBNR', 'PC_01Y0R1', 'PC_05RVL4', 'PC_057FE7', 'PC_05JFQY', 'PC_00HTJV', 'PC_02QIDH', 'PC_046RD9', 'PC_03E0CS', 'PC_00HGKK', 'PC_03813O', 'PC_01KTUJ', 'PC_0860OA', 'PC_04UWXG', 'PC_04PSFQ', 'PC_045HU3', 'PC_00UWAQ', 'PC_02BG1Z', 'PC_008806', 'PC_0278ZT', 'PC_01BZCN', 'PC_05W6XP', 'PC_073XSY', 'PC_08H11O', 'PC_02QW19', 'PC_01EAHK', 'PC_02UBXP', 'PC_02DAU3', 'PC_021V0R', 'PC_07TVF3', 'PC_00S0UG', 'PC_016TKH', 'PC_07TVA5', 'PC_03PBKY', 'PC_06SE34', 'PC_02K5A5', 'PC_07XIBT', 'PC_06ZU26', 'PC_06FIXN', 'PC_00595C', 'PC_05KEYA', 'PC_0804W2', 'PC_021SYL', 'PC_06WRVL', 'PC_06UZ3U', 'PC_07TVM3', 'PC_00SR1W', 'PC_00UFF9', 'PC_06K75W', 'PC_05N2GV', 'PC_04MR4A', 'PC_02G1YM', 'PC_01QQ1T', 'PC_05TZZ7', 'PC_05W661', 'PC_07D4R9', 'PC_02VB7G', 'PC_00VTNG', 'PC_03K305', 'PC_05MI4Z', 'PC_018DM6', 'PC_031XPV', 'PC_036385', 'PC_011LAW', 'PC_04OIFF', 'PC_07UMX8', 'PC_030ACC', 'PC_04SFDZ', 'PC_00YPBK', 'PC_04PJ1H', 'PC_03792B', 'PC_07AGJQ', 'PC_045E9O', 'PC_032XC4', 'PC_07NWKC', 'PC_06CM2C', 'PC_0654DH', 'PC_07UIKR', 'PC_04FB23', 'PC_00NN46', 'PC_04R5US', 'PC_03PEL5', 'PC_06SL6B', 'PC_05AVFK', 'PC_01YHQS', 'PC_00915C', 'PC_01J1VJ', 'PC_060UD5', 'PC_00646F', 'PC_024L0V', 'PC_05D714', 'PC_048XFW'}
MSA failed
Edge PC_02EV3G PC_04ULOH 0.08390672504901886
lkey 348
lkey_clusters {'PC_060QAC', 'PC_01K875', 'PC_04V0PU', 'PC_01KBB8', 'PC_01296N', 'PC_013NPU', 'PC_00ZQQF', 'PC_08H9YG', 'PC_081PV4', 'PC_007Z8T', 'PC_032CKH', 'PC_018GH8', 'PC_02HVML', 'PC_01FQRU', 'PC_02W8LT', 'PC_005N2L', 'PC_07WEO1', 'PC_04Z3ZQ', 'PC_06OK2P', 'PC_00MO21', 'PC_04DQ0W', 'PC_059TG9', 'PC_06EWH0', 'PC_04U1L7', 'PC_07TV5W', 'PC_07AWUL', 'PC_036HDQ', 'PC_07VAB2', 'PC_01CDCN', 'PC_05R7KZ', 'PC_02V4AV', 'PC_05LDC6', 'PC_072EU7', 'PC_01J10M', 'PC_02T3ZI', 'PC_0060XJ', 'PC_05K9FR', 'PC_03NC9U', 'PC_02R0M8', 'PC_04LTCQ', 'PC_00A54M', 'PC_0370DG', 'PC_080PLJ', 'PC_057CGE', 'PC_02EMSJ', 'PC_06IRIY', 'PC_03AMPS', 'PC_07TV5U', 'PC_05VOPA', 'PC_053J9J', 'PC_04N53M', 'PC_022NID', 'PC_05F9H4', 'PC_022ZLX', 'PC_01WTO6', 'PC_07OCLR', 'PC_05FIVY', 'PC_085L07', 'PC_03870X', 'PC_01GLB1', 'PC_001RDT', 'PC_02OSJL', 'PC_04OJOB', 'PC_073NJA', 'PC_062QHL', 'PC_0708R1', 'PC_00LSXD', 'PC_063IUN', 'PC_05RS5N', 'PC_009036', 'PC_08I3IM', 'PC_05AJOI', 'PC_011686', 'PC_02AR9H', 'PC_0837FY', 'PC_074GF9', 'PC_02D1GK', 'PC_0753N9', 'PC_03EHDR', 'PC_023QTD', 'PC_02GRBP', 'PC_05O6MK', 'PC_027RDL', 'PC_02V4LH', 'PC_02KEHU', 'PC_01W301', 'PC_02JM6C', 'PC_05ILWE', 'PC_030G1S', 'PC_079ZWS', 'PC_02WCO0', 'PC_047570', 'PC_03JFL5', 'PC_07VJ0Q', 'PC_00A3UY', 'PC_03T6PW', 'PC_06ADE4', 'PC_02P3RH', 'PC_0294CS', 'PC_0473PG', 'PC_0393P5', 'PC_063TMD', 'PC_07TV9U', 'PC_07TV8B', 'PC_06J307', 'PC_087UOG', 'PC_06MGDC', 'PC_02UI5W', 'PC_0181PE', 'PC_05PJ7O', 'PC_0164QS', 'PC_0100UP', 'PC_02CC4R', 'PC_06AI9C', 'PC_00M7NJ', 'PC_03AR0I', 'PC_009LO1', 'PC_0085EC', 'PC_02BNS6', 'PC_01FJ7Z', 'PC_04ZZ0B', 'PC_07076R', 'PC_04USMD', 'PC_00UAB7', 'PC_01IC1B', 'PC_06LOIO', 'PC_05TX0J', 'PC_02EV3G', 'PC_017F5K', 'PC_02QMV0', 'PC_06BP65', 'PC_07A9N7', 'PC_04IINX', 'PC_001GNR', 'PC_0384LZ', 'PC_00772B', 'PC_05TFVL', 'PC_01GNRM', 'PC_042YRE', 'PC_07TUNO', 'PC_06DDSH', 'PC_05QB64', 'PC_04DUFN', 'PC_08I1Q6', 'PC_0843Z9', 'PC_07QLAB', 'PC_04E4JE', 'PC_039ZP9', 'PC_05MNXH', 'PC_07VHB5', 'PC_05KG1N', 'PC_06RYNI', 'PC_02LGPA', 'PC_02CEZD', 'PC_063JSC', 'PC_0490I5', 'PC_05OR8I', 'PC_05FXKZ', 'PC_07TV4U', 'PC_01D3OM', 'PC_06BL39', 'PC_04SK9M', 'PC_088DQF', 'PC_07TUPN', 'PC_04VBNR', 'PC_01Y0R1', 'PC_05RVL4', 'PC_057FE7', 'PC_05JFQY', 'PC_00HTJV', 'PC_02QIDH', 'PC_046RD9', 'PC_03E0CS', 'PC_00HGKK', 'PC_03813O', 'PC_01KTUJ', 'PC_0860OA', 'PC_04UWXG', 'PC_04PSFQ', 'PC_045HU3', 'PC_00UWAQ', 'PC_02BG1Z', 'PC_008806', 'PC_0278ZT', 'PC_01BZCN', 'PC_05W6XP', 'PC_073XSY', 'PC_08H11O', 'PC_02QW19', 'PC_01EAHK', 'PC_02UBXP', 'PC_02DAU3', 'PC_021V0R', 'PC_07TVF3', 'PC_00S0UG', 'PC_016TKH', 'PC_07TVA5', 'PC_03PBKY', 'PC_06SE34', 'PC_02K5A5', 'PC_07XIBT', 'PC_06ZU26', 'PC_06FIXN', 'PC_00595C', 'PC_05KEYA', 'PC_0804W2', 'PC_021SYL', 'PC_06WRVL', 'PC_06UZ3U', 'PC_07TVM3', 'PC_00SR1W', 'PC_00UFF9', 'PC_06K75W', 'PC_05N2GV', 'PC_04MR4A', 'PC_02G1YM', 'PC_01QQ1T', 'PC_05TZZ7', 'PC_05W661', 'PC_07D4R9', 'PC_02VB7G', 'PC_00VTNG', 'PC_03K305', 'PC_05MI4Z', 'PC_018DM6', 'PC_031XPV', 'PC_036385', 'PC_011LAW', 'PC_04OIFF', 'PC_07UMX8', 'PC_030ACC', 'PC_04SFDZ', 'PC_00YPBK', 'PC_04PJ1H', 'PC_03792B', 'PC_07AGJQ', 'PC_045E9O', 'PC_032XC4', 'PC_07NWKC', 'PC_06CM2C', 'PC_0654DH', 'PC_07UIKR', 'PC_04FB23', 'PC_00NN46', 'PC_04R5US', 'PC_03PEL5', 'PC_06SL6B', 'PC_05AVFK', 'PC_01YHQS', 'PC_00915C', 'PC_01J1VJ', 'PC_060UD5', 'PC_00646F', 'PC_024L0V', 'PC_05D714', 'PC_048XFW'}
MSA succeeded
Edge PC_01NR73 PC_07UMX8 0.08223079144954681
MSA failed
Edge PC_05TX0J PC_07AJXF 0.08219544589519501
lkey 349
lkey_clusters {'PC_060QAC', 'PC_01K875', 'PC_04V0PU', 'PC_01KBB8', 'PC_01296N', 'PC_013NPU', 'PC_00ZQQF', 'PC_08H9YG', 'PC_081PV4', 'PC_007Z8T', 'PC_032CKH', 'PC_018GH8', 'PC_02HVML', 'PC_01FQRU', 'PC_02W8LT', 'PC_005N2L', 'PC_07WEO1', 'PC_04Z3ZQ', 'PC_06OK2P', 'PC_00MO21', 'PC_04DQ0W', 'PC_059TG9', 'PC_06EWH0', 'PC_04U1L7', 'PC_07TV5W', 'PC_07AWUL', 'PC_036HDQ', 'PC_07VAB2', 'PC_01CDCN', 'PC_05R7KZ', 'PC_02V4AV', 'PC_05LDC6', 'PC_072EU7', 'PC_01J10M', 'PC_02T3ZI', 'PC_0060XJ', 'PC_05K9FR', 'PC_03NC9U', 'PC_02R0M8', 'PC_04LTCQ', 'PC_00A54M', 'PC_0370DG', 'PC_080PLJ', 'PC_057CGE', 'PC_02EMSJ', 'PC_06IRIY', 'PC_03AMPS', 'PC_07TV5U', 'PC_05VOPA', 'PC_053J9J', 'PC_04N53M', 'PC_022NID', 'PC_05F9H4', 'PC_022ZLX', 'PC_01WTO6', 'PC_07OCLR', 'PC_05FIVY', 'PC_085L07', 'PC_03870X', 'PC_01GLB1', 'PC_001RDT', 'PC_02OSJL', 'PC_04OJOB', 'PC_073NJA', 'PC_062QHL', 'PC_0708R1', 'PC_00LSXD', 'PC_063IUN', 'PC_05RS5N', 'PC_009036', 'PC_08I3IM', 'PC_05AJOI', 'PC_011686', 'PC_02AR9H', 'PC_0837FY', 'PC_074GF9', 'PC_02D1GK', 'PC_0753N9', 'PC_03EHDR', 'PC_023QTD', 'PC_02GRBP', 'PC_05O6MK', 'PC_027RDL', 'PC_02V4LH', 'PC_02KEHU', 'PC_01W301', 'PC_02JM6C', 'PC_05ILWE', 'PC_030G1S', 'PC_079ZWS', 'PC_02WCO0', 'PC_047570', 'PC_03JFL5', 'PC_07VJ0Q', 'PC_00A3UY', 'PC_03T6PW', 'PC_06ADE4', 'PC_02P3RH', 'PC_0294CS', 'PC_0473PG', 'PC_0393P5', 'PC_063TMD', 'PC_07TV9U', 'PC_07TV8B', 'PC_06J307', 'PC_087UOG', 'PC_06MGDC', 'PC_02UI5W', 'PC_0181PE', 'PC_05PJ7O', 'PC_0164QS', 'PC_0100UP', 'PC_02CC4R', 'PC_06AI9C', 'PC_00M7NJ', 'PC_03AR0I', 'PC_009LO1', 'PC_0085EC', 'PC_02BNS6', 'PC_01FJ7Z', 'PC_04ZZ0B', 'PC_07076R', 'PC_04USMD', 'PC_00UAB7', 'PC_01IC1B', 'PC_06LOIO', 'PC_05TX0J', 'PC_02EV3G', 'PC_017F5K', 'PC_02QMV0', 'PC_06BP65', 'PC_07A9N7', 'PC_04IINX', 'PC_001GNR', 'PC_0384LZ', 'PC_00772B', 'PC_05TFVL', 'PC_01GNRM', 'PC_042YRE', 'PC_07TUNO', 'PC_06DDSH', 'PC_05QB64', 'PC_04DUFN', 'PC_08I1Q6', 'PC_0843Z9', 'PC_07QLAB', 'PC_04E4JE', 'PC_039ZP9', 'PC_05MNXH', 'PC_07VHB5', 'PC_05KG1N', 'PC_06RYNI', 'PC_02LGPA', 'PC_02CEZD', 'PC_063JSC', 'PC_0490I5', 'PC_05OR8I', 'PC_05FXKZ', 'PC_07TV4U', 'PC_01D3OM', 'PC_06BL39', 'PC_04SK9M', 'PC_088DQF', 'PC_07TUPN', 'PC_04VBNR', 'PC_01Y0R1', 'PC_05RVL4', 'PC_057FE7', 'PC_05JFQY', 'PC_00HTJV', 'PC_02QIDH', 'PC_046RD9', 'PC_03E0CS', 'PC_00HGKK', 'PC_03813O', 'PC_01KTUJ', 'PC_0860OA', 'PC_04UWXG', 'PC_04PSFQ', 'PC_045HU3', 'PC_00UWAQ', 'PC_02BG1Z', 'PC_008806', 'PC_0278ZT', 'PC_01BZCN', 'PC_05W6XP', 'PC_073XSY', 'PC_08H11O', 'PC_02QW19', 'PC_01EAHK', 'PC_02UBXP', 'PC_02DAU3', 'PC_021V0R', 'PC_07TVF3', 'PC_04ULOH', 'PC_00S0UG', 'PC_016TKH', 'PC_07TVA5', 'PC_03PBKY', 'PC_06SE34', 'PC_02K5A5', 'PC_07XIBT', 'PC_06ZU26', 'PC_06FIXN', 'PC_00595C', 'PC_05KEYA', 'PC_0804W2', 'PC_021SYL', 'PC_06WRVL', 'PC_06UZ3U', 'PC_07TVM3', 'PC_00SR1W', 'PC_00UFF9', 'PC_06K75W', 'PC_05N2GV', 'PC_04MR4A', 'PC_02G1YM', 'PC_01QQ1T', 'PC_05TZZ7', 'PC_05W661', 'PC_07D4R9', 'PC_02VB7G', 'PC_00VTNG', 'PC_03K305', 'PC_05MI4Z', 'PC_018DM6', 'PC_031XPV', 'PC_036385', 'PC_011LAW', 'PC_04OIFF', 'PC_07UMX8', 'PC_030ACC', 'PC_04SFDZ', 'PC_00YPBK', 'PC_04PJ1H', 'PC_03792B', 'PC_07AGJQ', 'PC_045E9O', 'PC_01EZOK', 'PC_032XC4', 'PC_07NWKC', 'PC_06CM2C', 'PC_0654DH', 'PC_07UIKR', 'PC_04FB23', 'PC_00NN46', 'PC_04R5US', 'PC_03PEL5', 'PC_06SL6B', 'PC_05AVFK', 'PC_01YHQS', 'PC_00915C', 'PC_01J1VJ', 'PC_060UD5', 'PC_00646F', 'PC_024L0V', 'PC_05D714', 'PC_048XFW'}
MSA failed
Edge PC_03JFL5 PC_06187I 0.08052551001310349
lkey 349
lkey_clusters {'PC_060QAC', 'PC_01K875', 'PC_04V0PU', 'PC_01KBB8', 'PC_01296N', 'PC_013NPU', 'PC_00ZQQF', 'PC_08H9YG', 'PC_081PV4', 'PC_007Z8T', 'PC_032CKH', 'PC_018GH8', 'PC_02HVML', 'PC_01FQRU', 'PC_02W8LT', 'PC_005N2L', 'PC_07WEO1', 'PC_04Z3ZQ', 'PC_06OK2P', 'PC_00MO21', 'PC_04DQ0W', 'PC_059TG9', 'PC_06EWH0', 'PC_04U1L7', 'PC_07TV5W', 'PC_07AWUL', 'PC_036HDQ', 'PC_07VAB2', 'PC_01CDCN', 'PC_05R7KZ', 'PC_02V4AV', 'PC_05LDC6', 'PC_072EU7', 'PC_01J10M', 'PC_02T3ZI', 'PC_0060XJ', 'PC_05K9FR', 'PC_03NC9U', 'PC_02R0M8', 'PC_04LTCQ', 'PC_00A54M', 'PC_0370DG', 'PC_080PLJ', 'PC_057CGE', 'PC_02EMSJ', 'PC_06IRIY', 'PC_03AMPS', 'PC_07TV5U', 'PC_05VOPA', 'PC_053J9J', 'PC_04N53M', 'PC_022NID', 'PC_05F9H4', 'PC_022ZLX', 'PC_01WTO6', 'PC_07OCLR', 'PC_05FIVY', 'PC_085L07', 'PC_03870X', 'PC_01GLB1', 'PC_001RDT', 'PC_02OSJL', 'PC_04OJOB', 'PC_073NJA', 'PC_062QHL', 'PC_0708R1', 'PC_00LSXD', 'PC_063IUN', 'PC_05RS5N', 'PC_009036', 'PC_08I3IM', 'PC_05AJOI', 'PC_011686', 'PC_02AR9H', 'PC_0837FY', 'PC_074GF9', 'PC_02D1GK', 'PC_0753N9', 'PC_03EHDR', 'PC_023QTD', 'PC_02GRBP', 'PC_05O6MK', 'PC_027RDL', 'PC_02V4LH', 'PC_02KEHU', 'PC_01W301', 'PC_02JM6C', 'PC_05ILWE', 'PC_030G1S', 'PC_079ZWS', 'PC_02WCO0', 'PC_047570', 'PC_03JFL5', 'PC_07VJ0Q', 'PC_00A3UY', 'PC_03T6PW', 'PC_06ADE4', 'PC_02P3RH', 'PC_0294CS', 'PC_0473PG', 'PC_0393P5', 'PC_063TMD', 'PC_07TV9U', 'PC_07TV8B', 'PC_06J307', 'PC_087UOG', 'PC_06MGDC', 'PC_02UI5W', 'PC_0181PE', 'PC_05PJ7O', 'PC_0164QS', 'PC_0100UP', 'PC_02CC4R', 'PC_06AI9C', 'PC_00M7NJ', 'PC_03AR0I', 'PC_009LO1', 'PC_0085EC', 'PC_02BNS6', 'PC_01FJ7Z', 'PC_04ZZ0B', 'PC_07076R', 'PC_04USMD', 'PC_00UAB7', 'PC_01IC1B', 'PC_06LOIO', 'PC_05TX0J', 'PC_02EV3G', 'PC_017F5K', 'PC_02QMV0', 'PC_06BP65', 'PC_07A9N7', 'PC_04IINX', 'PC_001GNR', 'PC_0384LZ', 'PC_00772B', 'PC_05TFVL', 'PC_01GNRM', 'PC_042YRE', 'PC_07TUNO', 'PC_06DDSH', 'PC_05QB64', 'PC_04DUFN', 'PC_08I1Q6', 'PC_0843Z9', 'PC_07QLAB', 'PC_04E4JE', 'PC_039ZP9', 'PC_05MNXH', 'PC_07VHB5', 'PC_05KG1N', 'PC_06RYNI', 'PC_02LGPA', 'PC_02CEZD', 'PC_063JSC', 'PC_0490I5', 'PC_05OR8I', 'PC_05FXKZ', 'PC_07TV4U', 'PC_01D3OM', 'PC_06BL39', 'PC_04SK9M', 'PC_088DQF', 'PC_07TUPN', 'PC_04VBNR', 'PC_01Y0R1', 'PC_05RVL4', 'PC_057FE7', 'PC_05JFQY', 'PC_00HTJV', 'PC_02QIDH', 'PC_046RD9', 'PC_03E0CS', 'PC_00HGKK', 'PC_03813O', 'PC_01KTUJ', 'PC_0860OA', 'PC_04UWXG', 'PC_04PSFQ', 'PC_045HU3', 'PC_00UWAQ', 'PC_02BG1Z', 'PC_008806', 'PC_0278ZT', 'PC_01BZCN', 'PC_05W6XP', 'PC_073XSY', 'PC_08H11O', 'PC_02QW19', 'PC_01EAHK', 'PC_02UBXP', 'PC_02DAU3', 'PC_021V0R', 'PC_07TVF3', 'PC_04ULOH', 'PC_00S0UG', 'PC_016TKH', 'PC_07TVA5', 'PC_03PBKY', 'PC_06SE34', 'PC_02K5A5', 'PC_07XIBT', 'PC_06ZU26', 'PC_06FIXN', 'PC_00595C', 'PC_05KEYA', 'PC_0804W2', 'PC_021SYL', 'PC_06WRVL', 'PC_06UZ3U', 'PC_07TVM3', 'PC_00SR1W', 'PC_00UFF9', 'PC_06K75W', 'PC_05N2GV', 'PC_04MR4A', 'PC_02G1YM', 'PC_01QQ1T', 'PC_05TZZ7', 'PC_05W661', 'PC_07D4R9', 'PC_02VB7G', 'PC_00VTNG', 'PC_03K305', 'PC_05MI4Z', 'PC_018DM6', 'PC_031XPV', 'PC_036385', 'PC_011LAW', 'PC_04OIFF', 'PC_07UMX8', 'PC_030ACC', 'PC_04SFDZ', 'PC_00YPBK', 'PC_04PJ1H', 'PC_03792B', 'PC_07AGJQ', 'PC_045E9O', 'PC_01EZOK', 'PC_032XC4', 'PC_07NWKC', 'PC_06CM2C', 'PC_0654DH', 'PC_07UIKR', 'PC_04FB23', 'PC_00NN46', 'PC_04R5US', 'PC_03PEL5', 'PC_06SL6B', 'PC_05AVFK', 'PC_01YHQS', 'PC_00915C', 'PC_01J1VJ', 'PC_060UD5', 'PC_00646F', 'PC_024L0V', 'PC_05D714', 'PC_048XFW'}
MSA succeeded
Edge PC_05JUMB PC_07UIKR 0.07829568535089493
MSA failed
Edge PC_01WPL7 PC_030G1S 0.07401318103075027
lkey 346
lkey_clusters {'PC_05AOUJ', 'PC_057VS9', 'PC_04KM1I', 'PC_04M1B3', 'PC_05K4L1', 'PC_06WVB2', 'PC_00641O', 'PC_01OH6B', 'PC_060TP7', 'PC_016FRE', 'PC_04PM5Y', 'PC_0306GU', 'PC_01CVKZ', 'PC_0091OE', 'PC_06RYIX', 'PC_03QNGV', 'PC_002I3M', 'PC_07TV6Z', 'PC_072DWQ', 'PC_01PMZK', 'PC_002I4B', 'PC_00LBZX', 'PC_029RUP', 'PC_01FGFJ', 'PC_00NE3P', 'PC_02C8S3', 'PC_05XT7X', 'PC_06K136', 'PC_009BF9', 'PC_05VZ2W', 'PC_05DIQW', 'PC_02KYVT', 'PC_021EBE', 'PC_06UZ06', 'PC_05L7US', 'PC_04SSH3', 'PC_07TV02', 'PC_03HGF9', 'PC_02GYSI', 'PC_01VGHN', 'PC_01R1KR', 'PC_03970R', 'PC_074DQF', 'PC_02QUA0', 'PC_04THYL', 'PC_06HSND', 'PC_04HZ79', 'PC_02JQ3K', 'PC_05W9ZE', 'PC_04D7VO', 'PC_004U8P', 'PC_07TUTP', 'PC_03PSAN', 'PC_05MHF3', 'PC_05MVJQ', 'PC_00DU1R', 'PC_0109ZP', 'PC_06280D', 'PC_02XK62', 'PC_01WPL7', 'PC_051C8B'}
MSA succeeded
Edge PC_06CAC0 PC_08I3IM 0.07357081025838852
MSA failed
Edge PC_022TVQ PC_030G1S 0.07115544378757477
MSA succeeded
Edge PC_00XN01 PC_04PM5Y 0.05954016372561455
MSA succeeded
Edge PC_04BA1R PC_0804W2 0.2837305963039398
MSA succeeded
Edge PC_04V0PU PC_07A5YP 0.131278395652771
lkey 354
lkey_clusters {'PC_05K4L1', 'PC_060QAC', 'PC_01K875', 'PC_04V0PU', 'PC_06RYIX', 'PC_01KBB8', 'PC_03XWEE', 'PC_01PMZK', 'PC_01296N', 'PC_02C8S3', 'PC_013NPU', 'PC_00ZQQF', 'PC_08H9YG', 'PC_009BF9', 'PC_081PV4', 'PC_007Z8T', 'PC_032CKH', 'PC_018GH8', 'PC_02HVML', 'PC_01FQRU', 'PC_02W8LT', 'PC_005N2L', 'PC_07WEO1', 'PC_04Z3ZQ', 'PC_00XN01', 'PC_06OK2P', 'PC_00MO21', 'PC_04DQ0W', 'PC_00P0Q4', 'PC_059TG9', 'PC_06EWH0', 'PC_04U1L7', 'PC_07TV5W', 'PC_05MVJQ', 'PC_04SSH3', 'PC_07AWUL', 'PC_036HDQ', 'PC_07VAB2', 'PC_01CDCN', 'PC_04BA1R', 'PC_05R7KZ', 'PC_060TP7', 'PC_02V4AV', 'PC_05LDC6', 'PC_072EU7', 'PC_01CVKZ', 'PC_03QNGV', 'PC_07TV6Z', 'PC_01J10M', 'PC_02T3ZI', 'PC_0060XJ', 'PC_05K9FR', 'PC_03NC9U', 'PC_02R0M8', 'PC_01FGFJ', 'PC_04LTCQ', 'PC_00A54M', 'PC_0370DG', 'PC_080PLJ', 'PC_057CGE', 'PC_06UZ06', 'PC_04TBPN', 'PC_02EMSJ', 'PC_06IRIY', 'PC_03AMPS', 'PC_07TV5U', 'PC_05VOPA', 'PC_053J9J', 'PC_00DU1R', 'PC_02JQ3K', 'PC_04N53M', 'PC_05W9ZE', 'PC_022NID', 'PC_05F9H4', 'PC_07TUTP', 'PC_022ZLX', 'PC_01WTO6', 'PC_05MHF3', 'PC_07OCLR', 'PC_05FIVY', 'PC_04M1B3', 'PC_085L07', 'PC_03870X', 'PC_01GLB1', 'PC_001RDT', 'PC_02OSJL', 'PC_04OJOB', 'PC_073NJA', 'PC_062QHL', 'PC_0708R1', 'PC_00LSXD', 'PC_063IUN', 'PC_05RS5N', 'PC_009036', 'PC_08I3IM', 'PC_05AJOI', 'PC_029RUP', 'PC_011686', 'PC_02AR9H', 'PC_0837FY', 'PC_074GF9', 'PC_02D1GK', 'PC_0753N9', 'PC_03EHDR', 'PC_01VGHN', 'PC_023QTD', 'PC_02GRBP', 'PC_05O6MK', 'PC_06HSND', 'PC_027RDL', 'PC_02V4LH', 'PC_02KEHU', 'PC_01W301', 'PC_02JM6C', 'PC_02XK62', 'PC_05ILWE', 'PC_030G1S', 'PC_079ZWS', 'PC_02WCO0', 'PC_047570', 'PC_03JFL5', 'PC_00641O', 'PC_01OH6B', 'PC_07VJ0Q', 'PC_00A3UY', 'PC_03T6PW', 'PC_06ADE4', 'PC_02P3RH', 'PC_0306GU', 'PC_002I3M', 'PC_0294CS', 'PC_0473PG', 'PC_0393P5', 'PC_063TMD', 'PC_07TV9U', 'PC_00NE3P', 'PC_07TV8B', 'PC_06J307', 'PC_087UOG', 'PC_06MGDC', 'PC_02UI5W', 'PC_0181PE', 'PC_05PJ7O', 'PC_0164QS', 'PC_0100UP', 'PC_02CC4R', 'PC_06AI9C', 'PC_00M7NJ', 'PC_03970R', 'PC_03AR0I', 'PC_009LO1', 'PC_04THYL', 'PC_0085EC', 'PC_022TVQ', 'PC_02BNS6', 'PC_01FJ7Z', 'PC_04ZZ0B', 'PC_07076R', 'PC_04D7VO', 'PC_04USMD', 'PC_03PSAN', 'PC_00UAB7', 'PC_01IC1B', 'PC_06LOIO', 'PC_05TX0J', 'PC_02EV3G', 'PC_017F5K', 'PC_02QMV0', 'PC_05AOUJ', 'PC_06BP65', 'PC_07A9N7', 'PC_04IINX', 'PC_001GNR', 'PC_0384LZ', 'PC_00772B', 'PC_05TFVL', 'PC_01GNRM', 'PC_042YRE', 'PC_07TUNO', 'PC_0091OE', 'PC_06DDSH', 'PC_002I4B', 'PC_05QB64', 'PC_00LBZX', 'PC_04DUFN', 'PC_08I1Q6', 'PC_0843Z9', 'PC_07QLAB', 'PC_05VZ2W', 'PC_04E4JE', 'PC_039ZP9', 'PC_05MNXH', 'PC_07VHB5', 'PC_05KG1N', 'PC_06187I', 'PC_06RYNI', 'PC_02LGPA', 'PC_02CEZD', 'PC_074DQF', 'PC_063JSC', 'PC_0490I5', 'PC_05OR8I', 'PC_05FXKZ', 'PC_07TV4U', 'PC_01D3OM', 'PC_06280D', 'PC_06BL39', 'PC_04SK9M', 'PC_01WPL7', 'PC_088DQF', 'PC_04KM1I', 'PC_06WVB2', 'PC_07TUPN', 'PC_04VBNR', 'PC_01Y0R1', 'PC_016FRE', 'PC_05RVL4', 'PC_057FE7', 'PC_05JFQY', 'PC_04PM5Y', 'PC_00HTJV', 'PC_02QIDH', 'PC_046RD9', 'PC_03E0CS', 'PC_00HGKK', 'PC_03813O', 'PC_01KTUJ', 'PC_0860OA', 'PC_04UWXG', 'PC_04PSFQ', 'PC_02KYVT', 'PC_045HU3', 'PC_00UWAQ', 'PC_02BG1Z', 'PC_008806', 'PC_0278ZT', 'PC_07TV02', 'PC_01R1KR', 'PC_01BZCN', 'PC_05W6XP', 'PC_073XSY', 'PC_04HZ79', 'PC_08H11O', 'PC_02QW19', 'PC_01EAHK', 'PC_02UBXP', 'PC_02DAU3', 'PC_021V0R', 'PC_07TVF3', 'PC_04ULOH', 'PC_051C8B', 'PC_00S0UG', 'PC_016TKH', 'PC_07TVA5', 'PC_03PBKY', 'PC_06SE34', 'PC_02K5A5', 'PC_07XIBT', 'PC_06ZU26', 'PC_06FIXN', 'PC_00595C', 'PC_05KEYA', 'PC_0804W2', 'PC_021SYL', 'PC_06K136', 'PC_06WRVL', 'PC_06UZ3U', 'PC_07TVM3', 'PC_05DIQW', 'PC_021EBE', 'PC_00SR1W', 'PC_00UFF9', 'PC_06K75W', 'PC_05N2GV', 'PC_04MR4A', 'PC_02G1YM', 'PC_01QQ1T', 'PC_05TZZ7', 'PC_05W661', 'PC_07D4R9', 'PC_02VB7G', 'PC_02QUA0', 'PC_00VTNG', 'PC_03K305', 'PC_05MI4Z', 'PC_018DM6', 'PC_031XPV', 'PC_036385', 'PC_011LAW', 'PC_04OIFF', 'PC_07UMX8', 'PC_030ACC', 'PC_04SFDZ', 'PC_00YPBK', 'PC_04PJ1H', 'PC_026AIN', 'PC_03792B', 'PC_07AGJQ', 'PC_045E9O', 'PC_057VS9', 'PC_01EZOK', 'PC_032XC4', 'PC_07NWKC', 'PC_06CM2C', 'PC_0654DH', 'PC_07UIKR', 'PC_072DWQ', 'PC_04FB23', 'PC_00NN46', 'PC_04R5US', 'PC_03PEL5', 'PC_06SL6B', 'PC_05L7US', 'PC_05AVFK', 'PC_03HGF9', 'PC_02GYSI', 'PC_01YHQS', 'PC_00915C', 'PC_01J1VJ', 'PC_004U8P', 'PC_060UD5', 'PC_00646F', 'PC_024L0V', 'PC_05D714', 'PC_0109ZP', 'PC_048XFW', 'PC_05XT7X'}
MSA succeeded
Edge PC_01KP4T PC_0843Z9 0.12539885938167572
MSA succeeded
Edge PC_007Z8T PC_03ALOO 0.11853956431150436
lkey 356
lkey_clusters {'PC_05K4L1', 'PC_060QAC', 'PC_01K875', 'PC_04V0PU', 'PC_06RYIX', 'PC_01KBB8', 'PC_03XWEE', 'PC_01PMZK', 'PC_01296N', 'PC_02C8S3', 'PC_013NPU', 'PC_00ZQQF', 'PC_08H9YG', 'PC_009BF9', 'PC_081PV4', 'PC_007Z8T', 'PC_032CKH', 'PC_018GH8', 'PC_02HVML', 'PC_01FQRU', 'PC_02W8LT', 'PC_005N2L', 'PC_07WEO1', 'PC_04Z3ZQ', 'PC_00XN01', 'PC_06OK2P', 'PC_00MO21', 'PC_04DQ0W', 'PC_00P0Q4', 'PC_059TG9', 'PC_06EWH0', 'PC_04U1L7', 'PC_07TV5W', 'PC_05MVJQ', 'PC_04SSH3', 'PC_07AWUL', 'PC_036HDQ', 'PC_07VAB2', 'PC_01CDCN', 'PC_04BA1R', 'PC_05R7KZ', 'PC_060TP7', 'PC_02V4AV', 'PC_05LDC6', 'PC_072EU7', 'PC_01CVKZ', 'PC_03QNGV', 'PC_07TV6Z', 'PC_01J10M', 'PC_02T3ZI', 'PC_0060XJ', 'PC_05K9FR', 'PC_03NC9U', 'PC_02R0M8', 'PC_01FGFJ', 'PC_04LTCQ', 'PC_00A54M', 'PC_0370DG', 'PC_080PLJ', 'PC_057CGE', 'PC_06UZ06', 'PC_04TBPN', 'PC_02EMSJ', 'PC_06IRIY', 'PC_03AMPS', 'PC_07TV5U', 'PC_05VOPA', 'PC_053J9J', 'PC_02JQ3K', 'PC_04N53M', 'PC_05W9ZE', 'PC_022NID', 'PC_05F9H4', 'PC_07TUTP', 'PC_022ZLX', 'PC_01WTO6', 'PC_05MHF3', 'PC_07OCLR', 'PC_05FIVY', 'PC_04M1B3', 'PC_085L07', 'PC_03870X', 'PC_01GLB1', 'PC_001RDT', 'PC_02OSJL', 'PC_04OJOB', 'PC_073NJA', 'PC_062QHL', 'PC_0708R1', 'PC_00LSXD', 'PC_063IUN', 'PC_05RS5N', 'PC_009036', 'PC_08I3IM', 'PC_05AJOI', 'PC_029RUP', 'PC_011686', 'PC_02AR9H', 'PC_0837FY', 'PC_074GF9', 'PC_02D1GK', 'PC_0753N9', 'PC_03EHDR', 'PC_01VGHN', 'PC_023QTD', 'PC_02GRBP', 'PC_05O6MK', 'PC_06HSND', 'PC_027RDL', 'PC_02V4LH', 'PC_02KEHU', 'PC_024L0V', 'PC_01W301', 'PC_02JM6C', 'PC_02XK62', 'PC_05ILWE', 'PC_030G1S', 'PC_079ZWS', 'PC_02WCO0', 'PC_047570', 'PC_03JFL5', 'PC_00641O', 'PC_01OH6B', 'PC_07VJ0Q', 'PC_00A3UY', 'PC_03T6PW', 'PC_06ADE4', 'PC_02P3RH', 'PC_0306GU', 'PC_01KP4T', 'PC_002I3M', 'PC_0294CS', 'PC_0473PG', 'PC_0393P5', 'PC_063TMD', 'PC_07TV9U', 'PC_00NE3P', 'PC_07TV8B', 'PC_06J307', 'PC_087UOG', 'PC_06MGDC', 'PC_02UI5W', 'PC_0181PE', 'PC_05PJ7O', 'PC_0164QS', 'PC_0100UP', 'PC_02CC4R', 'PC_06AI9C', 'PC_00M7NJ', 'PC_03970R', 'PC_03AR0I', 'PC_009LO1', 'PC_04THYL', 'PC_0085EC', 'PC_022TVQ', 'PC_02BNS6', 'PC_01FJ7Z', 'PC_04ZZ0B', 'PC_07076R', 'PC_04D7VO', 'PC_04USMD', 'PC_03PSAN', 'PC_00UAB7', 'PC_01IC1B', 'PC_06LOIO', 'PC_05TX0J', 'PC_02EV3G', 'PC_017F5K', 'PC_02QMV0', 'PC_05AOUJ', 'PC_06BP65', 'PC_07A9N7', 'PC_04IINX', 'PC_001GNR', 'PC_0384LZ', 'PC_00772B', 'PC_05TFVL', 'PC_01GNRM', 'PC_042YRE', 'PC_07TUNO', 'PC_0091OE', 'PC_06DDSH', 'PC_002I4B', 'PC_05QB64', 'PC_00LBZX', 'PC_04DUFN', 'PC_08I1Q6', 'PC_0843Z9', 'PC_07QLAB', 'PC_05VZ2W', 'PC_04E4JE', 'PC_039ZP9', 'PC_05MNXH', 'PC_07VHB5', 'PC_05KG1N', 'PC_06187I', 'PC_06RYNI', 'PC_02LGPA', 'PC_02CEZD', 'PC_074DQF', 'PC_063JSC', 'PC_0490I5', 'PC_05OR8I', 'PC_05FXKZ', 'PC_07TV4U', 'PC_01D3OM', 'PC_06280D', 'PC_06BL39', 'PC_04SK9M', 'PC_01WPL7', 'PC_088DQF', 'PC_04KM1I', 'PC_06WVB2', 'PC_07TUPN', 'PC_04VBNR', 'PC_07A5YP', 'PC_01Y0R1', 'PC_016FRE', 'PC_05RVL4', 'PC_057FE7', 'PC_05JFQY', 'PC_04PM5Y', 'PC_00HTJV', 'PC_02QIDH', 'PC_046RD9', 'PC_03E0CS', 'PC_00HGKK', 'PC_03813O', 'PC_01KTUJ', 'PC_0860OA', 'PC_04UWXG', 'PC_04PSFQ', 'PC_02KYVT', 'PC_045HU3', 'PC_00UWAQ', 'PC_02BG1Z', 'PC_008806', 'PC_0278ZT', 'PC_07TV02', 'PC_01R1KR', 'PC_01BZCN', 'PC_05W6XP', 'PC_073XSY', 'PC_04HZ79', 'PC_08H11O', 'PC_02QW19', 'PC_01EAHK', 'PC_02UBXP', 'PC_02DAU3', 'PC_021V0R', 'PC_07TVF3', 'PC_04ULOH', 'PC_051C8B', 'PC_00S0UG', 'PC_016TKH', 'PC_07TVA5', 'PC_03PBKY', 'PC_06SE34', 'PC_02K5A5', 'PC_07XIBT', 'PC_06ZU26', 'PC_06FIXN', 'PC_00595C', 'PC_05KEYA', 'PC_0804W2', 'PC_021SYL', 'PC_06K136', 'PC_06WRVL', 'PC_06UZ3U', 'PC_07TVM3', 'PC_05DIQW', 'PC_021EBE', 'PC_00SR1W', 'PC_00UFF9', 'PC_06K75W', 'PC_05N2GV', 'PC_04MR4A', 'PC_02G1YM', 'PC_01QQ1T', 'PC_05TZZ7', 'PC_05W661', 'PC_07D4R9', 'PC_02VB7G', 'PC_02QUA0', 'PC_00VTNG', 'PC_03K305', 'PC_05MI4Z', 'PC_018DM6', 'PC_031XPV', 'PC_036385', 'PC_011LAW', 'PC_04OIFF', 'PC_07UMX8', 'PC_030ACC', 'PC_04SFDZ', 'PC_00YPBK', 'PC_04PJ1H', 'PC_026AIN', 'PC_03792B', 'PC_07AGJQ', 'PC_045E9O', 'PC_057VS9', 'PC_01EZOK', 'PC_032XC4', 'PC_07NWKC', 'PC_06CM2C', 'PC_0654DH', 'PC_07UIKR', 'PC_072DWQ', 'PC_04FB23', 'PC_00NN46', 'PC_04R5US', 'PC_03PEL5', 'PC_06SL6B', 'PC_05L7US', 'PC_05AVFK', 'PC_03HGF9', 'PC_02GYSI', 'PC_01YHQS', 'PC_00915C', 'PC_01J1VJ', 'PC_004U8P', 'PC_060UD5', 'PC_00646F', 'PC_00DU1R', 'PC_05D714', 'PC_0109ZP', 'PC_048XFW', 'PC_05XT7X'}
MSA succeeded
Edge PC_03GTZD PC_04SK9M 0.11814968287944794
lkey 306
lkey_clusters {'PC_03GTZD', 'PC_04L2GD', 'PC_0519TF', 'PC_024FEH', 'PC_02RGUO', 'PC_02NATZ', 'PC_05X98I', 'PC_07TUWD', 'PC_02YR8I', 'PC_041GAL', 'PC_02Q6QH', 'PC_012M5T', 'PC_029YIZ', 'PC_00QDYY', 'PC_01FFWH', 'PC_03FKVQ', 'PC_00D5EQ', 'PC_06PIV6', 'PC_003XGF', 'PC_00QNZJ', 'PC_0287UU', 'PC_01OCWE', 'PC_064DUW'}
MSA failed
Edge PC_03TCZP PC_05TX0J 0.11416839808225632
MSA failed
Edge PC_02WPLI PC_04V0PU 0.09385459870100021
MSA failed
Edge PC_018GH8 PC_0574V4 0.09232421219348907
lkey 357
lkey_clusters {'PC_05K4L1', 'PC_060QAC', 'PC_01K875', 'PC_04V0PU', 'PC_06RYIX', 'PC_01KBB8', 'PC_03XWEE', 'PC_01PMZK', 'PC_01296N', 'PC_02C8S3', 'PC_013NPU', 'PC_00ZQQF', 'PC_08H9YG', 'PC_009BF9', 'PC_081PV4', 'PC_007Z8T', 'PC_032CKH', 'PC_018GH8', 'PC_02HVML', 'PC_01FQRU', 'PC_02W8LT', 'PC_005N2L', 'PC_07WEO1', 'PC_04Z3ZQ', 'PC_00XN01', 'PC_06OK2P', 'PC_00MO21', 'PC_04DQ0W', 'PC_00P0Q4', 'PC_059TG9', 'PC_06EWH0', 'PC_04U1L7', 'PC_07TV5W', 'PC_05MVJQ', 'PC_04SSH3', 'PC_07AWUL', 'PC_036HDQ', 'PC_07VAB2', 'PC_01CDCN', 'PC_04BA1R', 'PC_05R7KZ', 'PC_060TP7', 'PC_02V4AV', 'PC_05LDC6', 'PC_072EU7', 'PC_01CVKZ', 'PC_03QNGV', 'PC_07TV6Z', 'PC_01J10M', 'PC_02T3ZI', 'PC_0060XJ', 'PC_05K9FR', 'PC_03NC9U', 'PC_02R0M8', 'PC_01FGFJ', 'PC_04LTCQ', 'PC_00A54M', 'PC_0370DG', 'PC_080PLJ', 'PC_057CGE', 'PC_06UZ06', 'PC_04TBPN', 'PC_02EMSJ', 'PC_06IRIY', 'PC_03AMPS', 'PC_07TV5U', 'PC_05VOPA', 'PC_053J9J', 'PC_02JQ3K', 'PC_04N53M', 'PC_05W9ZE', 'PC_022NID', 'PC_05F9H4', 'PC_07TUTP', 'PC_022ZLX', 'PC_01WTO6', 'PC_05MHF3', 'PC_07OCLR', 'PC_05FIVY', 'PC_04M1B3', 'PC_085L07', 'PC_03870X', 'PC_01GLB1', 'PC_001RDT', 'PC_02OSJL', 'PC_04OJOB', 'PC_073NJA', 'PC_062QHL', 'PC_0708R1', 'PC_00LSXD', 'PC_063IUN', 'PC_05RS5N', 'PC_009036', 'PC_08I3IM', 'PC_05AJOI', 'PC_029RUP', 'PC_011686', 'PC_02AR9H', 'PC_0837FY', 'PC_074GF9', 'PC_02D1GK', 'PC_0753N9', 'PC_03EHDR', 'PC_01VGHN', 'PC_023QTD', 'PC_02GRBP', 'PC_05O6MK', 'PC_06HSND', 'PC_027RDL', 'PC_02V4LH', 'PC_02KEHU', 'PC_024L0V', 'PC_01W301', 'PC_02JM6C', 'PC_02XK62', 'PC_05ILWE', 'PC_030G1S', 'PC_079ZWS', 'PC_02WCO0', 'PC_047570', 'PC_03JFL5', 'PC_00641O', 'PC_01OH6B', 'PC_07VJ0Q', 'PC_00A3UY', 'PC_03T6PW', 'PC_06ADE4', 'PC_02P3RH', 'PC_0306GU', 'PC_01KP4T', 'PC_002I3M', 'PC_0294CS', 'PC_0473PG', 'PC_0393P5', 'PC_063TMD', 'PC_07TV9U', 'PC_00NE3P', 'PC_07TV8B', 'PC_06J307', 'PC_087UOG', 'PC_06MGDC', 'PC_02UI5W', 'PC_0181PE', 'PC_05PJ7O', 'PC_0164QS', 'PC_0100UP', 'PC_02CC4R', 'PC_06AI9C', 'PC_00M7NJ', 'PC_03970R', 'PC_03AR0I', 'PC_009LO1', 'PC_04THYL', 'PC_0085EC', 'PC_022TVQ', 'PC_02BNS6', 'PC_01FJ7Z', 'PC_04ZZ0B', 'PC_07076R', 'PC_04D7VO', 'PC_04USMD', 'PC_03PSAN', 'PC_00UAB7', 'PC_01IC1B', 'PC_06LOIO', 'PC_05TX0J', 'PC_02EV3G', 'PC_017F5K', 'PC_02QMV0', 'PC_05AOUJ', 'PC_06BP65', 'PC_07A9N7', 'PC_04IINX', 'PC_001GNR', 'PC_0384LZ', 'PC_00772B', 'PC_05TFVL', 'PC_01GNRM', 'PC_042YRE', 'PC_07TUNO', 'PC_0091OE', 'PC_06DDSH', 'PC_002I4B', 'PC_05QB64', 'PC_00LBZX', 'PC_04DUFN', 'PC_08I1Q6', 'PC_0843Z9', 'PC_07QLAB', 'PC_05VZ2W', 'PC_04E4JE', 'PC_039ZP9', 'PC_05MNXH', 'PC_07VHB5', 'PC_05KG1N', 'PC_06187I', 'PC_06RYNI', 'PC_02LGPA', 'PC_02CEZD', 'PC_074DQF', 'PC_03ALOO', 'PC_063JSC', 'PC_0490I5', 'PC_05OR8I', 'PC_05FXKZ', 'PC_07TV4U', 'PC_01D3OM', 'PC_06280D', 'PC_06BL39', 'PC_04SK9M', 'PC_01WPL7', 'PC_088DQF', 'PC_04KM1I', 'PC_06WVB2', 'PC_07TUPN', 'PC_04VBNR', 'PC_07A5YP', 'PC_01Y0R1', 'PC_016FRE', 'PC_05RVL4', 'PC_057FE7', 'PC_05JFQY', 'PC_04PM5Y', 'PC_00HTJV', 'PC_02QIDH', 'PC_046RD9', 'PC_03E0CS', 'PC_00HGKK', 'PC_03813O', 'PC_01KTUJ', 'PC_0860OA', 'PC_04UWXG', 'PC_04PSFQ', 'PC_02KYVT', 'PC_045HU3', 'PC_00UWAQ', 'PC_02BG1Z', 'PC_008806', 'PC_0278ZT', 'PC_07TV02', 'PC_01R1KR', 'PC_01BZCN', 'PC_05W6XP', 'PC_073XSY', 'PC_04HZ79', 'PC_08H11O', 'PC_02QW19', 'PC_01EAHK', 'PC_02UBXP', 'PC_02DAU3', 'PC_021V0R', 'PC_07TVF3', 'PC_04ULOH', 'PC_051C8B', 'PC_00S0UG', 'PC_016TKH', 'PC_07TVA5', 'PC_03PBKY', 'PC_06SE34', 'PC_02K5A5', 'PC_07XIBT', 'PC_06ZU26', 'PC_06FIXN', 'PC_00595C', 'PC_05KEYA', 'PC_0804W2', 'PC_021SYL', 'PC_06K136', 'PC_06WRVL', 'PC_06UZ3U', 'PC_07TVM3', 'PC_05DIQW', 'PC_021EBE', 'PC_00SR1W', 'PC_00UFF9', 'PC_06K75W', 'PC_05N2GV', 'PC_04MR4A', 'PC_02G1YM', 'PC_01QQ1T', 'PC_05TZZ7', 'PC_05W661', 'PC_07D4R9', 'PC_02VB7G', 'PC_02QUA0', 'PC_00VTNG', 'PC_03K305', 'PC_05MI4Z', 'PC_018DM6', 'PC_031XPV', 'PC_036385', 'PC_011LAW', 'PC_04OIFF', 'PC_07UMX8', 'PC_030ACC', 'PC_04SFDZ', 'PC_00YPBK', 'PC_04PJ1H', 'PC_026AIN', 'PC_03792B', 'PC_07AGJQ', 'PC_045E9O', 'PC_057VS9', 'PC_01EZOK', 'PC_032XC4', 'PC_07NWKC', 'PC_06CM2C', 'PC_0654DH', 'PC_07UIKR', 'PC_072DWQ', 'PC_04FB23', 'PC_00NN46', 'PC_04R5US', 'PC_03PEL5', 'PC_06SL6B', 'PC_05L7US', 'PC_05AVFK', 'PC_03HGF9', 'PC_02GYSI', 'PC_01YHQS', 'PC_00915C', 'PC_01J1VJ', 'PC_004U8P', 'PC_060UD5', 'PC_00646F', 'PC_00DU1R', 'PC_05D714', 'PC_0109ZP', 'PC_048XFW', 'PC_05XT7X'}
MSA failed
Edge PC_03G9RN PC_072EU7 0.08931116759777069
lkey 317
lkey_clusters {'PC_03KYEF', 'PC_05PFLL', 'PC_08K2VM', 'PC_02NISQ', 'PC_03GU7O', 'PC_00QJR6', 'PC_07088H', 'PC_03G9RN'}
MSA failed
Edge PC_02EMSJ PC_06BZLT 0.08916065096855164
lkey 357
lkey_clusters {'PC_05K4L1', 'PC_060QAC', 'PC_01K875', 'PC_04V0PU', 'PC_06RYIX', 'PC_01KBB8', 'PC_03XWEE', 'PC_01PMZK', 'PC_01296N', 'PC_02C8S3', 'PC_013NPU', 'PC_00ZQQF', 'PC_08H9YG', 'PC_009BF9', 'PC_081PV4', 'PC_007Z8T', 'PC_032CKH', 'PC_018GH8', 'PC_02HVML', 'PC_01FQRU', 'PC_02W8LT', 'PC_005N2L', 'PC_07WEO1', 'PC_04Z3ZQ', 'PC_00XN01', 'PC_06OK2P', 'PC_00MO21', 'PC_04DQ0W', 'PC_00P0Q4', 'PC_059TG9', 'PC_06EWH0', 'PC_04U1L7', 'PC_07TV5W', 'PC_05MVJQ', 'PC_04SSH3', 'PC_07AWUL', 'PC_036HDQ', 'PC_07VAB2', 'PC_01CDCN', 'PC_04BA1R', 'PC_05R7KZ', 'PC_060TP7', 'PC_02V4AV', 'PC_05LDC6', 'PC_072EU7', 'PC_01CVKZ', 'PC_03QNGV', 'PC_07TV6Z', 'PC_01J10M', 'PC_02T3ZI', 'PC_0060XJ', 'PC_05K9FR', 'PC_03NC9U', 'PC_02R0M8', 'PC_01FGFJ', 'PC_04LTCQ', 'PC_00A54M', 'PC_0370DG', 'PC_080PLJ', 'PC_057CGE', 'PC_06UZ06', 'PC_04TBPN', 'PC_02EMSJ', 'PC_06IRIY', 'PC_03AMPS', 'PC_07TV5U', 'PC_05VOPA', 'PC_053J9J', 'PC_02JQ3K', 'PC_04N53M', 'PC_05W9ZE', 'PC_022NID', 'PC_05F9H4', 'PC_07TUTP', 'PC_022ZLX', 'PC_01WTO6', 'PC_05MHF3', 'PC_07OCLR', 'PC_05FIVY', 'PC_04M1B3', 'PC_085L07', 'PC_03870X', 'PC_01GLB1', 'PC_001RDT', 'PC_02OSJL', 'PC_04OJOB', 'PC_073NJA', 'PC_062QHL', 'PC_0708R1', 'PC_00LSXD', 'PC_063IUN', 'PC_05RS5N', 'PC_009036', 'PC_08I3IM', 'PC_05AJOI', 'PC_029RUP', 'PC_011686', 'PC_02AR9H', 'PC_0837FY', 'PC_074GF9', 'PC_02D1GK', 'PC_0753N9', 'PC_03EHDR', 'PC_01VGHN', 'PC_023QTD', 'PC_02GRBP', 'PC_05O6MK', 'PC_06HSND', 'PC_027RDL', 'PC_02V4LH', 'PC_02KEHU', 'PC_024L0V', 'PC_01W301', 'PC_02JM6C', 'PC_02XK62', 'PC_05ILWE', 'PC_030G1S', 'PC_079ZWS', 'PC_02WCO0', 'PC_047570', 'PC_03JFL5', 'PC_00641O', 'PC_01OH6B', 'PC_07VJ0Q', 'PC_00A3UY', 'PC_03T6PW', 'PC_06ADE4', 'PC_02P3RH', 'PC_0306GU', 'PC_01KP4T', 'PC_002I3M', 'PC_0294CS', 'PC_0473PG', 'PC_0393P5', 'PC_063TMD', 'PC_07TV9U', 'PC_00NE3P', 'PC_07TV8B', 'PC_06J307', 'PC_087UOG', 'PC_06MGDC', 'PC_02UI5W', 'PC_0181PE', 'PC_05PJ7O', 'PC_0164QS', 'PC_0100UP', 'PC_02CC4R', 'PC_06AI9C', 'PC_00M7NJ', 'PC_03970R', 'PC_03AR0I', 'PC_009LO1', 'PC_04THYL', 'PC_0085EC', 'PC_022TVQ', 'PC_02BNS6', 'PC_01FJ7Z', 'PC_04ZZ0B', 'PC_07076R', 'PC_04D7VO', 'PC_04USMD', 'PC_03PSAN', 'PC_00UAB7', 'PC_01IC1B', 'PC_06LOIO', 'PC_05TX0J', 'PC_02EV3G', 'PC_017F5K', 'PC_02QMV0', 'PC_05AOUJ', 'PC_06BP65', 'PC_07A9N7', 'PC_04IINX', 'PC_001GNR', 'PC_0384LZ', 'PC_00772B', 'PC_05TFVL', 'PC_01GNRM', 'PC_042YRE', 'PC_07TUNO', 'PC_0091OE', 'PC_06DDSH', 'PC_002I4B', 'PC_05QB64', 'PC_00LBZX', 'PC_04DUFN', 'PC_08I1Q6', 'PC_0843Z9', 'PC_07QLAB', 'PC_05VZ2W', 'PC_04E4JE', 'PC_039ZP9', 'PC_05MNXH', 'PC_07VHB5', 'PC_05KG1N', 'PC_06187I', 'PC_06RYNI', 'PC_02LGPA', 'PC_02CEZD', 'PC_074DQF', 'PC_03ALOO', 'PC_063JSC', 'PC_0490I5', 'PC_05OR8I', 'PC_05FXKZ', 'PC_07TV4U', 'PC_01D3OM', 'PC_06280D', 'PC_06BL39', 'PC_04SK9M', 'PC_01WPL7', 'PC_088DQF', 'PC_04KM1I', 'PC_06WVB2', 'PC_07TUPN', 'PC_04VBNR', 'PC_07A5YP', 'PC_01Y0R1', 'PC_016FRE', 'PC_05RVL4', 'PC_057FE7', 'PC_05JFQY', 'PC_04PM5Y', 'PC_00HTJV', 'PC_02QIDH', 'PC_046RD9', 'PC_03E0CS', 'PC_00HGKK', 'PC_03813O', 'PC_01KTUJ', 'PC_0860OA', 'PC_04UWXG', 'PC_04PSFQ', 'PC_02KYVT', 'PC_045HU3', 'PC_00UWAQ', 'PC_02BG1Z', 'PC_008806', 'PC_0278ZT', 'PC_07TV02', 'PC_01R1KR', 'PC_01BZCN', 'PC_05W6XP', 'PC_073XSY', 'PC_04HZ79', 'PC_08H11O', 'PC_02QW19', 'PC_01EAHK', 'PC_02UBXP', 'PC_02DAU3', 'PC_021V0R', 'PC_07TVF3', 'PC_04ULOH', 'PC_051C8B', 'PC_00S0UG', 'PC_016TKH', 'PC_07TVA5', 'PC_03PBKY', 'PC_06SE34', 'PC_02K5A5', 'PC_07XIBT', 'PC_06ZU26', 'PC_06FIXN', 'PC_00595C', 'PC_05KEYA', 'PC_0804W2', 'PC_021SYL', 'PC_06K136', 'PC_06WRVL', 'PC_06UZ3U', 'PC_07TVM3', 'PC_05DIQW', 'PC_021EBE', 'PC_00SR1W', 'PC_00UFF9', 'PC_06K75W', 'PC_05N2GV', 'PC_04MR4A', 'PC_02G1YM', 'PC_01QQ1T', 'PC_05TZZ7', 'PC_05W661', 'PC_07D4R9', 'PC_02VB7G', 'PC_02QUA0', 'PC_00VTNG', 'PC_03K305', 'PC_05MI4Z', 'PC_018DM6', 'PC_031XPV', 'PC_036385', 'PC_011LAW', 'PC_04OIFF', 'PC_07UMX8', 'PC_030ACC', 'PC_04SFDZ', 'PC_00YPBK', 'PC_04PJ1H', 'PC_026AIN', 'PC_03792B', 'PC_07AGJQ', 'PC_045E9O', 'PC_057VS9', 'PC_01EZOK', 'PC_032XC4', 'PC_07NWKC', 'PC_06CM2C', 'PC_0654DH', 'PC_07UIKR', 'PC_072DWQ', 'PC_04FB23', 'PC_00NN46', 'PC_04R5US', 'PC_03PEL5', 'PC_06SL6B', 'PC_05L7US', 'PC_05AVFK', 'PC_03HGF9', 'PC_02GYSI', 'PC_01YHQS', 'PC_00915C', 'PC_01J1VJ', 'PC_004U8P', 'PC_060UD5', 'PC_00646F', 'PC_00DU1R', 'PC_05D714', 'PC_0109ZP', 'PC_048XFW', 'PC_05XT7X'}
MSA failed
Edge PC_01NR73 PC_07UMX8 0.08223079144954681
MSA failed
Edge PC_05TX0J PC_07AJXF 0.08219544589519501
lkey 357
lkey_clusters {'PC_05K4L1', 'PC_060QAC', 'PC_01K875', 'PC_04V0PU', 'PC_06RYIX', 'PC_01KBB8', 'PC_03XWEE', 'PC_01PMZK', 'PC_01296N', 'PC_02C8S3', 'PC_013NPU', 'PC_00ZQQF', 'PC_08H9YG', 'PC_009BF9', 'PC_081PV4', 'PC_007Z8T', 'PC_032CKH', 'PC_018GH8', 'PC_02HVML', 'PC_01FQRU', 'PC_02W8LT', 'PC_005N2L', 'PC_07WEO1', 'PC_04Z3ZQ', 'PC_00XN01', 'PC_06OK2P', 'PC_00MO21', 'PC_04DQ0W', 'PC_00P0Q4', 'PC_059TG9', 'PC_06EWH0', 'PC_04U1L7', 'PC_07TV5W', 'PC_05MVJQ', 'PC_04SSH3', 'PC_07AWUL', 'PC_036HDQ', 'PC_07VAB2', 'PC_01CDCN', 'PC_04BA1R', 'PC_05R7KZ', 'PC_060TP7', 'PC_02V4AV', 'PC_05LDC6', 'PC_072EU7', 'PC_01CVKZ', 'PC_03QNGV', 'PC_07TV6Z', 'PC_01J10M', 'PC_02T3ZI', 'PC_0060XJ', 'PC_05K9FR', 'PC_03NC9U', 'PC_02R0M8', 'PC_01FGFJ', 'PC_04LTCQ', 'PC_00A54M', 'PC_0370DG', 'PC_080PLJ', 'PC_057CGE', 'PC_06UZ06', 'PC_04TBPN', 'PC_02EMSJ', 'PC_06IRIY', 'PC_03AMPS', 'PC_07TV5U', 'PC_05VOPA', 'PC_053J9J', 'PC_02JQ3K', 'PC_04N53M', 'PC_05W9ZE', 'PC_022NID', 'PC_05F9H4', 'PC_07TUTP', 'PC_022ZLX', 'PC_01WTO6', 'PC_05MHF3', 'PC_07OCLR', 'PC_05FIVY', 'PC_04M1B3', 'PC_085L07', 'PC_03870X', 'PC_01GLB1', 'PC_001RDT', 'PC_02OSJL', 'PC_04OJOB', 'PC_073NJA', 'PC_062QHL', 'PC_0708R1', 'PC_00LSXD', 'PC_063IUN', 'PC_05RS5N', 'PC_009036', 'PC_08I3IM', 'PC_05AJOI', 'PC_029RUP', 'PC_011686', 'PC_02AR9H', 'PC_0837FY', 'PC_074GF9', 'PC_02D1GK', 'PC_0753N9', 'PC_03EHDR', 'PC_01VGHN', 'PC_023QTD', 'PC_02GRBP', 'PC_05O6MK', 'PC_06HSND', 'PC_027RDL', 'PC_02V4LH', 'PC_02KEHU', 'PC_024L0V', 'PC_01W301', 'PC_02JM6C', 'PC_02XK62', 'PC_05ILWE', 'PC_030G1S', 'PC_079ZWS', 'PC_02WCO0', 'PC_047570', 'PC_03JFL5', 'PC_00641O', 'PC_01OH6B', 'PC_07VJ0Q', 'PC_00A3UY', 'PC_03T6PW', 'PC_06ADE4', 'PC_02P3RH', 'PC_0306GU', 'PC_01KP4T', 'PC_002I3M', 'PC_0294CS', 'PC_0473PG', 'PC_0393P5', 'PC_063TMD', 'PC_07TV9U', 'PC_00NE3P', 'PC_07TV8B', 'PC_06J307', 'PC_087UOG', 'PC_06MGDC', 'PC_02UI5W', 'PC_0181PE', 'PC_05PJ7O', 'PC_0164QS', 'PC_0100UP', 'PC_02CC4R', 'PC_06AI9C', 'PC_00M7NJ', 'PC_03970R', 'PC_03AR0I', 'PC_009LO1', 'PC_04THYL', 'PC_0085EC', 'PC_022TVQ', 'PC_02BNS6', 'PC_01FJ7Z', 'PC_04ZZ0B', 'PC_07076R', 'PC_04D7VO', 'PC_04USMD', 'PC_03PSAN', 'PC_00UAB7', 'PC_01IC1B', 'PC_06LOIO', 'PC_05TX0J', 'PC_02EV3G', 'PC_017F5K', 'PC_02QMV0', 'PC_05AOUJ', 'PC_06BP65', 'PC_07A9N7', 'PC_04IINX', 'PC_001GNR', 'PC_0384LZ', 'PC_00772B', 'PC_05TFVL', 'PC_01GNRM', 'PC_042YRE', 'PC_07TUNO', 'PC_0091OE', 'PC_06DDSH', 'PC_002I4B', 'PC_05QB64', 'PC_00LBZX', 'PC_04DUFN', 'PC_08I1Q6', 'PC_0843Z9', 'PC_07QLAB', 'PC_05VZ2W', 'PC_04E4JE', 'PC_039ZP9', 'PC_05MNXH', 'PC_07VHB5', 'PC_05KG1N', 'PC_06187I', 'PC_06RYNI', 'PC_02LGPA', 'PC_02CEZD', 'PC_074DQF', 'PC_03ALOO', 'PC_063JSC', 'PC_0490I5', 'PC_05OR8I', 'PC_05FXKZ', 'PC_07TV4U', 'PC_01D3OM', 'PC_06280D', 'PC_06BL39', 'PC_04SK9M', 'PC_01WPL7', 'PC_088DQF', 'PC_04KM1I', 'PC_06WVB2', 'PC_07TUPN', 'PC_04VBNR', 'PC_07A5YP', 'PC_01Y0R1', 'PC_016FRE', 'PC_05RVL4', 'PC_057FE7', 'PC_05JFQY', 'PC_04PM5Y', 'PC_00HTJV', 'PC_02QIDH', 'PC_046RD9', 'PC_03E0CS', 'PC_00HGKK', 'PC_03813O', 'PC_01KTUJ', 'PC_0860OA', 'PC_04UWXG', 'PC_04PSFQ', 'PC_02KYVT', 'PC_045HU3', 'PC_00UWAQ', 'PC_02BG1Z', 'PC_008806', 'PC_0278ZT', 'PC_07TV02', 'PC_01R1KR', 'PC_01BZCN', 'PC_05W6XP', 'PC_073XSY', 'PC_04HZ79', 'PC_08H11O', 'PC_02QW19', 'PC_01EAHK', 'PC_02UBXP', 'PC_02DAU3', 'PC_021V0R', 'PC_07TVF3', 'PC_04ULOH', 'PC_051C8B', 'PC_00S0UG', 'PC_016TKH', 'PC_07TVA5', 'PC_03PBKY', 'PC_06SE34', 'PC_02K5A5', 'PC_07XIBT', 'PC_06ZU26', 'PC_06FIXN', 'PC_00595C', 'PC_05KEYA', 'PC_0804W2', 'PC_021SYL', 'PC_06K136', 'PC_06WRVL', 'PC_06UZ3U', 'PC_07TVM3', 'PC_05DIQW', 'PC_021EBE', 'PC_00SR1W', 'PC_00UFF9', 'PC_06K75W', 'PC_05N2GV', 'PC_04MR4A', 'PC_02G1YM', 'PC_01QQ1T', 'PC_05TZZ7', 'PC_05W661', 'PC_07D4R9', 'PC_02VB7G', 'PC_02QUA0', 'PC_00VTNG', 'PC_03K305', 'PC_05MI4Z', 'PC_018DM6', 'PC_031XPV', 'PC_036385', 'PC_011LAW', 'PC_04OIFF', 'PC_07UMX8', 'PC_030ACC', 'PC_04SFDZ', 'PC_00YPBK', 'PC_04PJ1H', 'PC_026AIN', 'PC_03792B', 'PC_07AGJQ', 'PC_045E9O', 'PC_057VS9', 'PC_01EZOK', 'PC_032XC4', 'PC_07NWKC', 'PC_06CM2C', 'PC_0654DH', 'PC_07UIKR', 'PC_072DWQ', 'PC_04FB23', 'PC_00NN46', 'PC_04R5US', 'PC_03PEL5', 'PC_06SL6B', 'PC_05L7US', 'PC_05AVFK', 'PC_03HGF9', 'PC_02GYSI', 'PC_01YHQS', 'PC_00915C', 'PC_01J1VJ', 'PC_004U8P', 'PC_060UD5', 'PC_00646F', 'PC_00DU1R', 'PC_05D714', 'PC_0109ZP', 'PC_048XFW', 'PC_05XT7X'}
MSA failed
Edge PC_05JUMB PC_07UIKR 0.07829568535089493
MSA failed
Edge PC_06CAC0 PC_08I3IM 0.07357081025838852
MSA failed
Edge PC_03GTZD PC_04SK9M 0.11814968287944794
lkey 306
lkey_clusters {'PC_03GTZD', 'PC_04L2GD', 'PC_0519TF', 'PC_024FEH', 'PC_02RGUO', 'PC_02NATZ', 'PC_05X98I', 'PC_07TUWD', 'PC_02YR8I', 'PC_041GAL', 'PC_02Q6QH', 'PC_012M5T', 'PC_029YIZ', 'PC_00QDYY', 'PC_01FFWH', 'PC_03FKVQ', 'PC_00D5EQ', 'PC_06PIV6', 'PC_003XGF', 'PC_00QNZJ', 'PC_0287UU', 'PC_01OCWE', 'PC_064DUW'}
MSA failed
Edge PC_03TCZP PC_05TX0J 0.11416839808225632
MSA failed
there are 4 alignments. Each with 111, 100, 36, 6 sequences.
```

In [145]:

```
rnase_set1 = set(rnase_df.loc[rnase_df["cluster"].isin({'PC_03GU7O', 'PC_00QJR6', 'PC_07088H', 'PC_02NISQ', 'PC_03G9RN', 'PC_05PFLL', 'PC_08K2VM', 'PC_03KYEF'}), "supercluster_markov"])
rnase_set1
```

Out[145]:

```
{'PC_03G9RN', 'PC_07088H'}
```

In [146]:

```
rnase_set2 = set(rnase_df.loc[rnase_df["cluster"].isin({'PC_02RGUO', 'PC_06PIV6', 'PC_01OCWE', 'PC_07TUWD', 'PC_041GAL', 'PC_012M5T', 'PC_003XGF', 'PC_00D5EQ', 'PC_064DUW', 'PC_03FKVQ', 'PC_00QNZJ', 'PC_0519TF', 'PC_02YR8I', 'PC_029YIZ', 'PC_02Q6QH', 'PC_04L2GD', 'PC_03GTZD', 'PC_01FFWH', 'PC_05X98I', 'PC_024FEH', 'PC_00QDYY', 'PC_0287UU', 'PC_02NATZ'}), "supercluster_markov"])
rnase_set2
```

Out[146]:

```
{'PC_00D5EQ', 'PC_02Q6QH'}
```

In [147]:

```
rnase_set3 = set(rnase_df.loc[rnase_df["cluster"].isin({'PC_039ZP9', 'PC_06SL6B', 'PC_030ACC', 'PC_01FJ7Z', 'PC_03NC9U', 'PC_072EU7', 'PC_05W661', 'PC_02JQ3K', 'PC_032XC4', 'PC_07XIBT', 'PC_0109ZP', 'PC_03QNGV', 'PC_07A5YP', 'PC_02LGPA', 'PC_07OCLR', 'PC_0654DH', 'PC_06RYNI', 'PC_016FRE', 'PC_05KG1N', 'PC_05K9FR', 'PC_07TV02', 'PC_08I3IM', 'PC_00UFF9', 'PC_02UBXP', 'PC_059TG9', 'PC_00A54M', 'PC_05LDC6', 'PC_02QMV0', 'PC_048XFW', 'PC_07TV8B', 'PC_06UZ06', 'PC_06HSND', 'PC_03PEL5', 'PC_06BL39', 'PC_0278ZT', 'PC_002I4B', 'PC_00SR1W', 'PC_01IC1B', 'PC_009LO1', 'PC_02W8LT', 'PC_022TVQ', 'PC_057FE7', 'PC_023QTD', 'PC_016TKH', 'PC_02BNS6', 'PC_057VS9', 'PC_0100UP', 'PC_00P0Q4', 'PC_01J1VJ', 'PC_00915C', 'PC_06DDSH', 'PC_00ZQQF', 'PC_027RDL', 'PC_0708R1', 'PC_05PJ7O', 'PC_080PLJ', 'PC_07TV9U', 'PC_01KP4T', 'PC_013NPU', 'PC_05N2GV', 'PC_02BG1Z', 'PC_022NID', 'PC_01FQRU', 'PC_07TUNO', 'PC_00S0UG', 'PC_0860OA', 'PC_029RUP', 'PC_0306GU', 'PC_01KTUJ', 'PC_04N53M', 'PC_07D4R9', 'PC_01YHQS', 'PC_011LAW', 'PC_03AMPS', 'PC_045HU3', 'PC_011686', 'PC_00HGKK', 'PC_02CEZD', 'PC_051C8B', 'PC_02D1GK', 'PC_04THYL', 'PC_07AWUL', 'PC_07UMX8', 'PC_0753N9', 'PC_03970R', 'PC_07AGJQ', 'PC_07NWKC', 'PC_05FXKZ', 'PC_046RD9', 'PC_01KBB8', 'PC_04KM1I', 'PC_021V0R', 'PC_05AJOI', 'PC_04PJ1H', 'PC_04HZ79', 'PC_02V4AV', 'PC_042YRE', 'PC_01QQ1T', 'PC_005N2L', 'PC_05XT7X', 'PC_04MR4A', 'PC_05ILWE', 'PC_06SE34', 'PC_00NE3P', 'PC_05QB64', 'PC_062QHL', 'PC_06CM2C', 'PC_05L7US', 'PC_024L0V', 'PC_0164QS', 'PC_00M7NJ', 'PC_03870X', 'PC_0837FY', 'PC_008806', 'PC_0294CS', 'PC_07TVM3', 'PC_0181PE', 'PC_05TX0J', 'PC_0393P5', 'PC_07TVA5', 'PC_05AOUJ', 'PC_0473PG', 'PC_04USMD', 'PC_04TBPN', 'PC_00HTJV', 'PC_02QUA0', 'PC_04OIFF', 'PC_02QIDH', 'PC_08H11O', 'PC_06AI9C', 'PC_030G1S', 'PC_07VJ0Q', 'PC_060UD5', 'PC_03K305', 'PC_01R1KR', 'PC_06BP65', 'PC_05TZZ7', 'PC_0490I5', 'PC_02GYSI', 'PC_01FGFJ', 'PC_021EBE', 'PC_081PV4', 'PC_00NN46', 'PC_02XK62', 'PC_05R7KZ', 'PC_03PSAN', 'PC_02WCO0', 'PC_07TUPN', 'PC_05W6XP', 'PC_021SYL', 'PC_00MO21', 'PC_06MGDC', 'PC_0060XJ', 'PC_06UZ3U', 'PC_04VBNR', 'PC_07A9N7', 'PC_073XSY', 'PC_06ADE4', 'PC_06J307', 'PC_05JFQY', 'PC_05FIVY', 'PC_05KEYA', 'PC_02KEHU', 'PC_031XPV', 'PC_0091OE', 'PC_04DUFN', 'PC_07UIKR', 'PC_03813O', 'PC_04LTCQ', 'PC_04OJOB', 'PC_06K75W', 'PC_00LSXD', 'PC_07WEO1', 'PC_03EHDR', 'PC_063IUN', 'PC_074GF9', 'PC_03E0CS', 'PC_07TVF3', 'PC_02DAU3', 'PC_018GH8', 'PC_02JM6C', 'PC_04V0PU', 'PC_05K4L1', 'PC_02V4LH', 'PC_02AR9H', 'PC_04M1B3', 'PC_04DQ0W', 'PC_01CVKZ', 'PC_03XWEE', 'PC_04D7VO', 'PC_03JFL5', 'PC_00YPBK', 'PC_072DWQ', 'PC_05MVJQ', 'PC_06WRVL', 'PC_03PBKY', 'PC_06LOIO', 'PC_00XN01', 'PC_00A3UY', 'PC_04R5US', 'PC_05TFVL', 'PC_06WVB2', 'PC_001RDT', 'PC_07QLAB', 'PC_05MNXH', 'PC_07076R', 'PC_04PSFQ', 'PC_04SK9M', 'PC_022ZLX', 'PC_00DU1R', 'PC_017F5K', 'PC_053J9J', 'PC_063TMD', 'PC_03ALOO', 'PC_00646F', 'PC_032CKH', 'PC_045E9O', 'PC_08H9YG', 'PC_087UOG', 'PC_01296N', 'PC_04BA1R', 'PC_02HVML', 'PC_04PM5Y', 'PC_004U8P', 'PC_07TV6Z', 'PC_07TV5U', 'PC_06ZU26', 'PC_06IRIY', 'PC_05O6MK', 'PC_01OH6B', 'PC_088DQF', 'PC_01CDCN', 'PC_06OK2P', 'PC_06280D', 'PC_07VHB5', 'PC_01J10M', 'PC_07VAB2', 'PC_047570', 'PC_06K136', 'PC_07TV5W', 'PC_007Z8T', 'PC_02P3RH', 'PC_02EMSJ', 'PC_03T6PW', 'PC_05RVL4', 'PC_02G1YM', 'PC_01EZOK', 'PC_06187I', 'PC_02CC4R', 'PC_01VGHN', 'PC_01Y0R1', 'PC_018DM6', 'PC_02K5A5', 'PC_04IINX', 'PC_002I3M', 'PC_01PMZK', 'PC_00VTNG', 'PC_05DIQW', 'PC_0370DG', 'PC_001GNR', 'PC_02R0M8', 'PC_026AIN', 'PC_04SSH3', 'PC_036385', 'PC_04E4JE', 'PC_02UI5W', 'PC_04U1L7', 'PC_04ULOH', 'PC_0843Z9', 'PC_03AR0I', 'PC_05F9H4', 'PC_04SFDZ', 'PC_06FIXN', 'PC_00UWAQ', 'PC_05VOPA', 'PC_00UAB7', 'PC_063JSC', 'PC_03792B', 'PC_04ZZ0B', 'PC_036HDQ', 'PC_057CGE', 'PC_08I1Q6', 'PC_00772B', 'PC_05D714', 'PC_02OSJL', 'PC_04FB23', 'PC_04UWXG', 'PC_02VB7G', 'PC_009036', 'PC_02KYVT', 'PC_00595C', 'PC_07TUTP', 'PC_02QW19', 'PC_05MHF3', 'PC_073NJA', 'PC_01D3OM', 'PC_01GLB1', 'PC_01BZCN', 'PC_01GNRM', 'PC_00LBZX', 'PC_009BF9', 'PC_060TP7', 'PC_06EWH0', 'PC_02T3ZI', 'PC_01WTO6', 'PC_01W301', 'PC_03HGF9', 'PC_05VZ2W', 'PC_085L07', 'PC_05MI4Z', 'PC_05OR8I', 'PC_02EV3G', 'PC_079ZWS', 'PC_060QAC', 'PC_07TV4U', 'PC_01K875', 'PC_05AVFK', 'PC_0085EC', 'PC_06RYIX', 'PC_04Z3ZQ', 'PC_0384LZ', 'PC_02GRBP', 'PC_02C8S3', 'PC_01EAHK', 'PC_0804W2', 'PC_01WPL7', 'PC_074DQF', 'PC_05RS5N', 'PC_00641O', 'PC_05W9ZE'}), "supercluster_markov"])
len(rnase_set3)
```

Out[147]:

```
62
```

In [148]:

```
rnase_set4 = set(rnase_df.loc[~rnase_df["cluster"].isin({'PC_03GU7O', 'PC_00QJR6', 'PC_07088H', 'PC_02NISQ', 'PC_03G9RN', 'PC_05PFLL', 'PC_08K2VM', 'PC_03KYEF', 'PC_02RGUO', 'PC_06PIV6', 'PC_01OCWE', 'PC_07TUWD', 'PC_041GAL', 'PC_012M5T', 'PC_003XGF', 'PC_00D5EQ', 'PC_064DUW', 'PC_03FKVQ', 'PC_00QNZJ', 'PC_0519TF', 'PC_02YR8I', 'PC_029YIZ', 'PC_02Q6QH', 'PC_04L2GD', 'PC_03GTZD', 'PC_01FFWH', 'PC_05X98I', 'PC_024FEH', 'PC_00QDYY', 'PC_0287UU', 'PC_02NATZ', 'PC_039ZP9', 'PC_06SL6B', 'PC_030ACC', 'PC_01FJ7Z', 'PC_03NC9U', 'PC_072EU7', 'PC_05W661', 'PC_02JQ3K', 'PC_032XC4', 'PC_07XIBT', 'PC_0109ZP', 'PC_03QNGV', 'PC_07A5YP', 'PC_02LGPA', 'PC_07OCLR', 'PC_0654DH', 'PC_06RYNI', 'PC_016FRE', 'PC_05KG1N', 'PC_05K9FR', 'PC_07TV02', 'PC_08I3IM', 'PC_00UFF9', 'PC_02UBXP', 'PC_059TG9', 'PC_00A54M', 'PC_05LDC6', 'PC_02QMV0', 'PC_048XFW', 'PC_07TV8B', 'PC_06UZ06', 'PC_06HSND', 'PC_03PEL5', 'PC_06BL39', 'PC_0278ZT', 'PC_002I4B', 'PC_00SR1W', 'PC_01IC1B', 'PC_009LO1', 'PC_02W8LT', 'PC_022TVQ', 'PC_057FE7', 'PC_023QTD', 'PC_016TKH', 'PC_02BNS6', 'PC_057VS9', 'PC_0100UP', 'PC_00P0Q4', 'PC_01J1VJ', 'PC_00915C', 'PC_06DDSH', 'PC_00ZQQF', 'PC_027RDL', 'PC_0708R1', 'PC_05PJ7O', 'PC_080PLJ', 'PC_07TV9U', 'PC_01KP4T', 'PC_013NPU', 'PC_05N2GV', 'PC_02BG1Z', 'PC_022NID', 'PC_01FQRU', 'PC_07TUNO', 'PC_00S0UG', 'PC_0860OA', 'PC_029RUP', 'PC_0306GU', 'PC_01KTUJ', 'PC_04N53M', 'PC_07D4R9', 'PC_01YHQS', 'PC_011LAW', 'PC_03AMPS', 'PC_045HU3', 'PC_011686', 'PC_00HGKK', 'PC_02CEZD', 'PC_051C8B', 'PC_02D1GK', 'PC_04THYL', 'PC_07AWUL', 'PC_07UMX8', 'PC_0753N9', 'PC_03970R', 'PC_07AGJQ', 'PC_07NWKC', 'PC_05FXKZ', 'PC_046RD9', 'PC_01KBB8', 'PC_04KM1I', 'PC_021V0R', 'PC_05AJOI', 'PC_04PJ1H', 'PC_04HZ79', 'PC_02V4AV', 'PC_042YRE', 'PC_01QQ1T', 'PC_005N2L', 'PC_05XT7X', 'PC_04MR4A', 'PC_05ILWE', 'PC_06SE34', 'PC_00NE3P', 'PC_05QB64', 'PC_062QHL', 'PC_06CM2C', 'PC_05L7US', 'PC_024L0V', 'PC_0164QS', 'PC_00M7NJ', 'PC_03870X', 'PC_0837FY', 'PC_008806', 'PC_0294CS', 'PC_07TVM3', 'PC_0181PE', 'PC_05TX0J', 'PC_0393P5', 'PC_07TVA5', 'PC_05AOUJ', 'PC_0473PG', 'PC_04USMD', 'PC_04TBPN', 'PC_00HTJV', 'PC_02QUA0', 'PC_04OIFF', 'PC_02QIDH', 'PC_08H11O', 'PC_06AI9C', 'PC_030G1S', 'PC_07VJ0Q', 'PC_060UD5', 'PC_03K305', 'PC_01R1KR', 'PC_06BP65', 'PC_05TZZ7', 'PC_0490I5', 'PC_02GYSI', 'PC_01FGFJ', 'PC_021EBE', 'PC_081PV4', 'PC_00NN46', 'PC_02XK62', 'PC_05R7KZ', 'PC_03PSAN', 'PC_02WCO0', 'PC_07TUPN', 'PC_05W6XP', 'PC_021SYL', 'PC_00MO21', 'PC_06MGDC', 'PC_0060XJ', 'PC_06UZ3U', 'PC_04VBNR', 'PC_07A9N7', 'PC_073XSY', 'PC_06ADE4', 'PC_06J307', 'PC_05JFQY', 'PC_05FIVY', 'PC_05KEYA', 'PC_02KEHU', 'PC_031XPV', 'PC_0091OE', 'PC_04DUFN', 'PC_07UIKR', 'PC_03813O', 'PC_04LTCQ', 'PC_04OJOB', 'PC_06K75W', 'PC_00LSXD', 'PC_07WEO1', 'PC_03EHDR', 'PC_063IUN', 'PC_074GF9', 'PC_03E0CS', 'PC_07TVF3', 'PC_02DAU3', 'PC_018GH8', 'PC_02JM6C', 'PC_04V0PU', 'PC_05K4L1', 'PC_02V4LH', 'PC_02AR9H', 'PC_04M1B3', 'PC_04DQ0W', 'PC_01CVKZ', 'PC_03XWEE', 'PC_04D7VO', 'PC_03JFL5', 'PC_00YPBK', 'PC_072DWQ', 'PC_05MVJQ', 'PC_06WRVL', 'PC_03PBKY', 'PC_06LOIO', 'PC_00XN01', 'PC_00A3UY', 'PC_04R5US', 'PC_05TFVL', 'PC_06WVB2', 'PC_001RDT', 'PC_07QLAB', 'PC_05MNXH', 'PC_07076R', 'PC_04PSFQ', 'PC_04SK9M', 'PC_022ZLX', 'PC_00DU1R', 'PC_017F5K', 'PC_053J9J', 'PC_063TMD', 'PC_03ALOO', 'PC_00646F', 'PC_032CKH', 'PC_045E9O', 'PC_08H9YG', 'PC_087UOG', 'PC_01296N', 'PC_04BA1R', 'PC_02HVML', 'PC_04PM5Y', 'PC_004U8P', 'PC_07TV6Z', 'PC_07TV5U', 'PC_06ZU26', 'PC_06IRIY', 'PC_05O6MK', 'PC_01OH6B', 'PC_088DQF', 'PC_01CDCN', 'PC_06OK2P', 'PC_06280D', 'PC_07VHB5', 'PC_01J10M', 'PC_07VAB2', 'PC_047570', 'PC_06K136', 'PC_07TV5W', 'PC_007Z8T', 'PC_02P3RH', 'PC_02EMSJ', 'PC_03T6PW', 'PC_05RVL4', 'PC_02G1YM', 'PC_01EZOK', 'PC_06187I', 'PC_02CC4R', 'PC_01VGHN', 'PC_01Y0R1', 'PC_018DM6', 'PC_02K5A5', 'PC_04IINX', 'PC_002I3M', 'PC_01PMZK', 'PC_00VTNG', 'PC_05DIQW', 'PC_0370DG', 'PC_001GNR', 'PC_02R0M8', 'PC_026AIN', 'PC_04SSH3', 'PC_036385', 'PC_04E4JE', 'PC_02UI5W', 'PC_04U1L7', 'PC_04ULOH', 'PC_0843Z9', 'PC_03AR0I', 'PC_05F9H4', 'PC_04SFDZ', 'PC_06FIXN', 'PC_00UWAQ', 'PC_05VOPA', 'PC_00UAB7', 'PC_063JSC', 'PC_03792B', 'PC_04ZZ0B', 'PC_036HDQ', 'PC_057CGE', 'PC_08I1Q6', 'PC_00772B', 'PC_05D714', 'PC_02OSJL', 'PC_04FB23', 'PC_04UWXG', 'PC_02VB7G', 'PC_009036', 'PC_02KYVT', 'PC_00595C', 'PC_07TUTP', 'PC_02QW19', 'PC_05MHF3', 'PC_073NJA', 'PC_01D3OM', 'PC_01GLB1', 'PC_01BZCN', 'PC_01GNRM', 'PC_00LBZX', 'PC_009BF9', 'PC_060TP7', 'PC_06EWH0', 'PC_02T3ZI', 'PC_01WTO6', 'PC_01W301', 'PC_03HGF9', 'PC_05VZ2W', 'PC_085L07', 'PC_05MI4Z', 'PC_05OR8I', 'PC_02EV3G', 'PC_079ZWS', 'PC_060QAC', 'PC_07TV4U', 'PC_01K875', 'PC_05AVFK', 'PC_0085EC', 'PC_06RYIX', 'PC_04Z3ZQ', 'PC_0384LZ', 'PC_02GRBP', 'PC_02C8S3', 'PC_01EAHK', 'PC_0804W2', 'PC_01WPL7', 'PC_074DQF', 'PC_05RS5N', 'PC_00641O', 'PC_05W9ZE'}), "supercluster_markov"])
rnase_set4
```

Out[148]:

```
{'PC_01NR73',
 'PC_02WPLI',
 'PC_03TCZP',
 'PC_05JUMB',
 'PC_06BZLT',
 'PC_06MGDC',
 'PC_07AJXF',
 'PC_08I3IM'}
```

In [149]:

```
rnase_cluster_ids = []

for i, cluster in enumerate(rnase_prog_msas):
    this = []
    seqs = SeqIO.parse(StringIO(cluster.decode()), "fasta")
    for seq in seqs:
        if seq.id.startswith("PC_") and seq.id.endswith("_consensus"):
            this.append(seq.id[:-len("_consensus")])
        elif seq.id.startswith("PC_"):
            this.append(seq.id)
    rnase_cluster_ids.append(this)
    
    this_comps = rnase_df.loc[rnase_df["member"].isin(set(this)), "supercluster_component"].unique()
    this_markov = rnase_df.loc[rnase_df["member"].isin(set(this)), "supercluster_markov"].unique()
    this_greedy = rnase_df.loc[rnase_df["member"].isin(set(this)), "supercluster_greedy"].unique()
    this_clusters = rnase_df.loc[rnase_df["member"].isin(set(this)), "cluster"].unique()
    this_effectors = rnase_df.loc[rnase_df["member"].isin(set(this)), "effectors"].unique()
    
    
    print("Cluster", i, "components:", this_comps, "markov:", this_markov, "greedy:", this_greedy, "cluster:", repr(this_clusters), "effectors:", this_effectors)
```

```
Cluster 0 components: ['PC_032CKH'] markov: ['PC_00D5EQ' 'PC_02Q6QH'] greedy: ['PC_01FFWH' 'PC_02YR8I' 'PC_06PIV6' 'PC_041GAL'] cluster: array(['PC_01FFWH', 'PC_02YR8I', 'PC_003XGF', 'PC_00D5EQ', 'PC_00QDYY',
       'PC_00QNZJ', 'PC_012M5T', 'PC_01OCWE', 'PC_0287UU', 'PC_03GTZD',
       'PC_0519TF', 'PC_06PIV6', 'PC_07TUWD', 'PC_02NATZ', 'PC_02RGUO',
       'PC_041GAL', 'PC_064DUW', 'PC_024FEH', 'PC_02Q6QH'], dtype=object) effectors: [nan]
Cluster 1 components: ['PC_032CKH'] markov: ['PC_01NR73' 'PC_02WPLI' 'PC_03G9RN' 'PC_03TCZP' 'PC_05JUMB' 'PC_06BZLT'
 'PC_07088H' 'PC_08I3IM'] greedy: ['PC_01NR73' 'PC_02WPLI' 'PC_03G9RN' 'PC_03TCZP' 'PC_05JUMB' 'PC_06BZLT'
 'PC_00QJR6' 'PC_03KYEF' 'PC_08I3IM'] cluster: array(['PC_01NR73', 'PC_02WPLI', 'PC_02NISQ', 'PC_03G9RN', 'PC_05PFLL',
       'PC_03TCZP', 'PC_05JUMB', 'PC_06BZLT', 'PC_00QJR6', 'PC_08K2VM',
       'PC_07088H', 'PC_03KYEF', 'PC_06CAC0'], dtype=object) effectors: [nan]
Cluster 2 components: ['PC_032CKH'] markov: ['PC_00XN01' 'PC_02BG1Z' 'PC_03ALOO'] greedy: ['PC_00XN01' 'PC_02BG1Z' 'PC_03ALOO'] cluster: array(['PC_00XN01', 'PC_07A5YP', 'PC_03ALOO'], dtype=object) effectors: [nan]
Cluster 3 components: ['PC_032CKH'] markov: ['PC_06MGDC'] greedy: ['PC_06MGDC'] cluster: array(['PC_0574V4'], dtype=object) effectors: [nan]
```

In [150]:

```
rnase_set3.intersection(rnase_set4)
```

Out[150]:

```
{'PC_06MGDC', 'PC_08I3IM'}
```

In [151]:

```
rnase_set1_df = eff_with_unique[
    eff_with_unique["supercluster_markov"].isin(rnase_set1)
]

rnase_set1_hmms, rnase_set1_msas, rnase_set1_unfiltered_dfs, rnase_set1_filtered_dfs, rnase_set1_markov_cmap, rnase_set1_greedy_cmap = runit(
    "rnase_set1",
    G,
    rnase_set1_df,
    enriched,
    unenriched,
)
```

```
markov
```

```
greedy
```

```
markov mst
```

```
greedy mst
```

```
there are 1 alignments. Each with 91 sequences.
# hmmbuild :: profile HMM construction from multiple sequence alignments
# HMMER 3.3 (Nov 2019); http://hmmer.org/
# Copyright (C) 2019 Howard Hughes Medical Institute.
# Freely distributed under the BSD open source license.
# - - - - - - - - - - - - - - - - - - - - - - - - - - - - - - - - - - - -
# input alignment file:             /tmp/tmpz9atmo1_
# output HMM file:                  /tmp/tmp6gi3uvve
# name (the single) HMM:            rnase_set1
# input alignment is asserted as:   protein
# - - - - - - - - - - - - - - - - - - - - - - - - - - - - - - - - - - - -

# idx name                  nseq  alen  mlen eff_nseq re/pos description
#---- -------------------- ----- ----- ----- -------- ------ -----------
1     rnase_set1              91   281   172     3.01  0.590 

# CPU time: 0.08u 0.00s 00:00:00.08 Elapsed: 00:00:00.08
```

In [153]:

```
get_plots(
    "rnase_set1",
    0,
    rnase_set1_hmms,
    rnase_set1_msas,
    rnase_set1_unfiltered_dfs,
    rnase_set1_filtered_dfs,
    rnase_set1_markov_cmap,
    rnase_set1_greedy_cmap,
    run_fastree=True,
    show=True,
)
```

```
# hmmbuild :: profile HMM construction from multiple sequence alignments
# HMMER 3.3 (Nov 2019); http://hmmer.org/
# Copyright (C) 2019 Howard Hughes Medical Institute.
# Freely distributed under the BSD open source license.
# - - - - - - - - - - - - - - - - - - - - - - - - - - - - - - - - - - - -
# input alignment file:             /tmp/tmpvl49y9j8
# output HMM file:                  /tmp/tmpyqbkzhh_
# name (the single) HMM:            rnase_set1
# input alignment is asserted as:   protein
# - - - - - - - - - - - - - - - - - - - - - - - - - - - - - - - - - - - -

# idx name                  nseq  alen  mlen eff_nseq re/pos description
#---- -------------------- ----- ----- ----- -------- ------ -----------
1     rnase_set1              40   371   221     3.69  0.590 

# CPU time: 0.09u 0.01s 00:00:00.10 Elapsed: 00:00:00.09


rnase_set1-unfiltered_logo
```

```
rnase_set1-unfiltered_logo-maxgap0.5
```

```
rnase_set1-filtered_logo
```

```
rnase_set1-filtered_logo-maxgap0.5
```

```
rnase_set1-filtered_logo_group
```

```
rnase_set1-filtered_logo_group-maxgap0.5
```

```
rnase_set1-filtered_logo_group-maxgap0.5minsize10
```

```
rnase_set1-unfiltered_logo_group
```

```
rnase_set1-unfiltered_logo_group-maxgap0.5
```

```
rnase_set1-unfiltered_logo_group-maxgap0.5minsize10
```

```
rnase_set1-unfiltered_markov
```

```
rnase_set1-unfiltered_greedy
```

```
rnase_set1-filtered_markov
```

```
rnase_set1-filtered_greedy
```

```
rnase_set1-unfiltered_markov-maxgap0.5
```

```
rnase_set1-unfiltered_greedy-maxgap0.5
```

```
rnase_set1-filtered_markov-maxgap0.5
```

```
rnase_set1-filtered_greedy-maxgap0.5
```

In [154]:

```
rnase_set2_df = eff_with_unique[
    eff_with_unique["supercluster_markov"].isin(rnase_set2)
]

rnase_set2_hmms, rnase_set2_msas, rnase_set2_unfiltered_dfs, rnase_set2_filtered_dfs, rnase_set2_markov_cmap, rnase_set2_greedy_cmap = runit(
    "rnase_set2",
    G,
    rnase_set2_df,
    enriched,
    unenriched,
)
```

```
markov
```

```
greedy
```

```
markov mst
```

```
greedy mst
```

```
there are 1 alignments. Each with 111 sequences.
# hmmbuild :: profile HMM construction from multiple sequence alignments
# HMMER 3.3 (Nov 2019); http://hmmer.org/
# Copyright (C) 2019 Howard Hughes Medical Institute.
# Freely distributed under the BSD open source license.
# - - - - - - - - - - - - - - - - - - - - - - - - - - - - - - - - - - - -
# input alignment file:             /tmp/tmpihfx3rfn
# output HMM file:                  /tmp/tmpyc222r25
# name (the single) HMM:            rnase_set2
# input alignment is asserted as:   protein
# - - - - - - - - - - - - - - - - - - - - - - - - - - - - - - - - - - - -

# idx name                  nseq  alen  mlen eff_nseq re/pos description
#---- -------------------- ----- ----- ----- -------- ------ -----------
1     rnase_set2             111    94    80     5.28  0.709 

# CPU time: 0.04u 0.00s 00:00:00.04 Elapsed: 00:00:00.04
```

In [155]:

```
get_plots(
    "rnase_set2",
    0,
    rnase_set2_hmms,
    rnase_set2_msas,
    rnase_set2_unfiltered_dfs,
    rnase_set2_filtered_dfs,
    rnase_set2_markov_cmap,
    rnase_set2_greedy_cmap,
    run_fastree=True,
    show=True,
)
```

```
# hmmbuild :: profile HMM construction from multiple sequence alignments
# HMMER 3.3 (Nov 2019); http://hmmer.org/
# Copyright (C) 2019 Howard Hughes Medical Institute.
# Freely distributed under the BSD open source license.
# - - - - - - - - - - - - - - - - - - - - - - - - - - - - - - - - - - - -
# input alignment file:             /tmp/tmpzlqpj4qj
# output HMM file:                  /tmp/tmpg8i50qeb
# name (the single) HMM:            rnase_set2
# input alignment is asserted as:   protein
# - - - - - - - - - - - - - - - - - - - - - - - - - - - - - - - - - - - -

# idx name                  nseq  alen  mlen eff_nseq re/pos description
#---- -------------------- ----- ----- ----- -------- ------ -----------
1     rnase_set2             133   177   113     4.85  0.590 

# CPU time: 0.05u 0.00s 00:00:00.05 Elapsed: 00:00:00.05


rnase_set2-unfiltered_logo
```

```
rnase_set2-unfiltered_logo-maxgap0.5
```

```
rnase_set2-filtered_logo
```

```
rnase_set2-filtered_logo-maxgap0.5
```

```
rnase_set2-filtered_logo_group
```

```
rnase_set2-filtered_logo_group-maxgap0.5
```

```
rnase_set2-filtered_logo_group-maxgap0.5minsize10
```

```
rnase_set2-unfiltered_logo_group
```

```
rnase_set2-unfiltered_logo_group-maxgap0.5
```

```
rnase_set2-unfiltered_logo_group-maxgap0.5minsize10
```

```
rnase_set2-unfiltered_markov
```

```
rnase_set2-unfiltered_greedy
```

```
rnase_set2-filtered_markov
```

```
rnase_set2-filtered_greedy
```

```
rnase_set2-unfiltered_markov-maxgap0.5
```

```
rnase_set2-unfiltered_greedy-maxgap0.5
```

```
rnase_set2-filtered_markov-maxgap0.5
```

```
rnase_set2-filtered_greedy-maxgap0.5
```

In [156]:

```
rnase_set3_df = eff_with_unique[
    eff_with_unique["supercluster_markov"].isin(rnase_set3)
]

rnase_set3_hmms, rnase_set3_msas, rnase_set3_unfiltered_dfs, rnase_set3_filtered_dfs, rnase_set3_markov_cmap, rnase_set3_greedy_cmap = runit(
    "rnase_set3",
    G,
    rnase_set3_df,
    enriched,
    unenriched,
)
```

```
markov
```

```
greedy
```

```
markov mst
```

```
greedy mst
```

```
there are 3 alignments. Each with 36, 6, 2 sequences.
# hmmbuild :: profile HMM construction from multiple sequence alignments
# HMMER 3.3 (Nov 2019); http://hmmer.org/
# Copyright (C) 2019 Howard Hughes Medical Institute.
# Freely distributed under the BSD open source license.
# - - - - - - - - - - - - - - - - - - - - - - - - - - - - - - - - - - - -
# input alignment file:             /tmp/tmpjno0ih9i
# output HMM file:                  /tmp/tmpkt4yldxl
# name (the single) HMM:            rnase_set3
# input alignment is asserted as:   protein
# - - - - - - - - - - - - - - - - - - - - - - - - - - - - - - - - - - - -

# idx name                  nseq  alen  mlen eff_nseq re/pos description
#---- -------------------- ----- ----- ----- -------- ------ -----------
1     rnase_set3              36   121   101     1.74  0.591 

# CPU time: 0.04u 0.01s 00:00:00.05 Elapsed: 00:00:00.04


# hmmbuild :: profile HMM construction from multiple sequence alignments
# HMMER 3.3 (Nov 2019); http://hmmer.org/
# Copyright (C) 2019 Howard Hughes Medical Institute.
# Freely distributed under the BSD open source license.
# - - - - - - - - - - - - - - - - - - - - - - - - - - - - - - - - - - - -
# input alignment file:             /tmp/tmptr9xiods
# output HMM file:                  /tmp/tmpkpveo4wn
# name (the single) HMM:            rnase_set3
# input alignment is asserted as:   protein
# - - - - - - - - - - - - - - - - - - - - - - - - - - - - - - - - - - - -

# idx name                  nseq  alen  mlen eff_nseq re/pos description
#---- -------------------- ----- ----- ----- -------- ------ -----------
1     rnase_set3               6   171   169     0.57  0.590 

# CPU time: 0.06u 0.00s 00:00:00.06 Elapsed: 00:00:00.06


# hmmbuild :: profile HMM construction from multiple sequence alignments
# HMMER 3.3 (Nov 2019); http://hmmer.org/
# Copyright (C) 2019 Howard Hughes Medical Institute.
# Freely distributed under the BSD open source license.
# - - - - - - - - - - - - - - - - - - - - - - - - - - - - - - - - - - - -
# input alignment file:             /tmp/tmp48qsy3ye
# output HMM file:                  /tmp/tmp3_e6v8h1
# name (the single) HMM:            rnase_set3
# input alignment is asserted as:   protein
# - - - - - - - - - - - - - - - - - - - - - - - - - - - - - - - - - - - -

# idx name                  nseq  alen  mlen eff_nseq re/pos description
#---- -------------------- ----- ----- ----- -------- ------ -----------
1     rnase_set3               2   183   183     0.38  0.585 

# CPU time: 0.06u 0.00s 00:00:00.06 Elapsed: 00:00:00.06
```

In [157]:

```
get_plots(
    "rnase_set3",
    0,
    rnase_set3_hmms,
    rnase_set3_msas,
    rnase_set3_unfiltered_dfs,
    rnase_set3_filtered_dfs,
    rnase_set3_markov_cmap,
    rnase_set3_greedy_cmap,
    run_fastree=True,
    show=True,
)
```

```
# hmmbuild :: profile HMM construction from multiple sequence alignments
# HMMER 3.3 (Nov 2019); http://hmmer.org/
# Copyright (C) 2019 Howard Hughes Medical Institute.
# Freely distributed under the BSD open source license.
# - - - - - - - - - - - - - - - - - - - - - - - - - - - - - - - - - - - -
# input alignment file:             /tmp/tmpt4k09jxx
# output HMM file:                  /tmp/tmp4hb9e2g2
# name (the single) HMM:            rnase_set3
# input alignment is asserted as:   protein
# - - - - - - - - - - - - - - - - - - - - - - - - - - - - - - - - - - - -

# idx name                  nseq  alen  mlen eff_nseq re/pos description
#---- -------------------- ----- ----- ----- -------- ------ -----------
1     rnase_set3            1365  2331  1100     8.43  0.590 

# CPU time: 0.68u 0.01s 00:00:00.69 Elapsed: 00:00:00.69


rnase_set3-unfiltered_logo
 Warning: Character 'Z' is not in color_dict. Using black.
```

```
---------------------------------------------------------------------------
ValueError                                Traceback (most recent call last)
<ipython-input-157-aedb58f90023> in <module>
      9     rnase_set3_greedy_cmap,
     10     run_fastree=True,
---> 11     show=True,
     12 )

<ipython-input-45-faf363bbac27> in get_plots(name, index, hmms, msas, unfiltered_dfs, filtered_dfs, markov_cmap, greedy_cmap, outdir, palette, logo_palette, run_fastree, show)
     42     n = f"{name}-unfiltered_logo"
     43     print(n)
---> 44     l = plot_logo(unfiltered_df, n, show=show, outdir=outdir)
     45     n = f"{name}-unfiltered_logo-maxgap0.5"
     46     print(n)

<ipython-input-42-e188fa8e6c88> in plot_logo(df, name, max_gap, outdir, base_width, width_per_res, height, alignment_col, font, logo_palette, show)
     38 
     39     fig.savefig(os.path.join(outdir, f"{name}-total_logo.svg"))
---> 40     fig.savefig(os.path.join(outdir, f"{name}-total_logo.png"), dpi=600)
     41 
     42     if show:

~/.miniconda/envs/remote_homology/lib/python3.7/site-packages/matplotlib/figure.py in savefig(self, fname, transparent, **kwargs)
   2201             self.patch.set_visible(frameon)
   2202 
-> 2203         self.canvas.print_figure(fname, **kwargs)
   2204 
   2205         if frameon:

~/.miniconda/envs/remote_homology/lib/python3.7/site-packages/matplotlib/backend_bases.py in print_figure(self, filename, dpi, facecolor, edgecolor, orientation, format, bbox_inches, **kwargs)
   2124                     orientation=orientation,
   2125                     bbox_inches_restore=_bbox_inches_restore,
-> 2126                     **kwargs)
   2127             finally:
   2128                 if bbox_inches and restore_bbox:

~/.miniconda/envs/remote_homology/lib/python3.7/site-packages/matplotlib/backends/backend_agg.py in print_png(self, filename_or_obj, metadata, pil_kwargs, *args, **kwargs)
    512         }
    513 
--> 514         FigureCanvasAgg.draw(self)
    515         if pil_kwargs is not None:
    516             from PIL import Image

~/.miniconda/envs/remote_homology/lib/python3.7/site-packages/matplotlib/backends/backend_agg.py in draw(self)
    386         Draw the figure using the renderer.
    387         """
--> 388         self.renderer = self.get_renderer(cleared=True)
    389         # Acquire a lock on the shared font cache.
    390         with RendererAgg.lock, \

~/.miniconda/envs/remote_homology/lib/python3.7/site-packages/matplotlib/backends/backend_agg.py in get_renderer(self, cleared)
    402                           and getattr(self, "_lastKey", None) == key)
    403         if not reuse_renderer:
--> 404             self.renderer = RendererAgg(w, h, self.figure.dpi)
    405             self._lastKey = key
    406         elif cleared:

~/.miniconda/envs/remote_homology/lib/python3.7/site-packages/matplotlib/backends/backend_agg.py in __init__(self, width, height, dpi)
     90         self.width = width
     91         self.height = height
---> 92         self._renderer = _RendererAgg(int(width), int(height), dpi)
     93         self._filter_renderers = []
     94 

ValueError: Image size of 153360x1500 pixels is too large. It must be less than 2^16 in each direction.
```

This contains our effectors but it's too big, and I can already tell it won't be a great alignment (1100 match columns!?).

In [158]:

```
rnase_set4_df = eff_with_unique[
    eff_with_unique["supercluster_markov"].isin(rnase_set4)
]

rnase_set4_hmms, rnase_set4_msas, rnase_set4_unfiltered_dfs, rnase_set4_filtered_dfs, rnase_set4_markov_cmap, rnase_set4_greedy_cmap = runit(
    "rnase_set4",
    G,
    rnase_set4_df,
    enriched,
    unenriched,
)
```

```
markov
```

```
greedy
```

```
markov mst
```

```
greedy mst
```

```
there are 1 alignments. Each with 11 sequences.
# hmmbuild :: profile HMM construction from multiple sequence alignments
# HMMER 3.3 (Nov 2019); http://hmmer.org/
# Copyright (C) 2019 Howard Hughes Medical Institute.
# Freely distributed under the BSD open source license.
# - - - - - - - - - - - - - - - - - - - - - - - - - - - - - - - - - - - -
# input alignment file:             /tmp/tmpxp2n4cei
# output HMM file:                  /tmp/tmpy3ir1zld
# name (the single) HMM:            rnase_set4
# input alignment is asserted as:   protein
# - - - - - - - - - - - - - - - - - - - - - - - - - - - - - - - - - - - -

# idx name                  nseq  alen  mlen eff_nseq re/pos description
#---- -------------------- ----- ----- ----- -------- ------ -----------
1     rnase_set4              11   211   173     1.53  0.590 

# CPU time: 0.06u 0.00s 00:00:00.06 Elapsed: 00:00:00.07
```

In [159]:

```
get_plots(
    "rnase_set4",
    0,
    rnase_set4_hmms,
    rnase_set4_msas,
    rnase_set4_unfiltered_dfs,
    rnase_set4_filtered_dfs,
    rnase_set4_markov_cmap,
    rnase_set4_greedy_cmap,
    run_fastree=True,
    show=True,
)
```

```
# hmmbuild :: profile HMM construction from multiple sequence alignments
# HMMER 3.3 (Nov 2019); http://hmmer.org/
# Copyright (C) 2019 Howard Hughes Medical Institute.
# Freely distributed under the BSD open source license.
# - - - - - - - - - - - - - - - - - - - - - - - - - - - - - - - - - - - -
# input alignment file:             /tmp/tmp068mbyuu
# output HMM file:                  /tmp/tmpgtz0brqk
# name (the single) HMM:            rnase_set4
# input alignment is asserted as:   protein
# - - - - - - - - - - - - - - - - - - - - - - - - - - - - - - - - - - - -

# idx name                  nseq  alen  mlen eff_nseq re/pos description
#---- -------------------- ----- ----- ----- -------- ------ -----------
1     rnase_set4              20   376   234     3.83  0.590 

# CPU time: 0.09u 0.00s 00:00:00.09 Elapsed: 00:00:00.09


rnase_set4-unfiltered_logo
```

```
rnase_set4-unfiltered_logo-maxgap0.5
```

```
rnase_set4-filtered_logo
```

```
rnase_set4-filtered_logo-maxgap0.5
```

```
rnase_set4-filtered_logo_group
```

```
rnase_set4-filtered_logo_group-maxgap0.5
```

```
rnase_set4-filtered_logo_group-maxgap0.5minsize10
```

```
---------------------------------------------------------------------------
LogomakerError                            Traceback (most recent call last)
<ipython-input-159-9c474f6bdf1f> in <module>
      9     rnase_set4_greedy_cmap,
     10     run_fastree=True,
---> 11     show=True,
     12 )

<ipython-input-45-faf363bbac27> in get_plots(name, index, hmms, msas, unfiltered_dfs, filtered_dfs, markov_cmap, greedy_cmap, outdir, palette, logo_palette, run_fastree, show)
     64     print(n)
     65     l = plot_logo_group(filtered_df, n,
---> 66                         markov_cmap, max_gap=0.5, min_size=10, show=show, outdir=outdir)
     67 
     68 

<ipython-input-134-7f485e5fc5a3> in plot_logo_group(df, name, cluster_cmap, row_order, min_size, max_gap, outdir, base_width, width_per_res, height, supercluster_col, alignment_col, sharey, show, logo_palette)
     35             characters_to_ignore=".-X",
     36             to_type="counts",
---> 37             pseudocount=0
     38         )
     39         filter_ = (1 - (mat_counts.sum(axis=1) / len(df[alignment_col]))) <= max_gap

~/.miniconda/envs/remote_homology/lib/python3.7/site-packages/logomaker/src/error_handling.py in wrapped_func(*args, **kwargs)
    132             # Otherwise, raise error
    133             else:
--> 134                 raise e
    135 
    136         # If not in debug mode, and no error was detected above,

~/.miniconda/envs/remote_homology/lib/python3.7/site-packages/logomaker/src/error_handling.py in wrapped_func(*args, **kwargs)
    100         try:
    101             # Execute function
--> 102             result = func(*args, **kwargs)
    103 
    104             # If running functional test and expect to fail

~/.miniconda/envs/remote_homology/lib/python3.7/site-packages/logomaker/src/matrix.py in alignment_to_matrix(sequences, counts, to_type, background, characters_to_ignore, center_weights, pseudocount)
    519 
    520     # Make sure sequences has at least 1 element
--> 521     check(len(sequences) > 0, 'sequences must have length > 0.')
    522 
    523     # Make sure all elements are sequences

~/.miniconda/envs/remote_homology/lib/python3.7/site-packages/logomaker/src/error_handling.py in check(condition, message)
     54 
     55     if not condition:
---> 56         raise LogomakerError(message)
     57 
     58 

LogomakerError: sequences must have length > 0.
```

In [ ]:

In [161]:

```
rnase_effectors_markov = set(eff_with_unique.loc[eff_with_unique["effectors"].notnull(), "supercluster_markov"])
rnase_effectors_greedy = set(eff_with_unique.loc[eff_with_unique["effectors"].notnull(), "supercluster_greedy"])
rnase_effectors_df = eff_with_unique[
    (eff_with_unique["supercluster_component"] == "PC_032CKH") & \
    (
        eff_with_unique["supercluster_markov"].isin(rnase_effectors_markov) | \
         eff_with_unique["supercluster_greedy"].isin(rnase_effectors_greedy)
    )
]
rnase_effectors_df.head()
```

Out[161]:

|  | supercluster\_component | supercluster\_markov | supercluster\_greedy | cluster | effectors | phibase | organisms | lca\_rank | lca | cluster\_size | cluster\_length | member |
| --- | --- | --- | --- | --- | --- | --- | --- | --- | --- | --- | --- | --- |
| 181868 | PC\_032CKH | PC\_00595C | PC\_07TVM3 | PC\_00YPBK | NaN | NaN | Blumeria graminis f. sp. hordei DH14;Blumeria ... | species | Blumeria graminis | 8 | 146 | PC\_00YPBK |
| 181869 | PC\_032CKH | PC\_00595C | PC\_07TVM3 | PC\_00YPBK | NaN | NaN | Blumeria graminis f. sp. hordei DH14;Blumeria ... | species | Blumeria graminis | 8 | 146 | PC\_00HQ7Y |
| 181870 | PC\_032CKH | PC\_00595C | PC\_07TVM3 | PC\_00YPBK | NaN | NaN | Blumeria graminis f. sp. hordei DH14;Blumeria ... | species | Blumeria graminis | 8 | 146 | PC\_05K218 |
| 181871 | PC\_032CKH | PC\_00595C | PC\_07TVM3 | PC\_00YPBK | NaN | NaN | Blumeria graminis f. sp. hordei DH14;Blumeria ... | species | Blumeria graminis | 8 | 146 | PC\_010C61 |
| 181872 | PC\_032CKH | PC\_00595C | PC\_07TVM3 | PC\_00YPBK | NaN | NaN | Blumeria graminis f. sp. hordei DH14;Blumeria ... | species | Blumeria graminis | 8 | 146 | PC\_04ASWU |

In [164]:

```
rnase_effectors_hmms, rnase_effectors_msas, rnase_effectors_unfiltered_dfs, rnase_effectors_filtered_dfs, rnase_effectors_markov_cmap, rnase_effectors_greedy_cmap = runit(
    "rnase_effectors",
    G,
    rnase_effectors_df,
    enriched,
    unenriched,
)
```

```
markov
```

```
greedy
```

```
markov mst
```

```
greedy mst
```

```
there are 1 alignments. Each with 153 sequences.
# hmmbuild :: profile HMM construction from multiple sequence alignments
# HMMER 3.3 (Nov 2019); http://hmmer.org/
# Copyright (C) 2019 Howard Hughes Medical Institute.
# Freely distributed under the BSD open source license.
# - - - - - - - - - - - - - - - - - - - - - - - - - - - - - - - - - - - -
# input alignment file:             /tmp/tmp8m1j6q1p
# output HMM file:                  /tmp/tmpog32man9
# name (the single) HMM:            rnase_effectors
# input alignment is asserted as:   protein
# - - - - - - - - - - - - - - - - - - - - - - - - - - - - - - - - - - - -

# idx name                  nseq  alen  mlen eff_nseq re/pos description
#---- -------------------- ----- ----- ----- -------- ------ -----------
1     rnase_effectors        153   210   120    10.44  0.590 

# CPU time: 0.06u 0.00s 00:00:00.06 Elapsed: 00:00:00.06
```

In [165]:

```
get_plots(
    "rnase_effectors",
    0,
    rnase_effectors_hmms,
    rnase_effectors_msas,
    rnase_effectors_unfiltered_dfs,
    rnase_effectors_filtered_dfs,
    rnase_effectors_markov_cmap,
    rnase_effectors_greedy_cmap,
    run_fastree=True,
    show=True,
)
```

```
# hmmbuild :: profile HMM construction from multiple sequence alignments
# HMMER 3.3 (Nov 2019); http://hmmer.org/
# Copyright (C) 2019 Howard Hughes Medical Institute.
# Freely distributed under the BSD open source license.
# - - - - - - - - - - - - - - - - - - - - - - - - - - - - - - - - - - - -
# input alignment file:             /tmp/tmp66duue5h
# output HMM file:                  /tmp/tmp9zrd6bc8
# name (the single) HMM:            rnase_effectors
# input alignment is asserted as:   protein
# - - - - - - - - - - - - - - - - - - - - - - - - - - - - - - - - - - - -

# idx name                  nseq  alen  mlen eff_nseq re/pos description
#---- -------------------- ----- ----- ----- -------- ------ -----------
1     rnase_effectors        981   727   258    22.67  0.590 

# CPU time: 0.16u 0.01s 00:00:00.17 Elapsed: 00:00:00.16


rnase_effectors-unfiltered_logo
 Warning: Character 'Z' is not in color_dict. Using black.
```

```
rnase_effectors-unfiltered_logo-maxgap0.5
 Warning: Character 'Z' is not in color_dict. Using black.
```

```
rnase_effectors-filtered_logo
```

```
rnase_effectors-filtered_logo-maxgap0.5
```

```
rnase_effectors-filtered_logo_group
```

```
rnase_effectors-filtered_logo_group-maxgap0.5
```

```
rnase_effectors-filtered_logo_group-maxgap0.5minsize10
```

```
rnase_effectors-unfiltered_logo_group
 Warning: Character 'Z' is not in color_dict. Using black.
```

```
rnase_effectors-unfiltered_logo_group-maxgap0.5
 Warning: Character 'Z' is not in color_dict. Using black.
```

```
rnase_effectors-unfiltered_logo_group-maxgap0.5minsize10
 Warning: Character 'Z' is not in color_dict. Using black.
```

```
rnase_effectors-unfiltered_markov
 Warning: Character 'Z' is not in color_dict. Using black.
```

```
---------------------------------------------------------------------------
ValueError                                Traceback (most recent call last)
<ipython-input-165-2cc8b87cc160> in <module>
      9     rnase_effectors_greedy_cmap,
     10     run_fastree=True,
---> 11     show=True,
     12 )

<ipython-input-45-faf363bbac27> in get_plots(name, index, hmms, msas, unfiltered_dfs, filtered_dfs, markov_cmap, greedy_cmap, outdir, palette, logo_palette, run_fastree, show)
     86         max_gap=1,
     87         show=show,
---> 88         outdir=outdir,
     89     )
     90 

<ipython-input-44-2c72ccb9f635> in alnlogo(name, df, greedy_cmap, markov_cmap, alignment_col, max_gap, cpalette, logo_palette, base_width, width_per_base, base_height, height_per_seq, hratio, wratio, outdir, show)
    166 
    167     fig.savefig(os.path.join(outdir, f"{name}-alignment_logo.svg"))
--> 168     fig.savefig(os.path.join(outdir, f"{name}-alignment_logo.png"), dpi=600)
    169 
    170     if show:

~/.miniconda/envs/remote_homology/lib/python3.7/site-packages/matplotlib/figure.py in savefig(self, fname, transparent, **kwargs)
   2201             self.patch.set_visible(frameon)
   2202 
-> 2203         self.canvas.print_figure(fname, **kwargs)
   2204 
   2205         if frameon:

~/.miniconda/envs/remote_homology/lib/python3.7/site-packages/matplotlib/backend_bases.py in print_figure(self, filename, dpi, facecolor, edgecolor, orientation, format, bbox_inches, **kwargs)
   2124                     orientation=orientation,
   2125                     bbox_inches_restore=_bbox_inches_restore,
-> 2126                     **kwargs)
   2127             finally:
   2128                 if bbox_inches and restore_bbox:

~/.miniconda/envs/remote_homology/lib/python3.7/site-packages/matplotlib/backends/backend_agg.py in print_png(self, filename_or_obj, metadata, pil_kwargs, *args, **kwargs)
    512         }
    513 
--> 514         FigureCanvasAgg.draw(self)
    515         if pil_kwargs is not None:
    516             from PIL import Image

~/.miniconda/envs/remote_homology/lib/python3.7/site-packages/matplotlib/backends/backend_agg.py in draw(self)
    386         Draw the figure using the renderer.
    387         """
--> 388         self.renderer = self.get_renderer(cleared=True)
    389         # Acquire a lock on the shared font cache.
    390         with RendererAgg.lock, \

~/.miniconda/envs/remote_homology/lib/python3.7/site-packages/matplotlib/backends/backend_agg.py in get_renderer(self, cleared)
    402                           and getattr(self, "_lastKey", None) == key)
    403         if not reuse_renderer:
--> 404             self.renderer = RendererAgg(w, h, self.figure.dpi)
    405             self._lastKey = key
    406         elif cleared:

~/.miniconda/envs/remote_homology/lib/python3.7/site-packages/matplotlib/backends/backend_agg.py in __init__(self, width, height, dpi)
     90         self.width = width
     91         self.height = height
---> 92         self._renderer = _RendererAgg(int(width), int(height), dpi)
     93         self._filter_renderers = []
     94 

ValueError: Image size of 73110x12066 pixels is too large. It must be less than 2^16 in each direction.
```

In [166]:

```
alnlogo(
    "figure-rnase_effectors-gt0.5",
    rnase_effectors_filtered_dfs[0],
    rnase_effectors_greedy_cmap,
    rnase_effectors_markov_cmap,
    max_gap=0.5,
    outdir=OUTDIR,
    width_per_base=0.05,
    wratio=[0.01, 0.01, 0.01, 0.01, 1],
    hratio=[0.1, 1]
)
```

In [167]:

```
alnlogo(
    "figure-rnase_effectors",
    rnase_effectors_filtered_dfs[0],
    rnase_effectors_greedy_cmap,
    rnase_effectors_markov_cmap,
    max_gap=1,
    outdir=OUTDIR,
    width_per_base=0.05,
    wratio=[0.01, 0.01, 0.01, 0.01, 1],
    hratio=[0.1, 1]
)
```

In [170]:

```
plot_logo_group(
    rnase_effectors_filtered_dfs[0],
    "figure-rnase_effectors-maxgap0.5",
    rnase_effectors_markov_cmap,
    max_gap=0.5,
    min_size=10,
    outdir=OUTDIR,
    width_per_res=0.09,
    height=1.5,
)
```

In [169]:

```
plot_logo_group(
    rnase_effectors_filtered_dfs[0],
    "figure-rnase_effectors",
    rnase_effectors_markov_cmap,
    min_size=10,
    outdir=OUTDIR,
    width_per_res=0.09,
    height=1.5,
)
```

In [ ]:

In [ ]:

In [ ]:

In [171]:

```
ralph_markov = set(eff_with_unique.loc[eff_with_unique["effectors"].notnull(), "supercluster_markov"])
ralph_greedy = set(eff_with_unique.loc[eff_with_unique["effectors"].notnull(), "supercluster_greedy"])
ralph_df = eff_with_unique[
    (eff_with_unique["supercluster_component"] == "PC_032CKH") & \
    (
        eff_with_unique["supercluster_markov"].isin(rnase_effectors_markov) | \
         eff_with_unique["supercluster_greedy"].isin(rnase_effectors_greedy)
    ) & \
    (eff_with_unique["supercluster_markov"] != "PC_032CKH") & \
    (eff_with_unique["supercluster_greedy"] != "PC_032CKH")
]
ralph_df.head()
```

Out[171]:

|  | supercluster\_component | supercluster\_markov | supercluster\_greedy | cluster | effectors | phibase | organisms | lca\_rank | lca | cluster\_size | cluster\_length | member |
| --- | --- | --- | --- | --- | --- | --- | --- | --- | --- | --- | --- | --- |
| 181868 | PC\_032CKH | PC\_00595C | PC\_07TVM3 | PC\_00YPBK | NaN | NaN | Blumeria graminis f. sp. hordei DH14;Blumeria ... | species | Blumeria graminis | 8 | 146 | PC\_00YPBK |
| 181869 | PC\_032CKH | PC\_00595C | PC\_07TVM3 | PC\_00YPBK | NaN | NaN | Blumeria graminis f. sp. hordei DH14;Blumeria ... | species | Blumeria graminis | 8 | 146 | PC\_00HQ7Y |
| 181870 | PC\_032CKH | PC\_00595C | PC\_07TVM3 | PC\_00YPBK | NaN | NaN | Blumeria graminis f. sp. hordei DH14;Blumeria ... | species | Blumeria graminis | 8 | 146 | PC\_05K218 |
| 181871 | PC\_032CKH | PC\_00595C | PC\_07TVM3 | PC\_00YPBK | NaN | NaN | Blumeria graminis f. sp. hordei DH14;Blumeria ... | species | Blumeria graminis | 8 | 146 | PC\_010C61 |
| 181872 | PC\_032CKH | PC\_00595C | PC\_07TVM3 | PC\_00YPBK | NaN | NaN | Blumeria graminis f. sp. hordei DH14;Blumeria ... | species | Blumeria graminis | 8 | 146 | PC\_04ASWU |

In [ ]:

In [172]:

```
ralph_hmms, ralph_msas, ralph_unfiltered_dfs, ralph_filtered_dfs, ralph_markov_cmap, ralph_greedy_cmap = runit(
    "ralph",
    G,
    ralph_df,
    enriched,
    unenriched,
)
```

```
markov
```

```
greedy
```

```
markov mst
```

```
greedy mst
```

```
there are 1 alignments. Each with 157 sequences.
# hmmbuild :: profile HMM construction from multiple sequence alignments
# HMMER 3.3 (Nov 2019); http://hmmer.org/
# Copyright (C) 2019 Howard Hughes Medical Institute.
# Freely distributed under the BSD open source license.
# - - - - - - - - - - - - - - - - - - - - - - - - - - - - - - - - - - - -
# input alignment file:             /tmp/tmpezvjq0w5
# output HMM file:                  /tmp/tmp06ihbtiu
# name (the single) HMM:            ralph
# input alignment is asserted as:   protein
# - - - - - - - - - - - - - - - - - - - - - - - - - - - - - - - - - - - -

# idx name                  nseq  alen  mlen eff_nseq re/pos description
#---- -------------------- ----- ----- ----- -------- ------ -----------
1     ralph                  157   197   118    11.55  0.590 

# CPU time: 0.05u 0.01s 00:00:00.06 Elapsed: 00:00:00.06
```

In [173]:

```
get_plots(
    "ralph",
    0,
    ralph_hmms,
    ralph_msas,
    ralph_unfiltered_dfs,
    ralph_filtered_dfs,
    ralph_markov_cmap,
    ralph_greedy_cmap,
    run_fastree=True,
    show=True,
)
```

```
# hmmbuild :: profile HMM construction from multiple sequence alignments
# HMMER 3.3 (Nov 2019); http://hmmer.org/
# Copyright (C) 2019 Howard Hughes Medical Institute.
# Freely distributed under the BSD open source license.
# - - - - - - - - - - - - - - - - - - - - - - - - - - - - - - - - - - - -
# input alignment file:             /tmp/tmpym_3ystc
# output HMM file:                  /tmp/tmpax1xk0bu
# name (the single) HMM:            ralph
# input alignment is asserted as:   protein
# - - - - - - - - - - - - - - - - - - - - - - - - - - - - - - - - - - - -

# idx name                  nseq  alen  mlen eff_nseq re/pos description
#---- -------------------- ----- ----- ----- -------- ------ -----------
1     ralph                  192   321   170    11.59  0.590 

# CPU time: 0.08u 0.00s 00:00:00.08 Elapsed: 00:00:00.08


ralph-unfiltered_logo
```

```
ralph-unfiltered_logo-maxgap0.5
```

```
ralph-filtered_logo
```

```
ralph-filtered_logo-maxgap0.5
```

```
ralph-filtered_logo_group
```

```
ralph-filtered_logo_group-maxgap0.5
```

```
ralph-filtered_logo_group-maxgap0.5minsize10
```

```
ralph-unfiltered_logo_group
```

```
ralph-unfiltered_logo_group-maxgap0.5
```

```
ralph-unfiltered_logo_group-maxgap0.5minsize10
```

```
ralph-unfiltered_markov
```

```
ralph-unfiltered_greedy
```

```
ralph-filtered_markov
```

```
ralph-filtered_greedy
```

```
ralph-unfiltered_markov-maxgap0.5
```

```
ralph-unfiltered_greedy-maxgap0.5
```

```
ralph-filtered_markov-maxgap0.5
```

```
ralph-filtered_greedy-maxgap0.5
```

In [174]:

```
alnlogo(
    "figure-ralph-maxgap0.5",
    ralph_filtered_dfs[0],
    ralph_greedy_cmap,
    ralph_markov_cmap,
    max_gap=0.5,
    outdir=OUTDIR,
    width_per_base=0.05,
    wratio=[0.01, 0.01, 0.01, 0.01, 1],
    hratio=[0.1, 1]
)
```

In [175]:

```
plot_logo_group(
    ralph_filtered_dfs[0],
    "figure-ralph-maxgap0.5",
    ralph_markov_cmap,
    max_gap=0.5,
    min_size=10,
    outdir=OUTDIR,
    width_per_res=0.09,
    height=1.5,
)
```

In [ ]:

## Get HMMs for all of the markov clusters.¶

We'd like to distribute something that people can use from this work.
So here we'll generate a slightly refined MSA and HMM for clusters and superclusters containing effectors.

I'll use the markov clusters because from my observations, they more consistently grouped related content than greedy.
For singleton clusters we just do the regular cluster MSA (no supercluster). I'll also plot some logos and get a tree.

In [190]:

```
markov_clusters = eff_with_unique.loc[eff_with_unique["effectors"].notnull() & eff_with_unique["supercluster_markov"].notnull(), "supercluster_markov"].unique()
markov_clusters[:5]
```

Out[190]:

```
array(['PC_001EWQ', 'PC_03822Z', 'PC_00D94K', 'PC_00DXJA', 'PC_00GZGP'],
      dtype=object)
```

In [191]:

```
effector_clusters = eff_with_unique.loc[eff_with_unique["effectors"].notnull() & eff_with_unique["supercluster_markov"].isnull(), "cluster"].unique()
effector_clusters[:5]
```

Out[191]:

```
array(['PC_002QUS', 'PC_006B95', 'PC_00ARQD', 'PC_01AXRJ', 'PC_01HQ36'],
      dtype=object)
```

In [220]:

```
def get_cluster_hmms(
    name,
    kind,
    G,
    df,
    enriched,
    unenriched,
    base_width=0.5,
    width_per_res=0.1,
    height=2.0,
    font="DejaVu Sans",
    outdir=OUTDIR,
    logo_palette=LOGO_PALETTE
):
    os.makedirs(outdir, exist_ok=True)
    
    print("Running", name, kind)
    print(repr(set(df.loc[df["effectors"].notnull(), "effectors"].unique())))

    name = f"{kind}-{name}"
    hmms, msas, unfiltered_dfs, filtered_dfs, markov_cmap, greedy_cmap = runit(
        name,
        G,
        df,
        enriched,
        unenriched,
        outdir=outdir,
    )

    index = 0
    hmm = hmms[index]
    msa = msas[index]
    unfiltered_df = unfiltered_dfs[index]
    filtered_df = filtered_dfs[index]

    filtered_df.to_csv(os.path.join(outdir, f"{name}-filtered.tsv"), sep="\t", na_rep=".")
    unfiltered_df.to_csv(os.path.join(outdir, f"{name}-unfiltered.tsv"), sep="\t", na_rep=".")

    with open(os.path.join(outdir, f"{name}_enriched.hmm"), "wb") as handle:
        handle.write(hmm)

    filtered_msa = hhfilter(msa)
    with open(os.path.join(outdir, f"{name}.a3m"), "wb") as handle:
        handle.write(filtered_msa)

    filtered_hmm = hmmbuild(filtered_msa, name=name, verbose=True)
    with open(os.path.join(outdir, f"{name}.hmm"), "wb") as handle:
        handle.write(filtered_hmm)

    fas = a3m_to_fas(msa)
    with open(os.path.join(outdir, f"{name}.fasta"), "wb") as handle:
        handle.write(fas)

    #nwk = fasttree(fas)
    #with open(os.path.join(outdir, f"{name}.nwk"), "wb") as handle:
    #    handle.write(nwk)

    residues, indel = hmmlogo(filtered_msa)
    residues.reset_index(drop=True, inplace=True)
    
    fig, ax = plt.subplots(figsize=(base_width + width_per_res * len(residues), height))
    l = lm.Logo(residues, color_scheme=logo_palette, ax=ax, font_name=font)
    l.style_spines(spines=("top", "right"), visible=False)
    ax.set_xlim(-1, len(residues))

    fig.savefig(os.path.join(outdir, f"{name}-logo.svg"))
    fig.savefig(os.path.join(outdir, f"{name}-logo.png"), dpi=600)
    plt.show()
    plt.close()
    return
```

In [236]:

```
for cl in markov_clusters:
    o = pjoin(OUTDIR, "hmms")
    if os.path.exists(pjoin(o, f"markov-{cl}-logo.png")):
        print("skipping", cl)
        continue

    try:
        get_cluster_hmms(
            cl,
            "markov",
            G,
            eff_with_unique[eff_with_unique["supercluster_markov"] == cl],
            enriched,
            unenriched,
            outdir=pjoin(OUTDIR, "hmms"),
        )
    except ValueError:
        print("FAILED", cl)
```

```
skipping PC_001EWQ
skipping PC_03822Z
skipping PC_00D94K
skipping PC_00DXJA
skipping PC_00GZGP
skipping PC_00HH7V
skipping PC_059FW5
skipping PC_00NROE
skipping PC_00PLZF
skipping PC_00PY60
skipping PC_00ZF7V
skipping PC_011E13
skipping PC_0144K8
skipping PC_014PA9
skipping PC_016E7M
skipping PC_01ANVM
skipping PC_01DKY2
skipping PC_0094J7
skipping PC_01OETC
skipping PC_01OWJN
skipping PC_043ENH
skipping PC_022BD9
skipping PC_01T1MD
skipping PC_02ALJ9
skipping PC_02BMLW
skipping PC_02EHK0
skipping PC_017Q4B
skipping PC_02J8YC
skipping PC_02LJVJ
skipping PC_02O8L1
skipping PC_044YDJ
skipping PC_02MM79
skipping PC_02W1ZZ
skipping PC_021PXP
skipping PC_02ZN8N
skipping PC_030GYR
skipping PC_01D3OM
skipping PC_0278ZT
skipping PC_032CKH
skipping PC_04SK9M
skipping PC_01UOBI
skipping PC_03BSKP
skipping PC_03WSEZ
skipping PC_03PRB1
skipping PC_05OM83
skipping PC_03VR5X
skipping PC_03ZG0S
skipping PC_01NRLX
skipping PC_045SAO
skipping PC_0206OW
skipping PC_04E9WQ
skipping PC_04IDXX
skipping PC_01REOO
skipping PC_03HV2H
skipping PC_04YPRX
skipping PC_02XUK5
skipping PC_03DUMB
skipping PC_009H7U
skipping PC_00TPLE
skipping PC_05F15E
skipping PC_01204B
skipping PC_02VR38
skipping PC_03MDGJ
skipping PC_05PCSX
skipping PC_06O7HH
skipping PC_05UP4Q
skipping PC_05VEPP
Running PC_00RQZW markov
{'PGTG_16791'}
markov
```

```
greedy
```

```
markov mst
```

```
greedy mst
```

```
there are 1 alignments. Each with 348 sequences.
# hmmbuild :: profile HMM construction from multiple sequence alignments
# HMMER 3.3 (Nov 2019); http://hmmer.org/
# Copyright (C) 2019 Howard Hughes Medical Institute.
# Freely distributed under the BSD open source license.
# - - - - - - - - - - - - - - - - - - - - - - - - - - - - - - - - - - - -
# input alignment file:             /tmp/tmpbgutzxfi
# output HMM file:                  /tmp/tmp70n3lguj
# name (the single) HMM:            markov-PC_00RQZW
# input alignment is asserted as:   protein
# - - - - - - - - - - - - - - - - - - - - - - - - - - - - - - - - - - - -

# idx name                  nseq  alen  mlen eff_nseq re/pos description
#---- -------------------- ----- ----- ----- -------- ------ -----------
1     markov-PC_00RQZW       348  1281   870     4.48  0.590 

# CPU time: 0.44u 0.00s 00:00:00.44 Elapsed: 00:00:00.44


# hmmbuild :: profile HMM construction from multiple sequence alignments
# HMMER 3.3 (Nov 2019); http://hmmer.org/
# Copyright (C) 2019 Howard Hughes Medical Institute.
# Freely distributed under the BSD open source license.
# - - - - - - - - - - - - - - - - - - - - - - - - - - - - - - - - - - - -
# input alignment file:             /tmp/tmp_yj6p6ht
# output HMM file:                  /tmp/tmpdbm1z_5i
# name (the single) HMM:            markov-PC_00RQZW
# input alignment is asserted as:   protein
# - - - - - - - - - - - - - - - - - - - - - - - - - - - - - - - - - - - -

# idx name                  nseq  alen  mlen eff_nseq re/pos description
#---- -------------------- ----- ----- ----- -------- ------ -----------
1     markov-PC_00RQZW       320  2405  1221     6.14  0.590 

# CPU time: 0.60u 0.01s 00:00:00.61 Elapsed: 00:00:00.60


FAILED PC_00RQZW
skipping PC_069DIK
skipping PC_00VO2T
skipping PC_029W2K
skipping PC_02OZ0I
skipping PC_02URNV
skipping PC_0334QE
skipping PC_040O1D
skipping PC_04U5QD
skipping PC_04XZ1N
skipping PC_05C7YR
skipping PC_05CAN0
skipping PC_07ZZBE
skipping PC_081AOA
skipping PC_083GBU
skipping PC_04NETZ
skipping PC_016RN5
skipping PC_06QDZ9
skipping PC_03LC2L
skipping PC_06S6A1
skipping PC_07HELN
skipping PC_058FSP
skipping PC_016T2Z
skipping PC_07OLPP
skipping PC_00DO78
skipping PC_07QFFP
skipping PC_07R7MF
skipping PC_06AYNY
skipping PC_0832GJ
skipping PC_05JXM3
skipping PC_07QGEE
Running PC_03SKNA markov
{'Cgfl'}
markov
```

```
greedy
```

```
markov mst
```

```
greedy mst
```

```
there are 1 alignments. Each with 233 sequences.
# hmmbuild :: profile HMM construction from multiple sequence alignments
# HMMER 3.3 (Nov 2019); http://hmmer.org/
# Copyright (C) 2019 Howard Hughes Medical Institute.
# Freely distributed under the BSD open source license.
# - - - - - - - - - - - - - - - - - - - - - - - - - - - - - - - - - - - -
# input alignment file:             /tmp/tmp46bw4nmr
# output HMM file:                  /tmp/tmpo7mcjnze
# name (the single) HMM:            markov-PC_03SKNA
# input alignment is asserted as:   protein
# - - - - - - - - - - - - - - - - - - - - - - - - - - - - - - - - - - - -

# idx name                  nseq  alen  mlen eff_nseq re/pos description
#---- -------------------- ----- ----- ----- -------- ------ -----------
1     markov-PC_03SKNA       233   378   297     4.70  0.590 

# CPU time: 0.14u 0.00s 00:00:00.14 Elapsed: 00:00:00.14


# hmmbuild :: profile HMM construction from multiple sequence alignments
# HMMER 3.3 (Nov 2019); http://hmmer.org/
# Copyright (C) 2019 Howard Hughes Medical Institute.
# Freely distributed under the BSD open source license.
# - - - - - - - - - - - - - - - - - - - - - - - - - - - - - - - - - - - -
# input alignment file:             /tmp/tmpq8kpunws
# output HMM file:                  /tmp/tmp55h7hxfd
# name (the single) HMM:            markov-PC_03SKNA
# input alignment is asserted as:   protein
# - - - - - - - - - - - - - - - - - - - - - - - - - - - - - - - - - - - -

# idx name                  nseq  alen  mlen eff_nseq re/pos description
#---- -------------------- ----- ----- ----- -------- ------ -----------
1     markov-PC_03SKNA       959  3342  1228    11.41  0.590 

# CPU time: 0.74u 0.01s 00:00:00.75 Elapsed: 00:00:00.75


FAILED PC_03SKNA
skipping PC_02LHT3
skipping PC_08JJK6
skipping PC_08JMY0
skipping PC_07O88I
skipping PC_07OCEM
```

In [237]:

```
import numpy as np
```

In [238]:

```
markov_clusters[60:70]
```

Out[238]:

```
array(['PC_01204B', 'PC_02VR38', 'PC_03MDGJ', 'PC_05PCSX', 'PC_06O7HH',
       'PC_05UP4Q', 'PC_05VEPP', 'PC_00RQZW', 'PC_069DIK', 'PC_00VO2T'],
      dtype=object)
```

In [241]:

```
def get_singleton_hmms(
    name,
    kind,
    df,
    enriched,
    unenriched,
    base_width=0.5,
    width_per_res=0.1,
    height=2.0,
    font="DejaVu Sans",
    outdir=OUTDIR,
    logo_palette=LOGO_PALETTE
):
    os.makedirs(outdir, exist_ok=True)
    enriched_msa = enriched[name.encode()][:-1]
    unenriched_msa = unenriched[name.encode()][:-1]
    
    
    print("Running", name, kind)
    print(repr(set(df.loc[df["effectors"].notnull(), "effectors"].unique())))

    name = f"{kind}-{name}"
    index = 0
    
    hmm = hmmbuild(enriched_msa, name=name, verbose=True)
    msa = clustalo(hmm, unenriched_msa)
    
    with open(os.path.join(outdir, f"{name}_enriched.hmm"), "wb") as handle:
        handle.write(hmm)

    filtered_msa = hhfilter(msa)
    with open(os.path.join(outdir, f"{name}.a3m"), "wb") as handle:
        handle.write(filtered_msa)

    filtered_hmm = hmmbuild(filtered_msa, name=name, verbose=True)
    with open(os.path.join(outdir, f"{name}.hmm"), "wb") as handle:
        handle.write(filtered_hmm)

    fas = a3m_to_fas(msa)
    with open(os.path.join(outdir, f"{name}.fasta"), "wb") as handle:
        handle.write(fas)

    #nwk = fasttree(fas)
    #with open(os.path.join(outdir, f"{name}.nwk"), "wb") as handle:
    #    handle.write(nwk)

    residues, indel = hmmlogo(filtered_msa)
    residues.reset_index(inplace=True, drop=True)
    
    fig, ax = plt.subplots(figsize=(base_width + width_per_res * len(residues), height))
    l = lm.Logo(residues, color_scheme=logo_palette, ax=ax, font_name=font)
    l.style_spines(spines=("top", "right"), visible=False)
    ax.set_xlim(-1, len(residues))

    fig.savefig(os.path.join(outdir, f"{name}-logo.svg"))
    fig.savefig(os.path.join(outdir, f"{name}-logo.png"), dpi=600)
    plt.show()
    plt.close()
    return
```

In [ ]:

```
for cl in effector_clusters:
    o = pjoin(OUTDIR, "hmms")
    if os.path.exists(pjoin(o, f"cluster-{cl}-logo.png")):
        print("skipping", cl)
        continue

    if cl == "PC_023OAC":
        continue
    if cl == "PC_02LO13":
        continue
    if cl == "PC_03K6QP":
        continue

    try:
        get_singleton_hmms(
            cl,
            "cluster",
            eff_with_unique[eff_with_unique["cluster"] == cl],
            enriched,
            unenriched,
            outdir=pjoin(OUTDIR, "hmms"),
        )
    except ValueError:
        print("FAILED", cl)
```

```
skipping PC_002QUS
skipping PC_006B95
skipping PC_00ARQD
skipping PC_01AXRJ
skipping PC_01HQ36
skipping PC_01QMA6
skipping PC_01S0NS
skipping PC_01W4F6
skipping PC_01ZZX9
skipping PC_02741Z
skipping PC_027WVR
skipping PC_02H4U1
skipping PC_02HCCM
skipping PC_02QUH1
skipping PC_02RYKY
skipping PC_0394OJ
skipping PC_039FX8
skipping PC_039XF3
skipping PC_03B2V7
skipping PC_03G6PA
skipping PC_03SZXO
skipping PC_03TV1W
skipping PC_048H5Z
skipping PC_049VOF
skipping PC_04B4O8
skipping PC_04PSKU
Running PC_04VK6L cluster
{'CfAvr5'}
# hmmbuild :: profile HMM construction from multiple sequence alignments
# HMMER 3.3 (Nov 2019); http://hmmer.org/
# Copyright (C) 2019 Howard Hughes Medical Institute.
# Freely distributed under the BSD open source license.
# - - - - - - - - - - - - - - - - - - - - - - - - - - - - - - - - - - - -
# input alignment file:             /tmp/tmpa4gelfwc
# output HMM file:                  /tmp/tmpzn524s_0
# name (the single) HMM:            cluster-PC_04VK6L
# input alignment is asserted as:   protein
# - - - - - - - - - - - - - - - - - - - - - - - - - - - - - - - - - - - -

# idx name                  nseq  alen  mlen eff_nseq re/pos description
#---- -------------------- ----- ----- ----- -------- ------ -----------
1     cluster-PC_04VK6L        3   103   103     0.35  0.587 

# CPU time: 0.05u 0.00s 00:00:00.05 Elapsed: 00:00:00.05
```

In [ ]:

```
print("hi")
```

In [ ]:
